# Proteomics, Lipidomics, Metabolomics, and 16S DNA Sequencing of Dental Plaque From Patients With Diabetes and Periodontal Disease

**DOI:** 10.1016/j.mcpro.2021.100126

**Published:** 2021-07-29

**Authors:** Katherine A. Overmyer, Timothy W. Rhoads, Anna E. Merrill, Zhan Ye, Michael S. Westphall, Amit Acharya, Sanjay K. Shukla, Joshua J. Coon

**Affiliations:** 1 Morgridge Institute for Research, Madison, Wisconsin, USA; 2 Department of Biomolecular Chemistry, University of Wisconsin–Madison, Madison, Wisconsin, USA; 3 National Center for Quantitative Biology of Complex Systems, Madison, Wisconsin, USA; 4 Department of Chemistry, University of Wisconsin–Madison, Madison, Wisconsin, USA; 5 Center for Precision Medicine Research, Marshfield Clinic Research Institute, Marshfield, Wisconsin, USA; 6 Center for Oral and Systemic Health, Marshfield Clinic, Marshfield, Wisconsin, USA

**Keywords:** oral microbome, multi-omics, MS, lipidomics, metaproteomics, AGC, automatic gain control, FDR, false discovery rate, GO, gene ontology, HbA1c, glycated hemoglobin, OTU, operational taxonomic unit, PC, phosphatidylcholine, PD, periodontal disease, PE, phosphatidylethanolamine, PE-NMe, monomethyl phosphatidylethanolamine, PG, phosphatidylglycerol, Pre-DM/DM, prediabetes/diabetes, TGs, triglycerides

## Abstract

Oral microbiome influences human health, specifically prediabetes and type 2 diabetes (Pre-DM/DM) and periodontal diseases (PDs), through complex microbial interactions. To explore these relations, we performed 16S rDNA sequencing, metabolomics, lipidomics, and proteomics analyses on supragingival dental plaque collected from individuals with Pre-DM/DM (n = 39), Pre-DM/DM and PD (n = 37), PD alone (n = 11), or neither (n = 10). We identified on average 2790 operational taxonomic units and 2025 microbial and host proteins per sample and quantified 110 metabolites and 415 lipids. Plaque samples from Pre-DM/DM patients contained higher abundance of *Fusobacterium* and *Tannerella* than plaques from metabolically healthy patients. Phosphatidylcholines, plasmenyl phosphatidylcholines, ceramides containing non-OH fatty acids, and host proteins related to actin filament rearrangement were elevated in plaques from PD *versus* non-PD samples. Cross-omic correlation analysis enabled the detection of a strong association between *Lautropia* and monomethyl phosphatidylethanolamine (PE-NMe), which is striking because synthesis of PE-NMe is uncommon in oral bacteria. Lipidomics analysis of *in vitro* cultures of *Lautropia mirabilis* confirmed the synthesis of PE-NMe by the bacteria. This comprehensive analysis revealed a novel microbial metabolic pathway and significant associations of host-derived proteins with PD.

## Highlights

• Patients with periodontal disease or diabetes have unique microbial dysbiosis.
• Proteomics and 16S data provide complementary information about microbial diversity.
• Cross-omic correlation reveals host signatures associated with periodontal disease.
• Multi-omic data lead to finding about microbially synthesized lipids.

## Graphical Abstract

**Figure:**
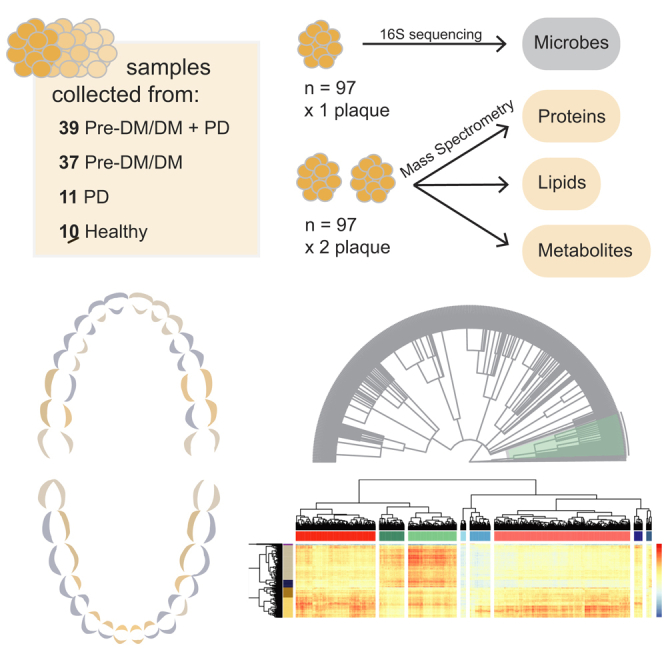

## In Brief

The human oral cavity houses a complex ecosystem of microbes, some of which have pathogenic influence on the host. Multi-omics analysis of oral plaques revealed key players in microbial communities derived from diabetic and periodontal disease patients. With cross-omic correlation analysis, we found host-specific proteins and associated lipids that were elevated in plaques from periodontal disease patients. Furthermore, this multi-omic approach leads to the finding that oral community member *Lautropia mirabilis* synthesizes monomethyl phosphatidylethanolamine, an uncommon lipid in oral microbiota.

The human oral cavity harbors a wide variety of microbes, over 700 species (1), and has some of the highest microbial diversity observed in humans (2). These oral-associated bacteria reside in saliva, on the tongue and cheeks, and in biofilms on tooth surface and under the gum lining (2). The development of plaque biofilms is particularly important to the etiology of oral diseases, such as tooth decay and periodontal disease (PD) (3, 4). And importantly, the pathogenic oral microbiota that contribute to the progression of PD are also correlated with systemic diseases, including diabetes, arthritis, and heart disease (5, 6, 7), suggesting that oral microbial ecologies have a broad impact on human health, and a better understanding of pathogenesis and host–microbe interactions will be essential for mitigating negative effects of pathogenic microbiota.

One such negative impact of these microbiota is the increased burden of PD in diabetics (8). PD begins with gum inflammation that progresses to permanent tooth and bone loss if untreated (9). Bacterial populations accumulate, and the progressive shifts in the plaque biofilm diversity are strongly associated with PD incidence and severity (4, 10). In particular, species that form what is called the ‘red complex’, *Tannerella forsythia*, *Porphyromonas gingivalis*, and *Treponema denticola*, are associated with gum bleeding on probing and probe depth, two common markers of PD severity (10). These red complex microbial populations are observed in conjunction with species such as *Fusobacterium nucleatum*, *Prevotella intermedia*, *Prevotella nigrescens*, and *Peptostreptococcus micros* species, which are more mildly associated with PD (10) and often are observed in biofilms before the red complex. Diabetics, especially those with poor glycemic control, are at a higher risk for developing PD (11), and higher glycated hemoglobin (HbA1c) values are associated with increased prevalence of red complex microbiota (12). Importantly, the relationship between PD and diabetes is bidirectional (8), and treating PD has been shown to improve HbA1c values of diabetics (13). Thus, better understanding on microbial populations and their microenvironment in diabetics with and without PD is an important goal toward mitigating the negative consequences of these two diseases.

During the development of dental plaque, increases in microbial diversity are mediated by changes in the microenvironment and manifestation of microbial niches (14, 15), as the local environment becomes optimal to support population growth. This growth can be aided by microbe–microbe interactions, host–microbe interactions, and metabolite availability (4). As dental plaque biofilms become established, measurable changes in microbial abundances as well as metabolites and host factors occur. Thus, a holistic approach to studying dental plaque could provide insight into how microbial populations interact and how they associate with host health.

Frequently, 16S/18S rDNA sequencing is used to estimate the size and diversity of microbial populations (1, 2); however, this method offers little information about microbial function or local environmental factors, although researchers have come up with ways to use 16S sequencing to deduce some functional information (*i.e.*, PICRUSt) (16). More recently, metagenomics and metatranscriptomics approaches have afforded greater evidence for microbial functional potential, that is, what genes are present and/or expressed in the population (17). These methods can provide clues to how microbes might interact within the biofilm, but importantly, these conclusions are greatly strengthened by biomolecule measurements, for example, when metagenomics is paired with metabolomics (18, 19). MS-based ‘omic analyses—such as metabolomics, lipidomics, and proteomics—uniquely offer high-throughput means of assessing the molecular details of the local environmental niches these microbes occupy as well as information about community composition and functional-level information. Indeed, several studies have used MS-based ‘omics to assess the oral microbiome, focusing on either metaproteomics (20, 21) or metabolomics (22, 23, 24, 25). As of yet, discovery lipidomics is not being applied to study the oral microbiome, and generally, the use of multi-omics for studying the oral microbiome is still uncommon (25), despite the fact that it has potential to offer a wealth of information about the local microbe–host environment (26).

We leveraged 16S rRNA gene sequencing and high-resolution MS to profile microbes, proteins, lipids, and metabolites in human dental plaques from nearly 100 individuals with PD and/or prediabetes/diabetes (Pre-DM/DM). Altogether, we performed >650 GC or LC-MS/MS experiments, collected >4.5 million tandem mass spectra and on average identified several thousand biomolecules in each sample. Using ecology diversity metrics, we identified increases in microbial diversity and changes in the microbial populations with PD and Pre-DM/DM, suggestive of microbial dysbiosis occurring with these diseases. Furthermore, we identify hundreds of proteins, metabolites, and lipids associated with PD and/or Pre-DM/DM. Based on these findings, we describe a rare lipid synthesis pathway in one of the common oral bacteria (*Lautropia mirabilis*) and demonstrate that these compounds along with other microbial-molecule associations can link microbial populations to function.

## Experimental Procedures

### Materials

Unless otherwise stated, materials were obtained from Sigma-Aldrich. Organic solvents and water used for extraction and MS analysis were of MS-grade quality.

### Experimental Design and Statistical Rationale

The study design consisted of 39 individuals with Pre-DM/DM, 37 individuals with Pre-DM/DM and PD, 11 individuals with PD, and ten individuals with neither Pre-DM/DM nor PD. The study design was such that the age and sex were similar among the four groups. Each patient contributed three randomly sampled dental plaques to the study, two samples from each patient were reserved for MS analysis and one sample was reserved for 16S rDNA sequencing. The 16S rDNA data were acquired with a single analysis for each sample (no technical or biological replicates). For the two biological replicates designated for MS analysis (*i.e.*, same individual, different tooth locations)—metabolites, lipids, and proteins were extracted from each sample, leading to two biological replicates for the acquired MS data. The samples analyzed by MS were analyzed in batches of n = 25 with a cultured mixed-microbial quality control sample in each batch. Each batch consisted of equal proportions of the individuals from the study design groups (∼40% Pre-DM/DM, ∼40% Pre-DM/DM with PD, ∼10% PD, and ∼10% neither Pre-DM/DM nor PD). In addition, biological replicates of individuals were processed on separate days and analyzed in separate batches. The mixed microbe quality control samples served as a technical replicate for extractions (equivalent pellets extracted with each batch) and also served as a technical replicate for lipidomics and GC-MS-based metabolomics analysis (repeated injection of batch’s quality control extract at regular intervals during the analysis). The quality control samples were used for guiding normalization and filtering features. For statistical analysis, we sought to evaluate effect of PD and Pre-DM/DM on measured biomolecules. Given the unbalanced study design, these effects were evaluated separately and as interaction terms using generalized additive mixed-effect models. The models also including confounding factors of age, gender, and tobacco-use status, and participant identifier were included as a random effect to account for replicate sampling from individuals (in MS-acquired data). Benjamini–Hochberg false discovery rate (FDR) correction was applied to all *p*-values used to assess statistical significance of the effects. Secondary to the investigation of Pre-DM/DM and PD effects on the measured biomolecules, the data were used to evaluate microbial–metabolite–lipid associations. For this approach, correlation analysis was applied with appropriate FDR correction.

### Recruiting Patients and Sample Collection

This study was approved by the IRB of Marshfield Clinic Research Institute under the IRB Protocol # SHU10115. PD and pre-DM/DM patients and healthy controls were recruited from the Marshfield Dental Clinic, Marshfield, WI, based on their prior medical and dental records; in total, 99 participants consented to the study; two patients were later excluded because of having a type I diabetes diagnosis. Participants were classified as Pre-DM/DM if they had been previously diagnosed with diabetes in their medical record or if they met the following criteria: fasting blood glucose 100 mg/dl or greater, HbA1c 5.7% or greater, and glucose tolerance test 140 mg/dl or greater. Patients were classified as having PD if they had undergone a periodontal examination and were classified as having moderate or severe periodontitis. Moderate periodontitis was classified as having either interproximal attachment loss ≥4 mm (two or more teeth) or interproximal probe depth ≥5 mm. Severe periodontitis was classified as having both interproximal attachment loss ≥6 mm (two or more teeth) and interproximal probe depth ≥5 mm. Patients were not required to abstain from food before sample collection. For every participant, supragingival plaque samples were collected from three locations, lower and upper molars and lower anterior lingual surfaces were targeted for collection, sample location was recorded by tooth numbers and surface (palatal *versus* lingual), and samples were frozen in a dry ice–isopropanol bath within 5 min of collection. Samples were maintained at less than −20 °C before analysis.

### 16S rDNA Sequencing

The V4 region of the 16S rRNA gene sequencing was performed by following the protocol published in (27). The Illumina pair-end reads of partial 16S rRNA sequences were used as input for the QIIME analyses (27); the analysis was performed in the following steps. (1) All the pair-end reads were assembled in one fastq file with samples independently tagged with their samples names, basic quality control steps were applied to make sure the quality of the fastq file and the parameters used are default of QIIME pipeline from http://qiime.org/tutorials/index.html. (2) operational taxonomic unit (OTU) picking step was performed using the pick_open_reference_otus.py protocol by searching reads against the Greengenes database with similarity set to 99% (28, 29). (3) Taxonomy assignment was performed using the ‘uclust’ method (30) and a 0.7 confidence cut-off with Greengenes taxonomy assignment (31). (4) Chimeric sequences were detected using the identify_chimeric_seqs.py function with the ‘usearch61’ method (30, 32); these chimeric sequences were removed from the OTU table. The OTU results were exported as a ‘biom’ file and imported into R for further analysis.

### Bacterial Culture Preparation for Lipidomics

Two ATCC strains of *L. mirabilis* (ATCC strain #s 51599 and 51601) and a clinical isolate of *Actinomyces odontolyticus* were grown 5-ml tubes of Mueller–Hinton broth at 37 °C in static culture for 48 h. After 48 h, bacterial cells were centrifuged, the supernatant was discarded, and fresh 5 ml of the Mueller–Hinton broth was added in to tubes, vortexed, and incubated for additional 48 h before collecting the cells pellet. All three cultures were grown in triplicates, and cell pellets were stored at −80 °C before lipid extractions.

### Sample Extraction for MS Analysis

Samples were kept on dry ice before extraction. To each sample, we added 500 μl of ice-cold extraction buffer (2:2:1 methanol–acetonitrile–water). Samples were probe-sonicated for 10 s over ice and then centrifuged for 5 min at 14,000*g* at 4 °C to pellet protein and other debris. The supernatant was centrifuged again at 14,000*g* for 5 min at 4 °C to ensure no precipitate was formed. The extract was divided for LC-MS–based lipidomics and GC-MS–based metabolomics analyses and dried by vacuum centrifugation. The precipitated protein was used for proteome analysis.

### GC Metabolomics

Dried extracts were resuspended in methoxyamine HCl (20 mg/ml in pyridine) and incubated at room temperature (RT) for 90 min. Samples were further derivatized at with MSTFA (Restek) for 30 min at 37 °C. Samples were analyzed on a Q Exactive GC-Orbitrap mass spectrometer using a TraceGOLD TG-5SilMS GC column (33, 34). Samples were injected using a 1:10 split at 275 °C and ionized using electron impact. The GC gradient ranged from 50 to 320 °C, linear over a 25 min gradient, and then a 4.4 min hold at 320 °C. Orbitrap MS acquisitions were collected in full-scan mode 50 to 650 m/z at a resolution of 30,000 ( at 200 m/z). Raw files were analyzed using an in-house tool for deconvolution of spectra, quantitation, and identification against in-house and NIST 2014 libraries (35, 36) (see also, https://github.com/coongroup/Y3K-GC-Quantitation-Software). Quantification is based on a feature’s quant ion apex intensity (peak height).

### LC Lipidomics

Dried extracts were resuspended in 65:30:5 isopropanol–acetonitrile–water. For each sample, 10 μl was injected onto a Water’s Acquity UPLC CSH C18 Column (2.1 mm × 100 mm) with a 5-mm VanGuard precolumn using a Vanquish Split Sampler HT autosampler (Thermo Scientific). Mobile phase A: 70:30 acetonitrile–water, 10 mM NH4Ac, and 0.025% acetic acid. Mobile phase B: 90:10 IPA–acetonitrile, 10 mM NH4Ac, and 0.025% acetic acid. The samples were run on a 30 min gradient with 400 μl/min flow rate: mobile phase B was maintained at 2% for 2 min, then increased to 30% over 3 min, then increased to 50% over 1 min, then increased to 85% over 14 min, and finally brought up to 99% over 1 min. Mobile phase B was held at 99% for 7 min, and then the column was equilibrated with mobile phase B at 2% for 1.75 min before the next injection. Mass spectra were acquired using a Thermo Focus Q-Exactive with polarity switching and top-2 data-dependent ms2 scans. Both full and ms/ms scans were acquired with a resolving power of 17,500. From 0 to 2 min, negative-mode scans were acquired with a mass range of 70 to 750 Da, and then, from 2 to 30 min, negative-mode full scans were acquired with a mass range of 200 to 1600 Da. Positive-mode full scans were acquired with a mass range of 200 to 1600 Da from 0 to 30 min. Full-scan MS were acquired with automatic gain control (AGC) targets set to 1e6 and a max injection time of 100 ms. MS/MS spectra were collected with AGC targets of 1e5, maximum injection time of 50 ms, 1.0 m/z isolation width, scan range of 200 to 2000, and normalized stepped collision energies of 20, 30, and 40. A 10-s dynamic exclusion was used. The source conditions were |4.0| kV for both positive and negative modes, 320 °C capillary temperature, 25 units sheath gas, 10 units aux gas, and 0 units sweep gas.

Lipidomics raw files were analyzed with the Thermo Compound Discoverer 2.0 application. Spectra from 2 to 21 min, 200 to 1600 Da mass, 1e5 signal threshold, and signal-to-noise (S/N) threshold of 1.5 were selected for alignment. Alignment of retention times was allowed a maximum shift of 0.2 min and a 10 ppm mass tolerance. Compounds were detected with a mass tolerance of 10 ppm, an intensity tolerance threshold of 10, an S/N threshold of 10, a maximum peak width of 0.5 min, a minimum peak intensity threshold of 1e5, a minimum of seven scans per peak, and minimum of three isotopes. Detected compounds were grouped with a 10-ppm tolerance and 0.2-min retention time tolerance. Gap filling was used with 10-ppm tolerance and 1.5 S/N. Compounds less than 10-fold greater than the solvent blank were marked at background features and removed. Retention time aligned compound tables containing integrated peak area for each feature and unaligned tables from Compound Discoverer, and mgf converted raw files were input into LipiDex software for lipid identification (37). MS/MS spectra were searched against an *in silico*–generated lipid spectral library (LipiDex library: Coon_Lab_HCD_Acetate). Spectral matches were filtered based on dot product score greater than 500 and a reverse dot product score greater than 700. Coeluting isobaric species with greater than 75% spectral interference were collapsed into a lipid with sum acyl-chain composition identification. Identifications outside a 3.5 median absolute retention time deviation of other lipids of the same class were excluded, and identifications found in less than two raw files were also excluded.

### LC Proteomics

Precipitated protein was solubilized in 8 M urea prepared in 50 mM Tris, pH 8.0. Proteins were then reduced and alkylated with TCEP (10 mM final) and 2-chloroacetamide (40 mM final) for 15 min at RT, with shaking. Samples were diluted with 50 mM Tris, pH 8, to a final 4 M urea concentration, then proteins were digested overnight with endoproteinase Lys-C (1:100 enzyme–protein, Wako Pure Chemical Industries). Samples were desalted with C18 Sep-Pak columns (Waters); peptides were then dried down and resuspended in 0.2% formic acid. Peptide concentration was estimated using a peptide colorimetric assay (Pierce), and 1 μg of peptides was analyzed by LC-MS/MS using an in-house packed nano-LC column (1.7-μm particle size, BEH C18, Waters) coupled to a Thermo Orbitrap Elite with a nano-electrospray ionization source and heated column compartment (38). The gradient mobile phases consisted of 0.2% formic acid (A) and 0.2% formic acid in 100% acetonitrile (B). The gradient consisted of ramp from 0 to 70% B over 80 min, followed by a re-equilibration at 0% B, with a total run time of 110 min. The MS was operated in a positive mode using a spray voltage of 2.5 kV, and S-lens RF of 57.1%, and a capillary temperature of 275 °C. Full-scan MS were acquired at 60,000 resolving power using a Fourier transform mass spectrometer, with 50 ms maximum injection times, 300 to 1500 m/z range, and 1e6 AGC target. Data-dependent MS/MS (top 15) were acquired by Fourier transform mass spectrometer with 15,000 resolving power, 100 ms maximum injection times, 5e6 AGC target, higher-energy collisional dissociation with 30 normalized collision energy, 0.1 s activation time, fixed first mass value of 115 *m/z*, 500 minimal signal, and a 2 *m/z* isolation width. The mass spectrometer was operated with monoisotopic precursor selection enabled, and with 45-s dynamic exclusions (±10 ppm).

Raw files were searched against a concatenated database containing peptides form the Human Oral Microbiome Database (02/2016) (39) and peptides in the human UniProt database (including isoforms) using a two-step search strategy (40); this combined database included a total of 1,329,621 entries. First, the initial search was completed individually on each sample using COMPASS (41), then we combined all first search identification matches to create a reduced fasta database, a total of 759,208 sequences, for a second search using MaxQuant (version v.1.5.6.0) (42). The search parameters were as follows: carbamidomethyl (C) fixed modification, oxidation (M), and acetyl (protein N-term) variable modifications, a 4.5-ppm peptide mass tolerance, a 20-ppm MS/MS fragment mass tolerance, minimum spectral scores of 0 for unmodified spectra and 40 for modified peptides, peptide spectral match FDR of 0.01, protein FDR of 0.01, minimum peptide length of 7, and a minimum number of peptides (razor) of 1, and ‘use only unmodified peptides’ was set to true. Match between runs was used with a 0.7-min matching time window and a 20-min alignment time window. *In silico* spectral libraries were generated with the LysC/P enzyme specificity, and a maximum of two missed cleavages were allowed.

MaxQuant’s resulting label-free quantification values were used for protein quantification; for proteins quantified by one peptide, annotated spectra were generated using the interactive peptide spectral annotator (43) (Supplemental data). Identified peptide sequences were queried against NCBI’s NR database (protein blast, v-2.4.0+) (44), and resulting hits were filtered using MEGAN6 (45) to assign lowest common ancestor to each peptide, which we then assembled into functional and taxonomy assignment at the protein groups level generated by the MaxQuant algorithm.

### Data Analysis

Data were analyzed using the R statistical and graphing environment (46). Normalization for batch effects were done with ComBat (47). For statistical analysis, we modeled the effect of diabetes and PD on the abundance of each molecule with generalized additive mixed-effect models using R package GAMLSS (48). We used models with fixed effects for diabetes, PD, interaction between diabetes and PD, and confounding factors of age, gender, and tobacco-use status. To account for replicate sampling from individuals (in MS-acquired data), we included participant identifier as a random effect in the models. Owing to the differences in analysis paradigms, we chose different data distributions to best fit the data: zero-adjusted Gamma distribution (16S data and proteomics), log normal distribution (lipidomics), and bimodal log normal distribution (metabolomics), due to imputed values of low-level features We evaluated significance of Pre-DM/DM and PD on our models with log-likelihood ratio testing (Equations 1 *versus* 2, and Equations 1 *versus* 3, respectively) and Benjamini–Hochberg FDR correction. The interaction between DM and PD was also evaluated (Equations 1 *versus* 4). For analysis of microbial diversity, we used R package vegan (49), and for plotting heat maps, we used pheatmap (50).

(1) moleculeabundance∼DM+PD+DM:PD+Age+Sex+Tobaccouse+1|Individual

(2) moleculeabundance∼PD+Age+Sex+Tobaccouse+1|Individual

(3) moleculeabundance∼DM+Age+Sex+Tobaccouse+1|Individual

(4) moleculeabundance∼DM:PD+Age+Sex+Tobaccouse+1|Individual

For linking proteins to additional metadata, MEGAN6 and database mapping files were used; these are available online at “https://software-ab.informatik.uni-tuebingen.de/download/megan6/welcome.html”. Protein UniProt IDs and eggNOG terms were then used for mapping gene ontology (GO) terms; this was done through web portals “https://www.ebi.ac.uk/QuickGO/services/annotation/” and http://eggnogapi.embl.de/nog_data/, respectively.

## Results

**Figure 1:**
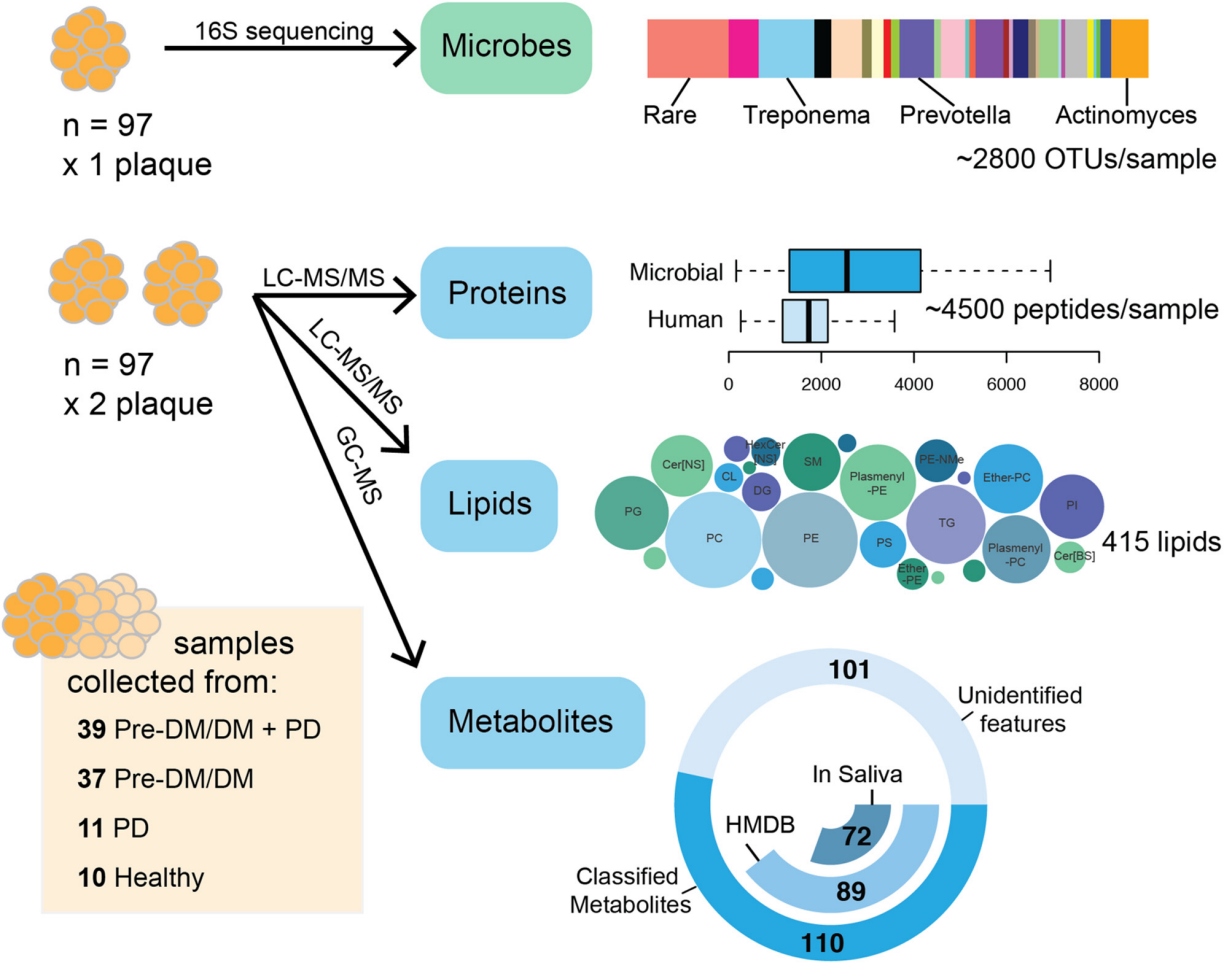
**Sample collection and processing strategy for the microbiome, proteome, lipidome, and metabolome analyses.** Patients were classified by prediabetes/diabetes (Pre-DM/DM) and periodontal disease (PD) status. One plaque sample was used for 16S rRNA sequencing to generate a list of operational taxonomic units (OTUs), and two plaque samples were used for MS-based analyses, proteomics, lipidomics, and metabolomics, which led to the identification of ~4500 peptides, 415 lipids, and 126 metabolites per sample, respectively.

We collected three supragingival plaque samples from buccal and palatal tooth surfaces from each of the 97 study participants (Pre-DM/DM, n = 39; Pre-DM/DM with PD, n = 37; PD, n = 11; or neither, n = 10, Table 1). Participants were recruited during an already scheduled clinical visit and were not given instructions to fast or preform any strict hygiene procedures before the visit; as such, these samples capture a typical state of the participant’s oral environment. The study participants were primarily non-Hispanic white (93%) and nonsmokers (84%); the Pre-DM/DM participants were significantly older than the metabolically healthy participants (62 ± 16 *versus* 43 ± 16 years old, *p* < 0.001), Table 1. One plaque sample was used for 16S rDNA sequencing, and two plaque samples were used for MS-based analyses–proteomics, lipidomics, and metabolomics (Fig. 1). For 16S sequencing, DNA sequences are easily amplified to enhance the quantitative signal, and data processing methods for determining the features present (*i.e.*, microbial populations) are well established. In contrast, for the MS-based approaches, two major challenges—limited sample amount and feature identification—still persist. To address them, we maximized our limited samples by extracting several compound classes (small molecules, lipids, and proteins) from a single plaque sample and used comprehensive libraries of standards and databases to annotate our raw data (>650 raw files). This methodology enabled detection of 50,752 OTUs by rRNA sequencing (99% sequence similarity; ∼ 2790/sample), 12,346 annotated protein groups (∼2025 proteins/sample), 415 lipids, and 89 metabolites (supplemental Table S1 and Fig. 1), making this study the most comprehensive analysis of dental plaque to date.

**Table 1:** Patient population statistics

Group	N	Sex (female/male)	Age^a^ Years Mean (SD)	Race/ethnicity		HbA1c^a^ % Mean (SD)	Fasting blood glucose^a^ mg/dl Mean (SD)	Periodontal disease (moderate/severe)	Tobacco use (current/former)
				White	Hispanic				
Pre-DM/DM + PD	39	(22/17)	61.3 (15.7)	37	3	7.1 (2.1)	126.0 (45.4)	(35/4)	(11/10)
Pre-DM/DM	37	(20/17)	64.1 (16.3)	36	1	6.7 (1.6)	126.1 (37.9)	(0/0)	(3/17)
PD	11	(9/2)	45.2 (15.9)	10	0	4.7 (0.2)	91.3 (6.6)	(9/2)	(2/6)
Healthy (non-PD/non-DM)	10	(8/2)	39.6 (16.0)	10	0	4.9 (0.1)	91.2 (6.1)	(0/0)	(0/4)

Patients were grouped by pre-diabetes/diabetes (Pre-DM/DM) and periodontal disease (PD) status. Pre-DM/DM patients were significantly older and had higher HbA1c and fasting blood glucose (*p* < 0.05).

a *p* < 0.05, Pre-DM/DM *versus* non–Pre-DM/DM.

To permit further informatic analysis, we assembled the dataset by first normalizing lipidomics and metabolomics data to account for sample-size variation and then applying batch normalization (47). Upon quality assessment, we removed two proteomic samples because of the low number of identified features. Finally, as we structured our statistical analysis to compare the plaque composition in healthy *versus* PD or Pre-DM/DM samples, we accounted for confounding variables, such as age, sex, and tobacco use. This analysis was done using log-likelihood ratio testing between generalized additive mixed effects models comparing models with and without the variable of interest (PD or Pre_DM/DM, see Experimental Procedures). In addition to deciphering features associated with the disease, we assessed microbiota diversity using both 16S rRNA–based taxonomy and proteomics-based taxonomy, determined molecular composition of the plaque biofilms, and clustered co-occurring molecules to infer molecular associations.

### Microbial Populations Show Unique Dysbiosis With PD and Pre-DM/DM

**Figure 2:**
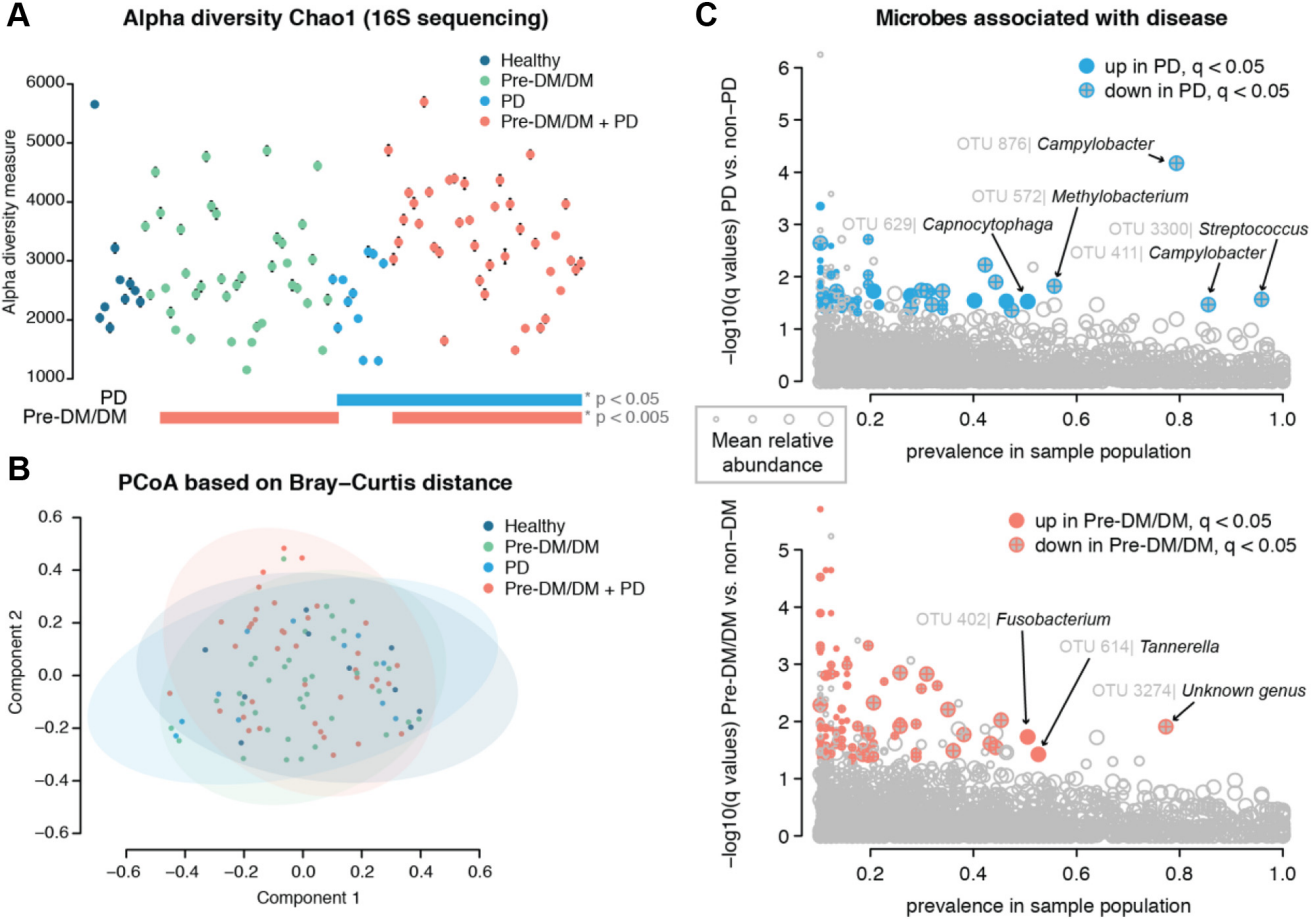
**Diversity of microbial populations is similar across patient plaque samples.** Patients’ plaque microbial communities were assessed by 16S rRNA sequencing. The Chao1 index was varied across patients (*A*) and were significantly different between patients with periodontal disease (PD) *versus* nonperiodontal disease and prediabetics/diabetics (Pre-DM/DM) *versus* nondiabetics (log-likelihood ratio test, *p* = 0.03 and *p* = 0.004, respectively). The Bray–Curtis distance for measuring beta diversity showed no significant difference between groups (*B*). When specific operational taxonomic units (OTUs) were assessed for association with PD (*C*, above) or Pre-DM/DM (*C*, below), we found several OTUs that were detected in a majority of our samples (prevalence in sample population > 50%) that also had q-values of <0.05 and log2 fold-change greater than 1 (up) or less than −1 (down) in disease *versus* nondisease.

Across our samples, we observed hundreds of common OTUs (256, >75% of patients) and numerous rare OTUs (50,496, <10% of patients). We used these to calculate standard ecology diversity metrics. The diversity within a sample, known as alpha diversity, varied across the plaque samples (Fig. 2*A*) and was greater with disease states (log-likelihood ratio test PD *versus* non-PD, *p* = 0.03, and Pre-DM/DM *versus* metabolically healthy, *p* = 0.004). Similarly, we assessed the diversity across samples or beta diversity using the Bray–Curtis distance (Fig. 2*B*). In contrast to alpha diversity, beta diversity was less associated with the diseases, as we observed a small but significant effect with Pre-DM/DM on beta diversity (permutation MANOVA, R^2^ = 0.017, *p* = 0.03). These data are consistent with previous publications reporting that biodiversity tends to be elevated in oral disease (51, 52, 53).

To establish which microbial species were significantly associated with disease, we performed linear regression analysis on OTUs observed in >10% of the samples (n = 4169, 3048 with genus assignment, 29 with genus and species assignment). We filtered our results based on disease significance, log_2_ fold-change (Log_2_FC), and prevalence in the sample population (Fig. 2*C* and supplemental Table S1). This data filtering led us to identification of 56 microbial species that were significantly different between PD and non-PD samples and 36 that were significantly different between Pre-DM/DM and metabolically healthy conditions. In PD, we observed a lower relative abundance of several *Streptococcus* spp., *Campylobacter* spp., *Actinomyces* spp., and *Methylobacterium* sp. Lower levels of *Streptococcus* spp. in PD have been reported previously (54, 55), and the loss of these genera is believed to play a role in disease progression by creating space for more pathogenic bacteria to thrive (54). We also observed higher abundance of *Capnocytophaga* in plaques of PD patients (Fig. 2*C* and supplemental Table S1). In contrast to other studies which have specifically focused on microbial measurements from the plaque at sites of periodontitis (10), these data were collected from supragingival regions and not specifically at affected tooth sites (supplemental Fig. S1). Thus, microbial populations that are typically higher in PD-affected tooth sites—red complex bacteria *T. forsythus*, *P. gingivalis*, and *T. denticola*—were not necessarily expected to be elevated in these samples.

Comparing Pre-DM/DM *versus* metabolically healthy group, we identified 36 significantly different OTUs (Fig. 2*D* and supplemental Table S1). Specifically, microbes belonging to *Fusobacterium* and *Tannerella* genera—classic periodontal pathogens (10)—were elevated in Pre-DM/MD. In agreement with our findings, a recent study reported that red complex genera, which includes *Tannerella*, were especially elevated on healthy tooth sites in DM *versus* non-DM (12). The elevation of these pathogenic bacteria in supragingival plaque could indicate an overall dysbiosis in DM, potentially increasing propensity for PD. Overall, our data support the hypothesis that dysbiosis occurs in both PD and Pre-DM/DM and importantly that the microbial changes that take place in either disease state are distinct.

### Metabolites, Lipids, and Proteins Found in Plaque Are of Human and Microbial Origins

To further characterize the composition of the oral plaques, we looked to our MS-based ‘omics data. We measured a diverse array of biomolecules, including amino acids, monosaccharides, phospholipids, triglycerides (TGs), and human and microbial proteins (Fig. 1). For each of the MS-based ‘omics, intrapatient abundance measurements were more similar than interpatient ones (supplemental Fig. S2). As expected, the identified compounds were likely of both human and microbial origins. A majority of the metabolites identified by GC-MS had been previously identified in saliva (88%, 72 of 89 compounds), as annotated in the Human Metabolomics Database (56, 57). The remaining 17 compounds had been detected in human feces or were annotated as human cellular metabolites, indicating a probable human, microbial, or food origin.

The lipids consisted of phospholipids—primarily phosphatidylcholine (PC), phosphatidylethanolamine (PE), and phosphatidylglycerol (PG)—as well as TGs and ceramides. Approximately 28% of the lipids contained odd-chain fatty acyl tails (supplemental Fig. S3), which are more commonly found in bacteria than eukaryotic cells (58). The percentage of odd-chain acyl tails was higher in the PGs (∼45%) than in other lipids, likely because PGs are also more common in bacterial than in mammalian membranes (59).

Finally, the proteins identified in the plaques belonged to various taxonomic branches. Nine percent were from eukaryotic taxonomic branches, and likely human in origin, and 15% were unassigned or assigned to the root taxonomy level (supplemental Table S1). A majority of the proteins were from various bacterial genera, including *Actinomyces*, *Corynebacterium*, *Leptotrichia*, *Capnocytophaga*, and *Prevotella* (top-5 genera based on representative proteins). *Actinomyces* and *Corynebacterium* were previously found to contribute to a majority of the oral biofilm proteome (60), suggesting that these genera indeed make up a majority of plaque proteins.

### Proteomics and 16S Data Provide Complementary Information About Microbial Diversity

Similar to 16S data, proteomics data provided insight into microbial diversity and relative abundance of genera. According to proteomics measurements, alpha diversity tended to be higher in plaques from PD and Pre-DM/DM than in heathy patients—a similar finding to what was observed with 16S data. After accounting for confounders (sex, age, and smoking status), this effect was, however, reduced (supplemental Fig. S4). Beta diversity showed no significant effect with respect to PD or Pre-DM/DM. Overall, the proteomics data resulted in similar trends, but with lesser effect sizes, to those observed in the 16S data.

**Figure 3:**
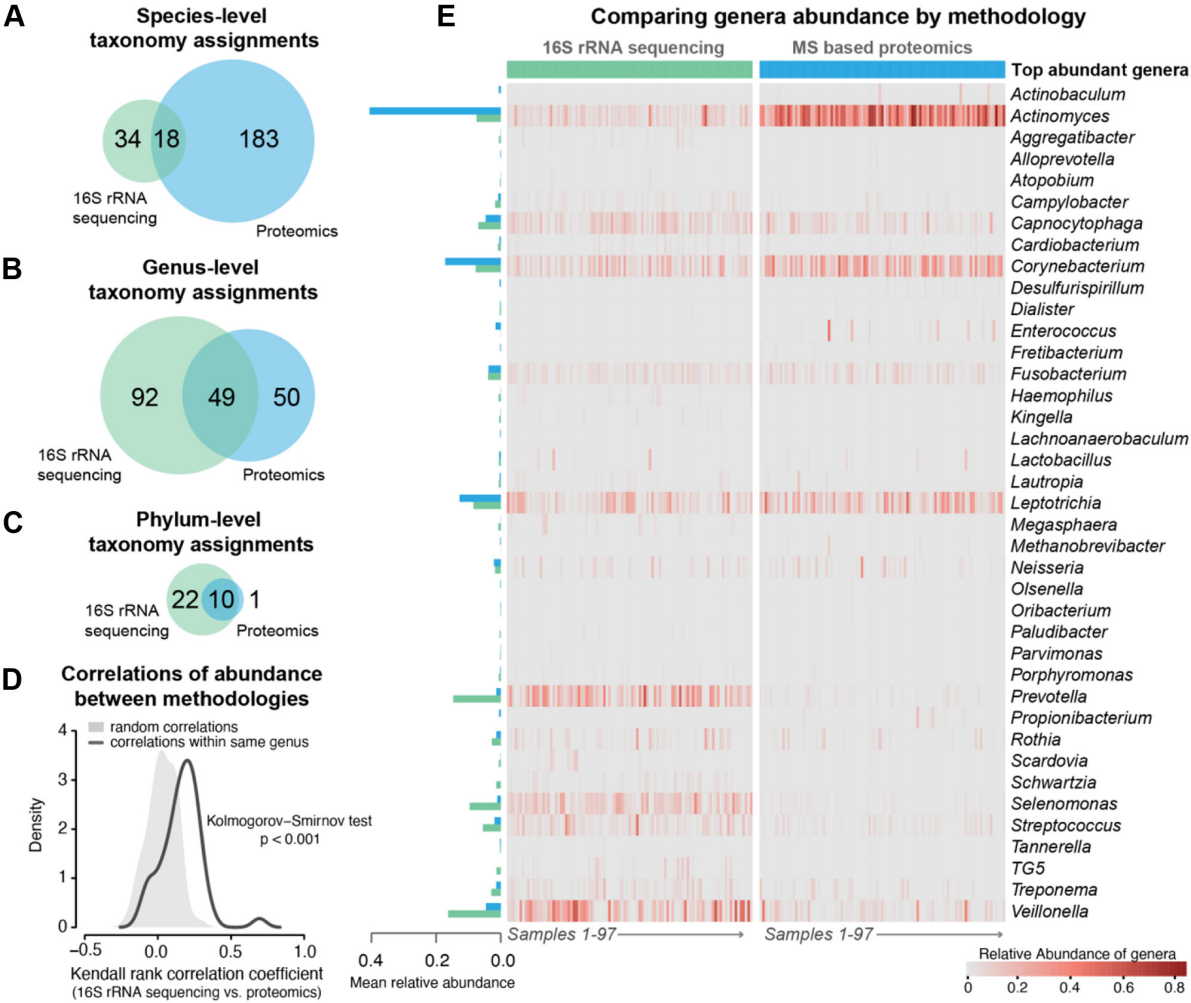
**Proteomics and 16S sequencing approaches result in similar taxonomic assignment across patient samples.** Taxonomy assignment resulted in 52 species, 141 genera, and 31 phyla by 16S rDNA sequencing and 101 species, 99 genera, and 11 phyla by proteomics, with 18 species, 49 genera, and ten phyla in common (*A*–*C*). Correlation between abundance of genera by 16S rDNA sequencing *versus* proteomics within individuals was better than expected by chance alone (*D*). The top-abundance genera showed good overlap (*E*), except *Actinomyces* and *Corynebacterium* were found in greater abundance by proteomics, while *Prevotella*, *Selenomonas*, *Streptococcus*, and *Veillonella* were found in greater abundance by 16S rDNA sequencing.

Next, we compared how well OTU-based bacterial identification matched to the bacterial identification determined by proteomics. We calculated species, genus, and phylum overlap (18, 49, and 10, respectively, Fig. 3, *A*–*C*). Notably, proteomics allowed for greater species-level resolution (n = 101 species level assignment, bacterial only) than 16S sequencing (n = 52 species level assignments). This lack of species level resolution is a well-known caveat of using 16S for understanding bacterial populations (61); thus, for comparing across the 16S data and the proteomics data, we used genus-level relative abundance measurements (Fig. 3, *D* and *E*). Note that different taxonomic ontologies were used for 16S data (Greengenes (31)) and proteomics (NCBI taxonomy (62)), and we, therefore, expected differences in taxonomy assignments (63). Despite this, we observed considerable overlap (∼34%) between all detected genera and even greater similarity for common genera (∼89% overlap between genera detected in >90% samples). In addition, abundance measurements at the genus level exhibited good agreement, as the observed within-individual correlation of abundance of genera between 16S data and proteomics was better than expected by chance alone (Fig. 3*C*, *p* < 0.001). We directly compared the most abundant genera detected by the two methods across our samples (mean relative abundance >0.1%, Fig. 3*D*); the notable differences between the methods were greater relative abundance of *Actinomyces* and *Corynebacterium* in proteomics *versus* 16S data and greater relative abundance of *Prevotella*, *Selenomonas*, *Streptococcus*, and *Veillonella* in 16S *versus* proteomics data. This discrepancy in abundance estimations could be due to differences in sampling locations (supplemental Fig. S1), relative higher expression of proteins in some genera in oral environment, or a product of the analytical technique and normalization strategy (relative abundance of count-based data *versus* peptide *m/z* signal intensity); however, there is some precedence for microbial differences in proteomics *versus* 16S data. For example, Belda-Ferre *et al.* (60). also noted higher relative abundance of *Actinomyces* and *Corynebacterium* in dental plaque as measured by metaproteomics than what was observed with sequencing-based approaches. Yet, another potential explanation of discrepancy could microbial size differences leading to different gene–protein ratios (64), relative copy number of 16S rRNA gene in a bacterium (65), and lack of species-level resolution in a genus by partial 16S rDNA sequences (66). These observations are intriguing and merit further exploration with a simultaneous sample preparation strategy (as was done with the MS-based data acquisition in this study).

### Plaques From PD Patients Contained Elevated Human-Derived Proteins, PCs, and Amino Acids

**Figure 4:**
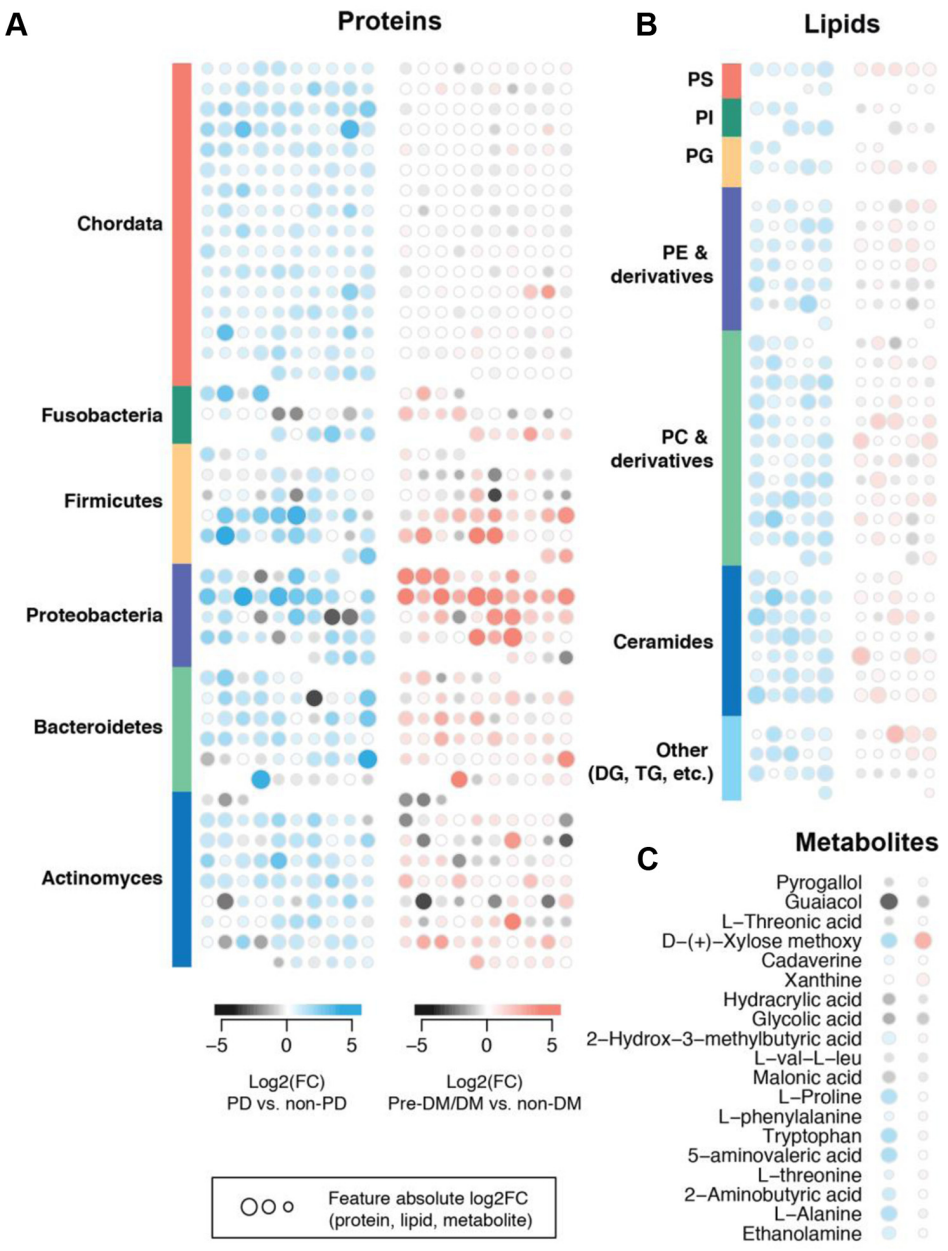
**Comparison of biomolecule abundance changes occurring with periodontal disease (PD) and pre-diabetes/diabetes (Pre-DM/DM).** Each symbol represents a unique protein (*A*), lipid (*B*), or metabolite (*C*). Proteins are grouped by phylum (*A*) and lipids are grouped by lipid class (*B*). DG, diacylglycerides; DM, diabetes; PC, phosphatidylcholine; PD, periodontal disease; PE, phosphatidylethanolamine; PG, phosphatidylglycerol; PI, phosphatidylinositol; PS, phosphatidylserine; TG, triglycerides.

We found 234 proteins, 76 lipids, and six metabolites that exhibited significant associations with PD, and 46 proteins, six lipids, and one metabolite that significantly associated with Pre-DM/DM (q-values < 0.05, supplemental Table S1 and Fig. 4).

For the plaque proteins that were associated with either PD or Pre-DM/DM (234 and 46, respectively), we assessed protein enrichment for certain taxonomic classes or functional pathways (kyoto encyclopedia of genes and genomes, and GO). The proteins that were elevated in plaque from patients with PD were enriched in proteins from genera *Oribacterium* (q-value < 0.05) and *Homo* (q-value < 0.01). PD-associated proteins were also more enriched for GO terms for signaling, actin cytoskeleton organization, cell communication, and nuclear factor kappa B transcription factor activity (q-values < 0.002). Proteins that were elevated in plaques from Pre-DM/DM patients were enriched in proteins from *Campylobacter* (q-value < 0.02). Overall, PD coincided with greater detection of human-derived proteins (phylum Chordata) than Pre-DM/DM (Fig. 4*A*) and suggests potential host-factor relevance in PD. Consistently, prior metaproteomics studies found human saliva proteins related to innate immunity to be elevated in periodontitis (21).

Many lipids were elevated in plaques from PD *versus* non-PD (n = 76), and this list was over-represented in PCs (n = 15), plasmenyl PCs (n = 11), and ceramides containing non-OH fatty acids and sphingosines (Cer[NS], n = 11). Some of these lipids include Cer[NS] d18:0_15:0, Cer[NS] d18:1_15:0, PC 39:2, and plasmenyl-PC P-18:0_20:4. Comparing lipids in Pre-DM/DM *versus* metabolically healthy samples, we detected five lipids elevated with Pre-DM/DM (TG 42:1, Cer[NS] d16:0_15:0, ether-PC O-32:1, ether-PC O-40:4, and TG 12:0_12:0_12:0) and one lipid that was lower in Pre-DM/DM (PC 18:2_18:1). Overall, PD resulted in larger changes in plaque PCs and ceramides than Pre-DM/DM (Fig. 4*B*).

Metabolites 5-aminovaleric acid, L-alanine, tryptophan, L-proline, and D-xylose were elevated in PD *versus* non-PD. D-xylose was also elevated in Pre-DM/DM. One metabolite, glycolic acid, was reduced in PD-associated plaques. Comparison of these metabolite changes in PD *versus* Pre-DM/DM is shown in Figure 4*C*; overall, PD resulted in elevated plaque amino acids, which is consistent with prior studies showing elevated amino acids in saliva of PD patients (24, 67, 68).

### Associations Between Lipids, Metabolites, and Proteins Indicate Disease Signatures Related to Actin Filament Rearrangement

**Figure 5:**
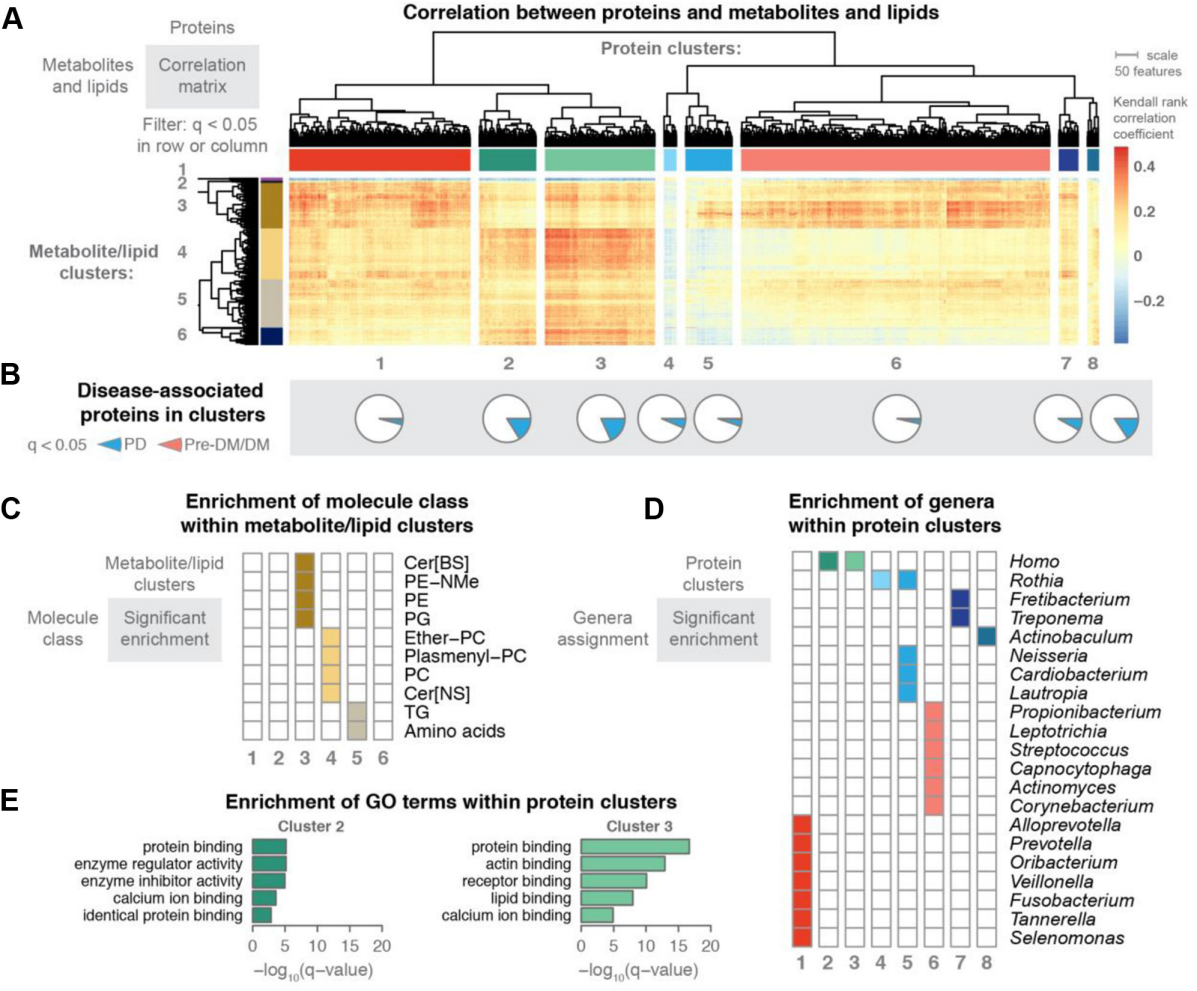
**Metabolite and lipid associations with plaque proteins manifested genus-specific clusters.** MS-acquired data from two plaque samples per patient were used to investigate how metabolite and lipid signatures correlate with plaque proteins. Kendall rank–based correlation was used to filter associations; metabolites, lipid, or proteins with at least one significant association (q < 0.05) are presented in the heat map with hierarchical clustering of rows and columns (*A*). Using k-means (k = 8) to define protein clusters, we observed proteins that were found significantly associated with either PD or pre-DM/DM in each cluster (*B*), but clusters 2 and 3 had higher proportions of disease-associated proteins (23% and 26%, respectively). We used k-means (k = 6) to define metabolite/lipid clusters—these clusters showed enrichment of specific classes of lipids and metabolites (*C*). The protein clusters were enriched for specific genera (*D*). Protein clusters 2 and 3 had significant enrichment for GO terms (*E*). Cer[BS], ceramides containing beta-OH fatty acids and sphingosines; DM, diabetes; GO, gene ontology; PC, phosphatidylcholine; PD, periodontal disease; PE, phosphatidylethanolamine; PE-NMe, monomethyl phosphatidylethanolamine; PG, phosphatidylglycerol; SM, sphingomyelin; TG, triglycerides.

To explore how the detected molecules might relate to the microbial populations, we performed correlation analysis across datasets using the Kendall nonparametric test. We found hundreds of significant correlations and strikingly, distinct clusters of correlations between proteins and metabolites and lipids, indicating that groups of proteins were related to specific metabolite/lipid profiles (Fig. 5*A*). We used hierarchical clustering with k-means k = 8 to define protein clusters and k = 6 to define metabolite/lipid clusters. Several protein clusters (clusters 2 and 3) contained a large portion of the disease-associated proteins that were found in this study (Fig. 5*B*).

To better explore the proteins and metabolites/lipids that composed the clusters, we preformed enrichment analysis (Fig. 5, *C* and *D*). We hypothesized that the protein clusters would contain proteins within related metabolic pathways; however, we found that most clusters were enriched in specific genera, while only a few clusters were enriched for specific pathways (GO biological processes, Fig. 5*E*). This finding suggested that microbial populations, rather than specific pathways, had a stronger association with metabolite and lipid levels. Notably, protein clusters 2 and 3, which had the greater number of disease-associated features that were found in this study, were not enriched in bacterial proteins, but instead were enriched in human-derived proteins. These human-derived proteins were also associated with GO terms related to protein binding (cluster 2 and 3), enzyme regulator activity (cluster 2), actin binding (cluster 3), receptor binding (cluster 3), and lipid binding (cluster 3). Significant elevation of the actin-binding proteins in plaques from PD *versus* non-PD patients is possibly due to bacterial invasion process and the loss of structural integrity at the tooth-endothelial interface (69, 70). These proteins were also strongly correlated with abundance of many plasmenyl-PCs, PCs, and Cer[NS], suggesting these lipids might also be related to the host’s response to disease.

Many of the microbial-associated protein clusters in Figure 5*A* showed positive correlations with lipid/metabolite cluster 3 that was enriched in PE, monomethyl phosphatidylethanolamine (PE-NMe), PG, and Cer[BS] lipids. As noted earlier, the PGs are likely derived from bacterial populations, and these clustering results are consistent with that conclusion. Likewise, PE-NMe are intermediates in the synthesis of PC in bacteria (71) and as such, would be expected to have association with bacterial proteins.

Further, protein cluster 1 was enriched in proteins derived from known oral pathogens: *Prevotella*, *Fusobacterium*, *Tannerella*, and *Selenomonas* genera (10). This large cluster exhibited positive correlations with many metabolites and lipids, *e.g.*, 5-aminovaleric acid, L-homoserine, hydrocinnamic acid, Cer[BS] containing odd-chain fatty-acyl chains, and Plasmenyl-PEs. In particular, 5-aminovaleric acid, a bacterial-derived metabolite generated during degradation of lysine, was positively correlated with proteins from *Selenomonas* and *Fusobacterium* – allowing us to hypothesize that these microbes might produce this metabolite. In general, the association between elevated amino acids and oral pathogens could explain why amino acids are biomarker candidates for PD (67, 68).

Protein Cluster 6, which featured early biofilm colonizers like *Actinomyces*, *Corynebacterium*, and *Streptococcus*, displayed higher correlations with PE-NMe, malic acid, and 2-isopropylmalic acid. 2-isopropylmalic acid was only correlated with *Corynebacterium*, which reassuringly was the only genus with detectable protein levels of the necessary synthetic enzyme 2-isopropylmalic acid synthase (EC 2.3.3.13).

**Figure 6:**
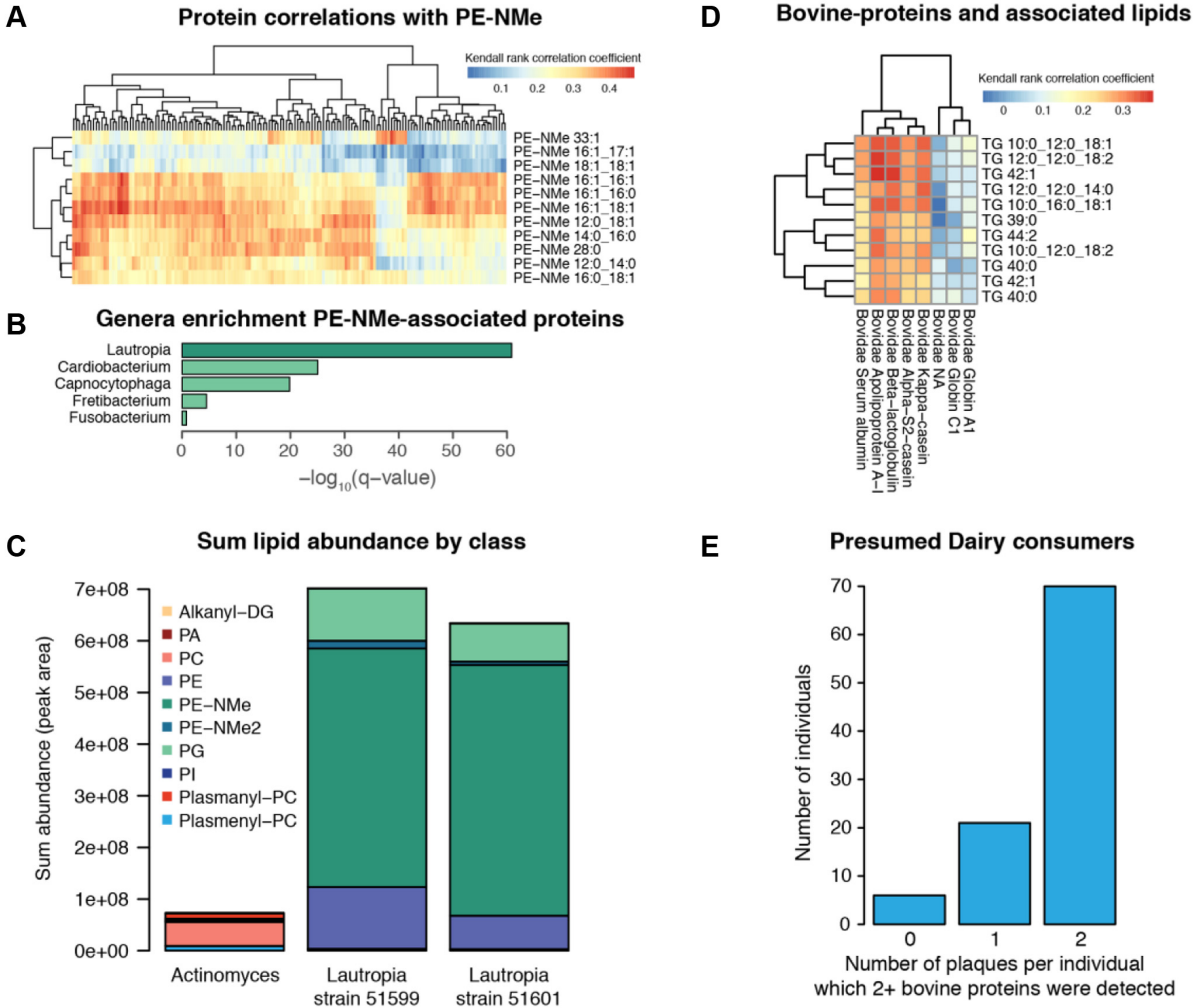
**Lipid–protein associations facilitate observations about food consumption and microbial lipid synthesis pathways.** Following from Figure 5, Kendall rank–based correlation was used to filter associations between metabolites, lipid, and proteins. PE-NMe were strongly associated with many bacterial proteins in protein cluster 6 of Figure 5 (*A*). The PE-NMe–associated proteins were highly enriched for *Lautropia* genera (*B*). Lipidomics profiles from single cultures of *Lautropia mirabilis* strains show PE-NMe are the dominate lipid class in these species (*C*). One small cluster shows strong association between medium-chain-length TG and bovine proteins (taxonomic family Bovidae) (*D*). Greater than 70% of individuals had dairy-associated proteins (2+ bovine proteins observed) in 2/2 plaque samples (*E*). DG, diacylglycerides; PA, phosphatidic acid; PC, phosphatidylcholine; PE, phosphatidylethanolamine; PE-NMe, monomethyl phosphatidylethanolamine; PE-NMe2, dimethyl phosphatidylethanolamine; PG, phosphatidylglycerol; PI, phosphatidylinositol; TG, triglycerides.

PE-NMe have been estimated to occur in only about 10 to 15% of bacteria (58, 72), and in our analysis they were strongly correlated to *Lautropia*, a genus not previously described as synthesizing PE-NMe (Fig. 6, *A* and *B*). To validate this potentially novel finding, we compared lipid levels from two *in vitro* grown strains of *L. mirabilis* to *A. odontolyticus*. *Actinomyces* had demonstrated low correlation to PE-NMe in our analysis, and thus was selected as a control. We confirmed PE-NMe synthesis in *Lautropia* and in fact found that PE-NMe were one of the more abundant lipids in these bacteria (Fig. 6*C*).

Finally, beyond host and microbial lipid associations, we found evidence of diet-associated features. In Protein Cluster 6 we found a lipid-protein association indicative of cow’s milk. One protein (Apolipoprotein A-I, of taxonomic family Bovidae) was positively correlated with several TGs containing medium-chain fatty-acyl tails (TG 12:0_12:0_14:0, TG 10:0_12:0_18:1, TG 10:0_16:0_18:1, TG 10:0_12:0_18:2, TG 12:0_12:0_18:2). The next most closely correlated proteins with these TGs were also assigned to taxonomic family Bovidae (alpha-S2-casein, kappa-casein, and beta-lactoglobulin). Together with APO-A1, these proteins constitute some of the most abundant proteins in cow’s milk (73). We annotated eight proteins to the taxonomic family Bovidae, and five of the eight showed strong correlations to medium chain–containing TGs (Fig. 6*D*). As TGs are also highly abundant in cow’s milk (74), we concluded that this association was likely due to protein–lipid associations indicative of dairy consumption. Over 70% percent of the individuals had detectable levels of two or more of these bovine proteins in both of their plaque samples (Fig. 6*E*).

In summary, the integrative MS-based multi-omics component of this analysis revealed findings beyond those typically seen with sequencing approaches and facilitated discovery of host–disease, microbial–lipid, and diet-induced associations in dental plaques, associations which are critical to furthering our understanding of the human microbiome.

## Discussion

This study provides a comprehensive and comparative analysis of the microbiome, proteome, lipidome, and metabolome of dental plaque samples from individuals with PD and Pre-DM/DM. We detected on average 5277 features per plaque from 97 individuals representing three disease groups and a control group, with over 7% of the detected features having disease associations with either gingival or metabolic health. We demonstrated that microbial dysbiosis occurred with PD and that these changes were distinct from the microbial dysbiosis that resulted from Pre-DM/DM. Specifically, PD patient samples contained reduced levels of *Streptococci* relative to controls, and the Pre-DM/DM patient samples had increased abundance of *Fusobacterium* and *Tannerella*. We compared microbial population estimations obtained *via* 16S sequencing and proteomics. In general, we observed good agreement between the methods; however, some genera had higher estimations by 16S sequencing approaches (*Prevotella*, *Selenomonas*, and *Veillonella*), while other genera had higher estimations by proteomics (*Actinomyces* and *Corynebacterium*). It is apparent that relatively predominant genera present in PD samples may be different than the present in diseases sites such as caries. Predominant oral microbes in individuals with no PDs are *Acinetobacter*, *Haemophilus*, and *Moraxella*, whereas in individuals with PD are *P. gingivalis*, *Tannerella forsythia*, and *T. denticola* (75). Tanner *et al.* (76). showed that caries is associated with acid-tolerant and acid-producing *Actinomyces*, members belonging to the *Actinomyces/Bifidobacterium* family. Predominance of *Streptococci* could be population specific; severity of caries driven by, perhaps, quality of oral care as *Streptococcus mutans*, *Streptococcus sobrinus*, *Streptococcus cristatus*, *Streptococcus australis*, and so forth, have been reported in Romanian children with high caries in contrast to *Streptococcus constellatus* and strains of *Actinomyces* such as HOT, 175, 446, and so forth, in Swedish children with low caries (76). Proteomics of the oral microbiome and therefore its relative abundance in dental plaque is likely to be driven by availability of sugars to the microbes besides other factors. Indeed, Rudney *et al.* (77) have shown that major proteins expressed by caries microcosm grown in a sucrose or sucrose-free medium have yielded different protein profiles associated with sucrose metabolism. Our observation of significant association of the lower abundance of *Methylobacterium* and *Campylobacter* in PD is novel, whereas a higher abundance of *Capnocytophaga* in PD has been observed before (78).

In addition, we used our MS data to correlate plaque proteins with metabolites and lipids. We revealed many microbial–molecule associations and importantly discovered host-specific disease features, such as actin filament–related proteins, which were highly correlated with PC and plasmenyl PCs and strongly associated with PD. In sum, this study provides a data-rich multilayered analysis of the complex ecosystem found in PD and pre-DM/DM.

One of our findings was the observation of unique dysbiosis occurring with PD and Pre-DM/DM. Although PD is often a comorbidity of DM (6, 12), we established that the supragingival microbial populations were distinct between PD and DM. In patients with PD, we observed lower relative abundance of *Streptococci*, a result consistent with the previous reports on reduced abundance of specific *Streptococcus* spp. in PD (54, 55). This loss of *Streptococci* has been suggested to contribute to disease progression by freeing space for more pathogenic bacteria to thrive (54). In this study, we did not specifically sample plaques from diseased tooth sites; thus, it is possible this loss of more neutral bacteria is wide spread in mouths of PD patients. Glycated hemoglobin or HbA1c is formed when glucose from blood reacts with the amino group on the hemoglobin and forming a ketoamine. The nexus between the type 2 diabetes, HbA1c, and oral pathogens associated with periodontitis and DM is beginning to emerge with certain groups of oral pathogens that seem to thrive better in above-normal HbA1c levels (79, 80). However, this complex interaction is likely to be modulated by microbial competition, their ability to utilize available sugars, and by the host genetic susceptibility. In patients with Pre-DM/DM, we observed elevated levels of periodontopathogenic pathogens, *Fusobacterium* and *Tannerella*, which also have been detected in other DM and non-DM obese populations (12, 81). Specifically, Aemaimanan *et al.* (12). reported on higher populations of these pathogens at healthy tooth sites in DM than those in patients with PD alone and that those microbial populations were correlated with HbA1c values, commonly used to monitor long-term glycemic control. Indeed, it has been reported that *Helicobacter pylori*, although not an oral pathogen, has also been associated with higher levels of HbA1c, possibly through the regulation of two hormones, leptin and ghrelin (82). Salivary glucose levels have been shown to be higher in uncontrolled and controlled diabetics than the healthy controls (83, 84, 85), but whether that excess glucose available in saliva is preferentially utilized by periodontal pathogens to make dental plaque or biofilm remains to be understood. *Capnocytophaga*, which had a higher relative abundance in PD in our study, has been reported to grow luxuriously in the presence of glucose (78). Furthermore, *F. nucleatum* have been shown to be in relatively higher abundance in people with HbA1c ≥to 8% and with a PD ≥5 mm (80). This suggests that establishment of pathogenic bacteria at healthy sites correlated with systemic glucose load and might expedite progression toward PD.

Furthermore, by utilizing MS-based multi-omics technologies, we discovered host-associated disease signatures. Plaques from PD patients contained significantly more host-derived proteins, which were enriched in actin filament–related proteins and likely have a mechanistic link to microbial invasion (69, 70). With the goal to understand how microbiota contribute to disease, approaches such as metaproteomics and metatranscriptomics, which enable detection of host response, will be critical for understanding these pathogenic interactions. We argue that MS-based ‘omics technologies could further strengthen our understanding of the host–microbe interactions, not only in being able to monitor potential protein response to microbial invasion but also in monitoring lipid changes in these host–microbe environments. Lipids are important biomolecules related to defense and invasion as they reside at the interface between cells and serve as structural or signaling molecules (72). In support of this idea, we found that the same host-derived proteins associated with PD were also strongly correlated with lipids classified as PCs or plasmenyl PCs. Further mechanistic studies will hopefully reveal how these molecules change during invasion and provide candidates for therapeutic intervention.

Beyond host–microbe interactions, lipidomics offers other novel insights about the oral microenvironment. A few recent studies have investigated saliva lipid profiles in chronic periodontitis (24, 86), however, quantified only a small number of lipids. The present study offers a far more comprehensive profiling of the oral biofilm lipidome and provides an important first step in linking these lipids to taxonomic branches, diet, and oral health. The fact that this multi-omics approach revealed a true, and yet described, lipid pathway in *Lautropia*, as well as a diet-related cluster suggestive of dairy consumption, showcases the discovery potential of this methodology. With the goal to better understand microbial microenvironments, a proteomic–lipidomic approach, like the one presented here, would offer significant biological insight, and we expect that future improvements to the methodology (*i.e.*, improved time or space resolution) could be hugely beneficial to our understanding of this complex system.

## Data Availability

Raw data, processed files, and code for analysis are available in the MassIVE repository MSV000084126 (ftp://massive.ucsd.edu/MSV000084126/).

## Supplemental data

This article contains supplemental data.

## Conflict of interest

J. J. C. is a consultant for Thermo Fisher Scientific. The remaining authors declare no conflicts of interest related to this work.
