# Supplementary material for: Proteomics, Lipidomics, Metabolomics, and 16S DNA Sequencing of Dental Plaque From Patients With Diabetes and Periodontal Disease
Source: Mol Cell Proteomics. 2021 Jul 29;20:100126. doi: 10.1016/j.mcpro.2021.100126 (PMC8426274; doi:10.1016/j.mcpro.2021.100126)
Supplement: Supplemental data [file mmc2.pdf]

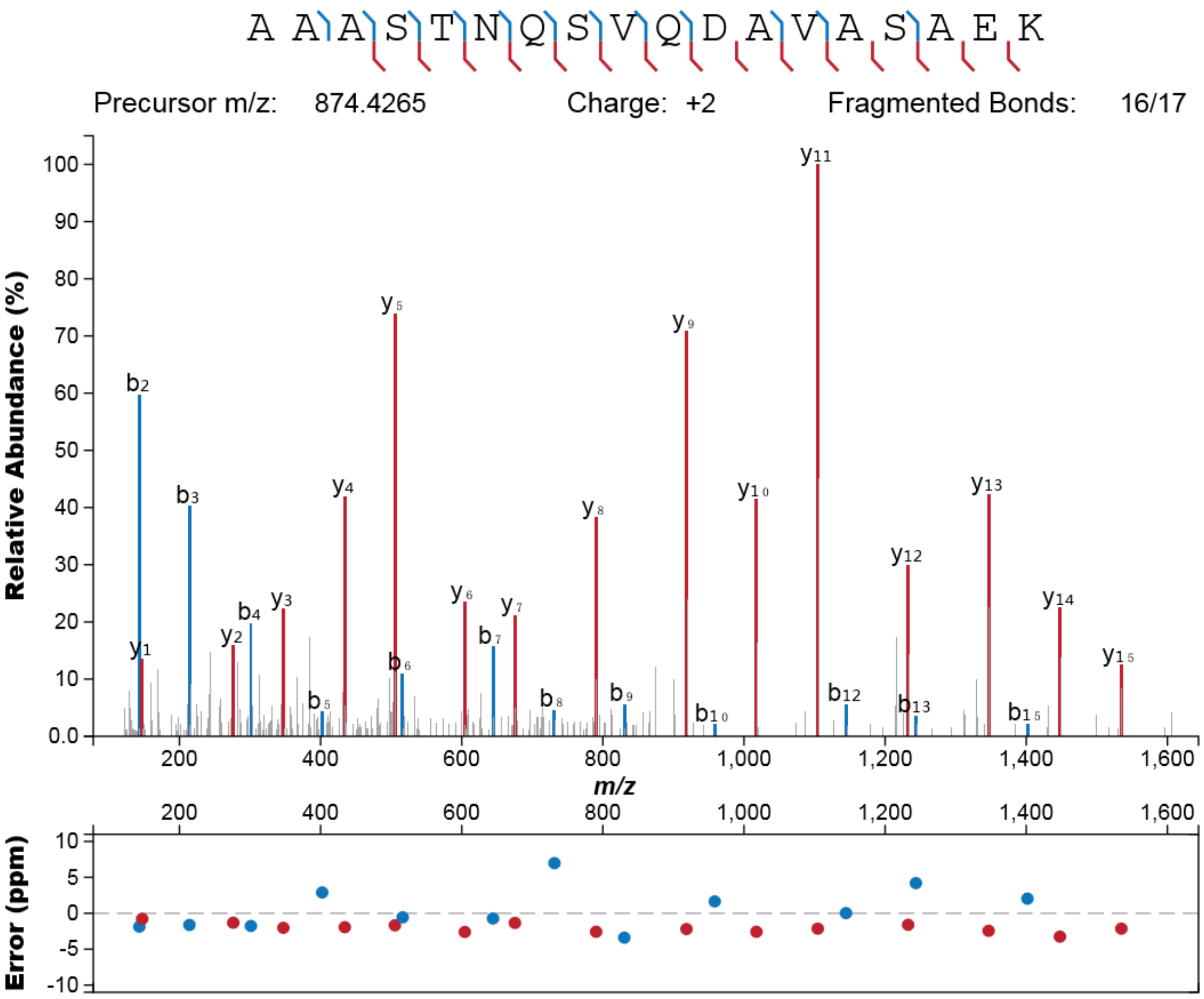
A A A S T N Q S V Q D A V A S A E K
Precursor m/z: 874.4265
Charge: +2
Fragmented Bonds: 16/17
Relative Abundance (%)
m/z
Error (ppm)
y11
y5
y9
b2
b3
y4
y10
y13
y8
y12
y14
y6
y7
y3
b4
y2
b7
y1
y15
b6
b12
b9
b5
b8
b13
b10
b15

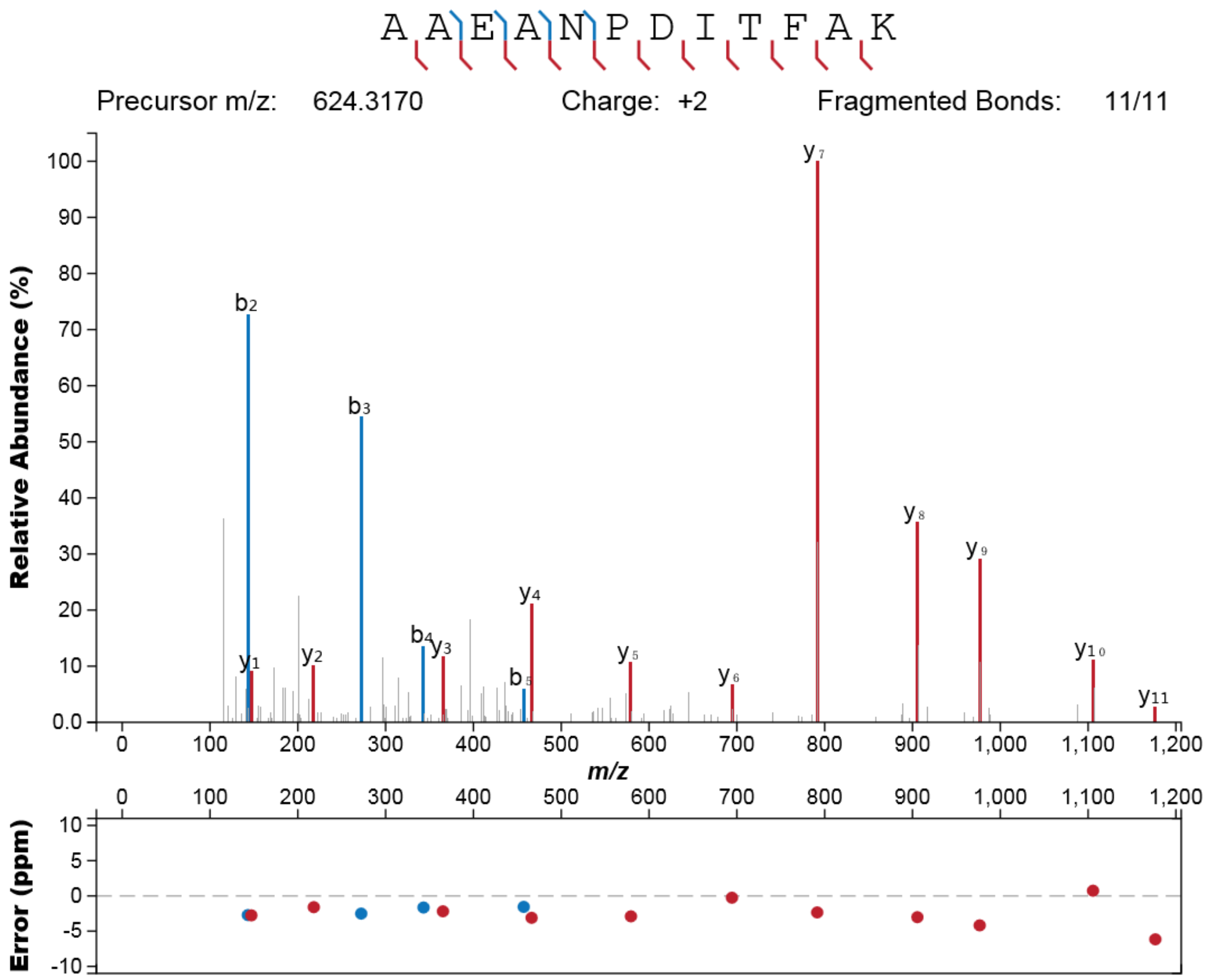

A A E A N P D I T F A K
Precursor m/z: 624.3170
Charge: +2
Fragmented Bonds: 11/11
Relative Abundance (%)
m/z
Error (ppm)
b2
b3
b4
b5
y1
y2
y3
y4
y5
y6
y7
y8
y9
y10
y11

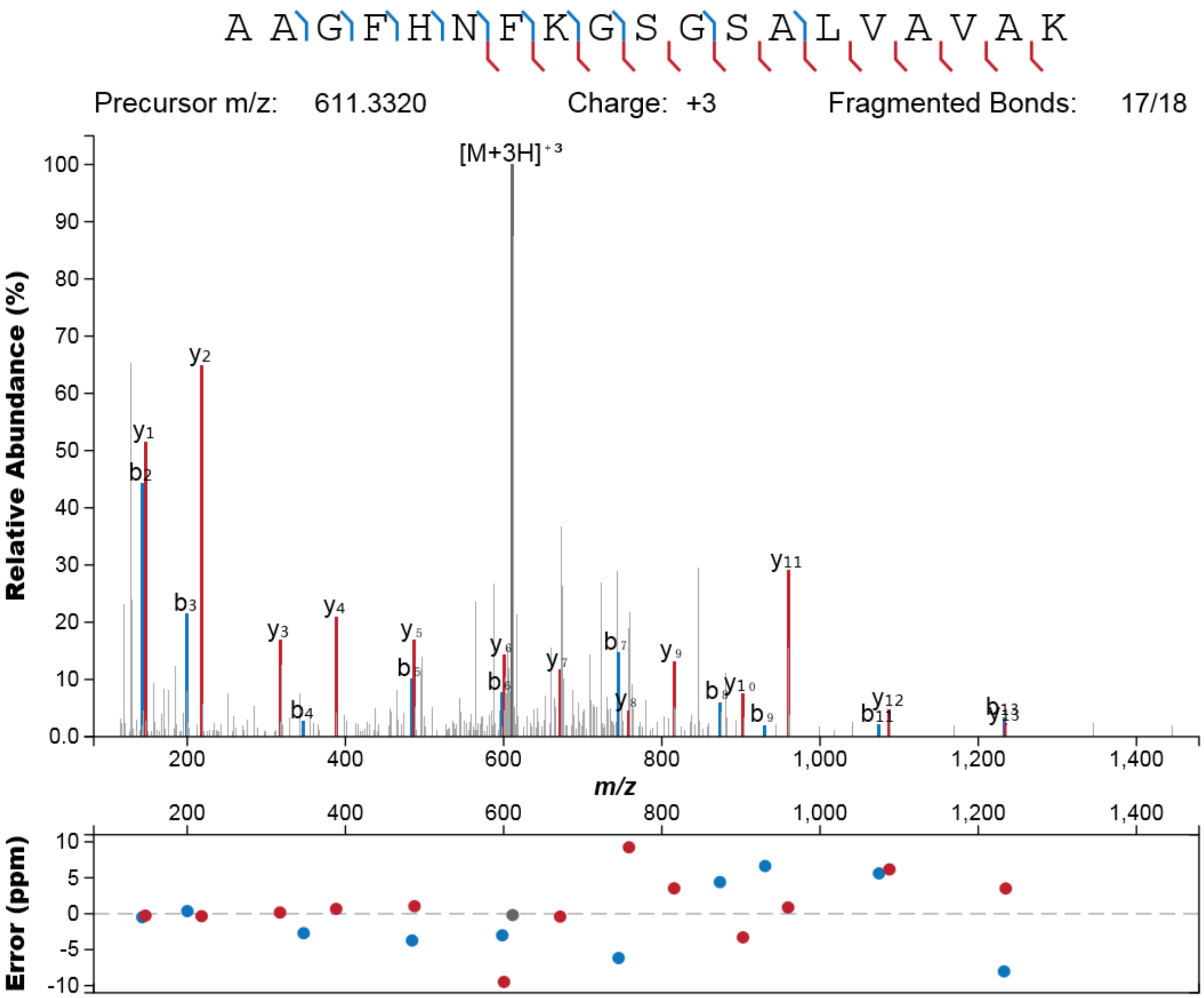
A A G F H N F K G S G S A L V A V A K
Precursor m/z: 611.3320
Charge: +3
Fragmented Bonds: 17/18
[M+3H]+3
Relative Abundance (%)
m/z
Error (ppm)
y1 y2 y3 y4 y5 y6 y7 y8 y9 y10 y11 y12 y13
b2 b3 b4 b5 b6 b7 b8 b9 b11 b13

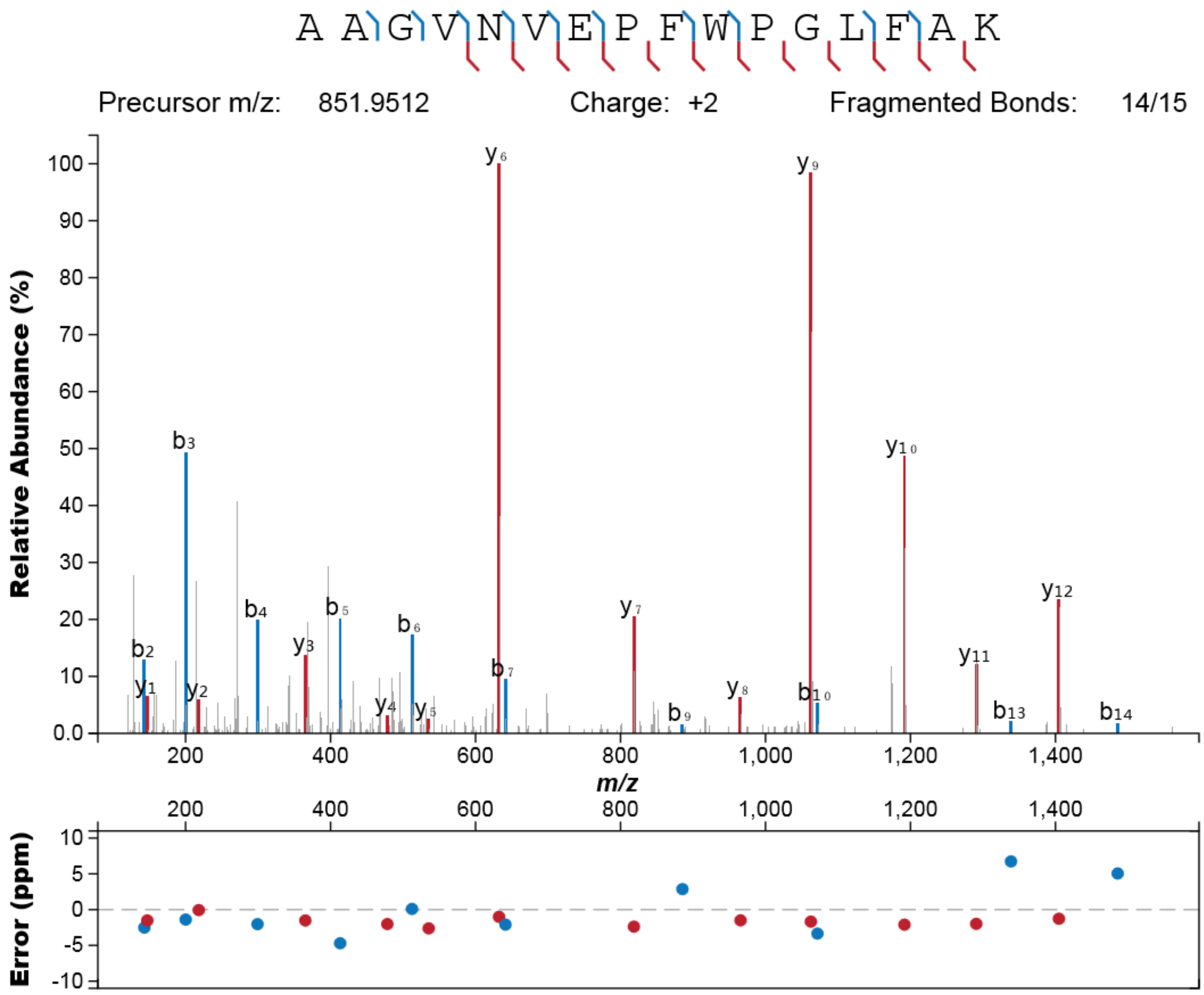

A A G V N V E P F W P G L F A K
Precursor m/z: 851.9512
Charge: +2
Fragmented Bonds: 14/15
Relative Abundance (%)
m/z
Error (ppm)
y6
y9
b3
y10
b4
b5
b6
y7
y12
y3
b2
y11
b7
y1
y2
y4
y5
b9
y8
b10
b13
b14

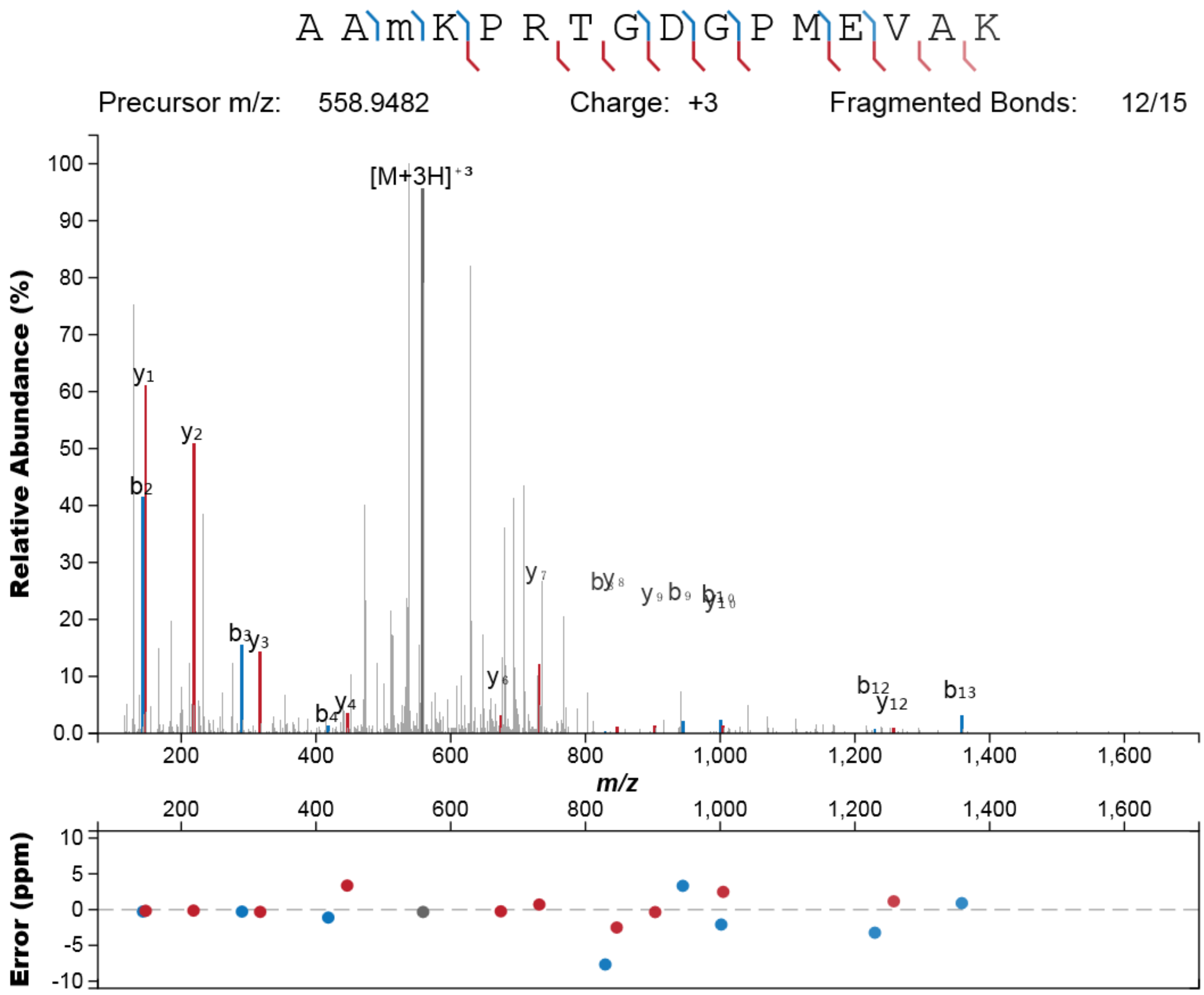

A A m K P R T G D G P M E V A K
Precursor m/z: 558.9482
Charge: +3
Fragmented Bonds: 12/15
Relative Abundance (%)
[M+3H]+3
y1
y2
b2
b3
y3
b4
y4
y6
y7
by8
y9
b9
b10
y10
b12
y12
b13
m/z
Error (ppm)

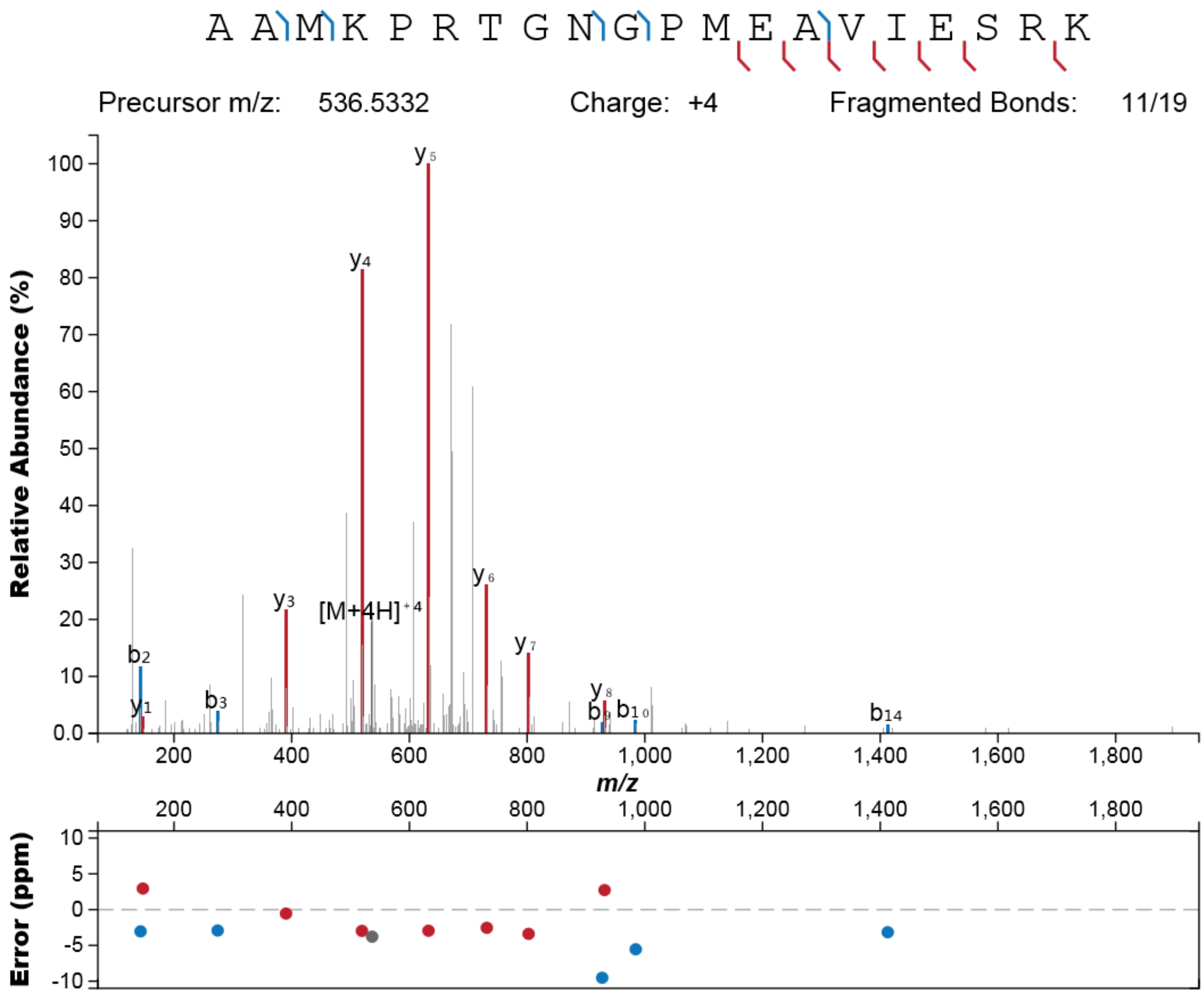

A A M K P R T G N G P M E A V I E S R K
Precursor m/z: 536.5332
Charge: +4
Fragmented Bonds: 11/19
Relative Abundance (%)
m/z
Error (ppm)
y5
y4
y6
y3
[M+4H]+4
y7
b2
y1
b3
y8
b9
b10
b14

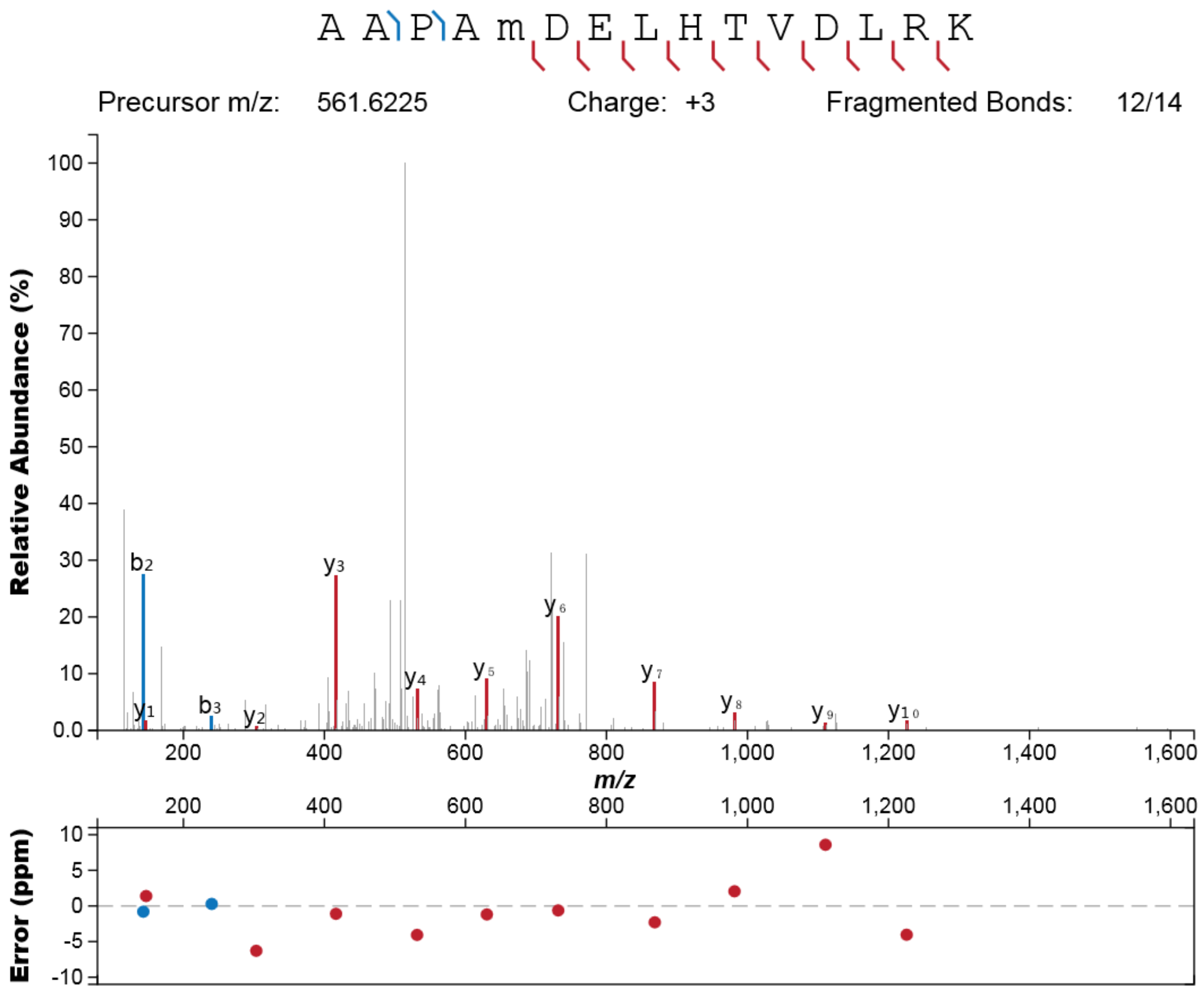
A A P A m D E L H T V D L R K
Precursor m/z: 561.6225
Charge: +3
Fragmented Bonds: 12/14
Relative Abundance (%)
m/z
Error (ppm)
b2
b3
y1
y2
y3
y4
y5
y6
y7
y8
y9
y10

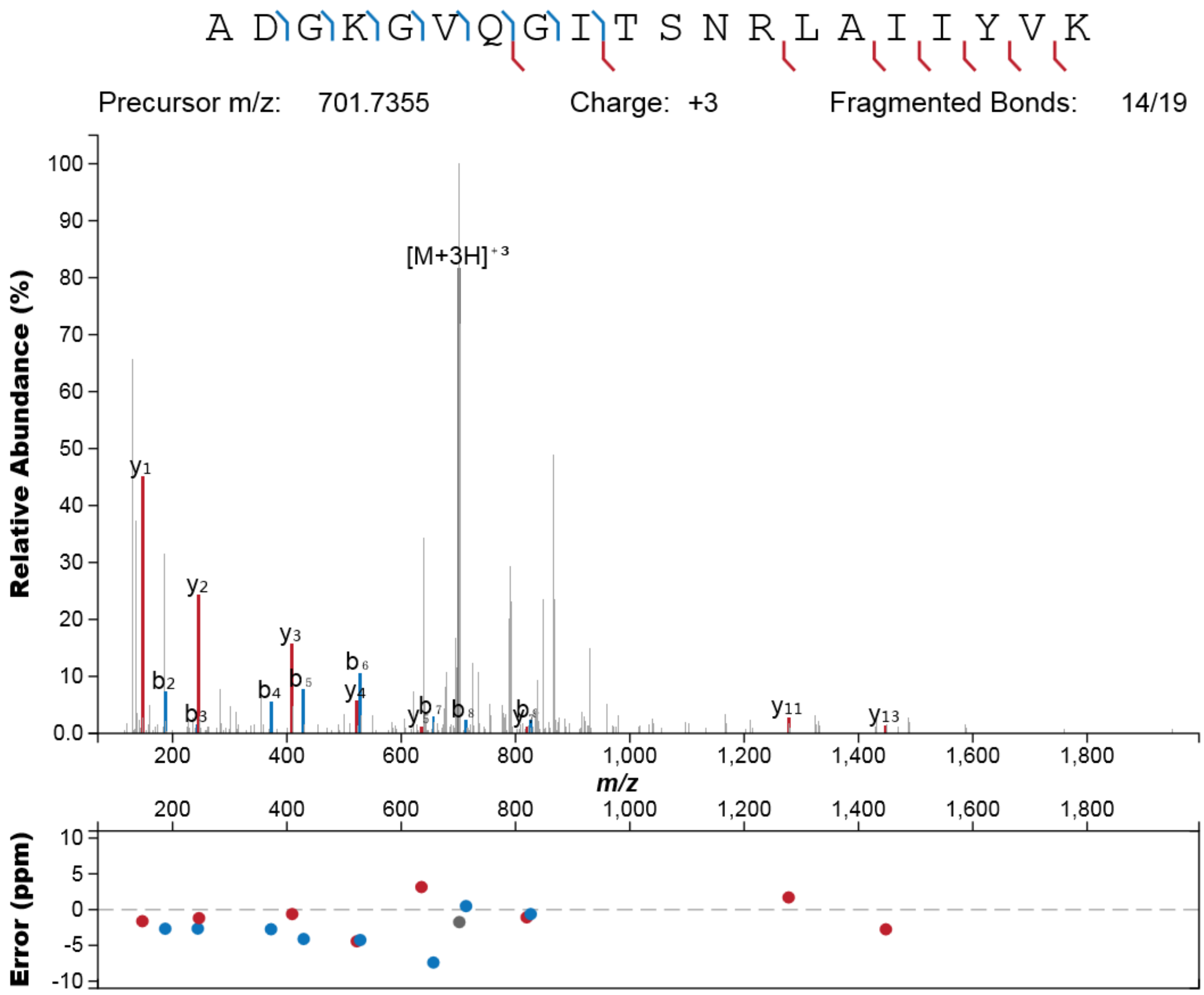
A D G K G V Q G I T S N R L A I I Y V K
Precursor m/z: 701.7355
Charge: +3
Fragmented Bonds: 14/19
Relative Abundance (%)
[M+3H]+3
y1
y2
y3
y4
y11
y13
b2
b3
b4
b5
b6
b7
b8
m/z
Error (ppm)

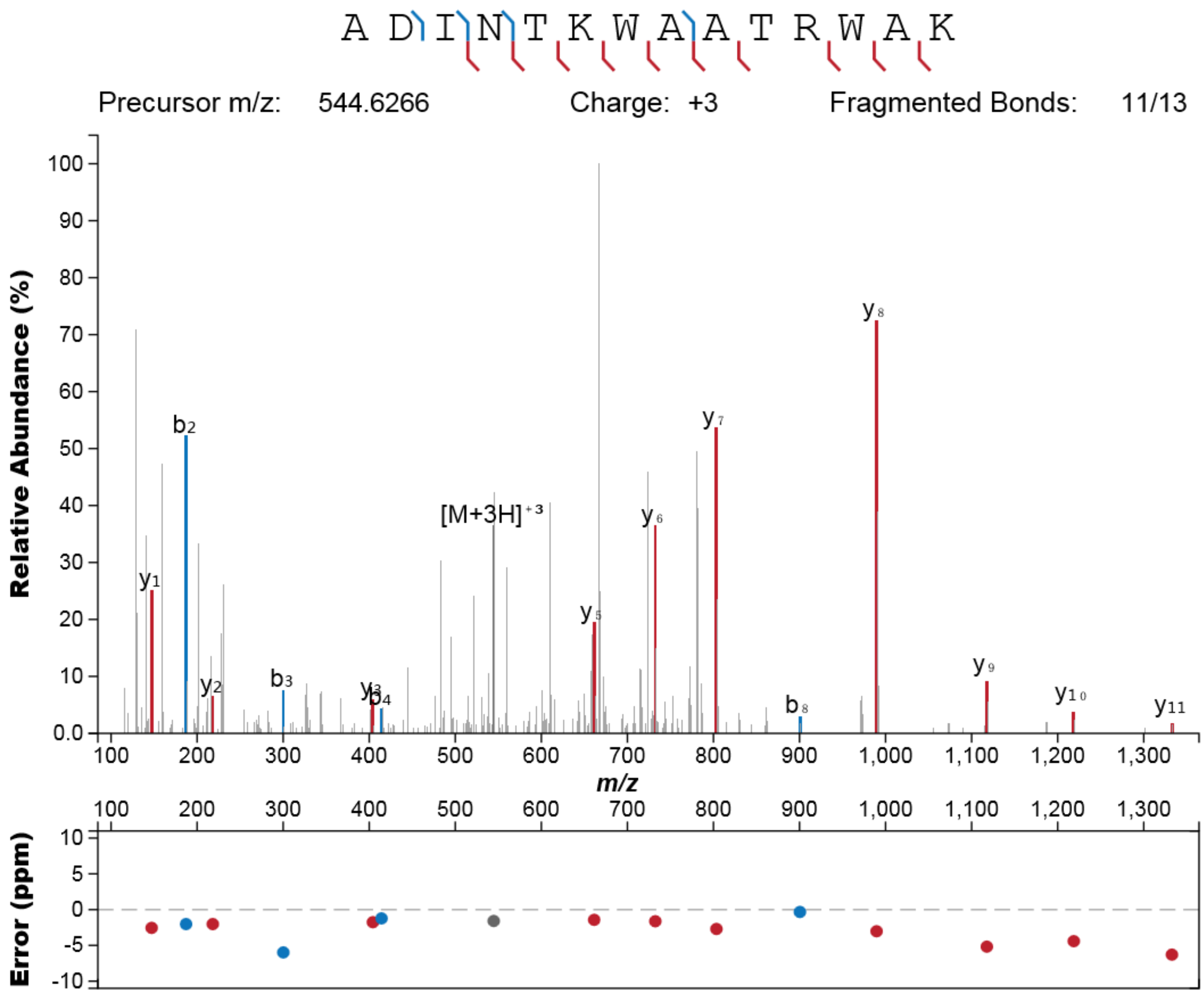

A D I N T K W A A T R W A K
Precursor m/z: 544.6266
Charge: +3
Fragmented Bonds: 11/13
Relative Abundance (%)
m/z
Error (ppm)
b2
b3
b4
b8
y1
y2
y3
y5
y6
y7
y8
y9
y10
y11
[M+3H]+3

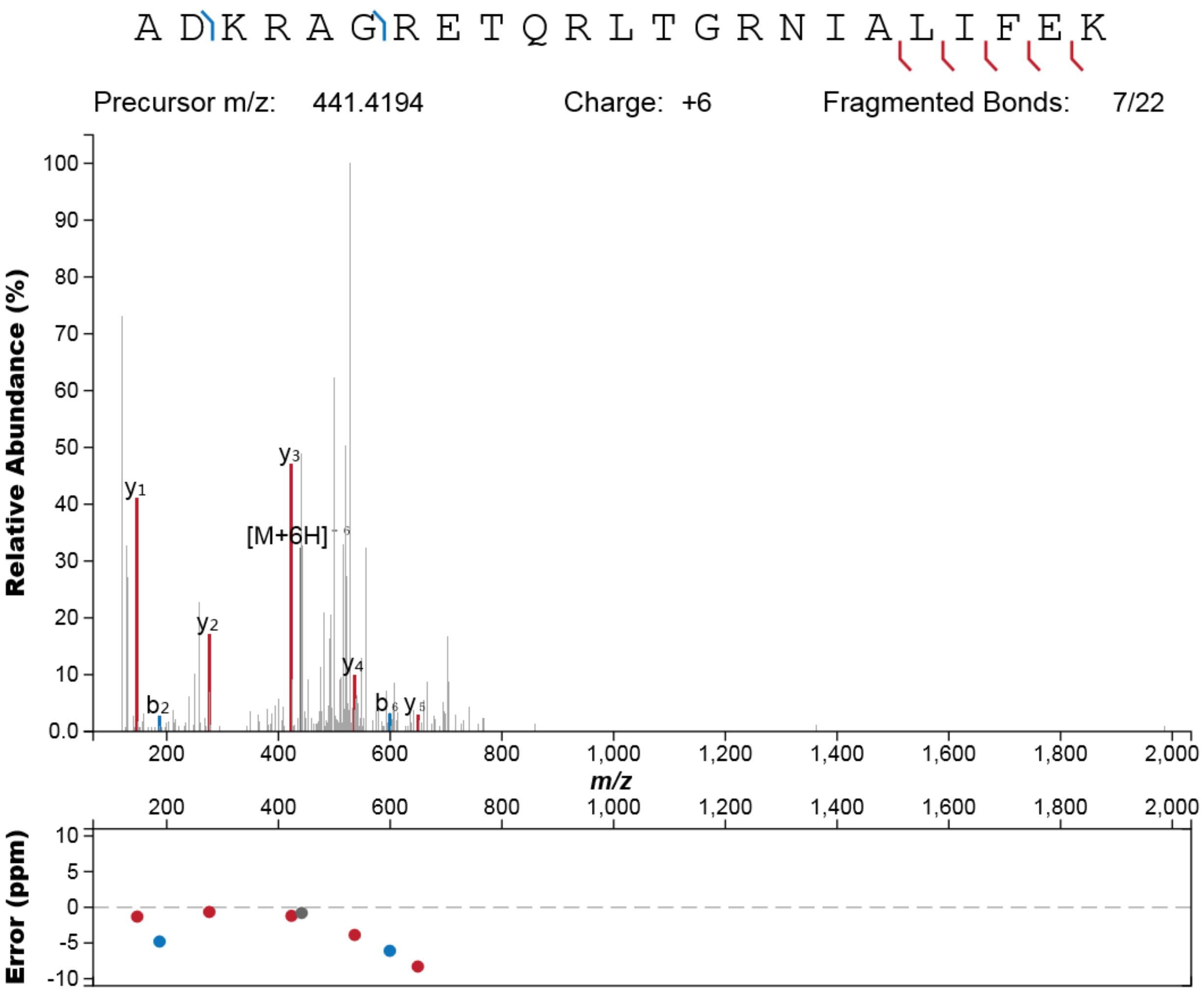
A D K R A G R E T Q R L T G R N I A L I F E K
Precursor m/z: 441.4194
Charge: +6
Fragmented Bonds: 7/22
Relative Abundance (%)
y1
b2
y2
y3
[M+6H]
y4
b6
y5
m/z
Error (ppm)

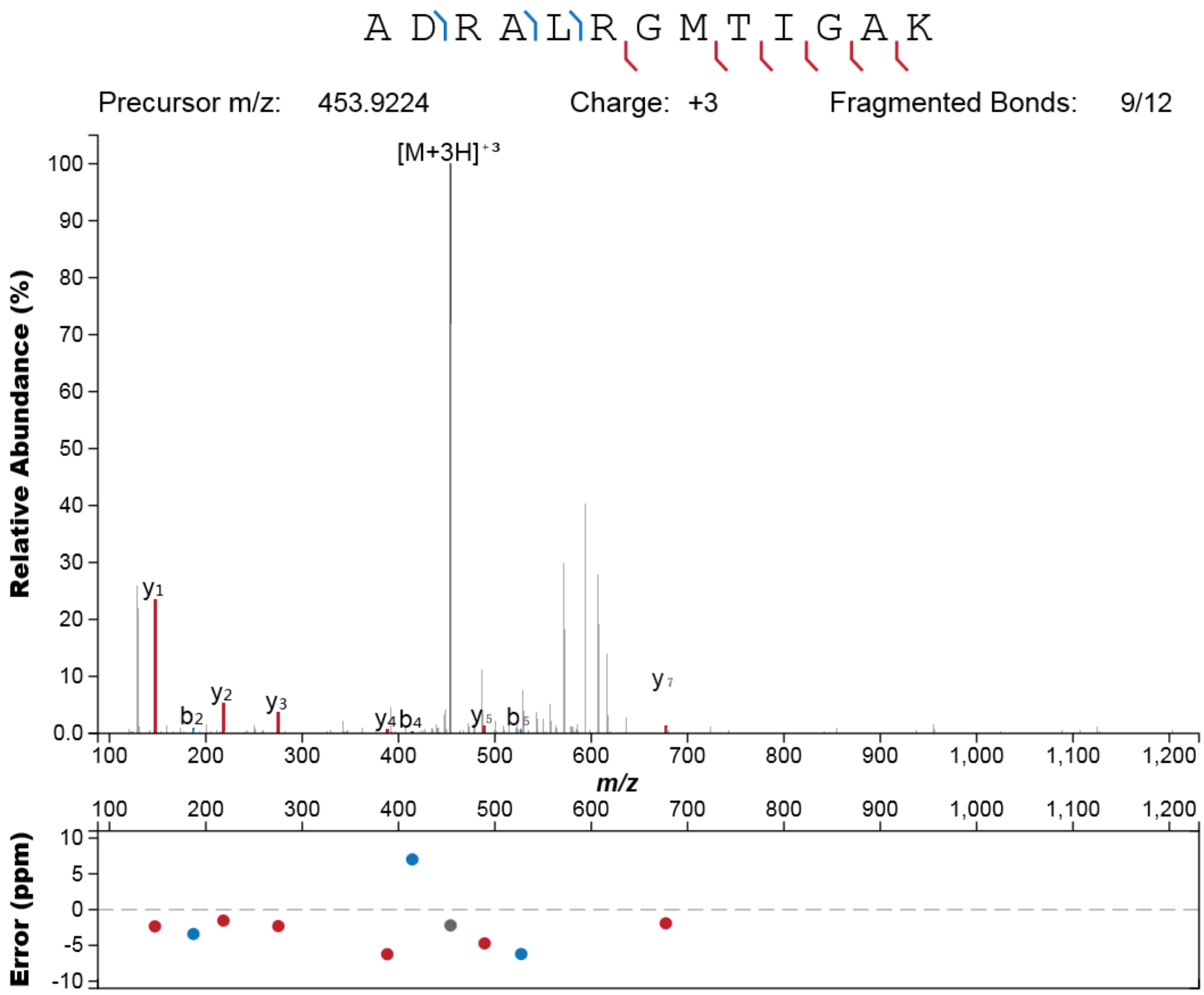
A D R A L R G M T I G A K
Precursor m/z: 453.9224
Charge: +3
Fragmented Bonds: 9/12
[M+3H]+3
Relative Abundance (%)
m/z
Error (ppm)
y1
b2
y2
y3
y4
b4
y5
b5
y7

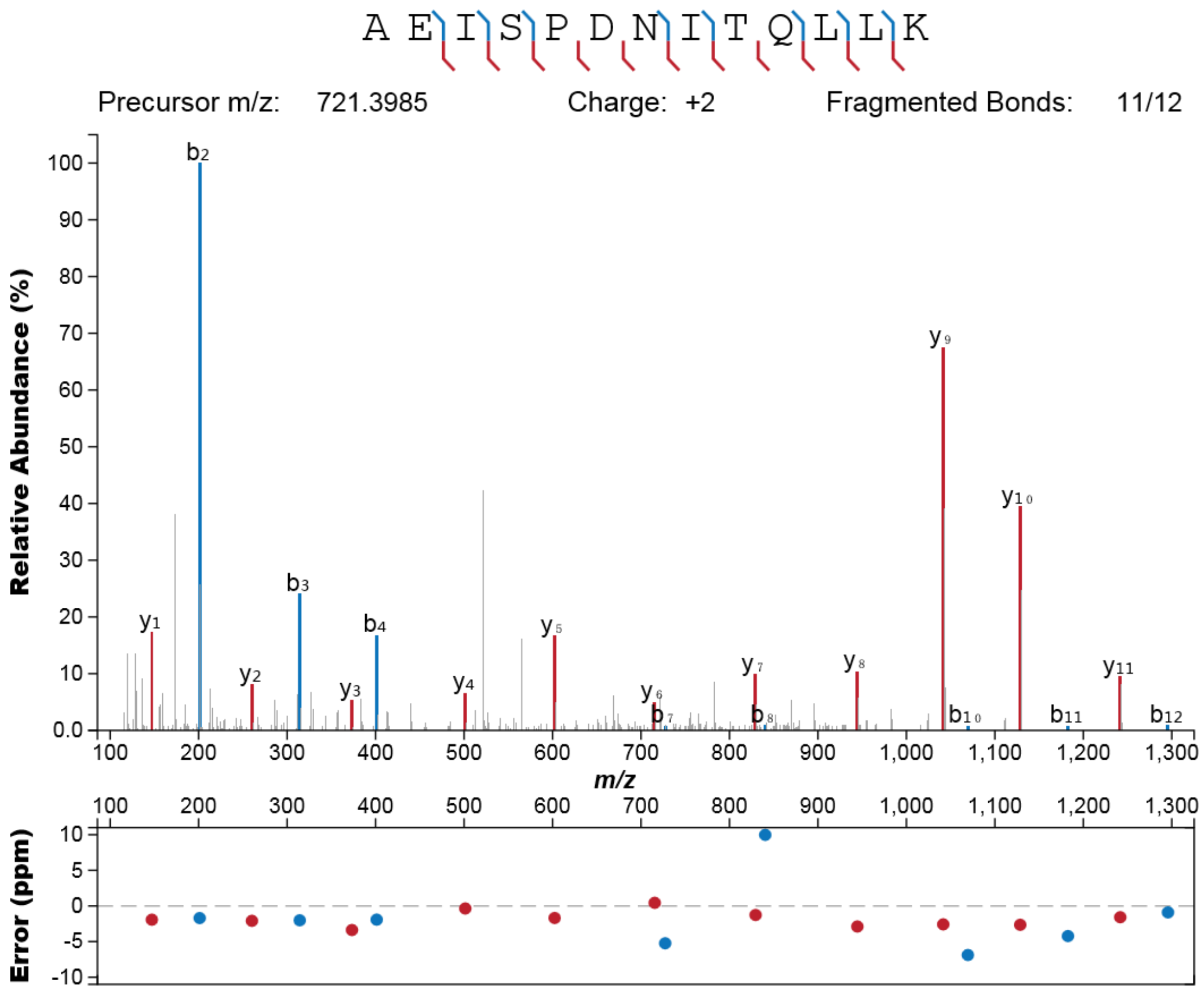
A E I S P D N I T Q L L K
Precursor m/z: 721.3985
Charge: +2
Fragmented Bonds: 11/12
Relative Abundance (%)
m/z
Error (ppm)
b2
b3
b4
b7
b8
b10
b11
b12
y1
y2
y3
y4
y5
y6
y7
y8
y9
y10
y11

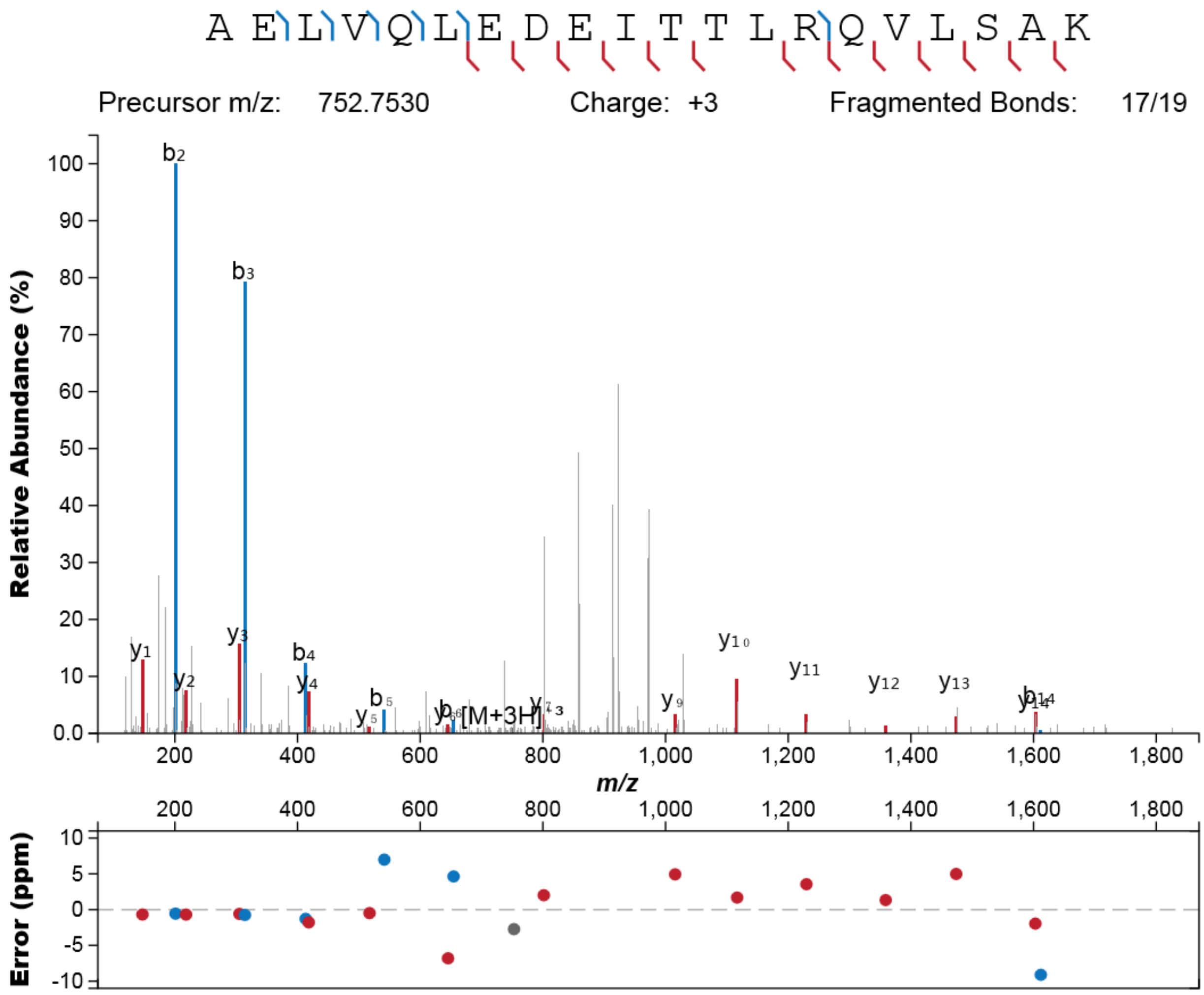
A E L V Q L E D E I T T L R Q V L S A K
Precursor m/z: 752.7530
Charge: +3
Fragmented Bonds: 17/19
Relative Abundance (%)
m/z
Error (ppm)
b2
b3
b4
b5
b6
y1
y2
y3
y4
y5
y9
y10
y11
y12
y13
y14
b14
[M+3H]

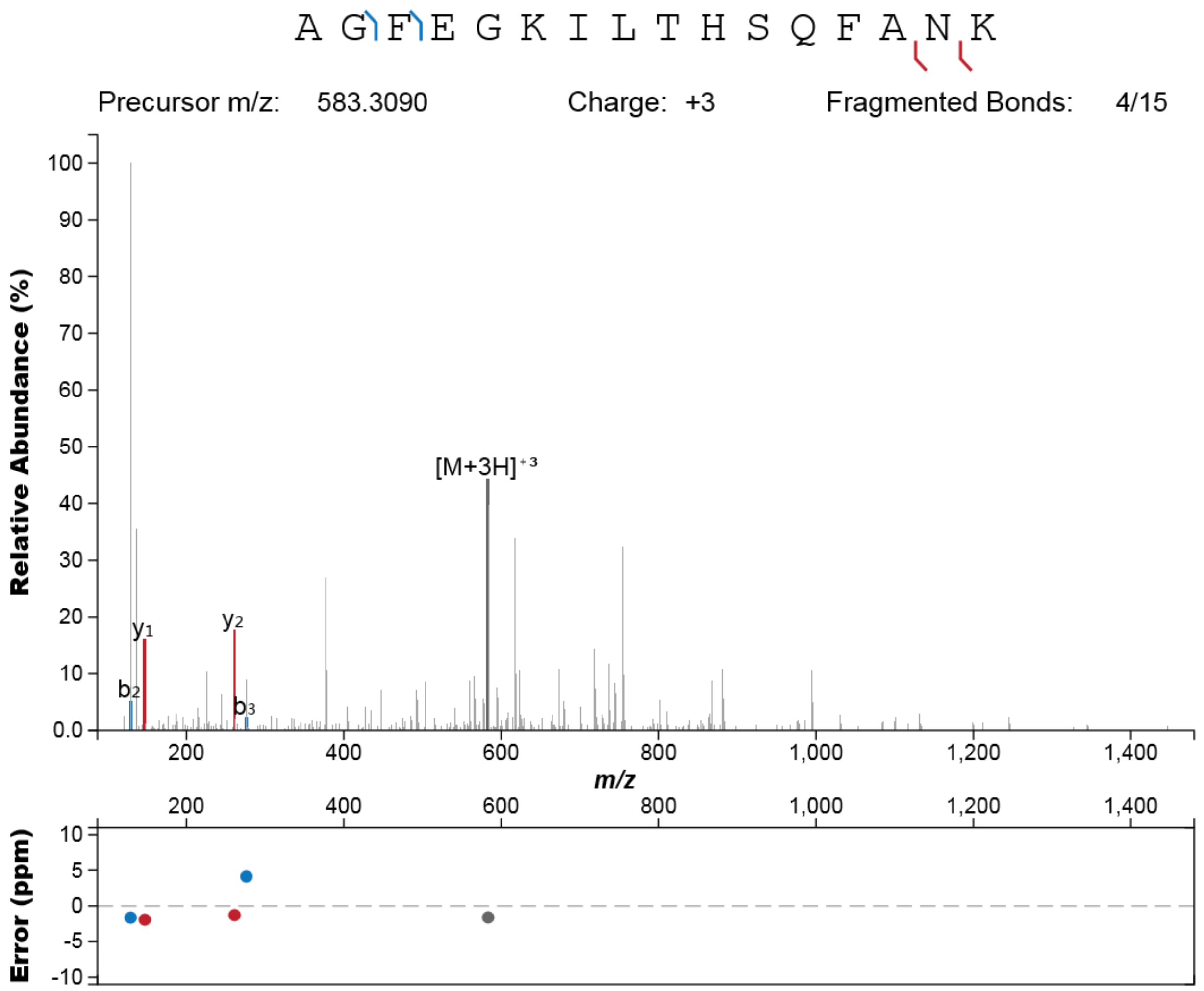
A G F E G K I L T H S Q F A N K
Precursor m/z: 583.3090
Charge: +3
Fragmented Bonds: 4/15
Relative Abundance (%)
[M+3H]+3
y1
y2
b2
b3
m/z
Error (ppm)

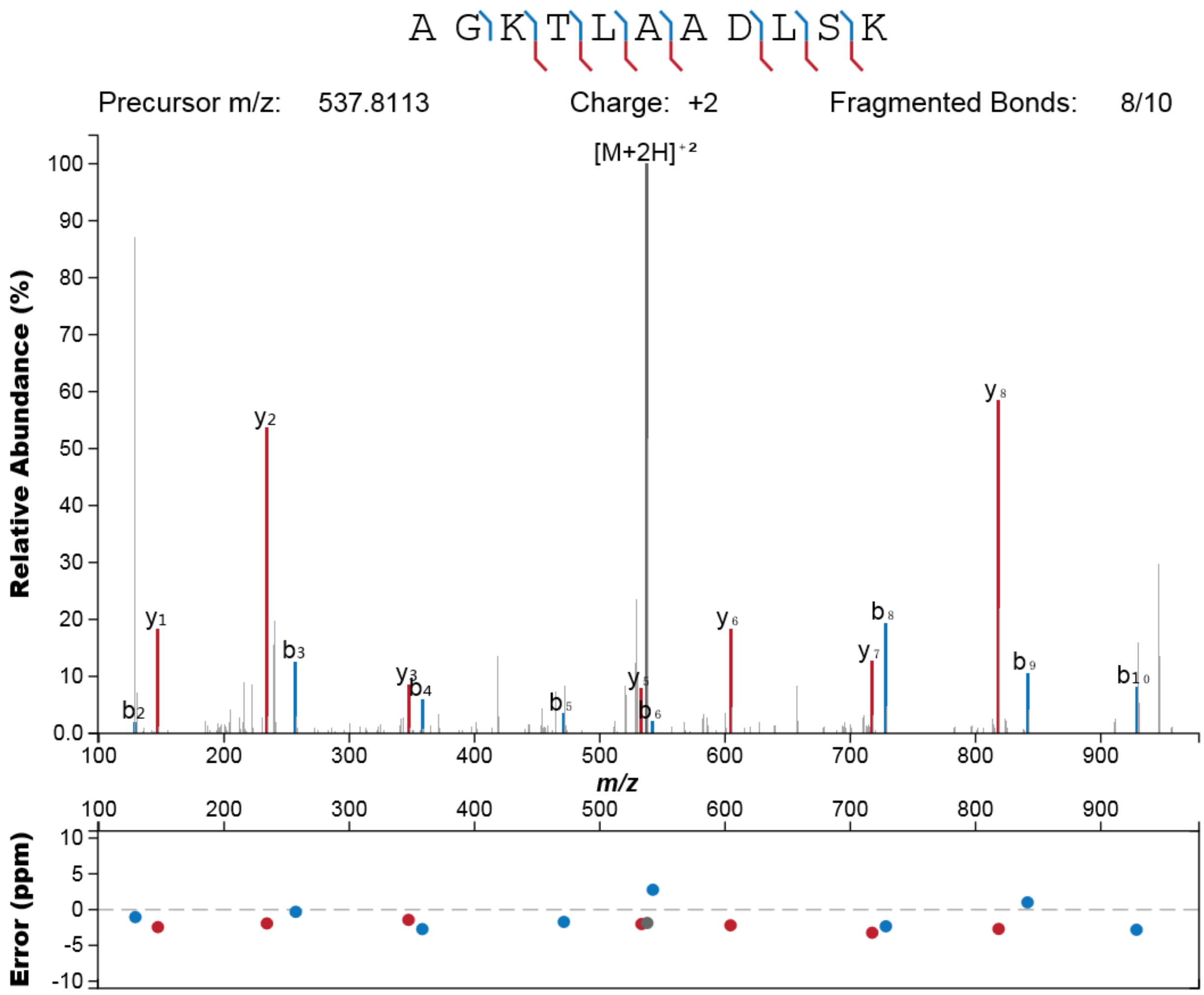

A G K T L A A D L S K
Precursor m/z: 537.8113
Charge: +2
Fragmented Bonds: 8/10
[M+2H]+2
Relative Abundance (%)
m/z
Error (ppm)
y1
y2
y3
y5
y6
y7
y8
b2
b3
b4
b5
b6
b8
b9
b10

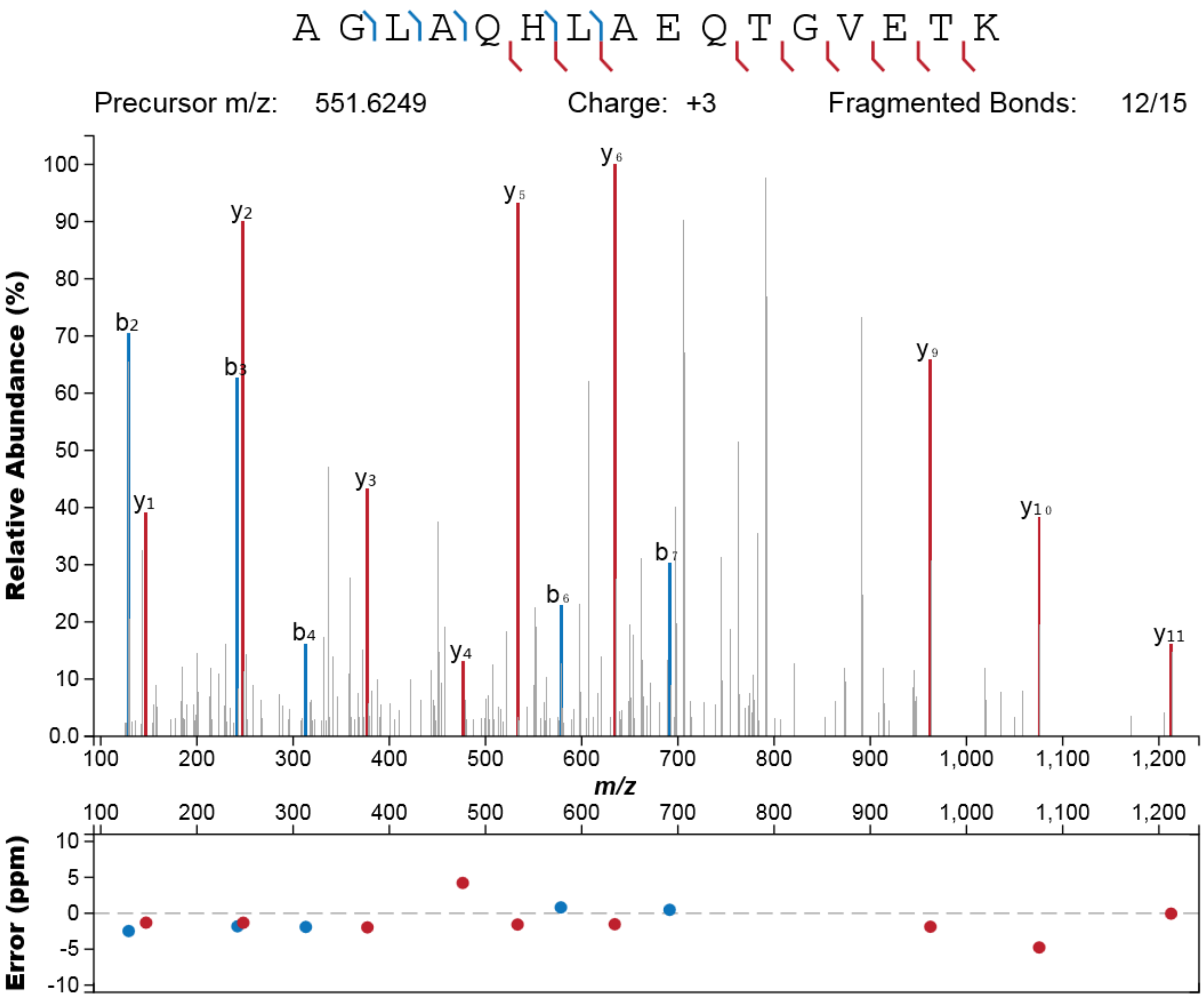
A G L A Q H L A E Q T G V E T K
Precursor m/z: 551.6249
Charge: +3
Fragmented Bonds: 12/15
Relative Abundance (%)
m/z
Error (ppm)
b2
b3
b4
b6
b7
y1
y2
y3
y4
y5
y6
y9
y10
y11

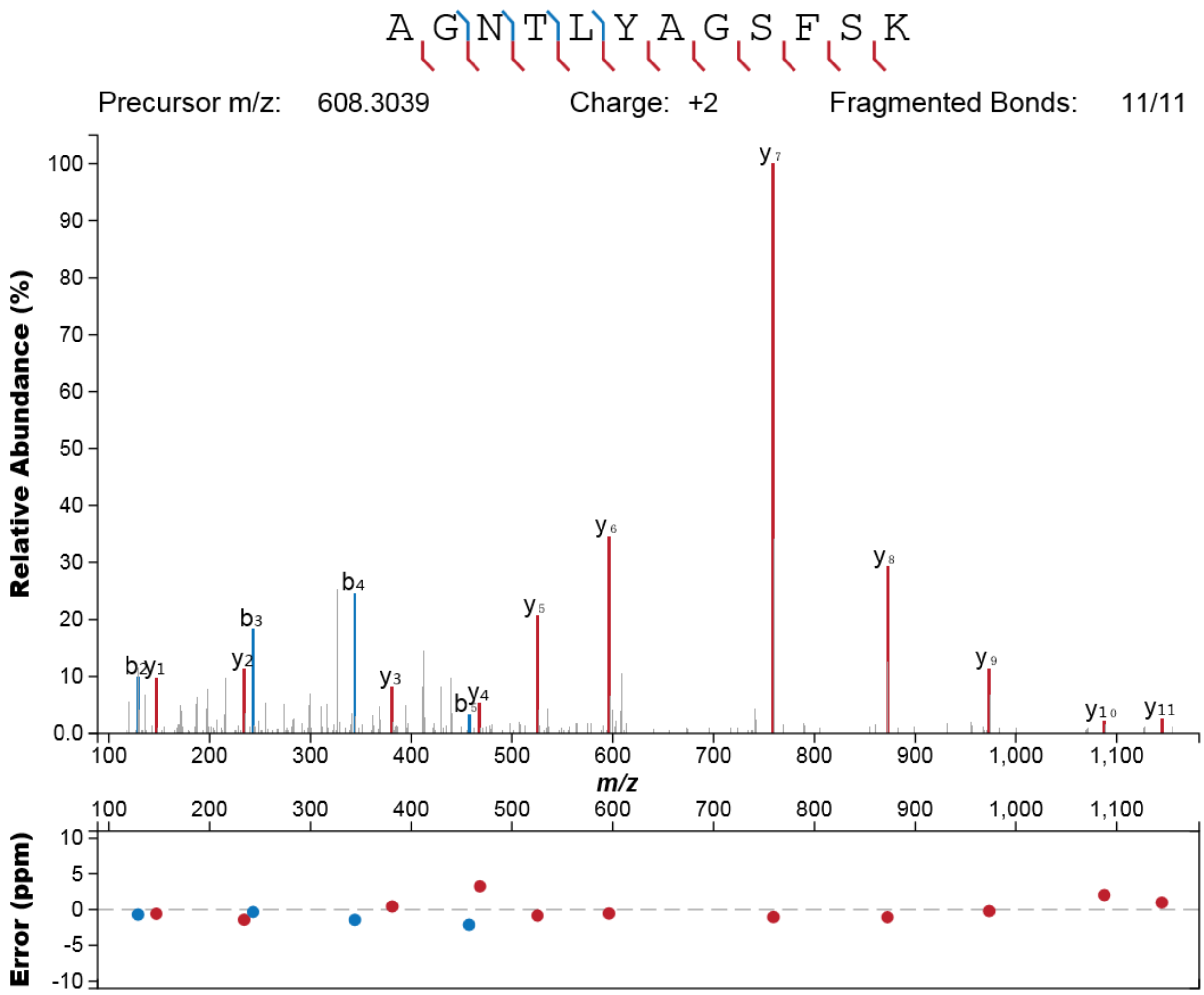
A G N T L Y A G S F S K
Precursor m/z: 608.3039
Charge: +2
Fragmented Bonds: 11/11
Relative Abundance (%)
m/z
Error (ppm)
b2
y1
y2
b3
b4
y3
b5
y4
y5
y6
y7
y8
y9
y10
y11

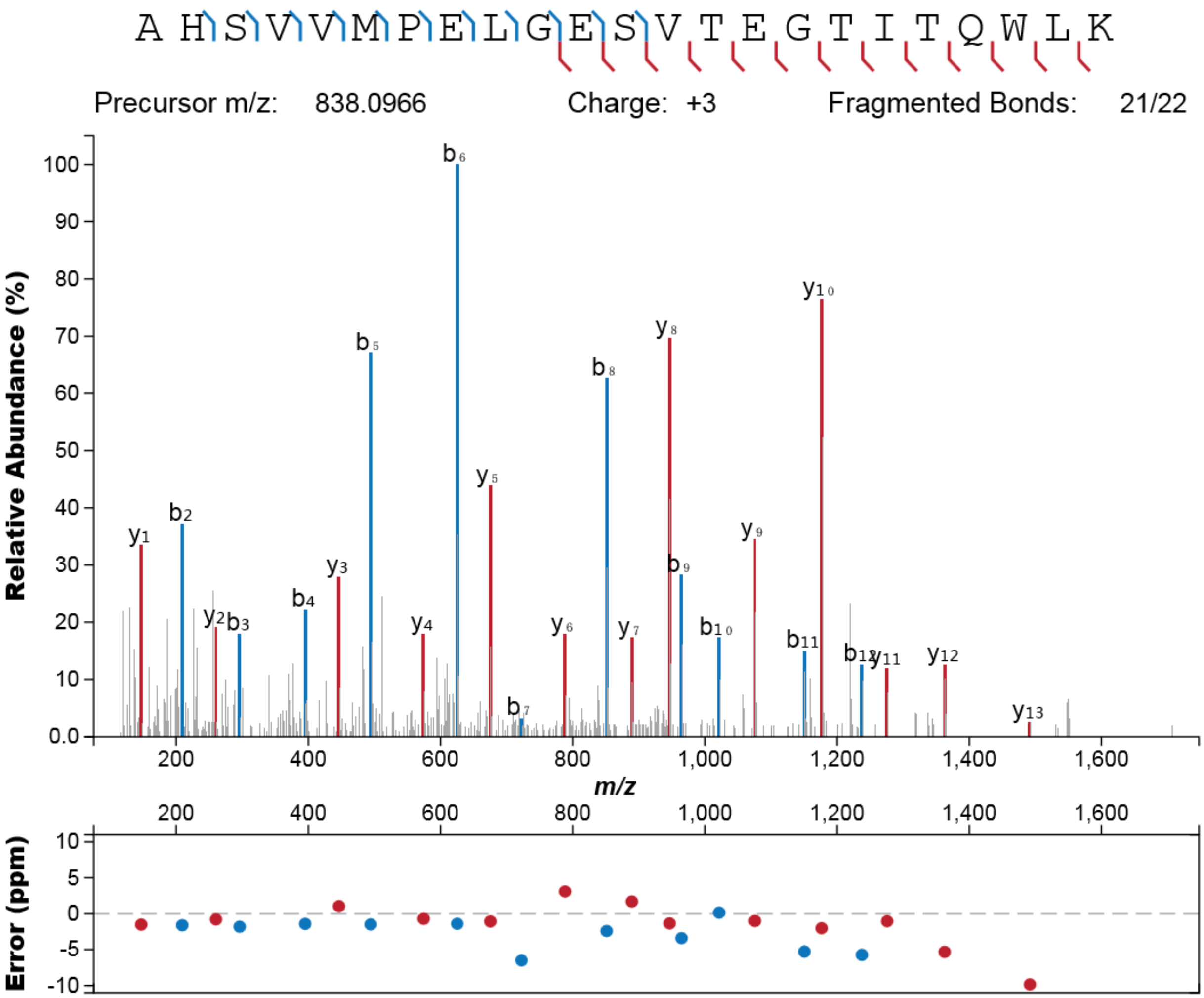

A H S V V M P E L G E S V T E G T I T Q W L K
Precursor m/z: 838.0966
Charge: +3
Fragmented Bonds: 21/22
Relative Abundance (%)
m/z
Error (ppm)

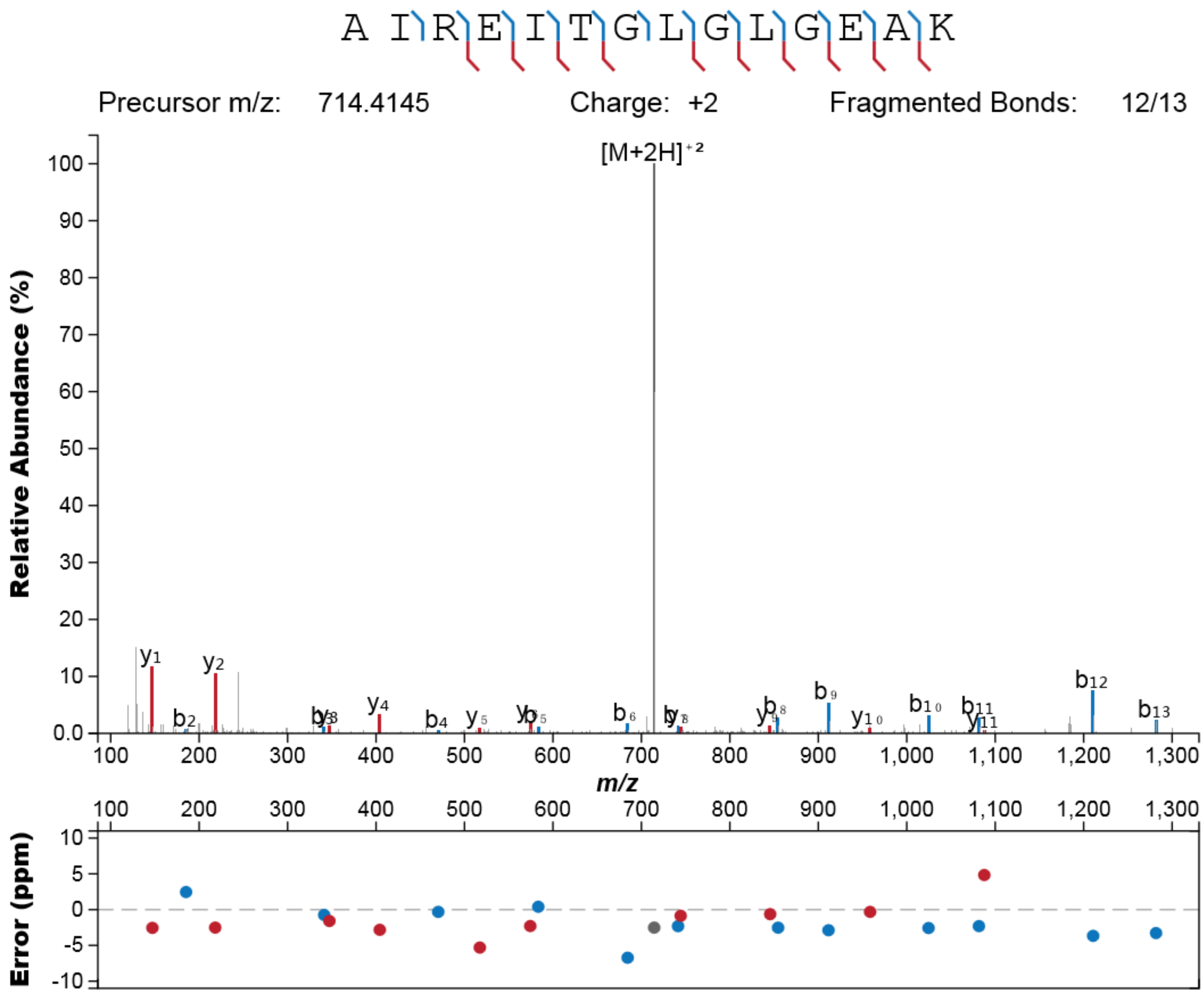
A I R E I T G L G L G E A K
Precursor m/z: 714.4145
Charge: +2
Fragmented Bonds: 12/13
[M+2H]+2
Relative Abundance (%)
m/z
Error (ppm)
y1
y2
b2
y3
b3
y4
b4
y5
y6
b5
b6
y7
b7
y8
y9
b8
b9
y10
b10
b11
y11
b12
b13

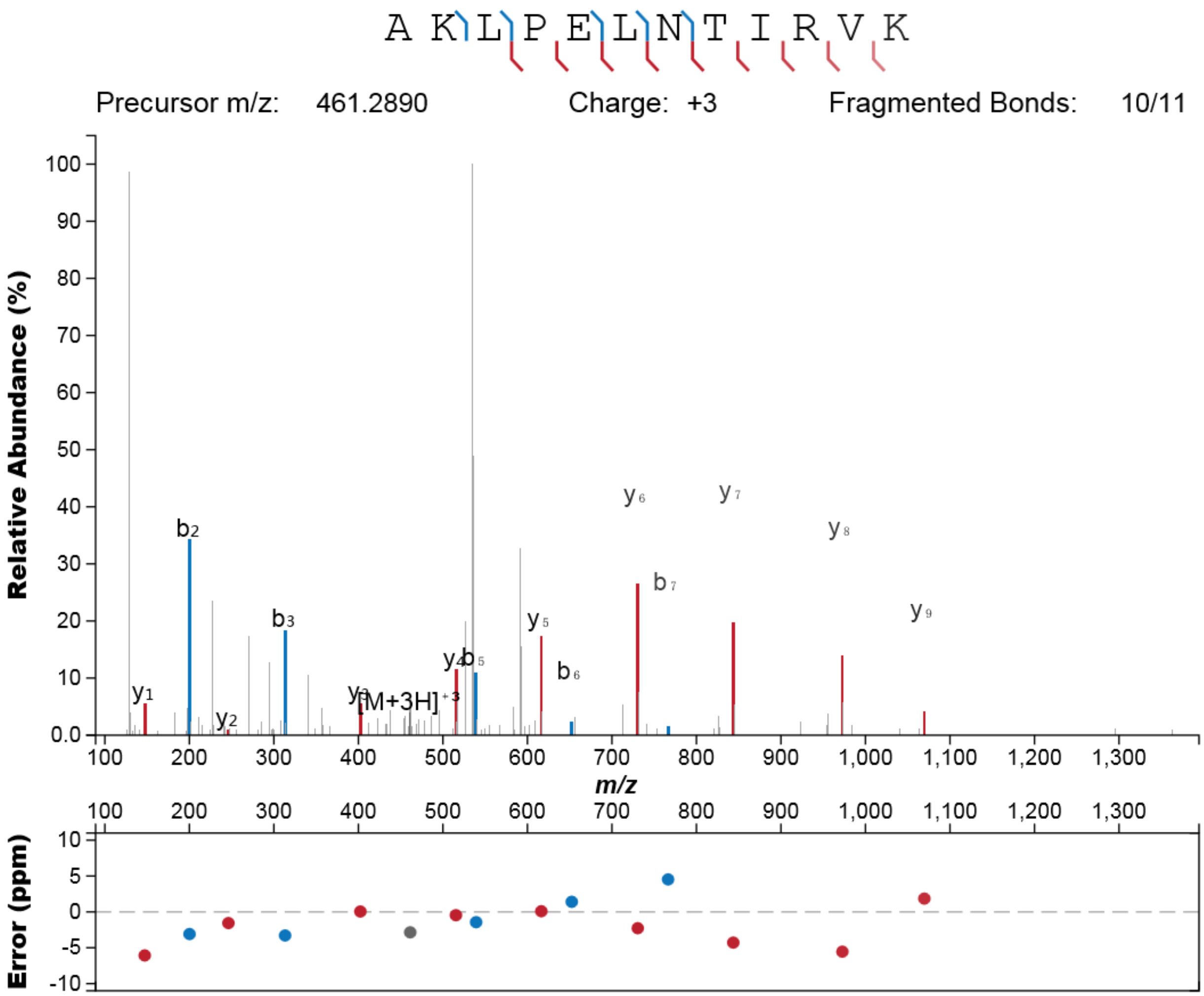
A K L P E L N T I R V K
Precursor m/z: 461.2890
Charge: +3
Fragmented Bonds: 10/11
Relative Abundance (%)
m/z
Error (ppm)
b2
b3
b5
b6
b7
y1
y2
y3
y4
y5
y6
y7
y8
y9
[M+3H]+3

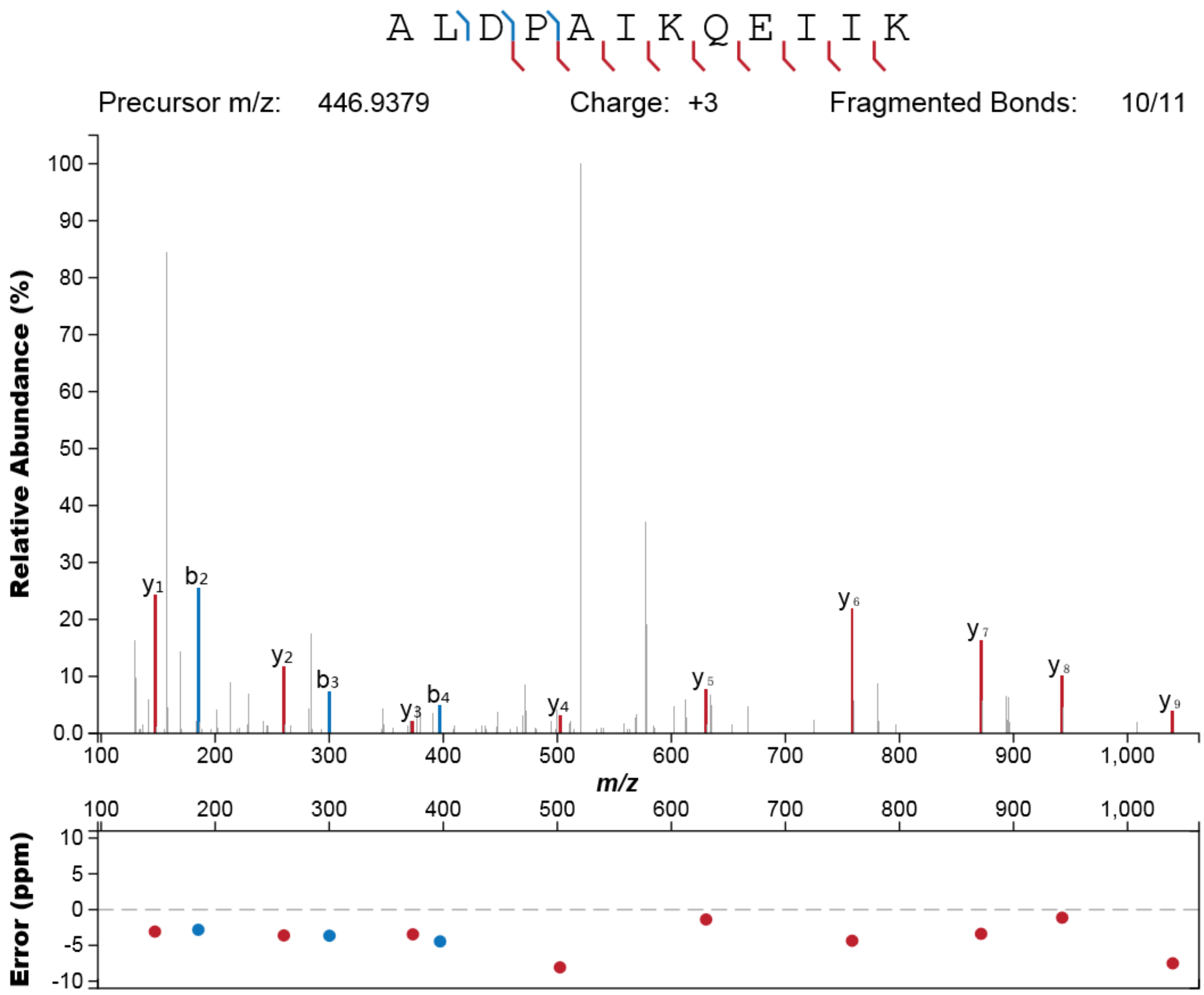

A L D P A I K Q E I I K
Precursor m/z: 446.9379
Charge: +3
Fragmented Bonds: 10/11
Relative Abundance (%)
m/z
Error (ppm)
y1
b2
y2
b3
y3
b4
y4
y5
y6
y7
y8
y9

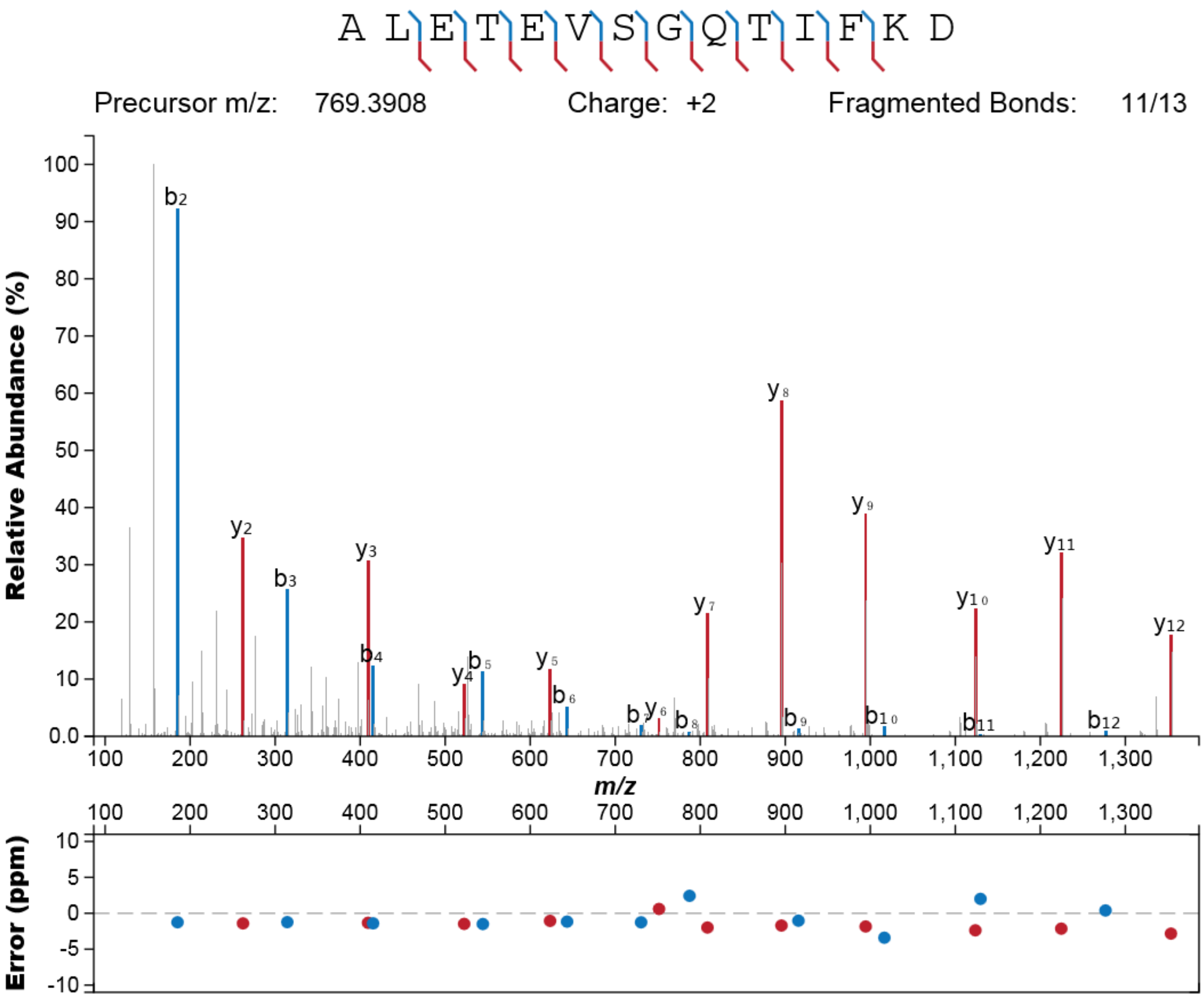
A L E T E V S G Q T I F K D
Precursor m/z: 769.3908
Charge: +2
Fragmented Bonds: 11/13
Relative Abundance (%)
m/z
Error (ppm)
b2
y2
b3
y3
b4
y4
b5
y5
b6
b7
y6
y7
b8
y8
b9
y9
b10
y10
b11
y11
b12
y12

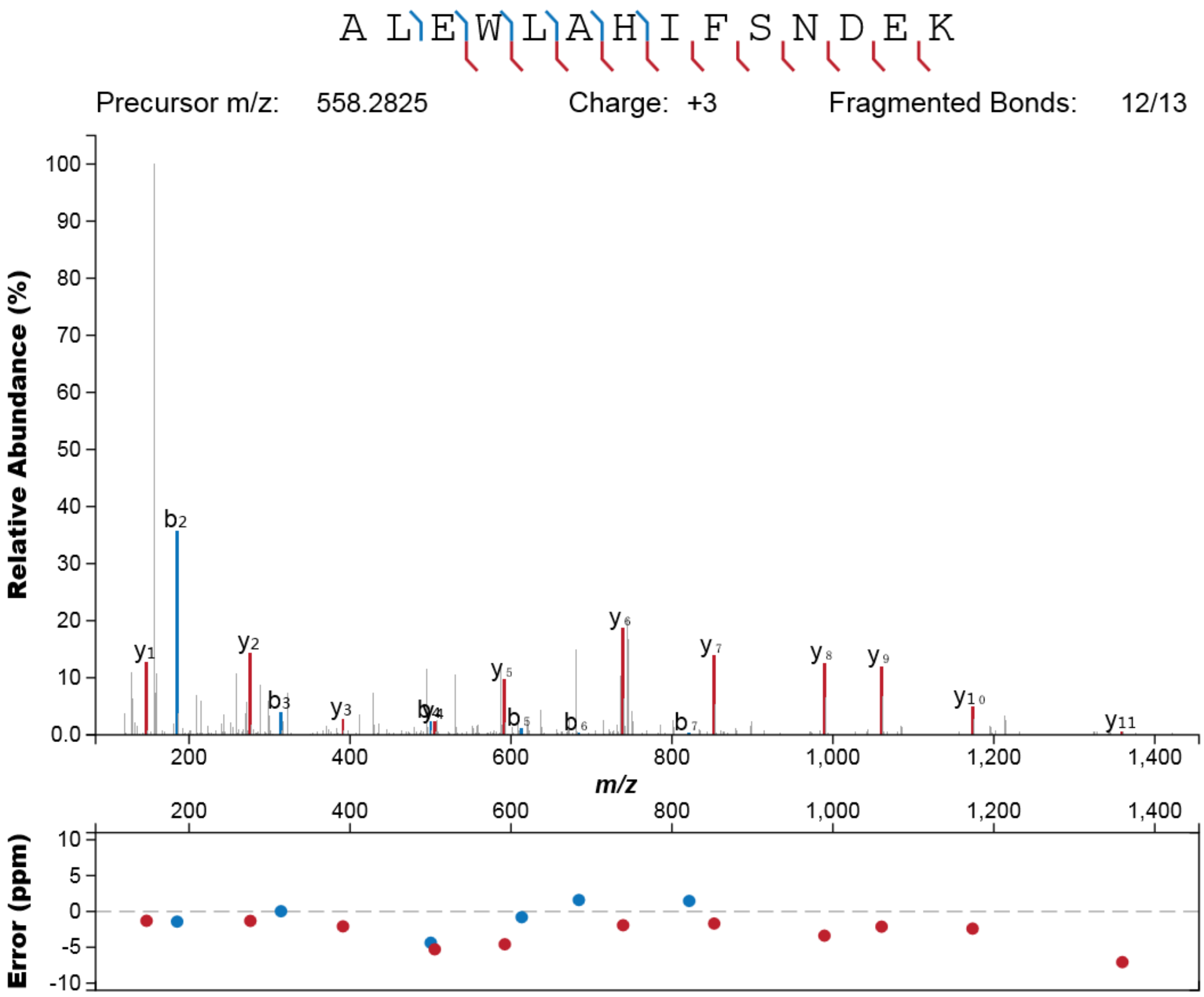
A L E W L A H I F S N D E K
Precursor m/z: 558.2825
Charge: +3
Fragmented Bonds: 12/13
Relative Abundance (%)
m/z
Error (ppm)

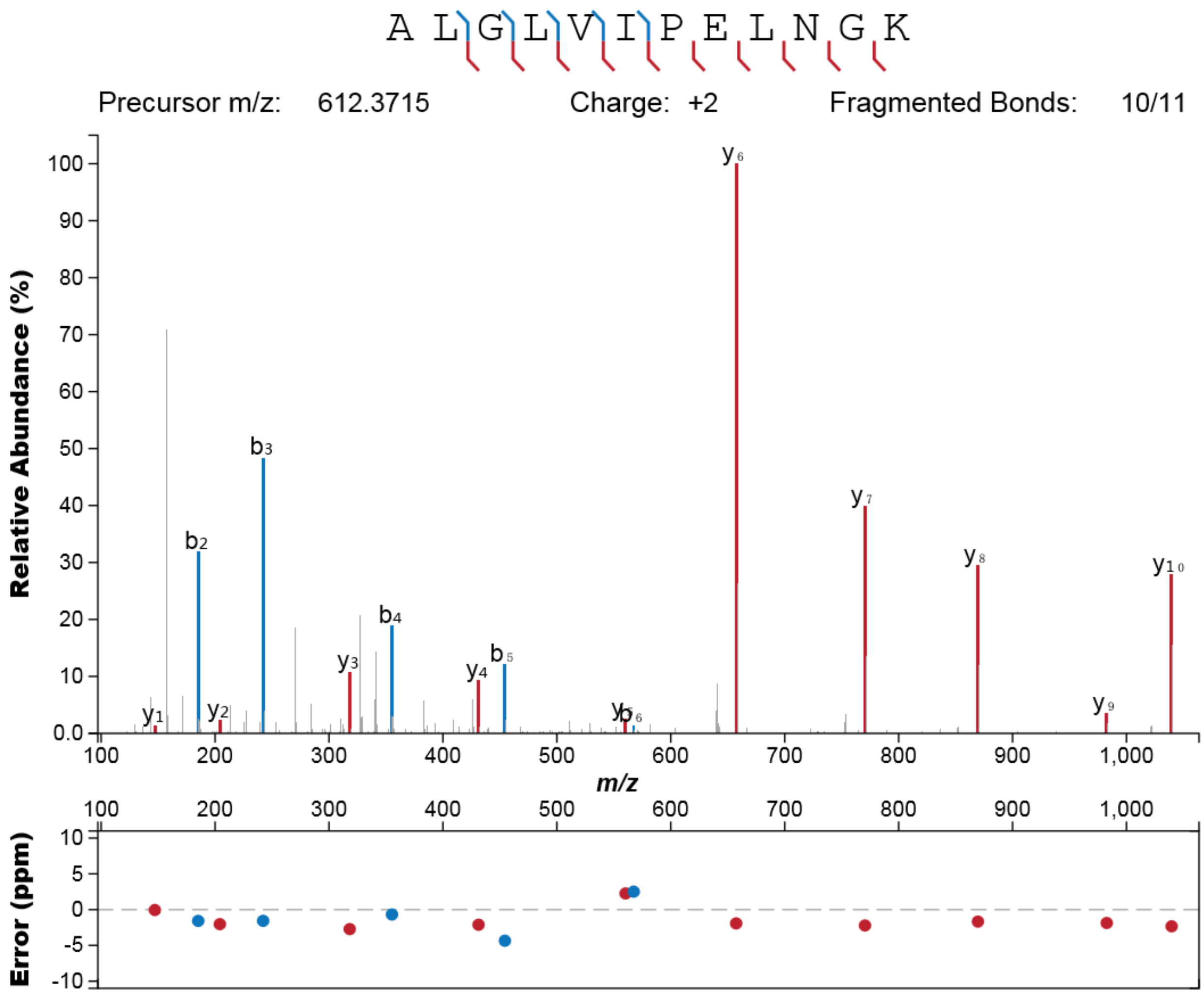

A L G L V I P E L N G K
Precursor m/z: 612.3715
Charge: +2
Fragmented Bonds: 10/11
Relative Abundance (%)
m/z
Error (ppm)
y1
y2
b2
b3
y3
b4
y4
b5
y5
b6
y6
y7
y8
y9
y10

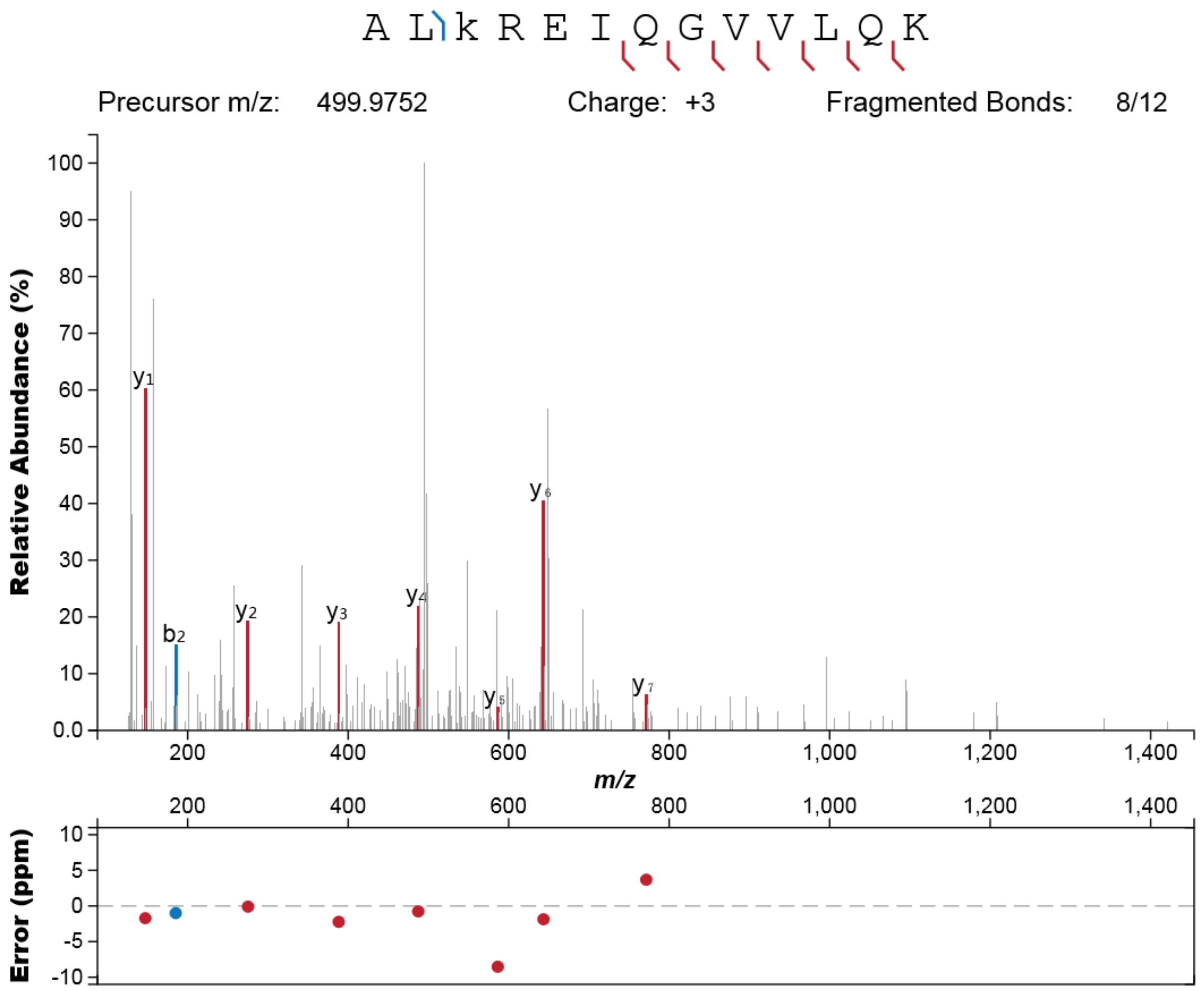

A L k R E I Q G V V L Q K
Precursor m/z: 499.9752
Charge: +3
Fragmented Bonds: 8/12
Relative Abundance (%)
y1
b2
y2
y3
y4
y5
y6
y7
m/z
Error (ppm)

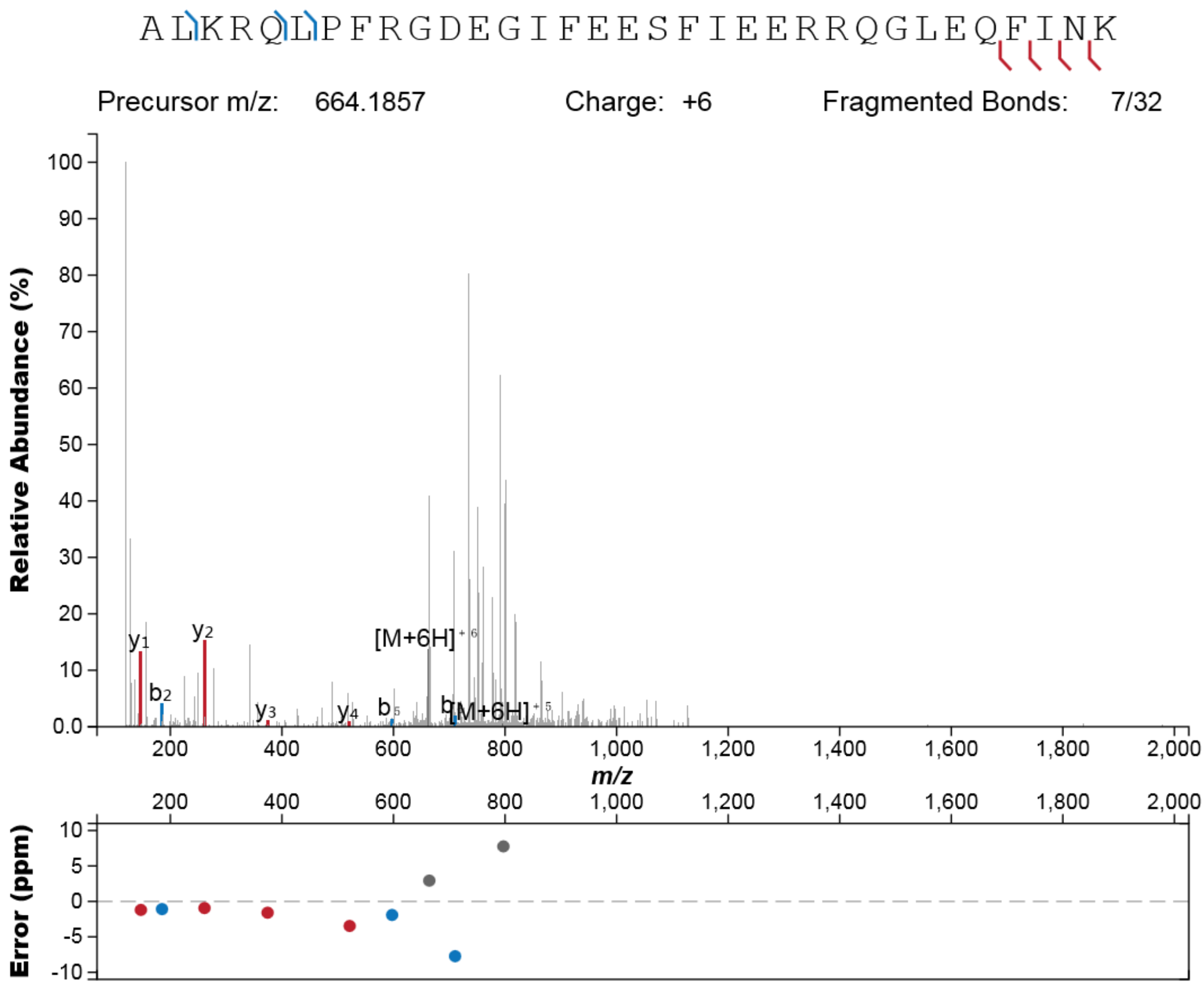

A L K R Q L P F R G D E G I F E E S F I E E R R Q G L E Q F I N K
Precursor m/z: 664.1857
Charge: +6
Fragmented Bonds: 7/32
Relative Abundance (%)
m/z
Error (ppm)
y1
y2
y3
y4
b2
b5
[M+6H]+6
[M+6H]+5

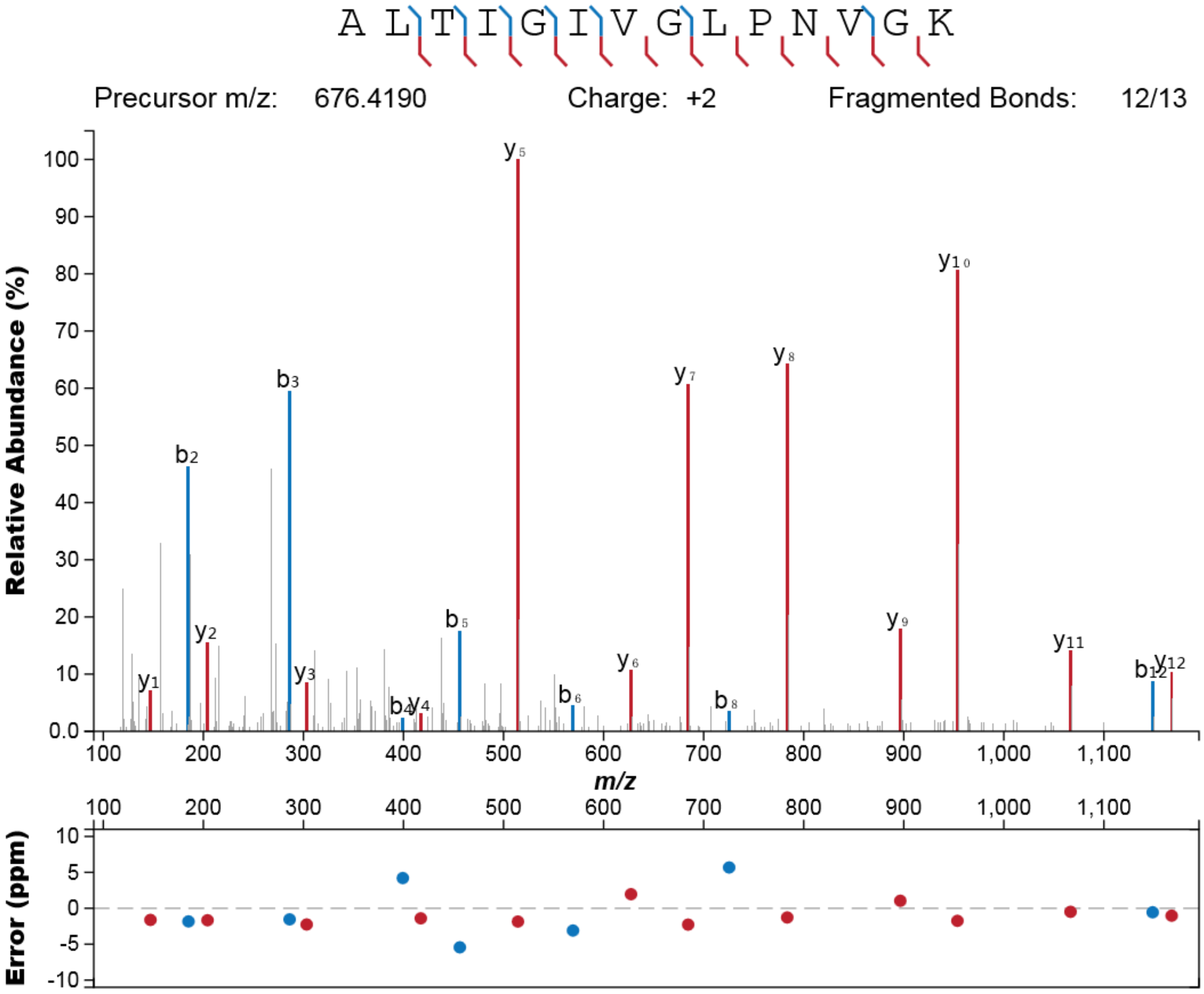

A L T I G I V G L P N V G K
Precursor m/z: 676.4190
Charge: +2
Fragmented Bonds: 12/13
Relative Abundance (%)
m/z
Error (ppm)
y5
y10
y8
y7
b3
b2
y2
y1
y3
b4
y4
b5
b6
y6
b8
y9
y11
b12
y12

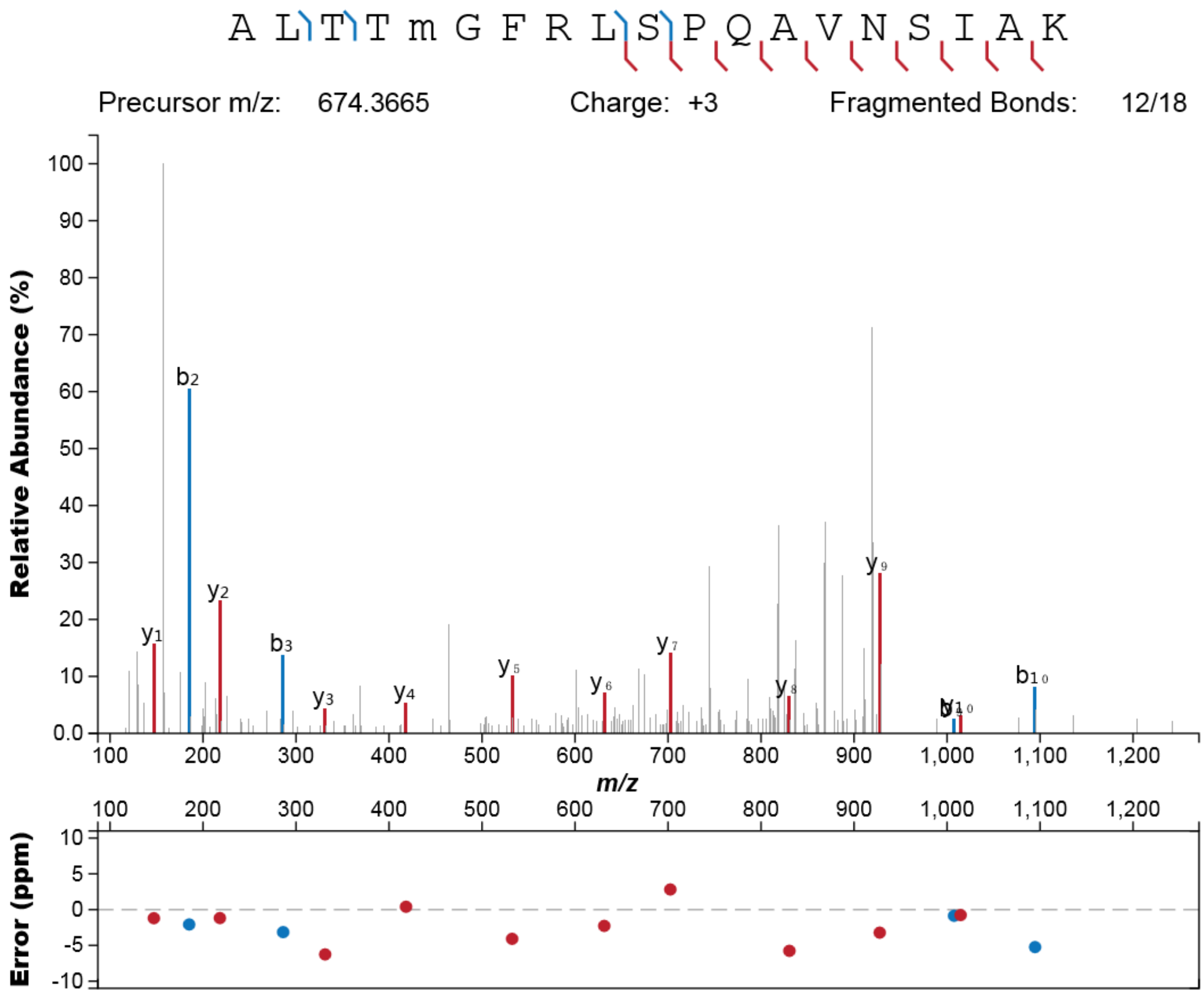

A L T T m G F R L S P Q A V N S I A K
Precursor m/z: 674.3665
Charge: +3
Fragmented Bonds: 12/18
Relative Abundance (%)
m/z
Error (ppm)

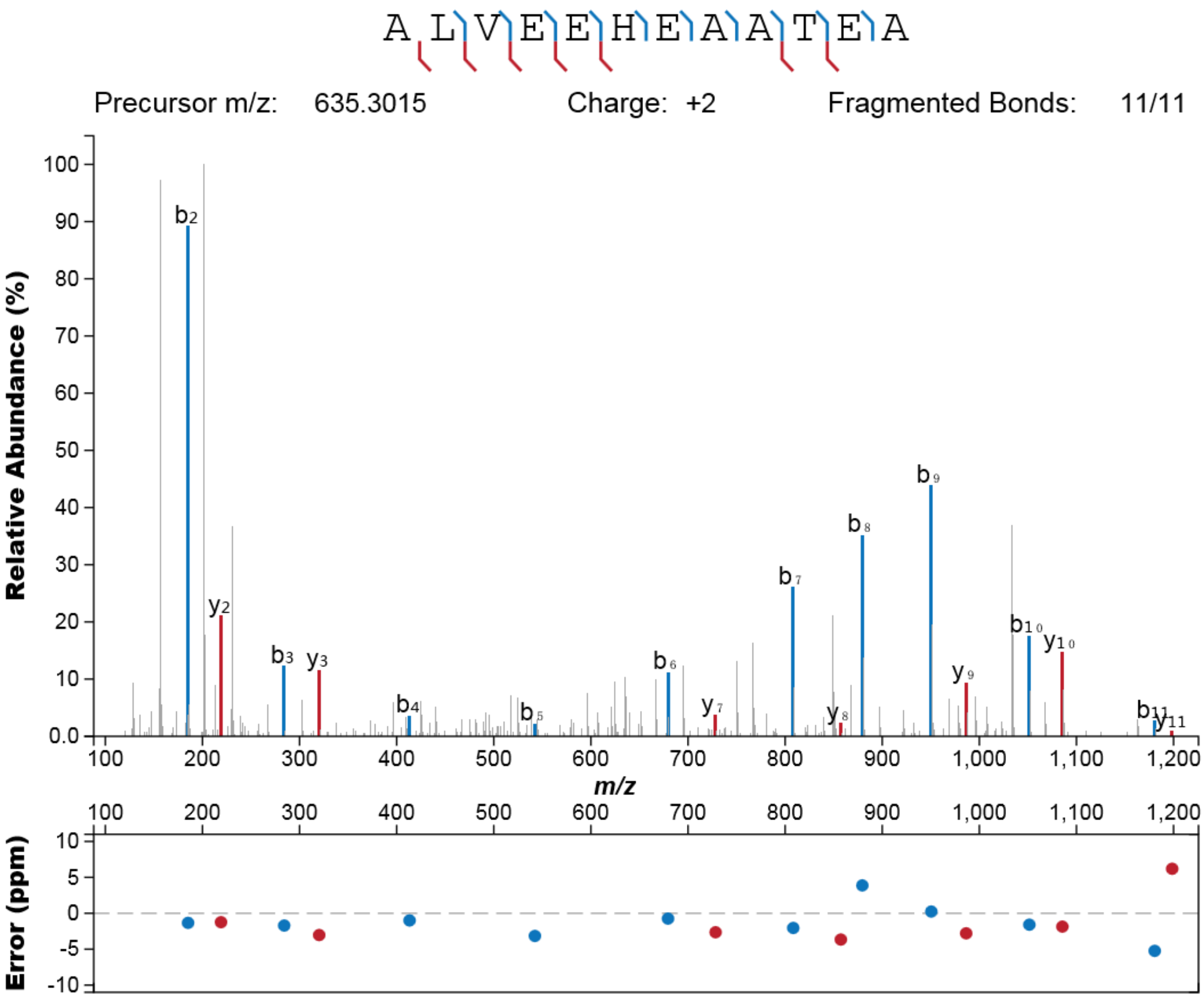
A L V E E H E A A T E A
Precursor m/z: 635.3015
Charge: +2
Fragmented Bonds: 11/11
Relative Abundance (%)
m/z
Error (ppm)
b2
y2
b3
y3
b4
b5
b6
y7
b7
y8
b8
b9
y9
b10
y10
b11
y11

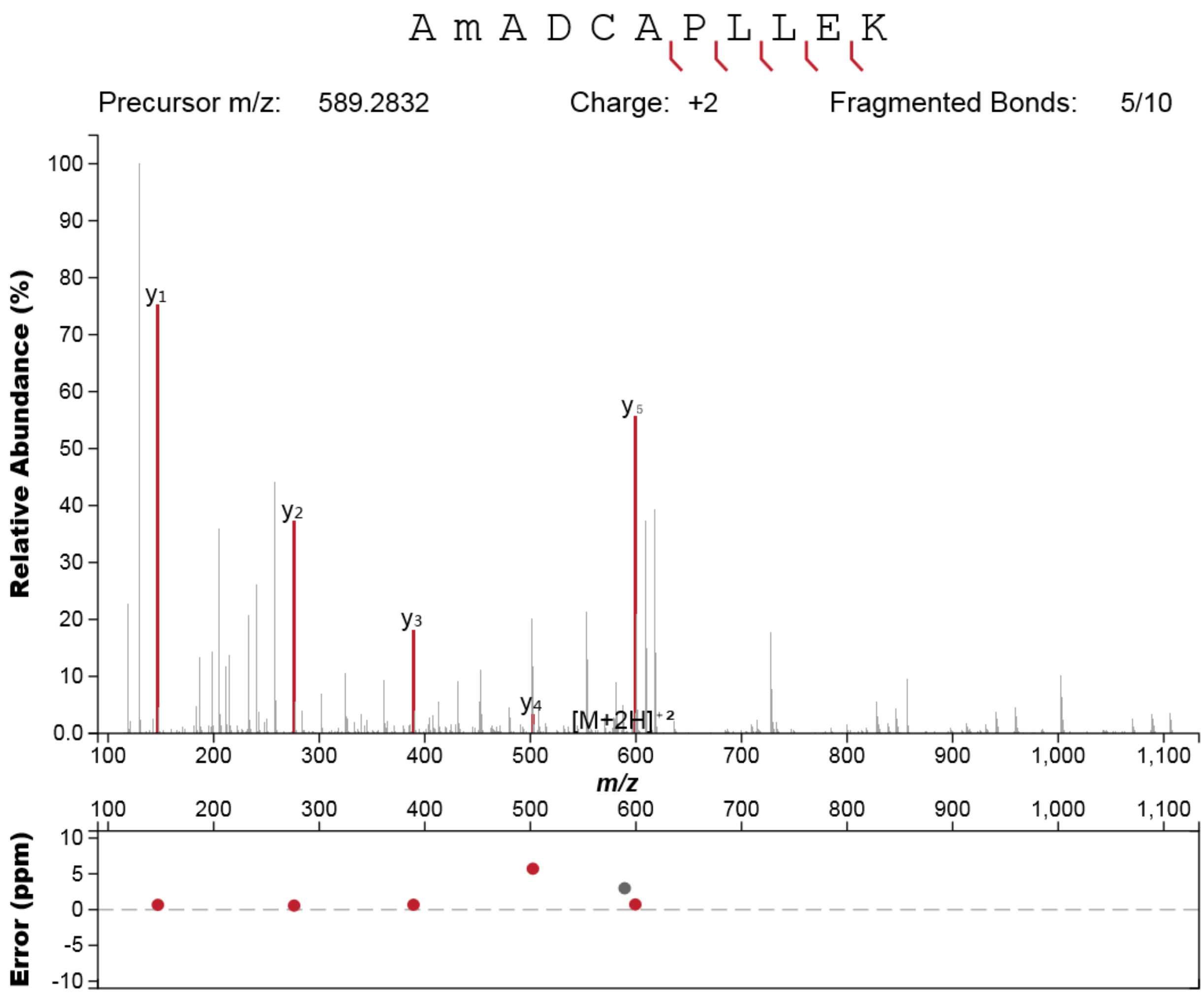

A m A D C A P L L E K
Precursor m/z: 589.2832
Charge: +2
Fragmented Bonds: 5/10
Relative Abundance (%)
y1
y2
y3
y4
y5
[M+2H]+2
m/z
Error (ppm)

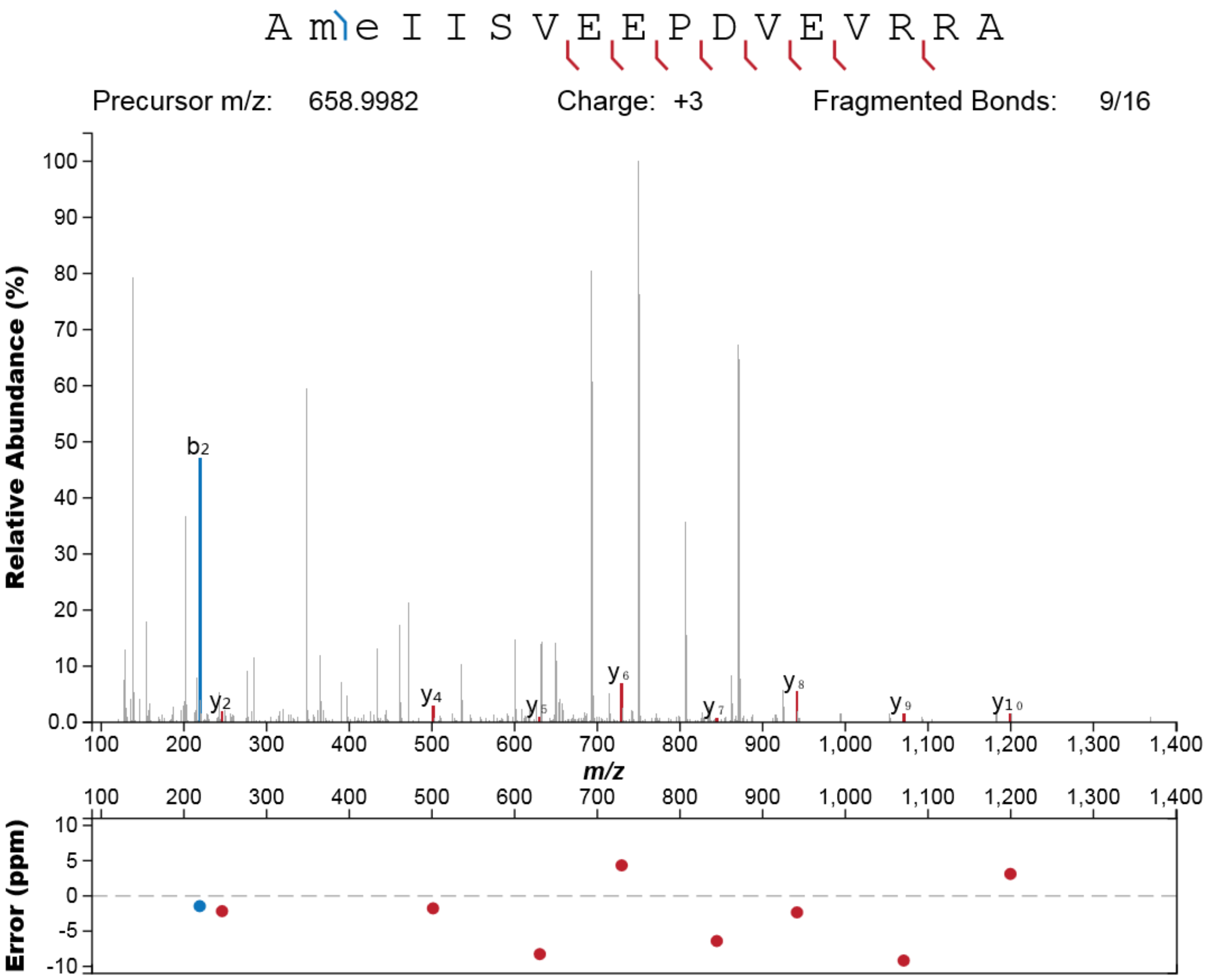
A m e I I S V E E P D V E V R R A
Precursor m/z: 658.9982
Charge: +3
Fragmented Bonds: 9/16
Relative Abundance (%)
m/z
Error (ppm)
b2
y2
y4
y5
y6
y7
y8
y9
y10

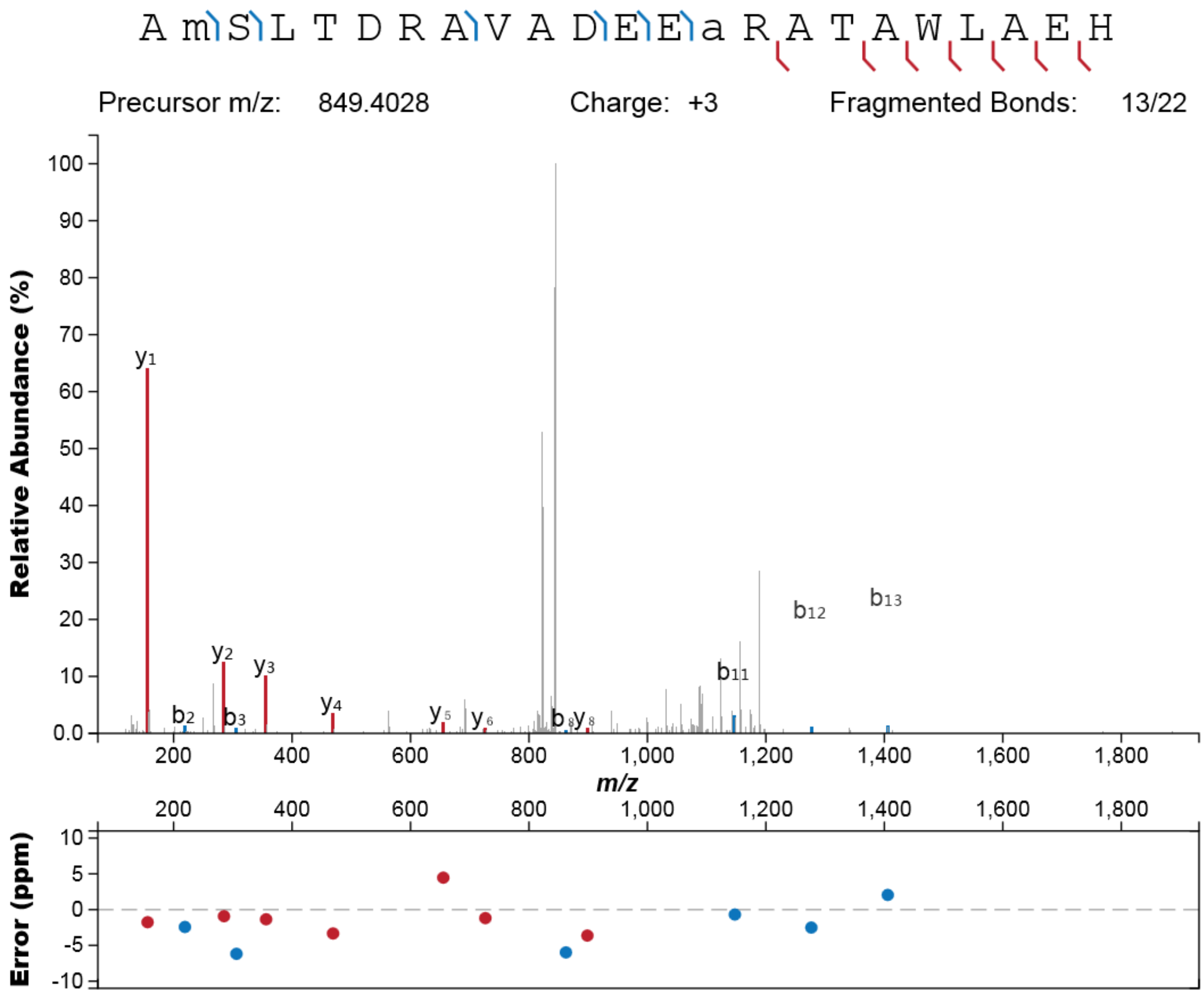
A m S L T D R A V A D E E a R A T A W L A E H
Precursor m/z: 849.4028
Charge: +3
Fragmented Bonds: 13/22
Relative Abundance (%)
m/z
Error (ppm)
y1
y2
y3
y4
y5
y6
y8
b2
b3
b8
b11
b12
b13

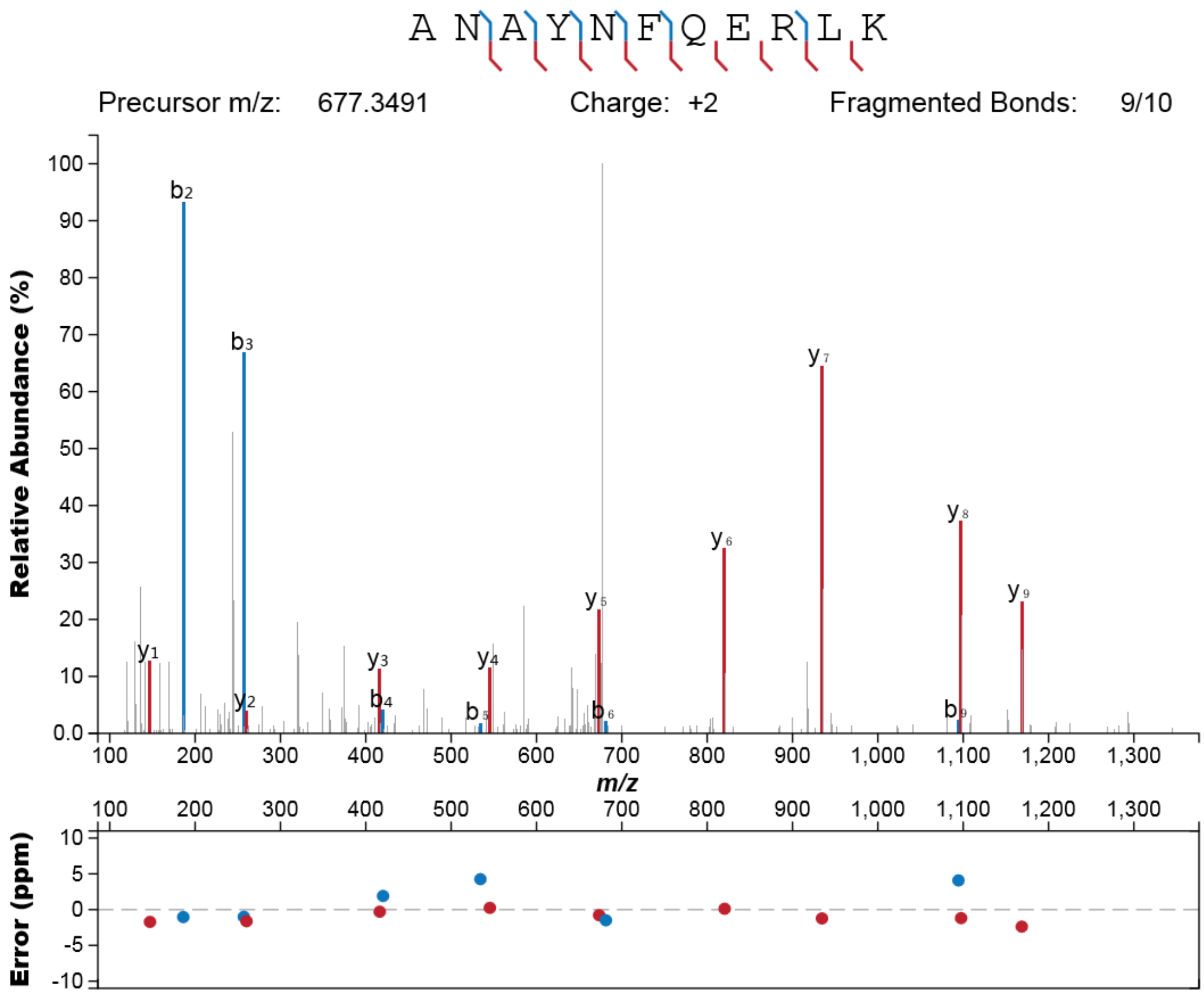
A N A Y N F Q E R L K
Precursor m/z: 677.3491
Charge: +2
Fragmented Bonds: 9/10
Relative Abundance (%)
m/z
Error (ppm)
b2
b3
b4
b5
b6
b9
y1
y2
y3
y4
y5
y6
y7
y8
y9

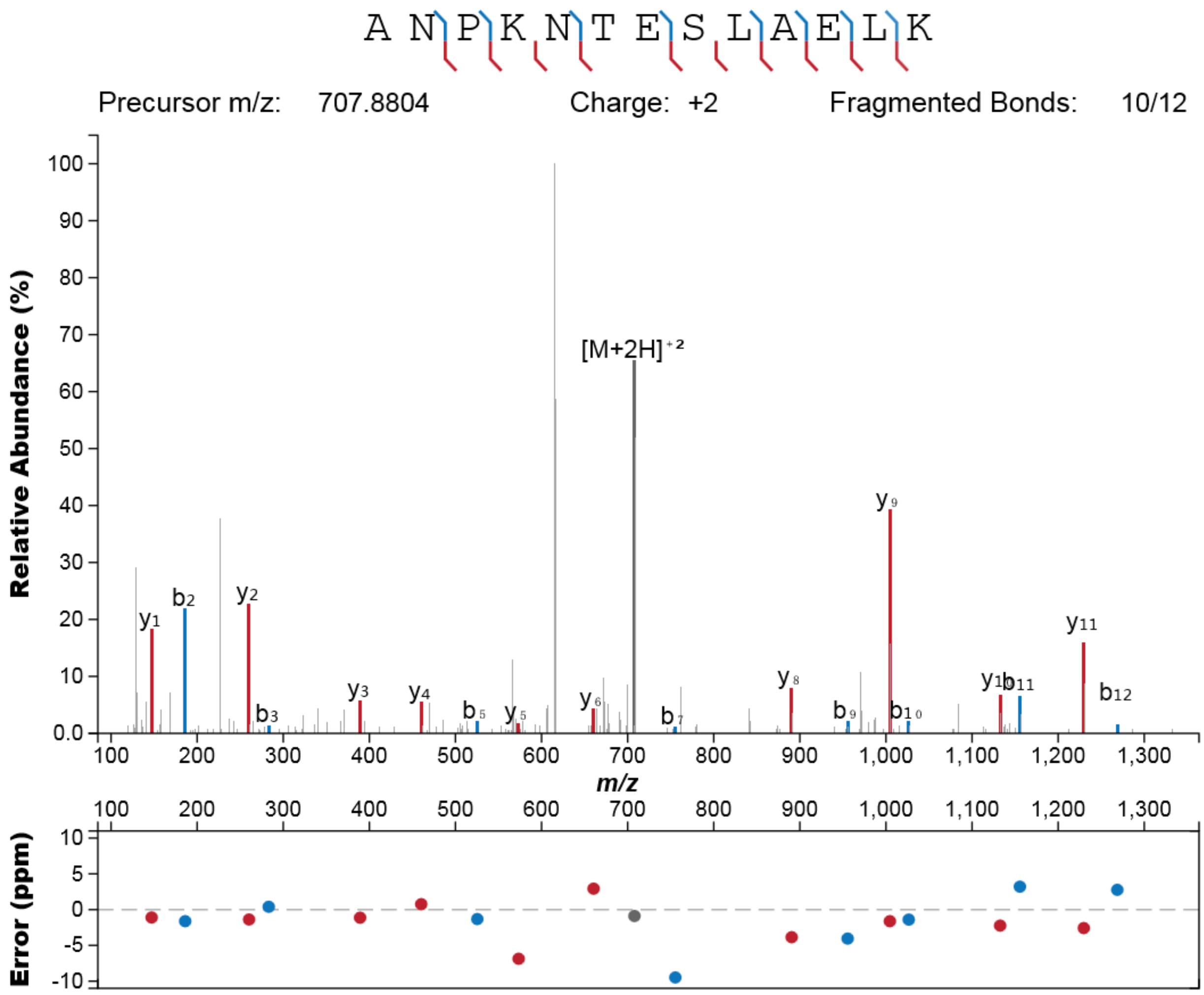

A N P K N T E S L A E L K
Precursor m/z: 707.8804
Charge: +2
Fragmented Bonds: 10/12
Relative Abundance (%)
[M+2H]+2
y1
b2
y2
b3
y3
y4
b5
y5
y6
b7
y8
b9
y9
b10
y10
b11
y11
b12
m/z
Error (ppm)

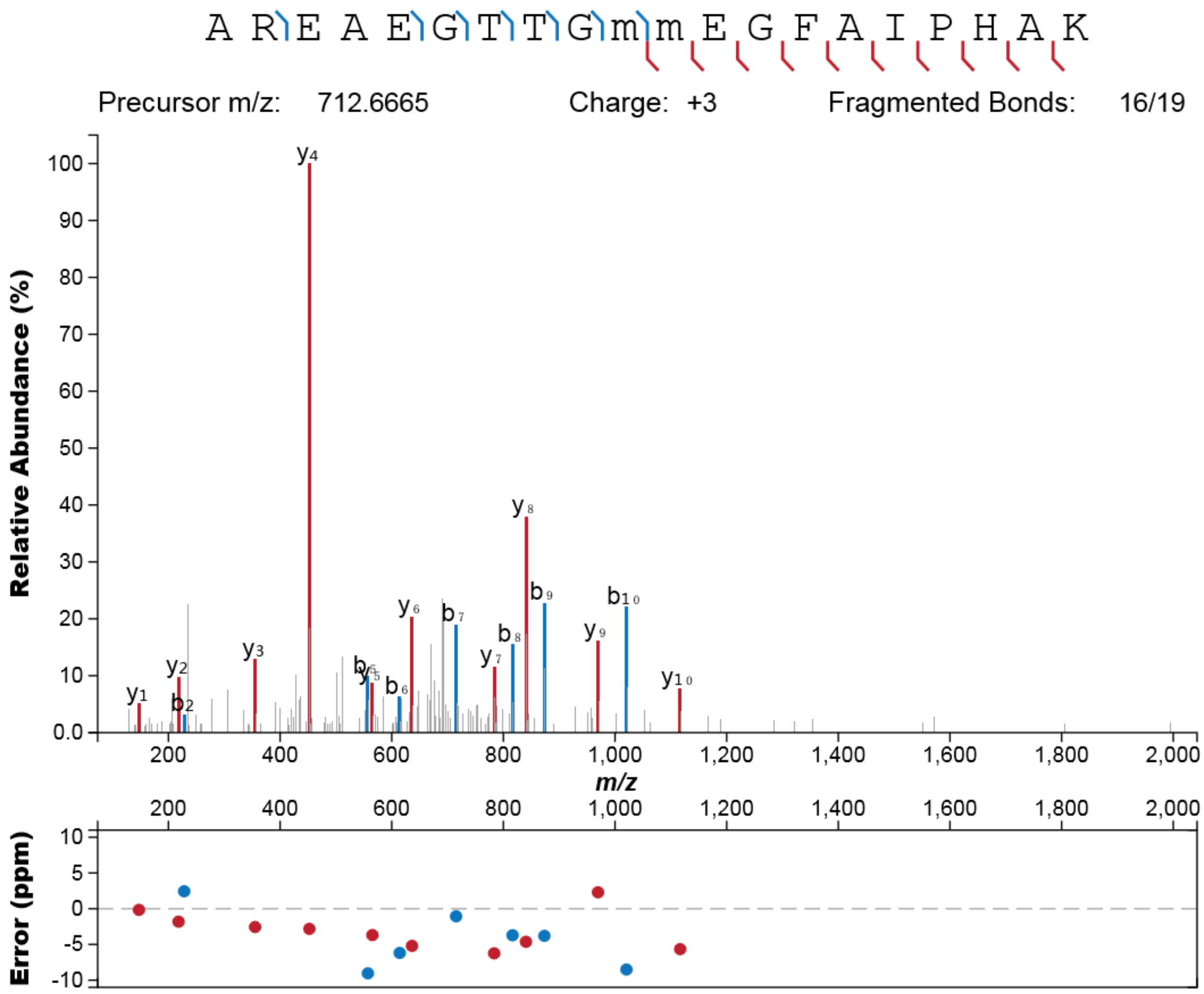
A R E A E G T T G m m E G F A I P H A K
Precursor m/z: 712.6665
Charge: +3
Fragmented Bonds: 16/19
Relative Abundance (%)
m/z
Error (ppm)
y4
y8
y6
b7
b9
b10
y9
y3
y2
y1
b2
b8
y7
b6
y5
b5
y10

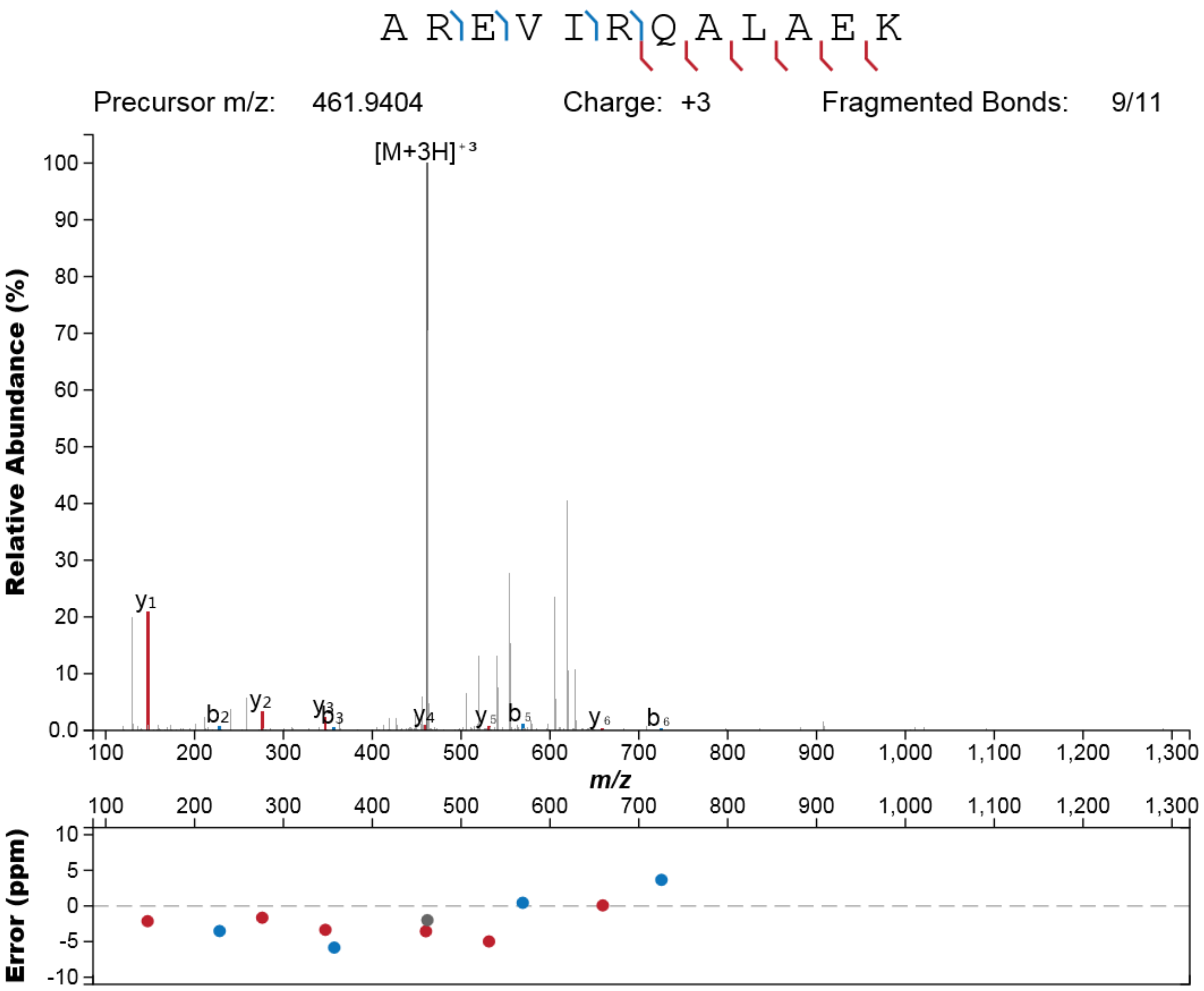

A R E V I R Q A L A E K
Precursor m/z: 461.9404
Charge: +3
Fragmented Bonds: 9/11
[M+3H]+3
Relative Abundance (%)
y1
b2
y2
y3
b3
y4
y5
b5
y6
b6
m/z
Error (ppm)

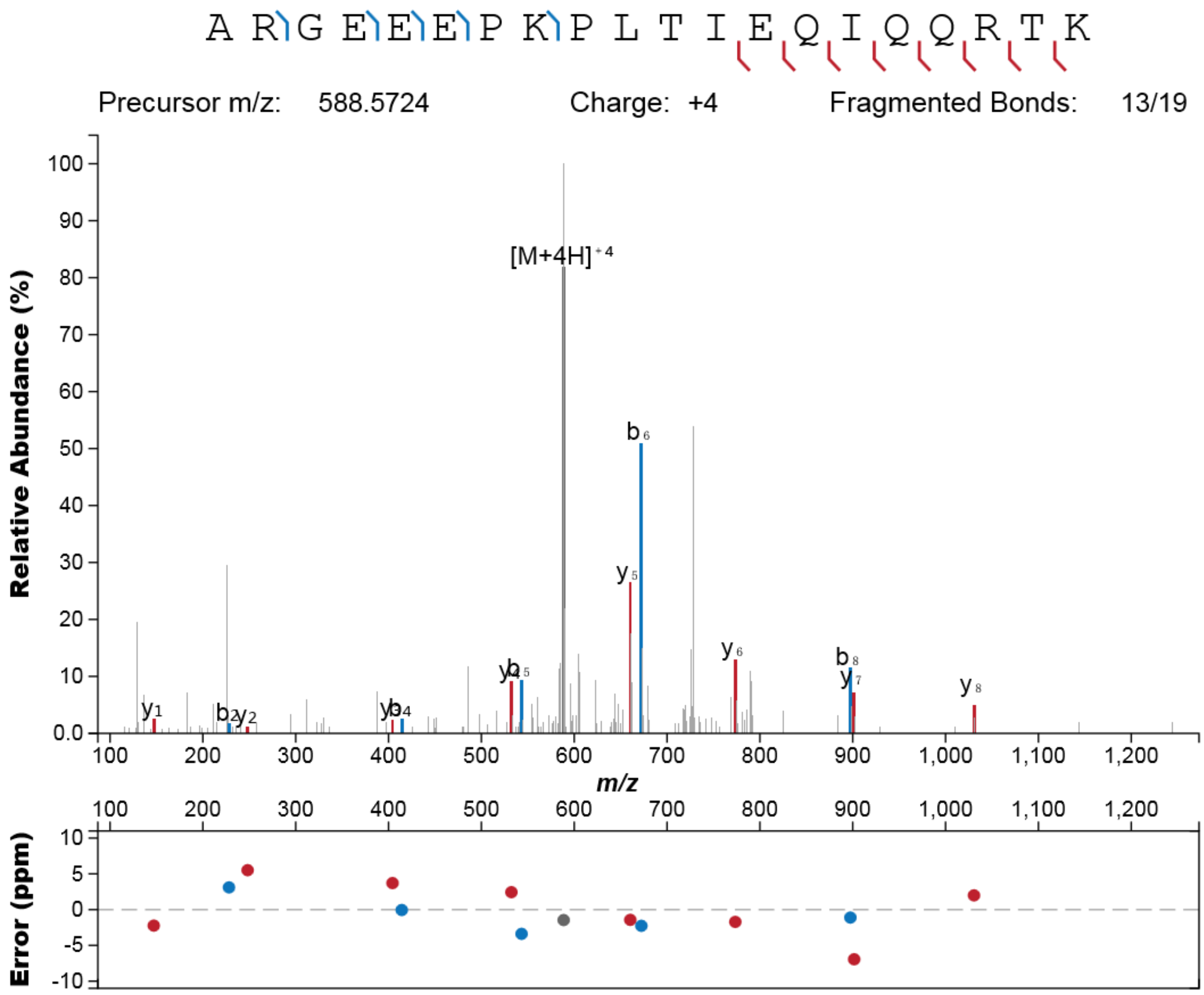
A R G E E E P K P L T I E Q I Q Q R T K
Precursor m/z: 588.5724
Charge: +4
Fragmented Bonds: 13/19
Relative Abundance (%)
[M+4H]+4
b6
y5
y6
b8
y7
y8
y1
b2
y2
y3
b4
y4
b5
m/z
Error (ppm)

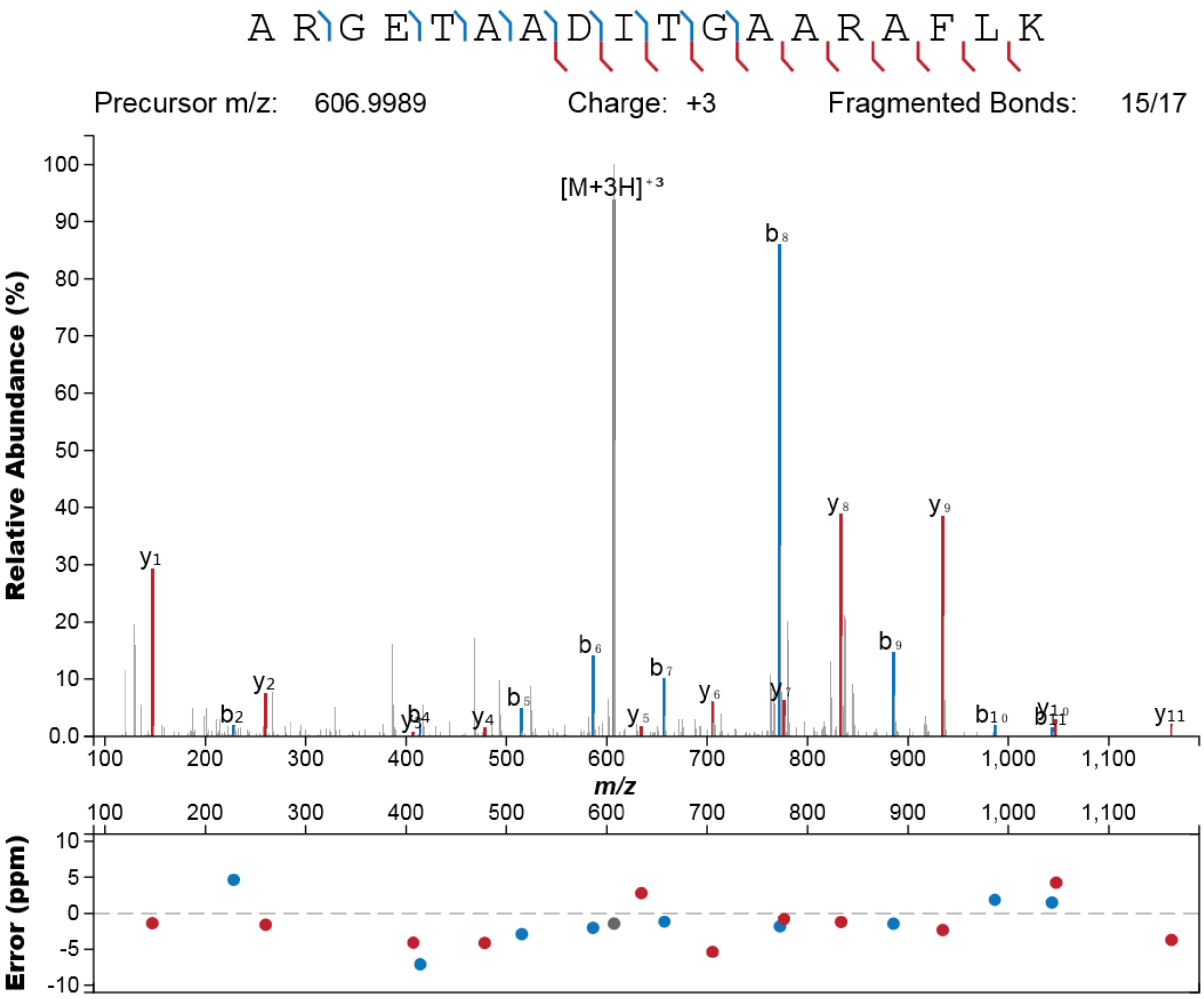
A R G E T A A D I T G A A R A F L K
Precursor m/z: 606.9989
Charge: +3
Fragmented Bonds: 15/17
[M+3H]+3
Relative Abundance (%)
m/z
Error (ppm)
y1 y2 y4 y5 y6 y7 y8 y9 y10 y11
b2 b4 b5 b6 b7 b8 b9 b10 b11

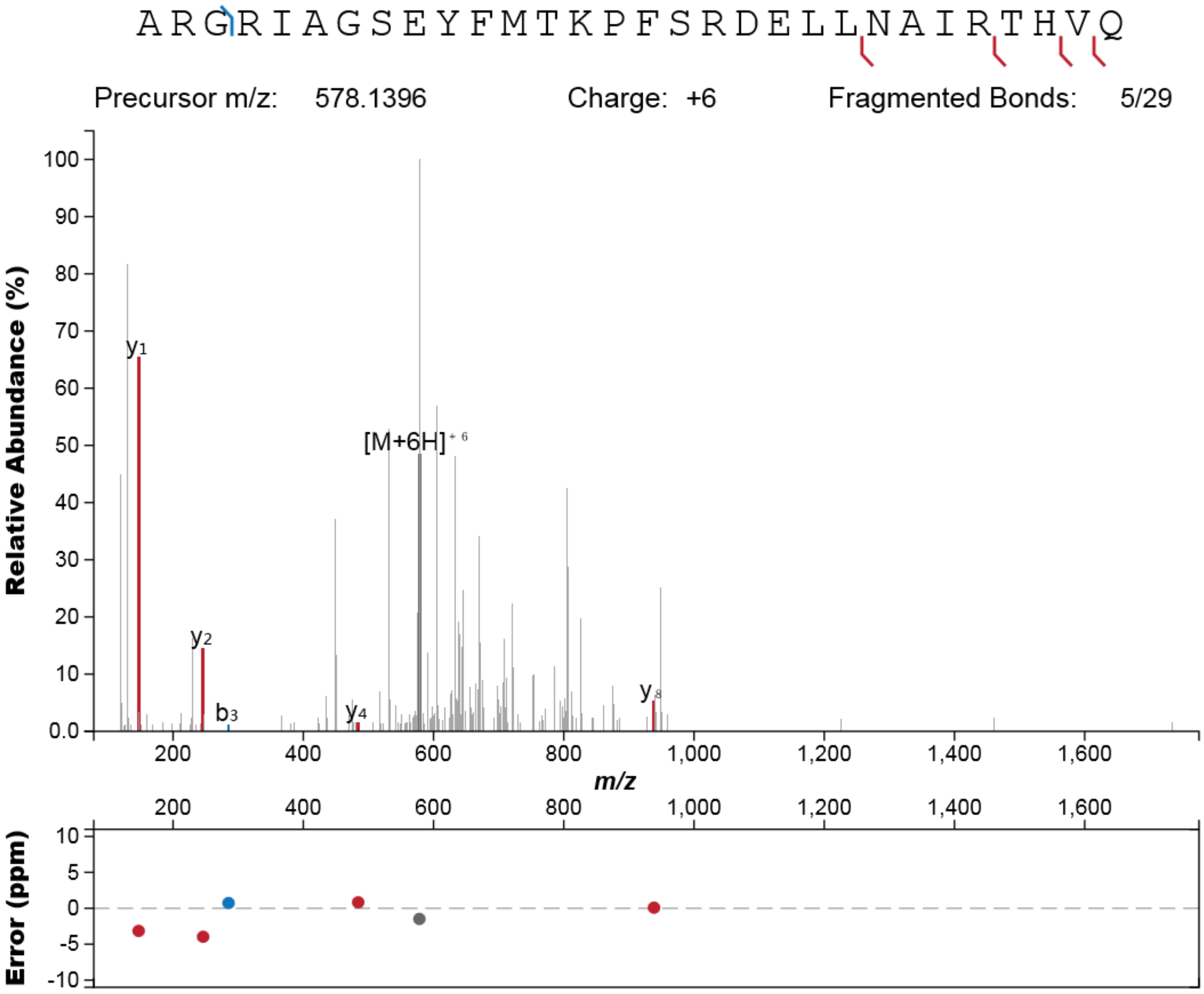

A R G R I A G S E Y F M T K P F S R D E L L N A I R T H V Q
Precursor m/z: 578.1396
Charge: +6
Fragmented Bonds: 5/29
Relative Abundance (%)
y1
y2
b3
y4
[M+6H]+6
y8
m/z
Error (ppm)

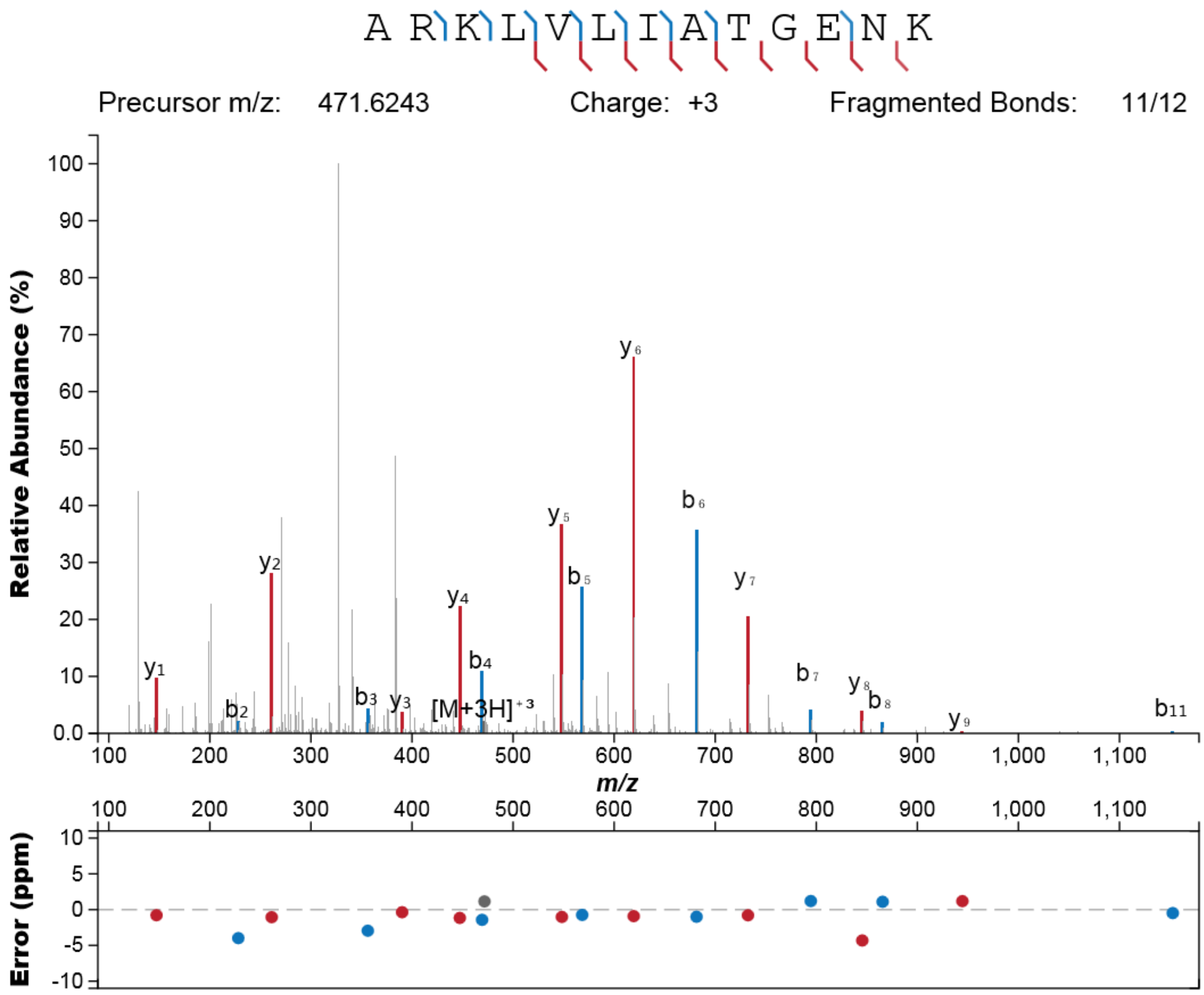
A R K L V L I A T G E N K
Precursor m/z: 471.6243
Charge: +3
Fragmented Bonds: 11/12
Relative Abundance (%)
m/z
Error (ppm)
y1
b2
y2
b3
y3
[M+3H]+3
y4
b4
y5
b5
y6
b6
y7
b7
y8
b8
y9
b11

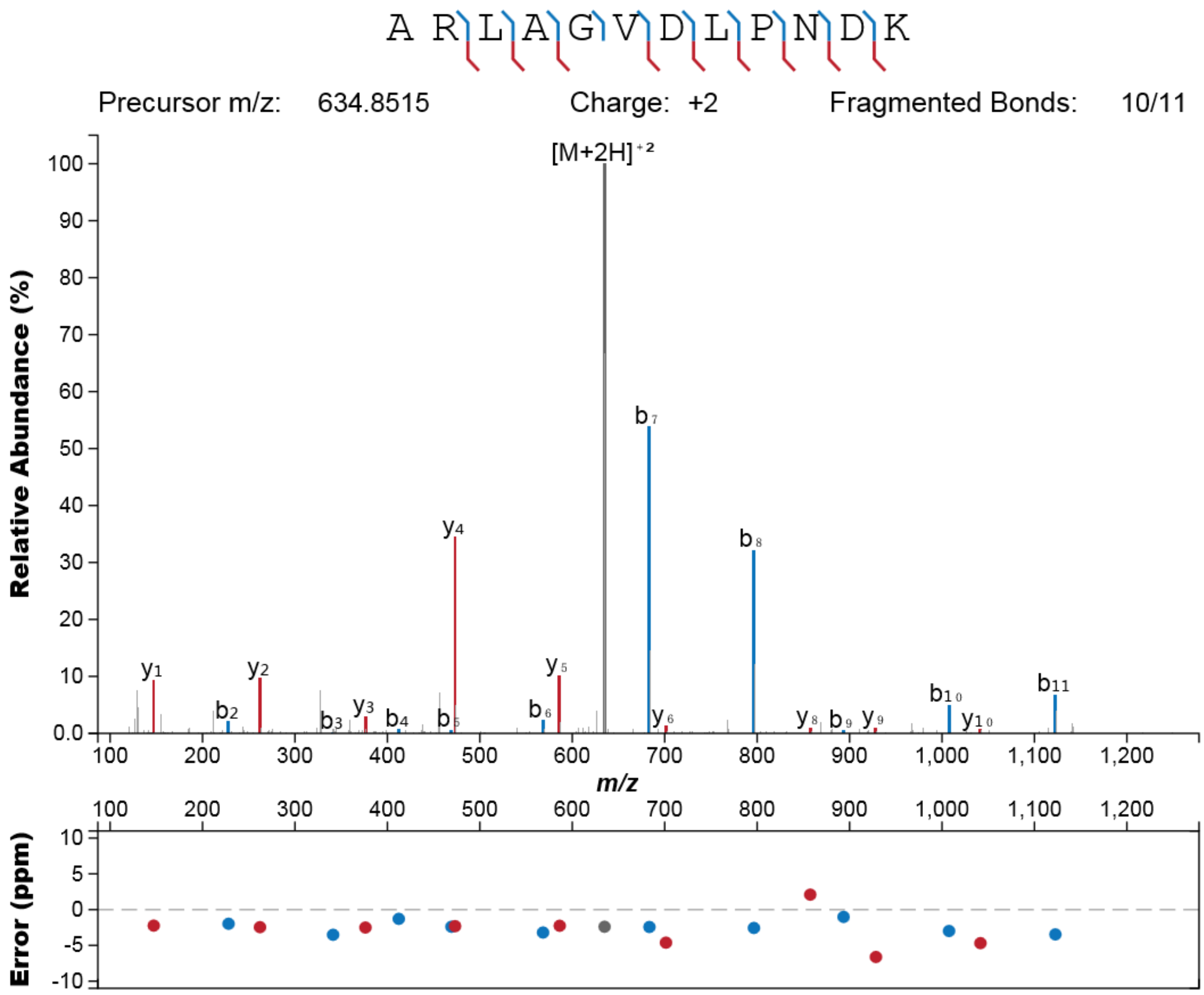
A R L A G V D L P N D K
Precursor m/z: 634.8515
Charge: +2
Fragmented Bonds: 10/11
[M+2H]+2
Relative Abundance (%)
m/z
Error (ppm)

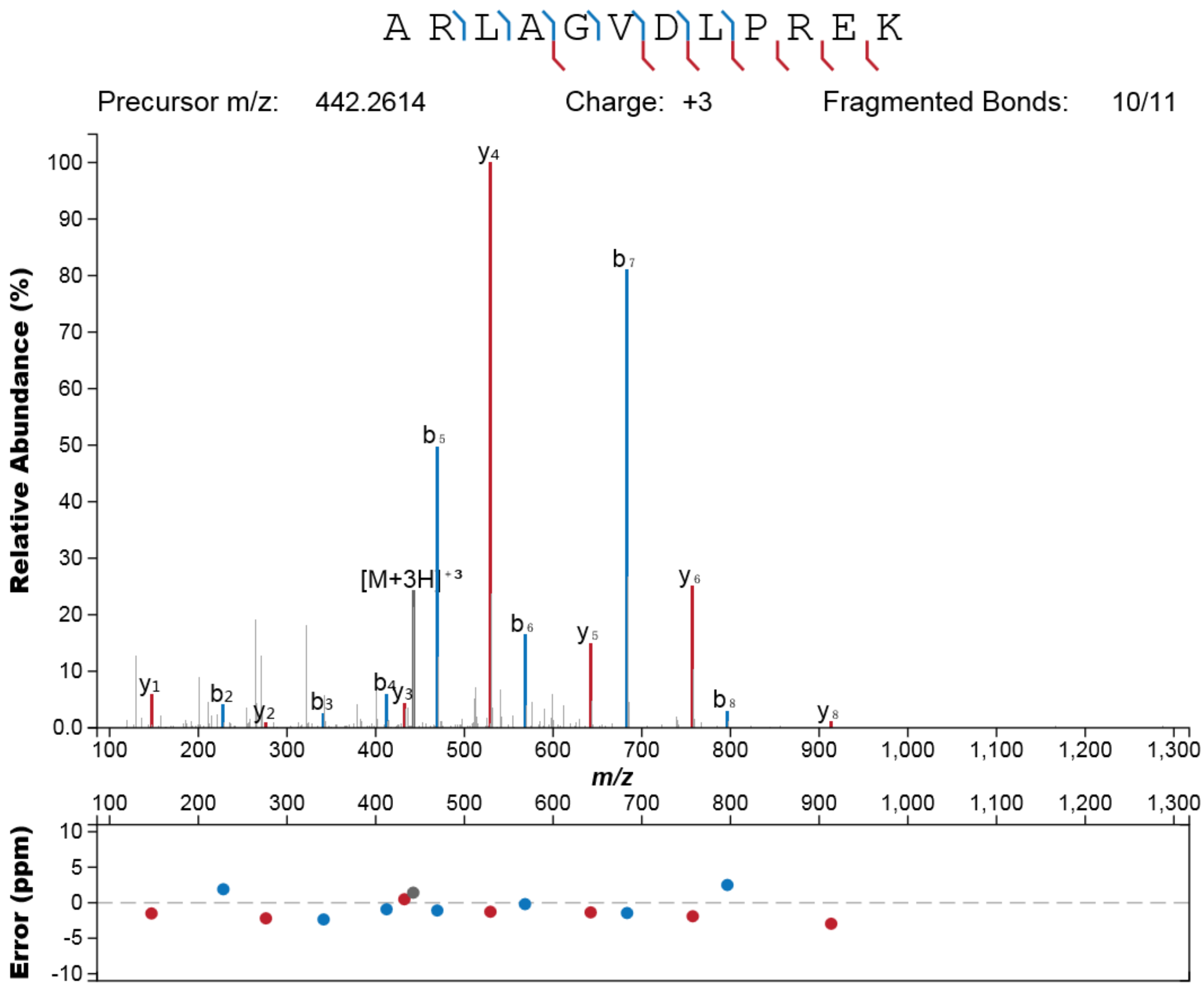
A R L A G V D L P R E K
Precursor m/z: 442.2614
Charge: +3
Fragmented Bonds: 10/11
Relative Abundance (%)
m/z
Error (ppm)
[M+3H]+3
y1
b2
y2
b3
b4
y3
b5
y4
b6
y5
b7
y6
b8
y8

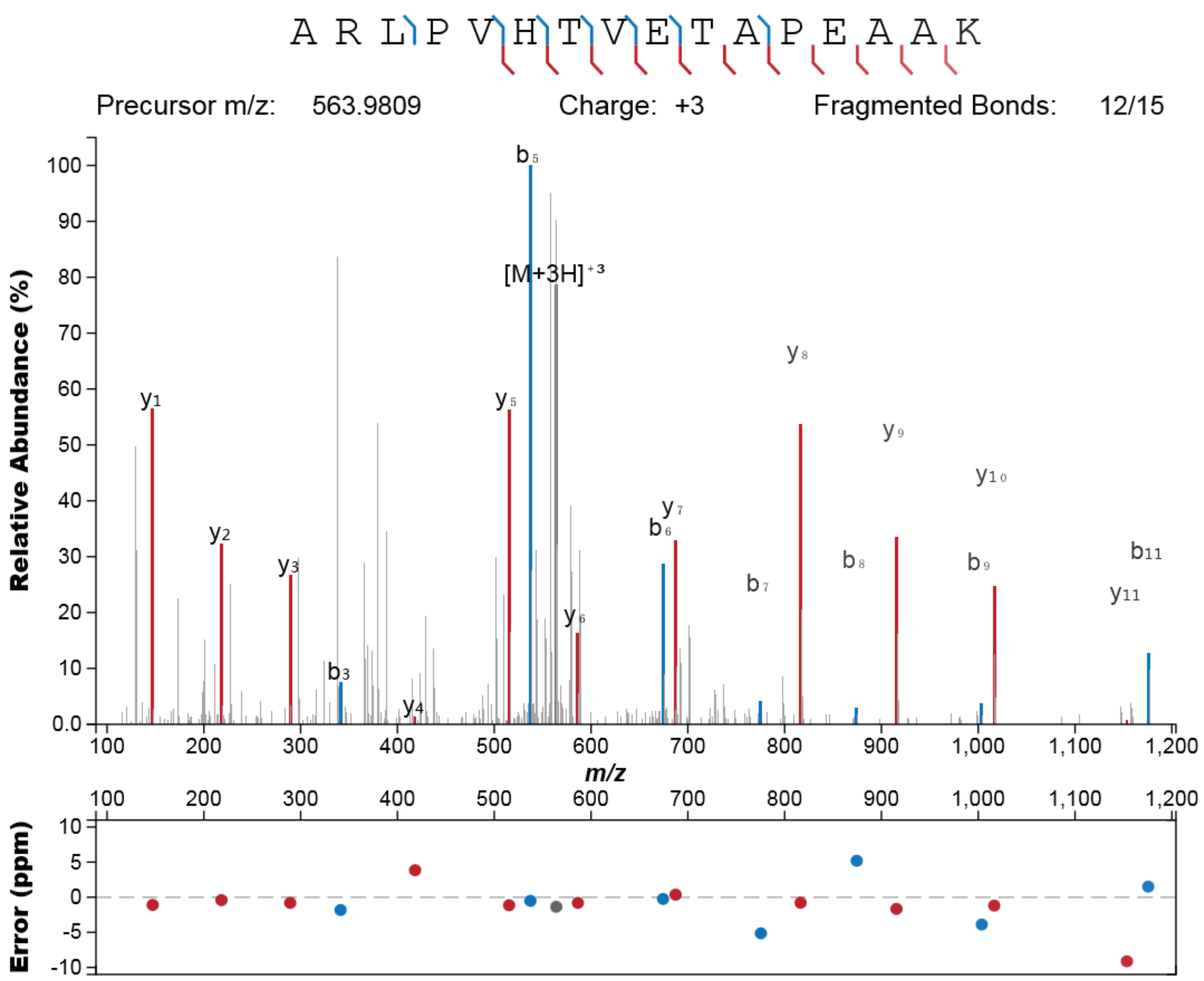

A R L P V H T V E T A P E A A K
Precursor m/z: 563.9809
Charge: +3
Fragmented Bonds: 12/15
Relative Abundance (%)
m/z
Error (ppm)
[M+3H]+3

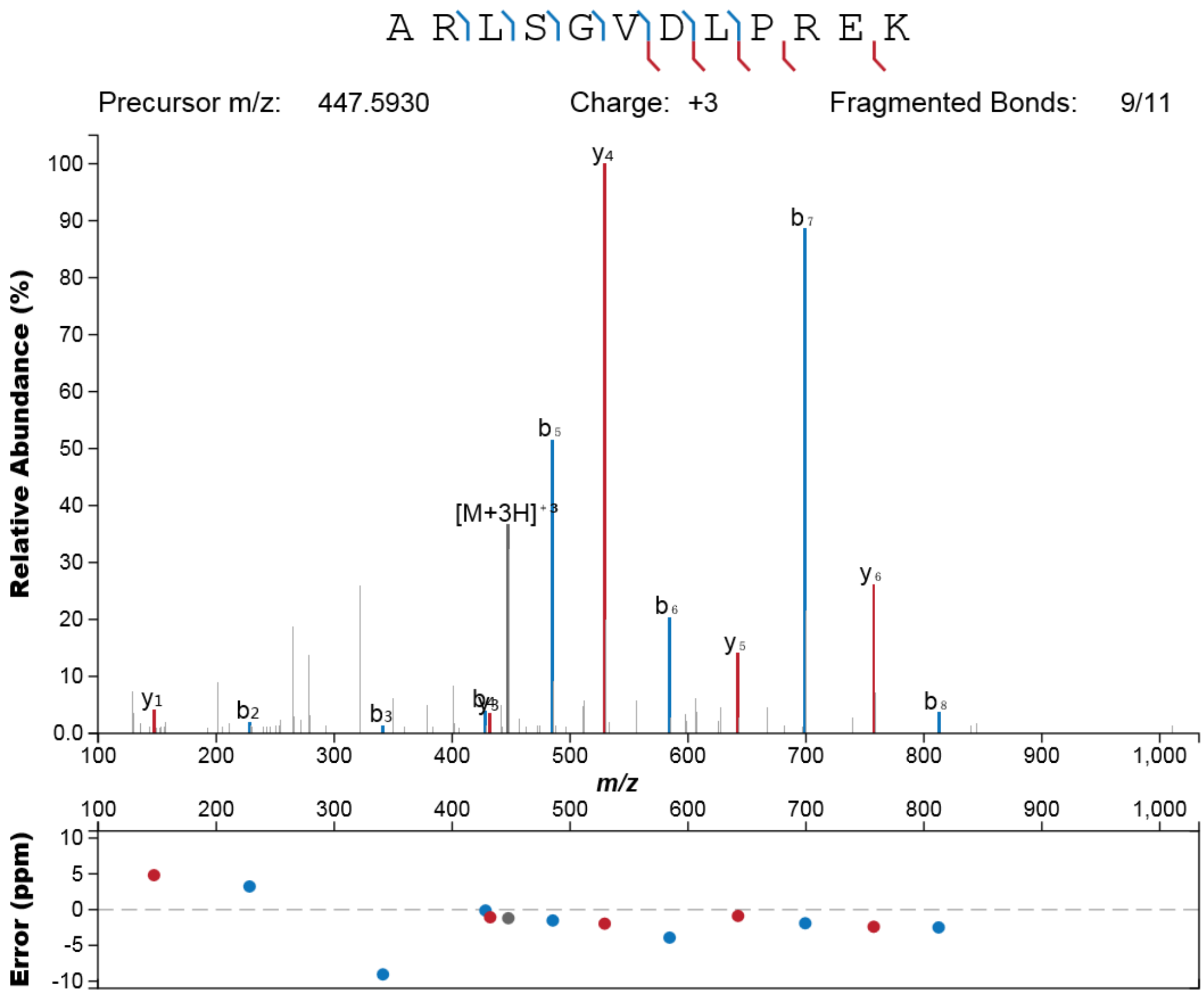

A R L S G V D L P R E K
Precursor m/z: 447.5930
Charge: +3
Fragmented Bonds: 9/11
Relative Abundance (%)
m/z
Error (ppm)
[M+3H]+3
y4
b7
b5
y6
b6
y5
y1
b2
b3
b8

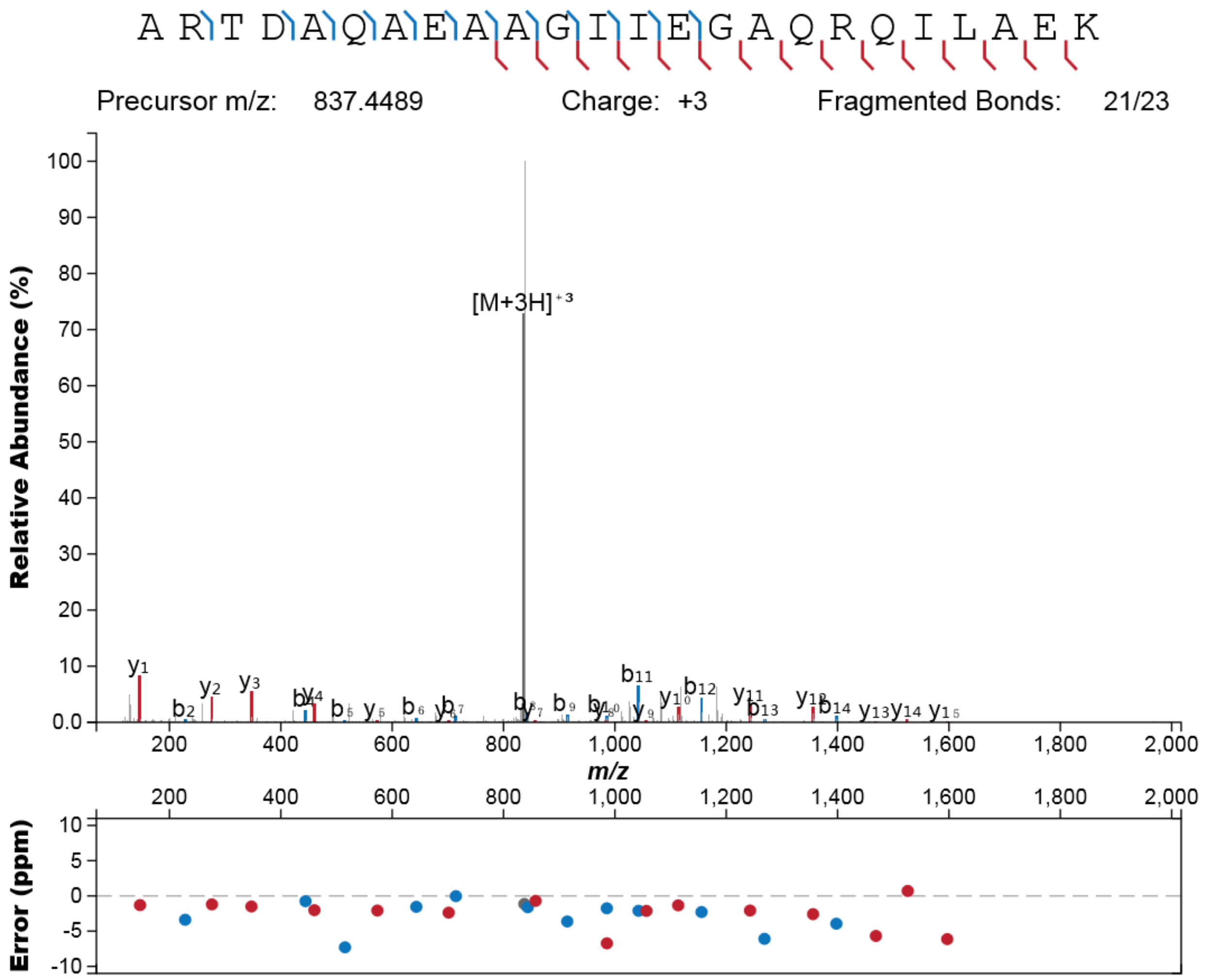

A R T D A Q A E A A G I I E G A Q R Q I L A E K
Precursor m/z: 837.4489
Charge: +3
Fragmented Bonds: 21/23
[M+3H]+3
Relative Abundance (%)
m/z
Error (ppm)

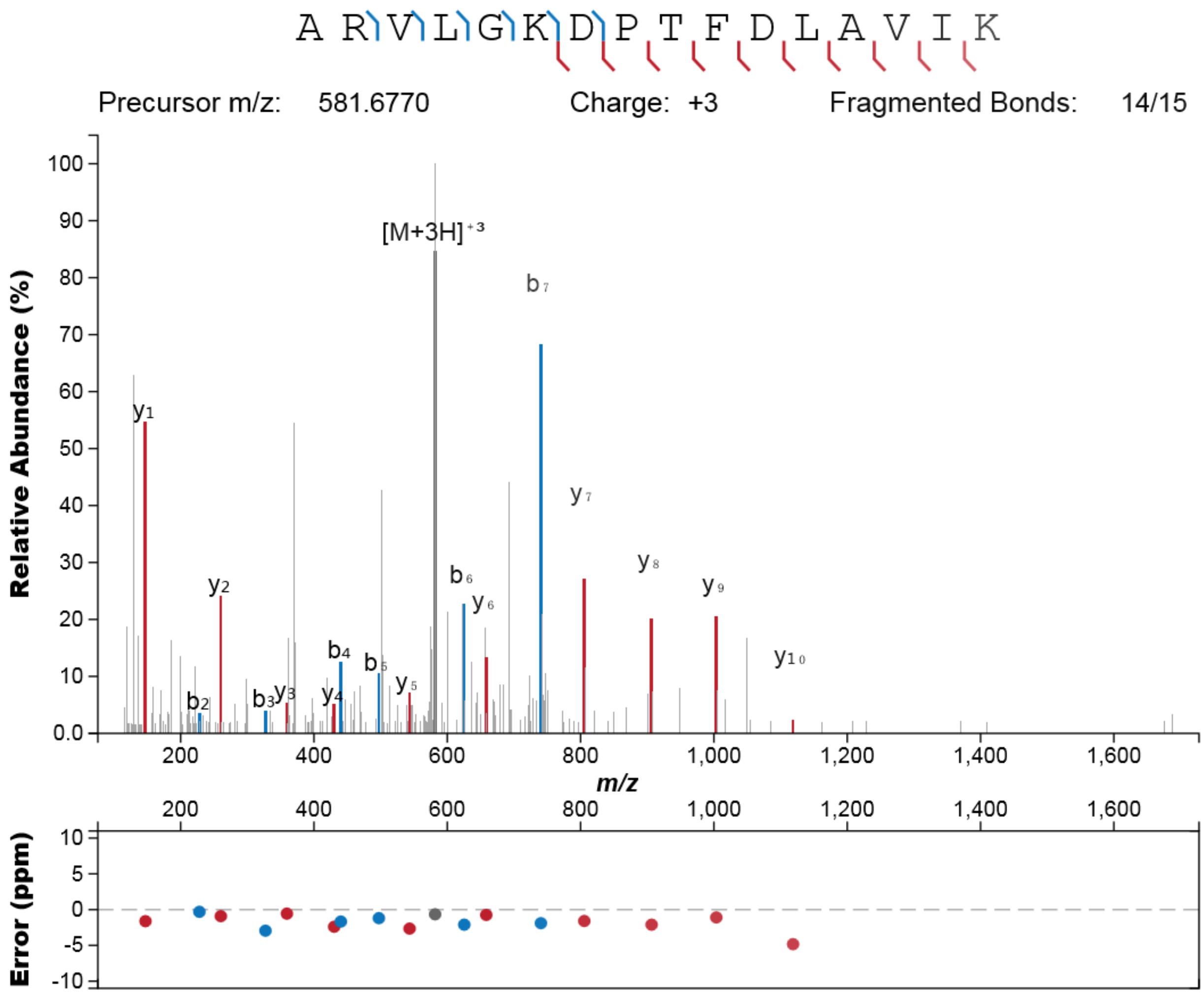
A R V L G K D P T F D L A V I K
Precursor m/z: 581.6770
Charge: +3
Fragmented Bonds: 14/15
Relative Abundance (%)
[M+3H]+3
y1
y2
b2
b3
y3
y4
b4
b5
y5
b6
y6
b7
y7
y8
y9
y10
m/z
Error (ppm)

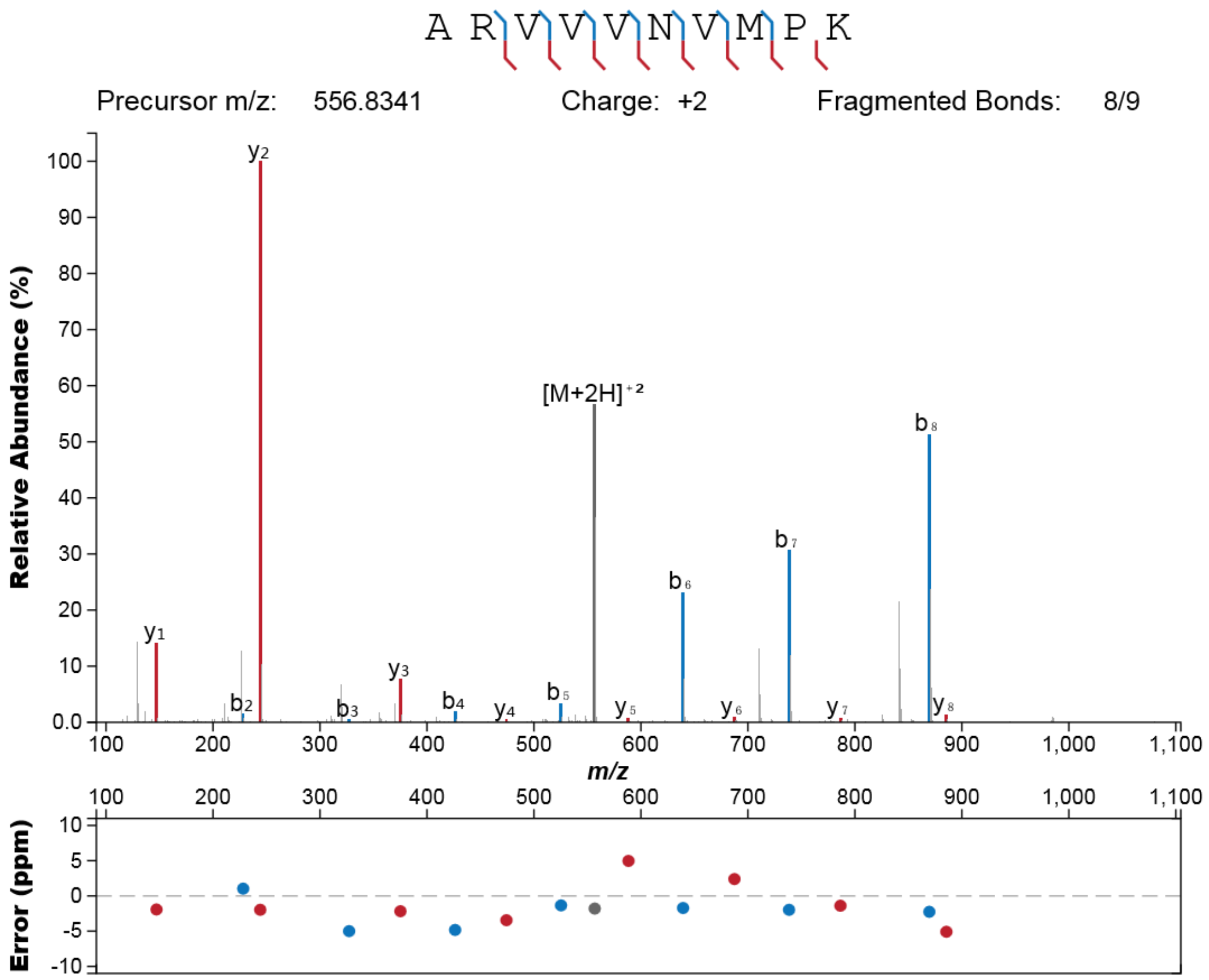
A R V V V N V M P K
Precursor m/z: 556.8341
Charge: +2
Fragmented Bonds: 8/9
Relative Abundance (%)
m/z
Error (ppm)
[M+2H]+2

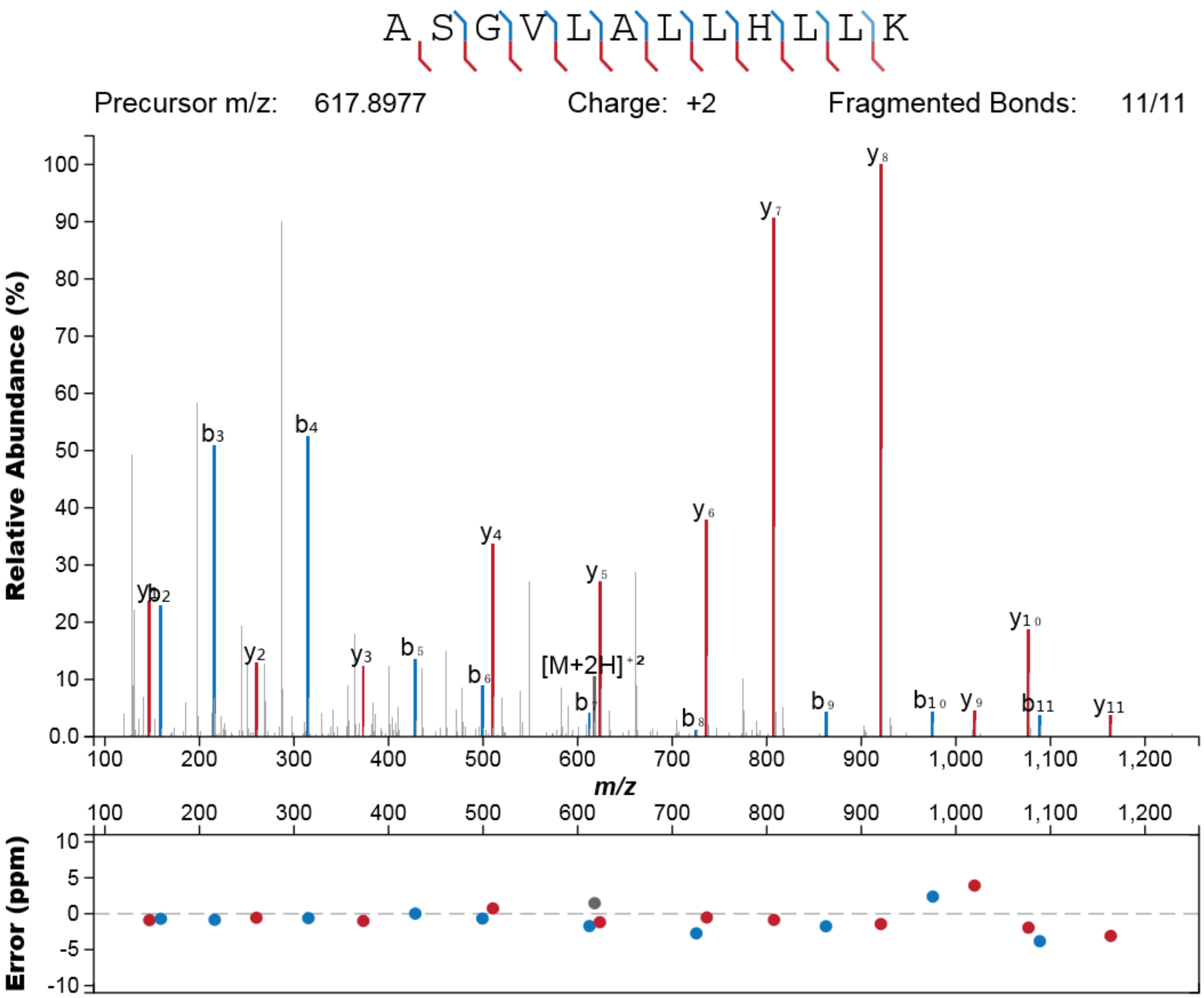

A S G V L A L L H L L K
Precursor m/z: 617.8977
Charge: +2
Fragmented Bonds: 11/11
Relative Abundance (%)
m/z
Error (ppm)
y8
y7
y6
y5
y4
y3
y2
y1
b2
b3
b4
b5
b6
b7
b8
b9
b10
y9
y10
b11
y11
[M+2H]+2

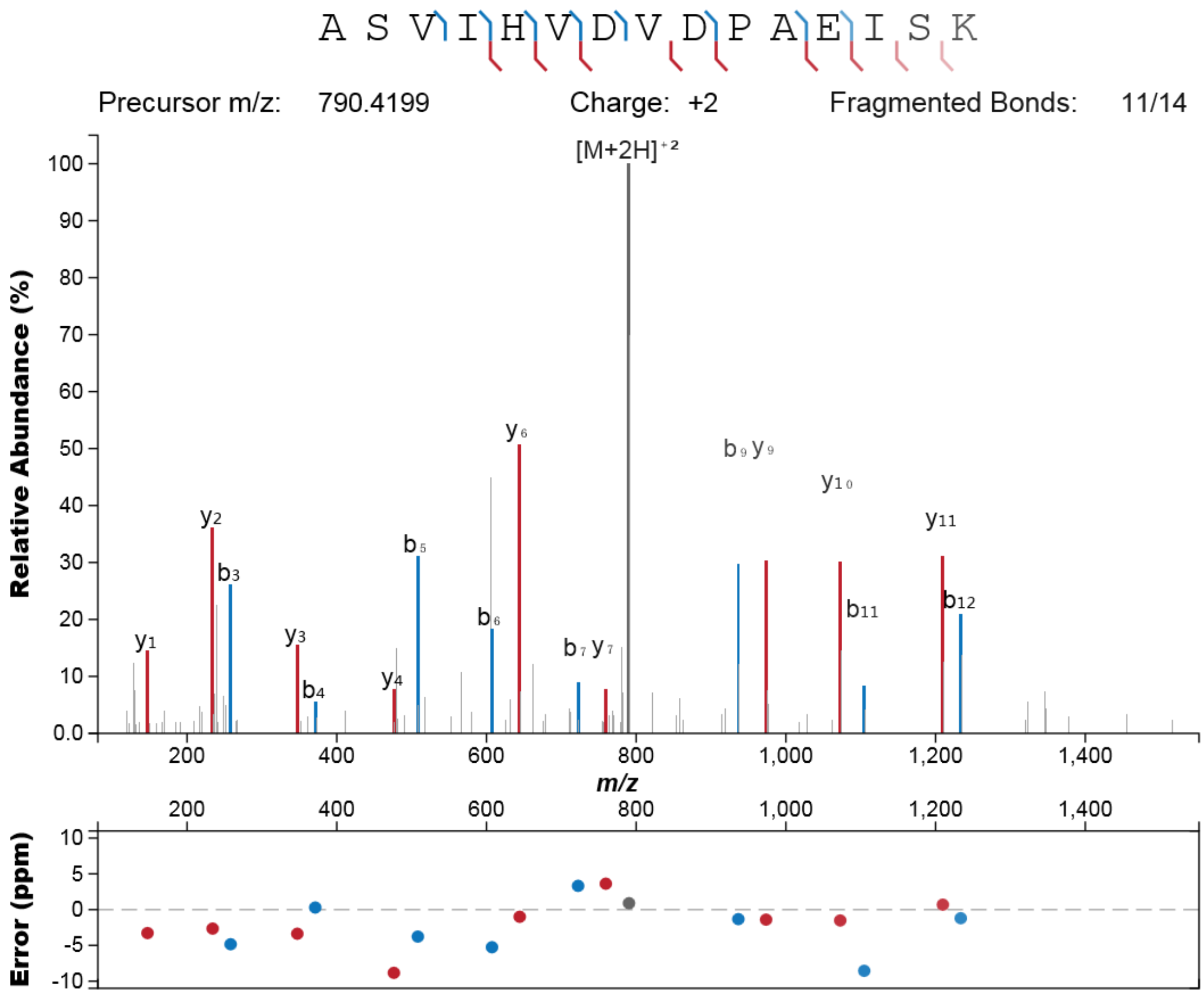
A S V I H V D V D P A E I S K
Precursor m/z: 790.4199
Charge: +2
Fragmented Bonds: 11/14
[M+2H]+2
Relative Abundance (%)
y1
y2
b3
y3
b4
y4
b5
b6
y6
b7 y7
b9 y9
y10
b11
y11
b12
m/z
Error (ppm)

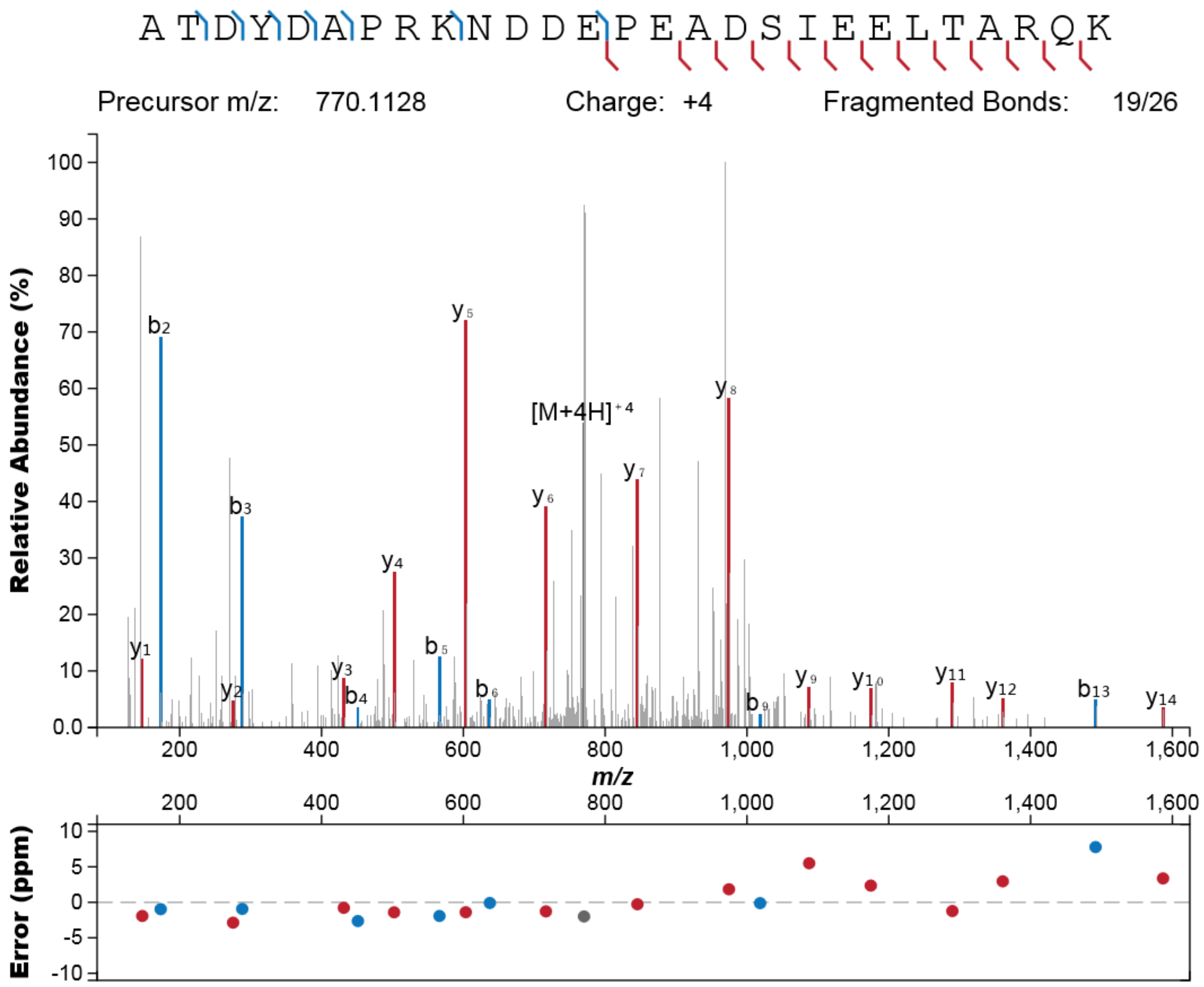
A T D Y D A P R K N D D E P E A D S I E E L T A R Q K
Precursor m/z: 770.1128
Charge: +4
Fragmented Bonds: 19/26
Relative Abundance (%)
m/z
[M+4H]+4
Error (ppm)

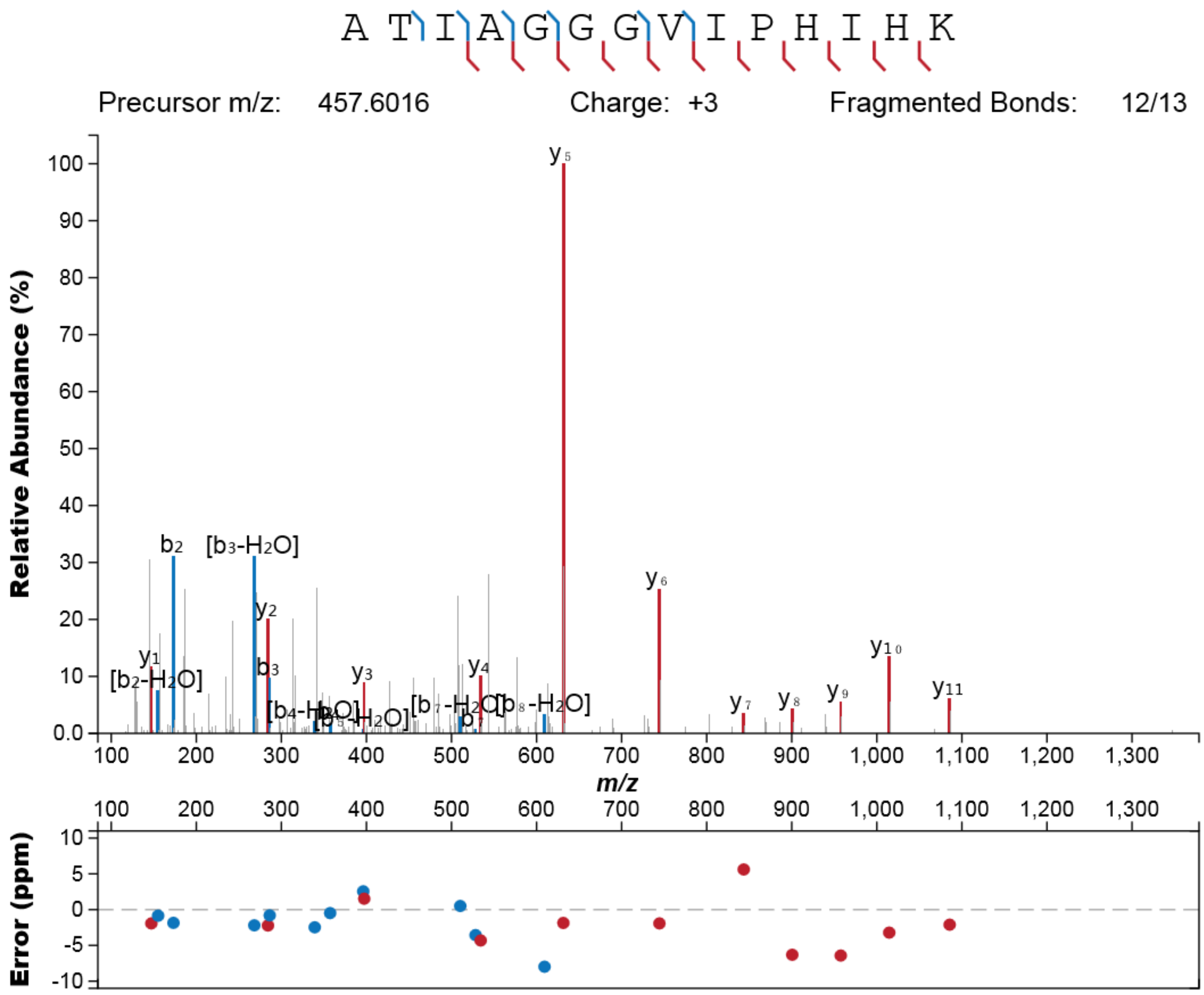

A T I A G G G V I P H I H K
Precursor m/z: 457.6016
Charge: +3
Fragmented Bonds: 12/13
Relative Abundance (%)
m/z
Error (ppm)
y5
y6
b2
[b3-H2O]
y2
b3
y1
[b2-H2O]
y3
y4
y7
y8
y9
y10
y11
[b8-H2O]

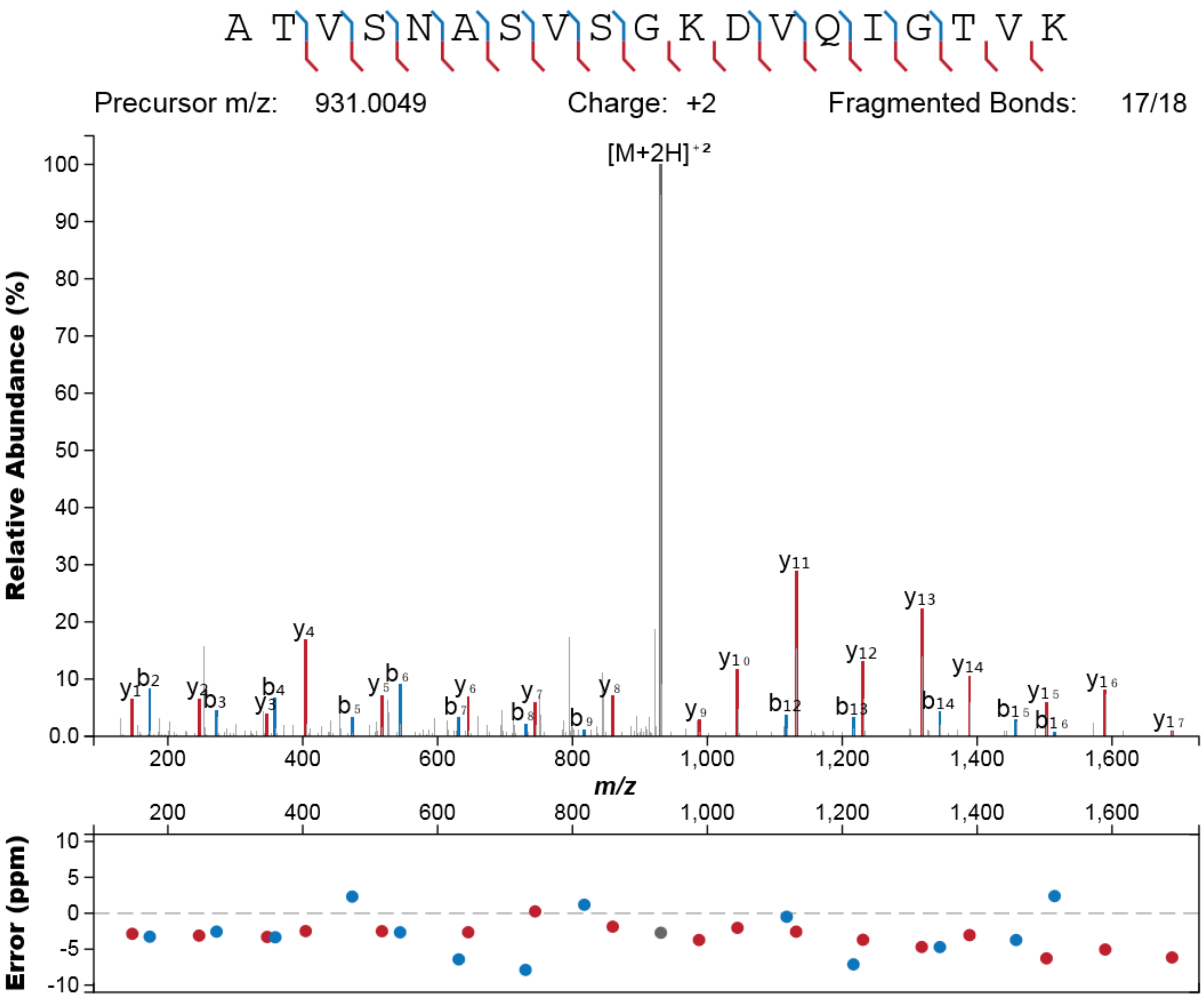
A T V S N A S V S G K D V Q I G T V K
Precursor m/z: 931.0049
Charge: +2
Fragmented Bonds: 17/18
[M+2H]+2
Relative Abundance (%)
m/z
Error (ppm)

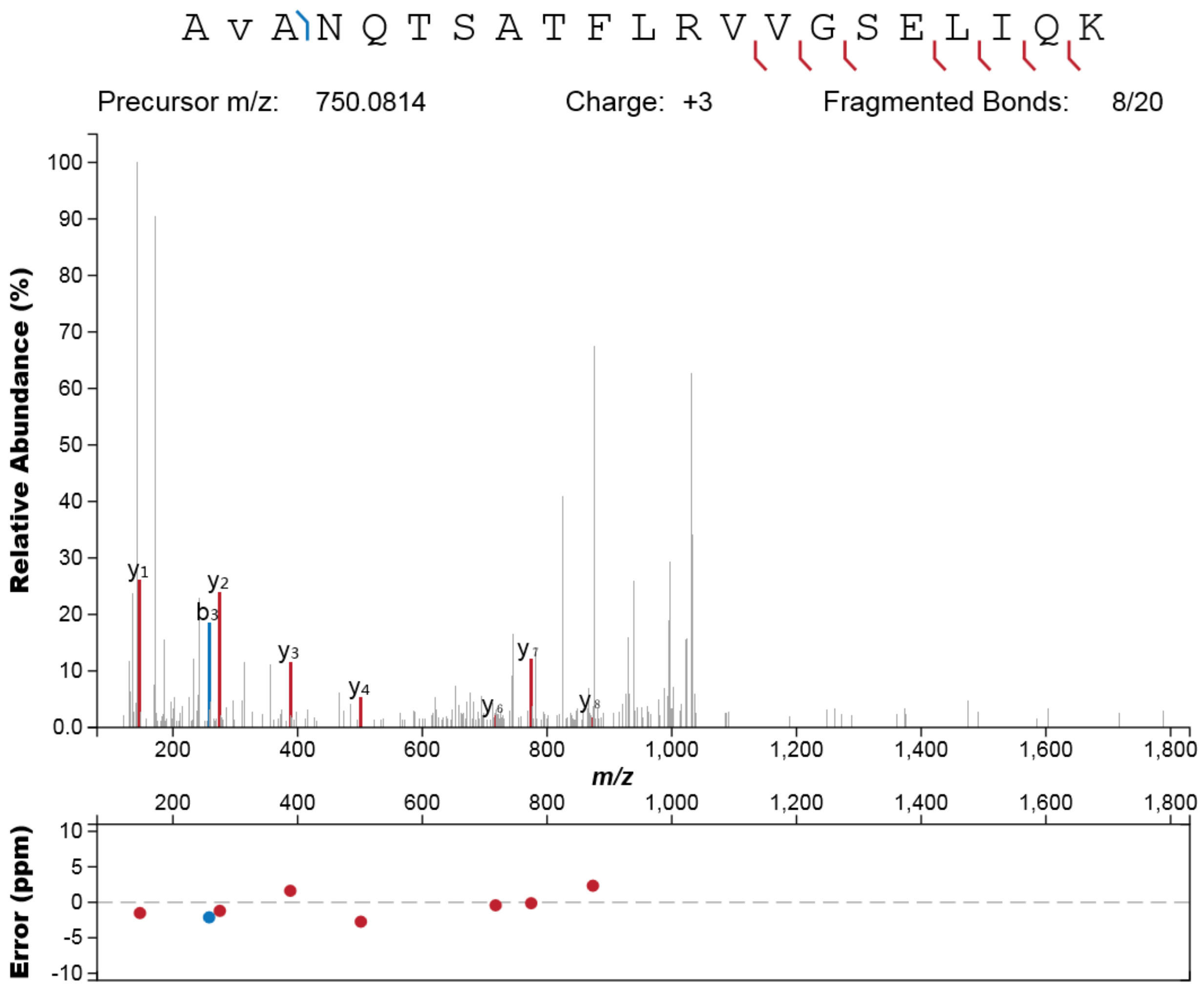

A v A N Q T S A T F L R V V G S E L I Q K
Precursor m/z: 750.0814
Charge: +3
Fragmented Bonds: 8/20
Relative Abundance (%)
m/z
Error (ppm)
y1
y2
b3
y3
y4
y6
y7
y8

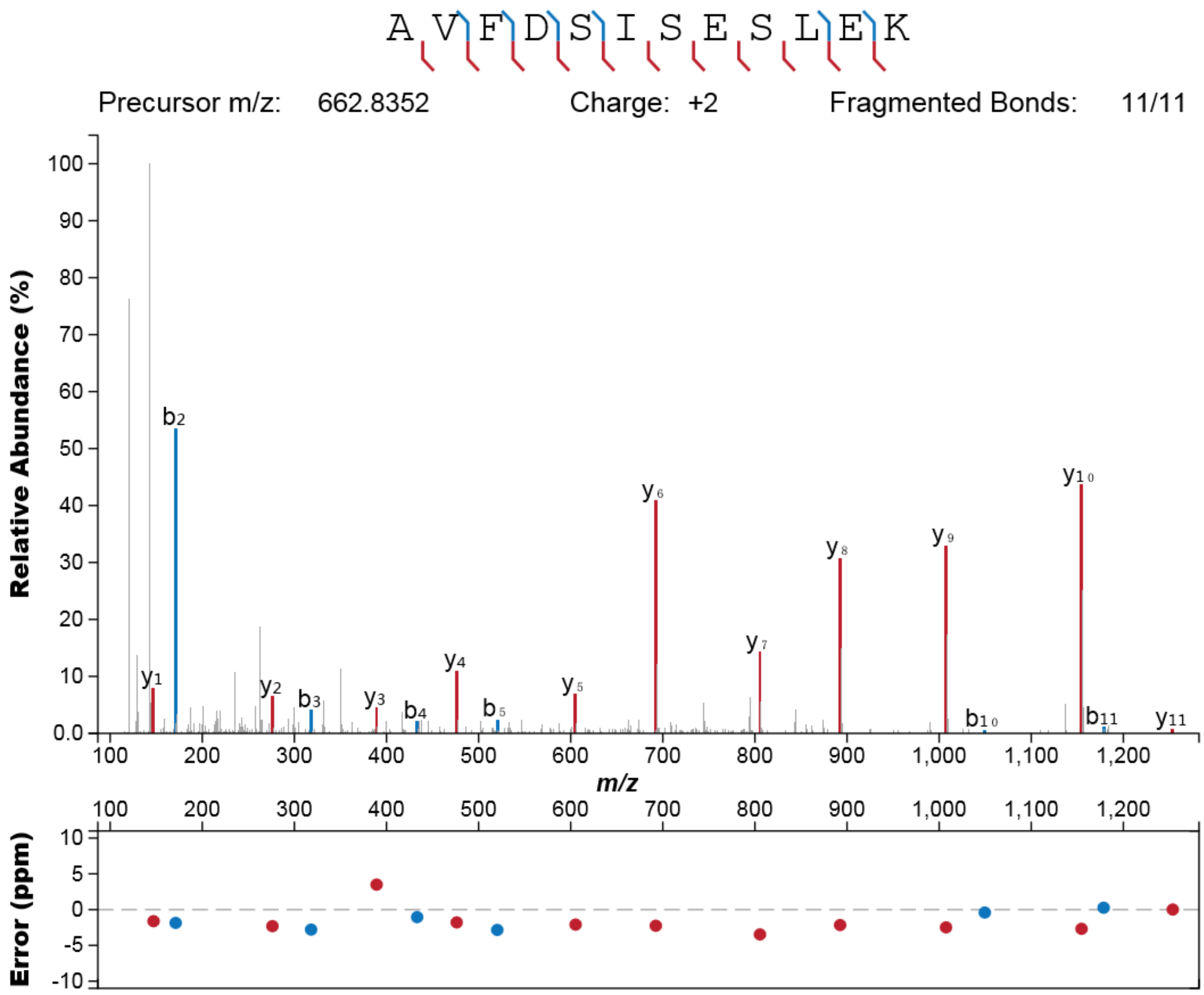

A V F D S I S E S L E K
Precursor m/z: 662.8352
Charge: +2
Fragmented Bonds: 11/11
Relative Abundance (%)
m/z
Error (ppm)
b2
y1
y2
b3
y3
b4
y4
b5
y5
y6
y7
y8
y9
b10
y10
b11
y11

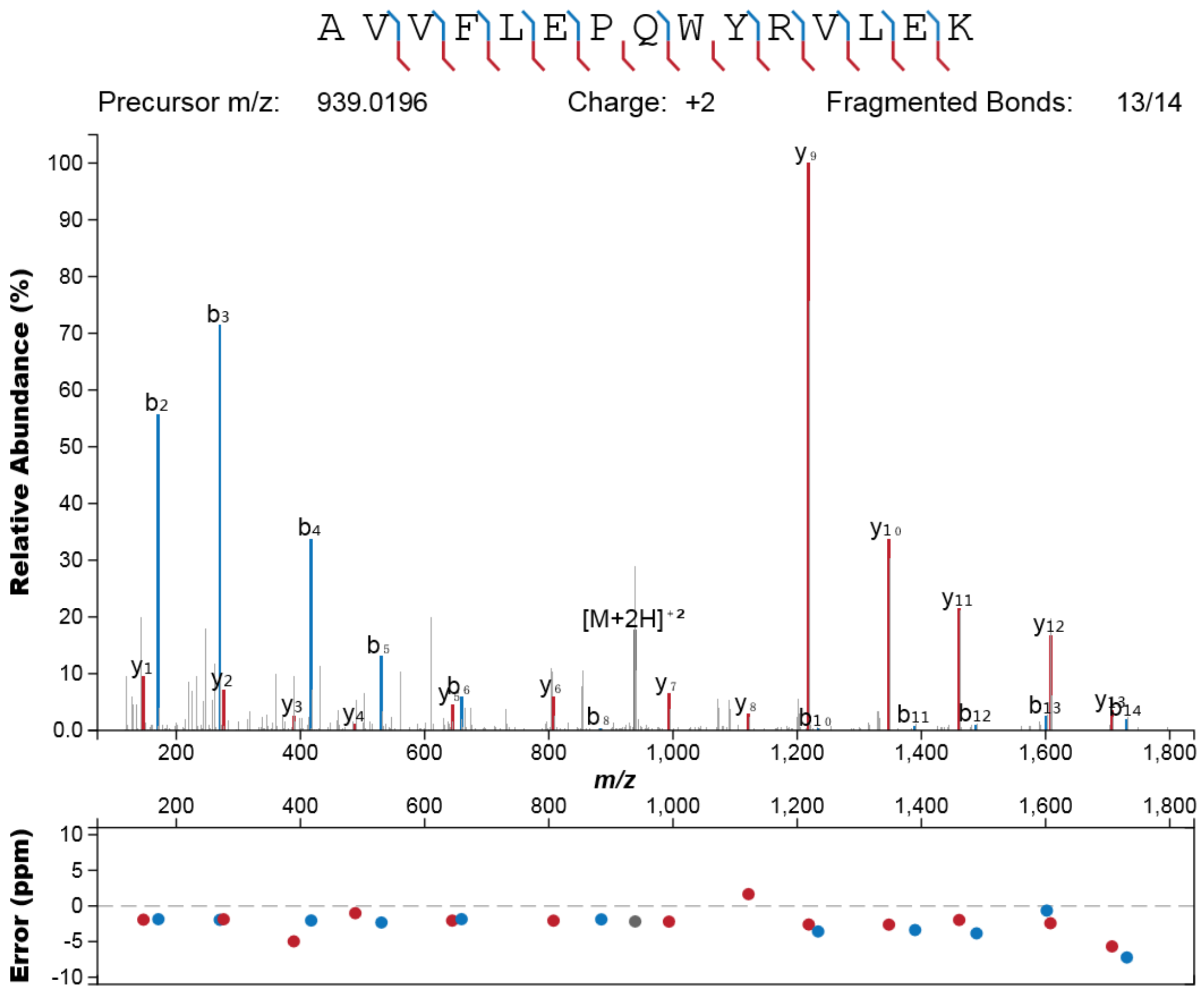
A V V F L E P Q W Y R V L E K
Precursor m/z: 939.0196
Charge: +2
Fragmented Bonds: 13/14
Relative Abundance (%)
m/z
[M+2H]+2
Error (ppm)

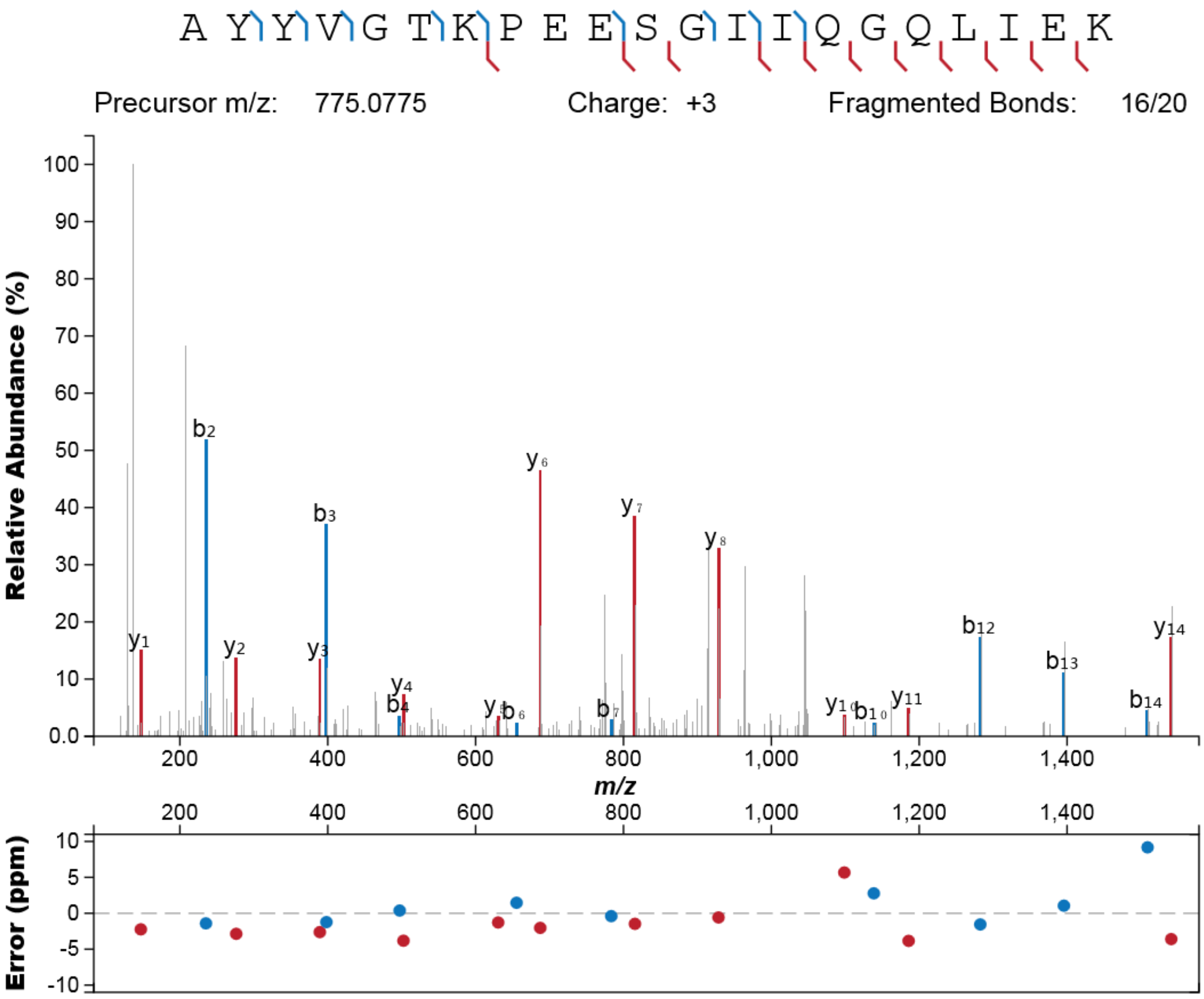

A Y Y V G T K P E E S G I I Q G Q L I E K
Precursor m/z: 775.0775
Charge: +3
Fragmented Bonds: 16/20
Relative Abundance (%)
m/z
Error (ppm)
b2
b3
b4
b6
b7
b10
b12
b13
b14
y1
y2
y3
y4
y5
y6
y7
y8
y10
y11
y14

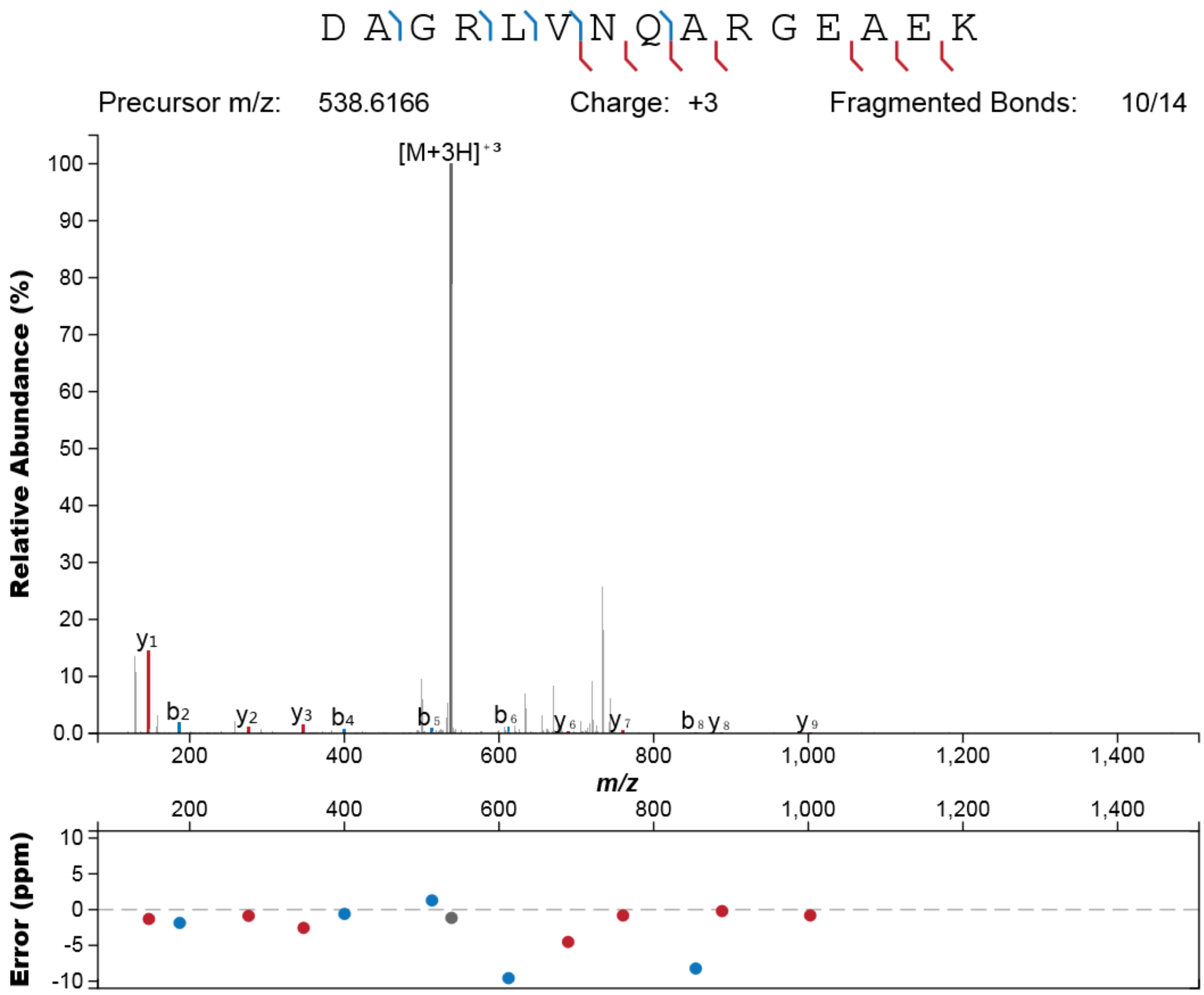
D A G R L V N Q A R G E A E K
Precursor m/z: 538.6166
Charge: +3
Fragmented Bonds: 10/14
[M+3H]+3
Relative Abundance (%)
m/z
Error (ppm)
y1
b2
y2
y3
b4
b5
b6
y6
y7
b8
y8
y9

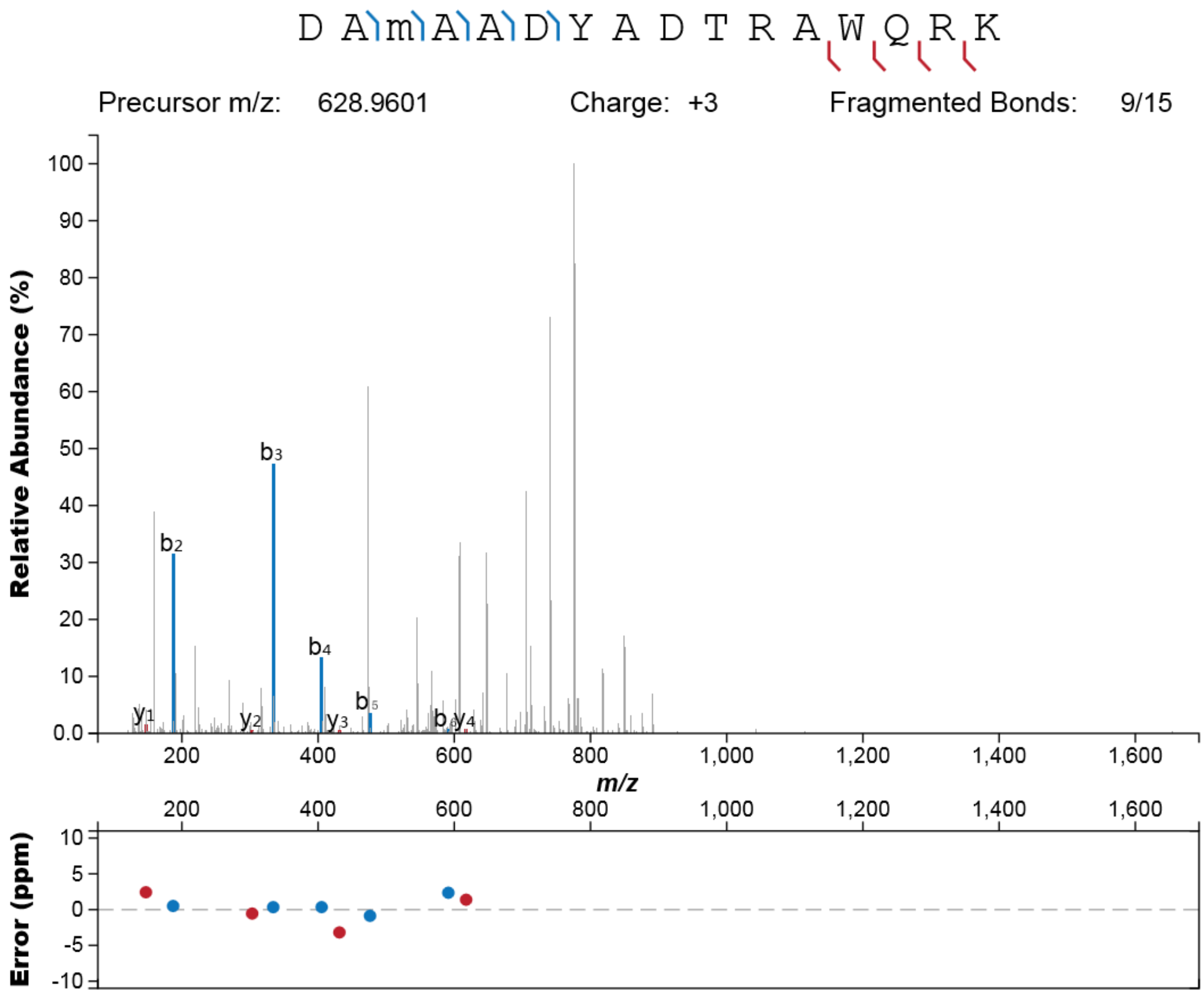
D A m A A D Y A D T R A W Q R K
Precursor m/z: 628.9601
Charge: +3
Fragmented Bonds: 9/15
Relative Abundance (%)
b2
b3
b4
b5
b6
y1
y2
y3
y4
m/z
Error (ppm)

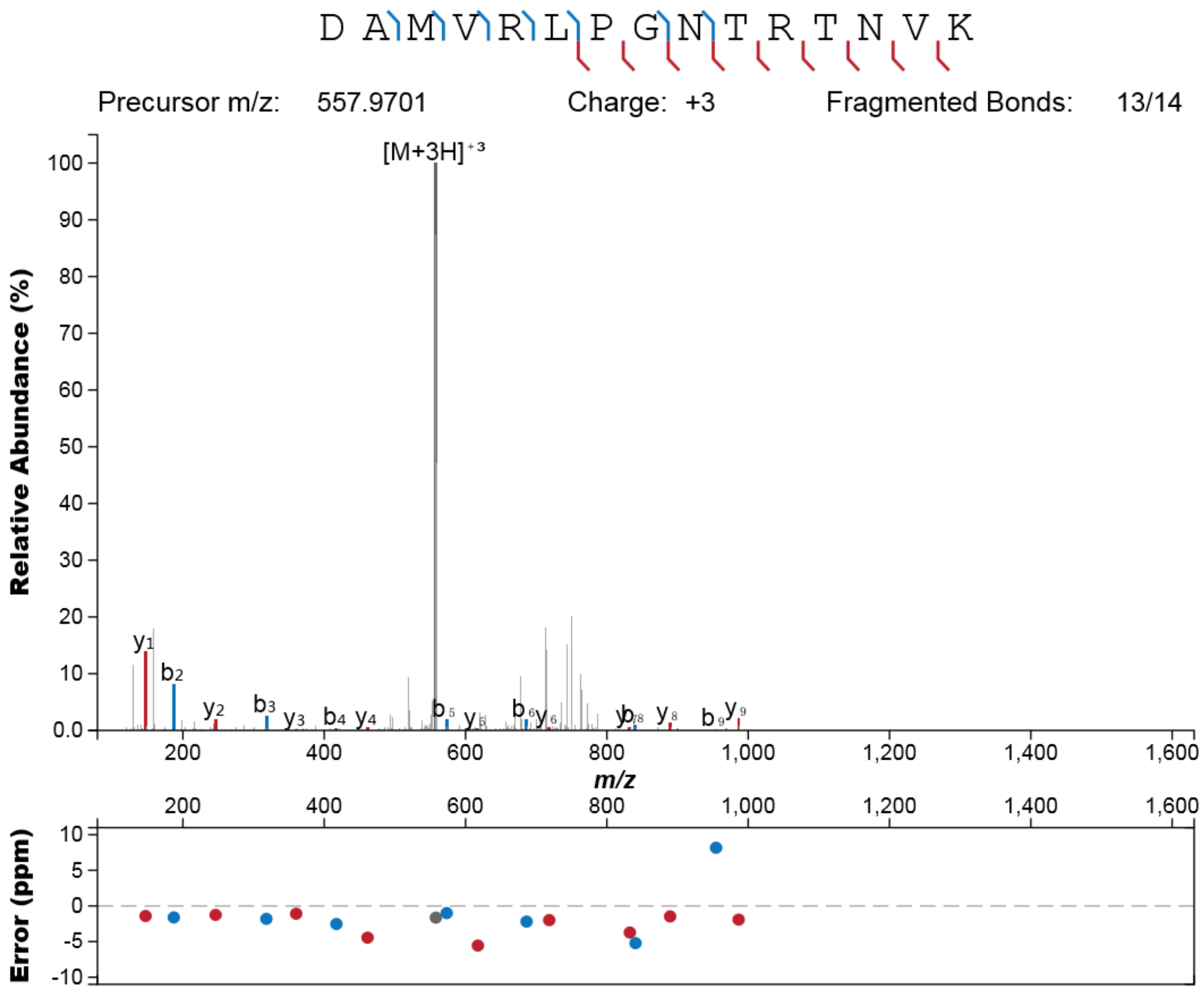
D A M V R L P G N T R T N V K
Precursor m/z: 557.9701
Charge: +3
Fragmented Bonds: 13/14
[M+3H]+3
Relative Abundance (%)
m/z
Error (ppm)

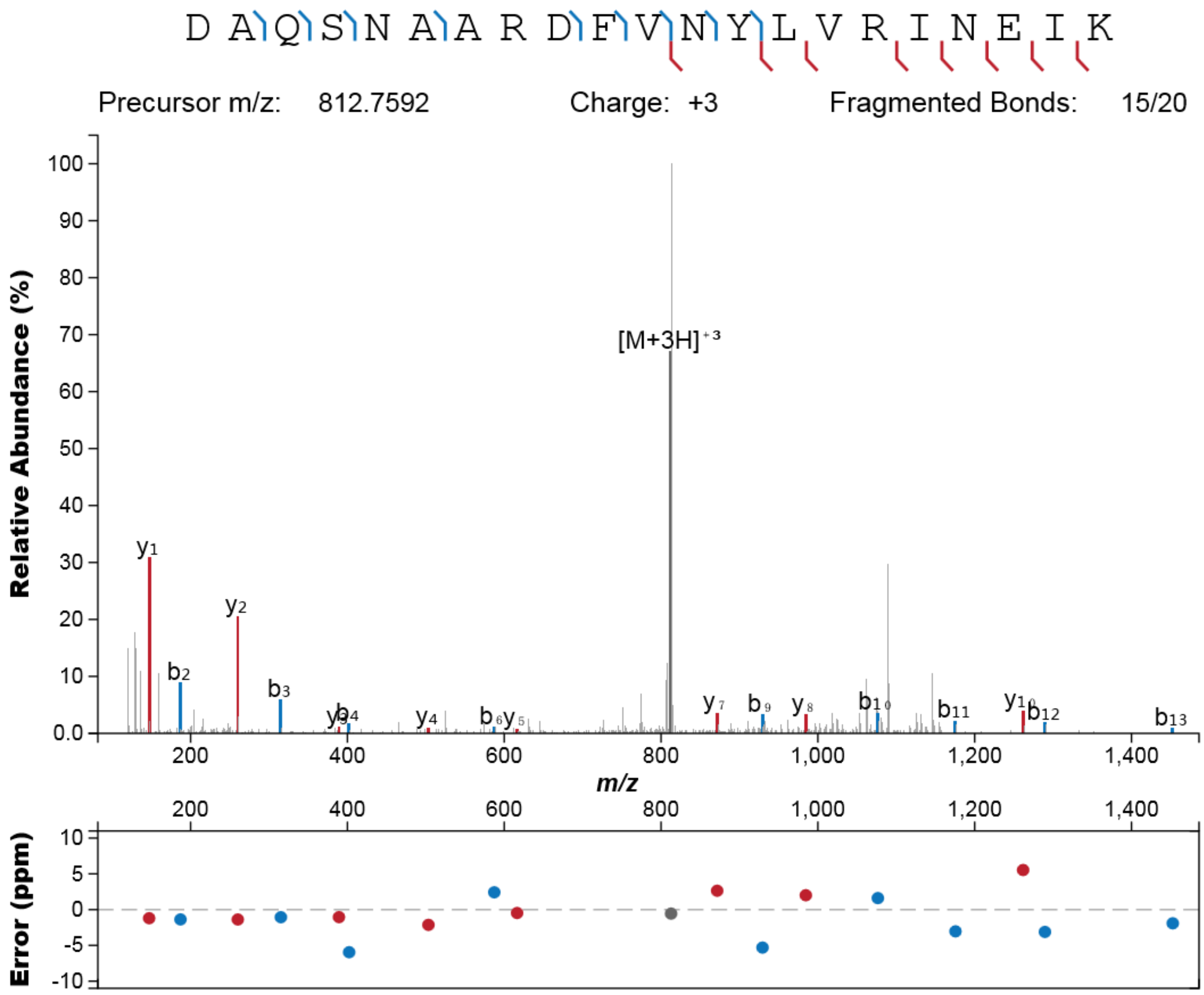
D A Q S N A A R D F V N Y L V R I N E I K
Precursor m/z: 812.7592
Charge: +3
Fragmented Bonds: 15/20
Relative Abundance (%)
[M+3H]+3
m/z
Error (ppm)

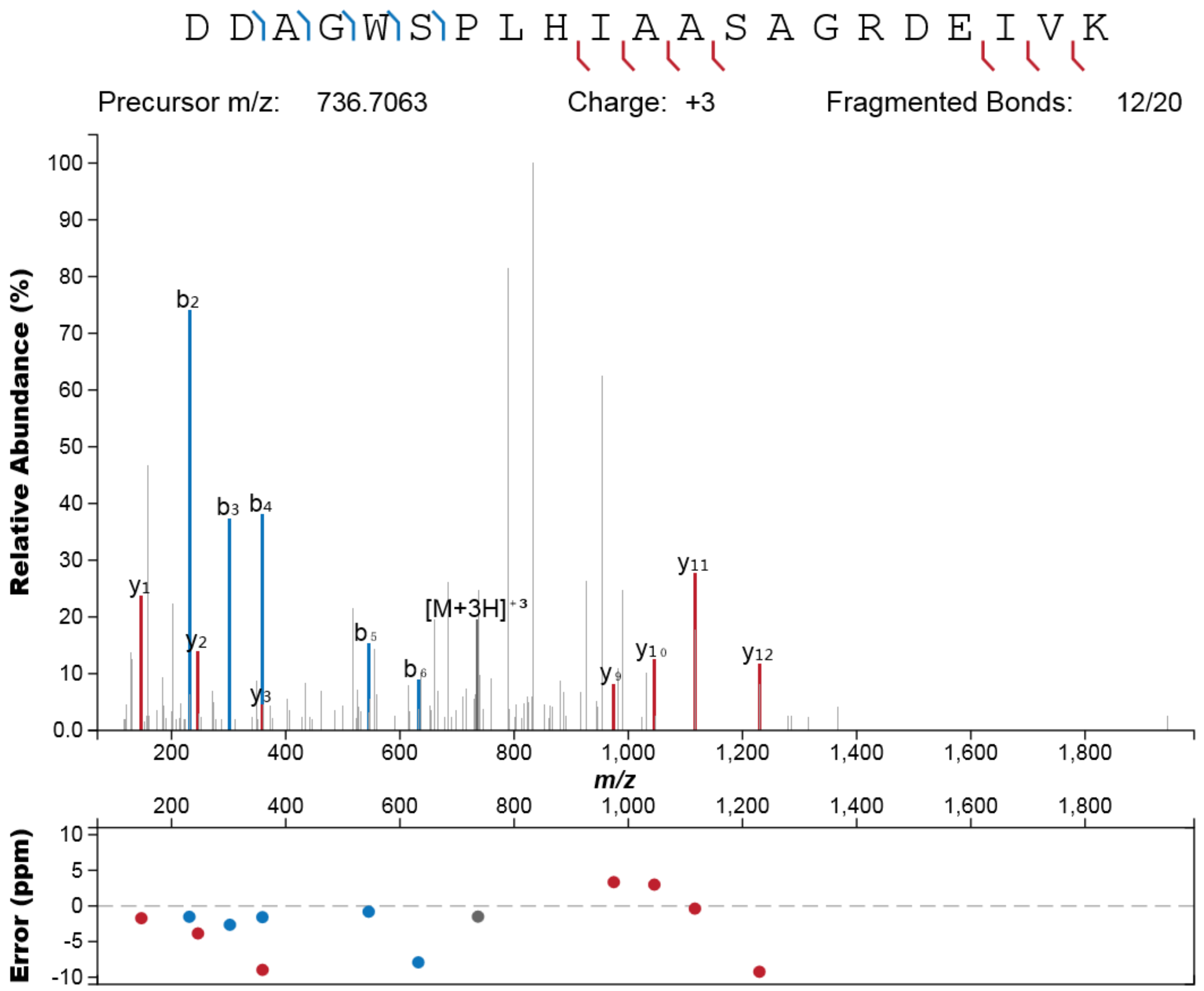
D D A G W S P L H I A A S A G R D E I V K
Precursor m/z: 736.7063
Charge: +3
Fragmented Bonds: 12/20
Relative Abundance (%)
m/z
Error (ppm)
b2
b3
b4
b5
b6
y1
y2
y3
y9
y10
y11
y12
[M+3H]+3

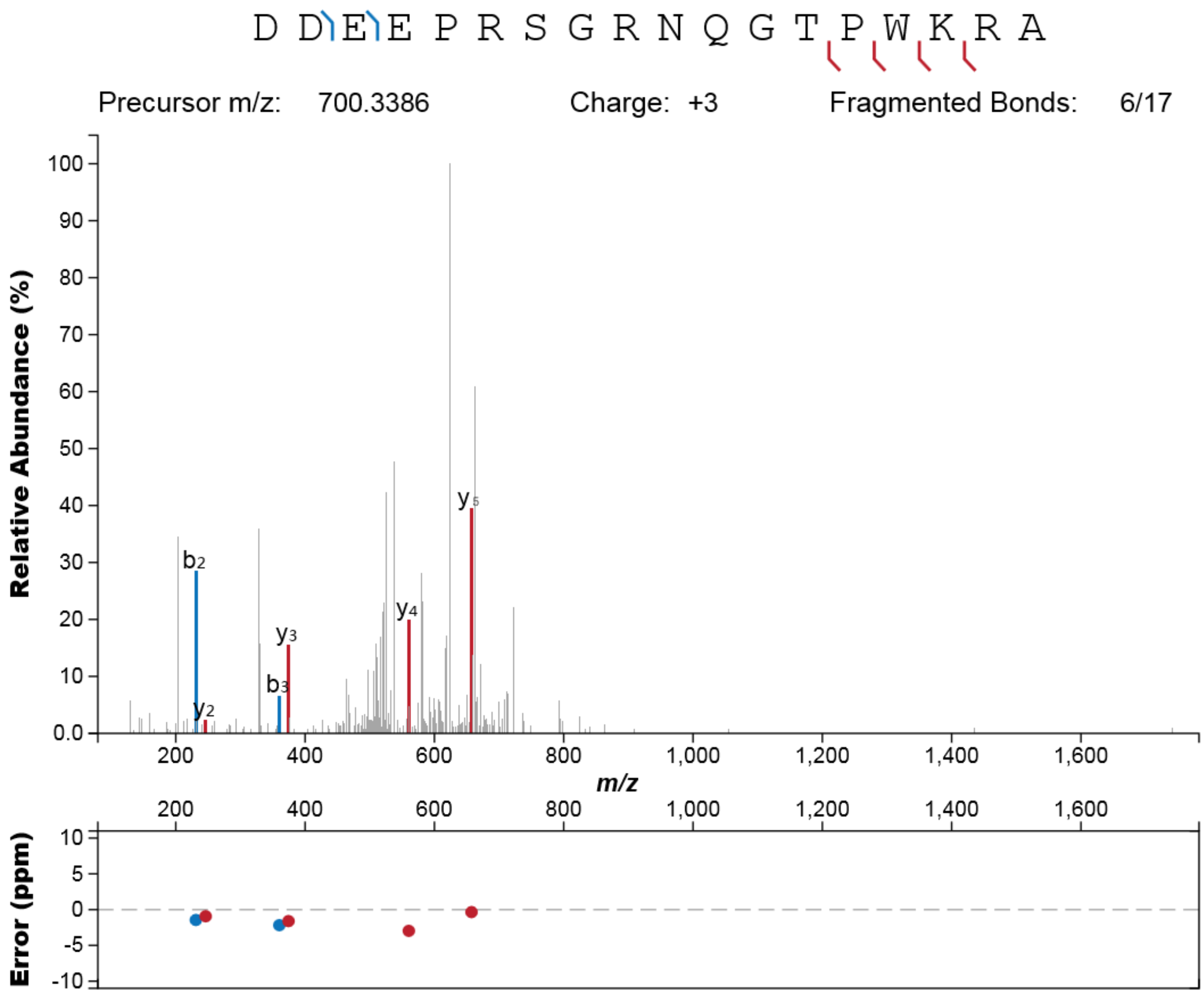

D D E E P R S G R N Q G T P W K R A
Precursor m/z: 700.3386
Charge: +3
Fragmented Bonds: 6/17
Relative Abundance (%)
m/z
Error (ppm)
b2
b3
y2
y3
y4
y5

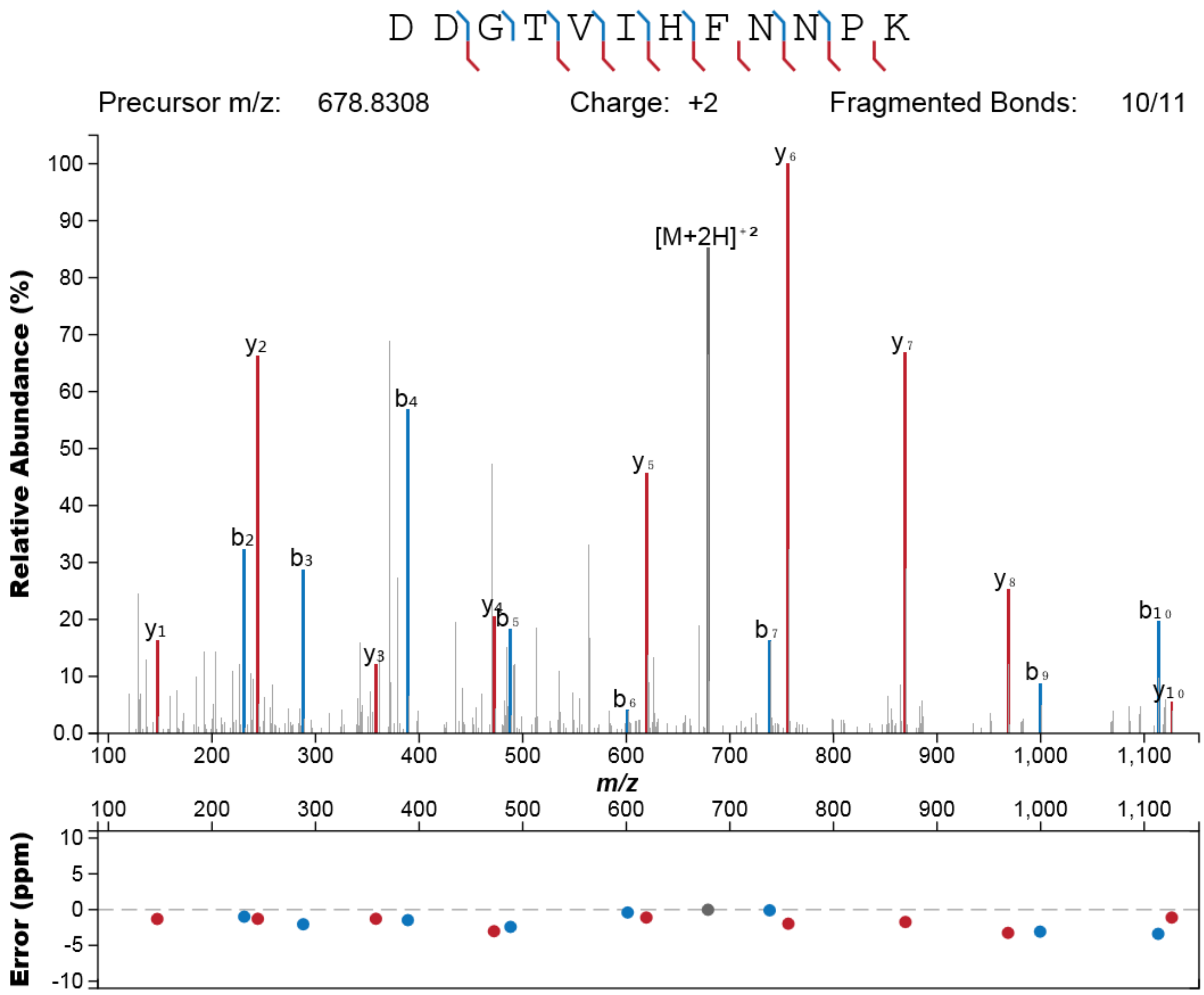
D D G T V I H F N N P K
Precursor m/z: 678.8308
Charge: +2
Fragmented Bonds: 10/11
Relative Abundance (%)
[M+2H]+2
y1
y2
y3
y4
y5
y6
y7
y8
y10
b2
b3
b4
b5
b6
b7
b9
b10
m/z
Error (ppm)

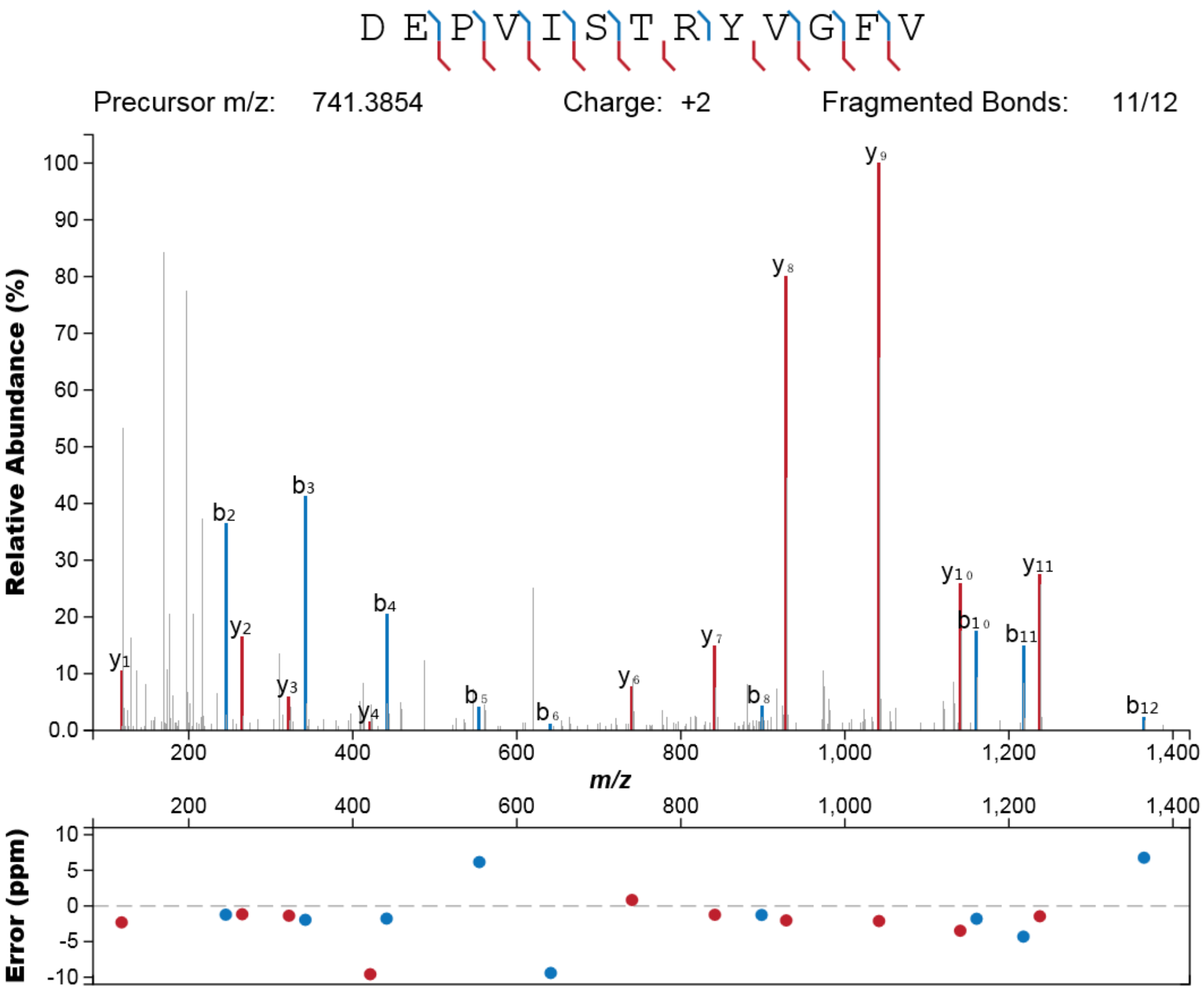
D E P V I S T R Y V G F V
Precursor m/z: 741.3854
Charge: +2
Fragmented Bonds: 11/12
Relative Abundance (%)
m/z
Error (ppm)
y1 y2 y3 y4 y6 y7 y8 y9 y10 y11
b2 b3 b4 b5 b6 b8 b10 b11 b12

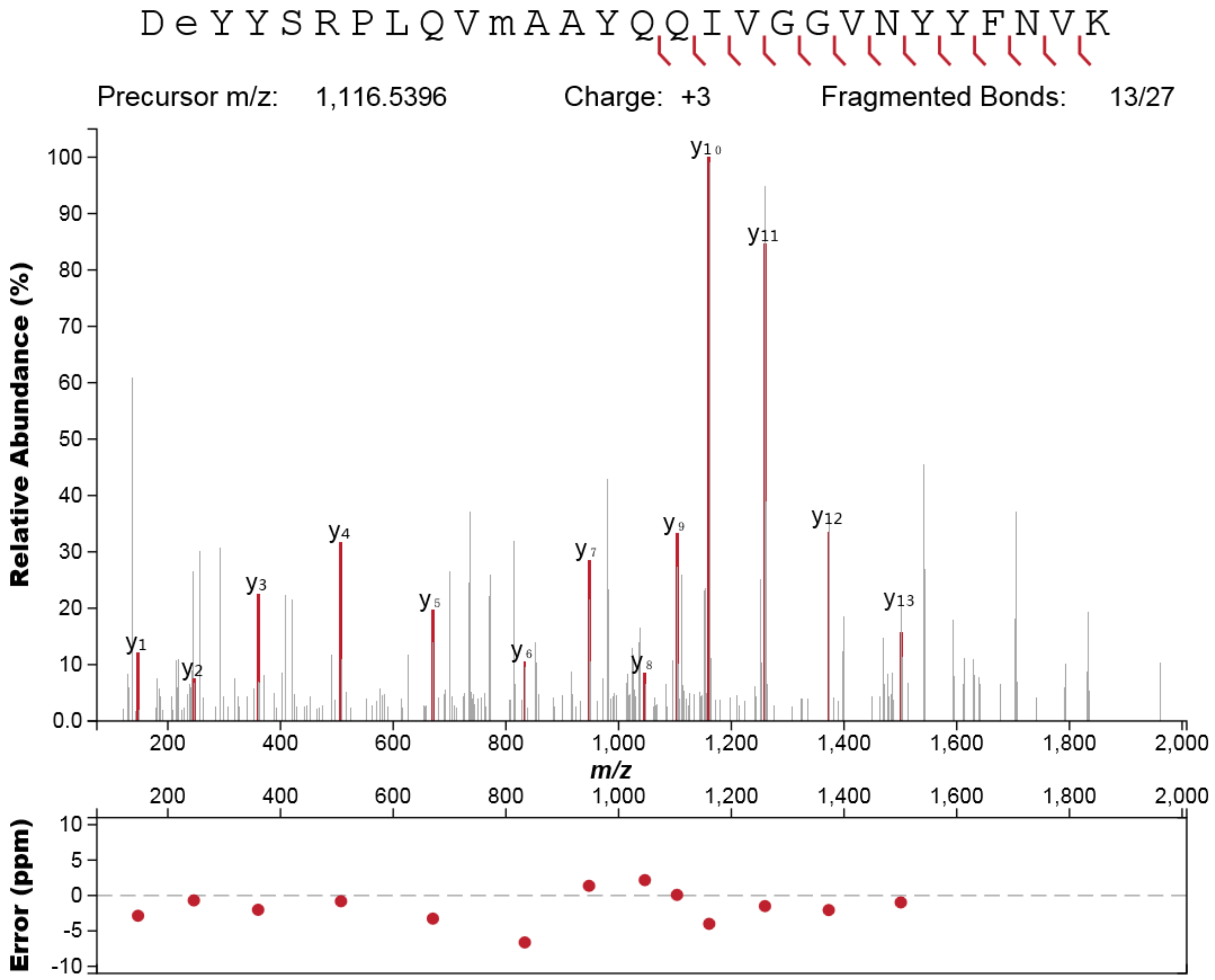

D e Y Y S R P L Q V m A A Y Q Q I V G G V N Y Y F N V K
Precursor m/z: 1,116.5396
Charge: +3
Fragmented Bonds: 13/27
Relative Abundance (%)
m/z
Error (ppm)
y1
y2
y3
y4
y5
y6
y7
y8
y9
y10
y11
y12
y13

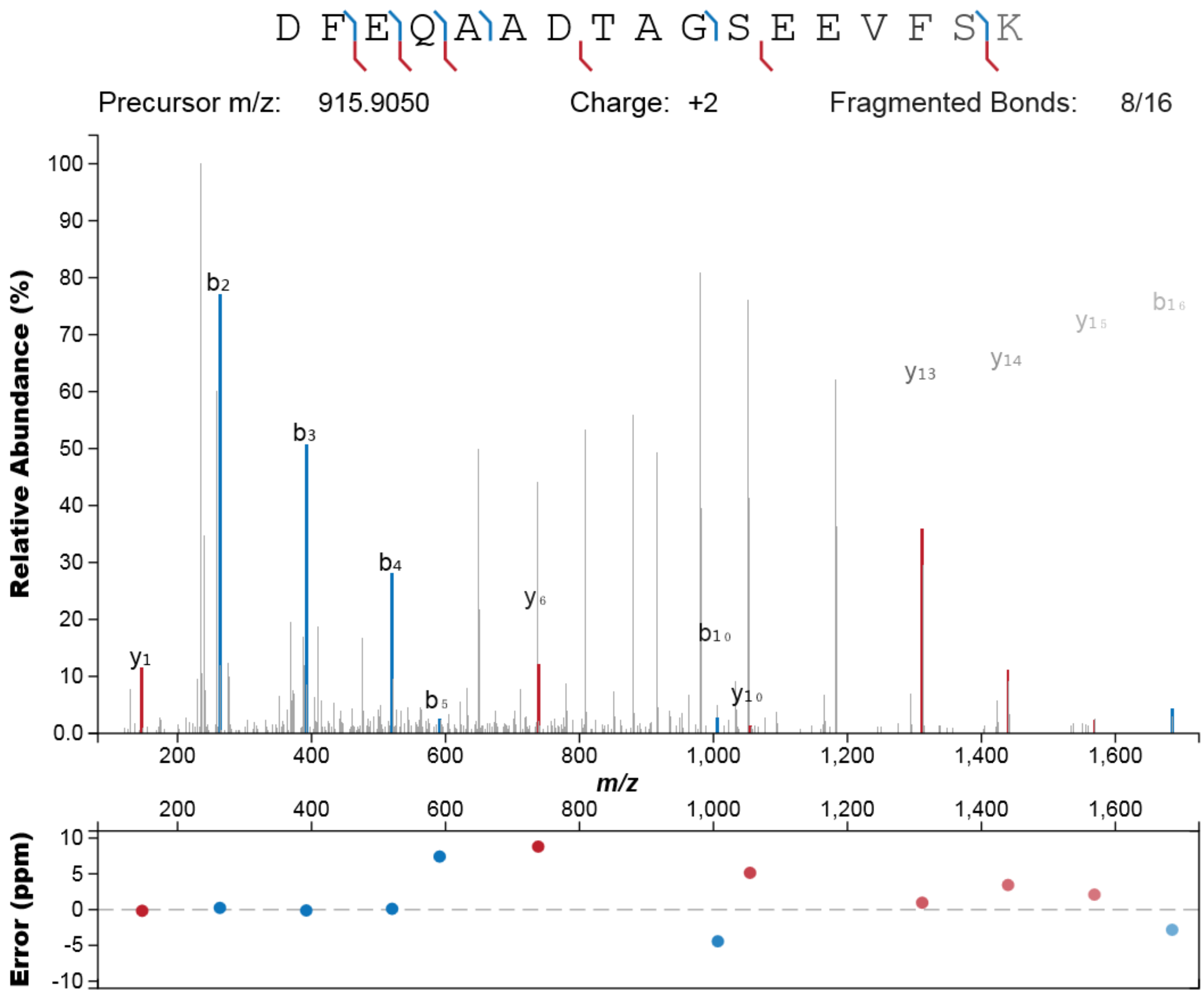

D F E Q A A D T A G S E E V F S K
Precursor m/z: 915.9050
Charge: +2
Fragmented Bonds: 8/16
Relative Abundance (%)
m/z
Error (ppm)
y1
b2
b3
b4
b5
y6
b10
y10
y13
y14
y15
b16

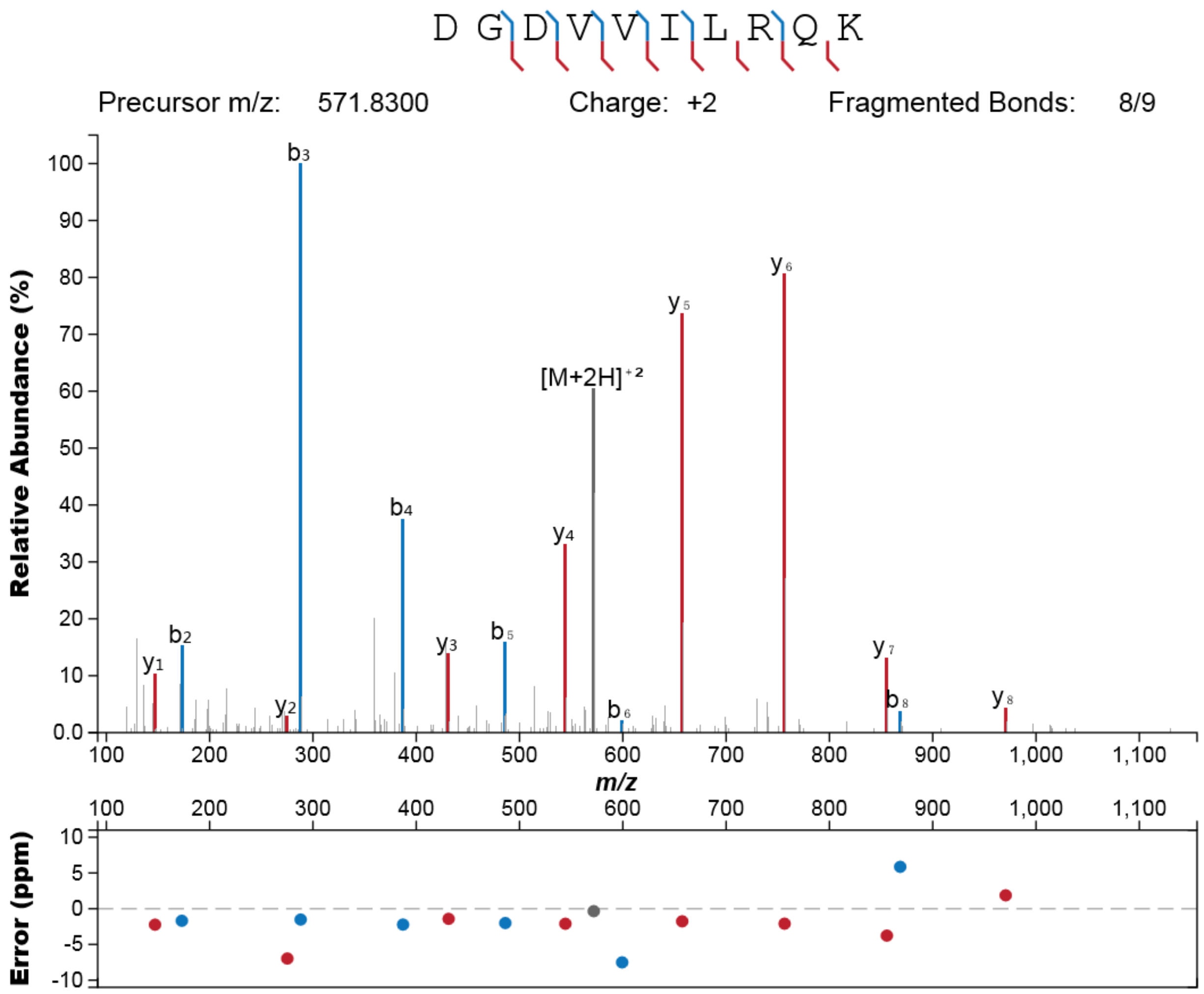

D G D V V I L R Q K
Precursor m/z: 571.8300
Charge: +2
Fragmented Bonds: 8/9
Relative Abundance (%)
b3
b2
y1
y2
b4
y3
b5
y4
[M+2H]+2
b6
y5
y6
y7
b8
y8
m/z
Error (ppm)

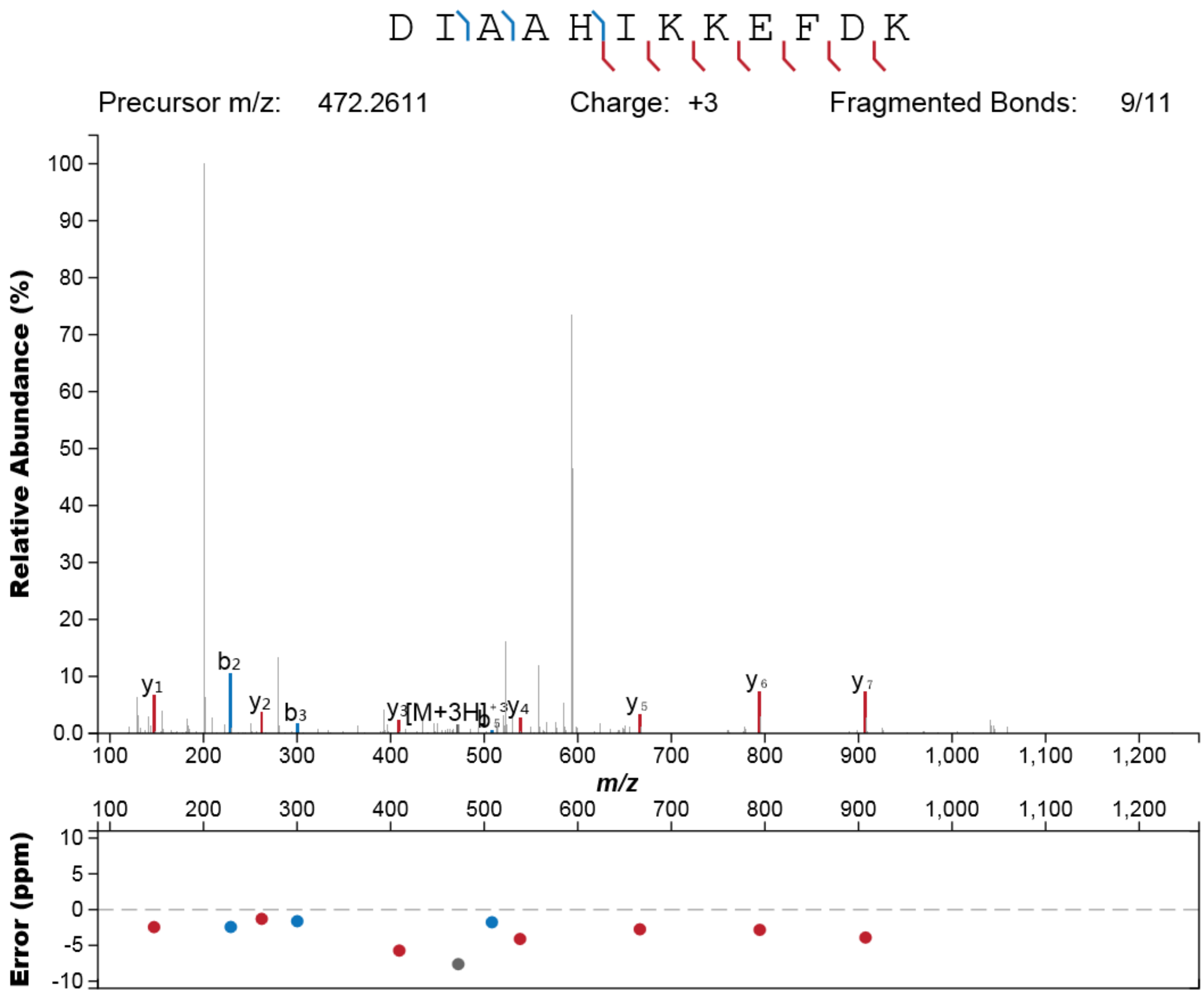
D I A A H I K K E F D K
Precursor m/z: 472.2611
Charge: +3
Fragmented Bonds: 9/11
Relative Abundance (%)
m/z
Error (ppm)
y1
b2
y2
b3
y3
[M+3H]+3
b5
y4
y5
y6
y7

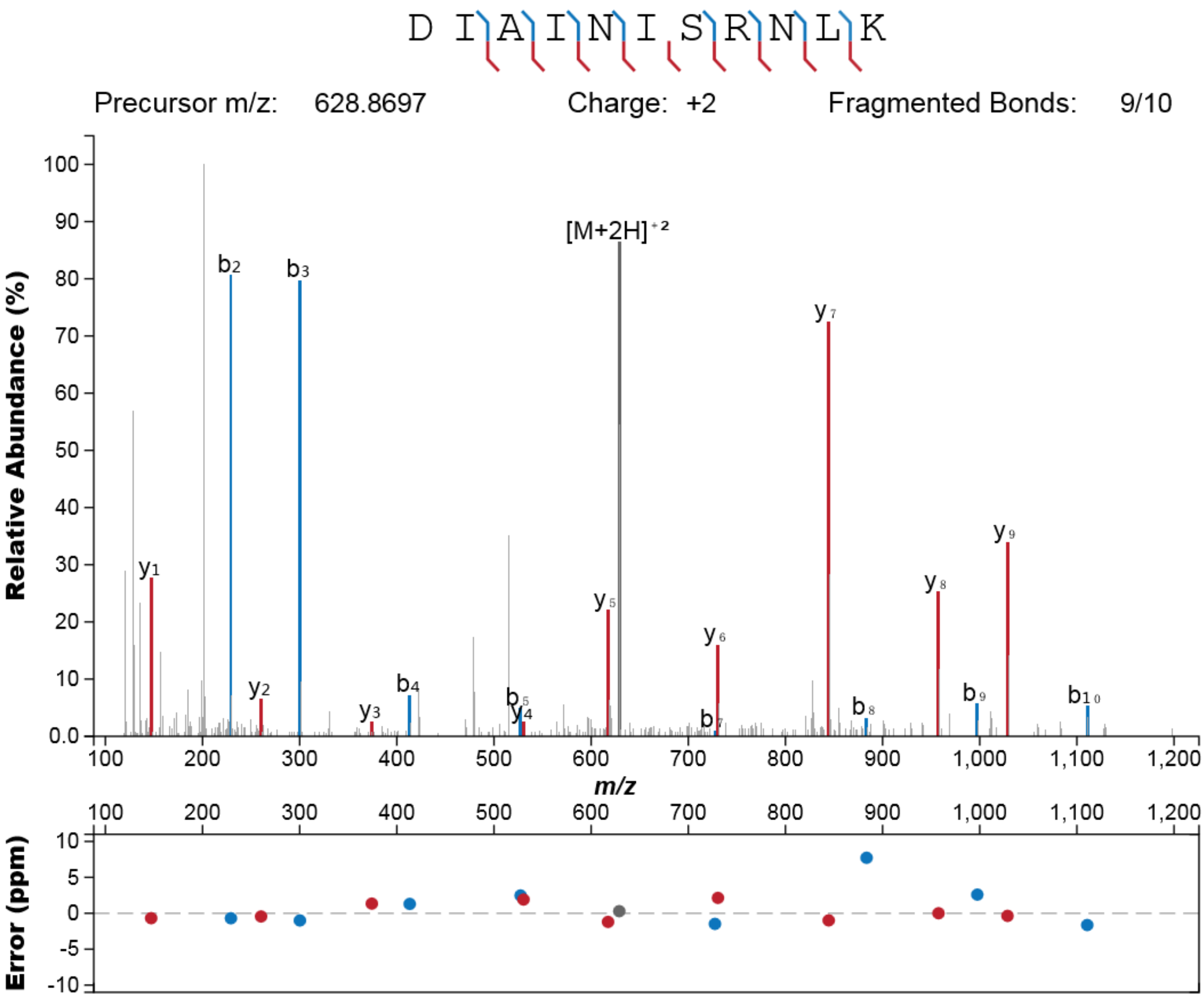
D I A I N I S R N L K
Precursor m/z: 628.8697
Charge: +2
Fragmented Bonds: 9/10
Relative Abundance (%)
[M+2H]+2
b2
b3
y1
y2
y3
b4
b5
y4
y5
b7
y6
y7
b8
y8
b9
y9
b10
m/z
Error (ppm)

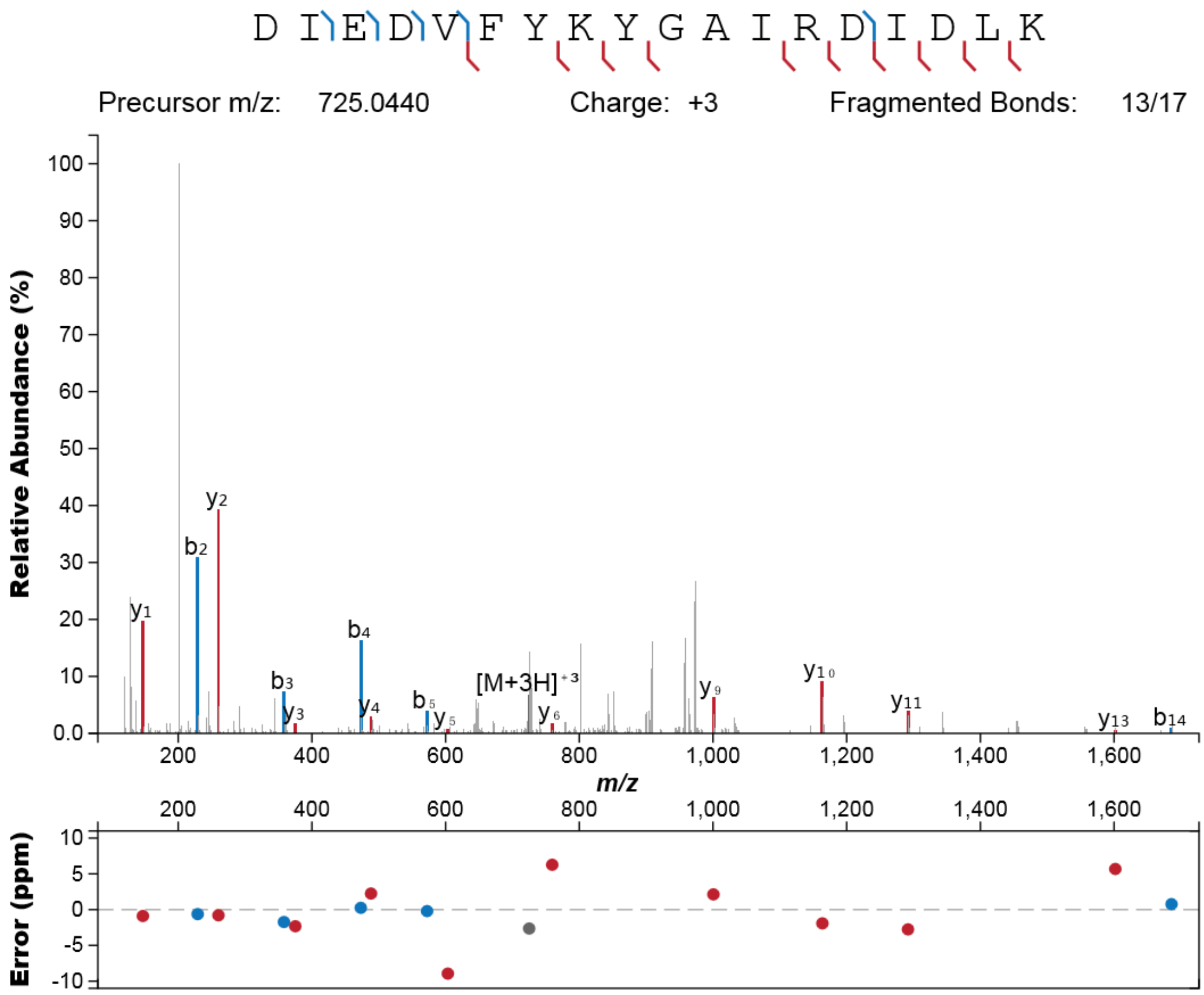
D I E D V F Y K Y G A I R D I D L K
Precursor m/z: 725.0440
Charge: +3
Fragmented Bonds: 13/17
Relative Abundance (%)
m/z
Error (ppm)
y1
b2
y2
b3
y3
b4
y4
b5
y5
[M+3H]+3
y6
y9
y10
y11
y13
b14

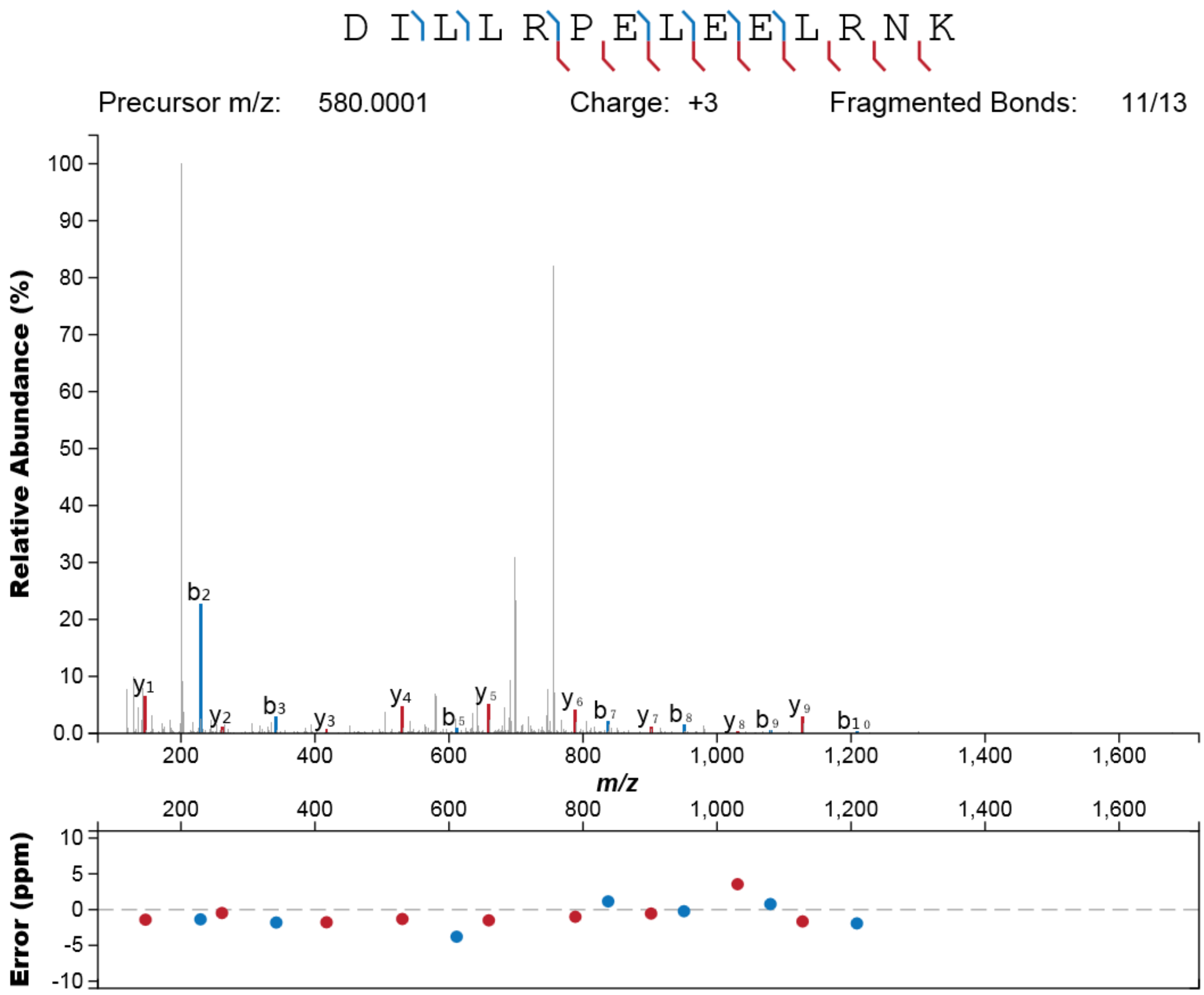
D I L L R P E L E E L R N K
Precursor m/z: 580.0001
Charge: +3
Fragmented Bonds: 11/13
Relative Abundance (%)
m/z
Error (ppm)
y1 b2 y2 b3 y3 y4 b5 y5 y6 b7 y7 b8 y8 b9 y9 b10

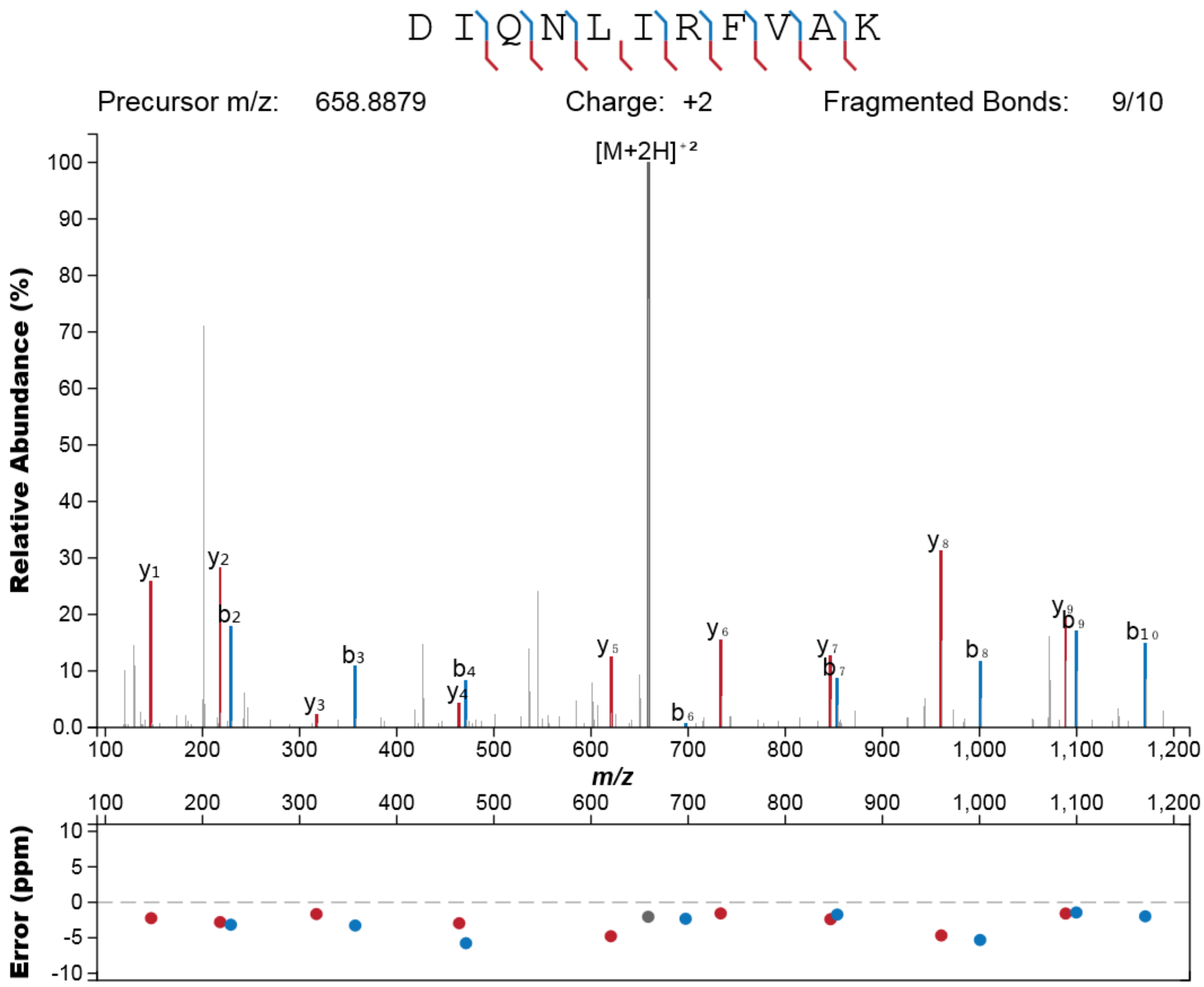

D I Q N L I R F V A K
Precursor m/z: 658.8879
Charge: +2
Fragmented Bonds: 9/10
[M+2H]+2
Relative Abundance (%)
m/z
Error (ppm)
y1
y2
b2
b3
y3
b4
y4
y5
b6
y6
y7
b7
y8
b8
y9
b9
b10

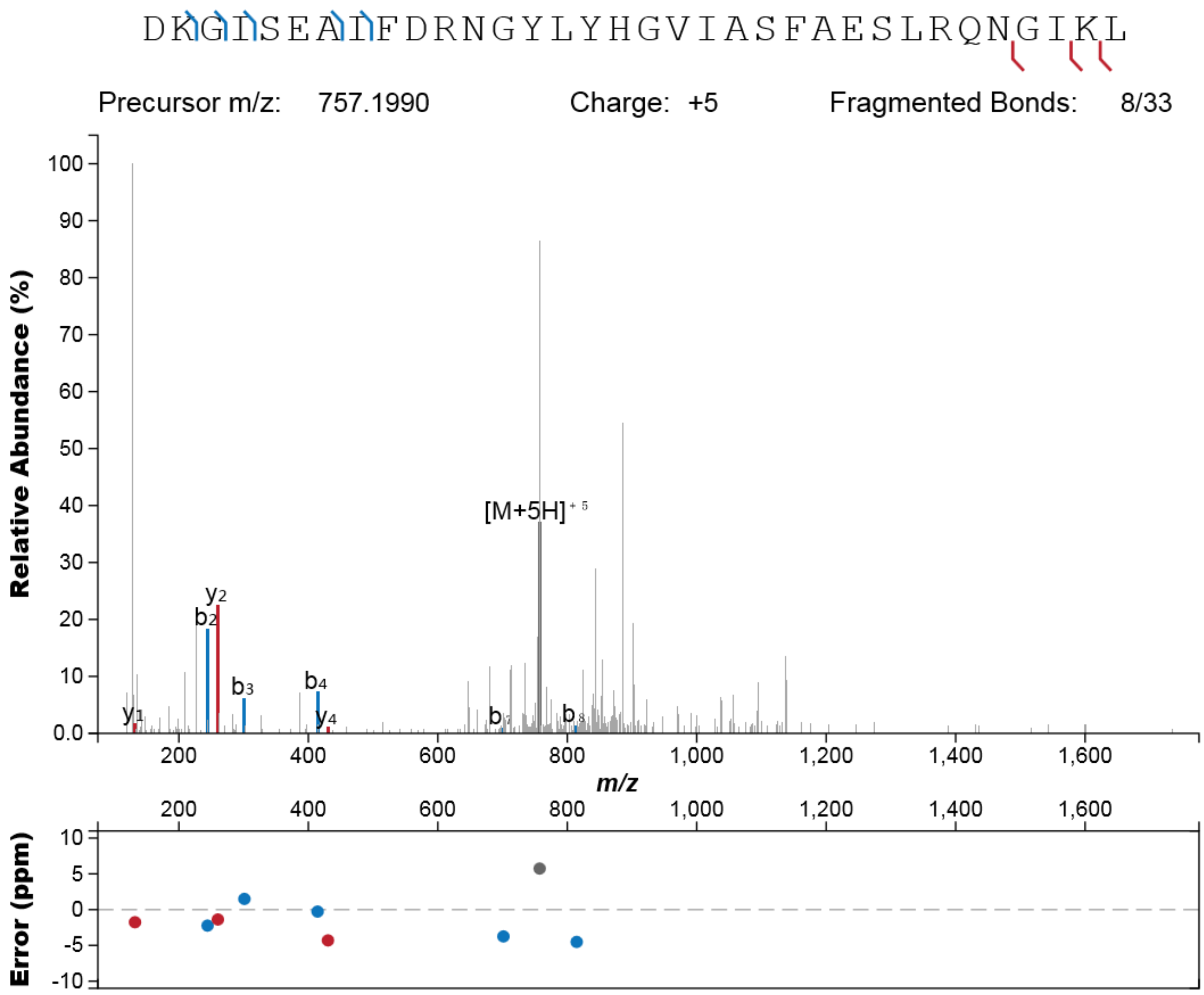
DKGISEAIFDRNGYLYHGVIASFAESLRQNGIKL
Precursor m/z: 757.1990
Charge: +5
Fragmented Bonds: 8/33
Relative Abundance (%)
[M+5H]+5
y1
b2
y2
b3
b4
y4
b7
b8
m/z
Error (ppm)

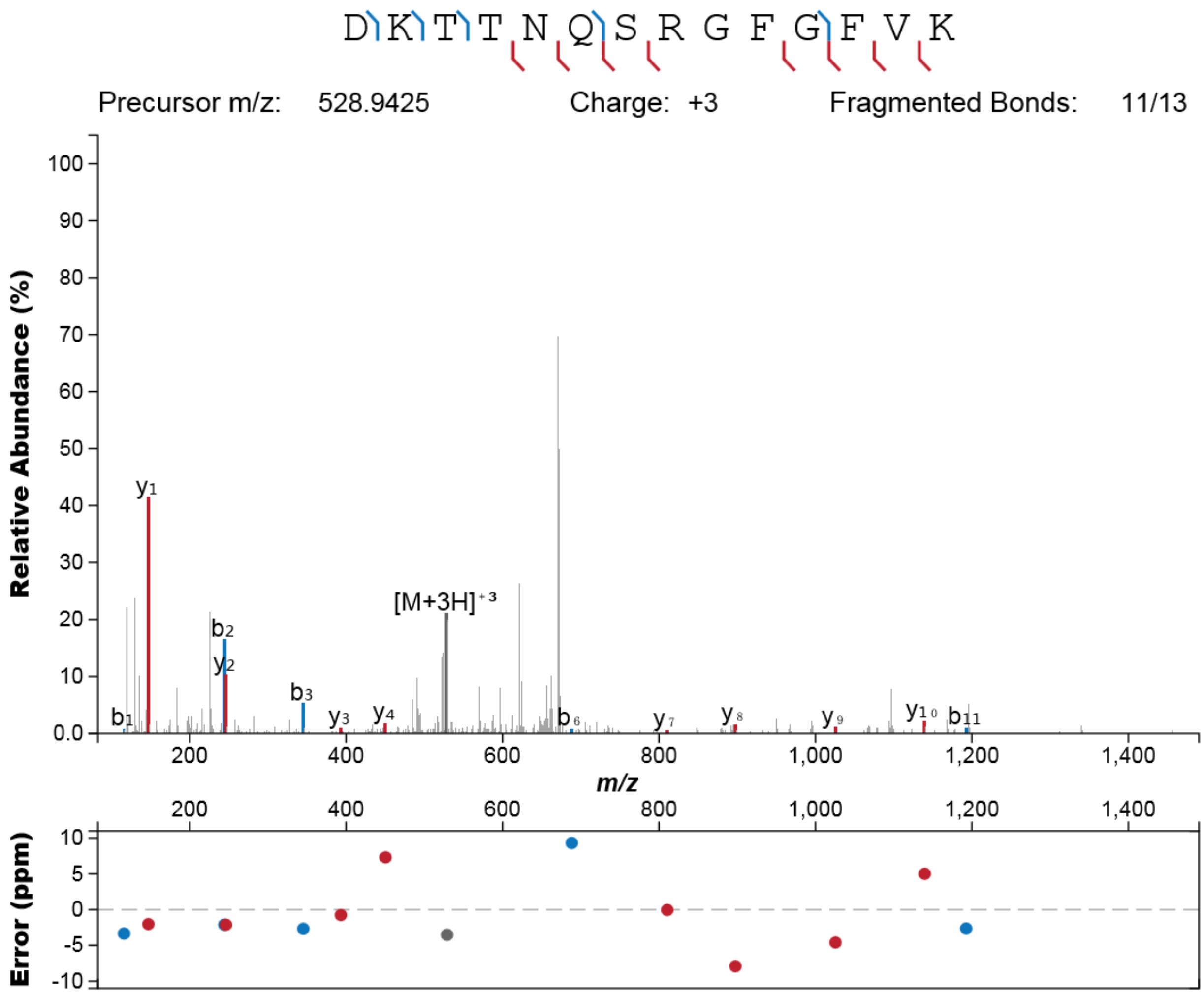
D K T T N Q S R G F G F V K
Precursor m/z: 528.9425
Charge: +3
Fragmented Bonds: 11/13
Relative Abundance (%)
m/z
Error (ppm)
[M+3H]+3
y1
b2
y2
b3
y3
y4
b1
b6
y7
y8
y9
y10
b11

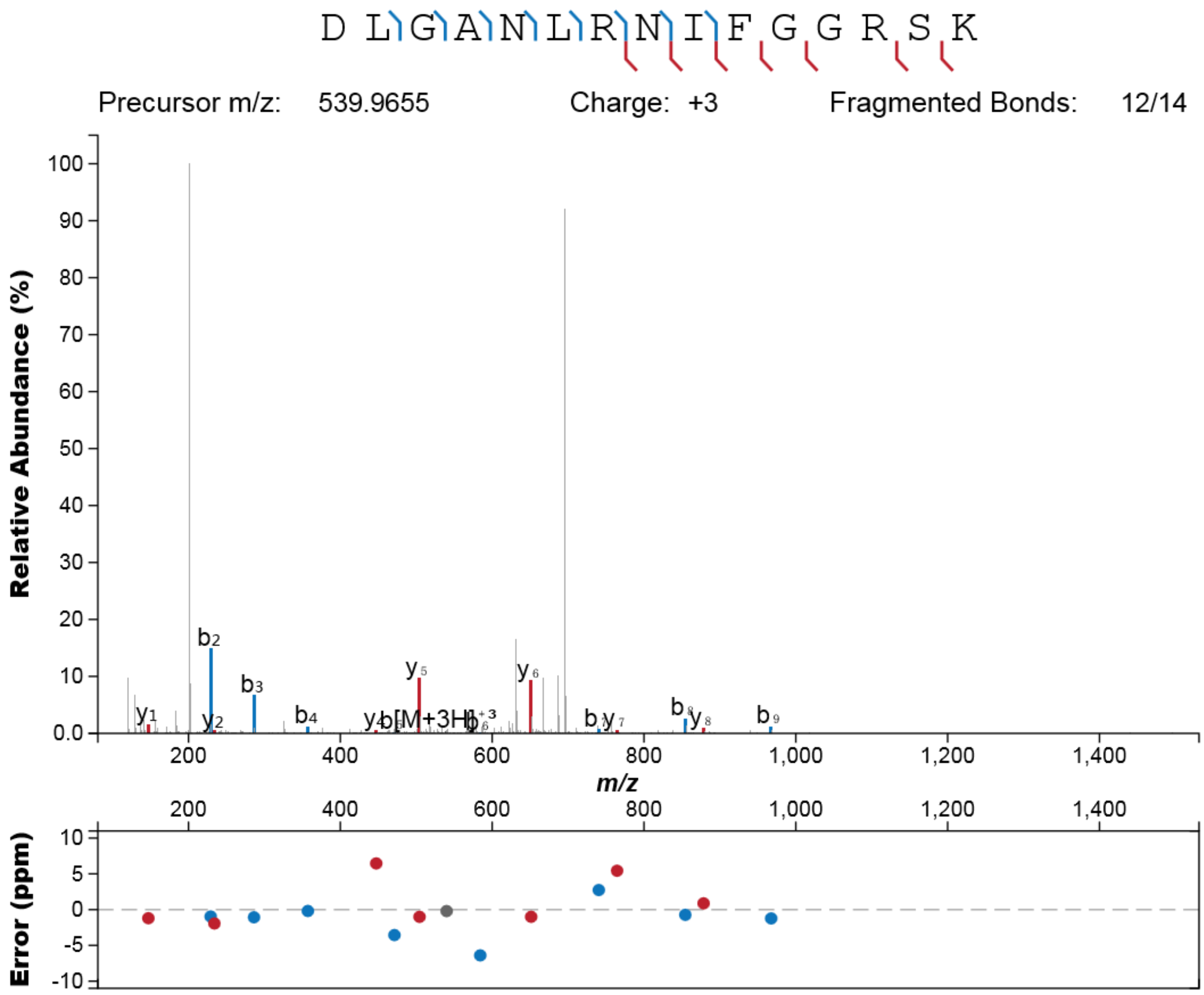
D L G A N L R N I F G G R S K
Precursor m/z: 539.9655
Charge: +3
Fragmented Bonds: 12/14
Relative Abundance (%)
m/z
Error (ppm)
b2
b3
b4
b9
y1
y2
y5
y6
y7
y8
[M+3H]+3

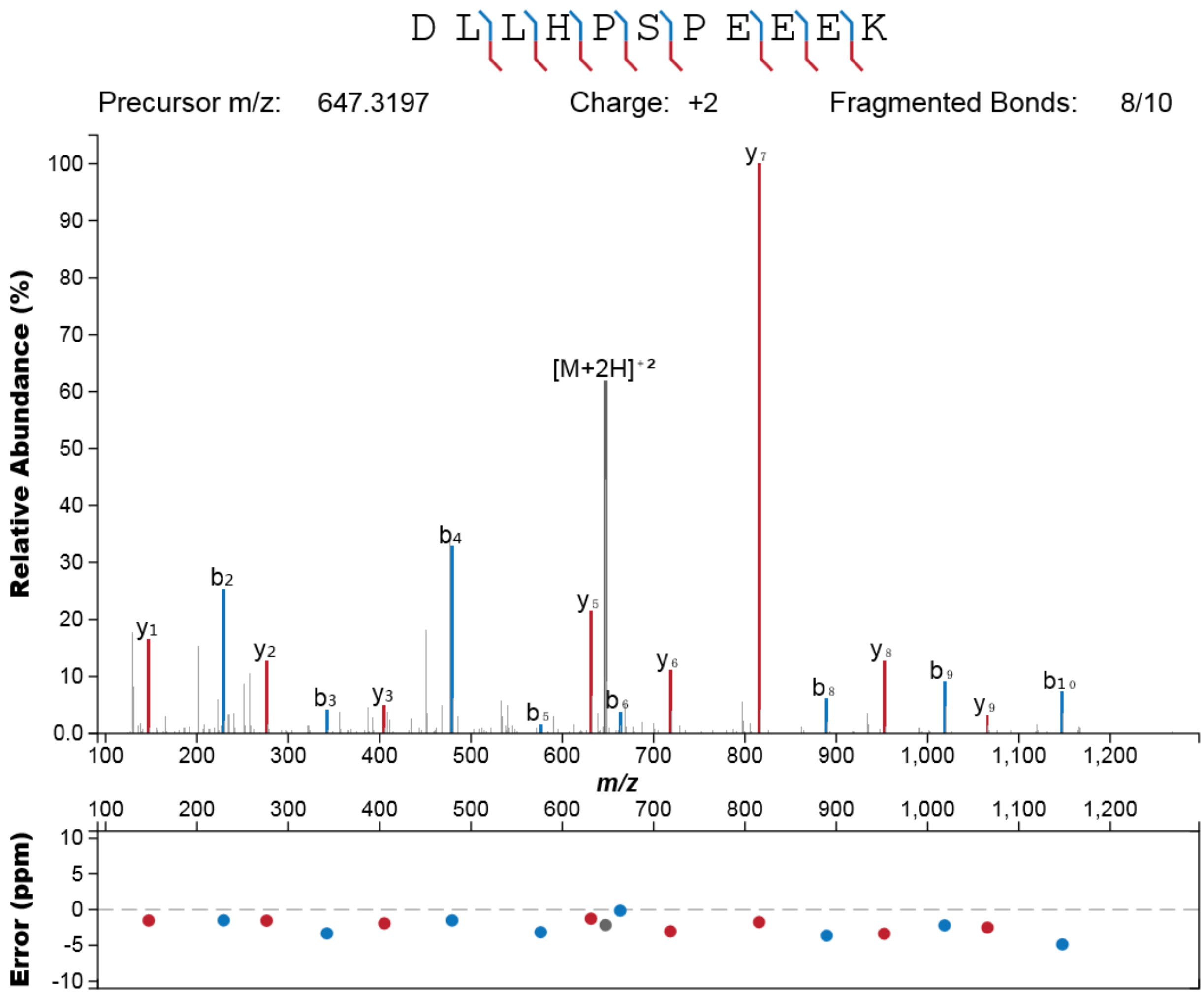
D L L H P S P E E E K
Precursor m/z: 647.3197
Charge: +2
Fragmented Bonds: 8/10
Relative Abundance (%)
m/z
Error (ppm)
[M+2H]+2
y1
y2
y3
y5
y6
y7
y8
y9
b2
b3
b4
b5
b6
b8
b9
b10

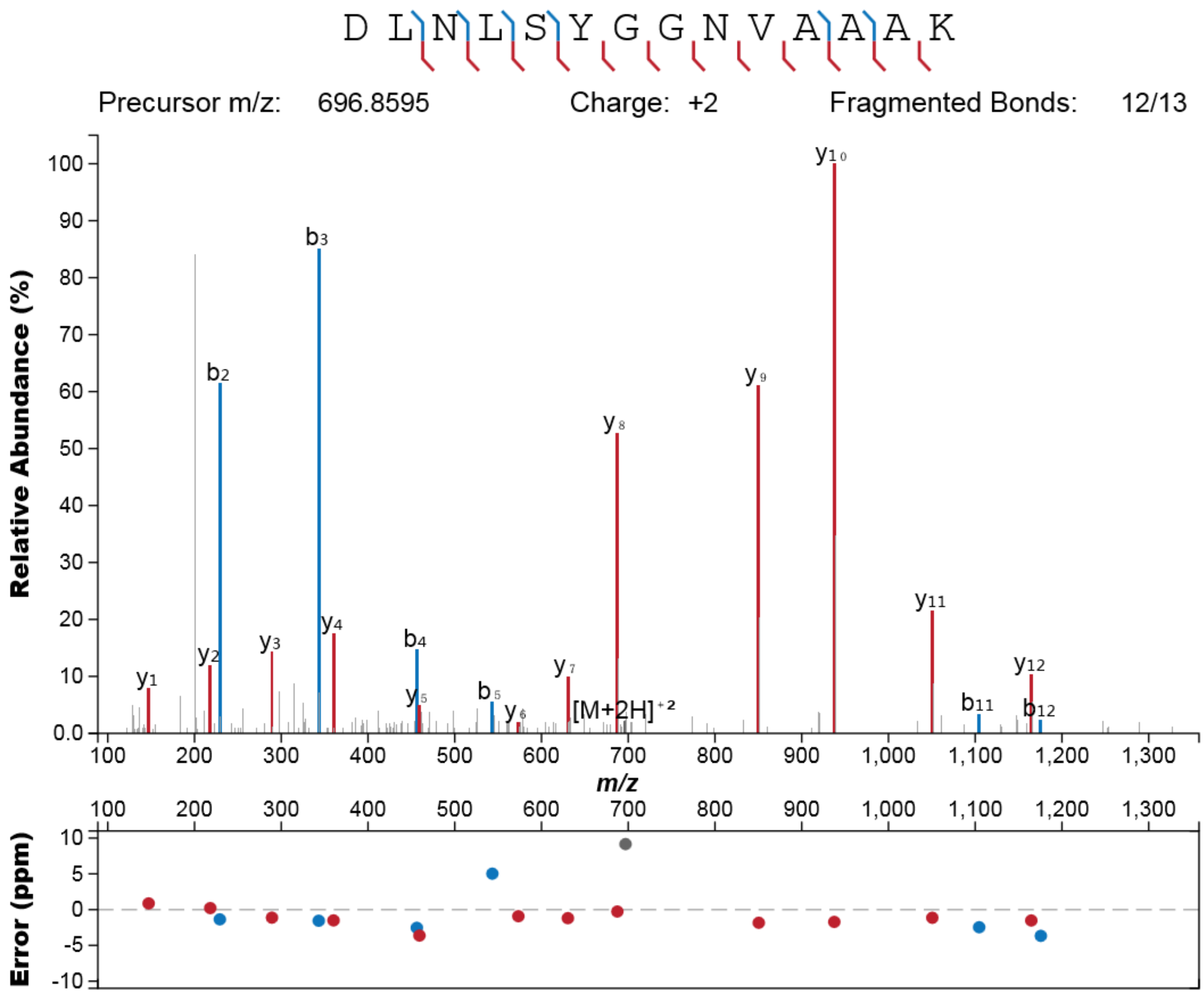
D L N L S Y G G N V A A A K
Precursor m/z: 696.8595
Charge: +2
Fragmented Bonds: 12/13
Relative Abundance (%)
m/z
Error (ppm)
y1 y2 b2 y3 b3 y4 b4 y5 b5 y6 y7 y8 [M+2H]+2 y9 y10 y11 b11 y12 b12

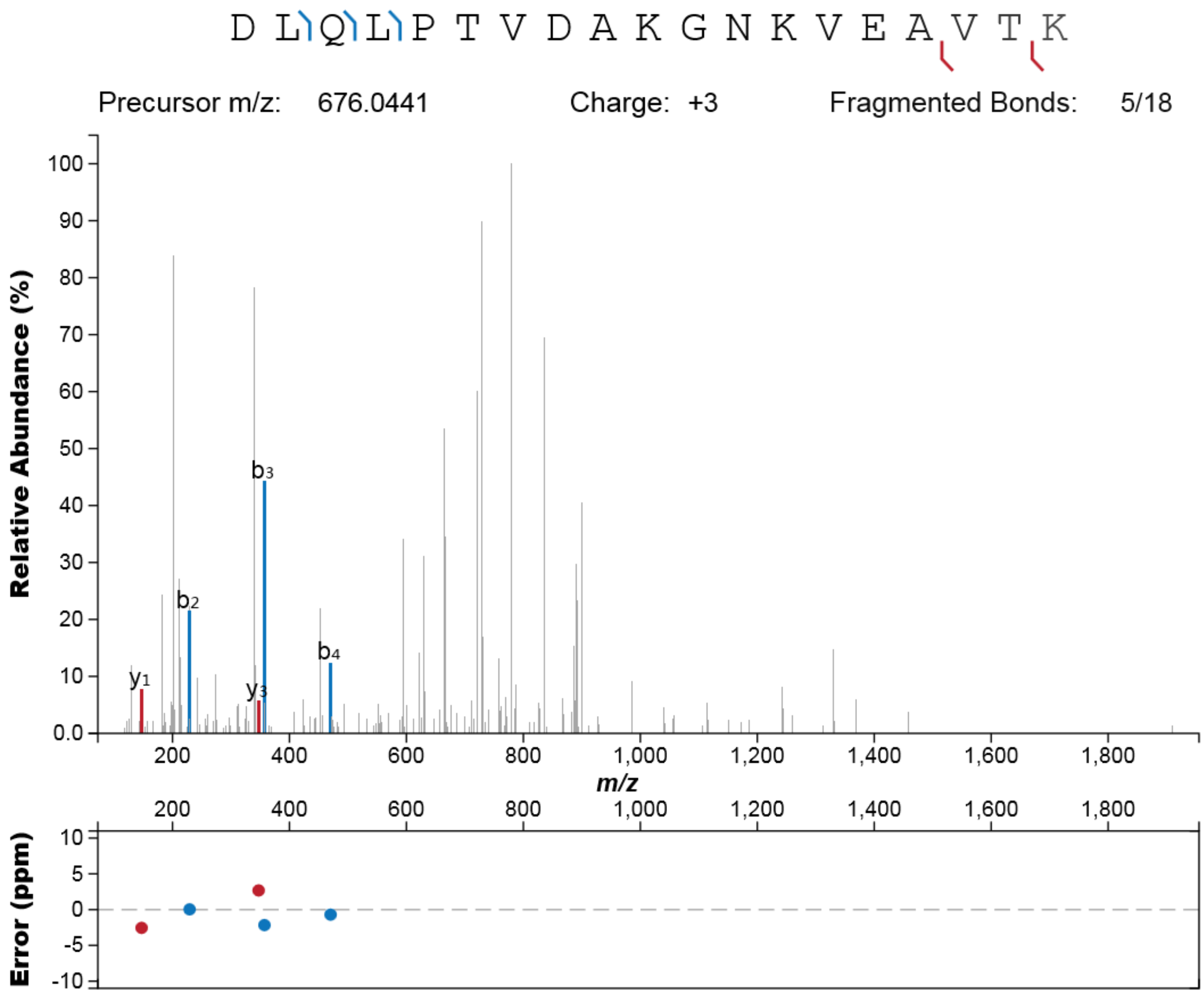

D L Q L P T V D A K G N K V E A V T K
Precursor m/z: 676.0441
Charge: +3
Fragmented Bonds: 5/18
Relative Abundance (%)
m/z
Error (ppm)
y1
b2
y3
b3
b4

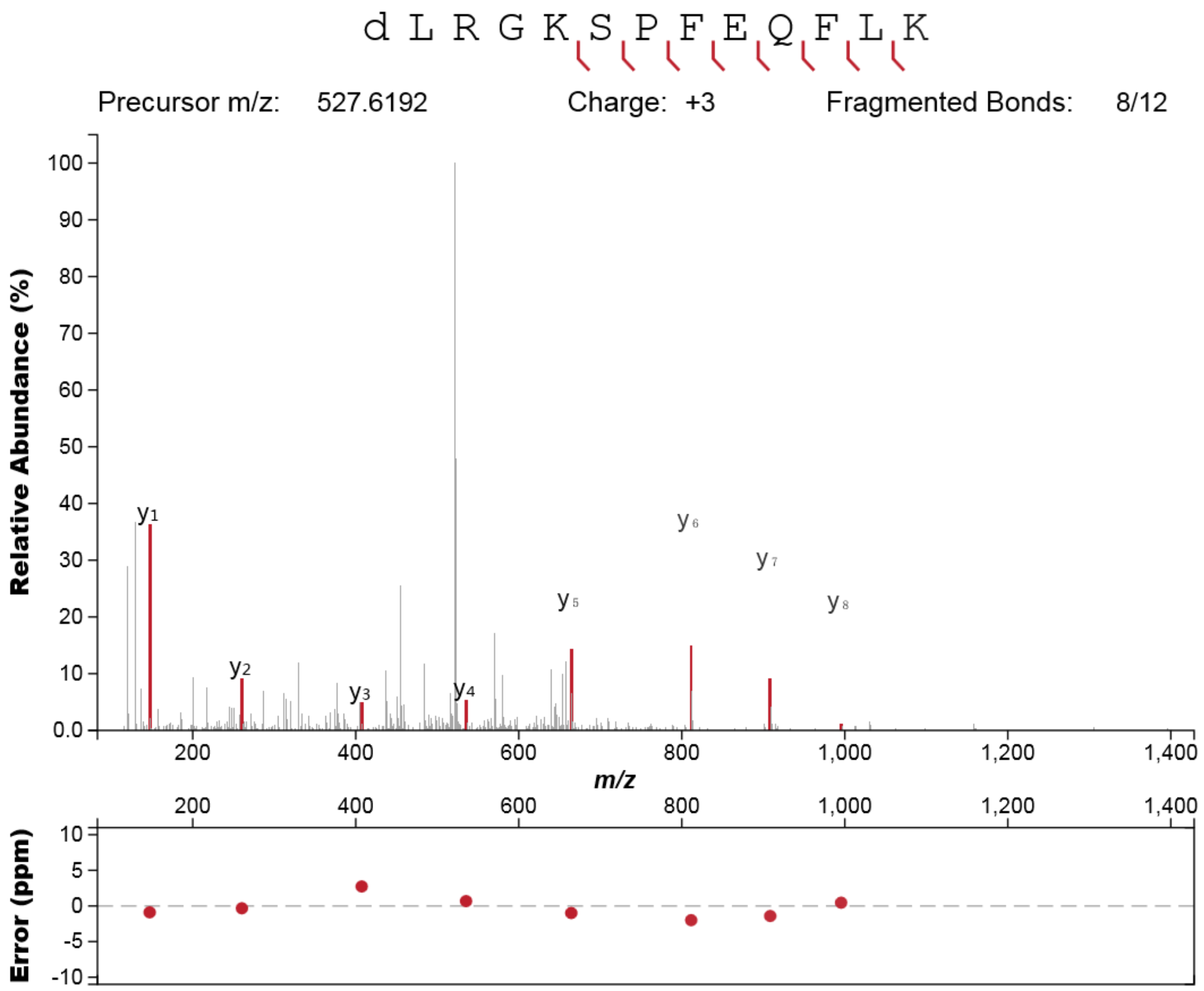

d L R G K S P F E Q F L K
Precursor m/z: 527.6192
Charge: +3
Fragmented Bonds: 8/12
Relative Abundance (%)
m/z
Error (ppm)
y1
y2
y3
y4
y5
y6
y7
y8

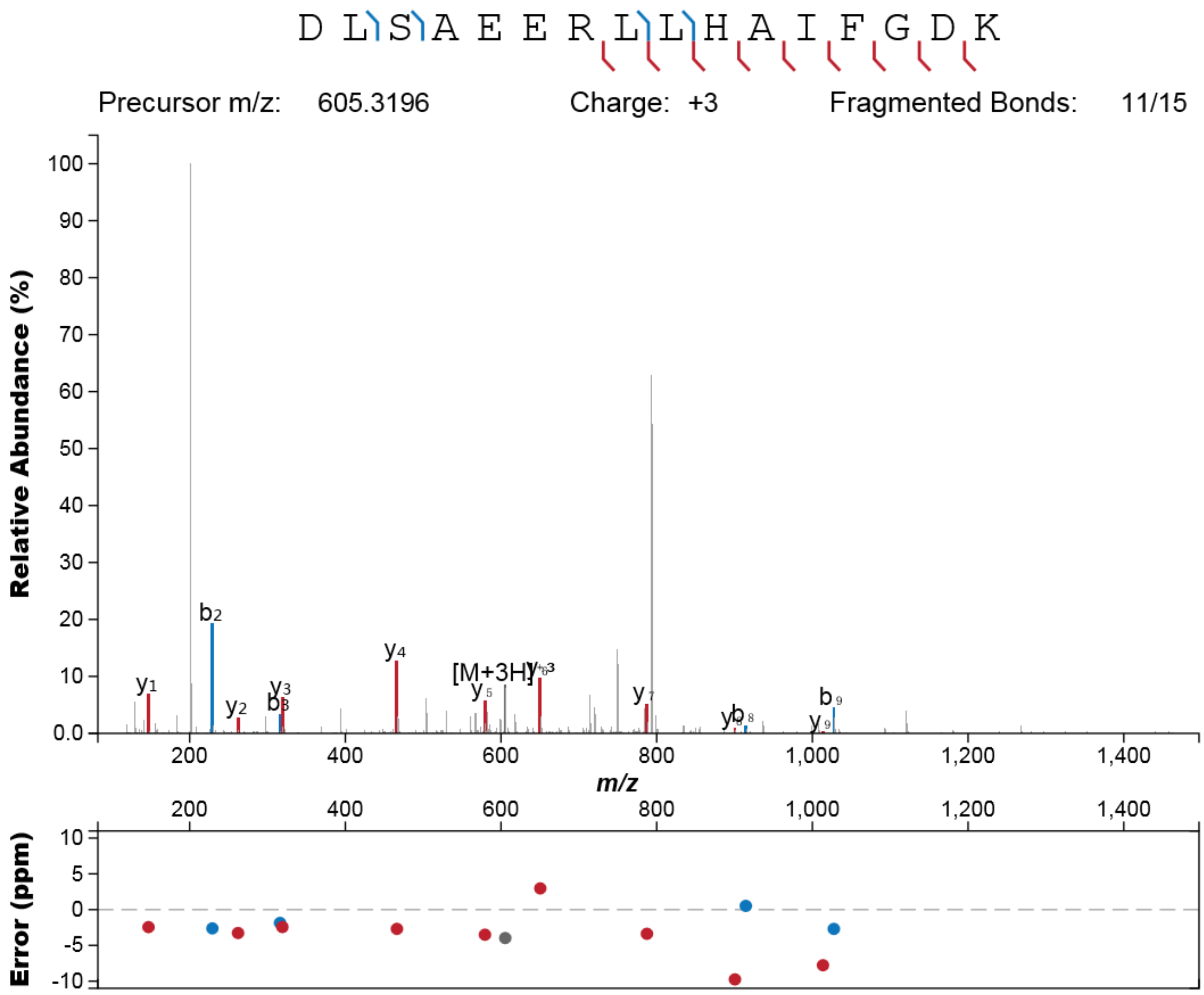
D L S A E E R L L H A I F G D K
Precursor m/z: 605.3196
Charge: +3
Fragmented Bonds: 11/15
Relative Abundance (%)
m/z
Error (ppm)

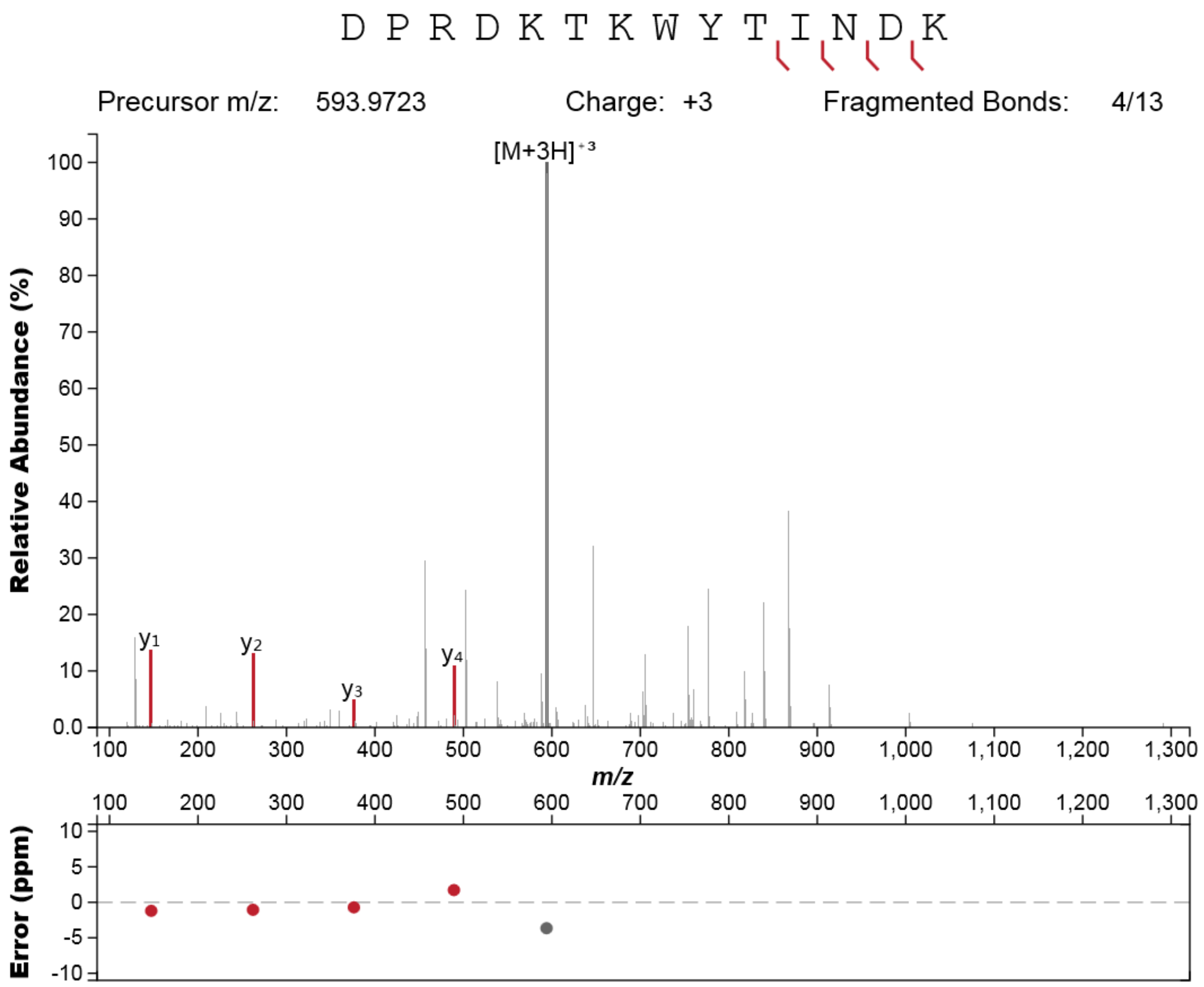
D P R D K T K W Y T I N D K
Precursor m/z: 593.9723
Charge: +3
Fragmented Bonds: 4/13
[M+3H]+3
y1
y2
y3
y4
Relative Abundance (%)
m/z
Error (ppm)

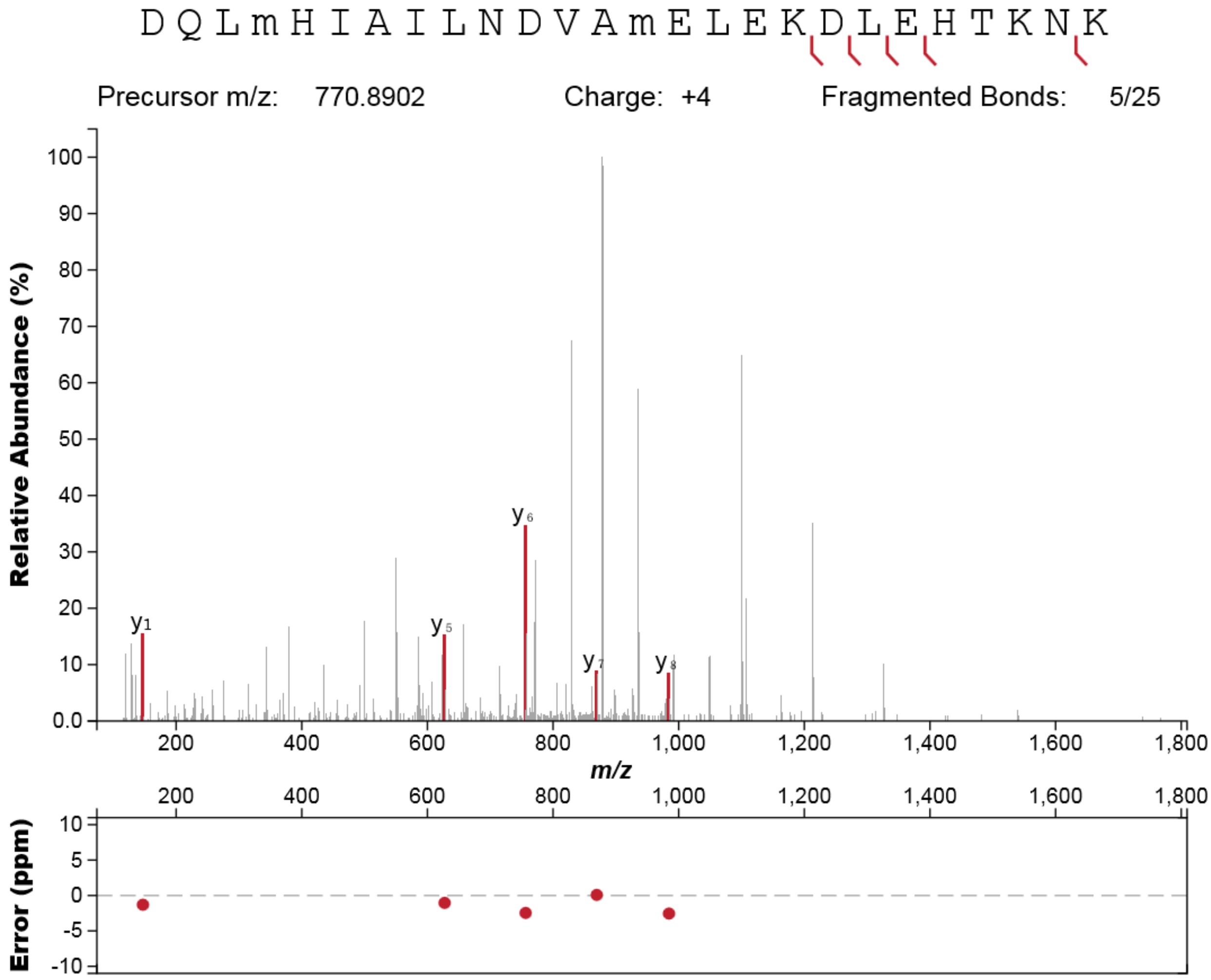
D Q L m H I A I L N D V A m E L E K D L E H T K N K
Precursor m/z: 770.8902
Charge: +4
Fragmented Bonds: 5/25
Relative Abundance (%)
y1
y5
y6
y7
y8
m/z
Error (ppm)

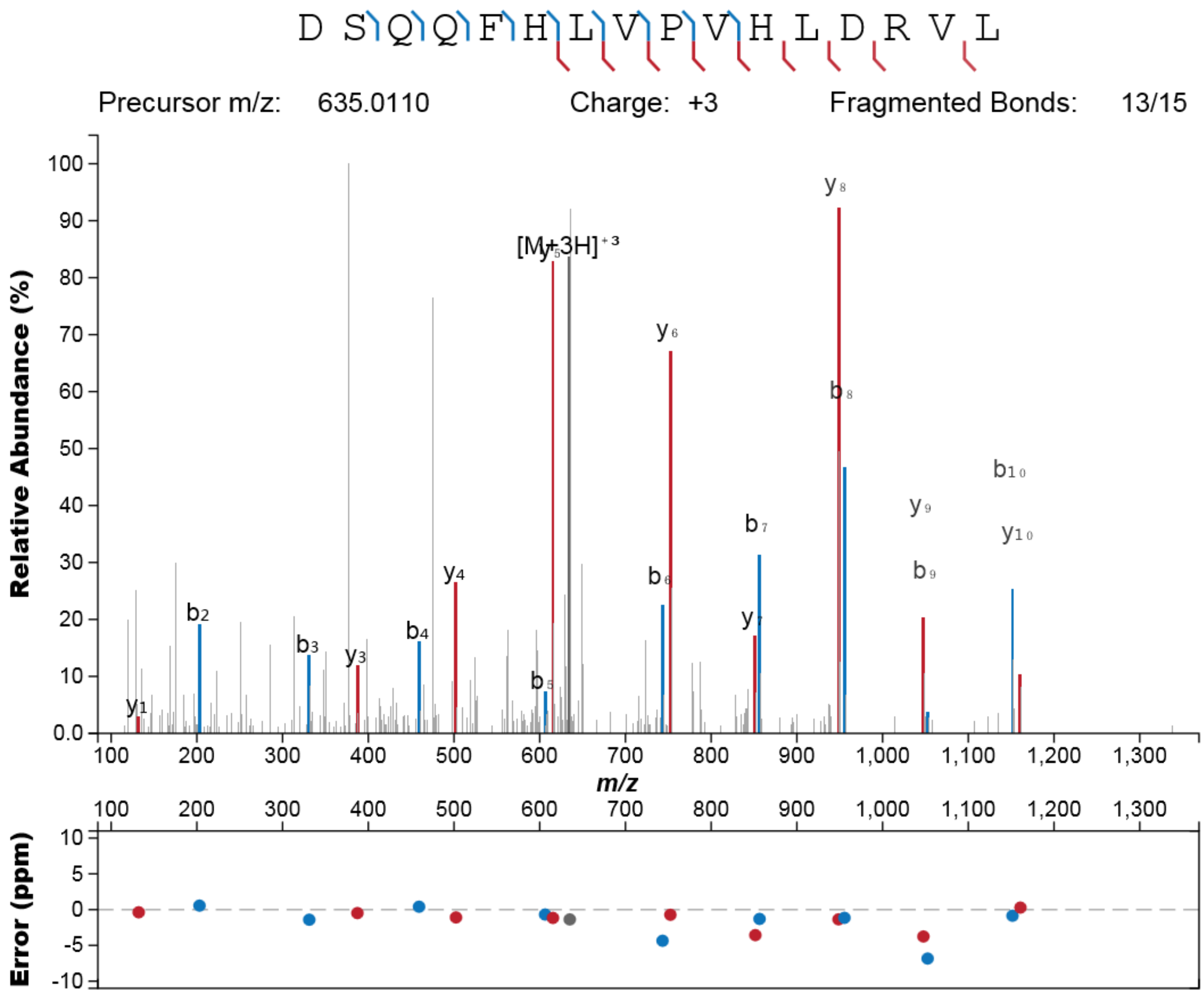
D S Q Q F H L V P V H L D R V L
Precursor m/z: 635.0110
Charge: +3
Fragmented Bonds: 13/15
Relative Abundance (%)
m/z
Error (ppm)
[M+3H]+3
y1 y3 y4 y5 y6 y7 y8 y9 y10
b2 b3 b4 b5 b6 b7 b8 b9 b10

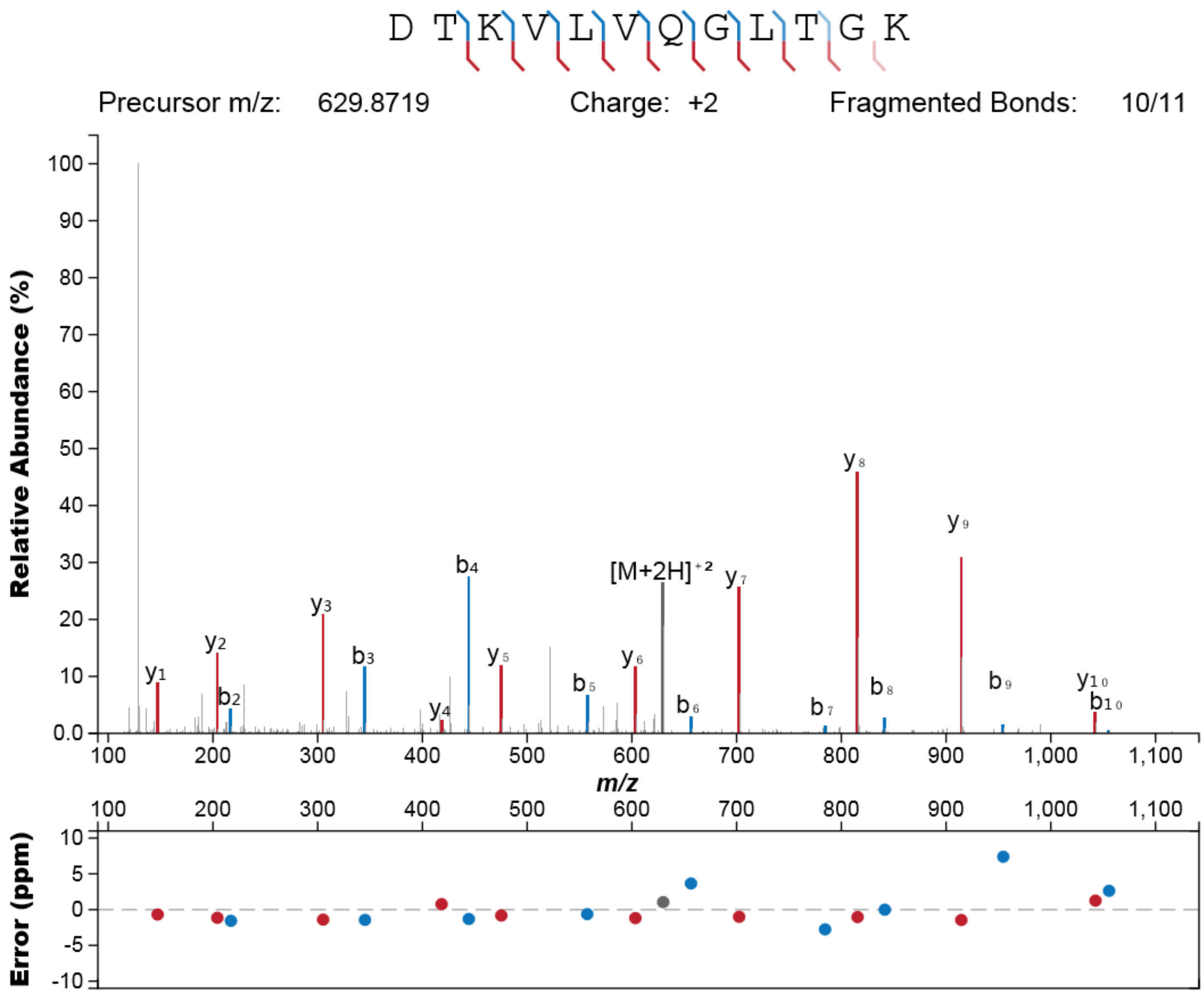
D T K V L V Q G L T G K
Precursor m/z: 629.8719
Charge: +2
Fragmented Bonds: 10/11
Relative Abundance (%)
m/z
Error (ppm)
[M+2H]+2
y1
y2
b2
y3
b3
y4
b4
y5
b5
y6
b6
y7
b7
y8
b8
y9
b9
y10
b10

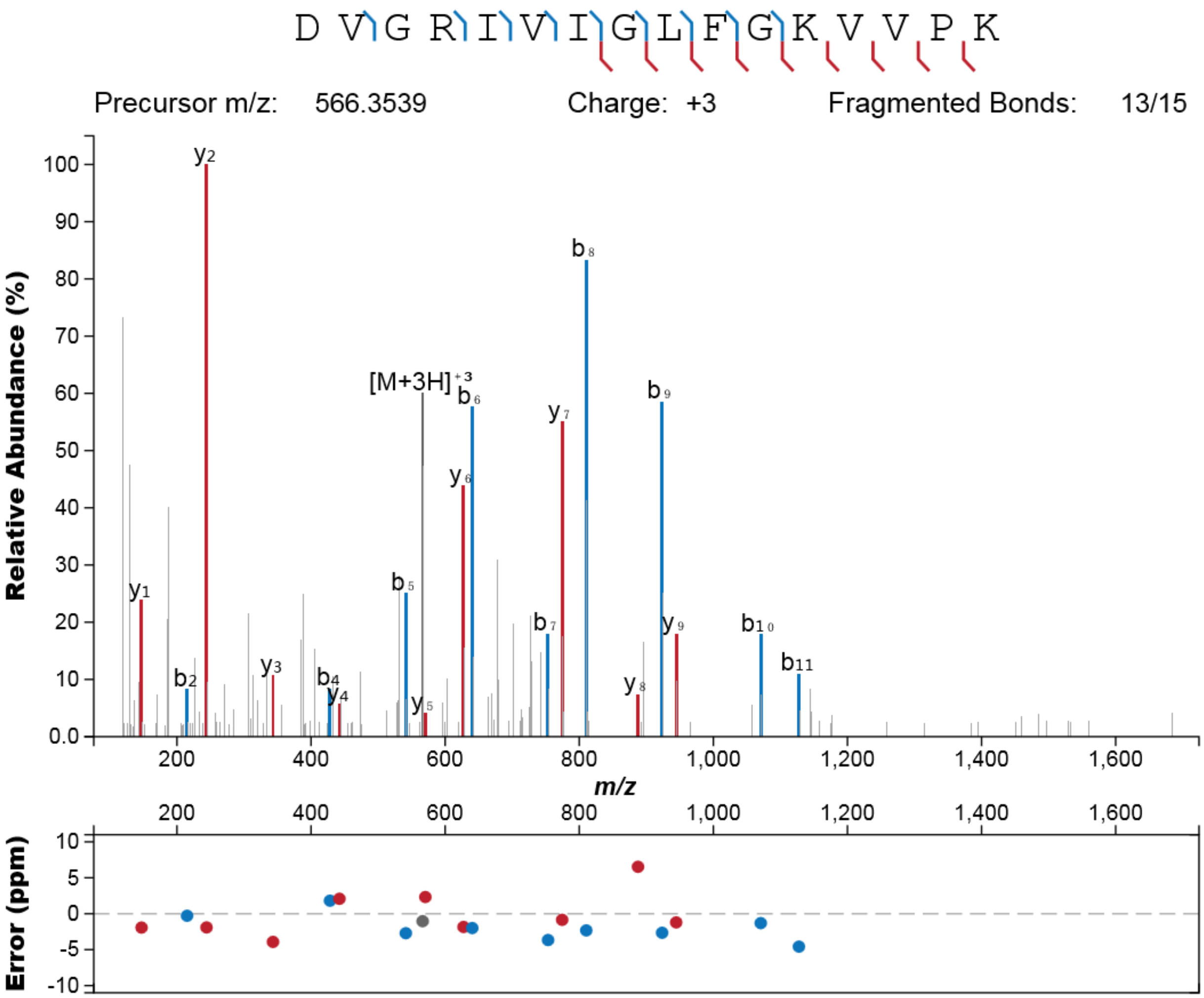
D V G R I V I G L F G K V V P K
Precursor m/z: 566.3539
Charge: +3
Fragmented Bonds: 13/15
Relative Abundance (%)
m/z
Error (ppm)
[M+3H]+3

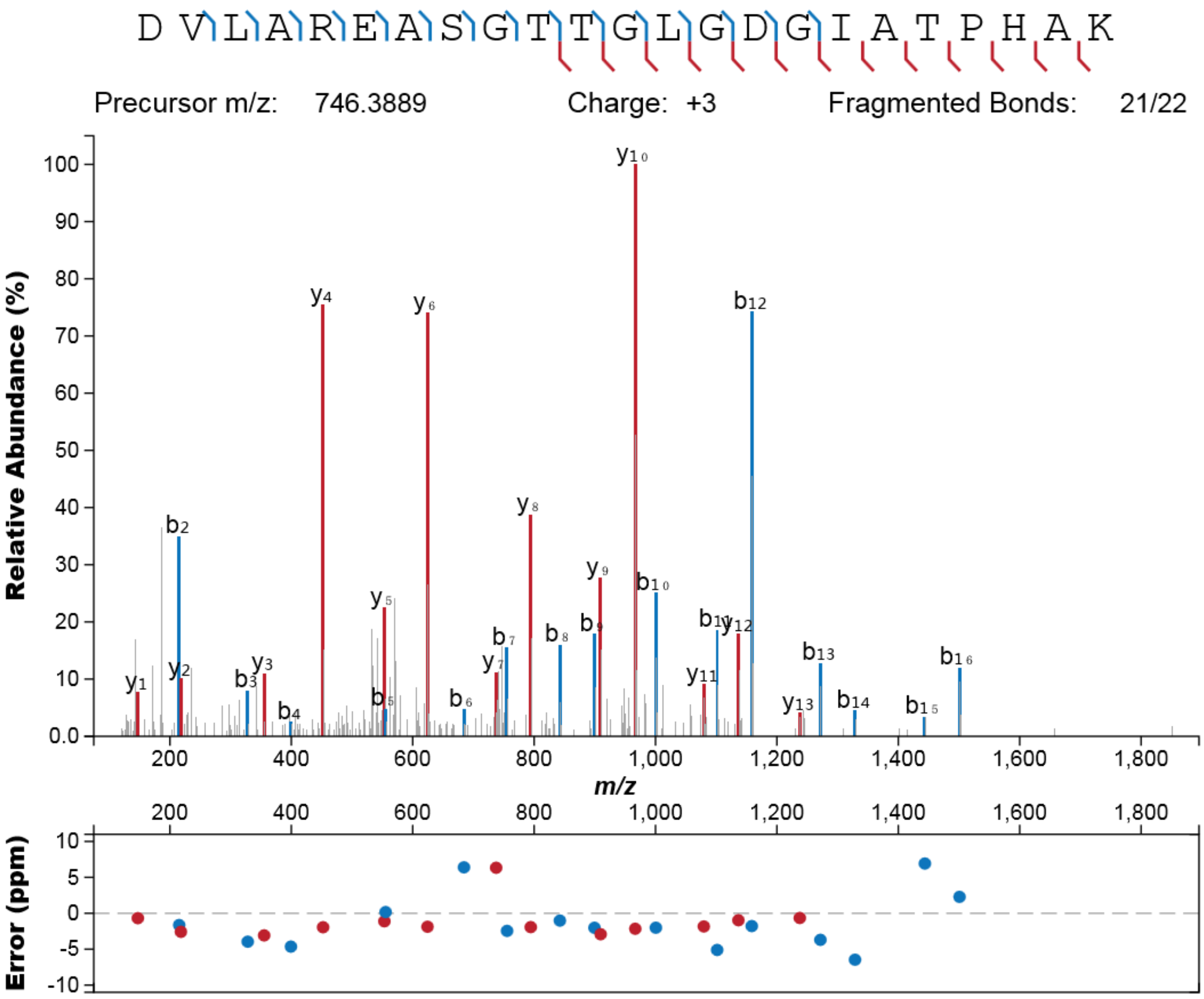

D V L A R E A S G T T G L G D G I A T P H A K
Precursor m/z: 746.3889
Charge: +3
Fragmented Bonds: 21/22
Relative Abundance (%)
m/z
Error (ppm)

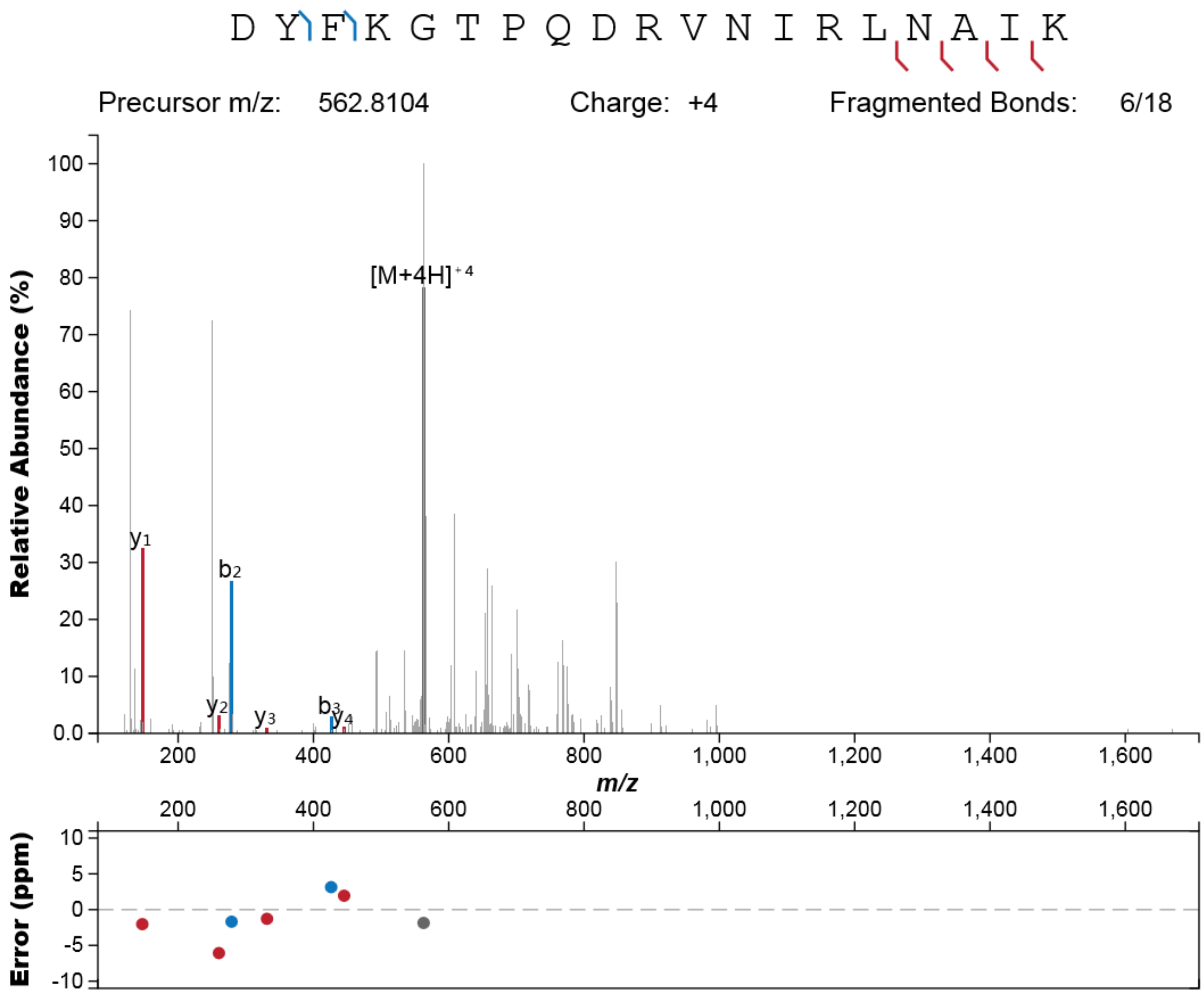

D Y F K G T P Q D R V N I R L N A I K
Precursor m/z: 562.8104
Charge: +4
Fragmented Bonds: 6/18
Relative Abundance (%)
[M+4H]+4
y1
b2
y2
y3
b3
y4
m/z
Error (ppm)

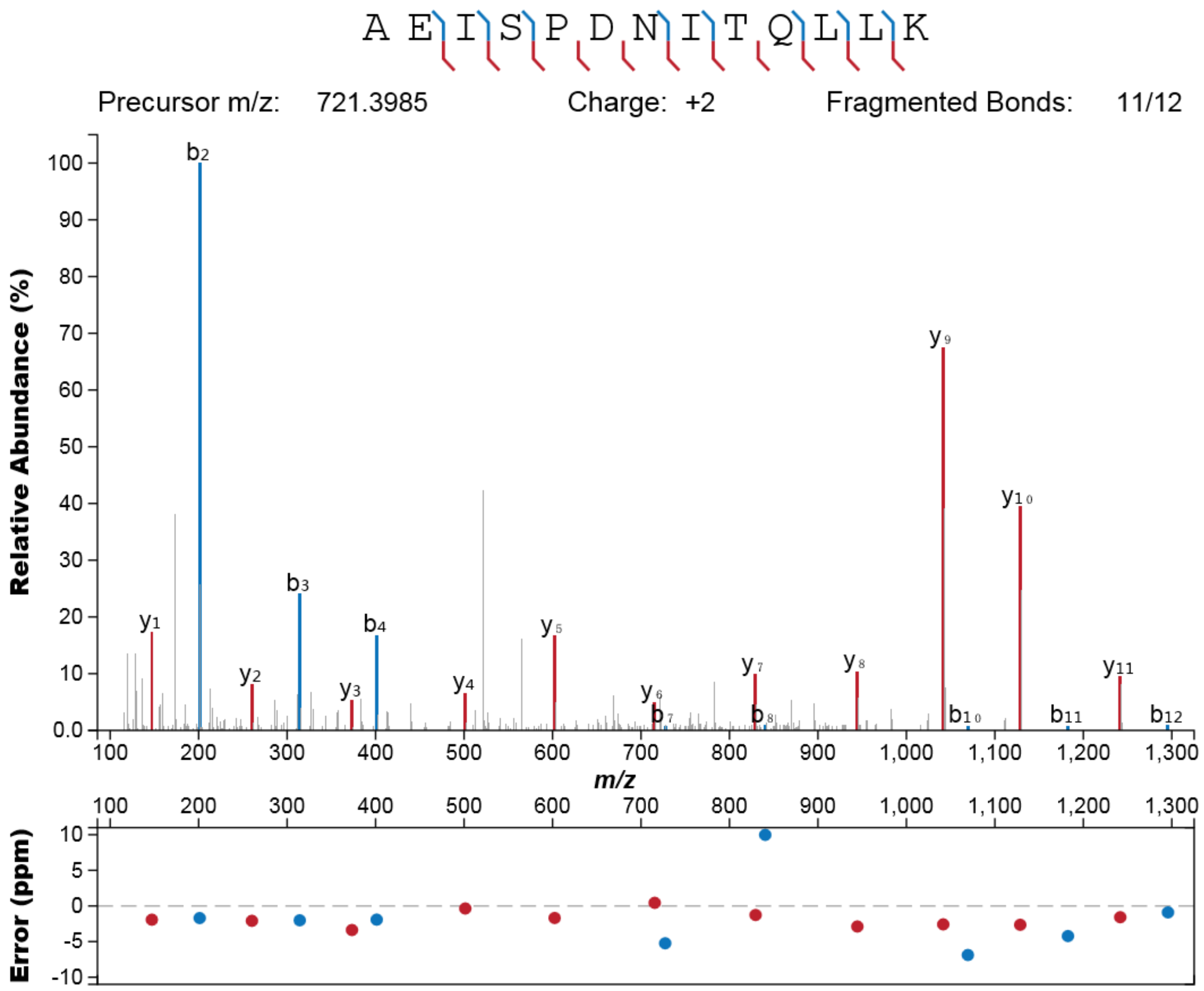

A E I S P D N I T Q L L K
Precursor m/z: 721.3985
Charge: +2
Fragmented Bonds: 11/12
Relative Abundance (%)
m/z
Error (ppm)
b2
b3
b4
b7
b8
b10
b11
b12
y1
y2
y3
y4
y5
y6
y7
y8
y9
y10
y11

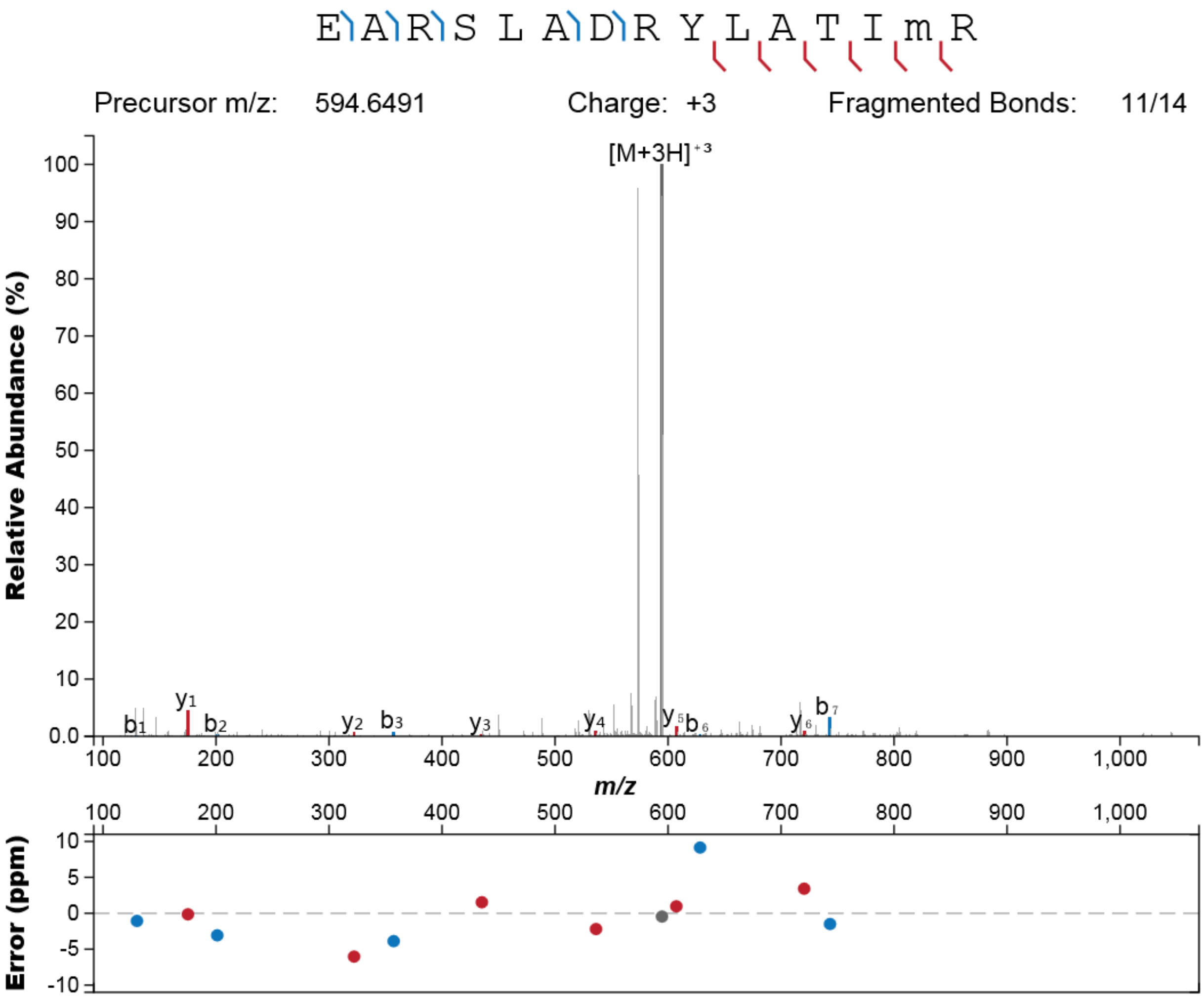

E A R S L A D R Y L A T I m R
Precursor m/z: 594.6491
Charge: +3
Fragmented Bonds: 11/14
[M+3H]+3
Relative Abundance (%)
m/z
Error (ppm)
b1
y1
b2
y2
b3
y3
y4
y5
b6
y6
b7

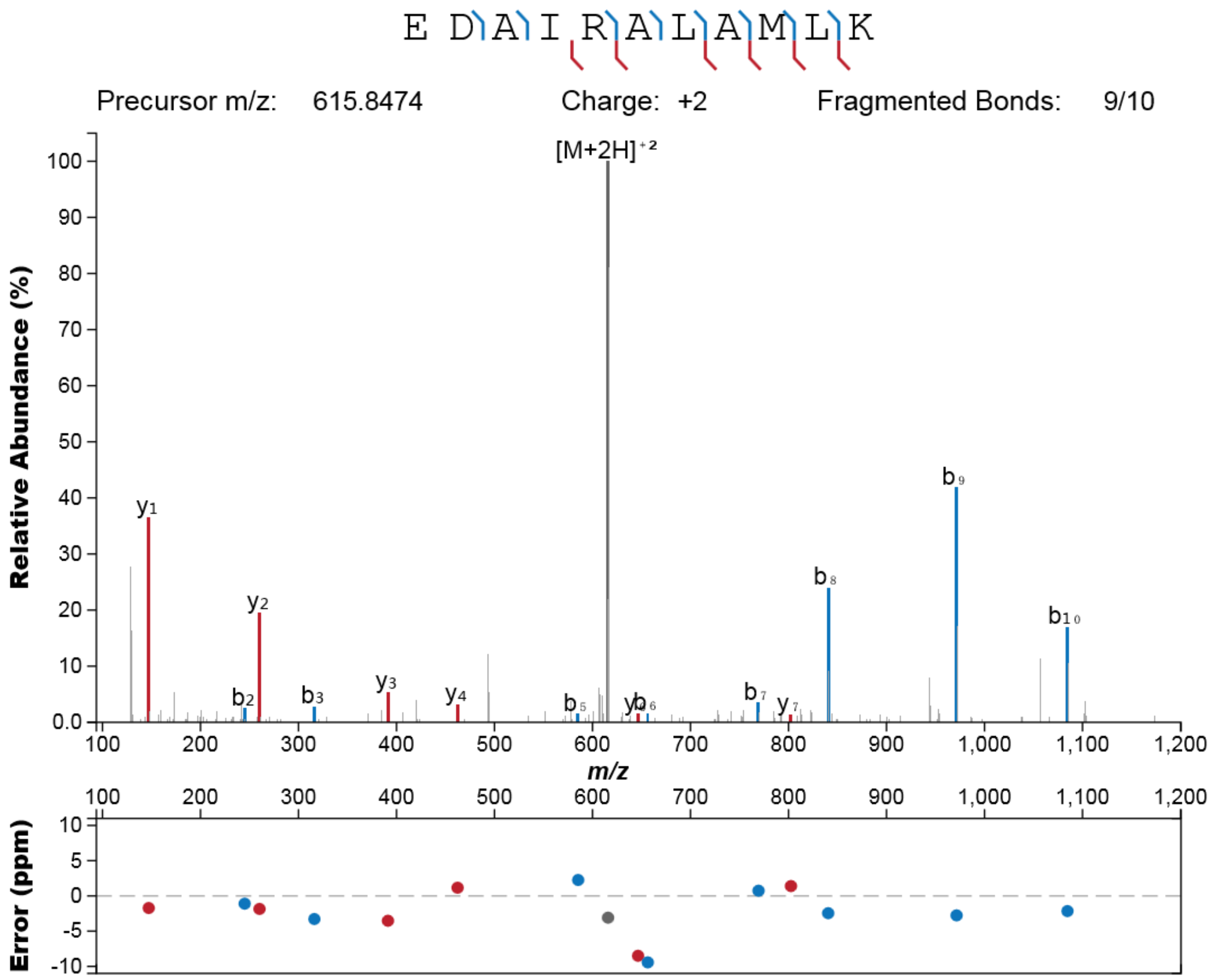

E D A I R A L A M L K
Precursor m/z: 615.8474
Charge: +2
Fragmented Bonds: 9/10
[M+2H]+2
Relative Abundance (%)
m/z
Error (ppm)
y1
y2
y3
y4
b2
b3
b5
b6
b7
y7
b8
b9
b10

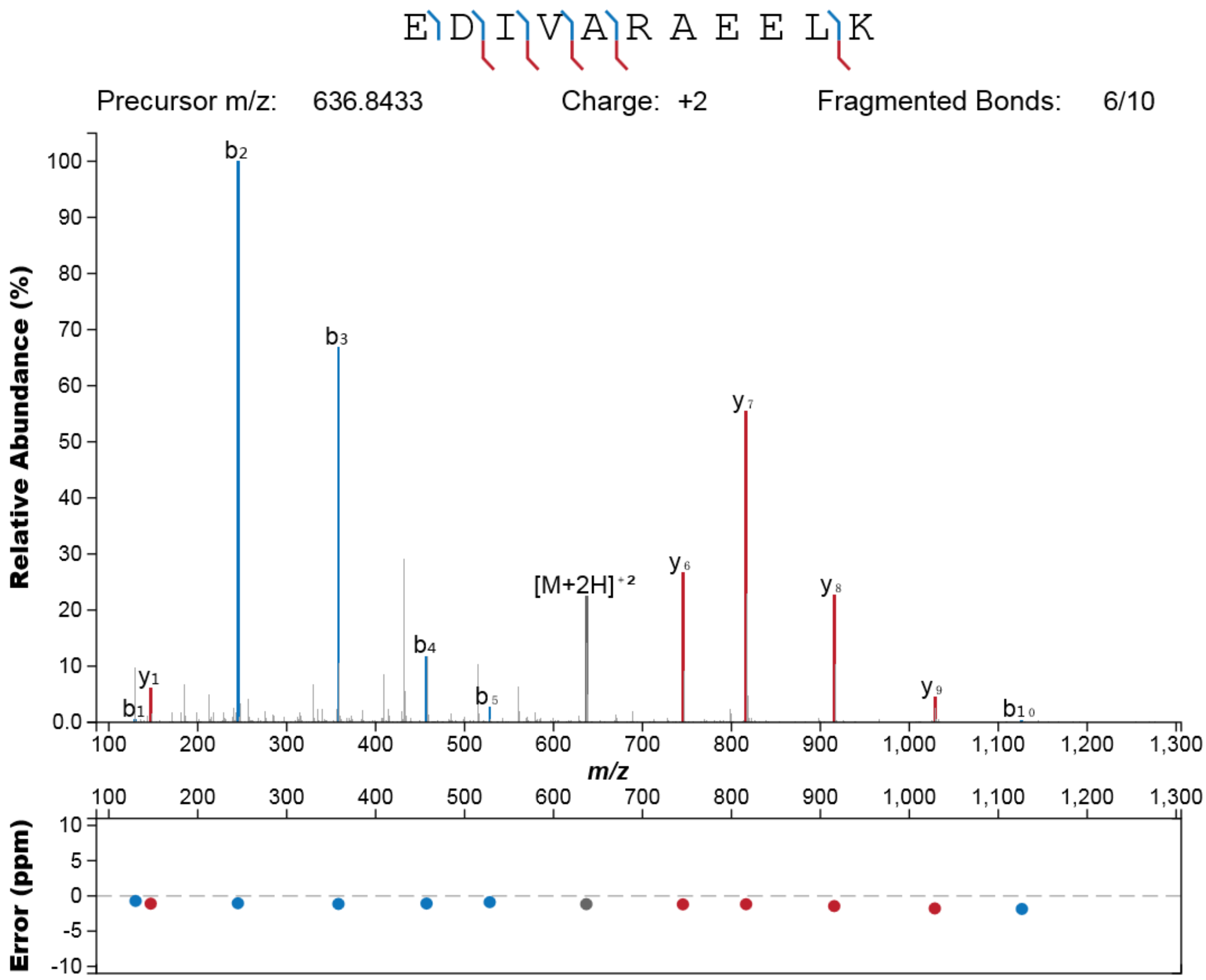

E D I V A R A E E L K
Precursor m/z: 636.8433
Charge: +2
Fragmented Bonds: 6/10
Relative Abundance (%)
b2
b3
y7
y6
[M+2H]+2
y8
b4
y1
b5
y9
b1
b10
m/z
Error (ppm)

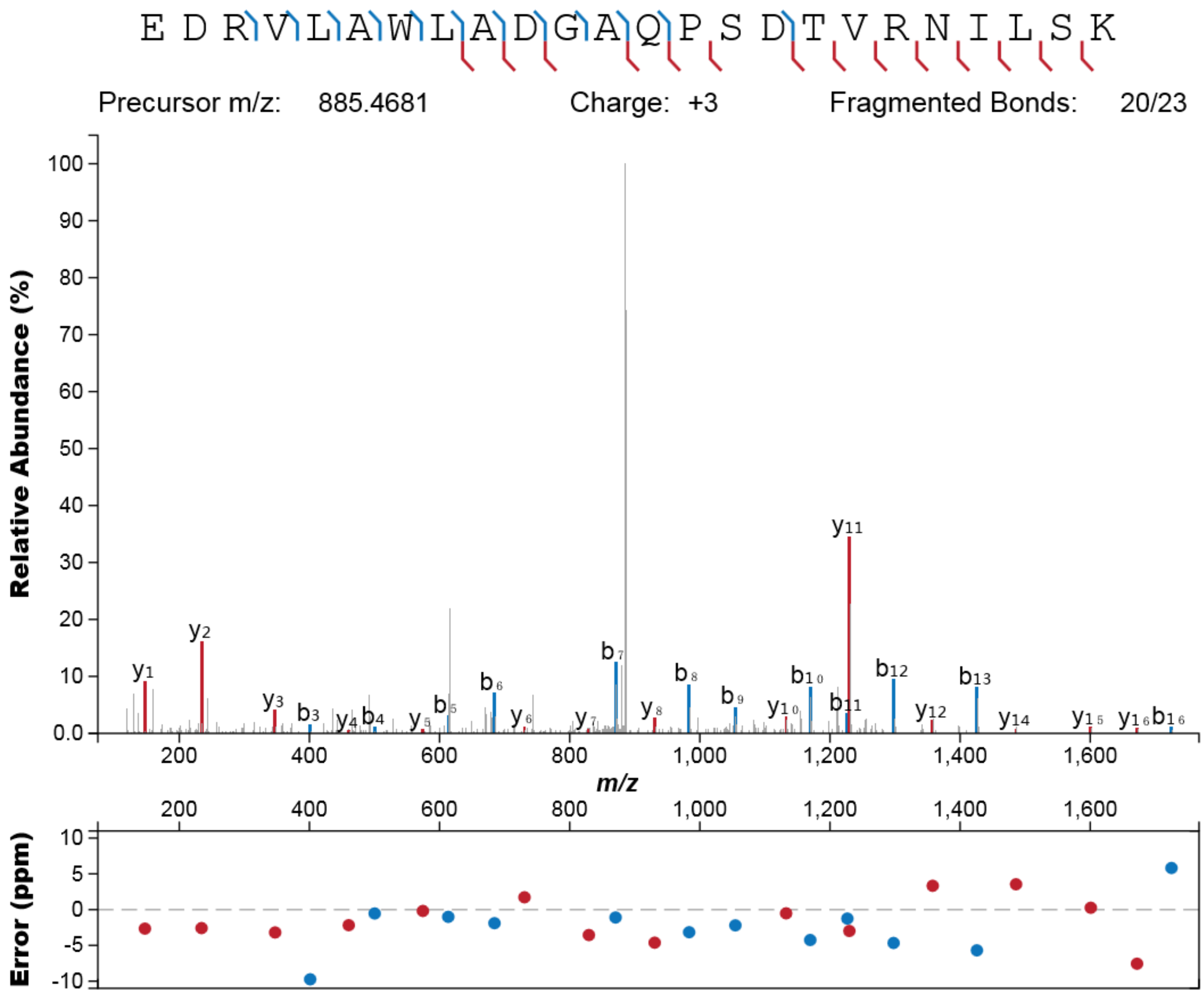

E D R V L A W L A D G A Q P S D T V R N I L S K
Precursor m/z: 885.4681
Charge: +3
Fragmented Bonds: 20/23
Relative Abundance (%)
m/z
Error (ppm)
y1 y2 y3 b3 y4 b4 y5 b5 b6 y6 y7 b7 y8 b8 b9 y10 b10 b11 y11 b12 y12 b13 y14 y15 y16 b16

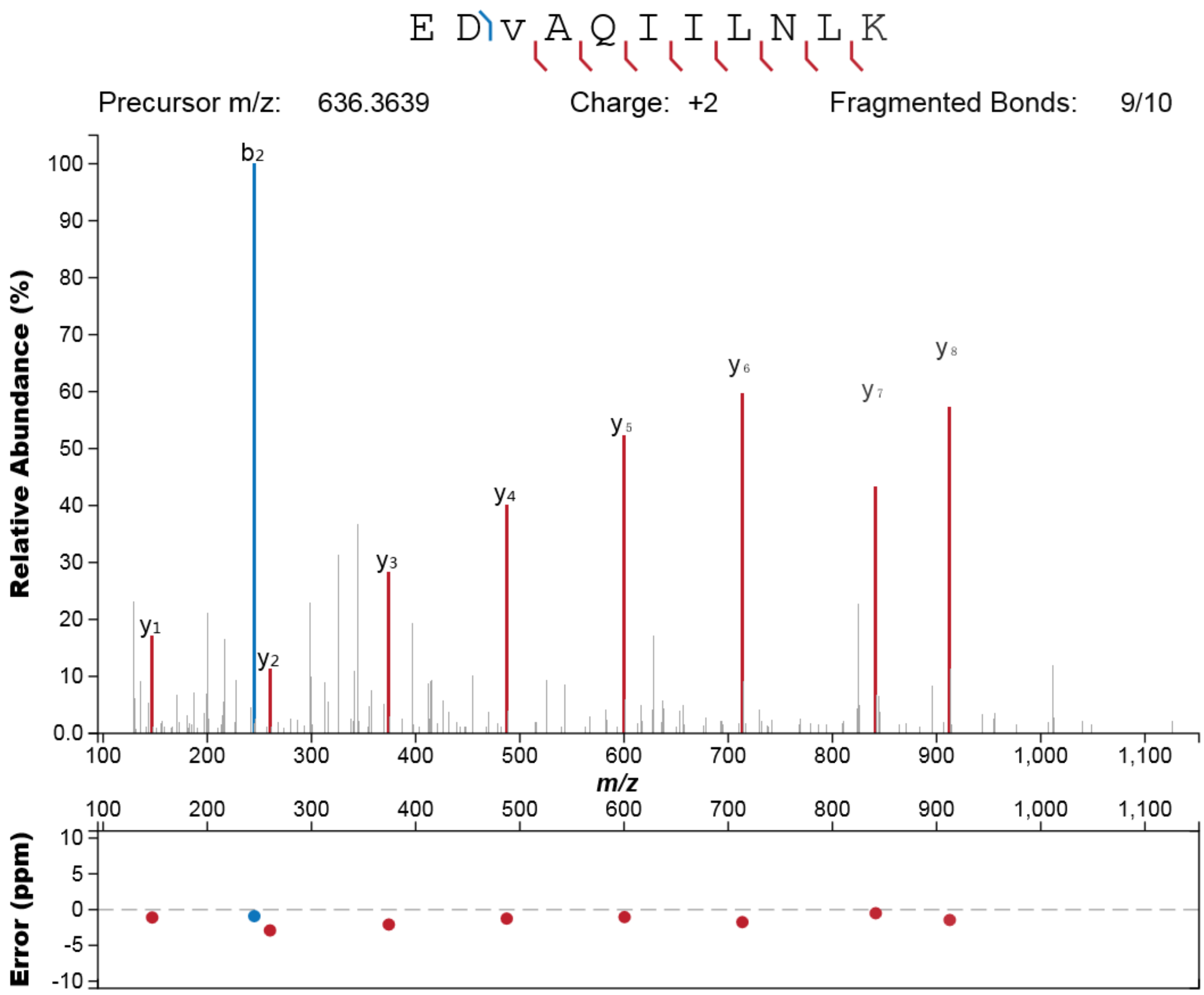
E D v A Q I I L N L K
Precursor m/z: 636.3639
Charge: +2
Fragmented Bonds: 9/10
Relative Abundance (%)
m/z
Error (ppm)
b2
y1
y2
y3
y4
y5
y6
y7
y8

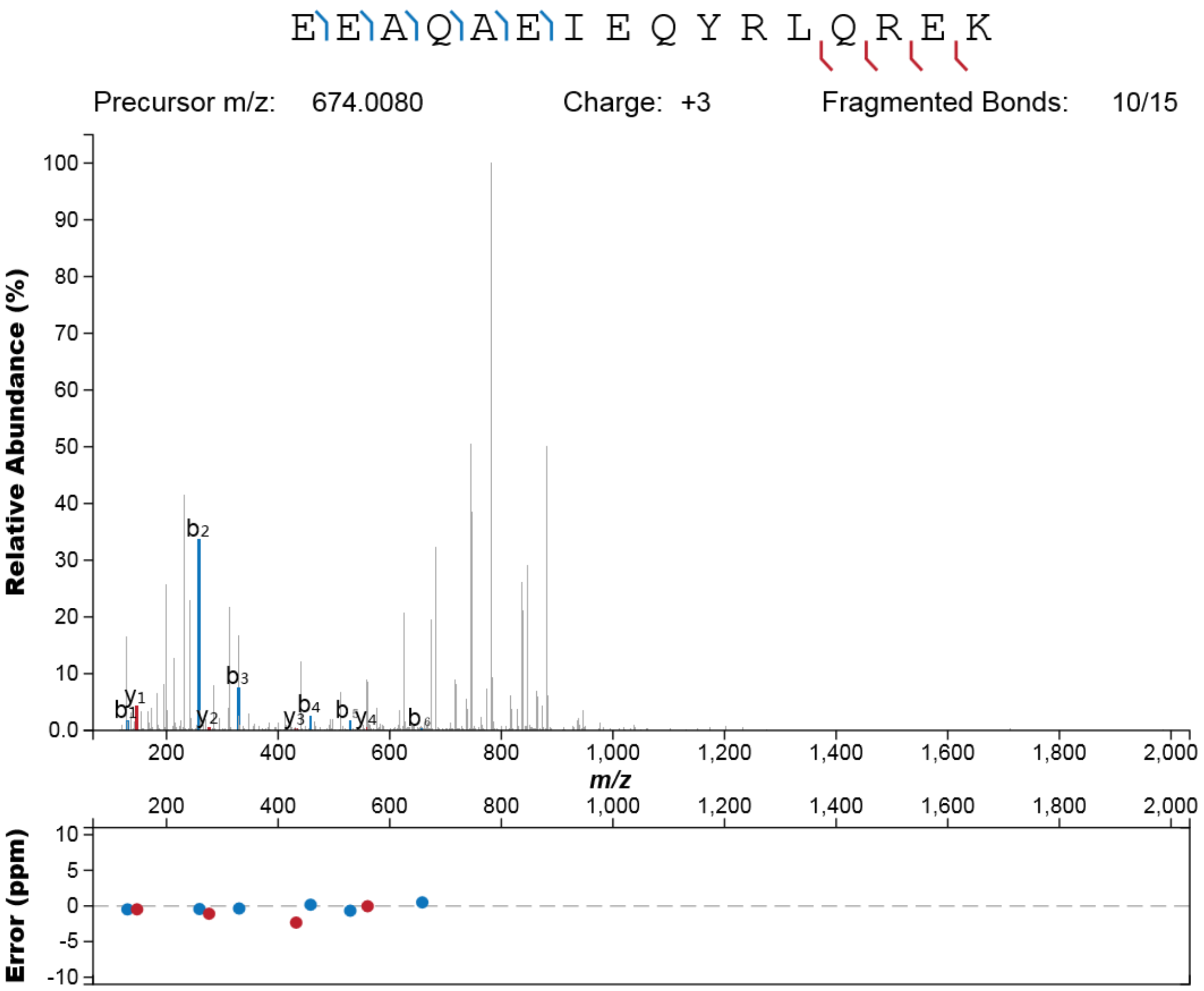
E E A Q A E I E Q Y R L Q R E K
Precursor m/z: 674.0080
Charge: +3
Fragmented Bonds: 10/15
Relative Abundance (%)
m/z
Error (ppm)
b2
b3
b4
b1
y1
y2
y3
y4
b6

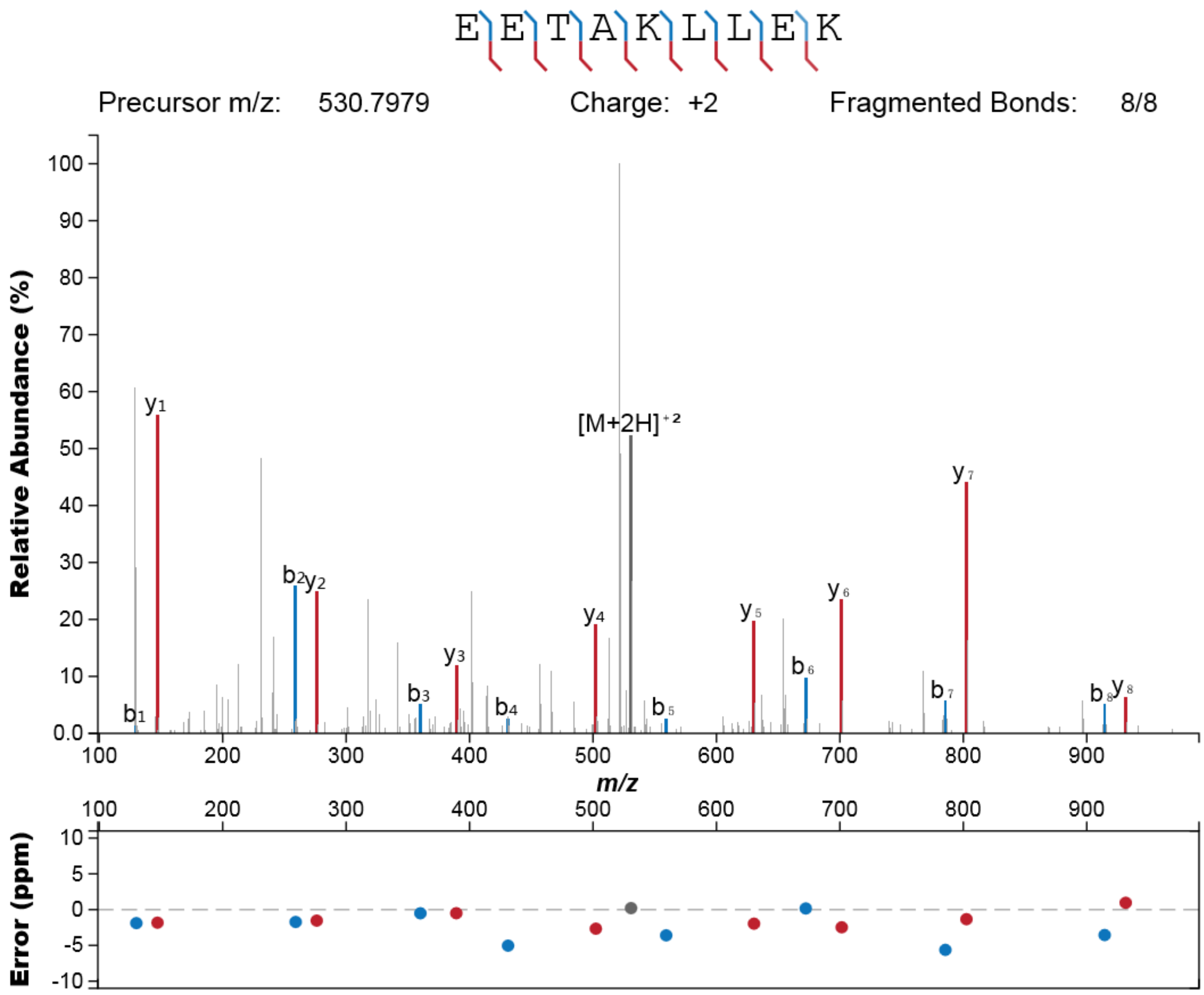

E E T A K L L L E K
Precursor m/z: 530.7979
Charge: +2
Fragmented Bonds: 8/8
Relative Abundance (%)
[M+2H]+2
y1 y2 y3 y4 y5 y6 y7 y8
b1 b2 b3 b4 b5 b6 b7 b8
m/z
Error (ppm)

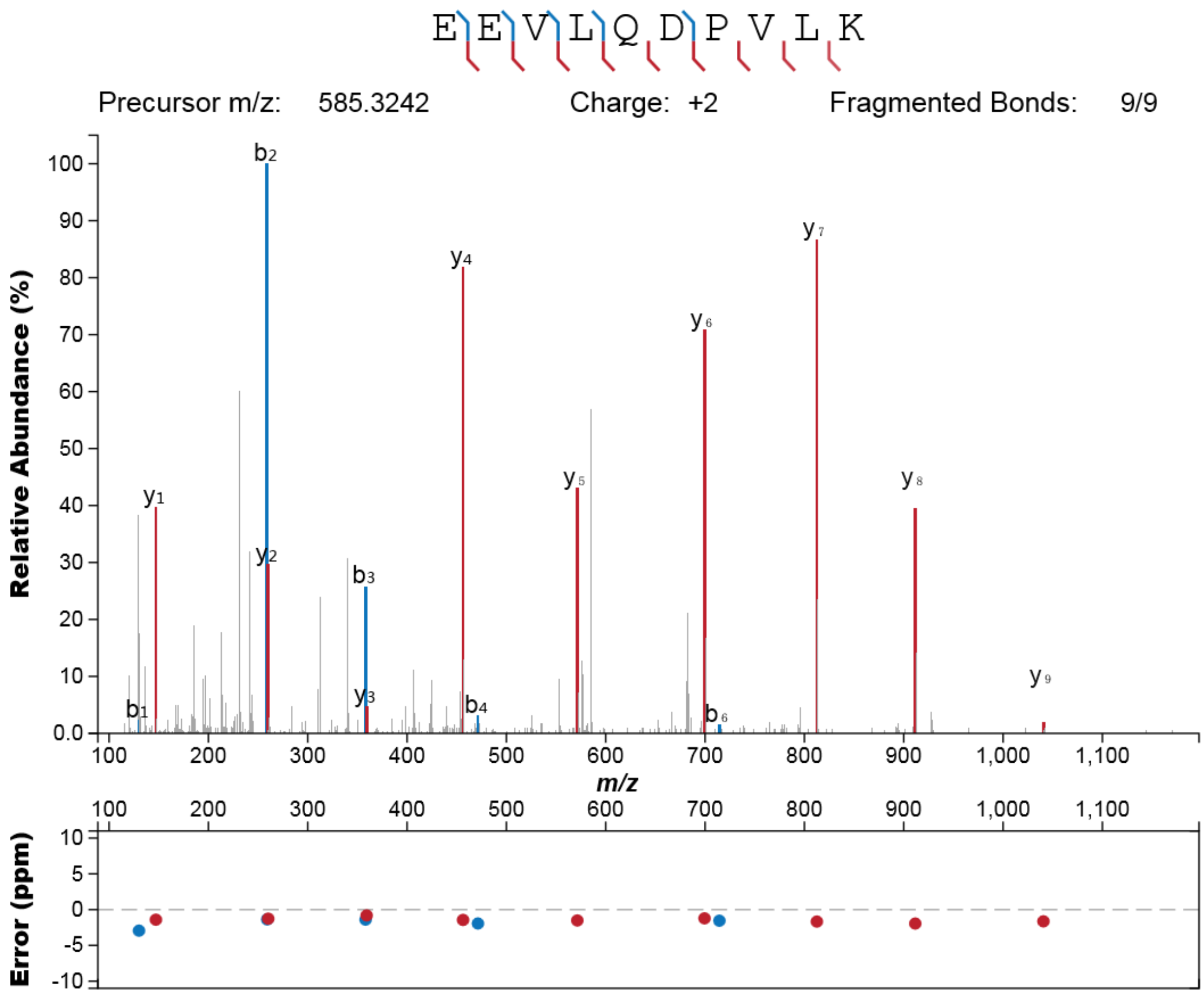

E E V L Q D P V L K
Precursor m/z: 585.3242
Charge: +2
Fragmented Bonds: 9/9
Relative Abundance (%)
b2
y7
y4
y6
y5
y8
y1
y2
b3
y3
b4
b1
b6
y9
m/z
Error (ppm)

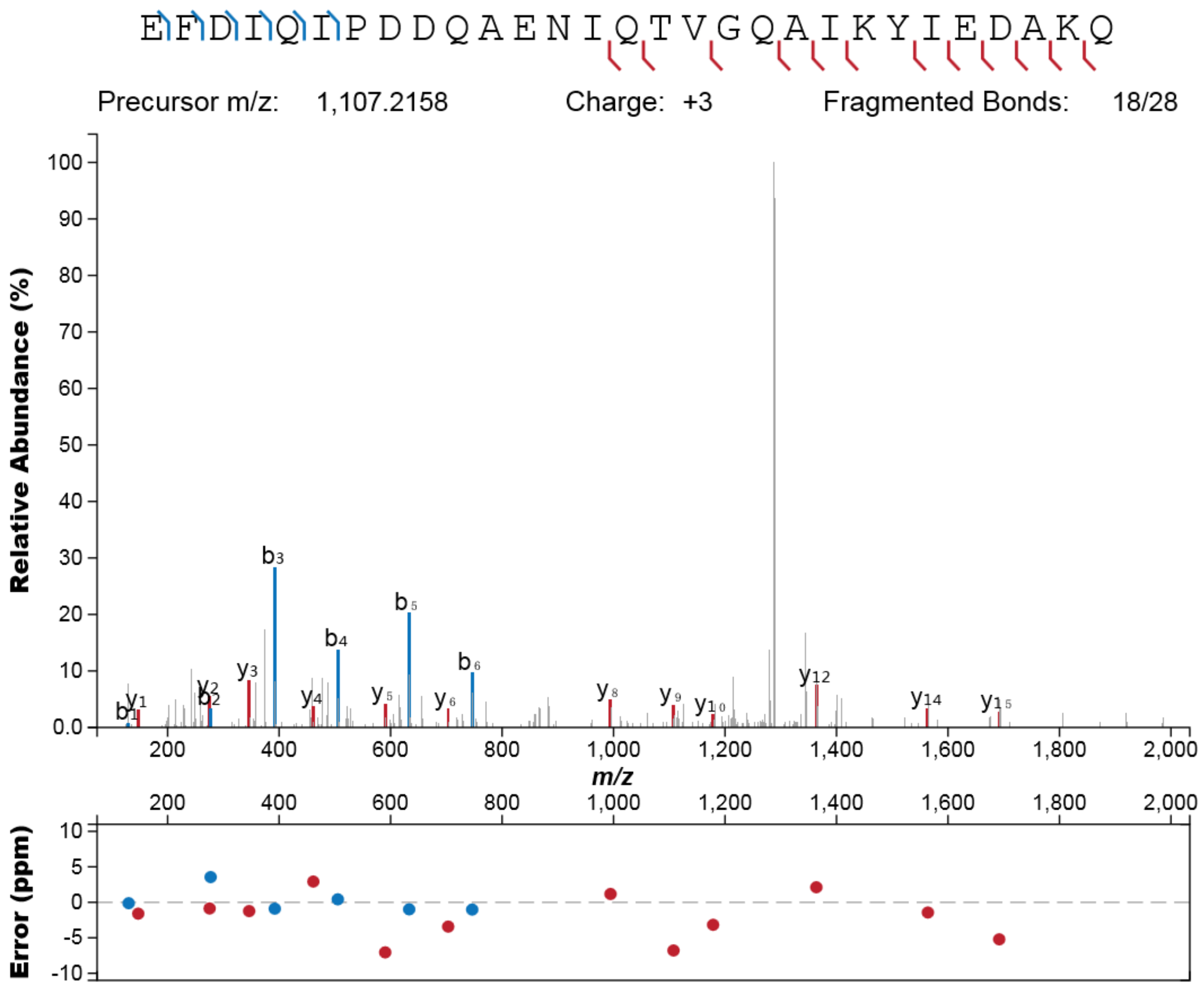

E F D I Q I P D D Q A E N I Q T V G Q A I K Y I E D A K Q
Precursor m/z: 1,107.2158
Charge: +3
Fragmented Bonds: 18/28
Relative Abundance (%)
m/z
Error (ppm)
b1 b2 b3 b4 b5 b6
y1 y2 y3 y4 y5 y6 y8 y9 y10 y12 y14 y15

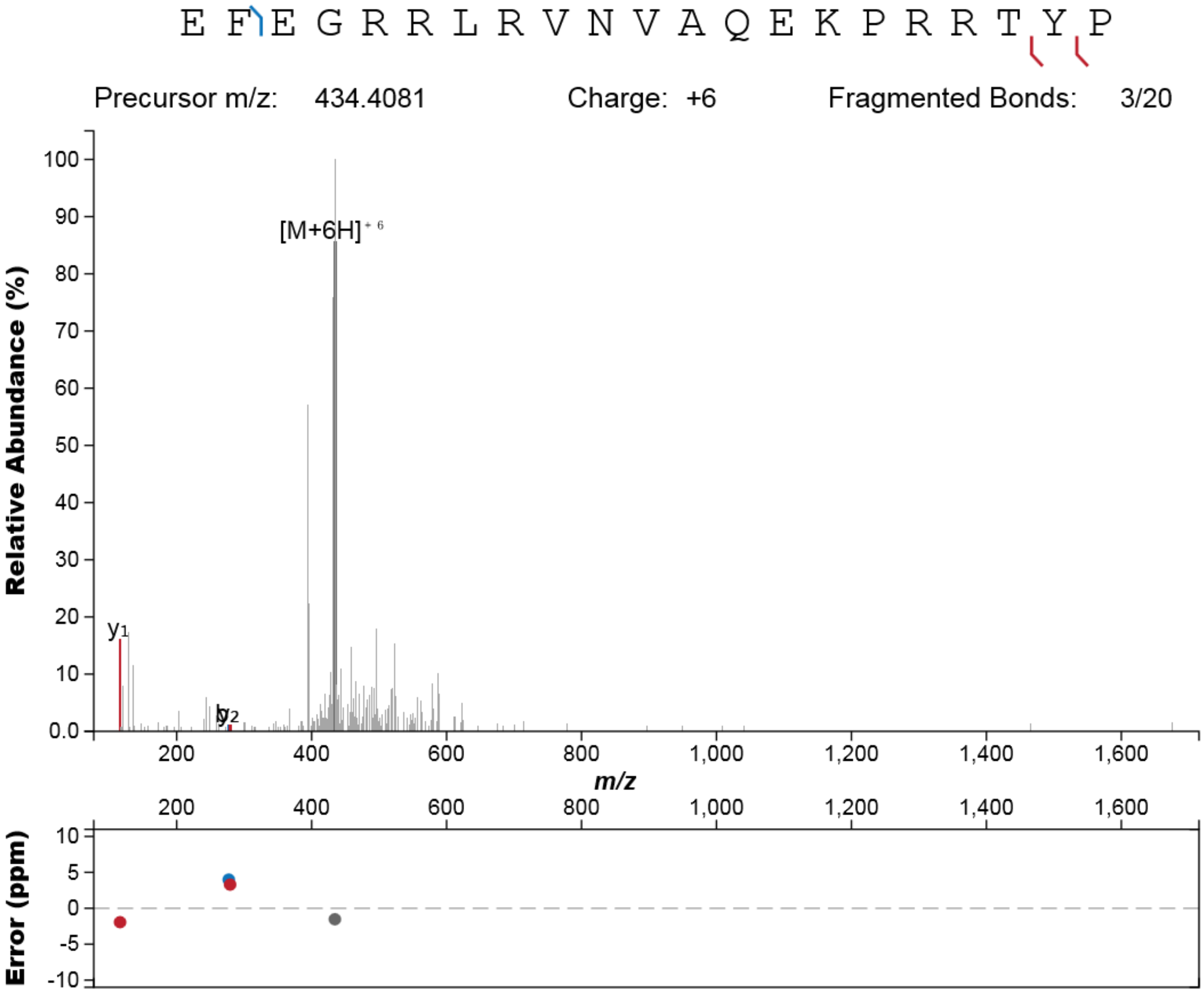

E F E G R R L R V N V A Q E K P R R T Y P
Precursor m/z: 434.4081
Charge: +6
Fragmented Bonds: 3/20
Relative Abundance (%)
[M+6H]+6
y1
b2
y2
m/z
Error (ppm)

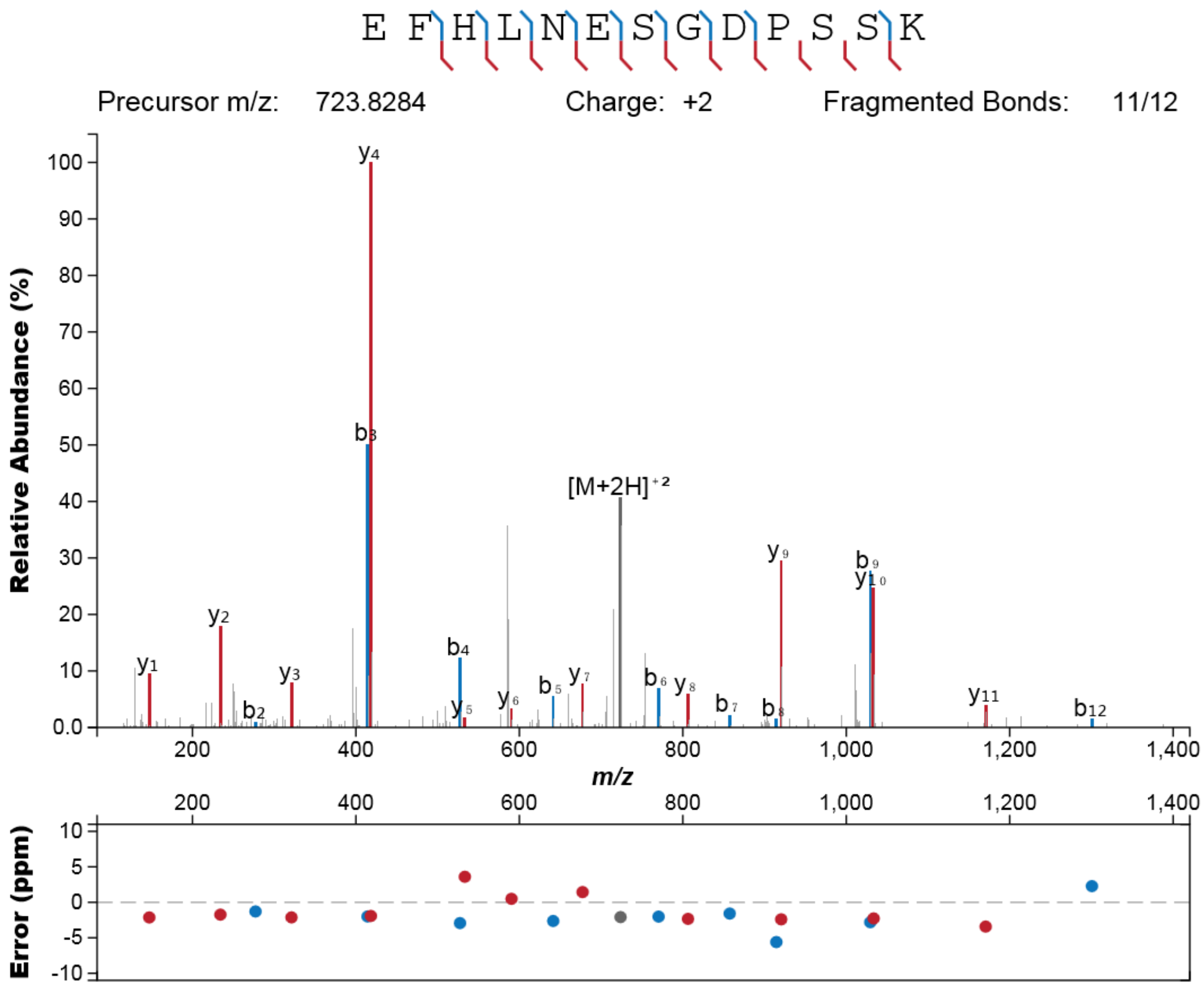

E F H L N E S G D P S S K
Precursor m/z: 723.8284
Charge: +2
Fragmented Bonds: 11/12
Relative Abundance (%)
m/z
Error (ppm)
[M+2H]+2
y1
y2
y3
y4
y5
y6
y7
y8
y9
y10
y11
b2
b3
b4
b5
b6
b7
b8
b9
b12

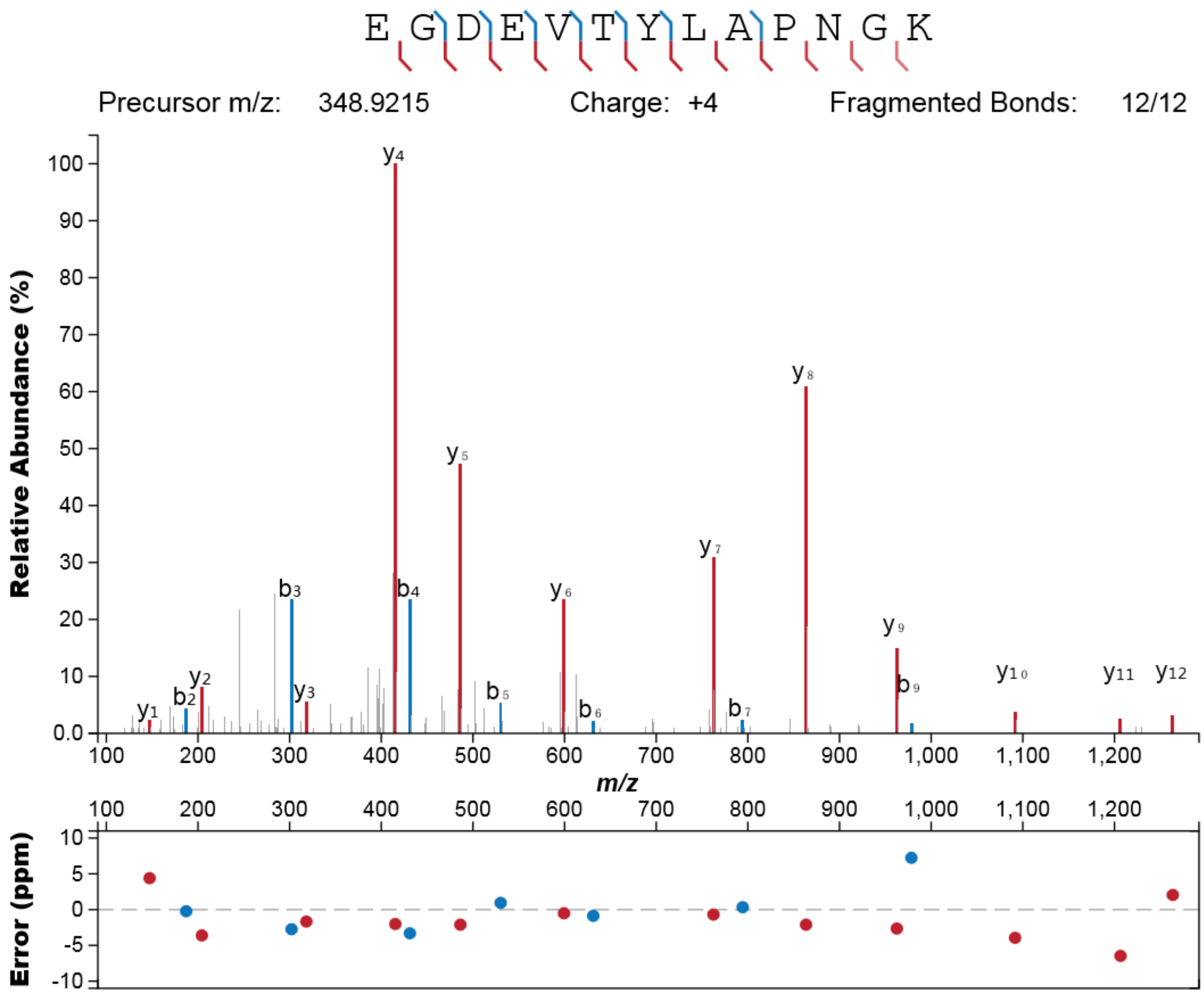
E G D E V T Y L A P N G K
Precursor m/z: 348.9215
Charge: +4
Fragmented Bonds: 12/12
Relative Abundance (%)
m/z
Error (ppm)
y1
b2
y2
b3
y3
y4
b4
y5
b5
y6
b6
y7
b7
y8
y9
b9
y10
y11
y12

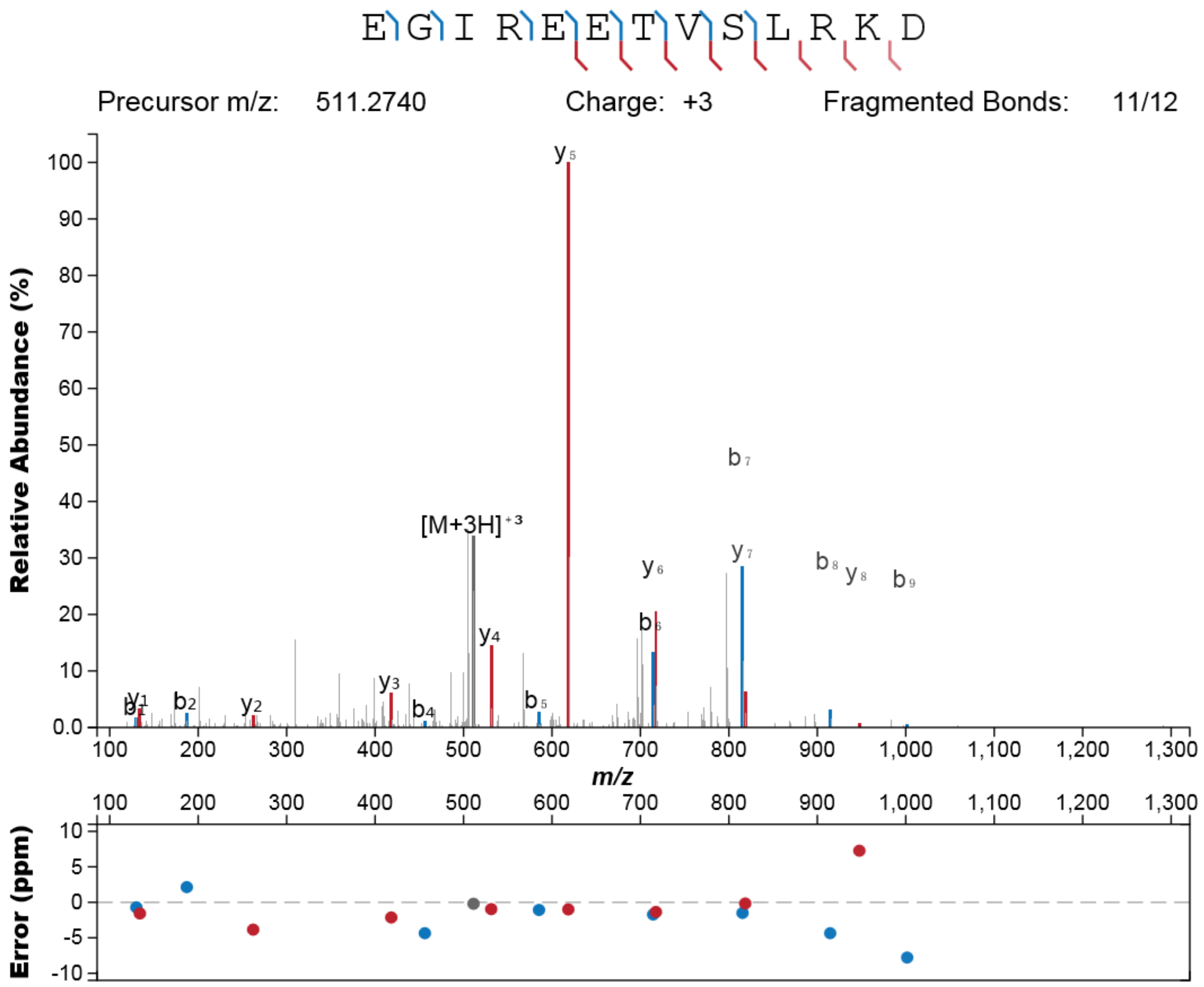

E G I R E E T V S L R K D
Precursor m/z: 511.2740
Charge: +3
Fragmented Bonds: 11/12
Relative Abundance (%)
m/z
Error (ppm)
y5
[M+3H]+3
b7
y7
y6
b8
y8
b9
b6
y4
y3
y1
b2
y2
b4
b5

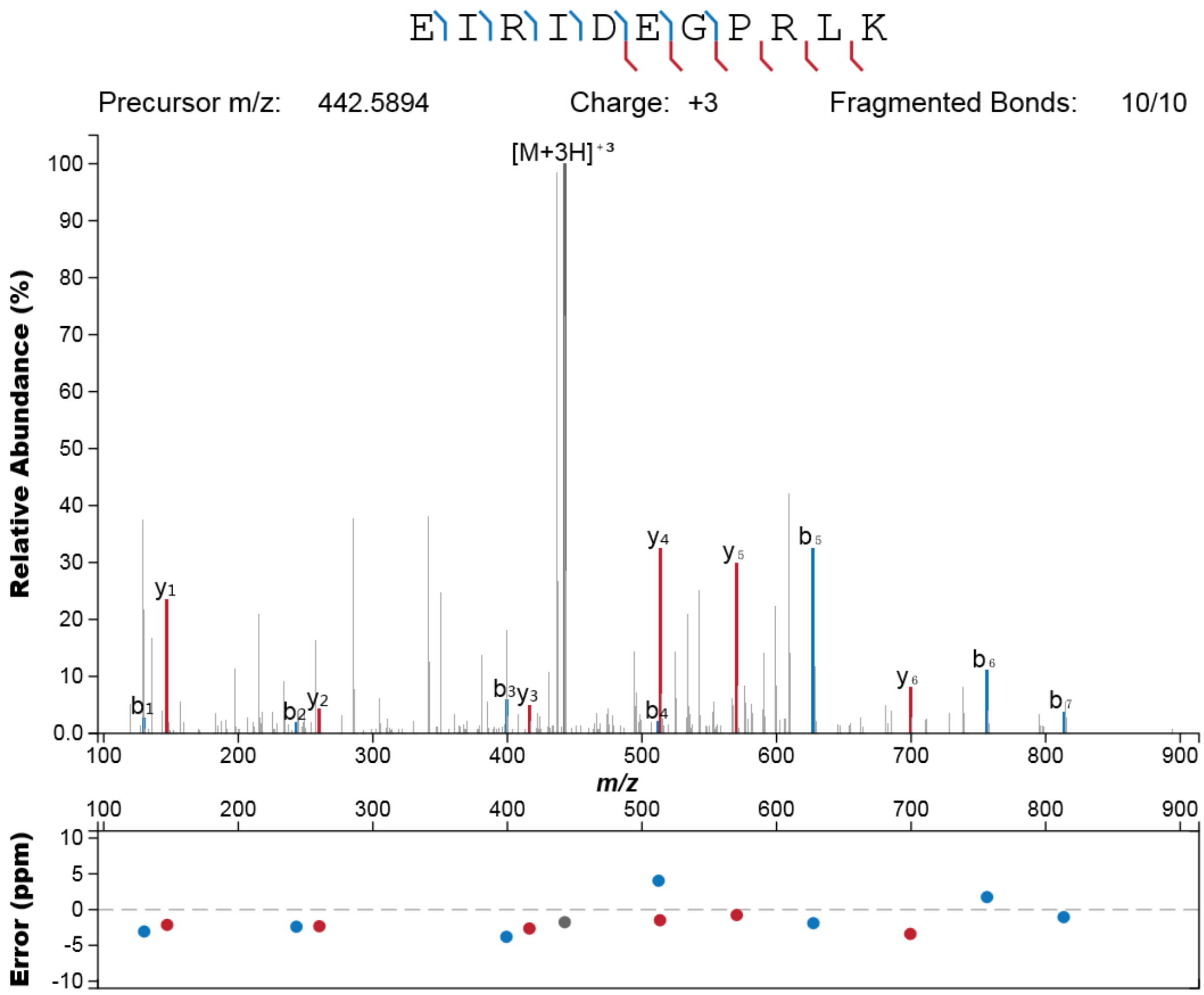

E I R I D E G P R L K
Precursor m/z: 442.5894
Charge: +3
Fragmented Bonds: 10/10
[M+3H]+3
Relative Abundance (%)
m/z
Error (ppm)
y1
y2
y3
y4
y5
y6
b1
b2
b3
b4
b5
b6
b7

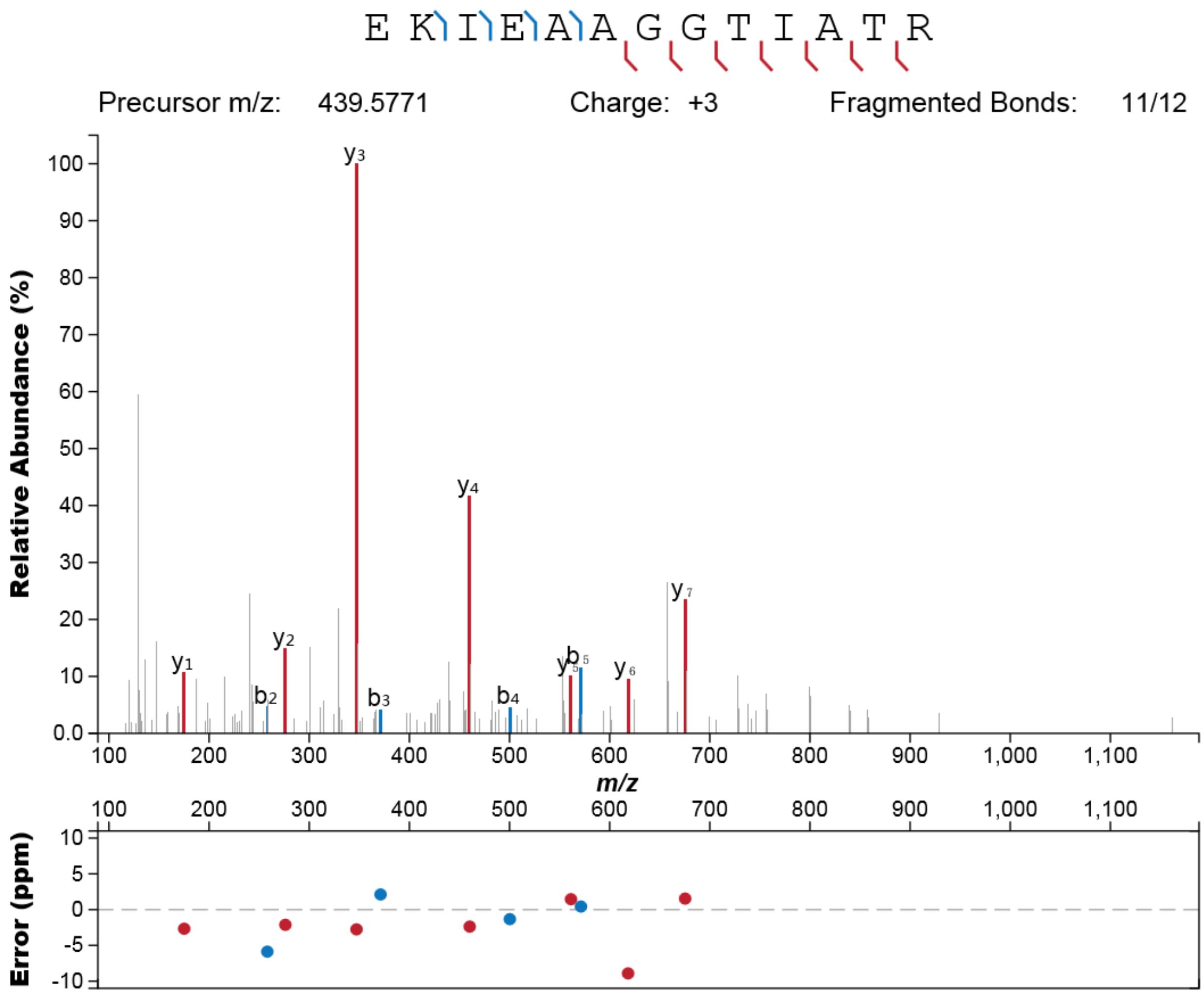
E K I E A A G G T I A T R
Precursor m/z: 439.5771
Charge: +3
Fragmented Bonds: 11/12
Relative Abundance (%)
m/z
Error (ppm)
y3
y4
y7
y1
y2
b2
b3
b4
y5
b5
y6

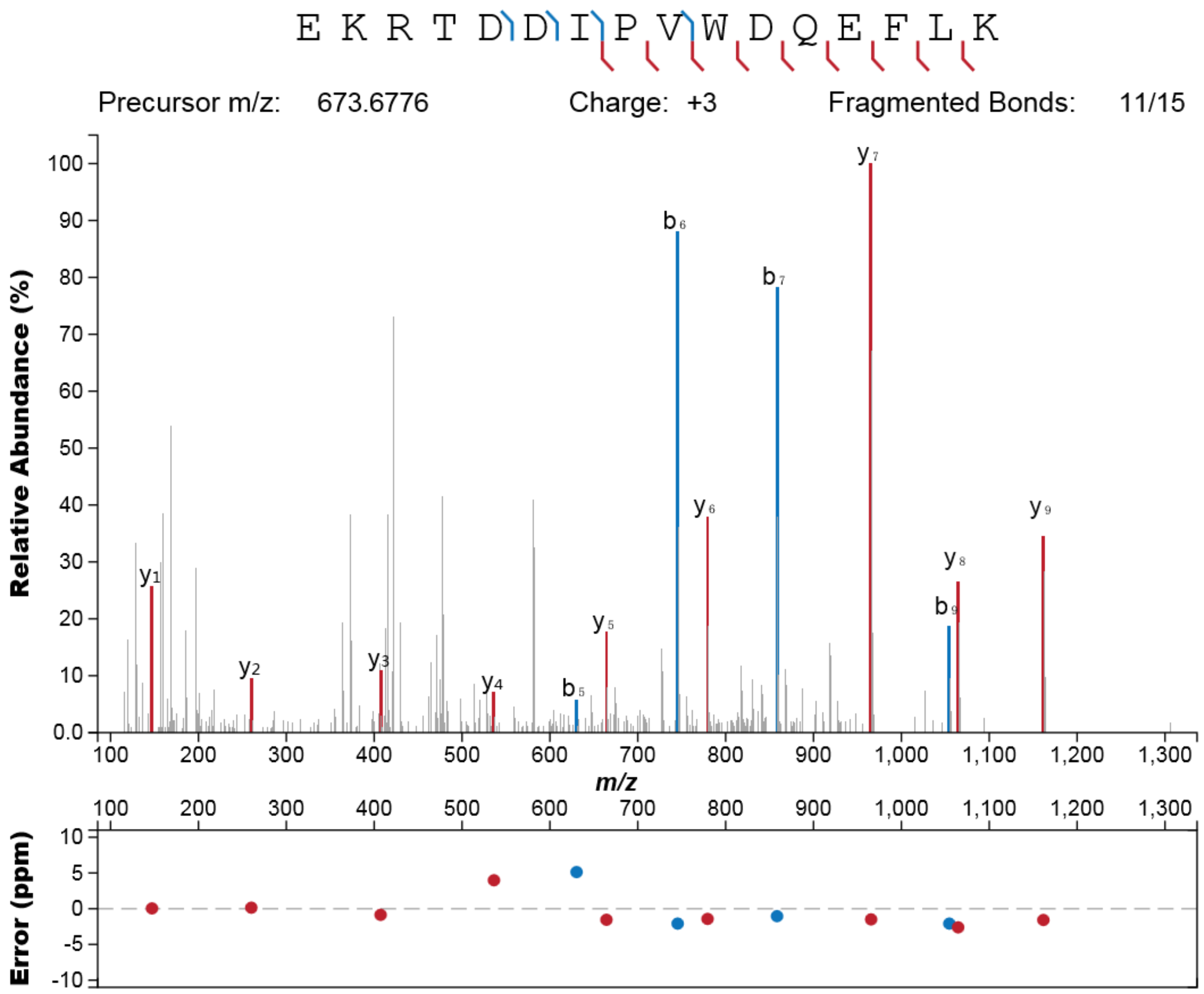
E K R T D D I P V W D Q E F L K
Precursor m/z: 673.6776
Charge: +3
Fragmented Bonds: 11/15
Relative Abundance (%)
m/z
Error (ppm)
y1
y2
y3
y4
b5
y5
b6
y6
b7
y7
b9
y8
y9

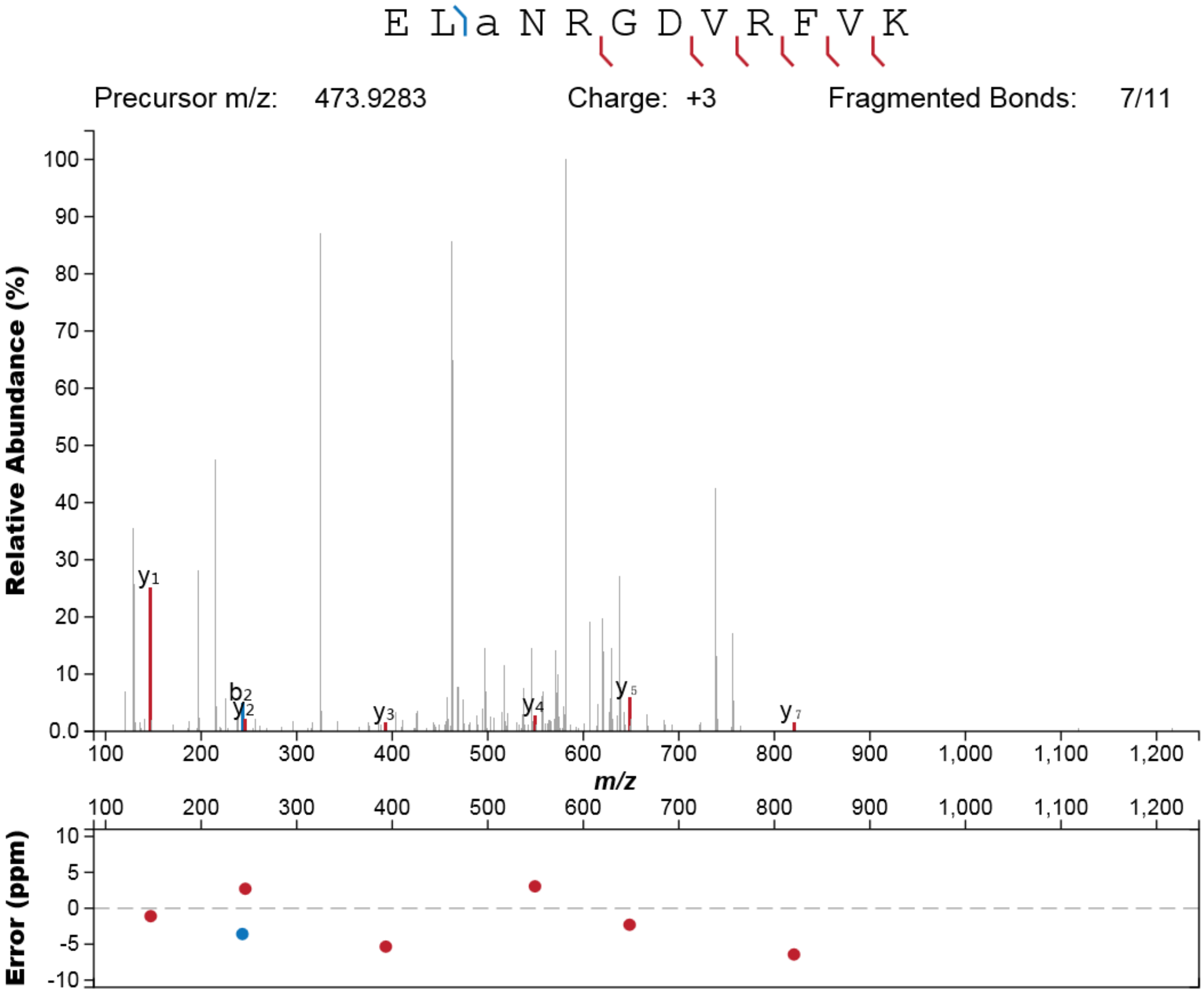

E L a N R G D V R F V K
Precursor m/z: 473.9283
Charge: +3
Fragmented Bonds: 7/11
Relative Abundance (%)
m/z
Error (ppm)
y1
b2
y2
y3
y4
y5
y7

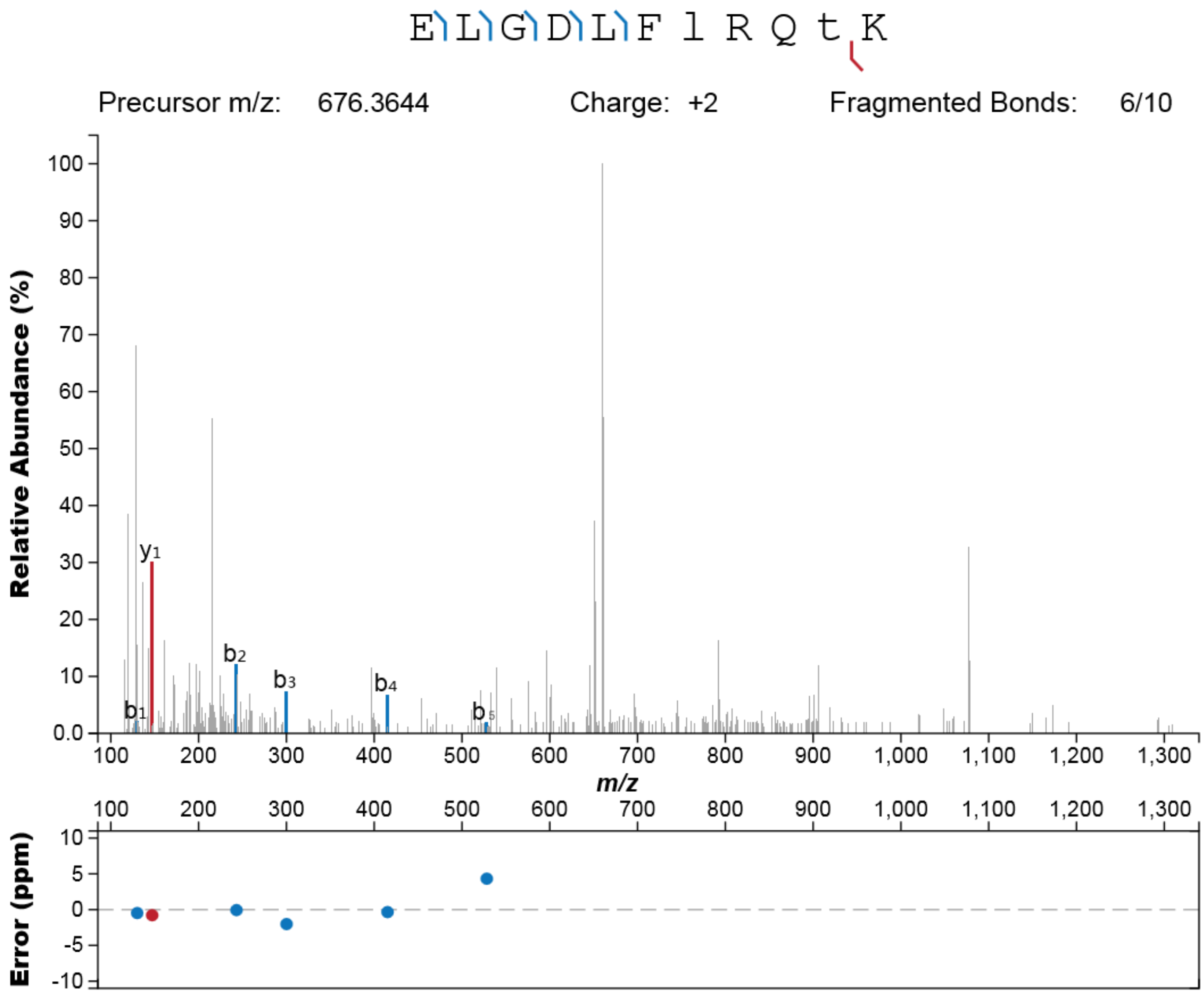

E L G D L F l R Q t K
Precursor m/z: 676.3644
Charge: +2
Fragmented Bonds: 6/10
Relative Abundance (%)
y1
b1
b2
b3
b4
b5
m/z
Error (ppm)

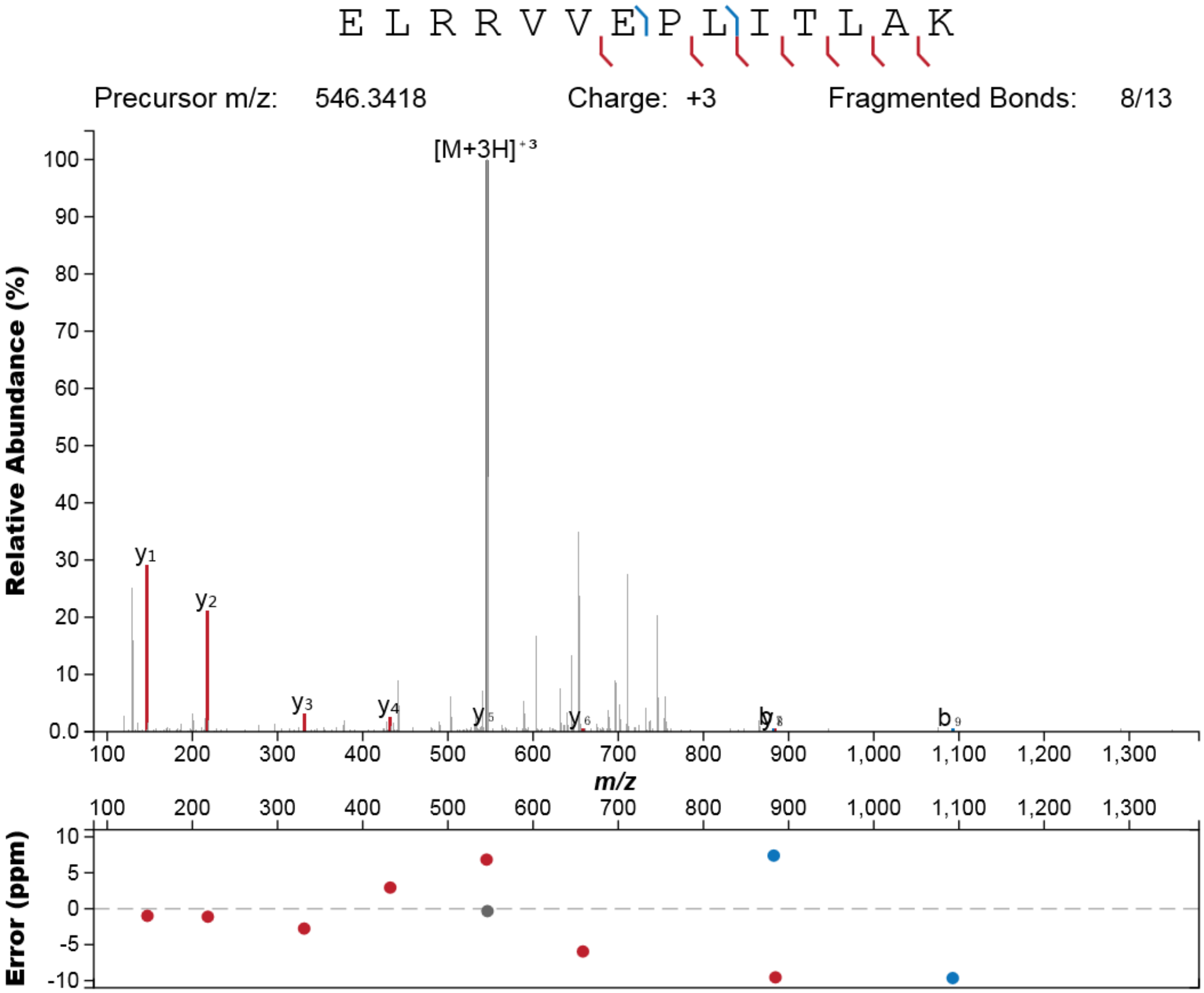

E L R R V V E P L I T L A K
Precursor m/z: 546.3418
Charge: +3
Fragmented Bonds: 8/13
[M+3H]+3
y1
y2
y3
y4
y5
y6
b9
Relative Abundance (%)
m/z
Error (ppm)

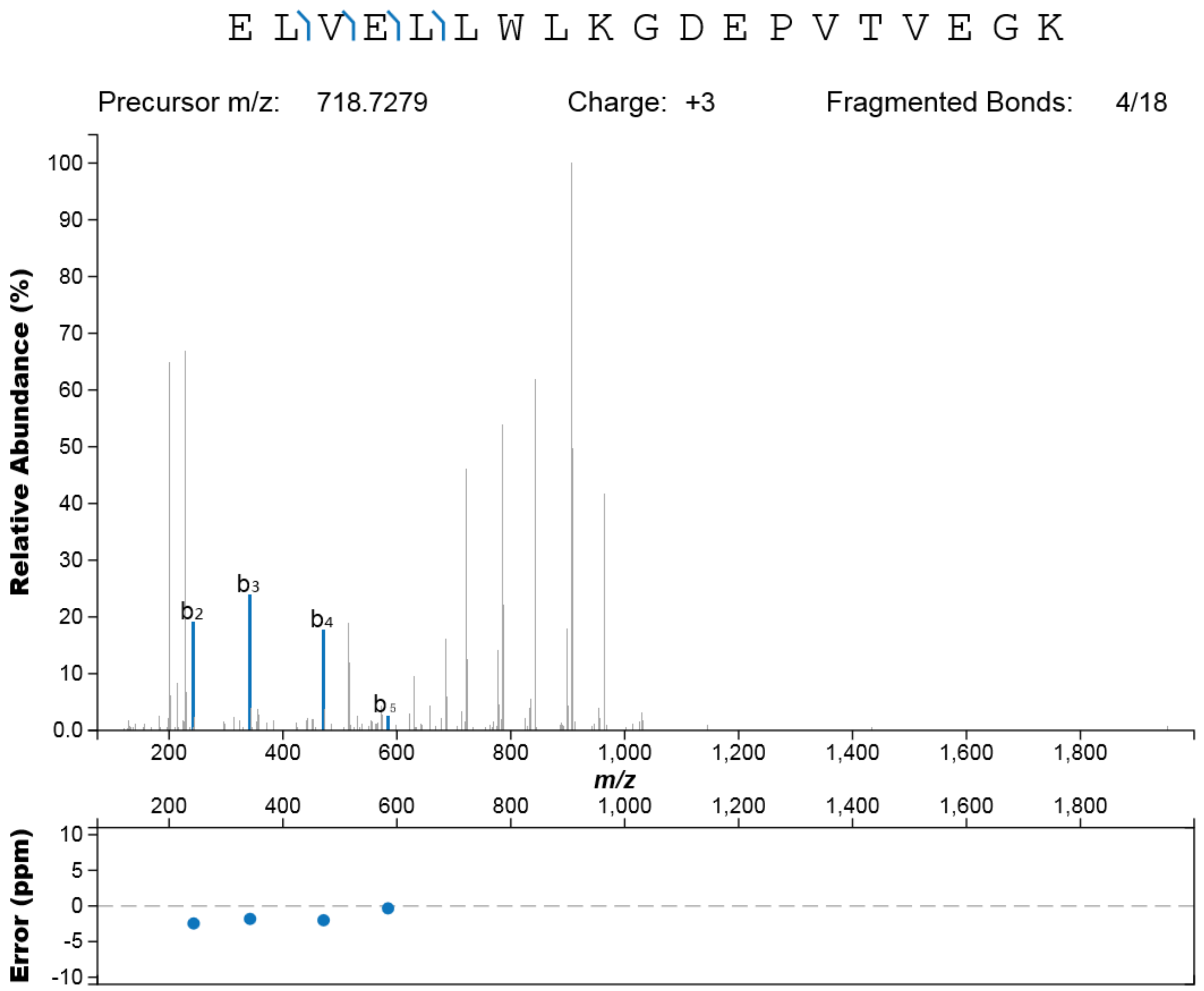

E L V E L L W L K G D E P V T V E G K
Precursor m/z: 718.7279
Charge: +3
Fragmented Bonds: 4/18
Relative Abundance (%)
b2
b3
b4
b5
m/z
Error (ppm)

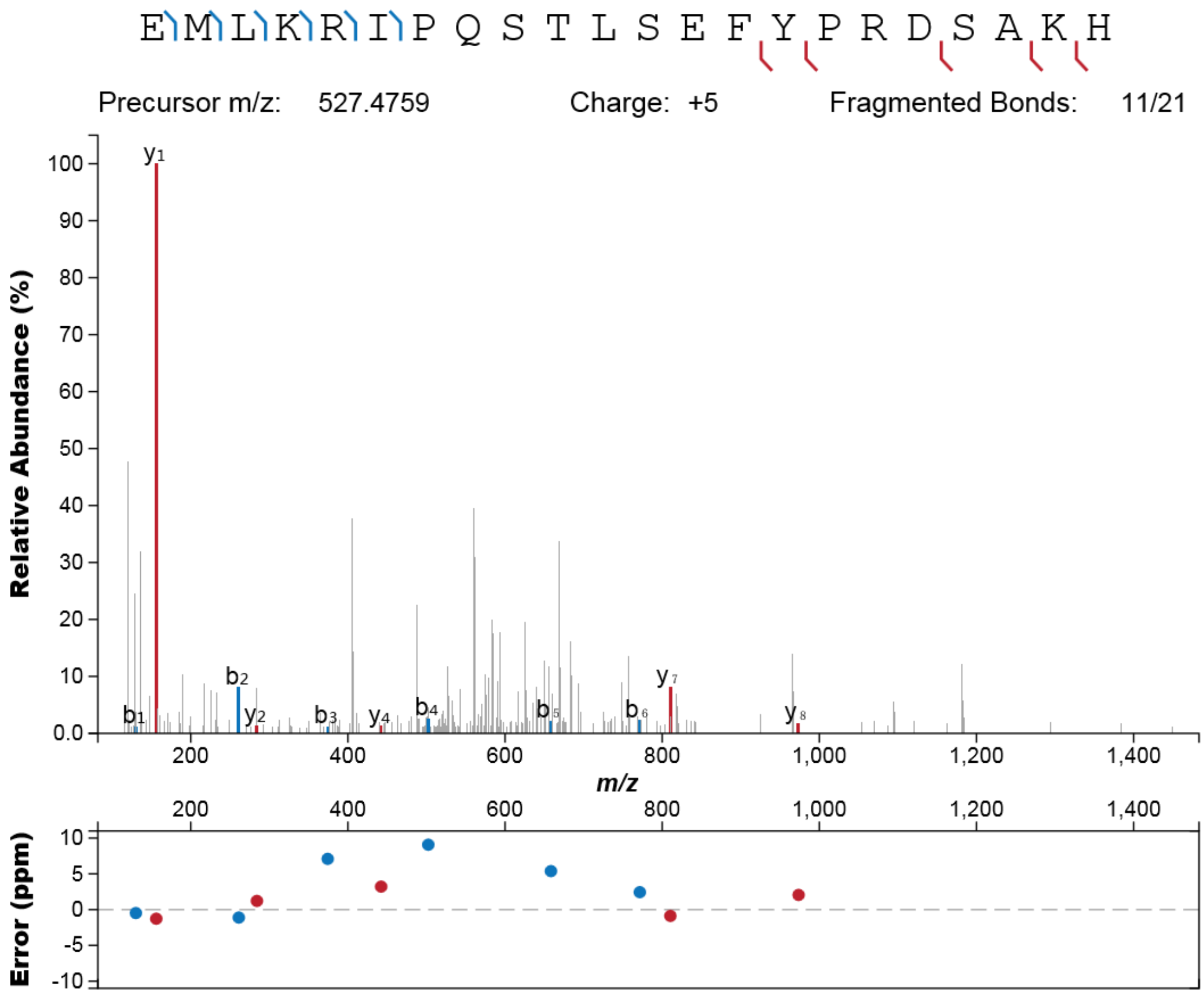
E M L K R I P Q S T L S E F Y P R D S A K H
Precursor m/z: 527.4759
Charge: +5
Fragmented Bonds: 11/21
Relative Abundance (%)
m/z
Error (ppm)
y1
b1
b2
y2
b3
y4
b4
b5
b6
y7
y8

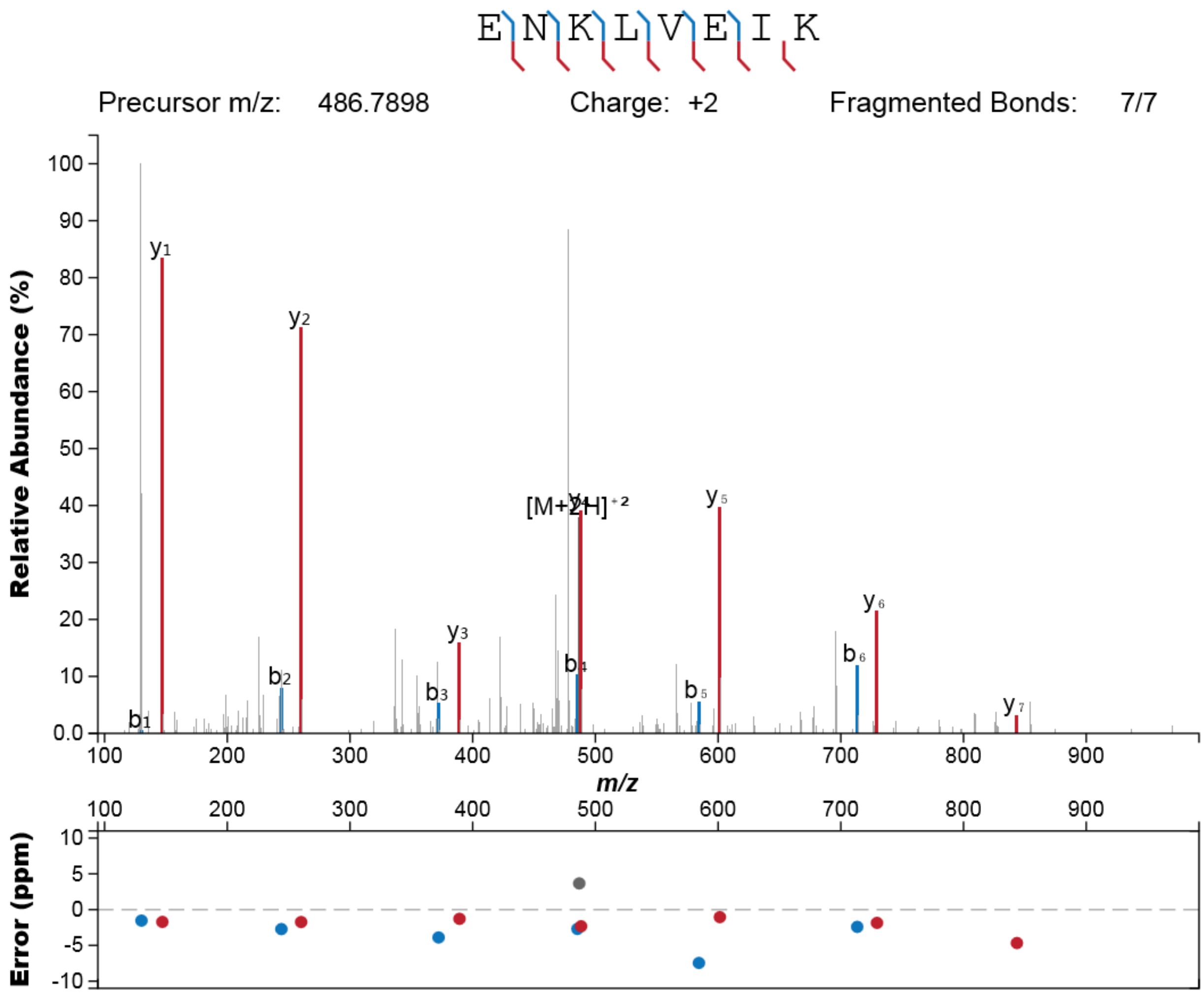

E N K L V E I K
Precursor m/z: 486.7898
Charge: +2
Fragmented Bonds: 7/7
Relative Abundance (%)
m/z
Error (ppm)
y1
y2
y3
y4
y5
y6
y7
b1
b2
b3
b4
b5
b6
[M+2H]+2

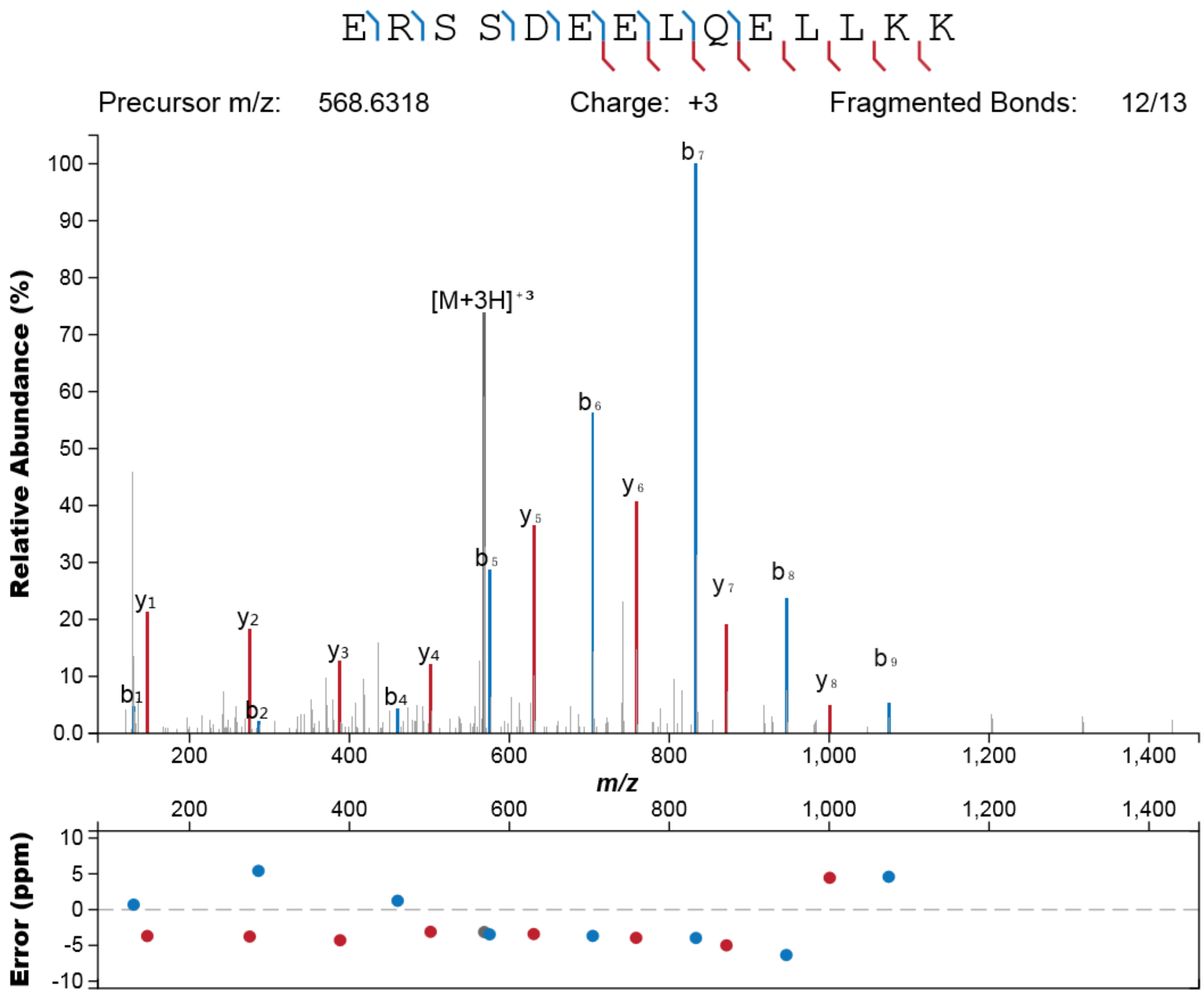
E R S S D E E L Q E L L K K
Precursor m/z: 568.6318
Charge: +3
Fragmented Bonds: 12/13
Relative Abundance (%)
[M+3H]+3
b1
b2
b4
b5
b6
b7
b8
b9
y1
y2
y3
y4
y5
y6
y7
y8
m/z
Error (ppm)

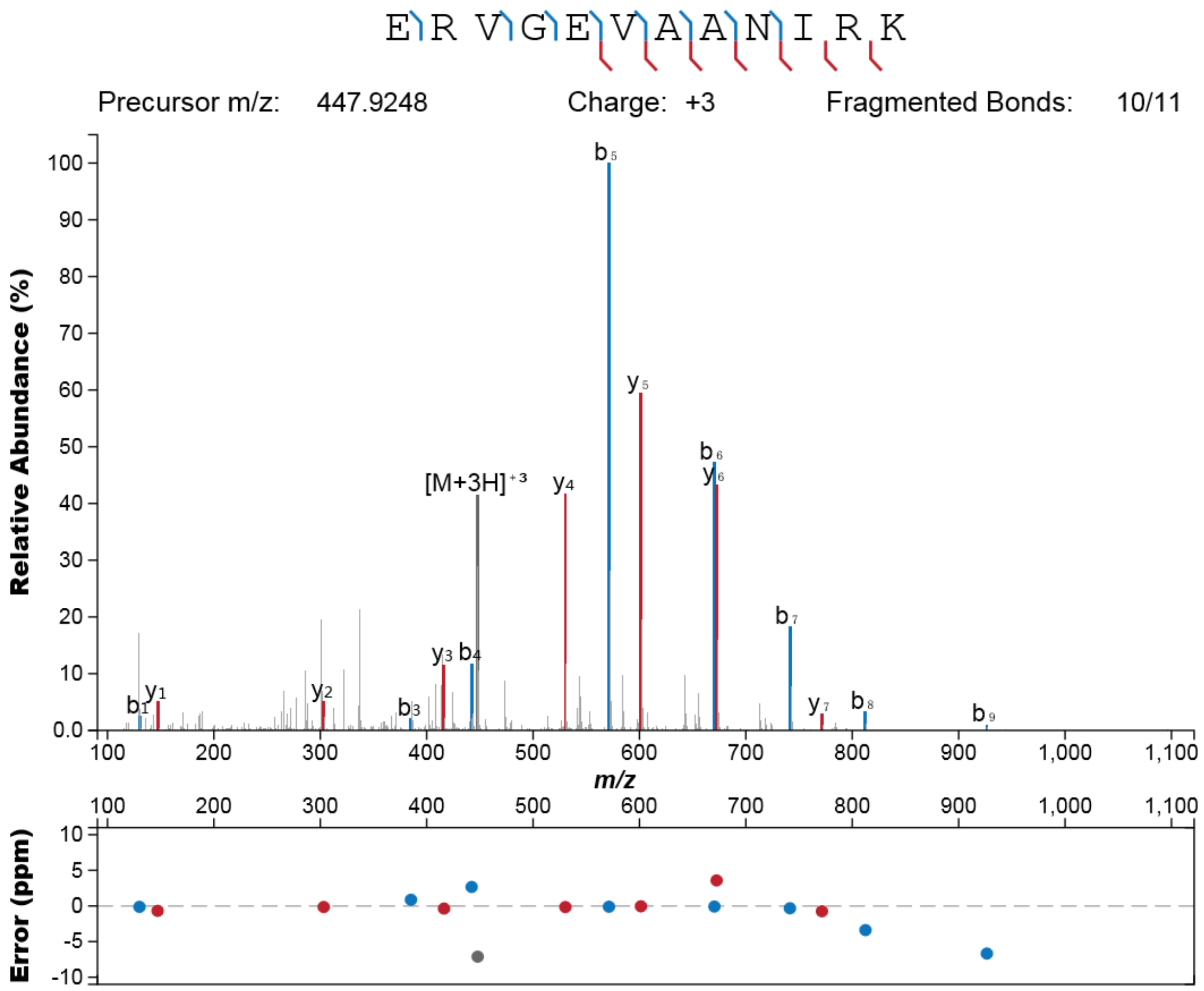
E R V G E V A A N I R K
Precursor m/z: 447.9248
Charge: +3
Fragmented Bonds: 10/11
Relative Abundance (%)
m/z
Error (ppm)
[M+3H]+3
b1 y1 y2 b3 y3 b4 y4 b5 y5 b6 y6 b7 y7 b8 b9

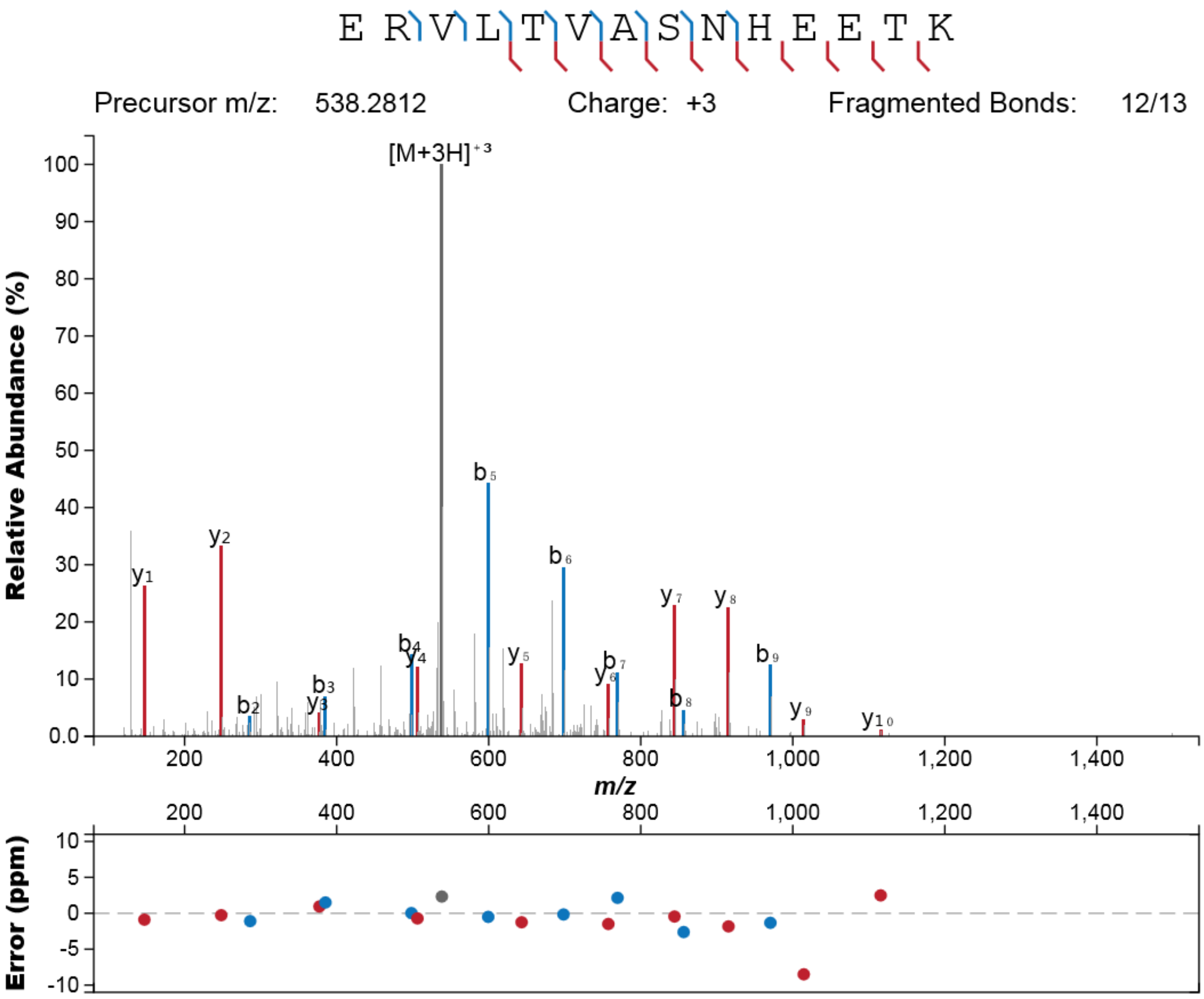

E R V L T V A S N H E E T K
Precursor m/z: 538.2812
Charge: +3
Fragmented Bonds: 12/13
[M+3H]+3
Relative Abundance (%)
m/z
Error (ppm)
y1
y2
b2
b3
y3
b4
y4
b5
b6
y5
b7
y6
y7
y8
b8
b9
y9
y10

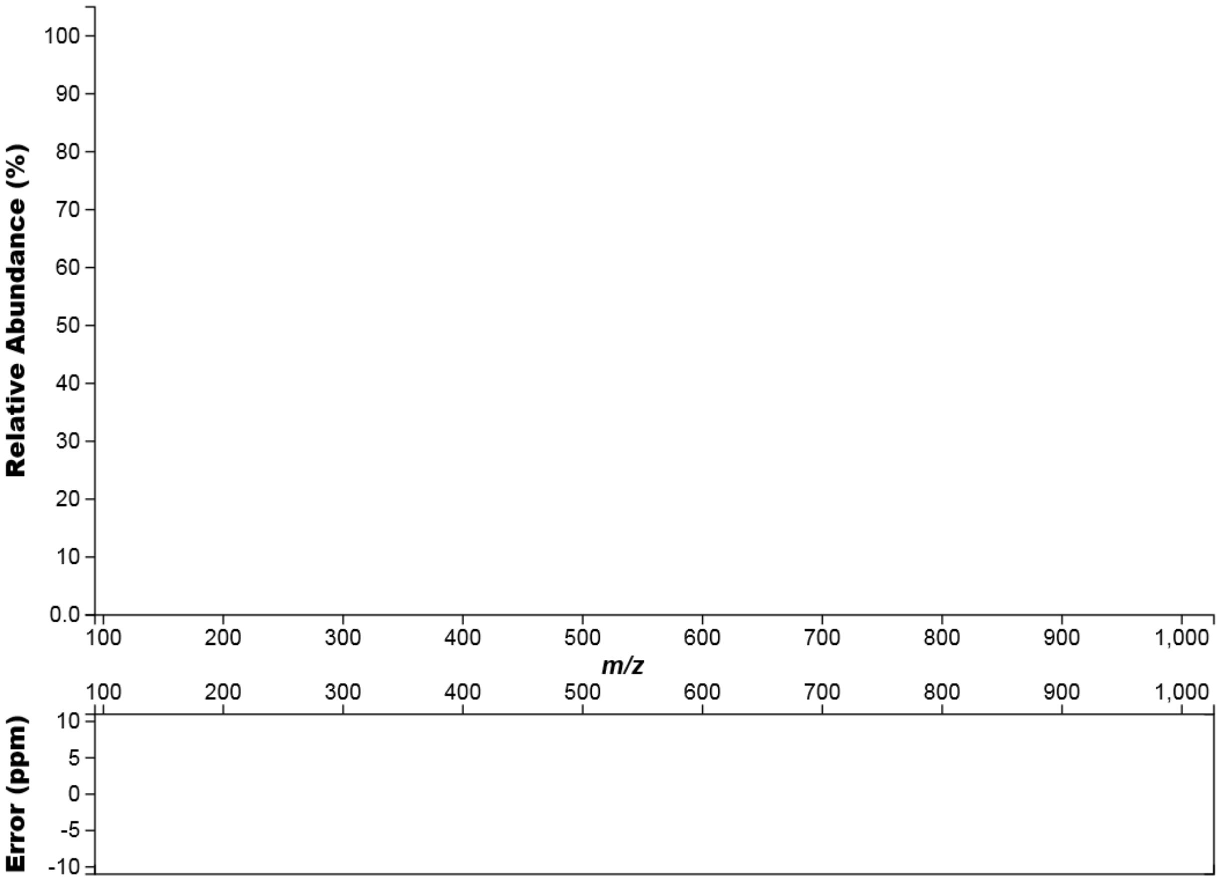

Relative Abundance (%)
100
90
80
70
60
50
40
30
20
10
0.0
100
200
300
400
500
600
700
800
900
1,000
m/z
Error (ppm)
10
5
0
-5
-10

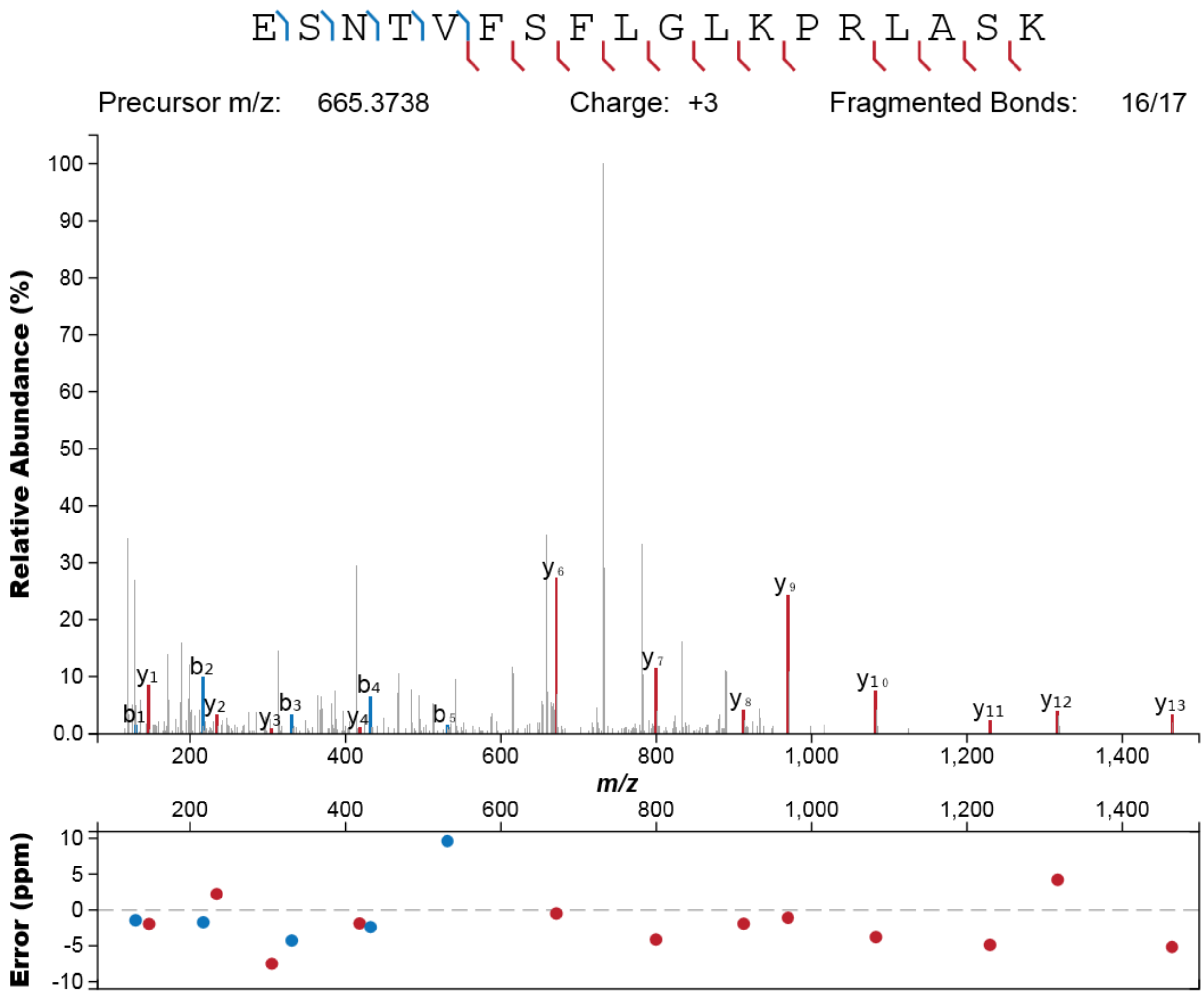
E S N T V F S F L G L K P R L A S K
Precursor m/z: 665.3738
Charge: +3
Fragmented Bonds: 16/17
Relative Abundance (%)
m/z
Error (ppm)

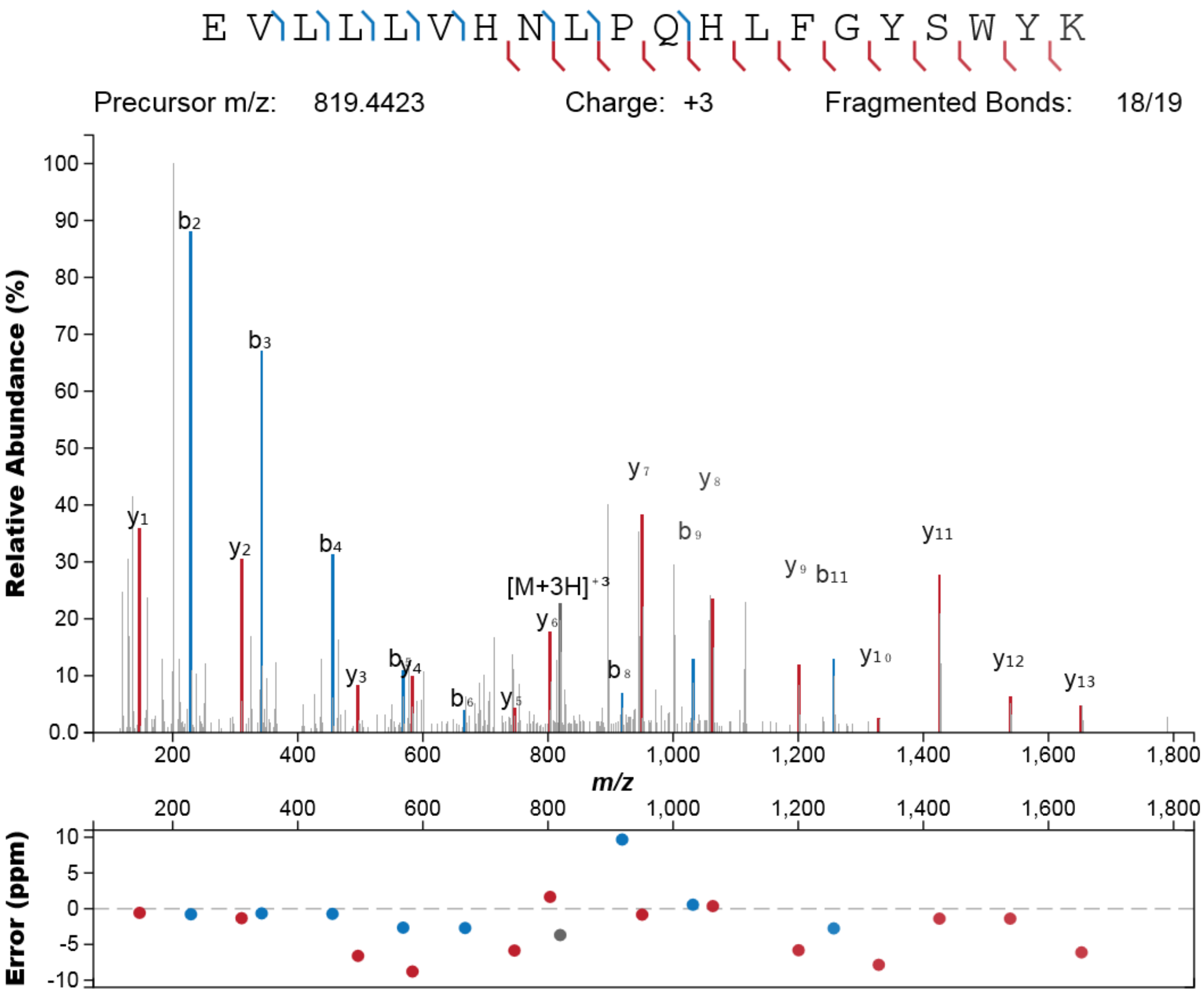
E V L L L V H N L P Q H L F G Y S W Y K
Precursor m/z: 819.4423
Charge: +3
Fragmented Bonds: 18/19
Relative Abundance (%)
m/z
Error (ppm)
[M+3H]+3

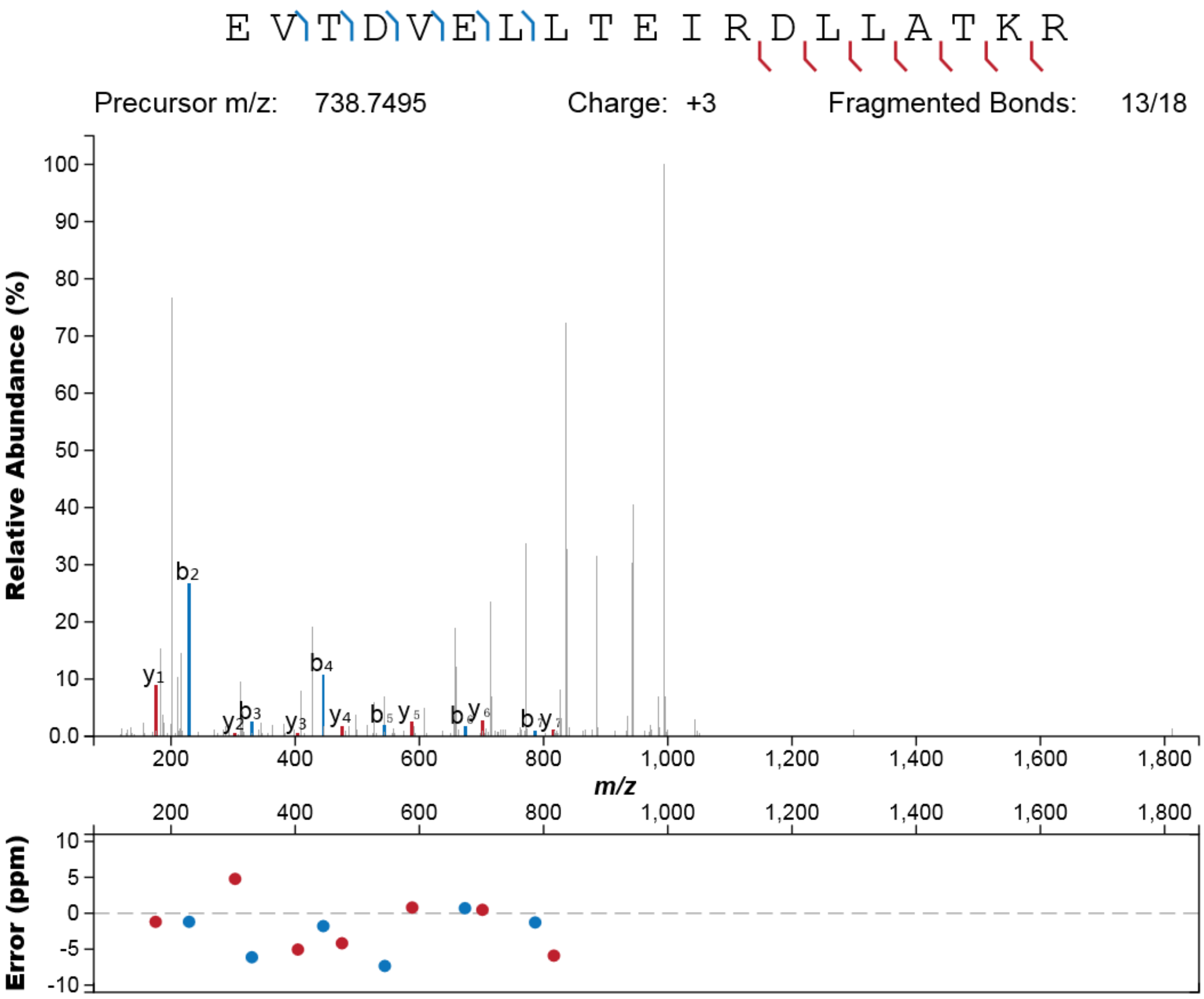
E V T D V E L L T E I R D L L A T K R
Precursor m/z: 738.7495
Charge: +3
Fragmented Bonds: 13/18
Relative Abundance (%)
m/z
Error (ppm)

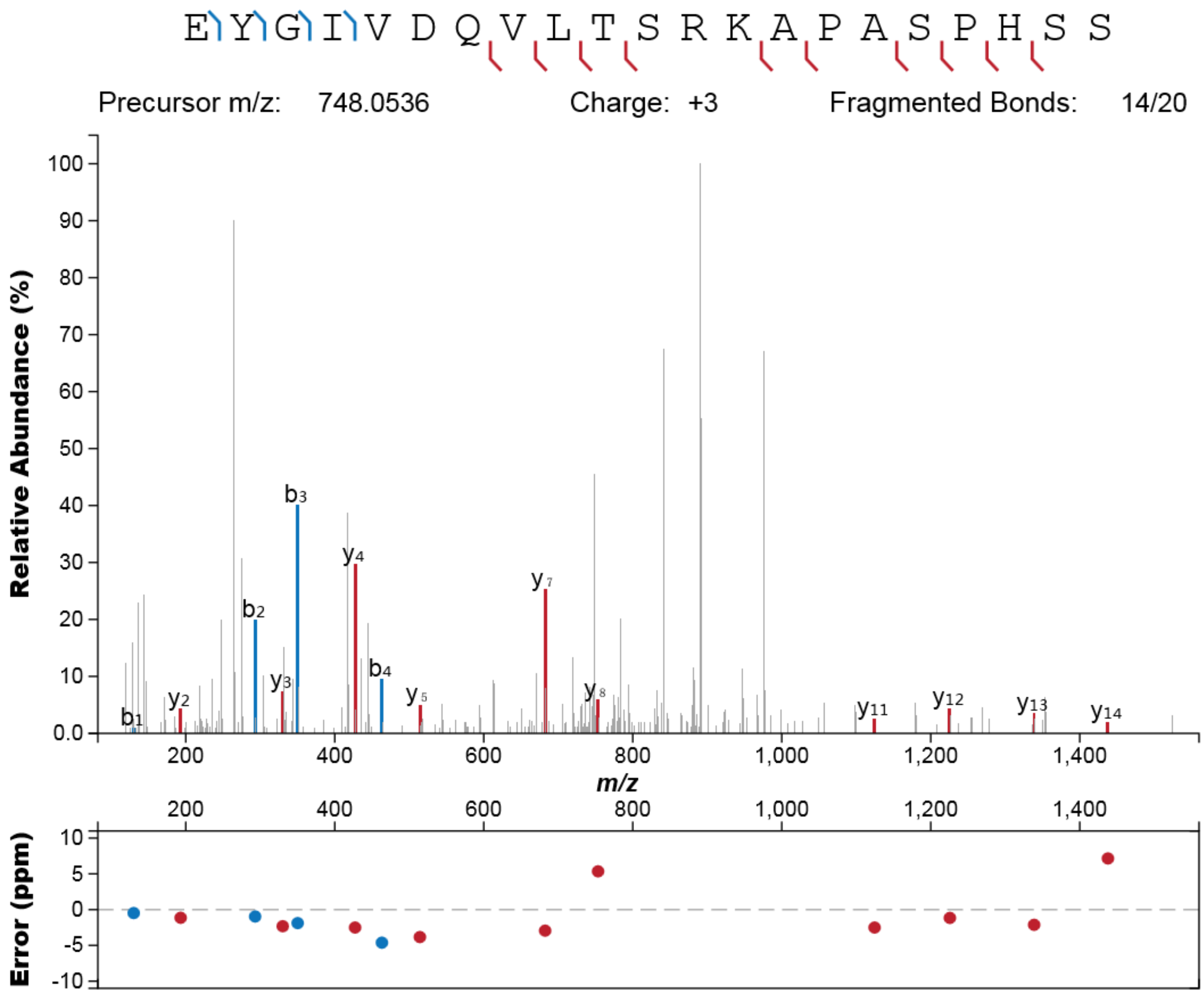

E Y G I V D Q V L T S R K A P A S P H S S
Precursor m/z: 748.0536
Charge: +3
Fragmented Bonds: 14/20
Relative Abundance (%)
b1
y2
b2
y3
b3
y4
b4
y5
y7
y8
y11
y12
y13
y14
m/z
Error (ppm)

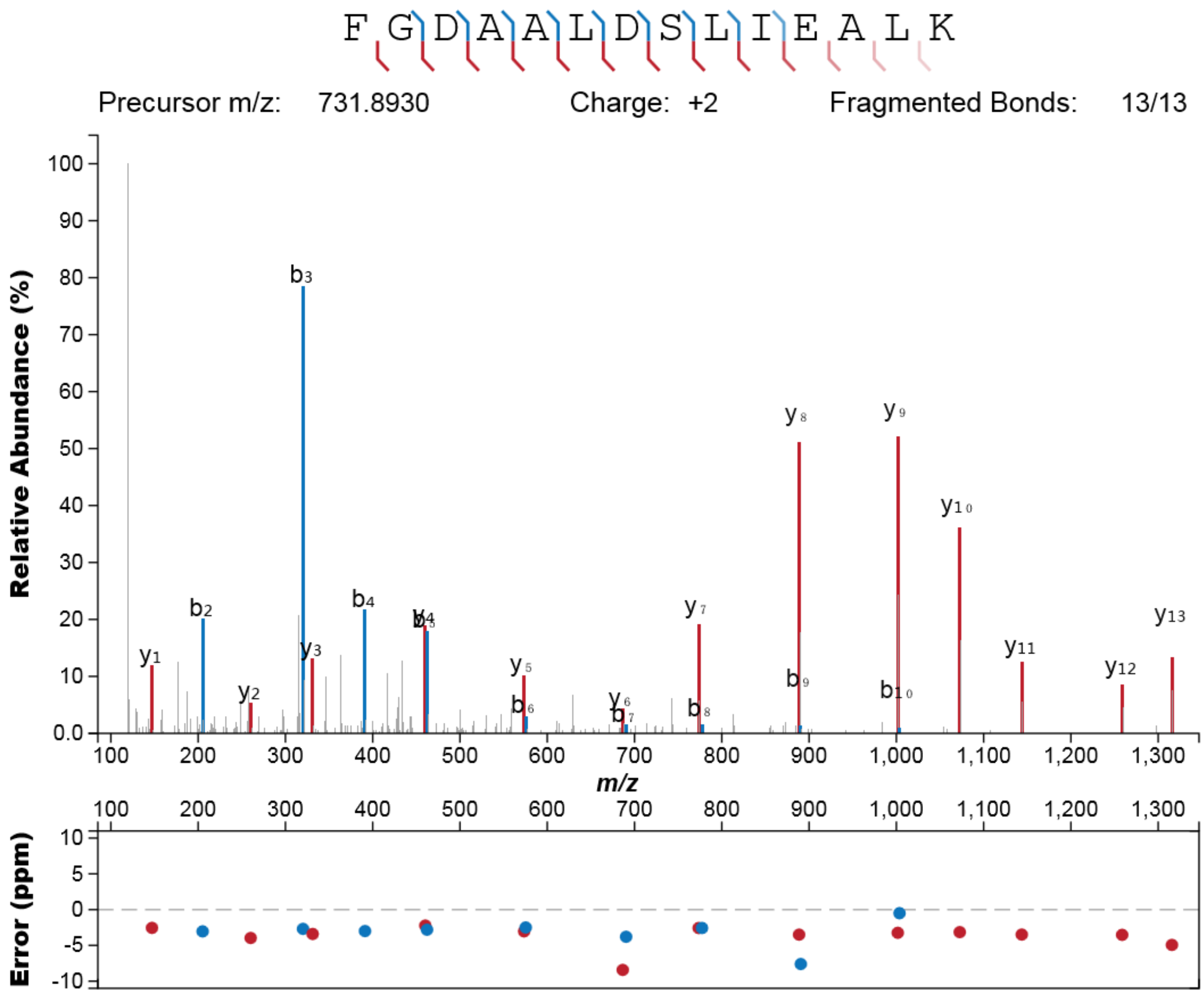
F G D A A L D S L I E A L K
Precursor m/z: 731.8930
Charge: +2
Fragmented Bonds: 13/13
Relative Abundance (%)
m/z
Error (ppm)
b2
b3
b4
b6
b7
b8
b9
b10
y1
y2
y3
y4
y5
y6
y7
y8
y9
y10
y11
y12
y13

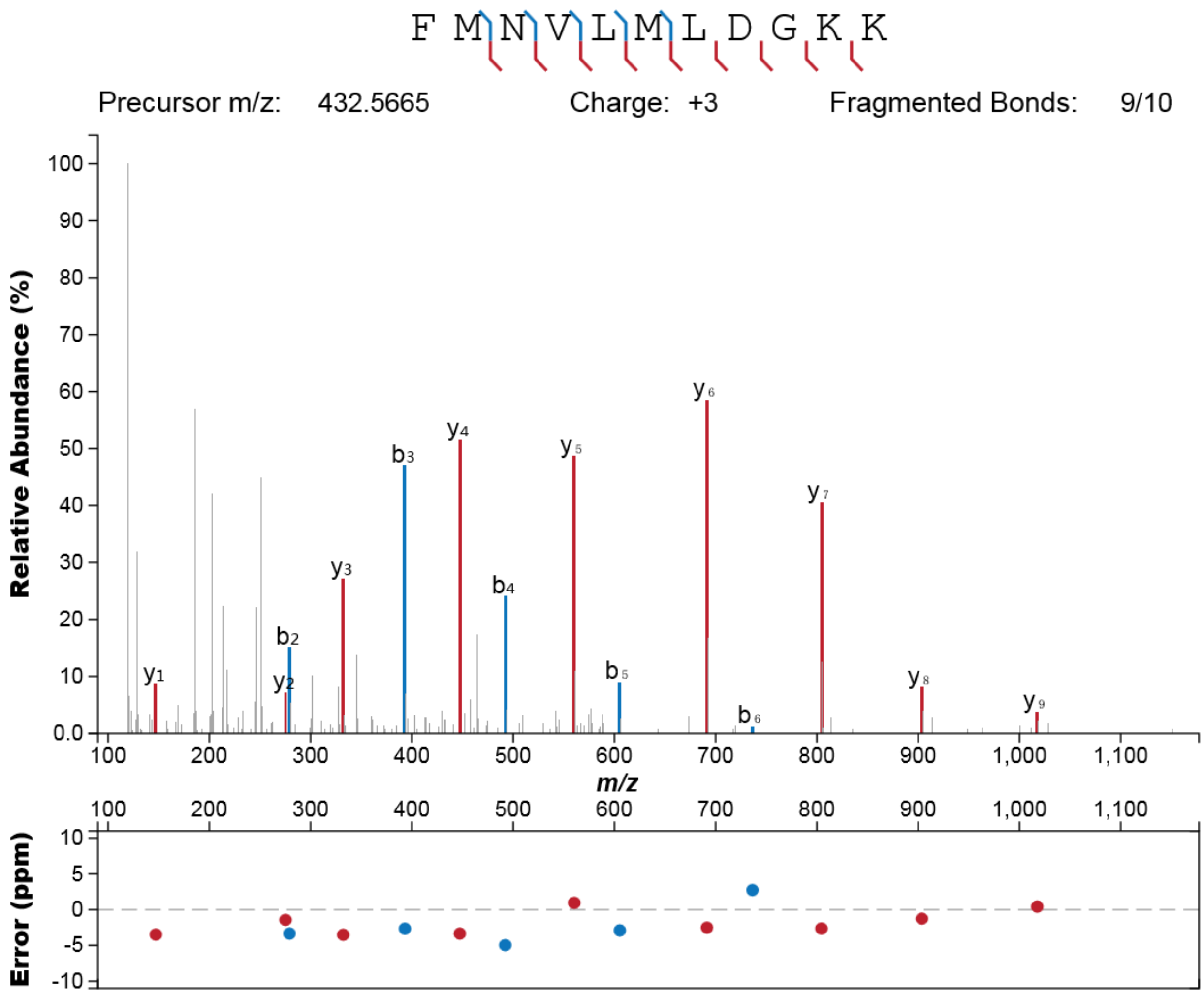
F M N V L M L D G K K
Precursor m/z: 432.5665
Charge: +3
Fragmented Bonds: 9/10
Relative Abundance (%)
m/z
Error (ppm)
y1
y2
b2
y3
b3
y4
b4
y5
b5
y6
b6
y7
y8
y9

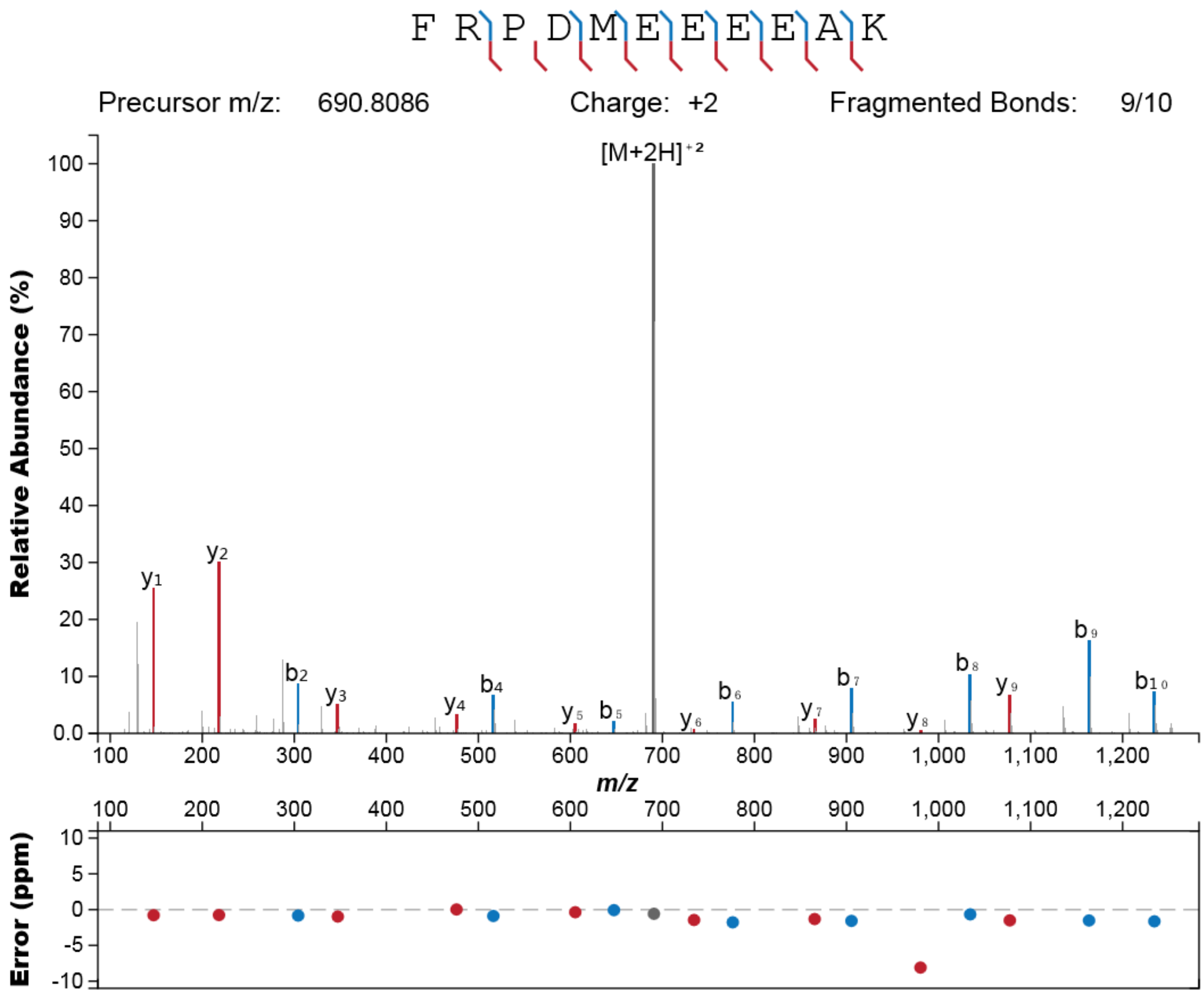

F R P D M E E E E A K
Precursor m/z: 690.8086
Charge: +2
Fragmented Bonds: 9/10
[M+2H]+2
Relative Abundance (%)
m/z
Error (ppm)
y1
y2
b2
y3
y4
b4
y5
b5
y6
b6
y7
b7
y8
b8
y9
b9
b10

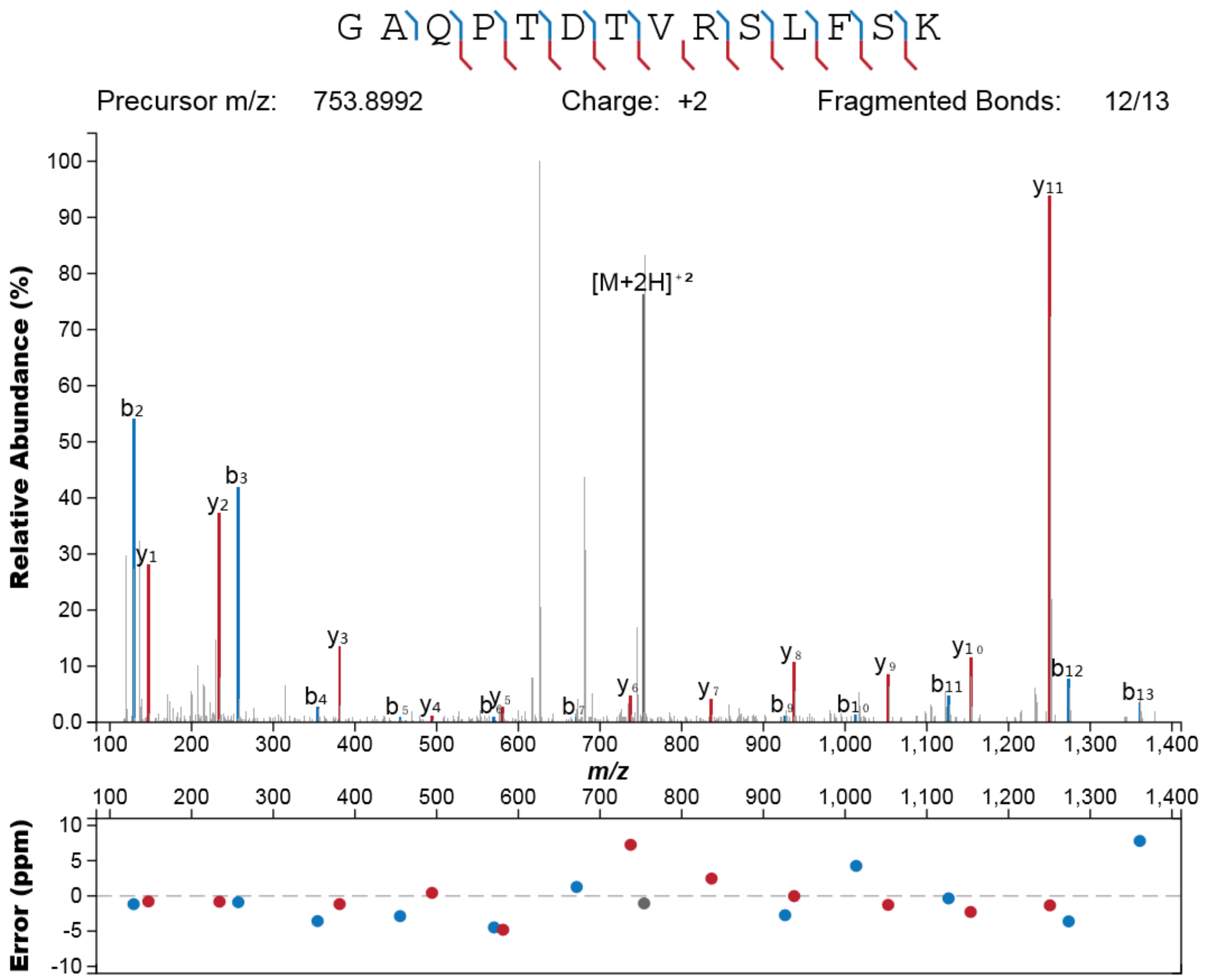

G A Q P T D T V R S L F S K
Precursor m/z: 753.8992
Charge: +2
Fragmented Bonds: 12/13
Relative Abundance (%)
[M+2H]+2
m/z
Error (ppm)

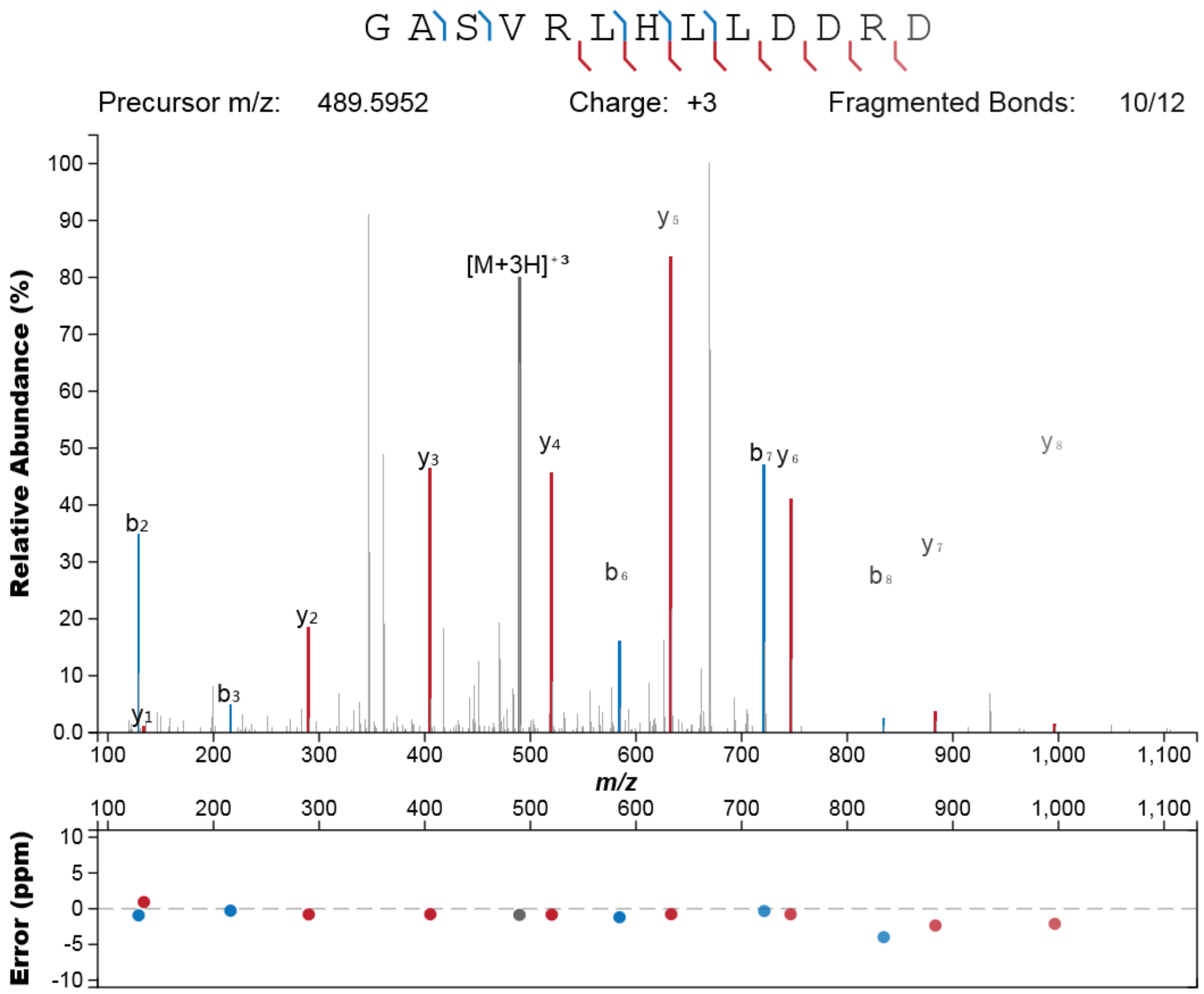

G A S V R L H L L D D R D
Precursor m/z: 489.5952
Charge: +3
Fragmented Bonds: 10/12
Relative Abundance (%)
[M+3H]+3
y5
y4
y3
b7 y6
y8
b2
y7
b6
b8
y2
b3
y1
m/z
Error (ppm)

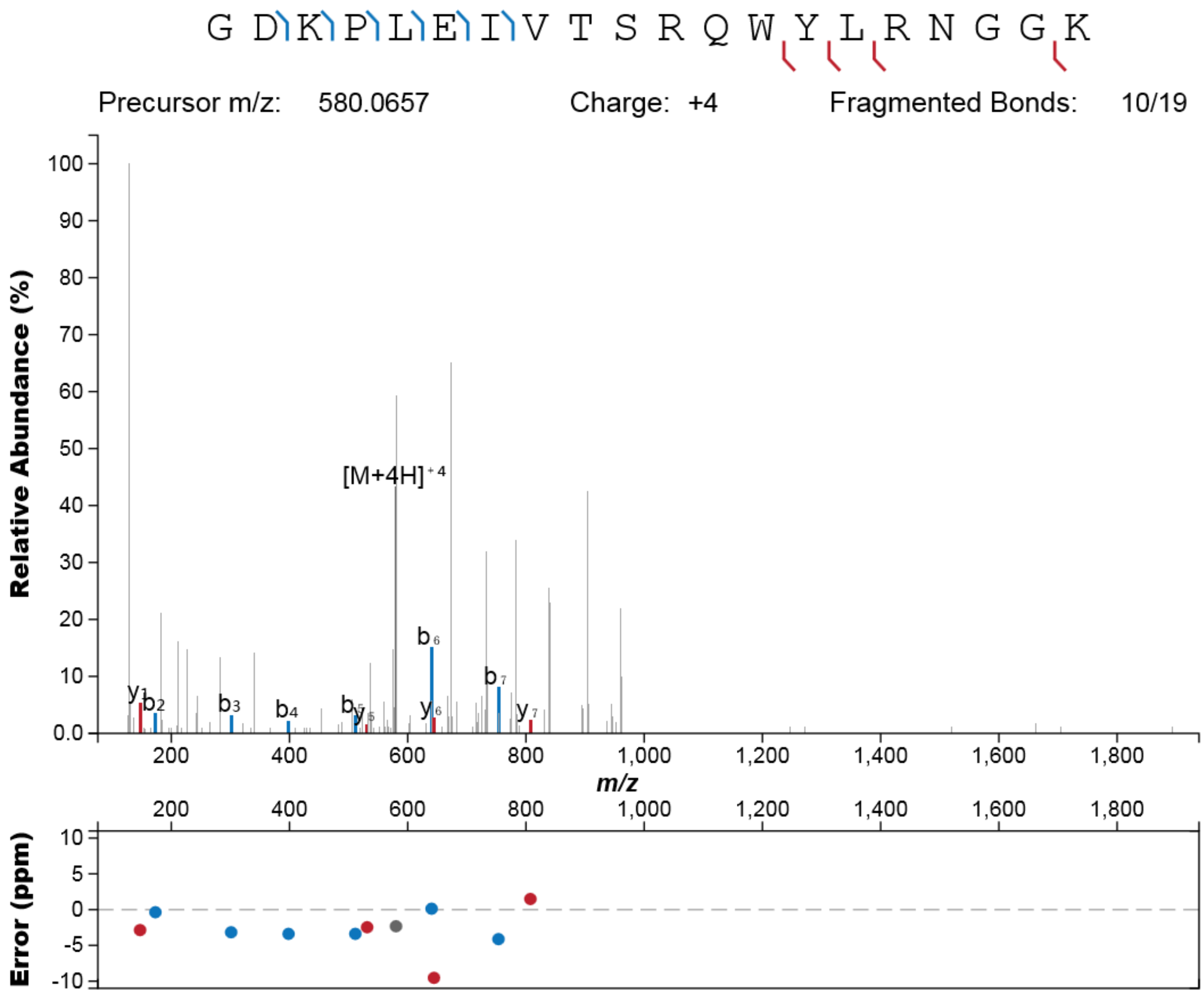

G D K P L E I V T S R Q W Y L R N G G K
Precursor m/z: 580.0657
Charge: +4
Fragmented Bonds: 10/19
Relative Abundance (%)
[M+4H]+4
b2
b3
b4
b5
b6
b7
y1
y5
y6
y7
m/z
Error (ppm)

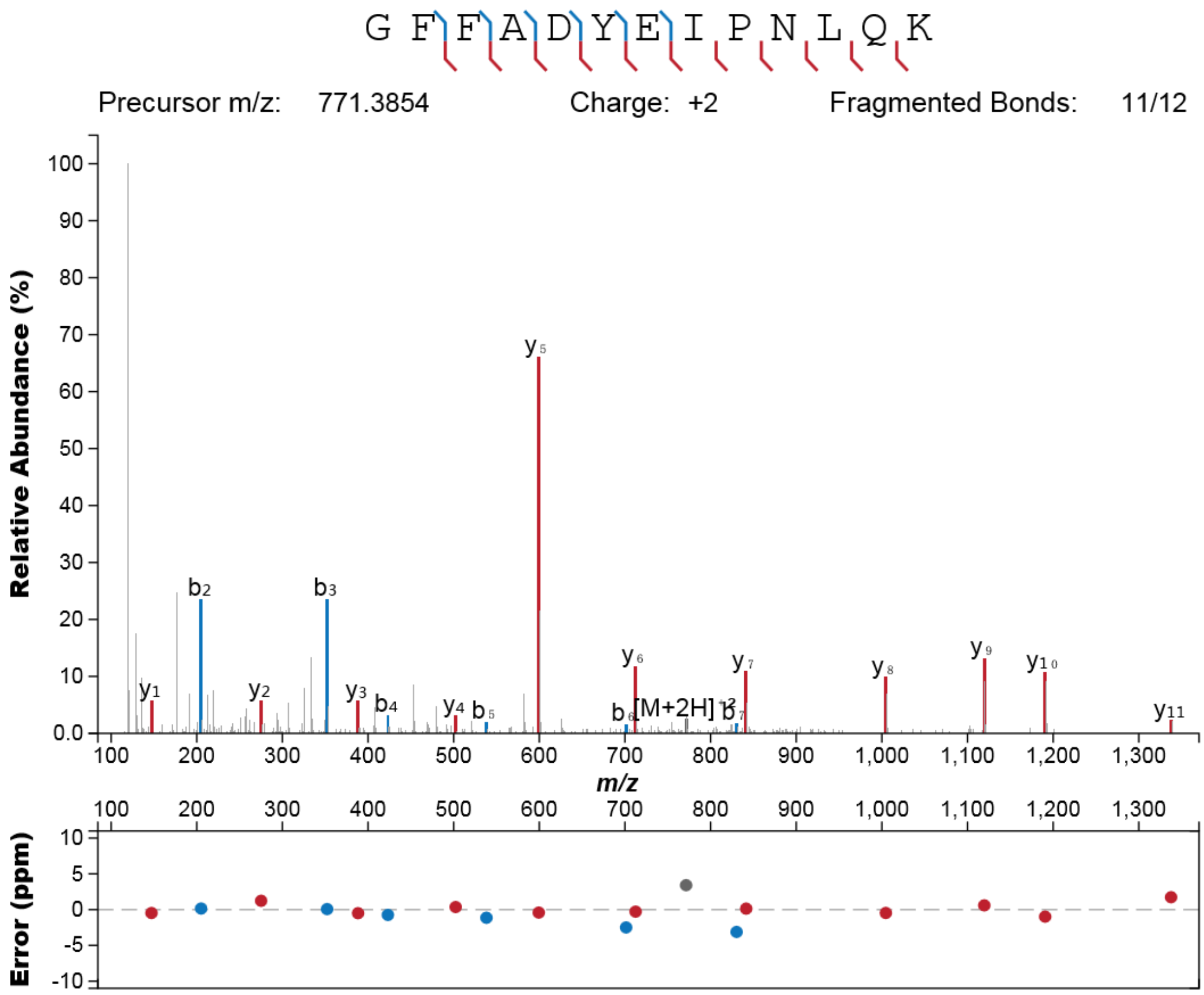

G F F A D Y E I P N L Q K
Precursor m/z: 771.3854
Charge: +2
Fragmented Bonds: 11/12
Relative Abundance (%)
m/z
Error (ppm)
y1
y2
y3
y4
y5
y6
y7
y8
y9
y10
y11
b2
b3
b4
b5
b6
b7
[M+2H]+2

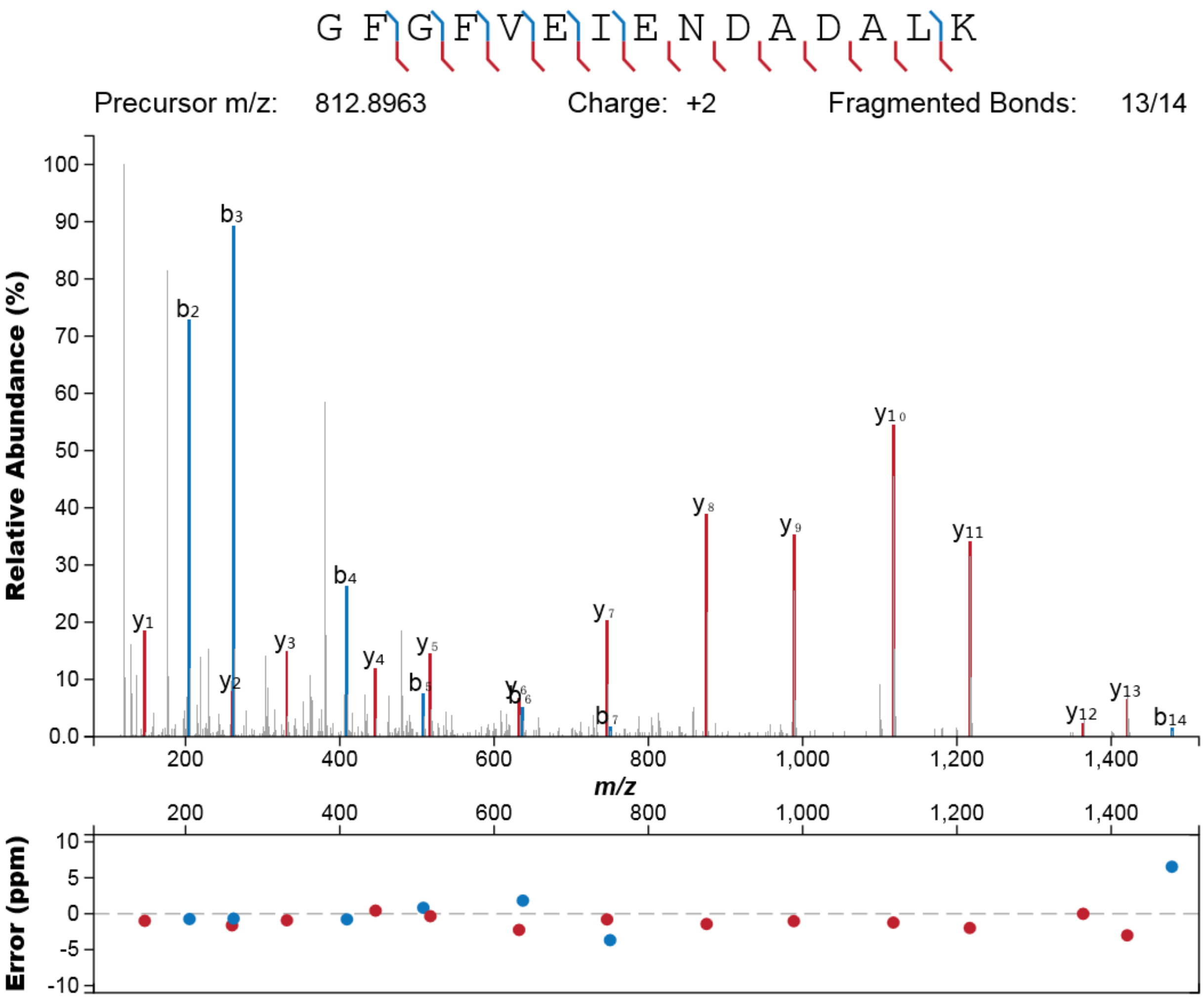
G F G F V E I E N D A D A L K
Precursor m/z: 812.8963
Charge: +2
Fragmented Bonds: 13/14
Relative Abundance (%)
m/z
Error (ppm)
y1
b2
b3
y2
y3
b4
y4
y5
b5
y6
b6
y7
b7
y8
y9
y10
y11
y12
y13
b14

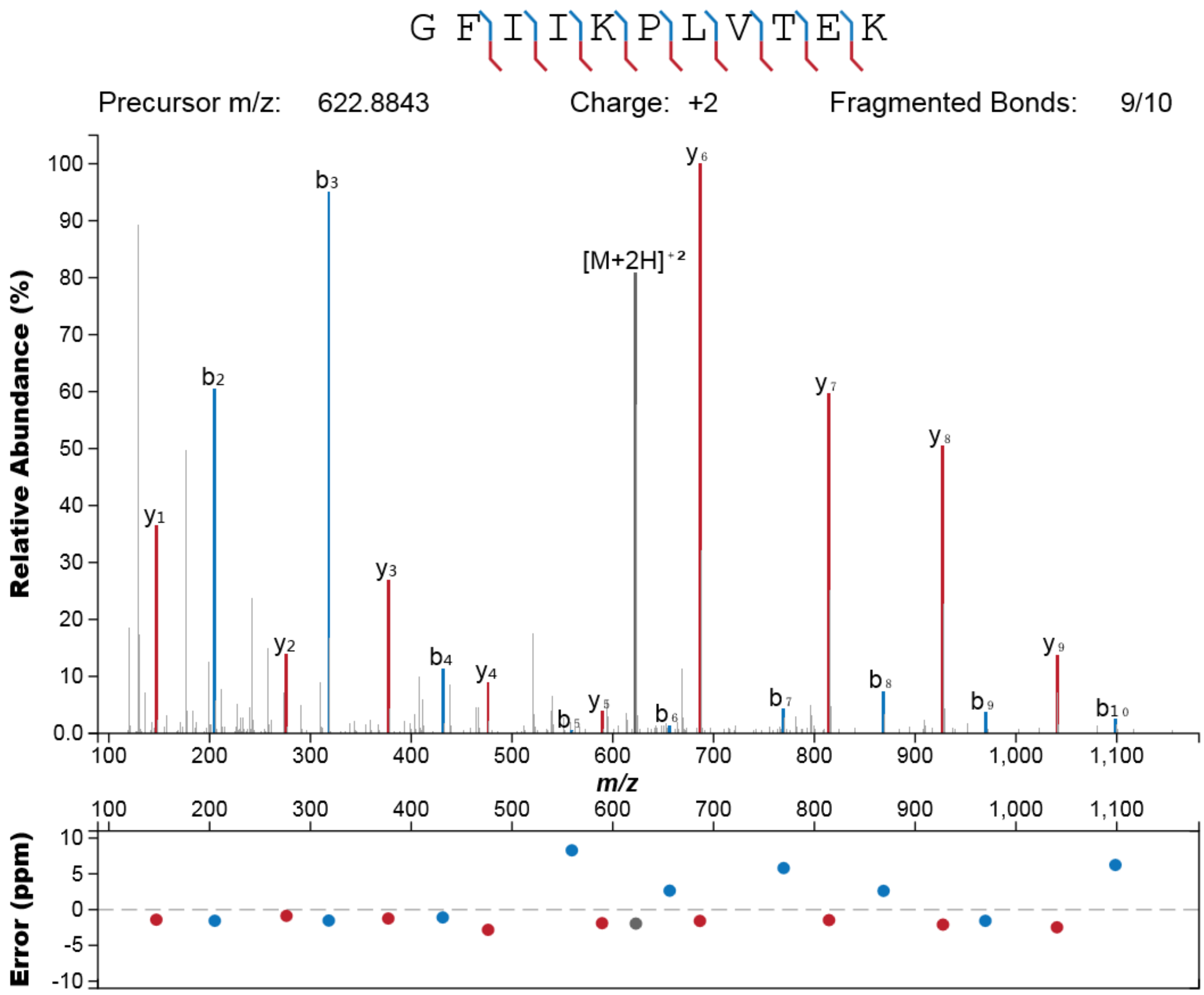
G F I I K P L V T E K
Precursor m/z: 622.8843
Charge: +2
Fragmented Bonds: 9/10
Relative Abundance (%)
m/z
Error (ppm)
[M+2H]+2
y1 y2 y3 y4 y5 y6 y7 y8 y9
b2 b3 b4 b5 b6 b7 b8 b9 b10

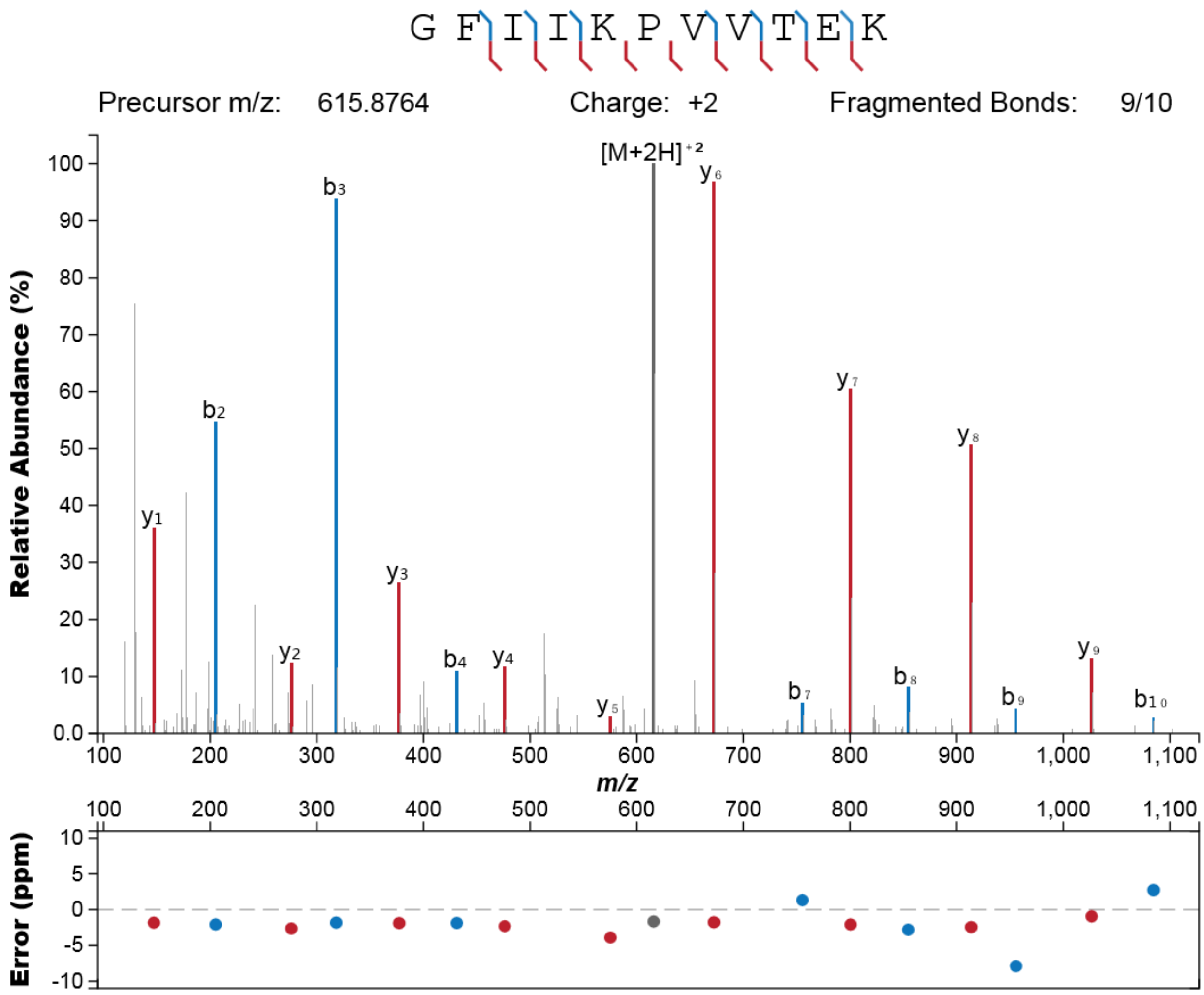
G F I I K P V V T E K
Precursor m/z: 615.8764
Charge: +2
Fragmented Bonds: 9/10
[M+2H]+2
b2
b3
b4
b7
b8
b9
b10
y1
y2
y3
y4
y5
y6
y7
y8
y9
Relative Abundance (%)
m/z
Error (ppm)

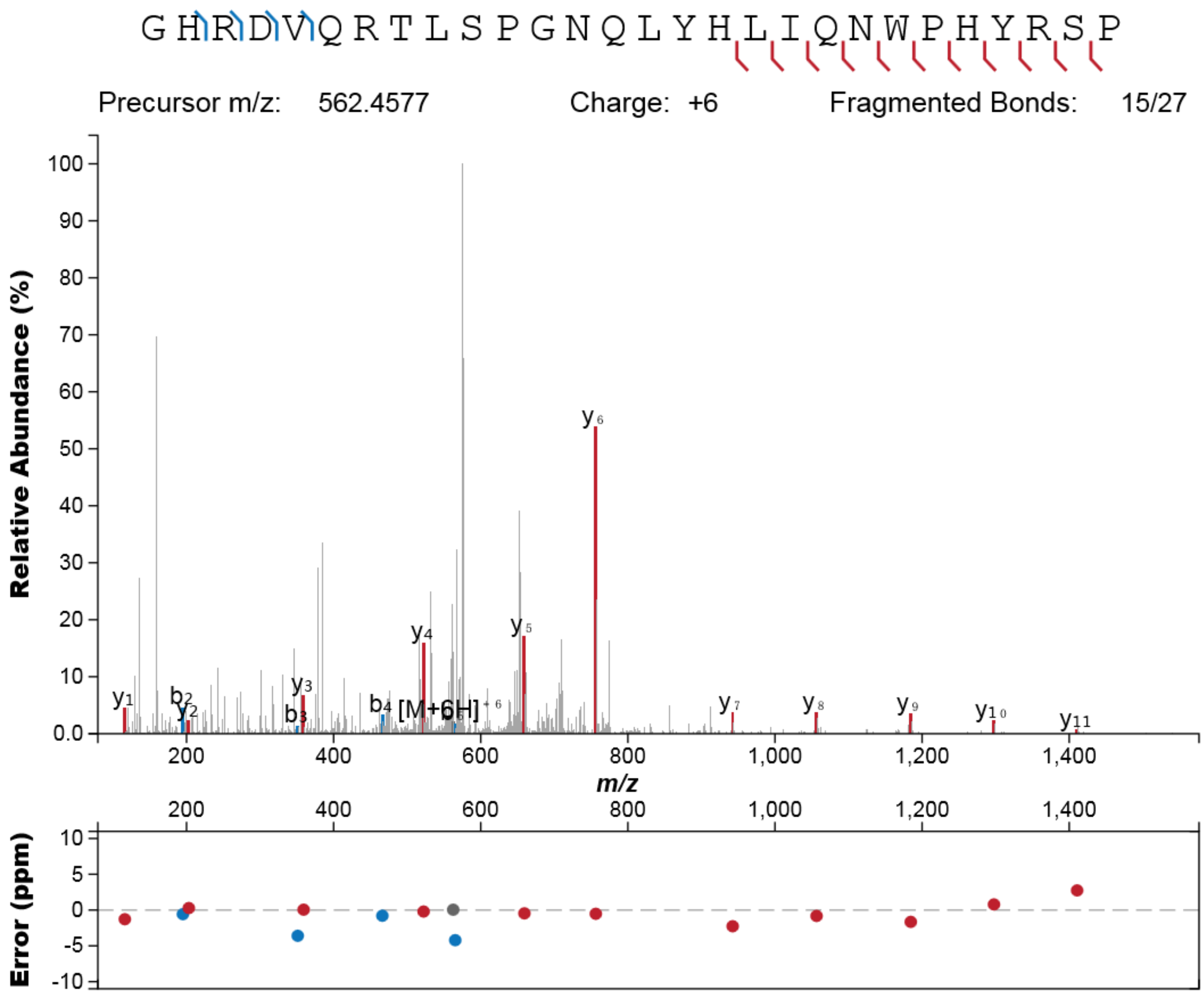

G H R D V Q R T L S P G N Q L Y H L I Q N W P H Y R S P
Precursor m/z: 562.4577
Charge: +6
Fragmented Bonds: 15/27
Relative Abundance (%)
m/z
Error (ppm)
y1
b2
y2
b3
y3
b4
[M+6H]+6
y4
y5
y6
y7
y8
y9
y10
y11

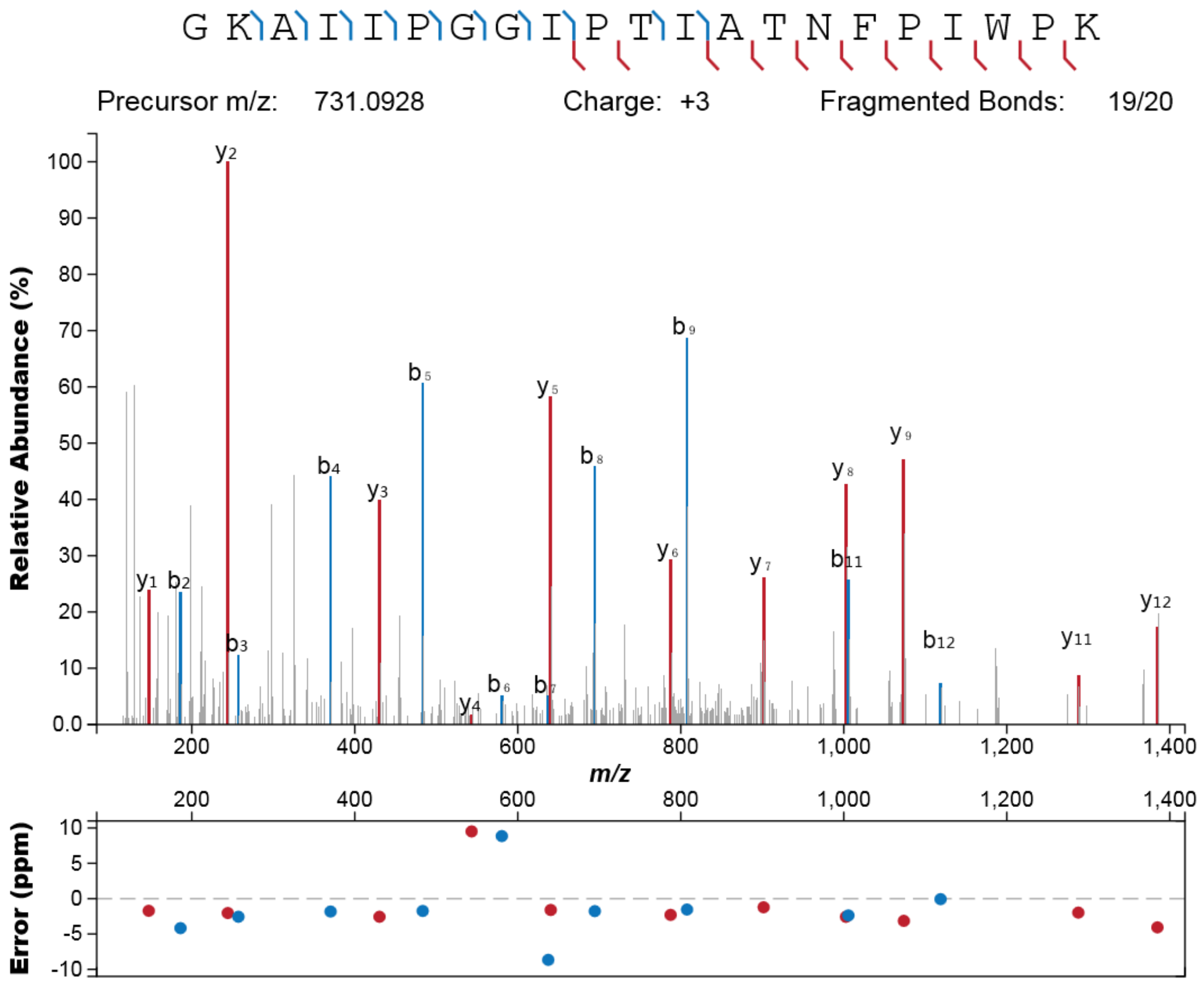
G K A I I P G G I P T I A T N F P I W P K
Precursor m/z: 731.0928
Charge: +3
Fragmented Bonds: 19/20
Relative Abundance (%)
m/z
Error (ppm)
y1 b2 y2 b3 b4 y3 b5 y4 b6 b7 y5 b8 y6 b9 y7 y8 b11 y9 b12 y11 y12

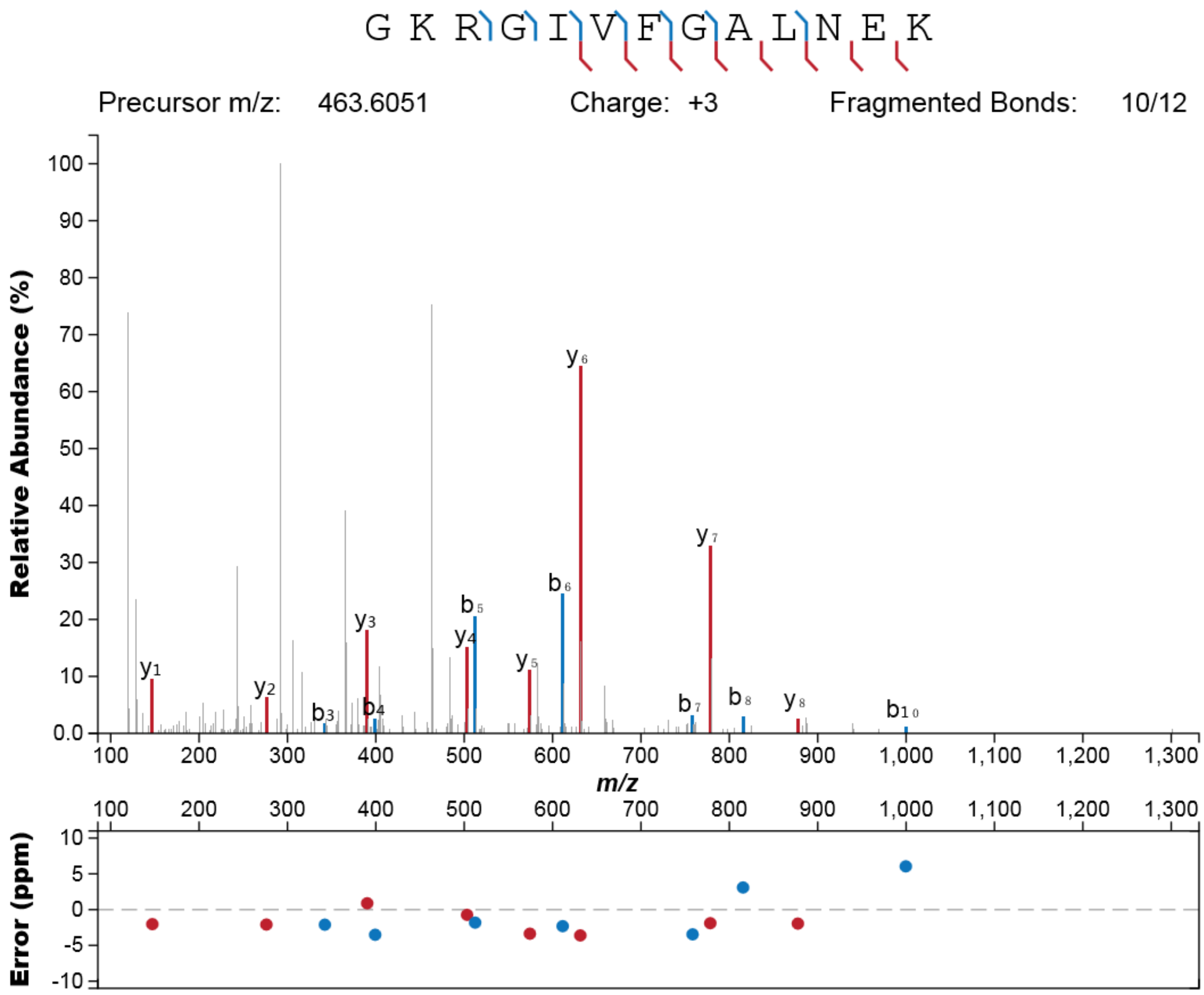
G K R G I V F G A L N E K
Precursor m/z: 463.6051
Charge: +3
Fragmented Bonds: 10/12
Relative Abundance (%)
m/z
Error (ppm)
y1
y2
b3
y3
b4
y4
b5
y5
b6
y6
b7
y7
b8
y8
b10

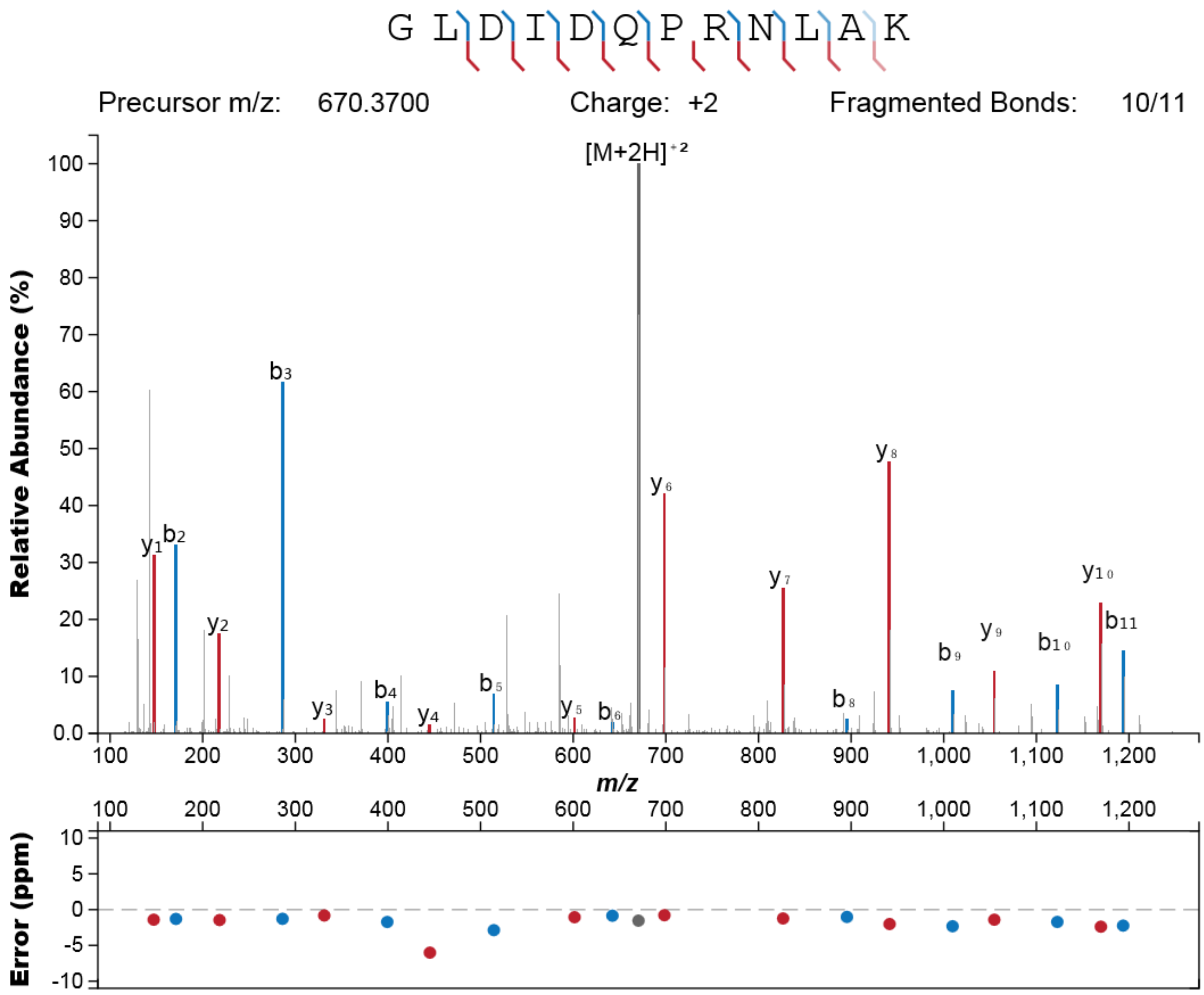
G L D I D Q P R N L A K
Precursor m/z: 670.3700
Charge: +2
Fragmented Bonds: 10/11
[M+2H]+2
Relative Abundance (%)
m/z
Error (ppm)
y1
b2
y2
b3
y3
b4
y4
b5
y5
b6
y6
y7
b8
y8
b9
y9
b10
y10
b11

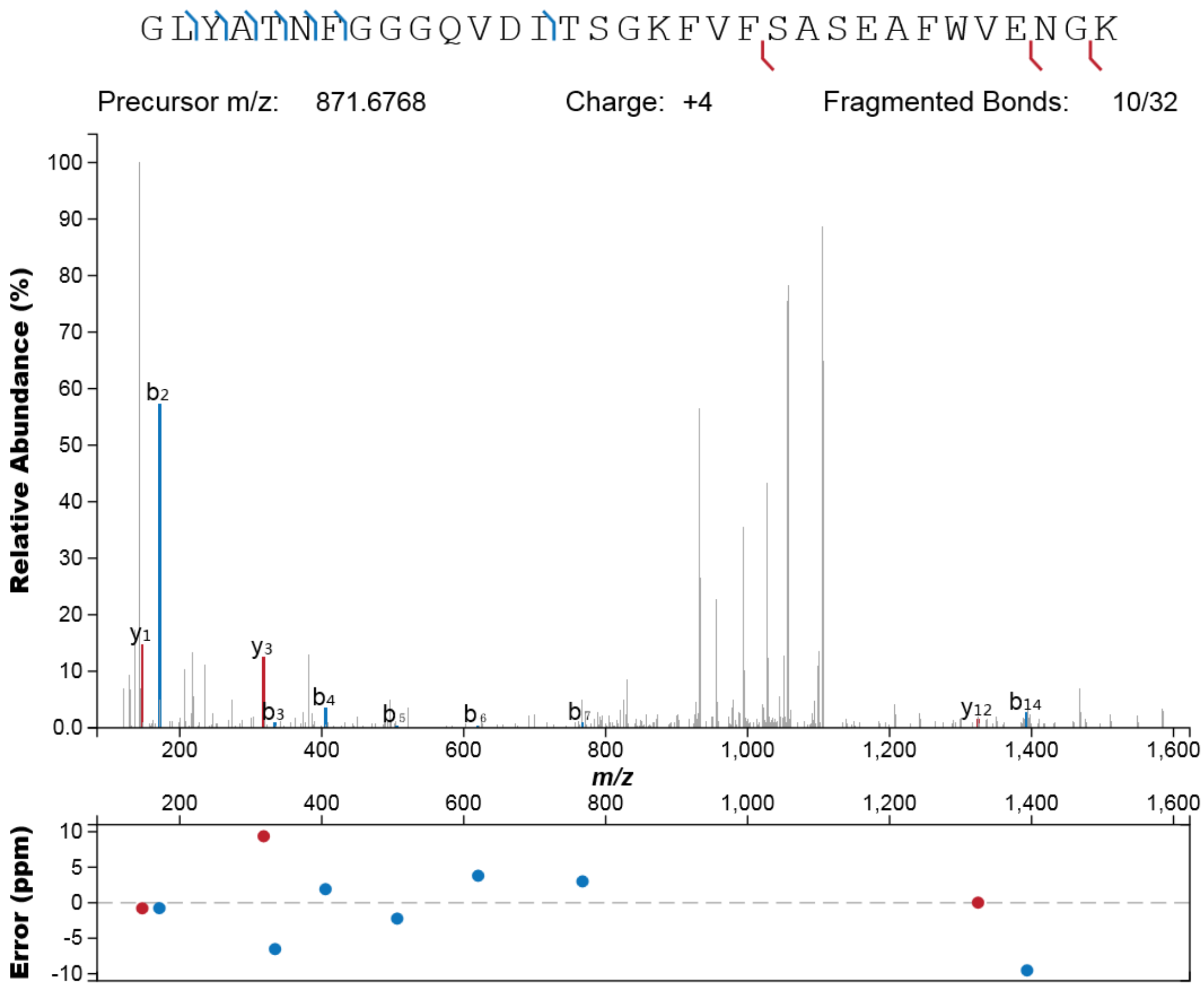

G L Y A T N F G G G Q V D I T S G K F V F S A S E A F W V E N G K
Precursor m/z: 871.6768
Charge: +4
Fragmented Bonds: 10/32
Relative Abundance (%)
m/z
Error (ppm)

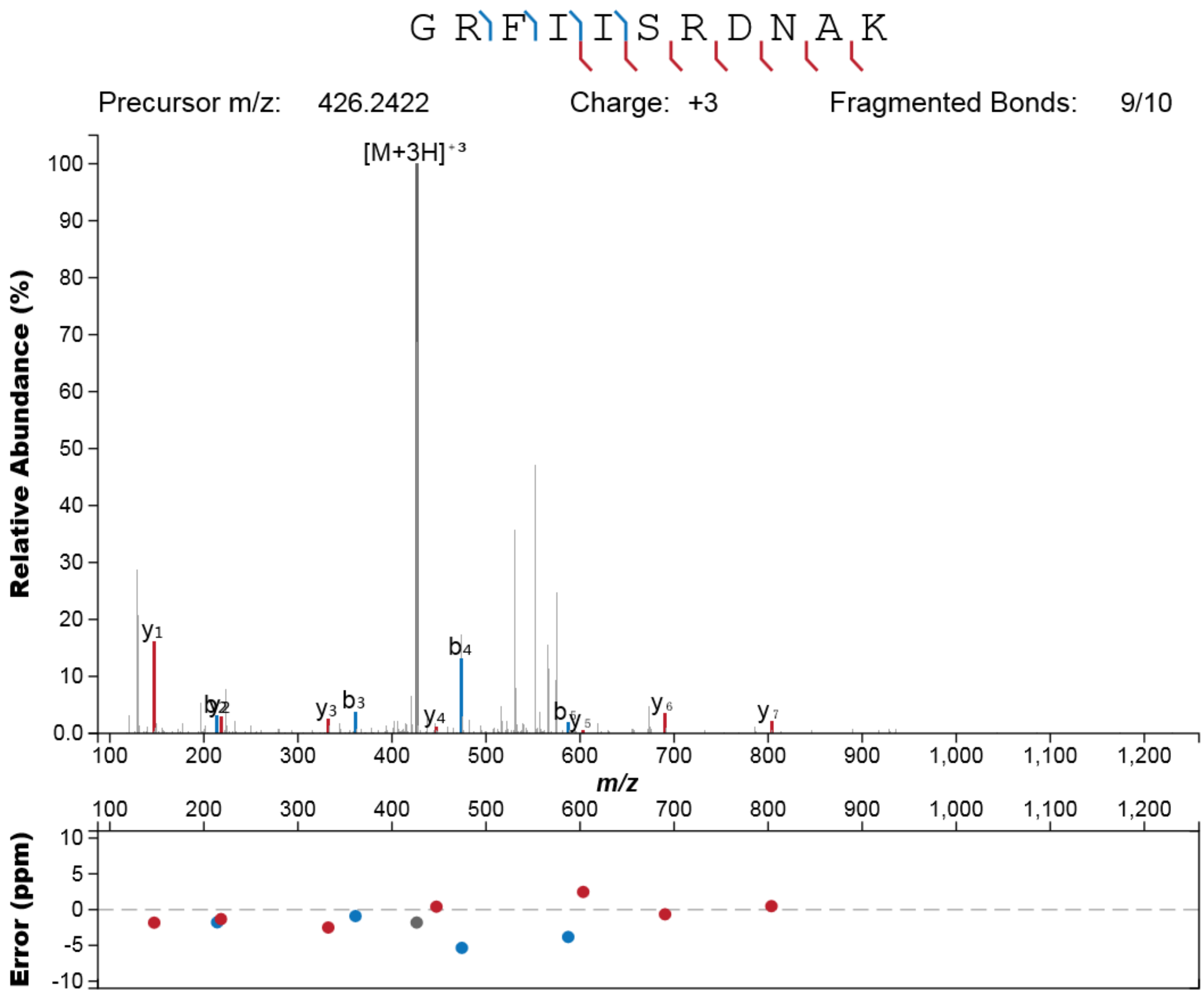
G R F I I S R D N A K
Precursor m/z: 426.2422
Charge: +3
Fragmented Bonds: 9/10
[M+3H]+3
Relative Abundance (%)
y1
b2
y2
y3
b3
y4
b4
b5
y5
y6
y7
m/z
Error (ppm)

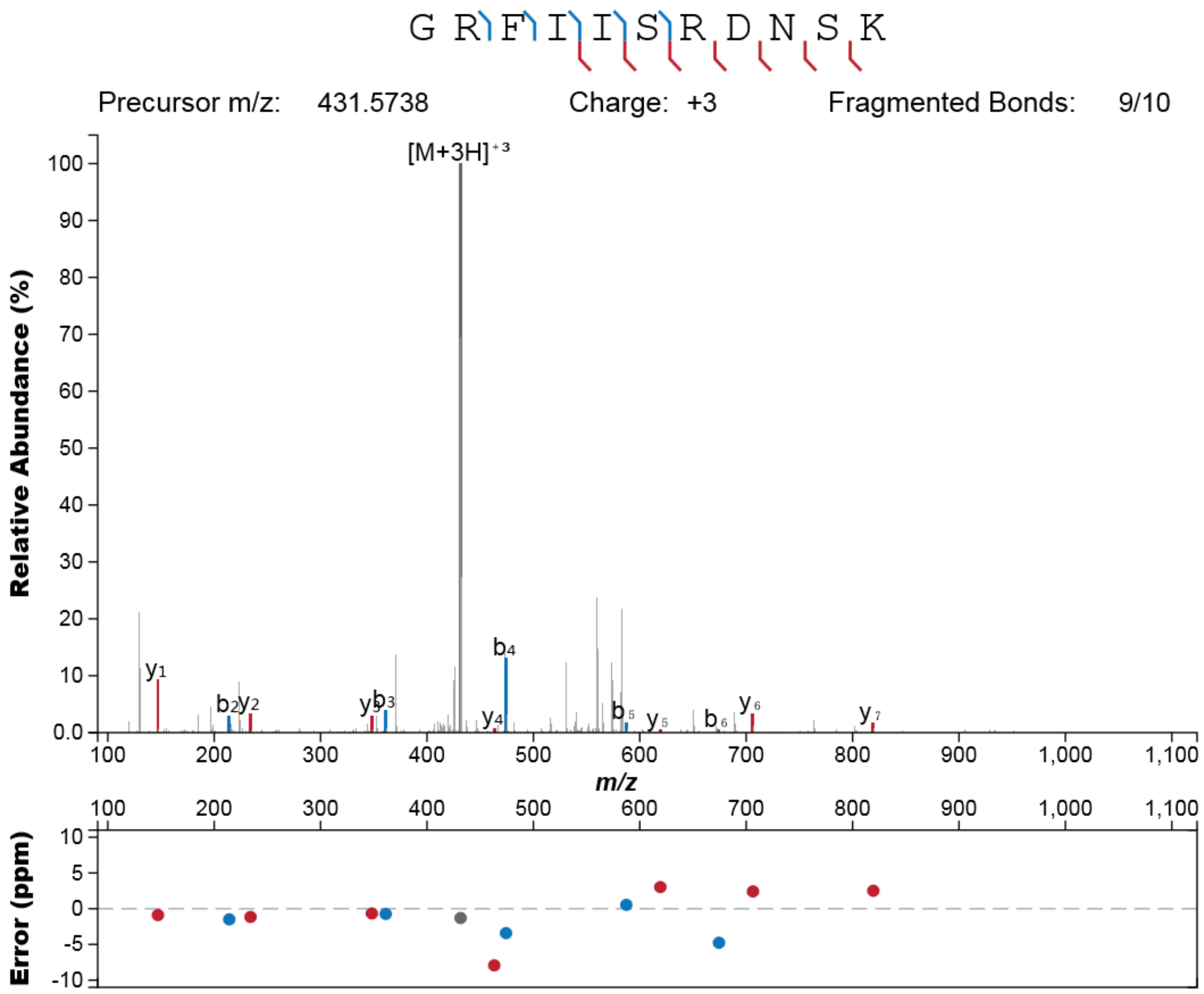

G R F I I S R D N S K
Precursor m/z: 431.5738
Charge: +3
Fragmented Bonds: 9/10
[M+3H]+3
Relative Abundance (%)
m/z
Error (ppm)

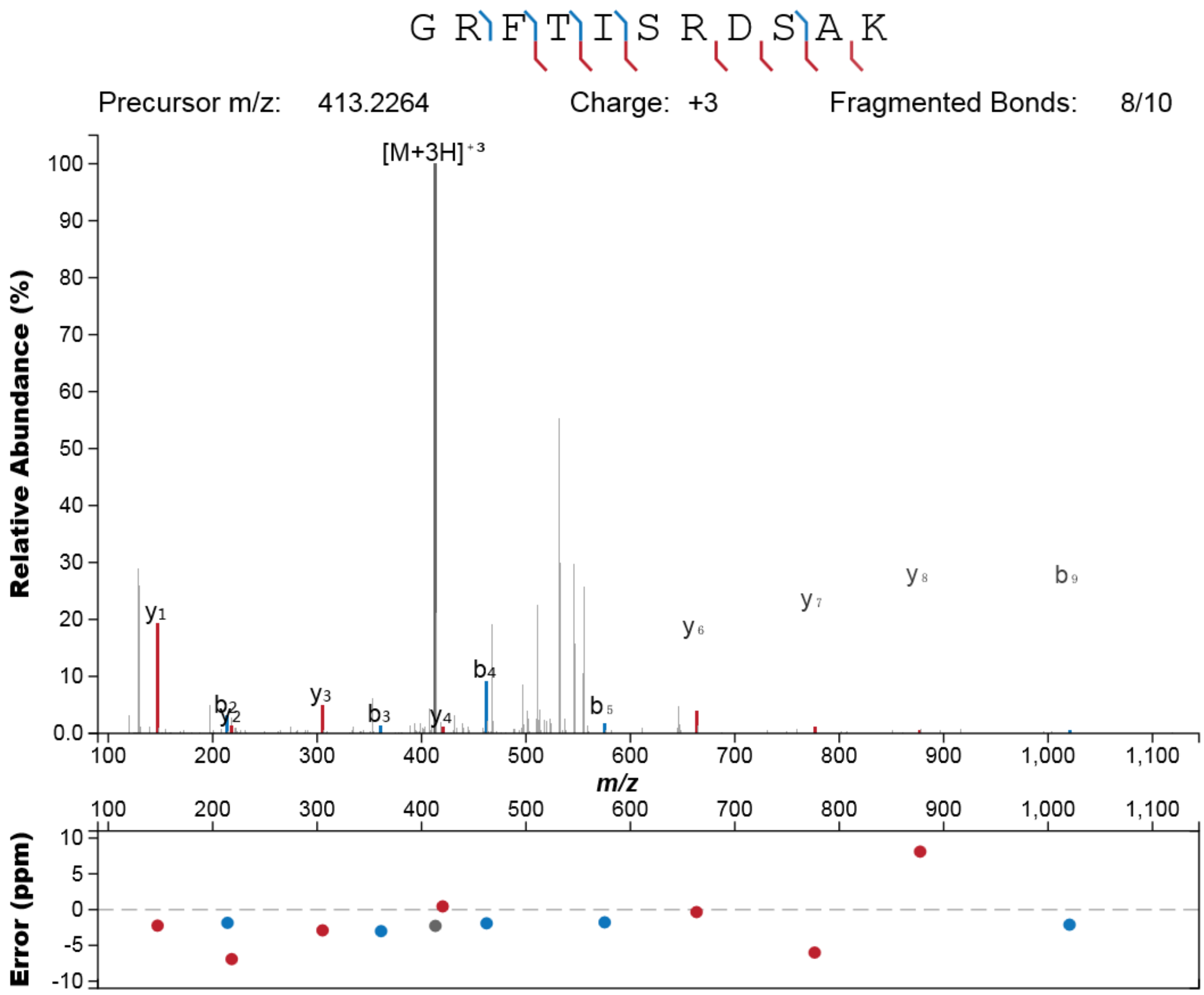

G R F T I S R D S A K
Precursor m/z: 413.2264
Charge: +3
Fragmented Bonds: 8/10
[M+3H]+3
Relative Abundance (%)
y1
b2
y2
y3
b3
y4
b4
b5
y6
y7
y8
b9
m/z
Error (ppm)

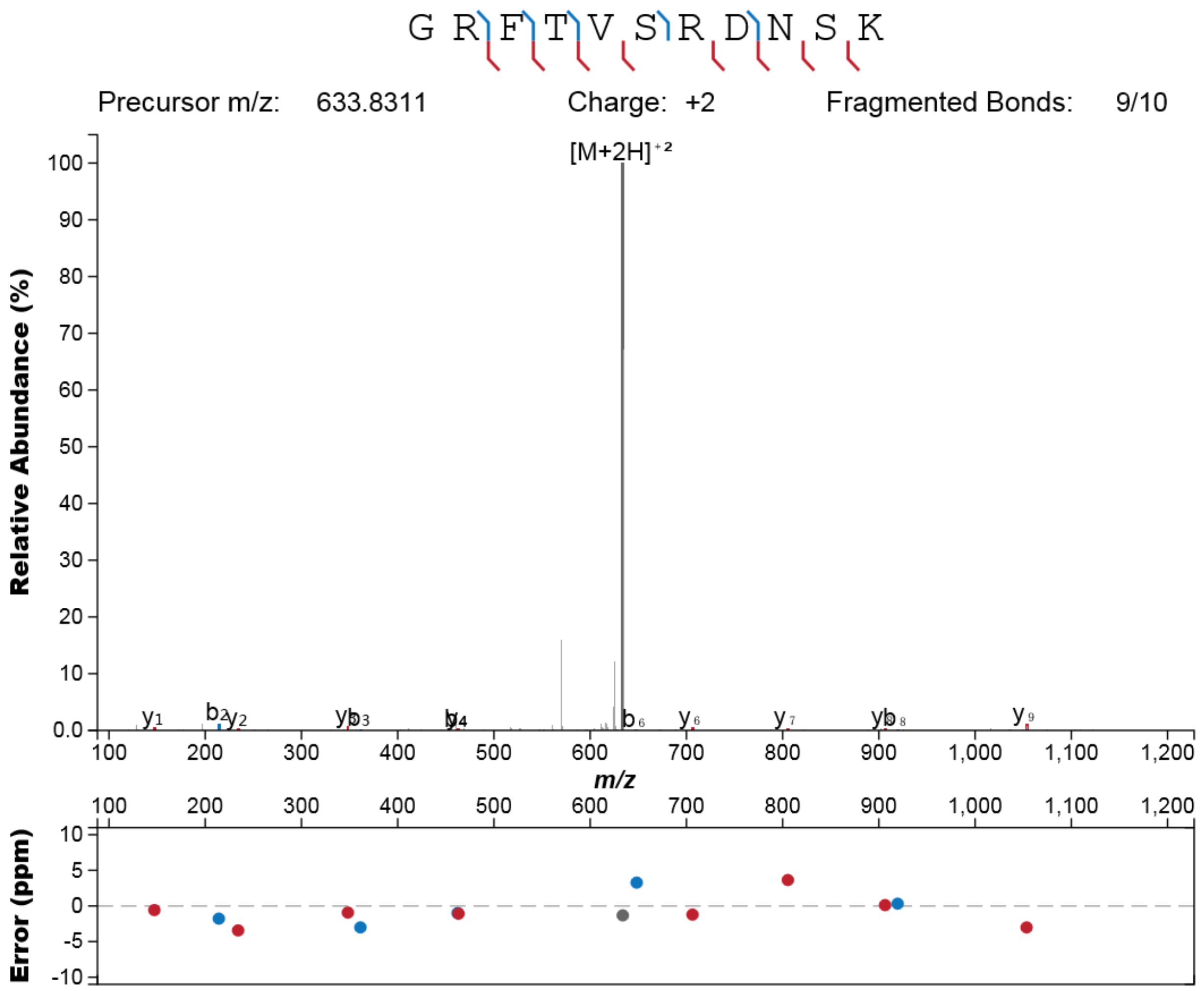
G R F T V S R D N S K
Precursor m/z: 633.8311
Charge: +2
Fragmented Bonds: 9/10
[M+2H]+2
Relative Abundance (%)
m/z
Error (ppm)

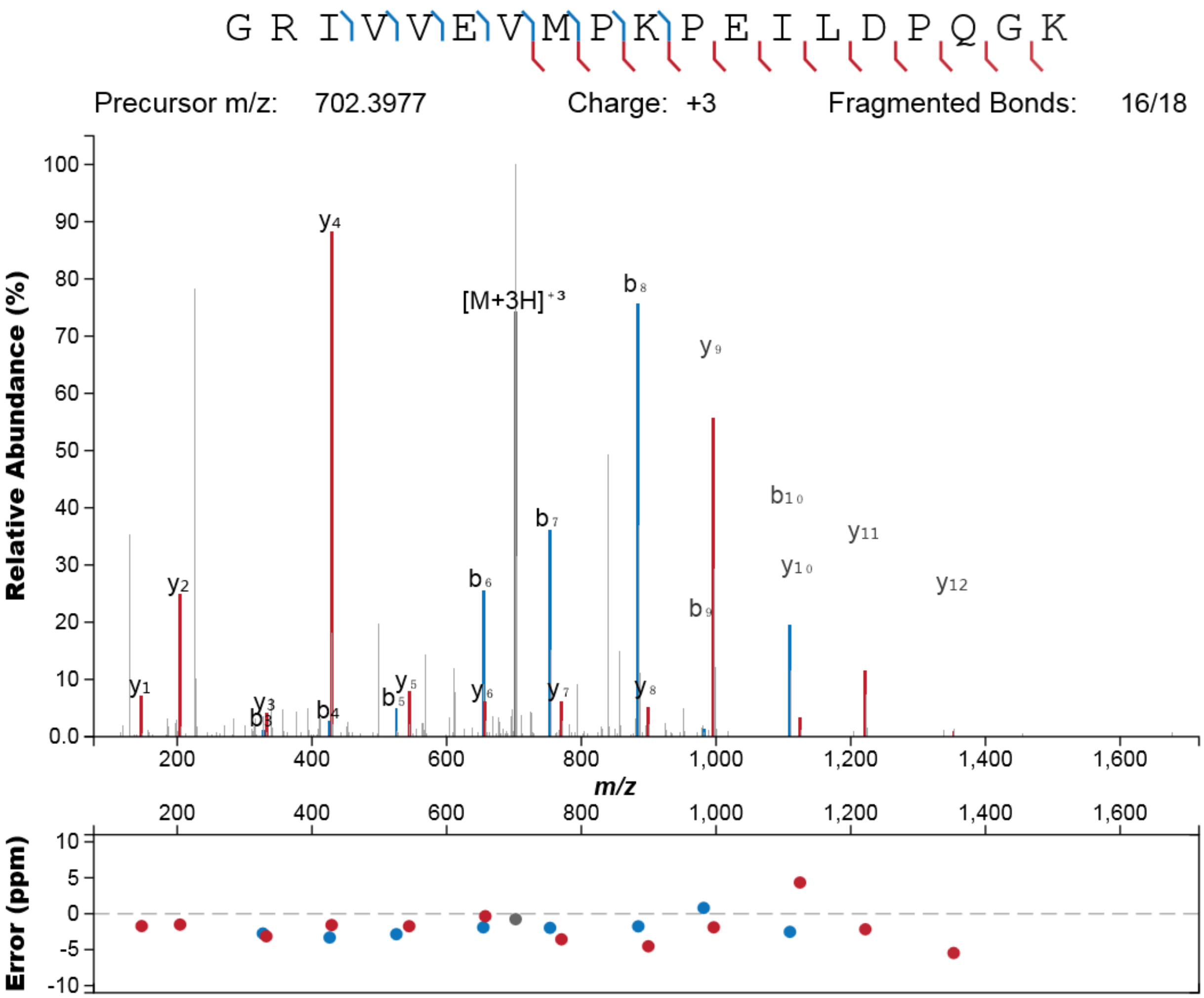

G R I V V E V M P K P E I L D P Q G K
Precursor m/z: 702.3977
Charge: +3
Fragmented Bonds: 16/18
Relative Abundance (%)
m/z
Error (ppm)
[M+3H]+3
y1
y2
y3
y4
y5
y6
y7
y8
y9
y10
y11
y12
b3
b4
b5
b6
b7
b8
b9
b10

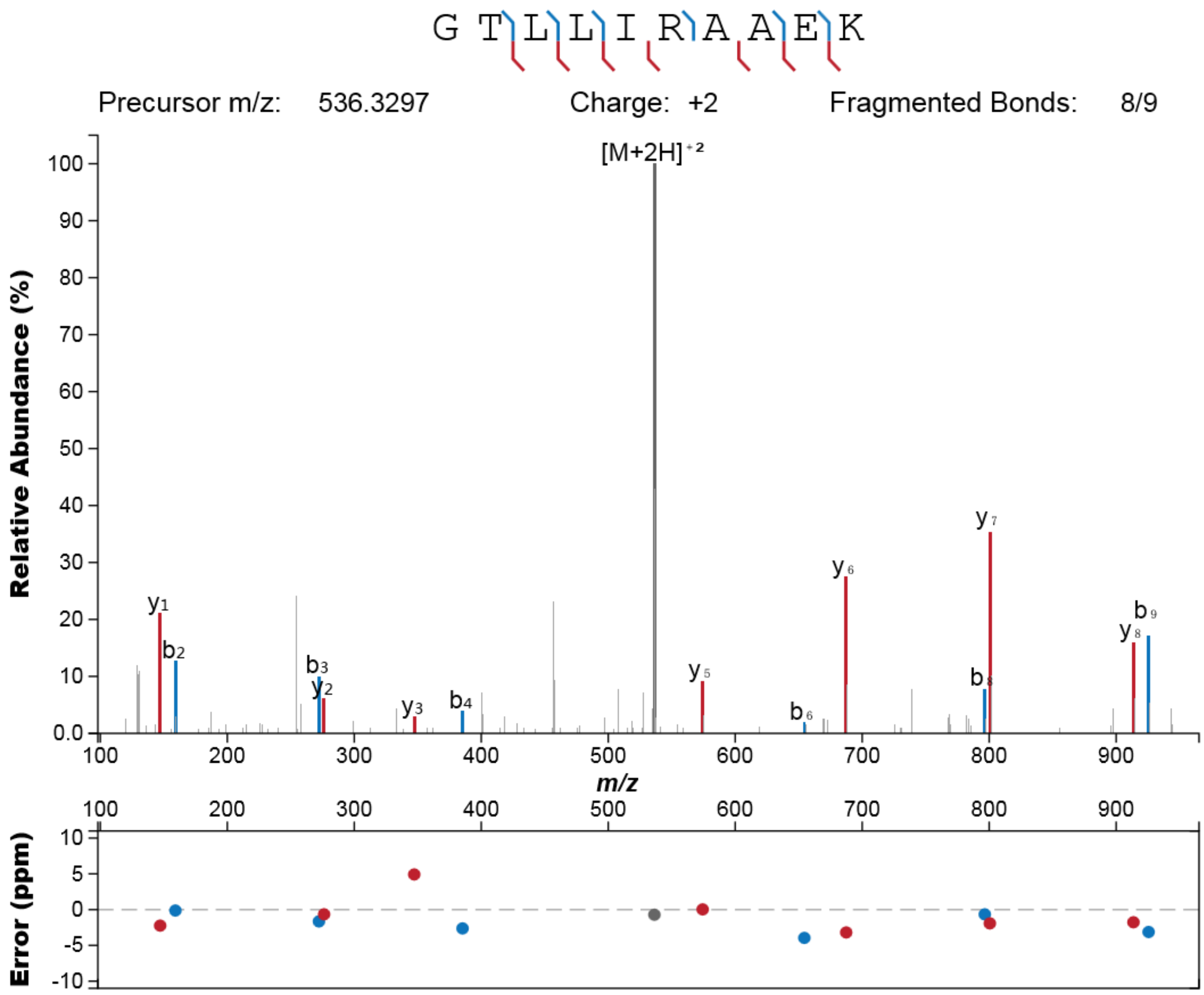

G T L L I R A A E K
Precursor m/z: 536.3297
Charge: +2
Fragmented Bonds: 8/9
[M+2H]+2
Relative Abundance (%)
m/z
Error (ppm)
y1
b2
b3
y2
y3
b4
y5
b6
y6
b8
y7
y8
b9

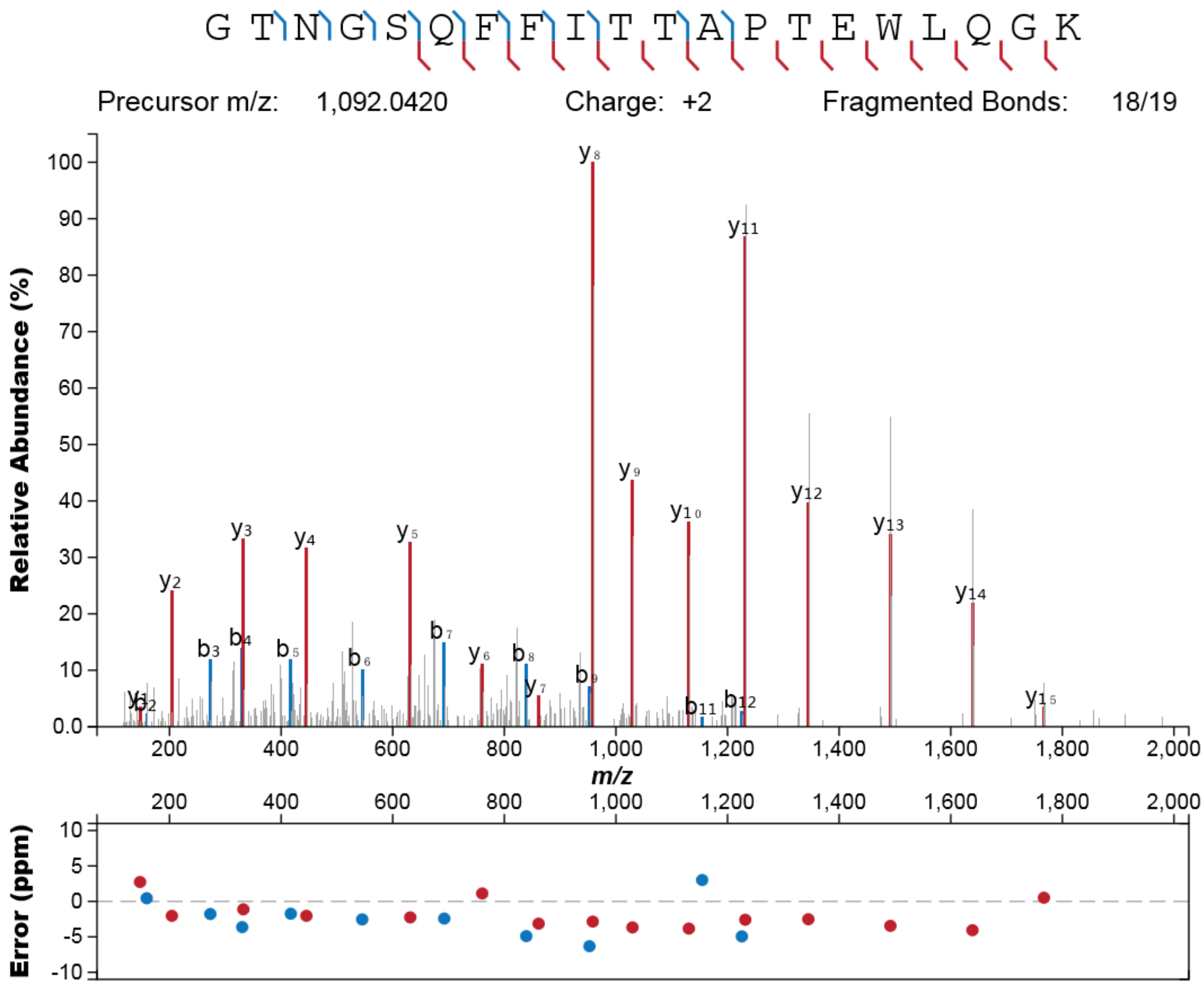
G T N G S Q F F I T T A P T E W L Q G K
Precursor m/z: 1,092.0420
Charge: +2
Fragmented Bonds: 18/19
Relative Abundance (%)
m/z
Error (ppm)
y2
y3
y4
y5
y6
y7
y8
y9
y10
y11
y12
y13
y14
y15
b2
b3
b4
b5
b6
b7
b8
b9
b11
b12

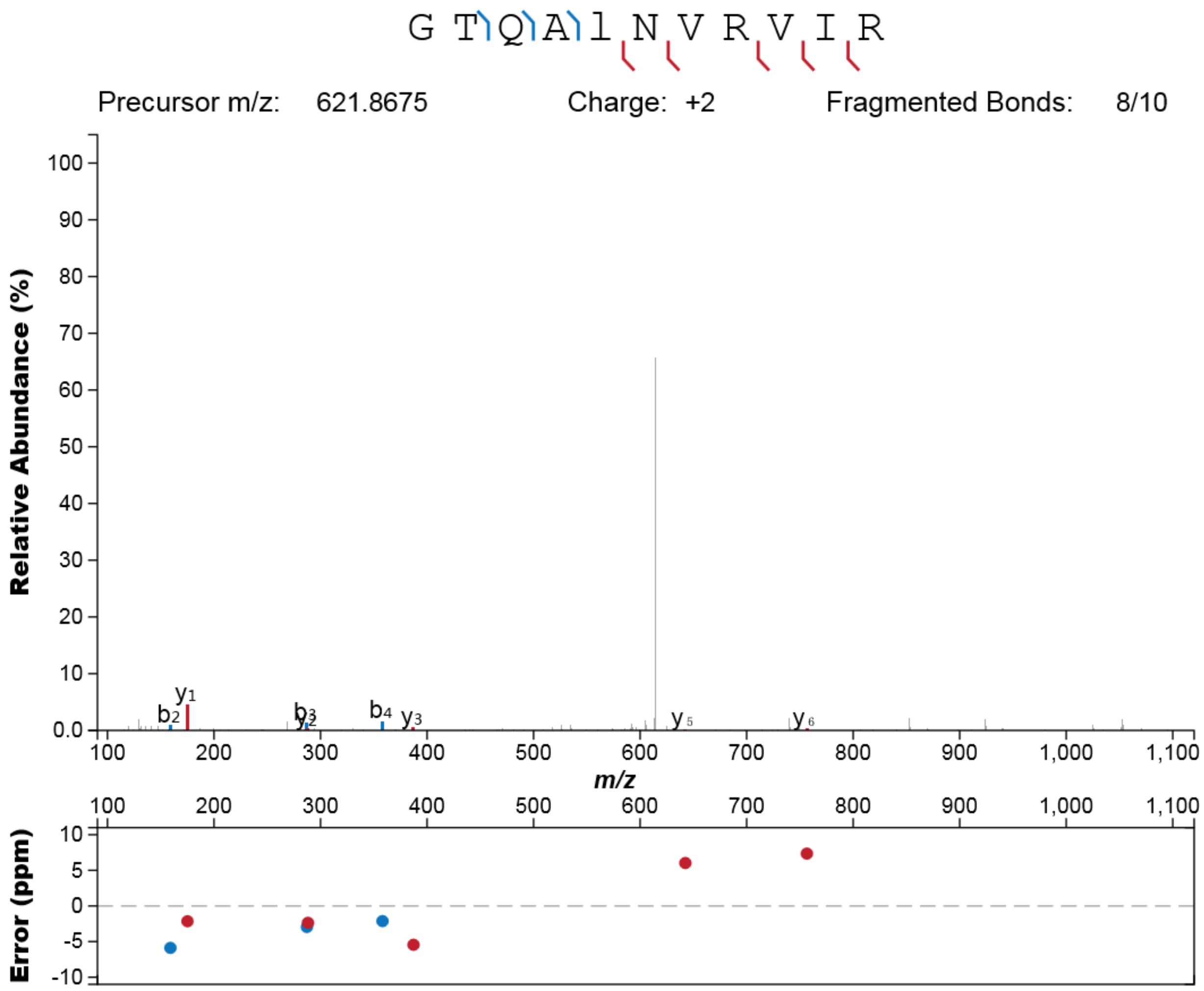

G T Q A l N V R V I R
Precursor m/z: 621.8675
Charge: +2
Fragmented Bonds: 8/10
Relative Abundance (%)
m/z
Error (ppm)
b2
y1
b3
y2
b4
y3
y5
y6

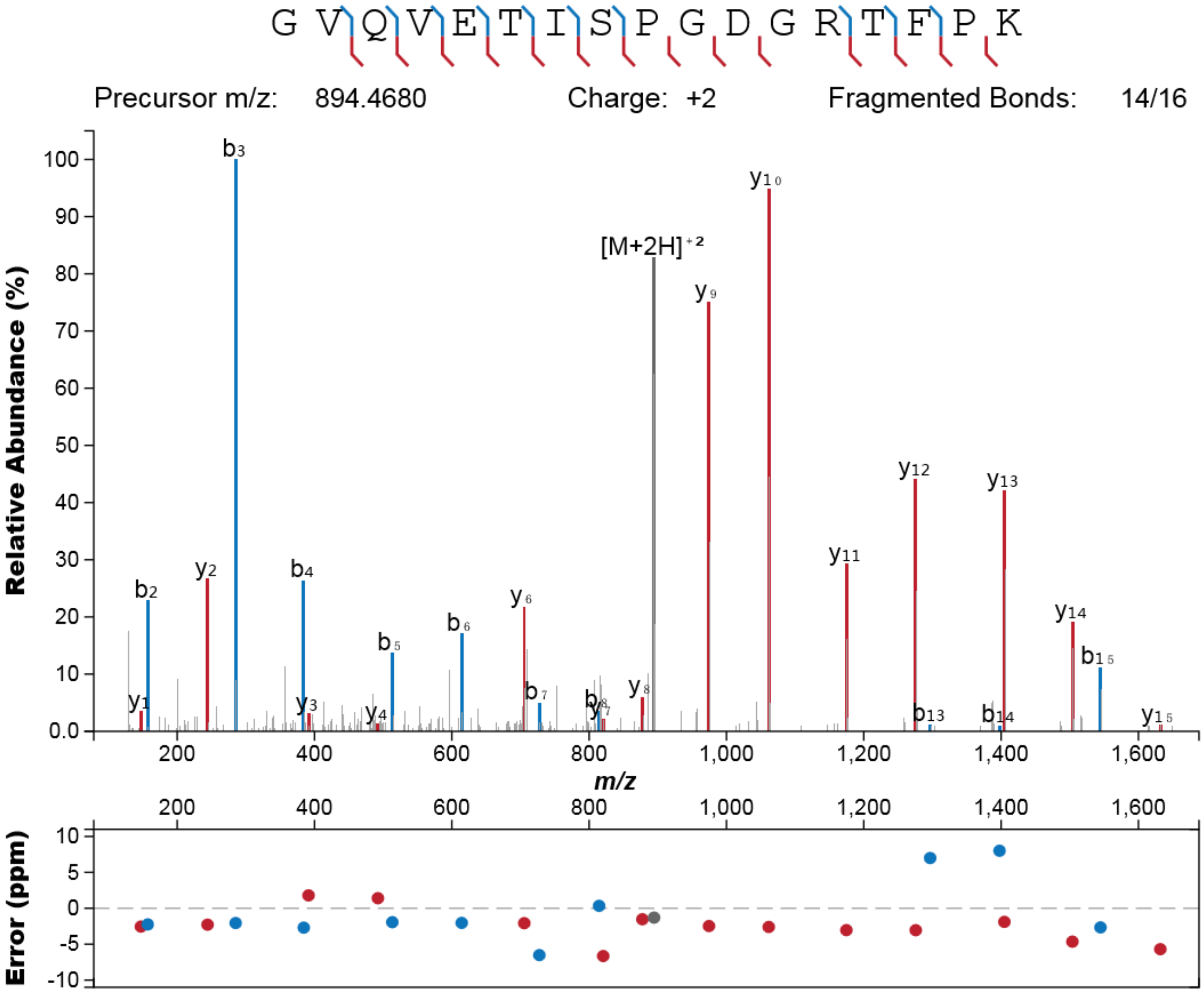
G V Q V E T I S P G D G R T F P K
Precursor m/z: 894.4680
Charge: +2
Fragmented Bonds: 14/16
Relative Abundance (%)
m/z
Error (ppm)
[M+2H]+2

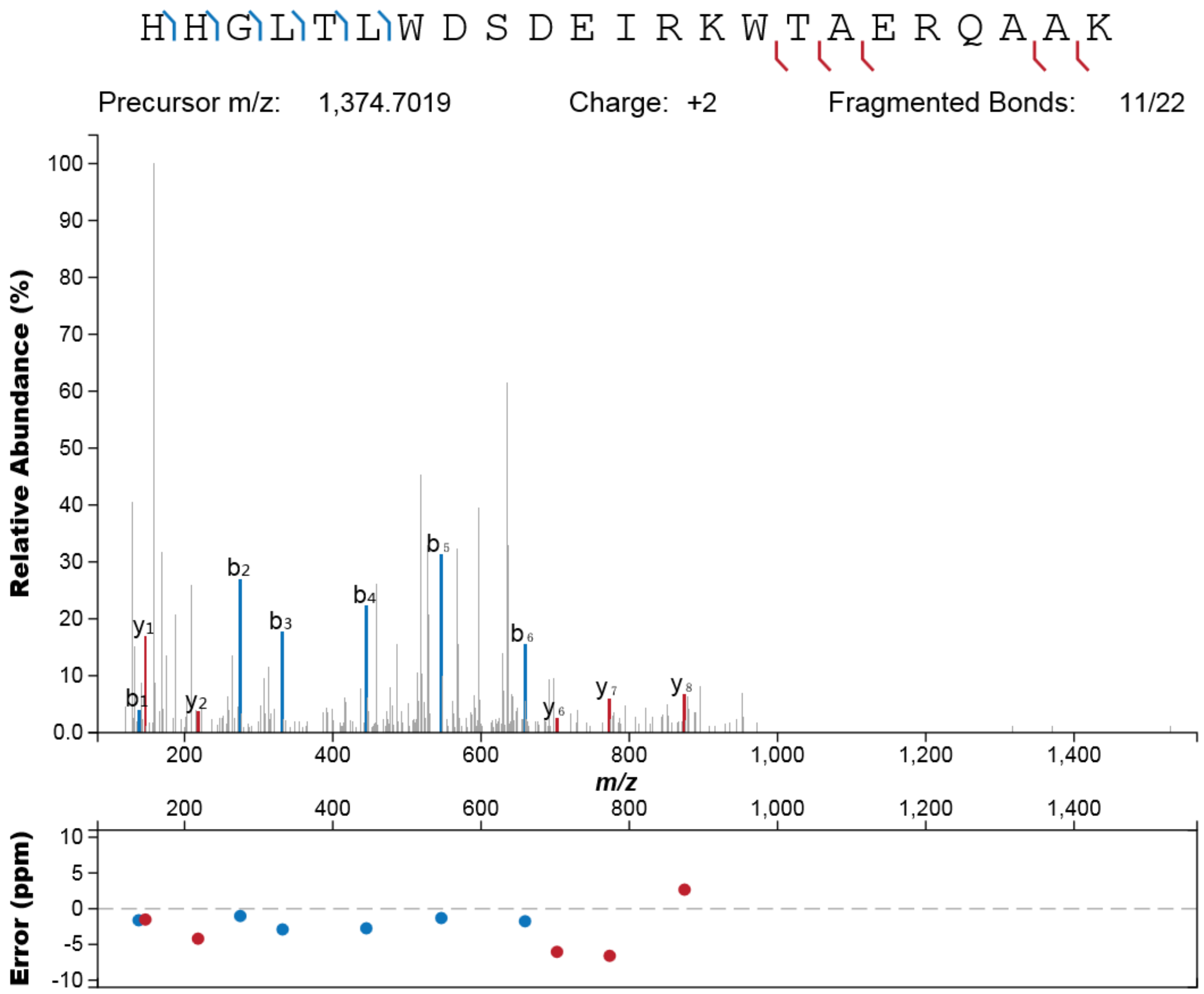
H H G L T L W D S D E I R K W T A E R Q A A K
Precursor m/z: 1,374.7019
Charge: +2
Fragmented Bonds: 11/22
Relative Abundance (%)
m/z
Error (ppm)
b1 b2 b3 b4 b5 b6
y1 y2 y6 y7 y8

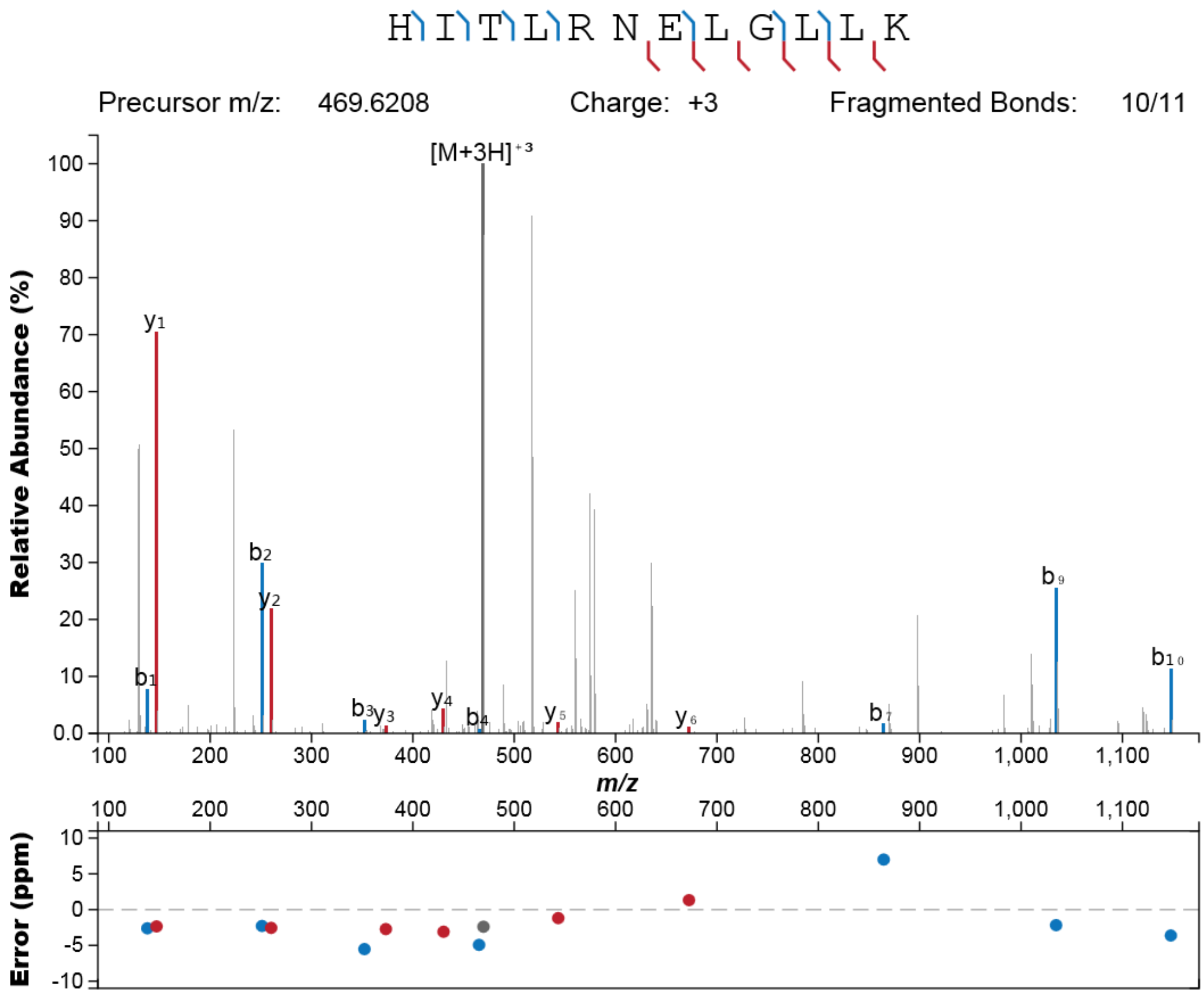
H I T L R N E L G L L K
Precursor m/z: 469.6208
Charge: +3
Fragmented Bonds: 10/11
[M+3H]+3
y1
b2
y2
b1
b3
y3
y4
b4
y5
y6
b7
b9
b10
Relative Abundance (%)
m/z
Error (ppm)

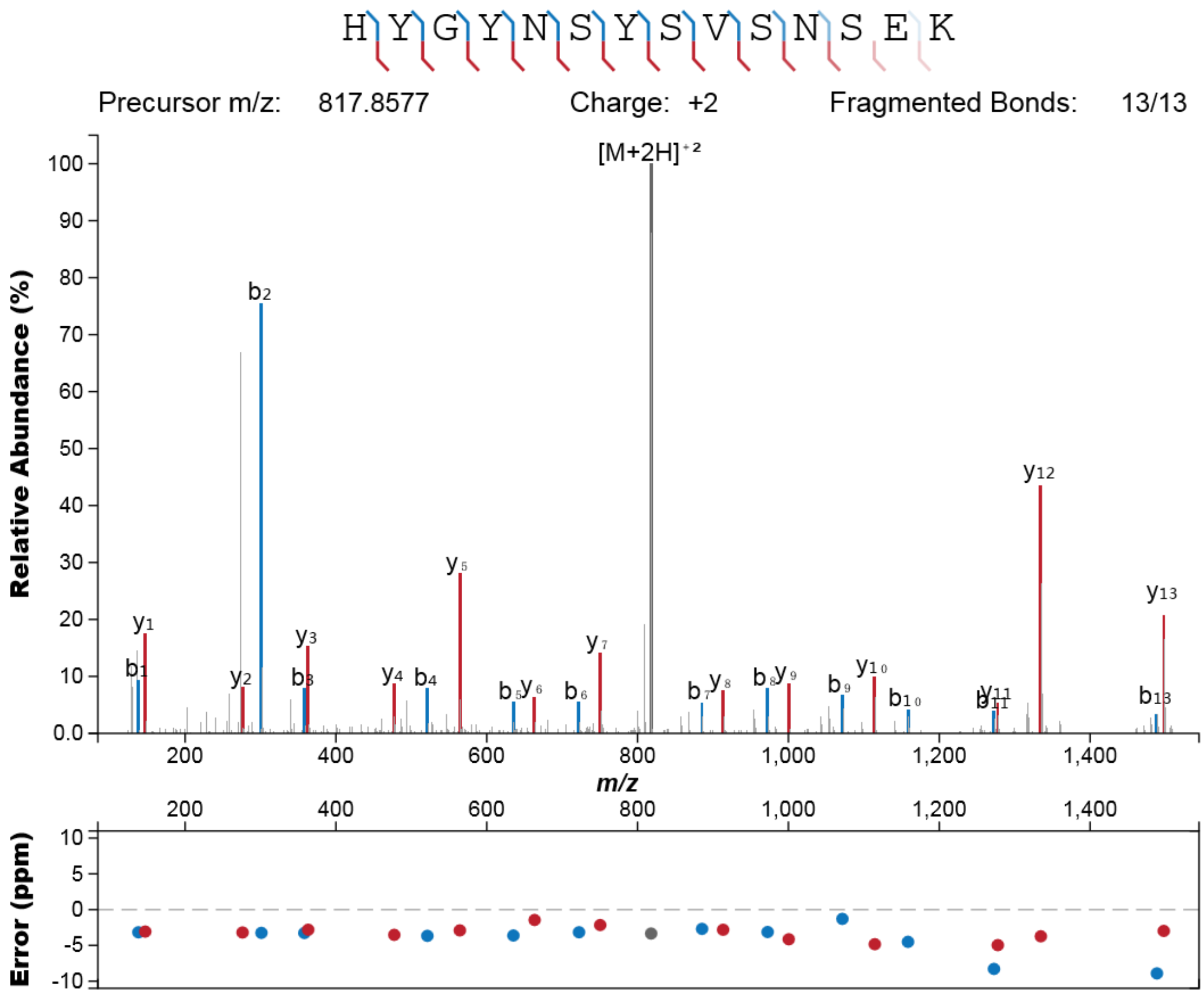

H Y G Y N S Y S V S N S E K
Precursor m/z: 817.8577
Charge: +2
Fragmented Bonds: 13/13
[M+2H]+2
Relative Abundance (%)
m/z
Error (ppm)

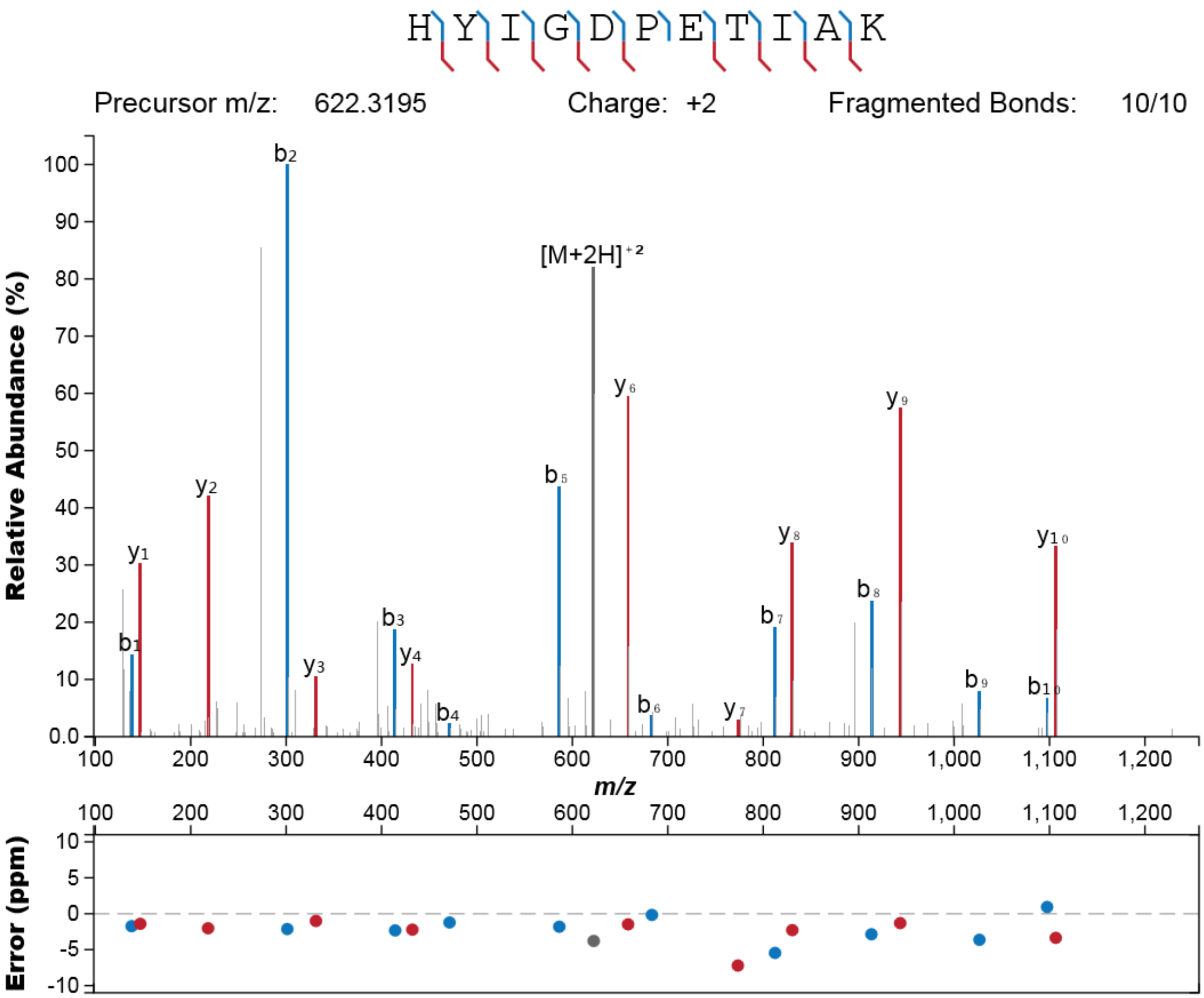

H Y I G D P E T I A K
Precursor m/z: 622.3195
Charge: +2
Fragmented Bonds: 10/10
Relative Abundance (%)
m/z
Error (ppm)
[M+2H]+2
b1 b2 b3 b4 b5 b6 b7 b8 b9 b10
y1 y2 y3 y4 y6 y7 y8 y9 y10

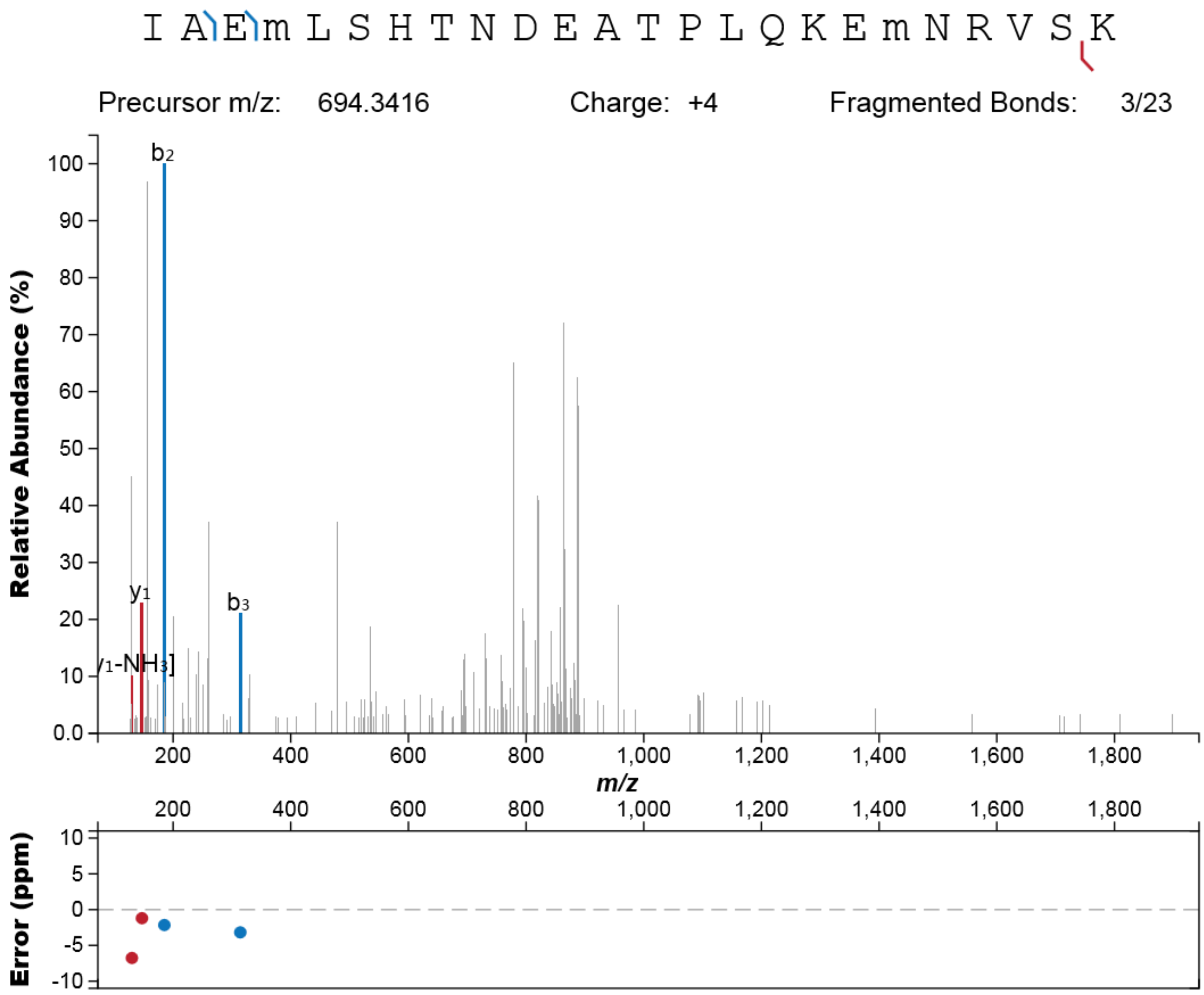

I A E m L S H T N D E A T P L Q K E m N R V S K
Precursor m/z: 694.3416
Charge: +4
Fragmented Bonds: 3/23
Relative Abundance (%)
b2
b3
y1
y1-NH3]
m/z
Error (ppm)

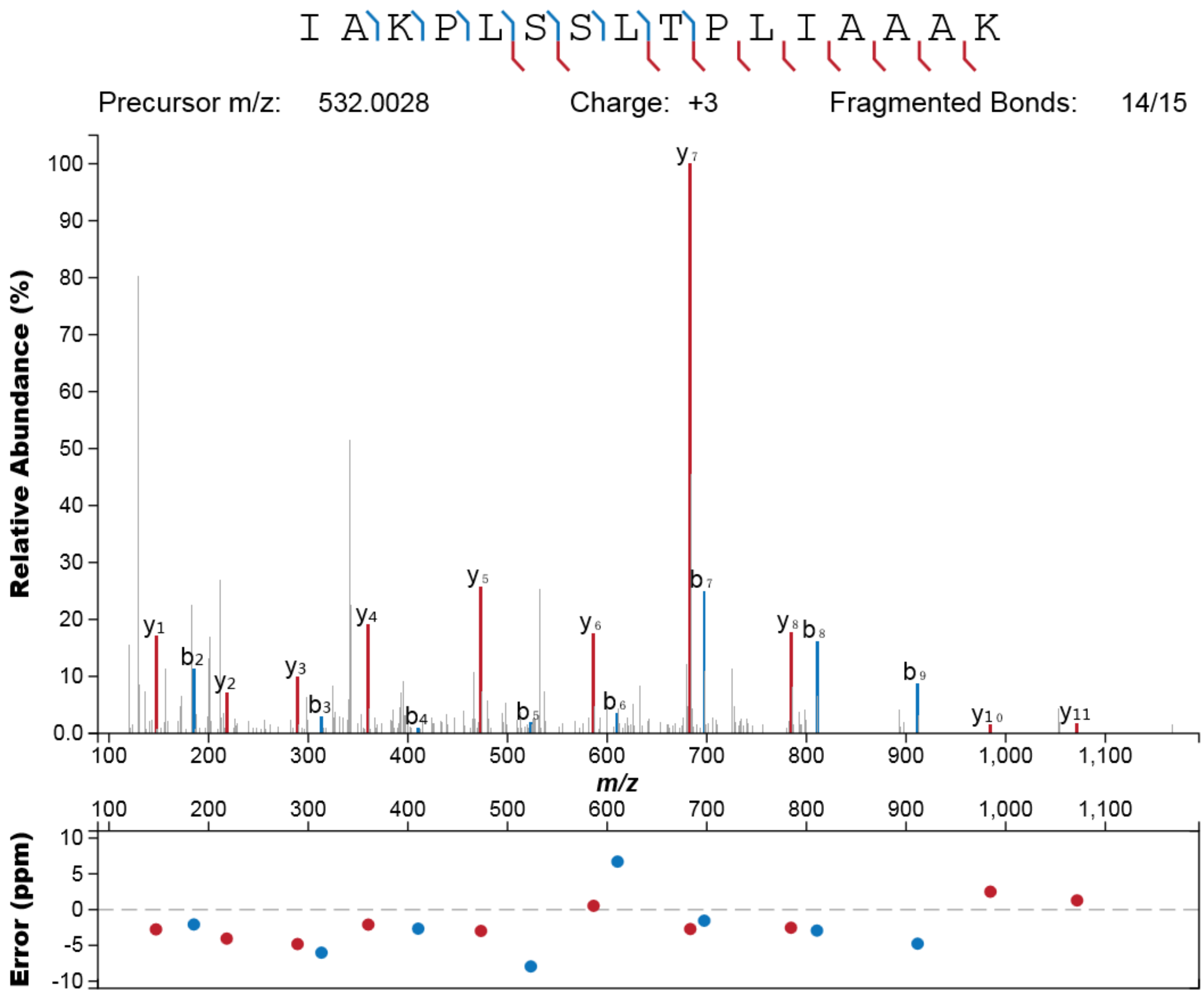

I A K P L S S L T P L I A A A K
Precursor m/z: 532.0028
Charge: +3
Fragmented Bonds: 14/15
Relative Abundance (%)
m/z
Error (ppm)
y7
b7
y5
y4
y1
b2
y2
y3
b3
b4
b5
y6
b6
y8
b8
b9
y10
y11

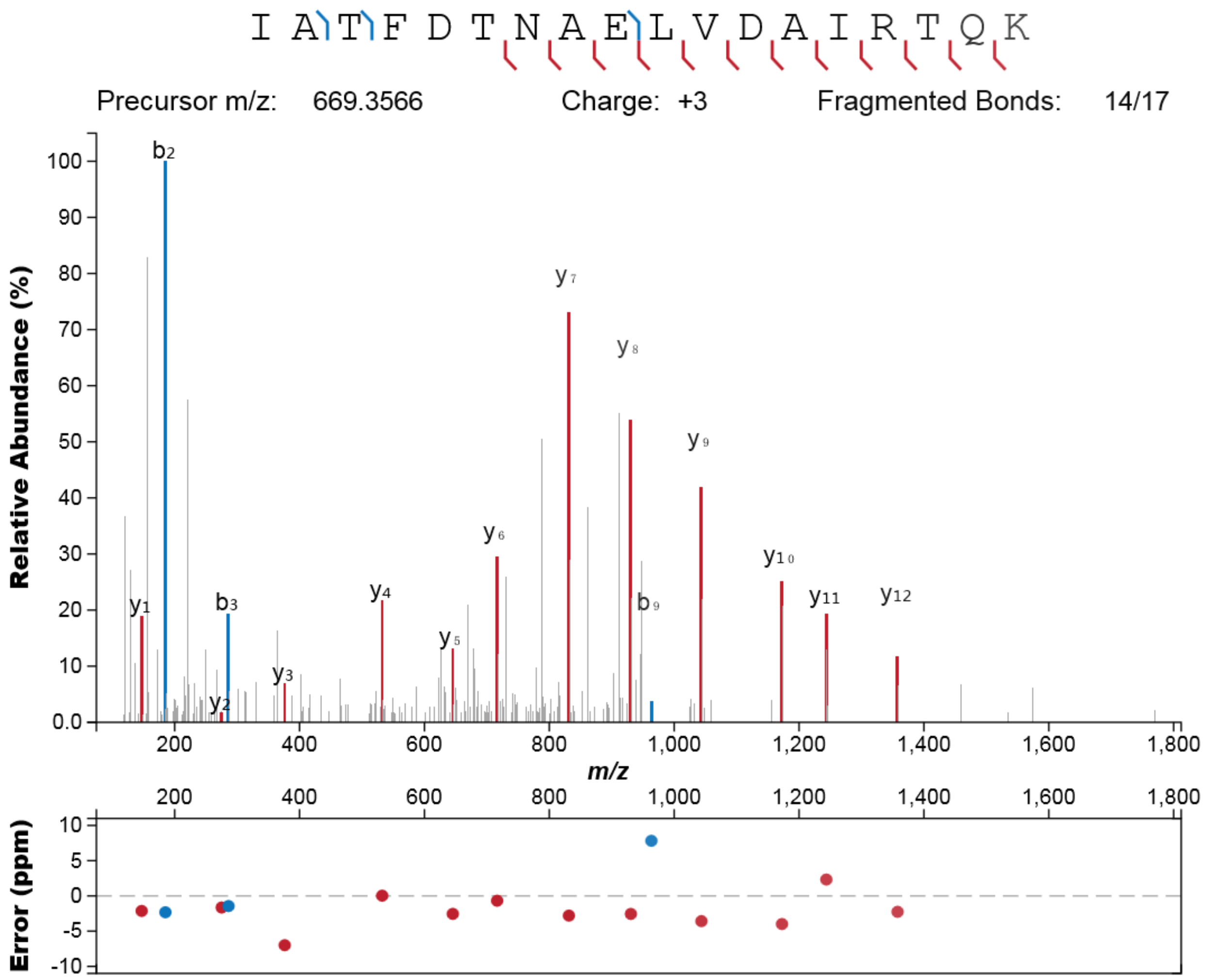
I A T F D T N A E L V D A I R T Q K
Precursor m/z: 669.3566
Charge: +3
Fragmented Bonds: 14/17
Relative Abundance (%)
m/z
Error (ppm)
b2
b3
b9
y1
y2
y3
y4
y5
y6
y7
y8
y9
y10
y11
y12

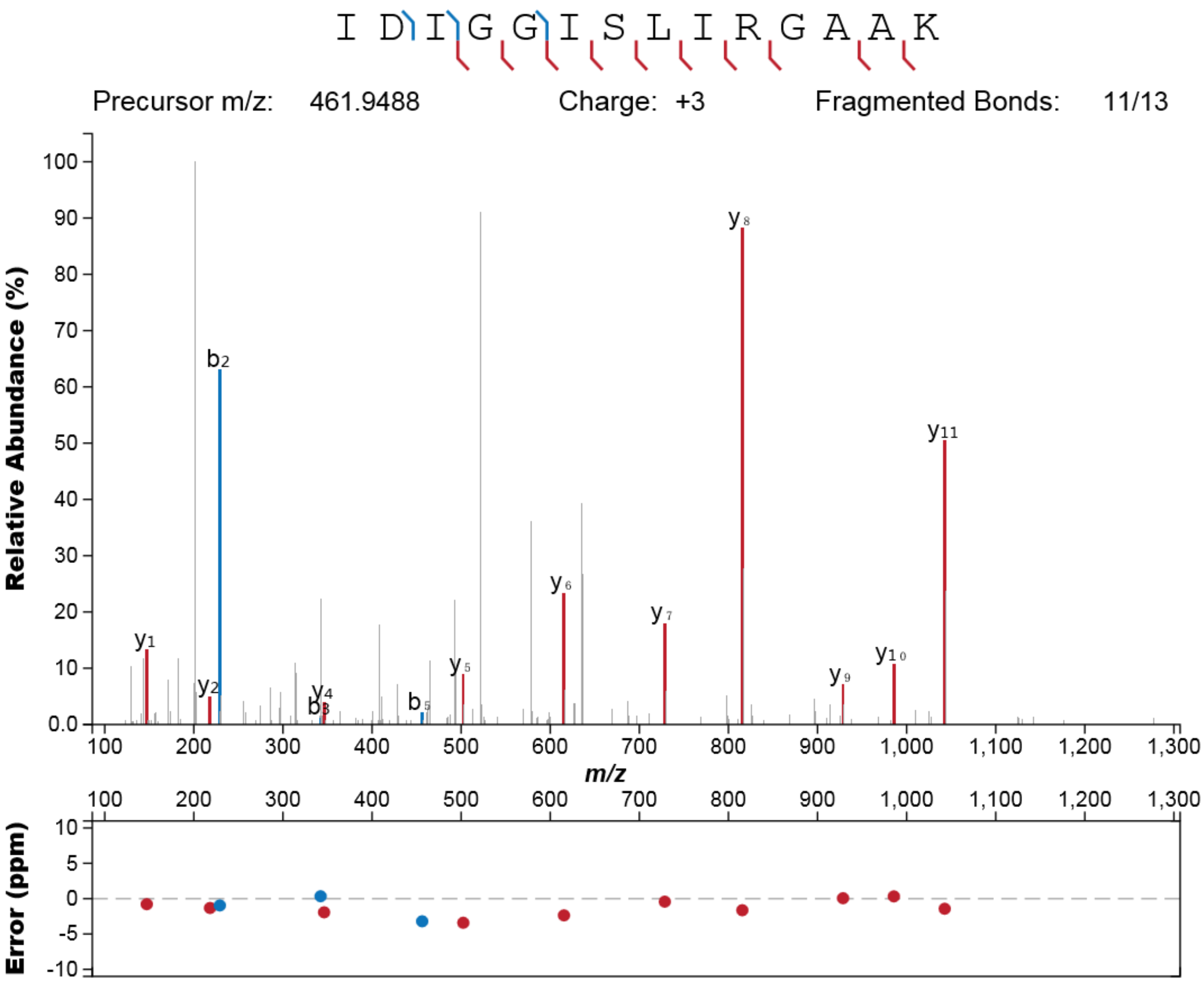
I D I G G I S L I R G A A K
Precursor m/z: 461.9488
Charge: +3
Fragmented Bonds: 11/13
Relative Abundance (%)
m/z
Error (ppm)
b2
b3
b5
y1
y2
y4
y5
y6
y7
y8
y9
y10
y11

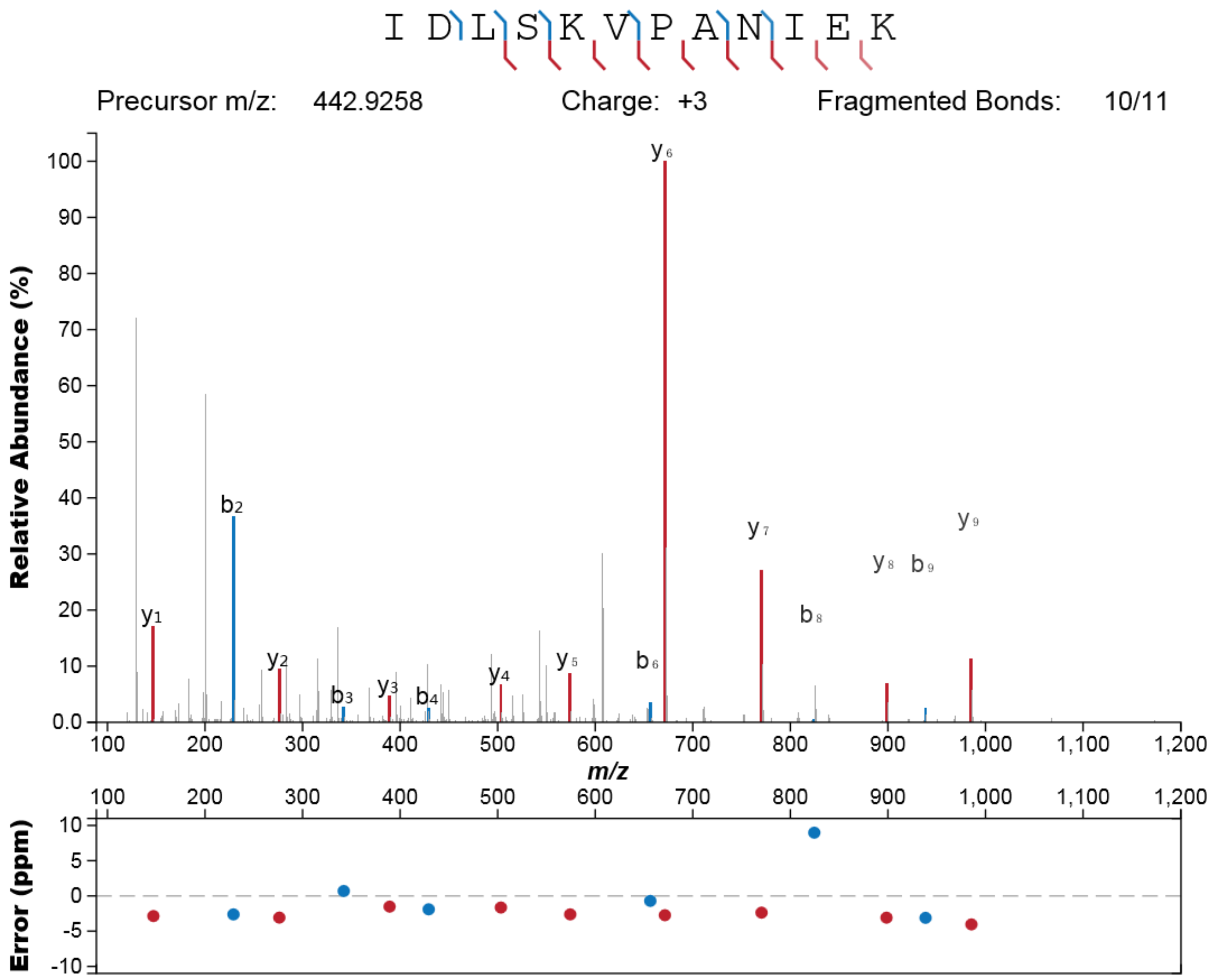

I D L S K V P A N I E K
Precursor m/z: 442.9258
Charge: +3
Fragmented Bonds: 10/11
Relative Abundance (%)
m/z
Error (ppm)
y1
b2
y2
b3
y3
b4
y4
y5
b6
y6
y7
b8
y8
b9
y9

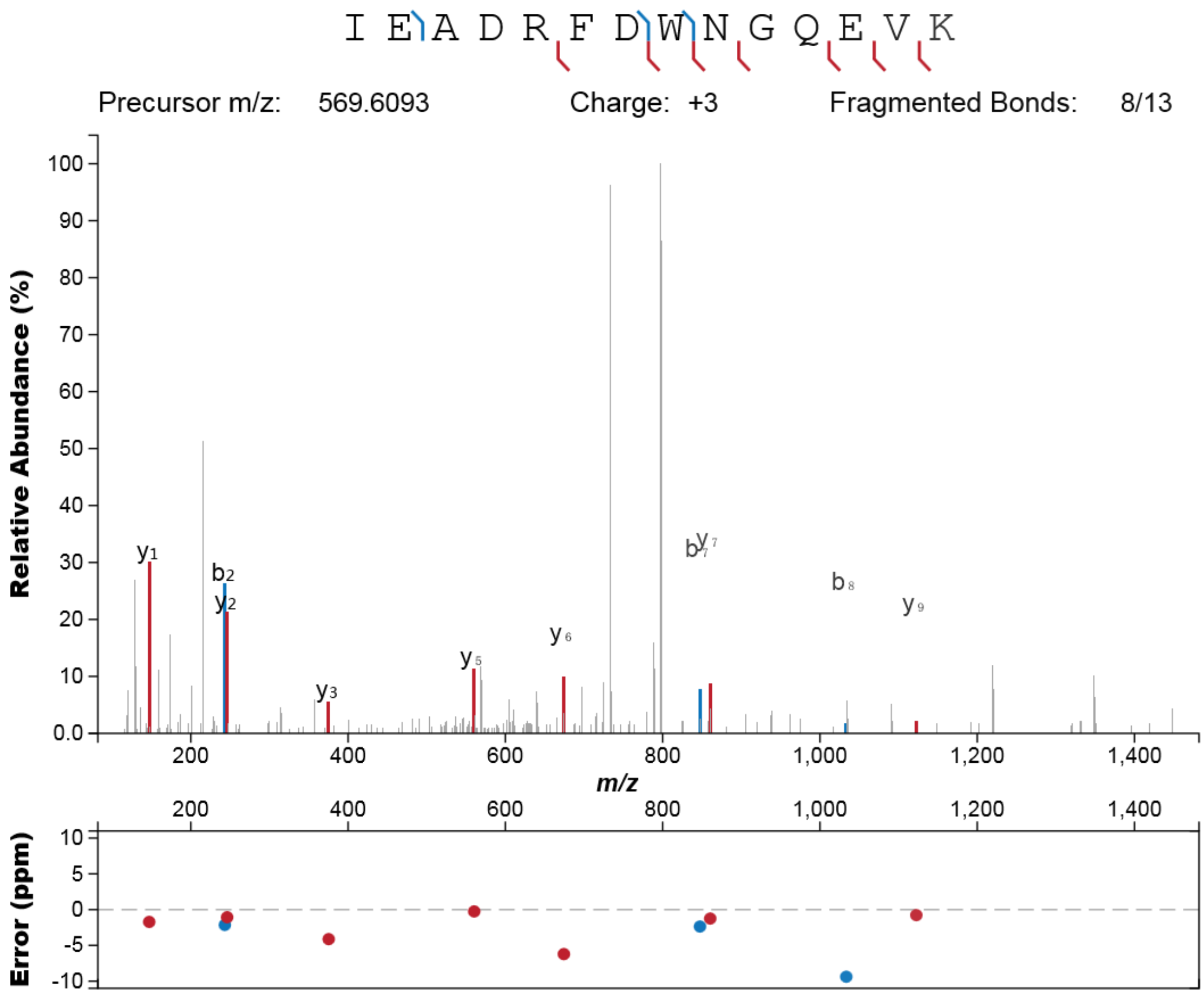
I E A D R F D W N G Q E V K
Precursor m/z: 569.6093
Charge: +3
Fragmented Bonds: 8/13
Relative Abundance (%)
m/z
Error (ppm)
y1
b2
y2
y3
y5
y6
b7
y7
b8
y9

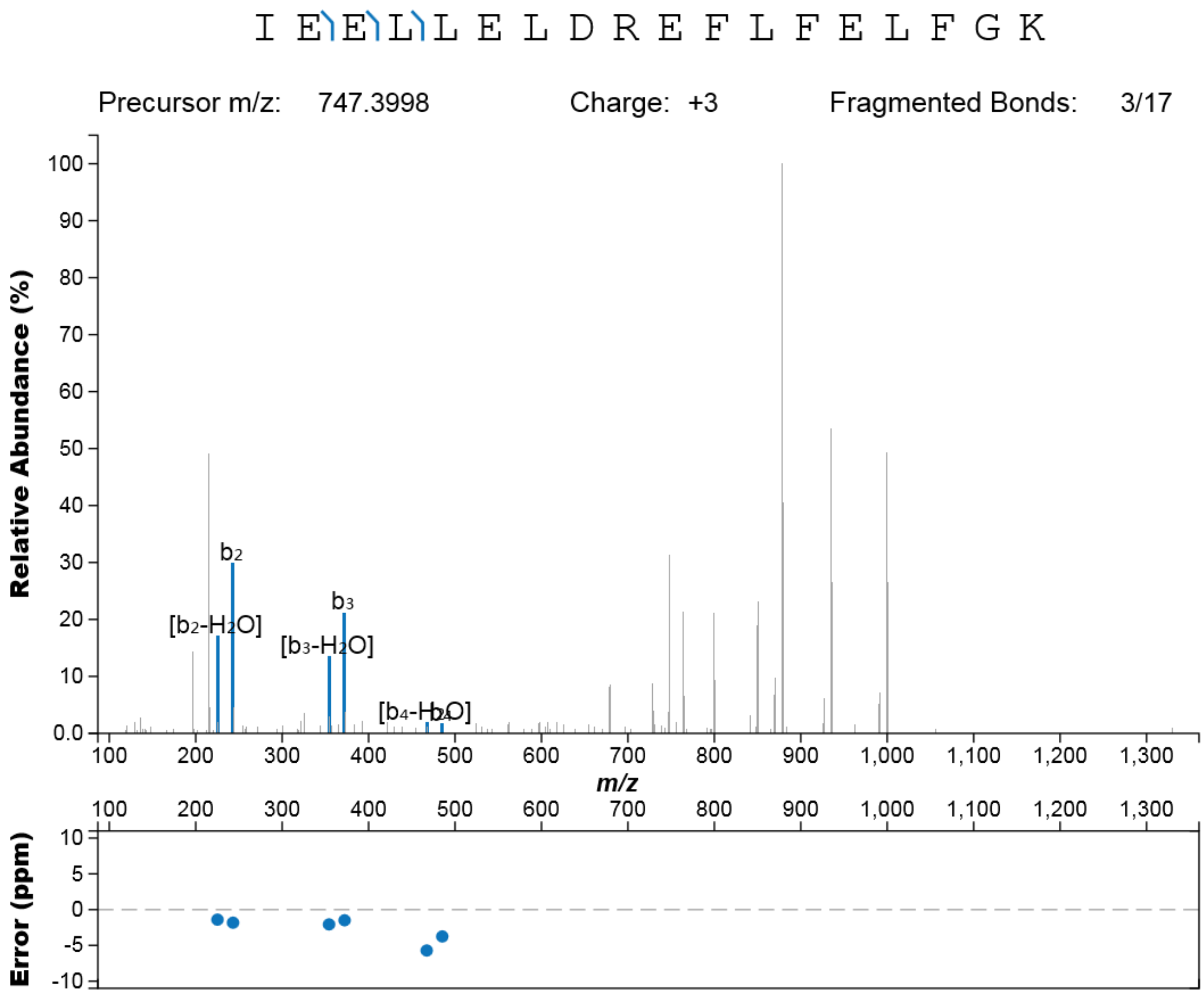

I E E L L E L D R E F L F E L F G K
Precursor m/z: 747.3998
Charge: +3
Fragmented Bonds: 3/17
Relative Abundance (%)
b2
[b2-H2O]
b3
[b3-H2O]
[b4-H2O]
b4
m/z
Error (ppm)

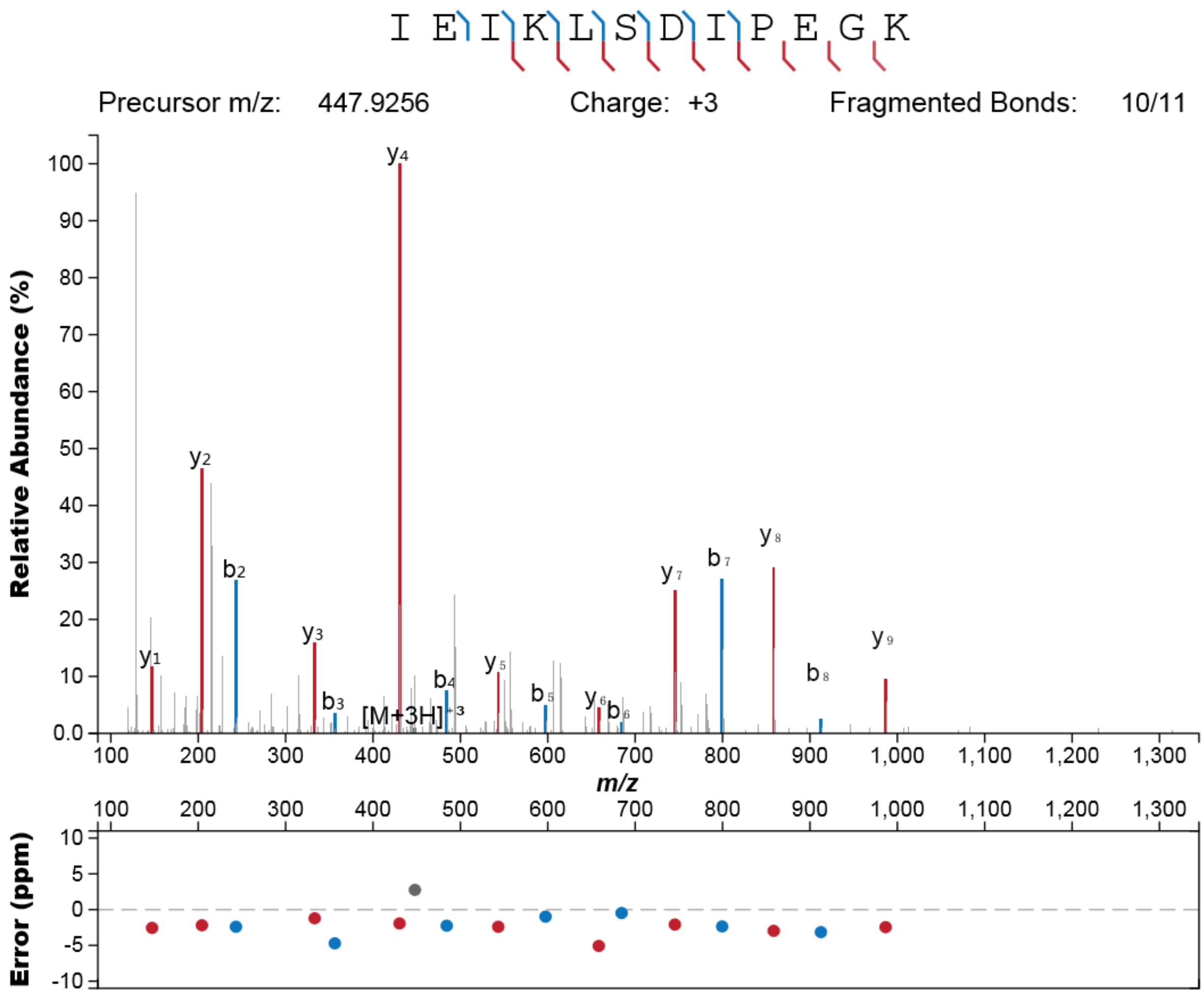
I E I K L S D I P E G K
Precursor m/z: 447.9256
Charge: +3
Fragmented Bonds: 10/11
Relative Abundance (%)
m/z
Error (ppm)
y1
y2
b2
y3
b3
[M+3H]+3
y4
b4
y5
b5
y6
b6
y7
b7
y8
b8
y9

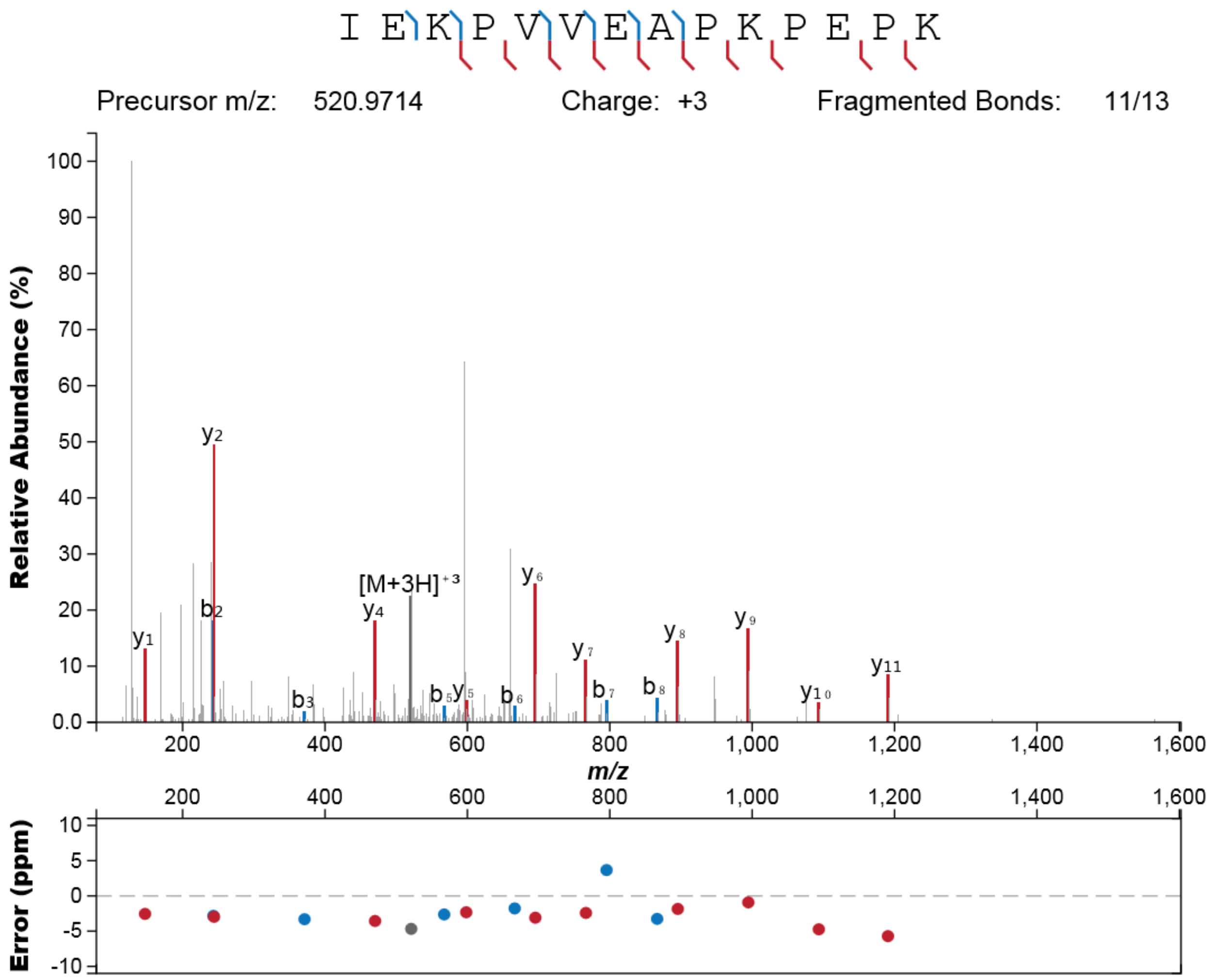

I E K P V V E A P K P E P K
Precursor m/z: 520.9714
Charge: +3
Fragmented Bonds: 11/13
Relative Abundance (%)
m/z
Error (ppm)
[M+3H]+3
y1
y2
b2
b3
y4
b5
y5
b6
y6
y7
b7
b8
y8
y9
y10
y11

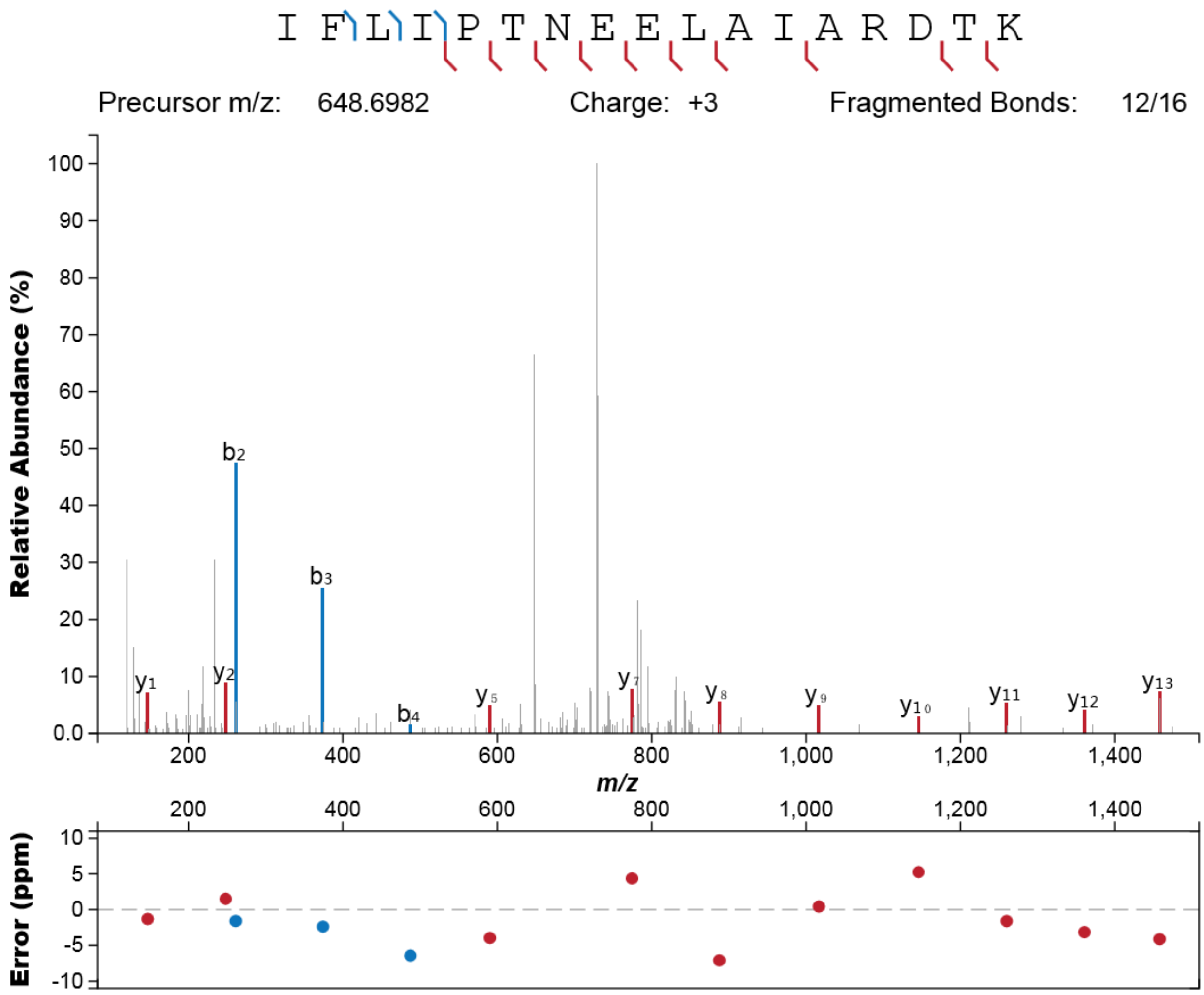
I F L I P T N E E L A I A R D T K
Precursor m/z: 648.6982
Charge: +3
Fragmented Bonds: 12/16
Relative Abundance (%)
m/z
Error (ppm)
b2
b3
b4
y1
y2
y5
y7
y8
y9
y10
y11
y12
y13

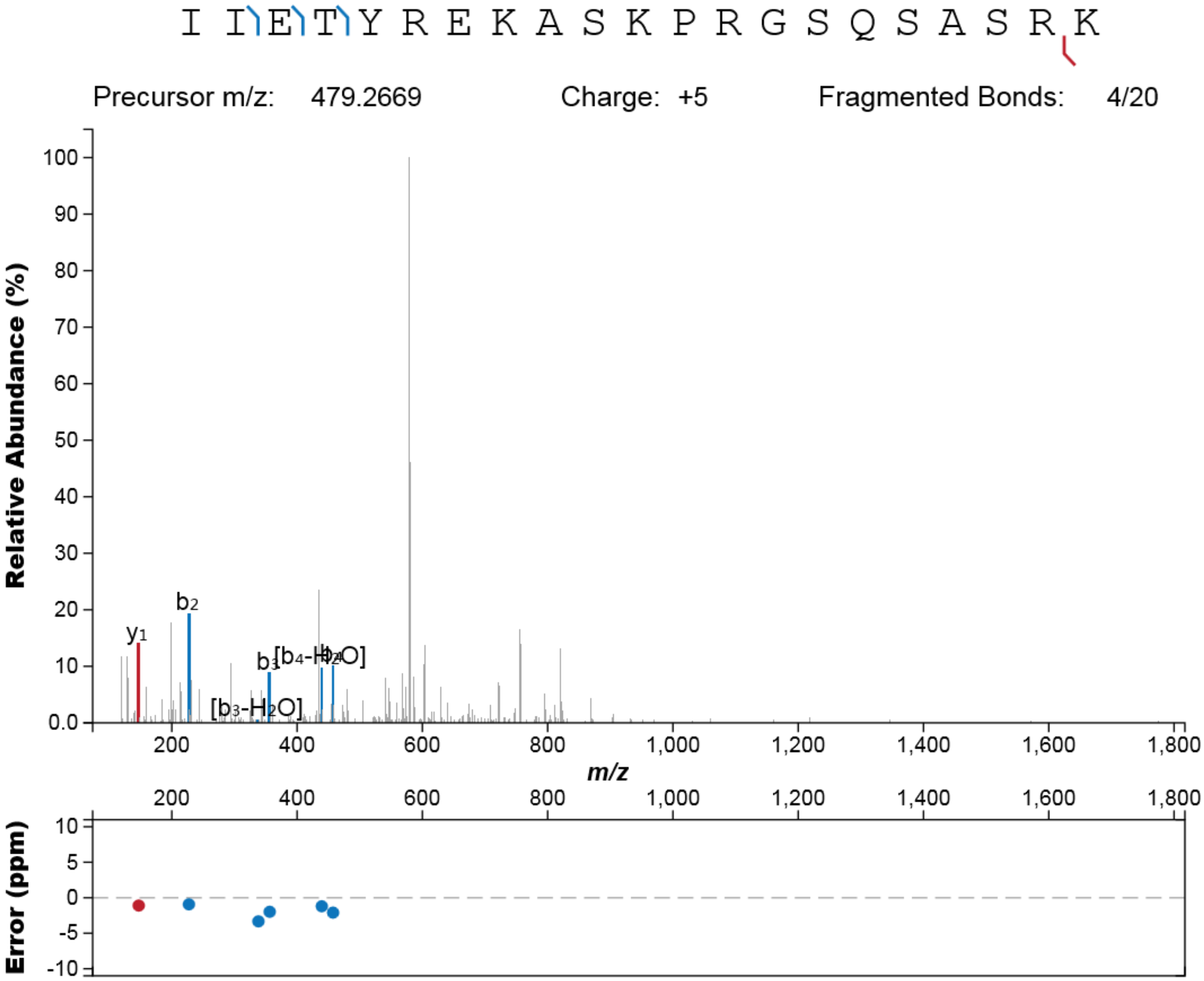
I I E T Y R E K A S K P R G S Q S A S R K
Precursor m/z: 479.2669
Charge: +5
Fragmented Bonds: 4/20
Relative Abundance (%)
y1
b2
b3
[b3-H2O]
[b4-H2O]
b4
m/z
Error (ppm)

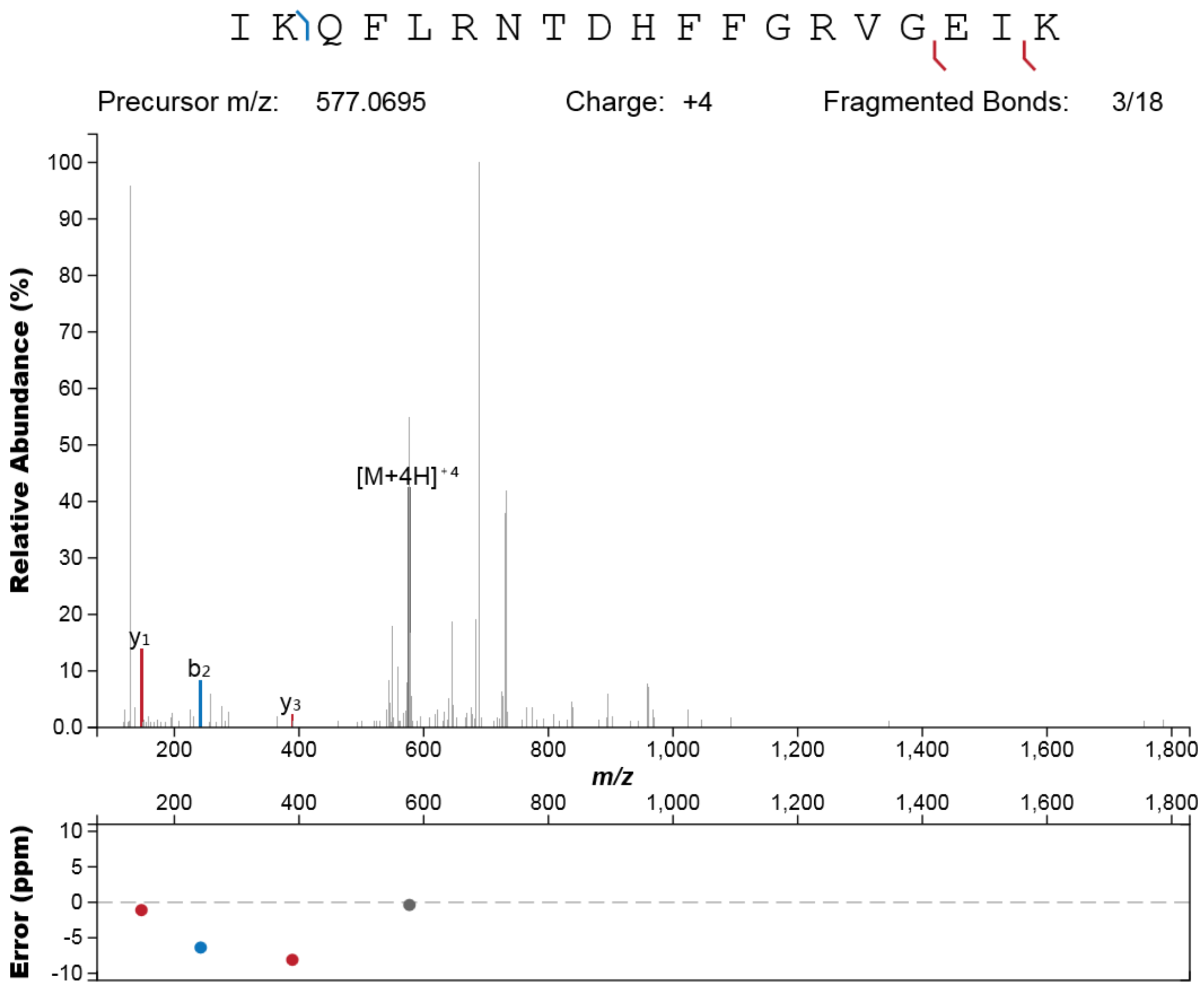

I K Q F L R N T D H F F G R V G E I K
Precursor m/z: 577.0695
Charge: +4
Fragmented Bonds: 3/18
Relative Abundance (%)
y1
b2
y3
[M+4H]+4
m/z
Error (ppm)

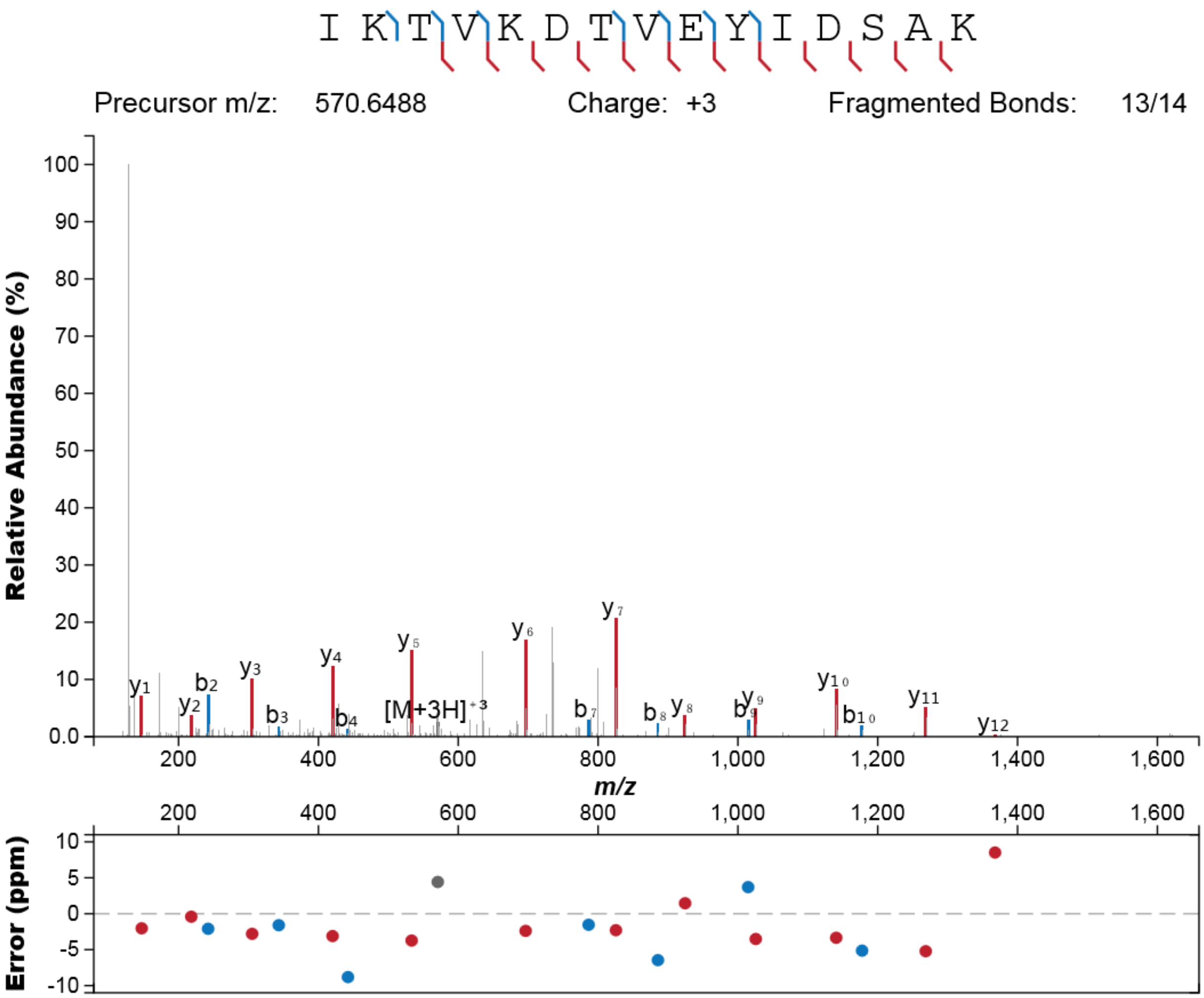

I K T V K D T V E Y I D S A K
Precursor m/z: 570.6488
Charge: +3
Fragmented Bonds: 13/14
Relative Abundance (%)
m/z
Error (ppm)
[M+3H]+3
y1
y2
y3
y4
y5
y6
y7
y8
y9
y10
y11
y12
b2
b3
b4
b7
b8
b9
b10

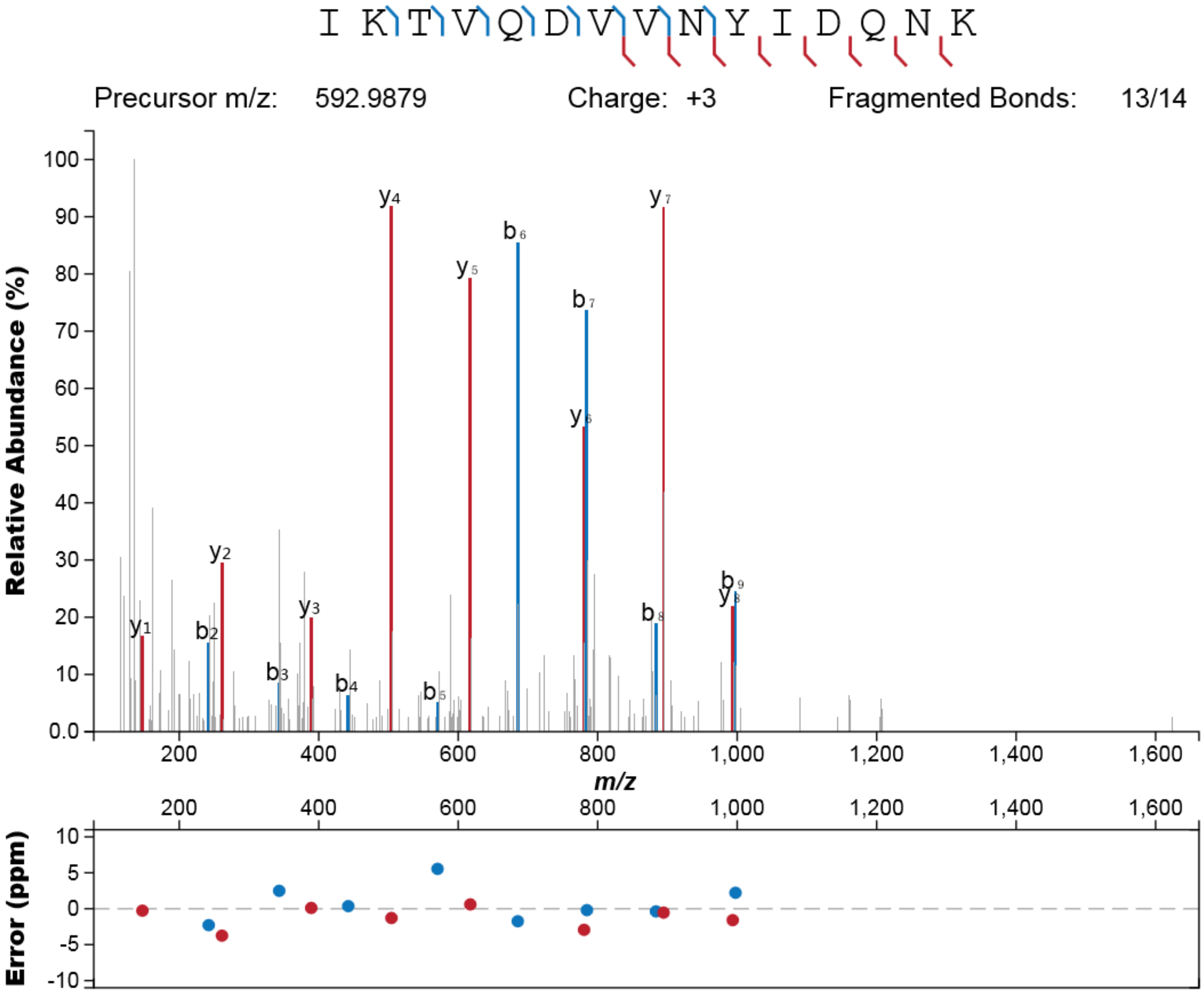

I K T V Q D V V N Y I D Q N K
Precursor m/z: 592.9879
Charge: +3
Fragmented Bonds: 13/14
Relative Abundance (%)
m/z
Error (ppm)
y1
b2
y2
b3
y3
b4
y4
b5
y5
b6
y6
b7
y7
b8
y8
b9

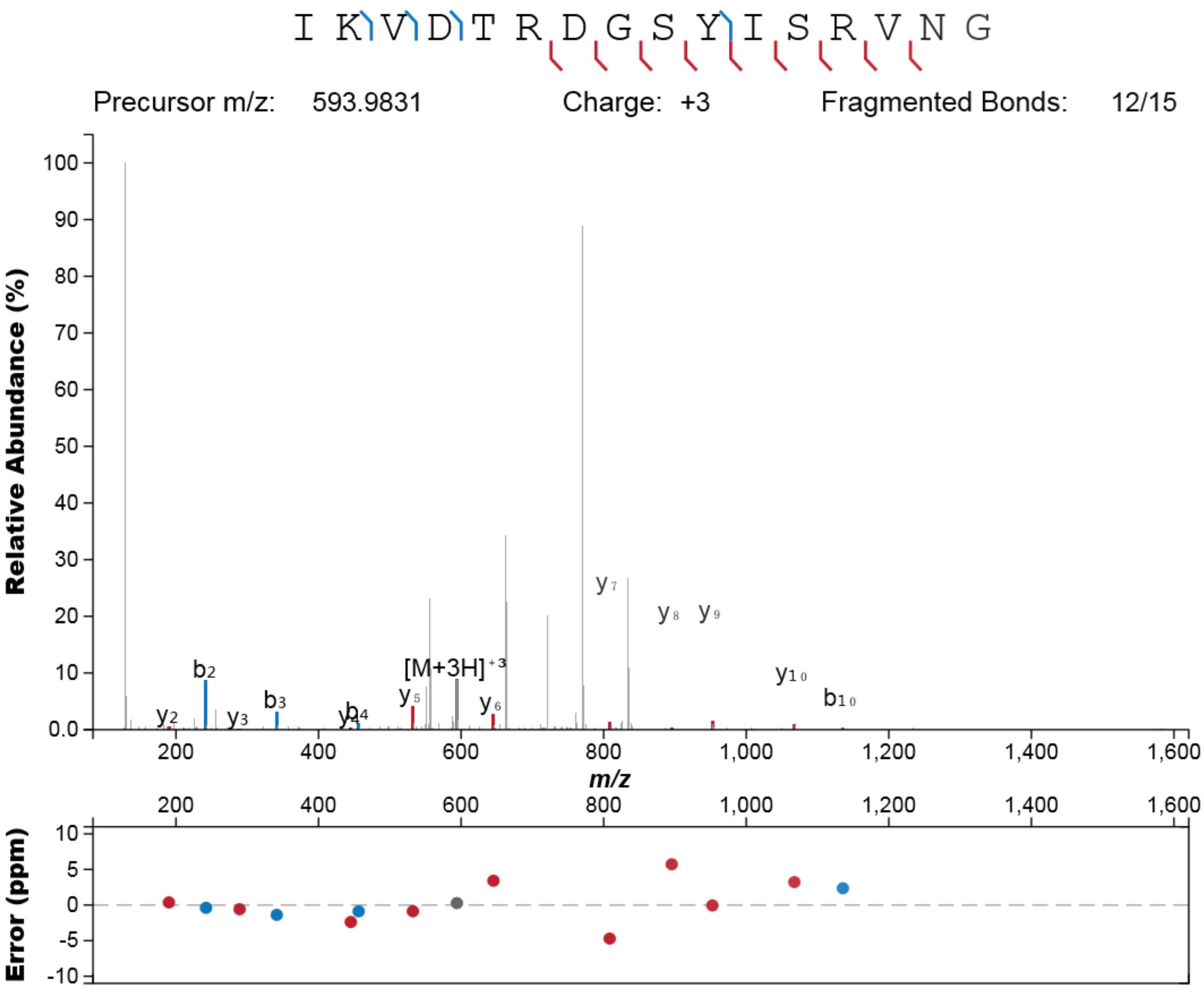

I K V D T R D G S Y I S R V N G
Precursor m/z: 593.9831
Charge: +3
Fragmented Bonds: 12/15
Relative Abundance (%)
m/z
Error (ppm)
b2
b3
b4
b10
y2
y3
y5
y6
y7
y8
y9
y10
[M+3H]+3

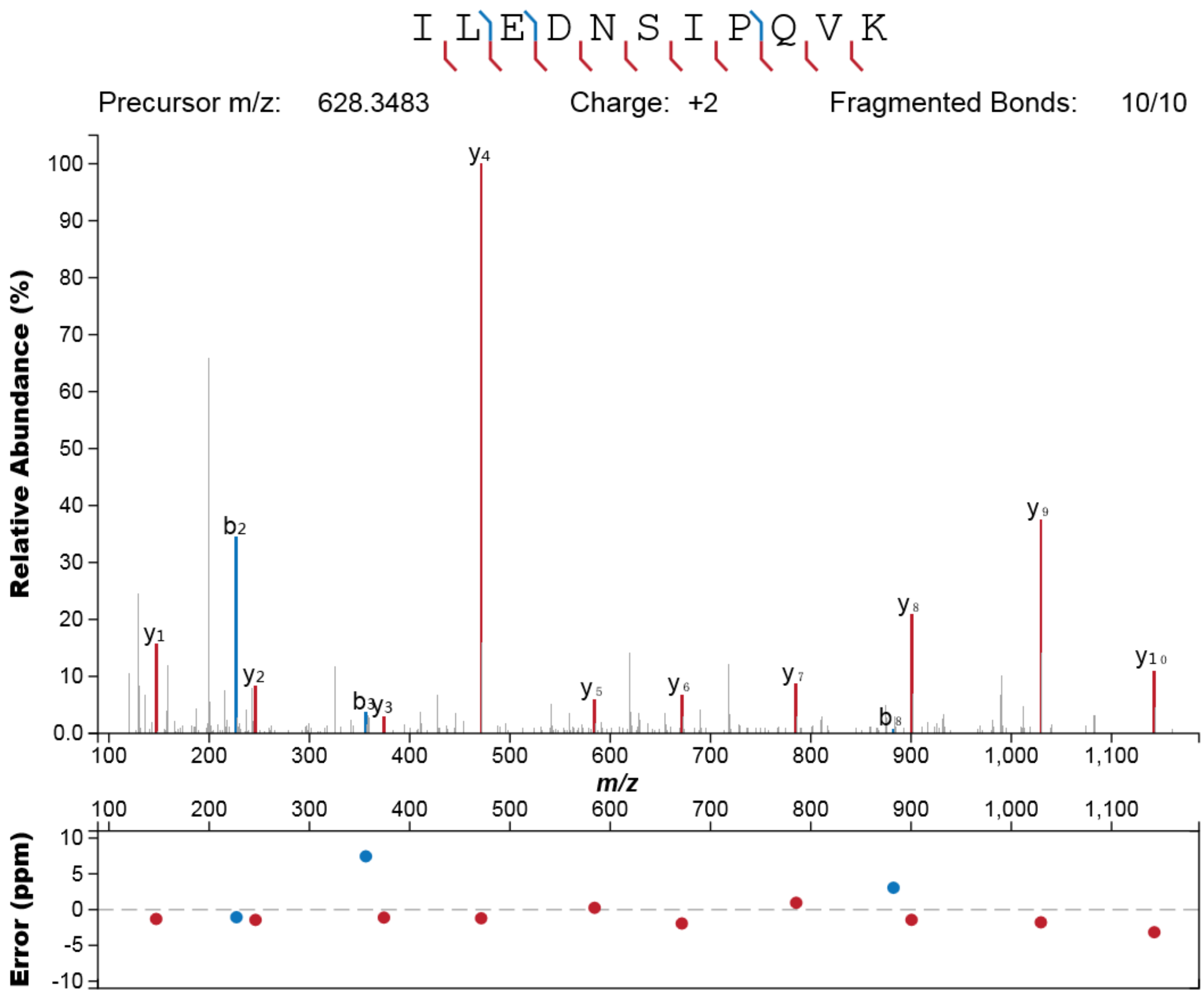
I L E D N S I P Q V K
Precursor m/z: 628.3483
Charge: +2
Fragmented Bonds: 10/10
Relative Abundance (%)
m/z
Error (ppm)
y1
y2
y3
y4
y5
y6
y7
y8
y9
y10
b2
b3
b8

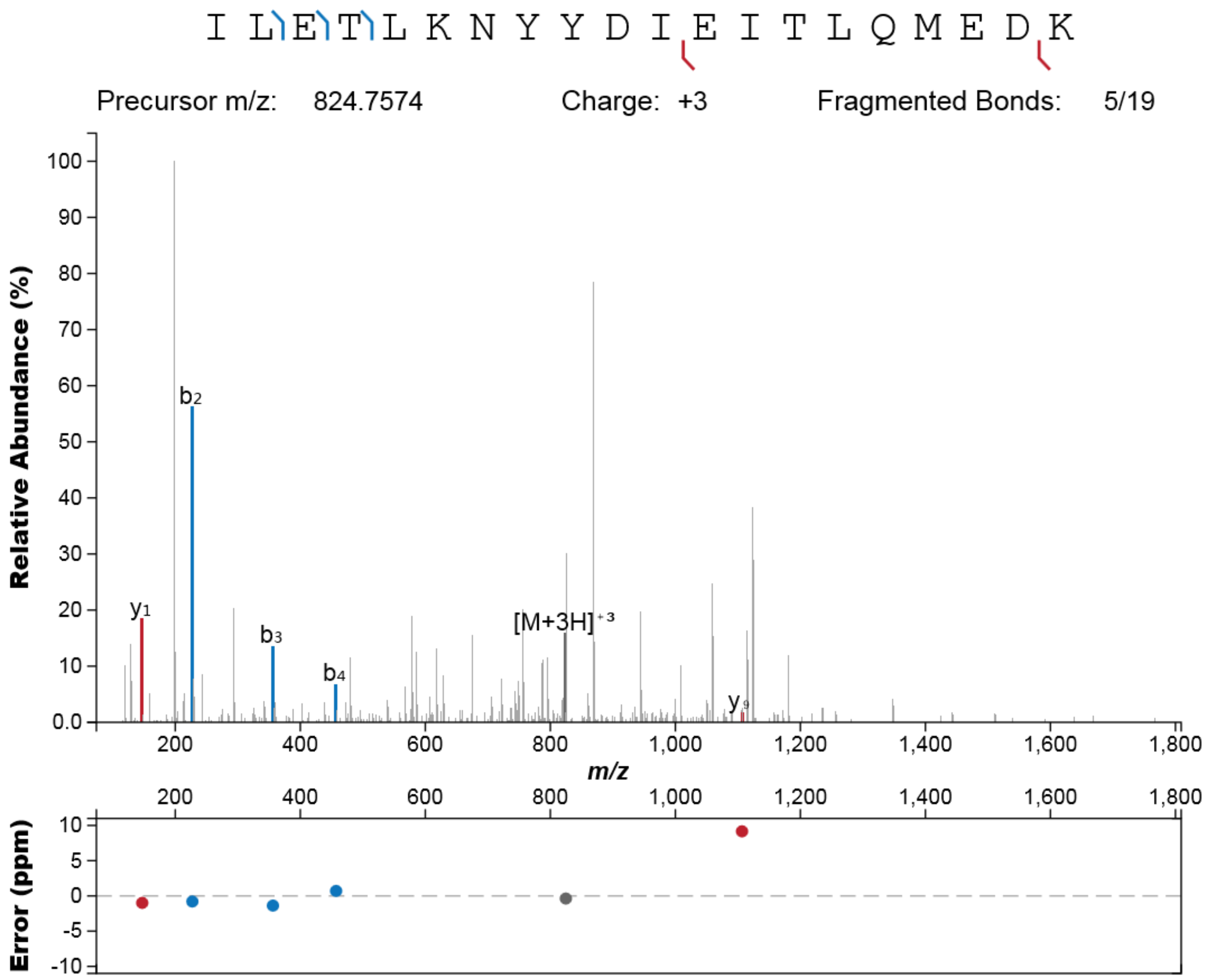

I L E T L K N Y Y D I E I T L Q M E D K
Precursor m/z: 824.7574
Charge: +3
Fragmented Bonds: 5/19
Relative Abundance (%)
y1
b2
b3
b4
[M+3H]+3
y9
m/z
Error (ppm)

I L G L F A m N H m N V Y I D R E I L K

Precursor m/z: 606.3210 Charge: +4 Fragmented Bonds: 2/19

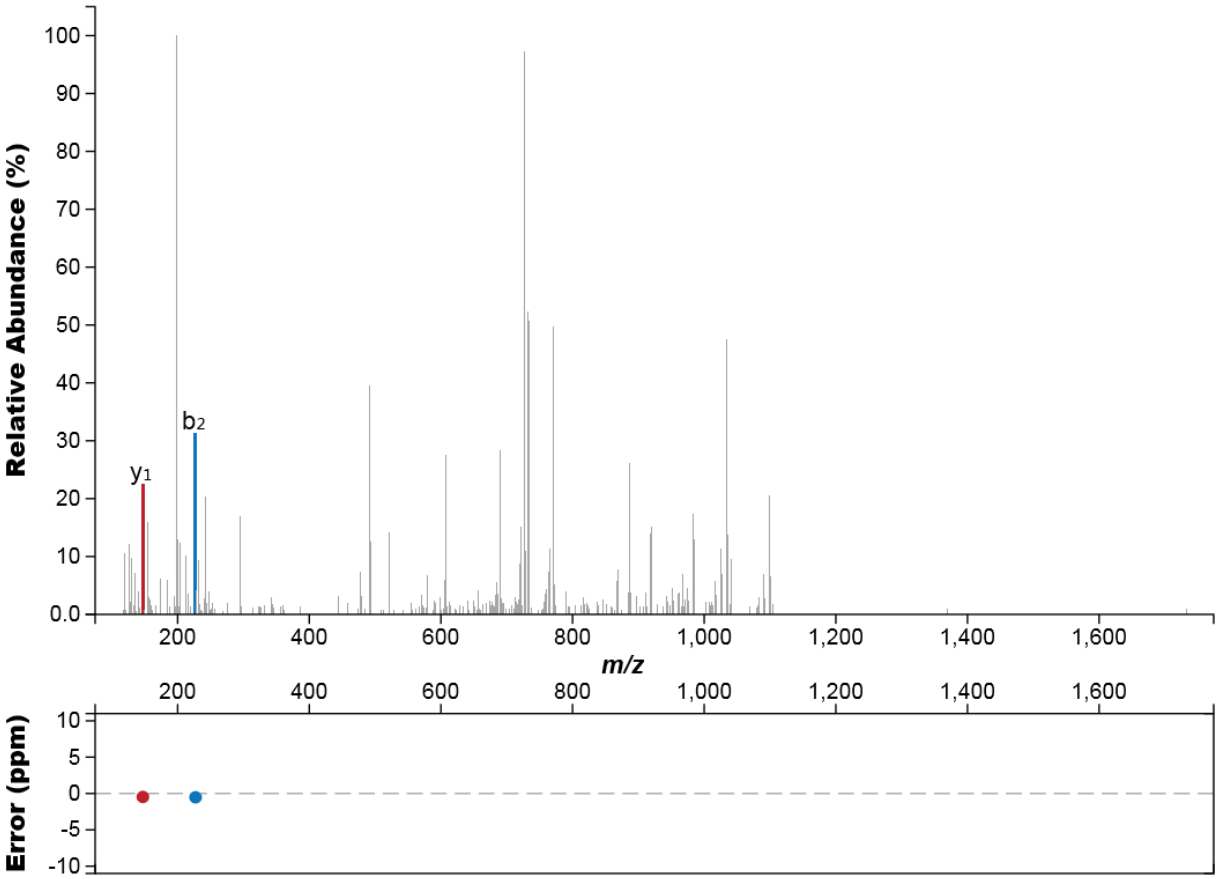

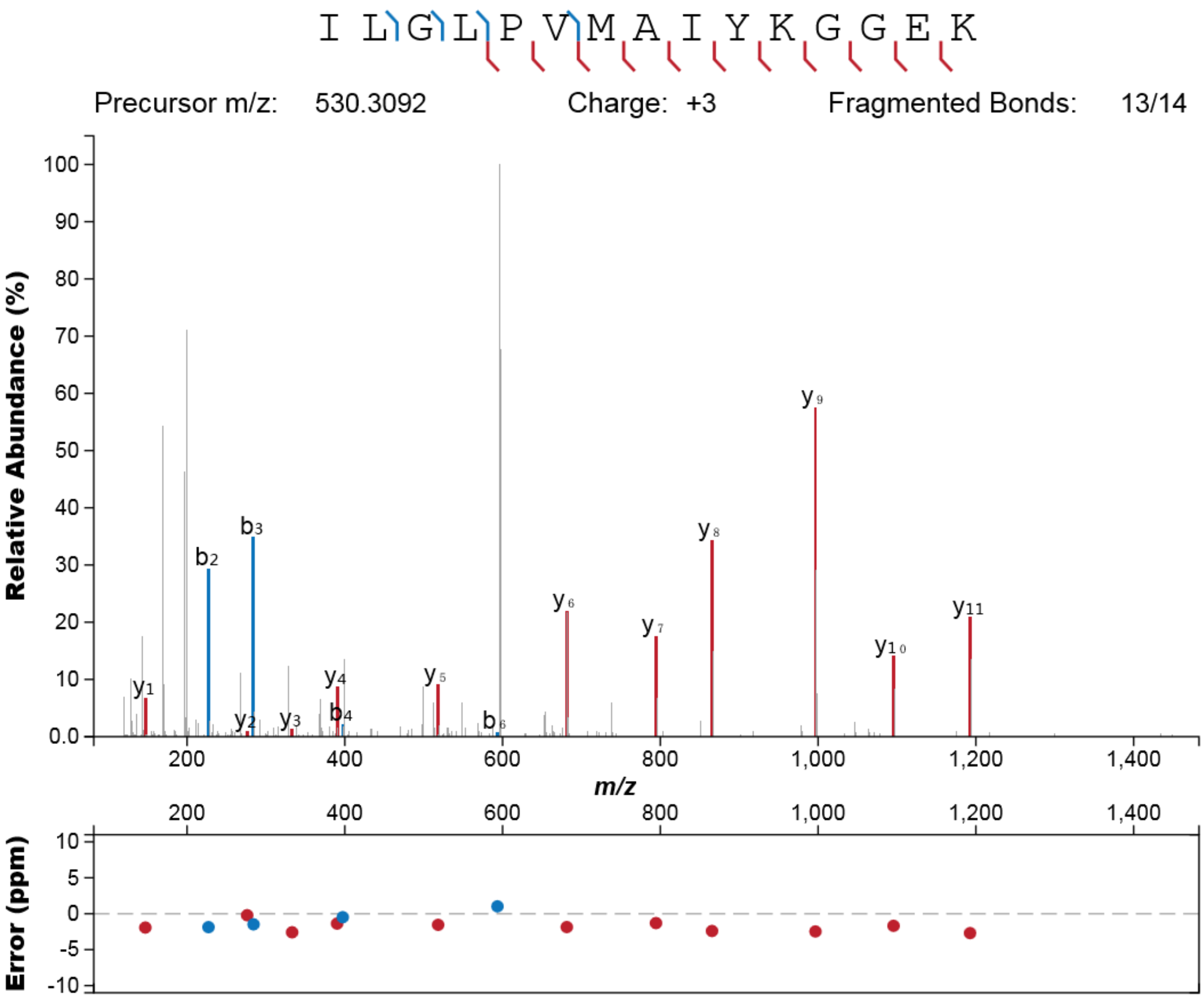

I L G L P V M A I Y K G G E K
Precursor m/z: 530.3092
Charge: +3
Fragmented Bonds: 13/14
Relative Abundance (%)
m/z
Error (ppm)
b2
b3
b4
b6
y1
y2
y3
y4
y5
y6
y7
y8
y9
y10
y11

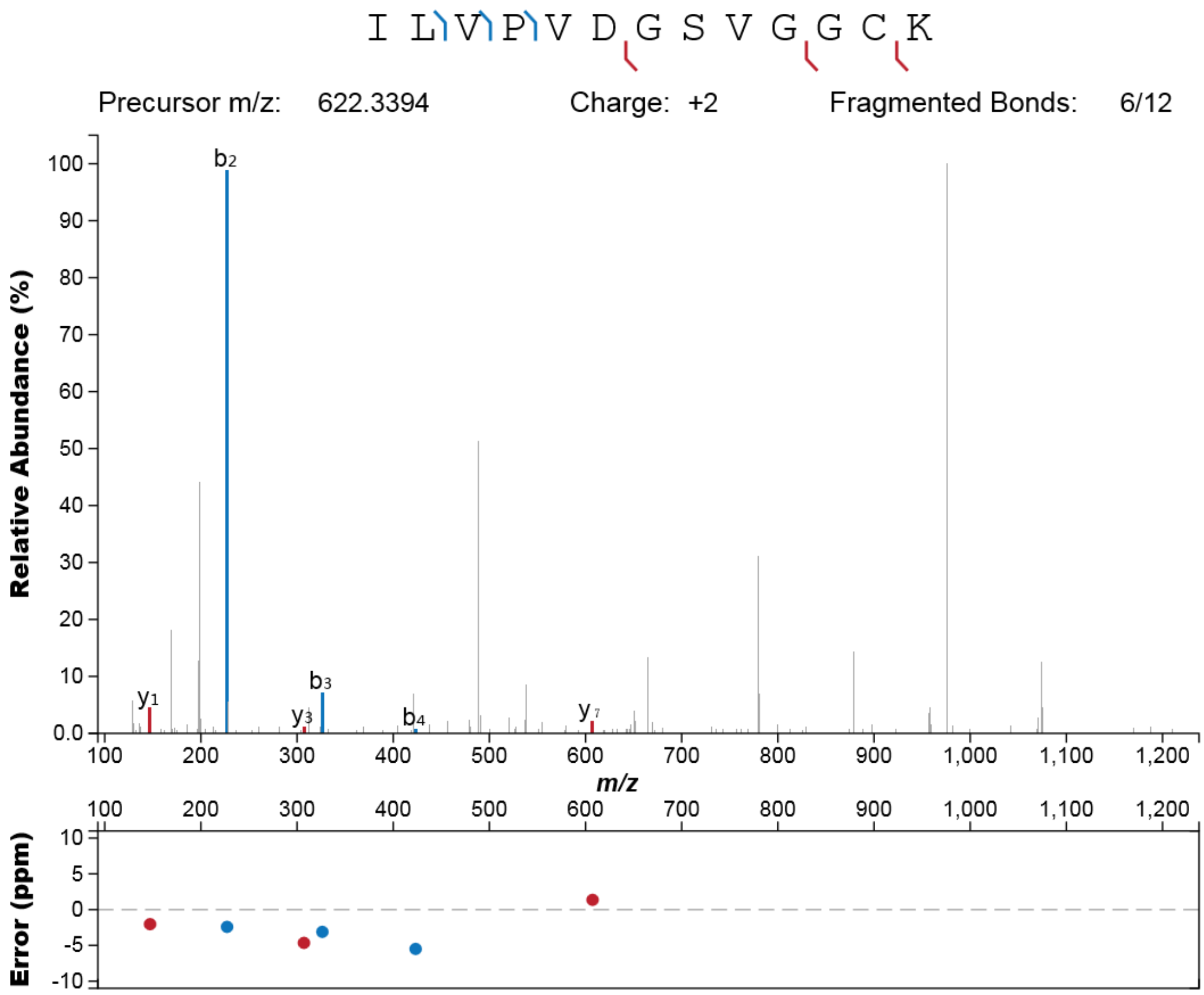

I L V P V D G S V G G C K
Precursor m/z: 622.3394
Charge: +2
Fragmented Bonds: 6/12
Relative Abundance (%)
b2
y1
y3
b3
b4
y7
m/z
Error (ppm)

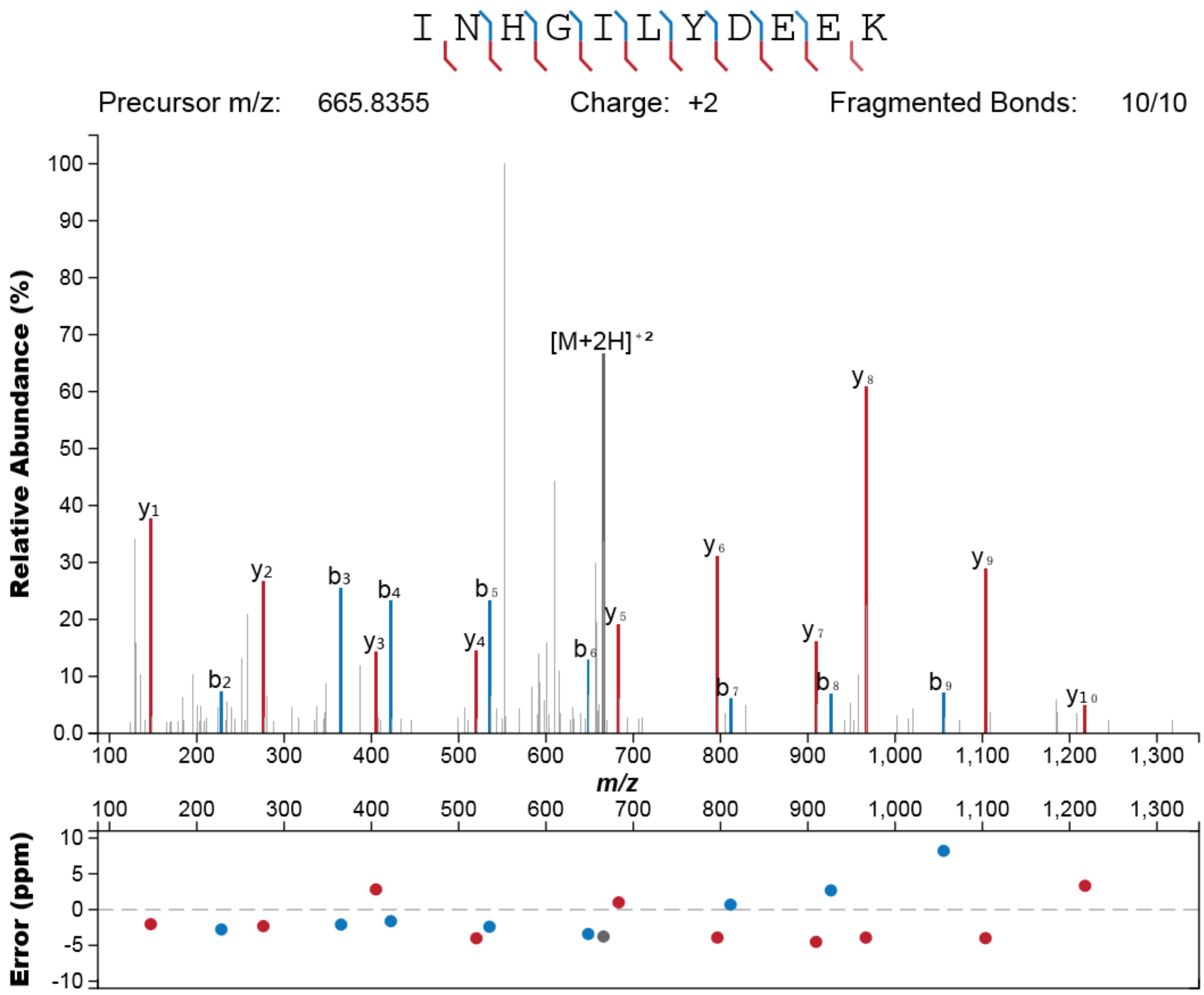

I N H G I L Y D E E K
Precursor m/z: 665.8355
Charge: +2
Fragmented Bonds: 10/10
Relative Abundance (%)
[M+2H]+2
y1
y2
b2
b3
y3
b4
y4
b5
b6
y5
y6
b7
y7
b8
y8
b9
y9
y10
m/z
Error (ppm)

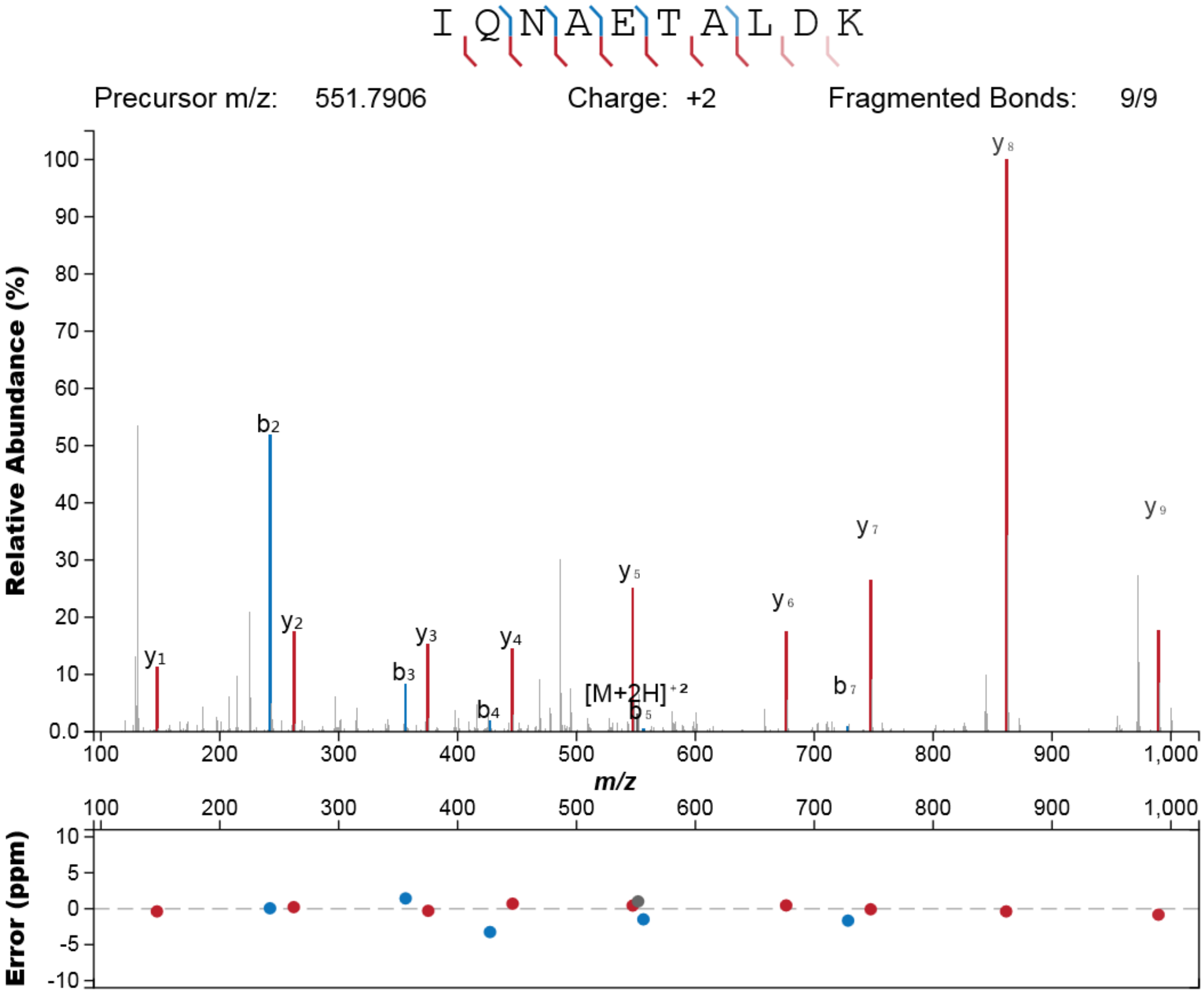

I Q N A E T A L D K
Precursor m/z: 551.7906
Charge: +2
Fragmented Bonds: 9/9
Relative Abundance (%)
m/z
Error (ppm)
y1
b2
y2
b3
y3
b4
y4
[M+2H]+2
b5
y5
y6
b7
y7
y8
y9

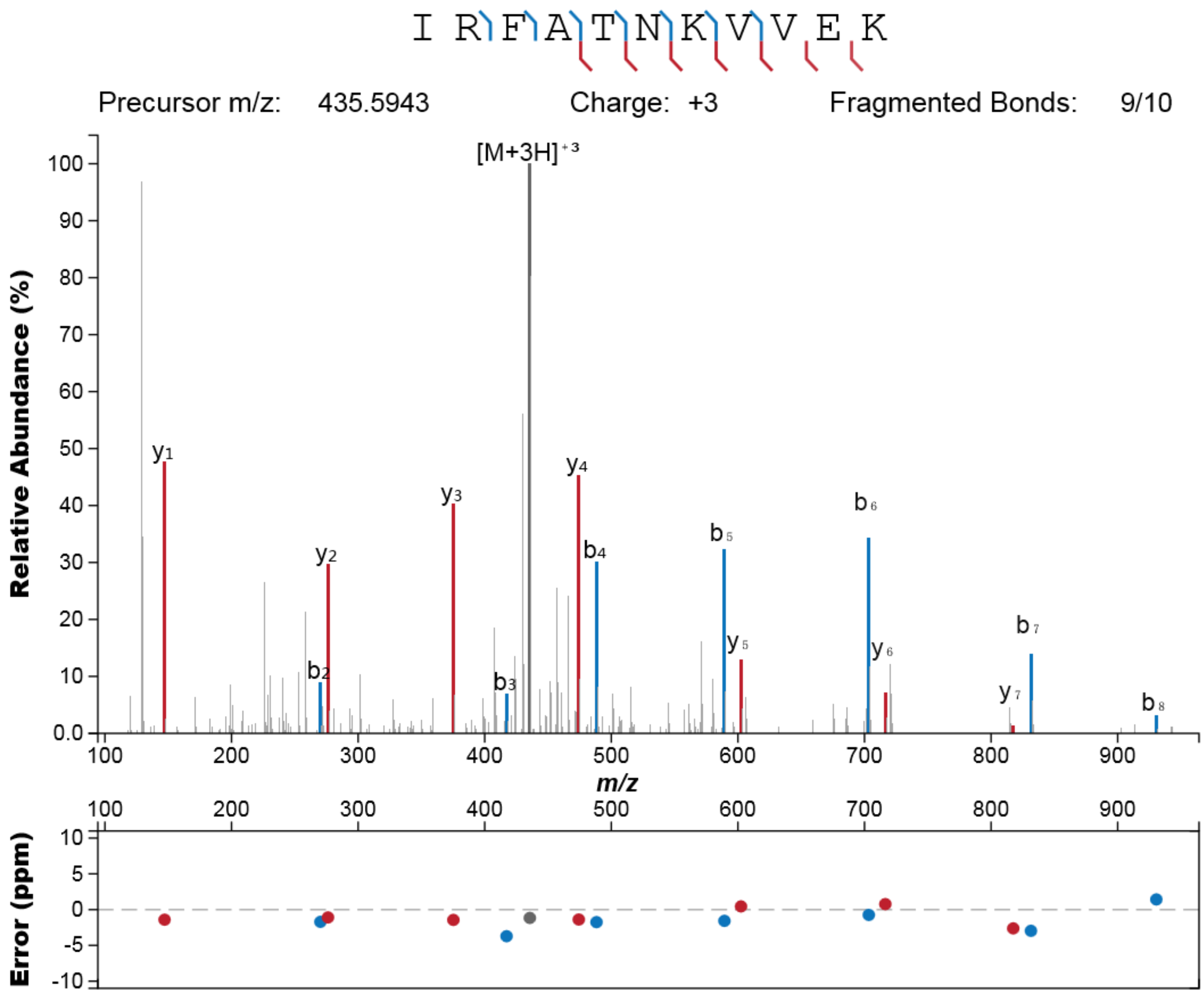
I R F A T N K V V E K
Precursor m/z: 435.5943
Charge: +3
Fragmented Bonds: 9/10
[M+3H]+3
Relative Abundance (%)
m/z
Error (ppm)

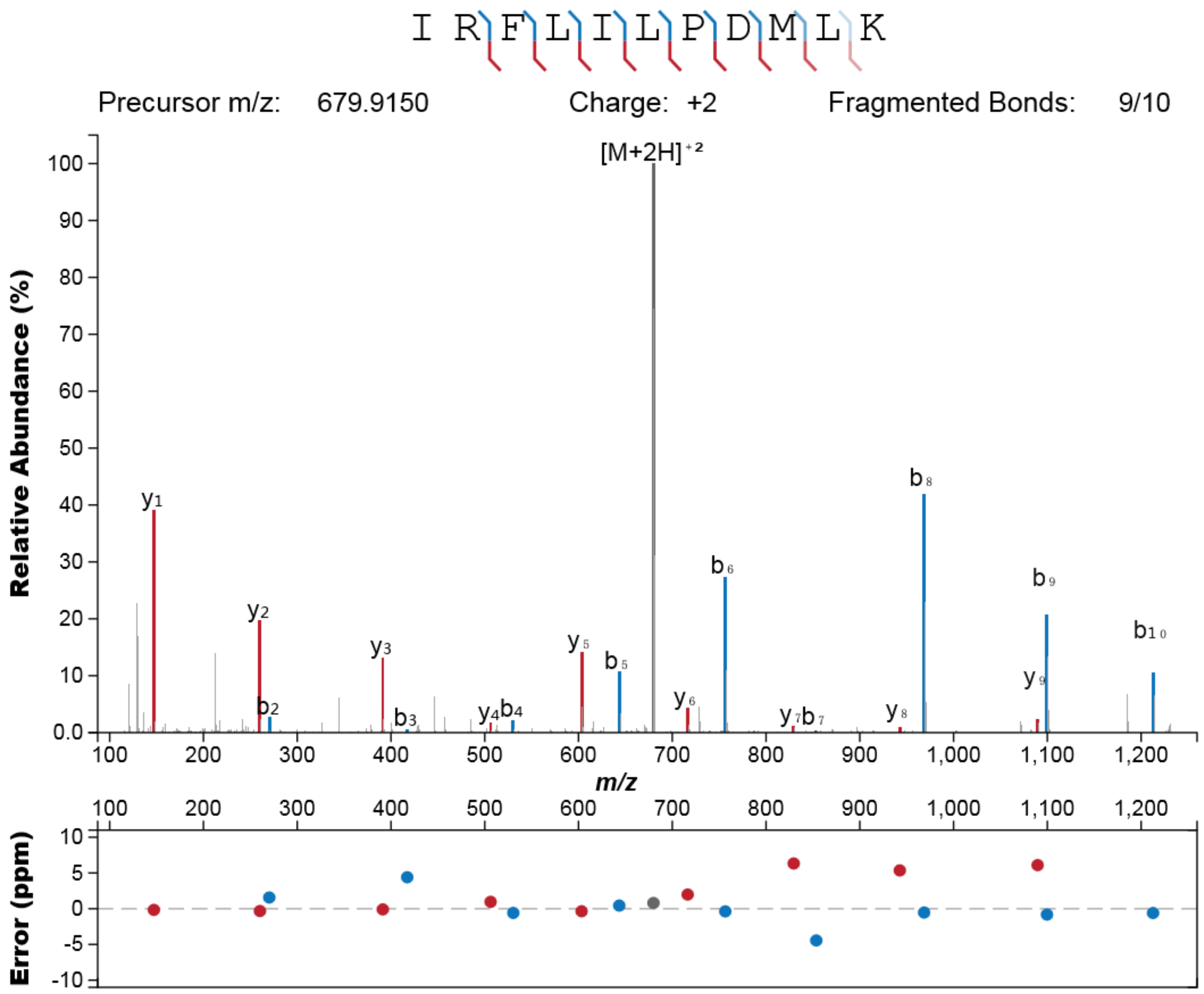
I R F L I L P D M L K
Precursor m/z: 679.9150
Charge: +2
Fragmented Bonds: 9/10
[M+2H]+2
Relative Abundance (%)
m/z
Error (ppm)
y1
y2
b2
y3
b3
y4
b4
y5
b5
y6
b6
y7
b7
y8
b8
y9
b9
b10

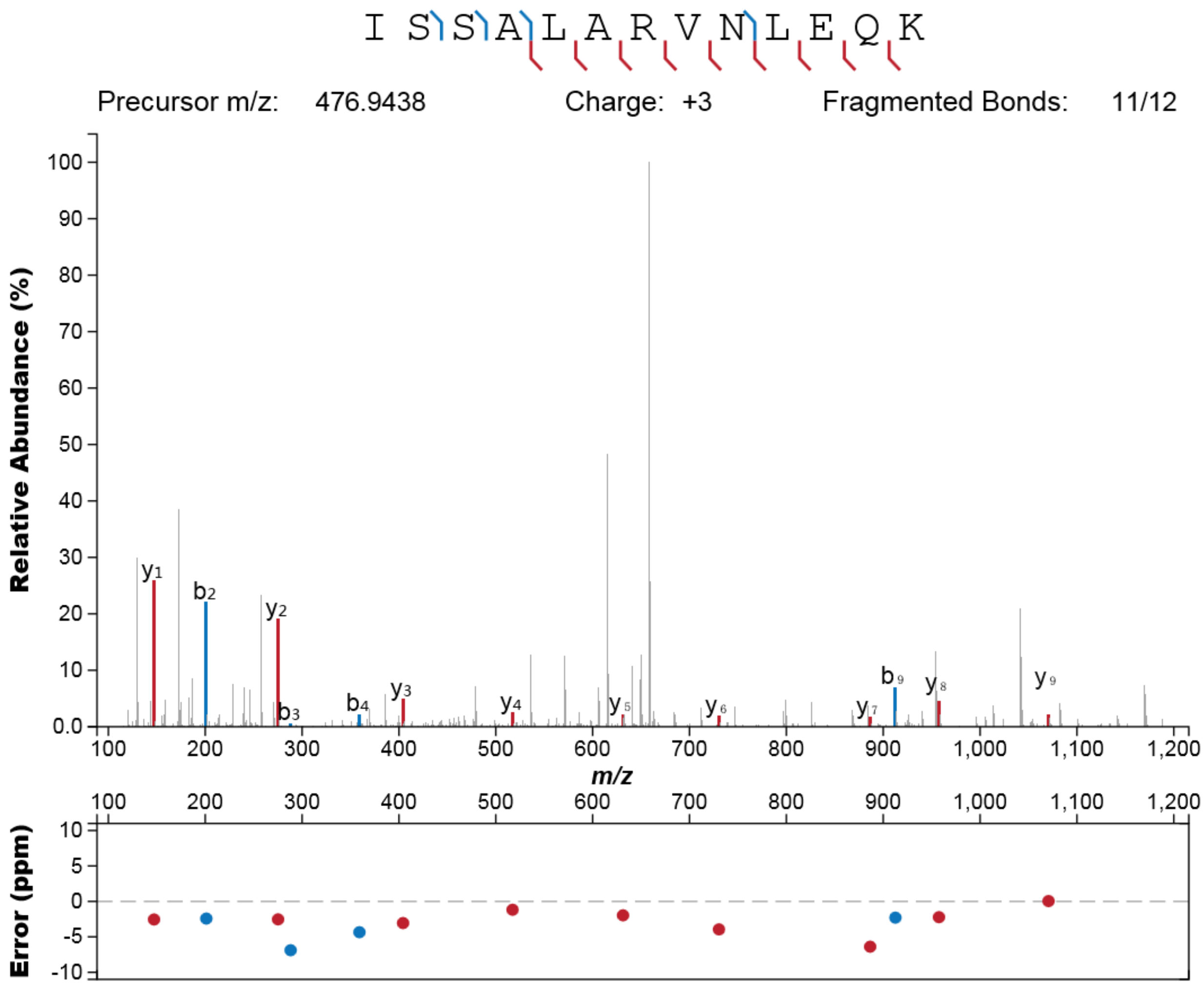

I S S A L A R V N L E Q K
Precursor m/z: 476.9438
Charge: +3
Fragmented Bonds: 11/12
Relative Abundance (%)
m/z
Error (ppm)
y1
b2
y2
b3
b4
y3
y4
y5
y6
y7
b9
y8
y9

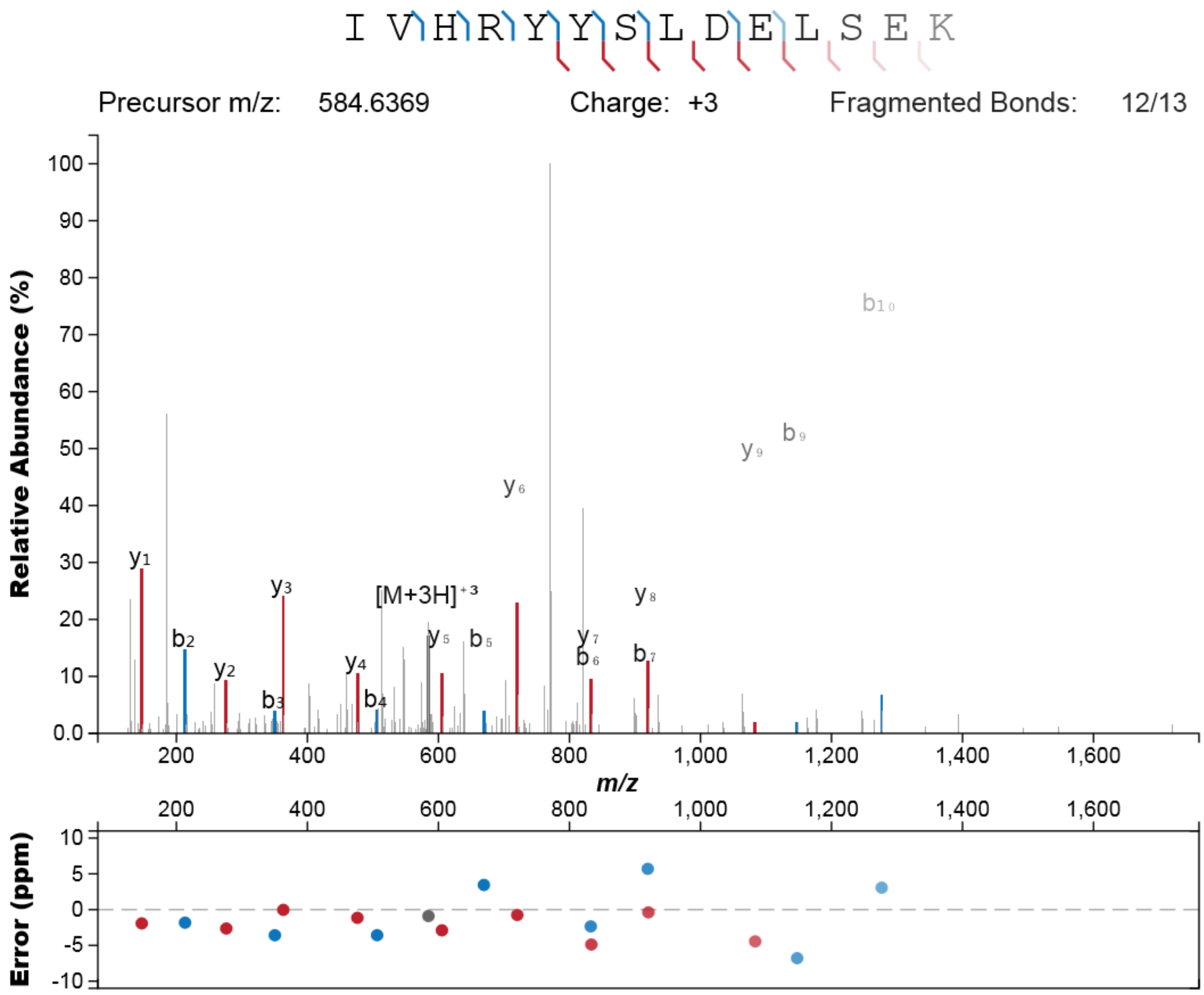

I V H R Y Y S L D E L S E K
Precursor m/z: 584.6369
Charge: +3
Fragmented Bonds: 12/13
Relative Abundance (%)
m/z
Error (ppm)
y1
b2
y2
b3
y3
y4
b4
[M+3H]+3
y5
b5
y6
y7
b6
y8
b7
y9
b9
b10

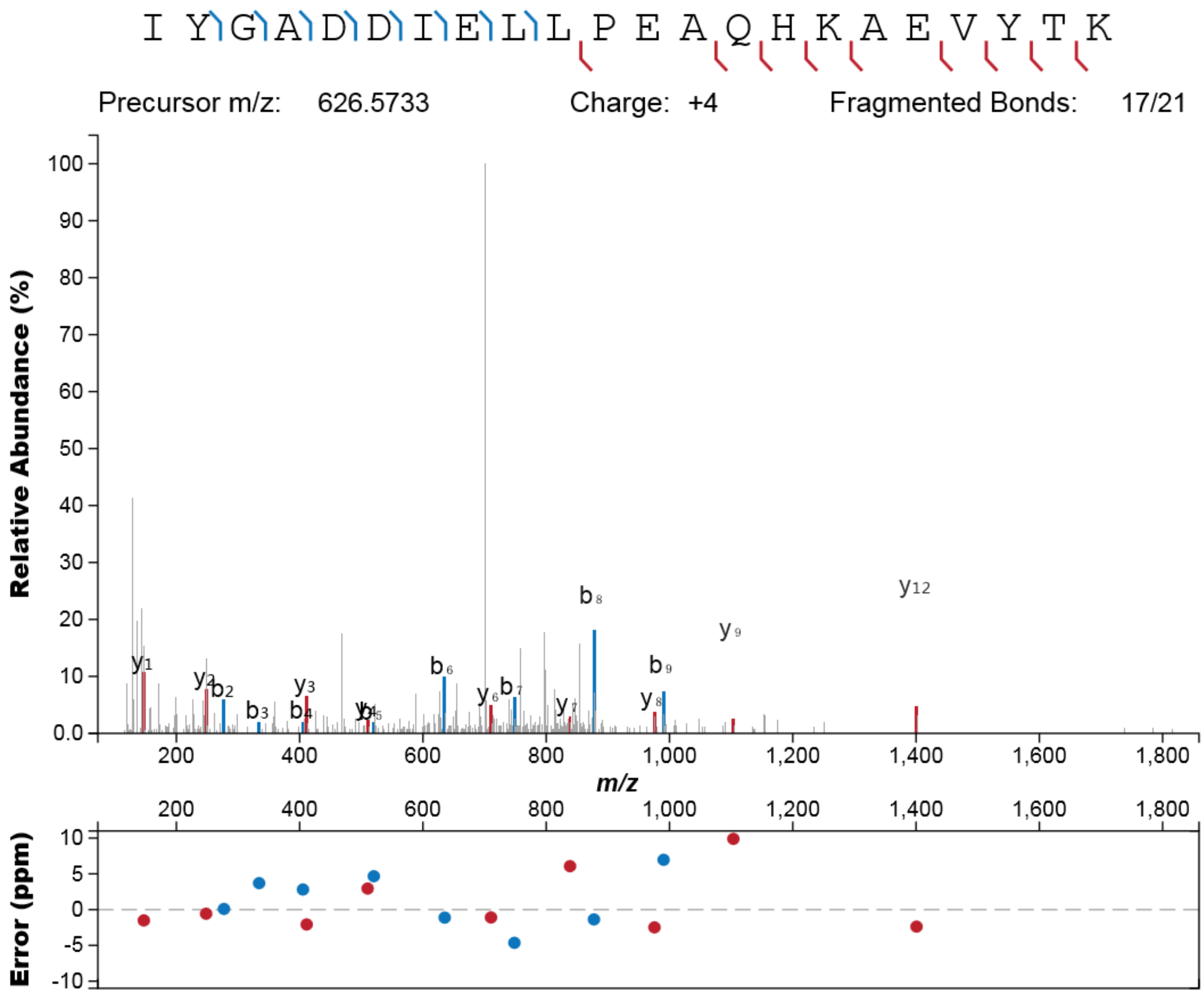
I Y G A D D I E L L P E A Q H K A E V Y T K
Precursor m/z: 626.5733
Charge: +4
Fragmented Bonds: 17/21
Relative Abundance (%)
m/z
Error (ppm)

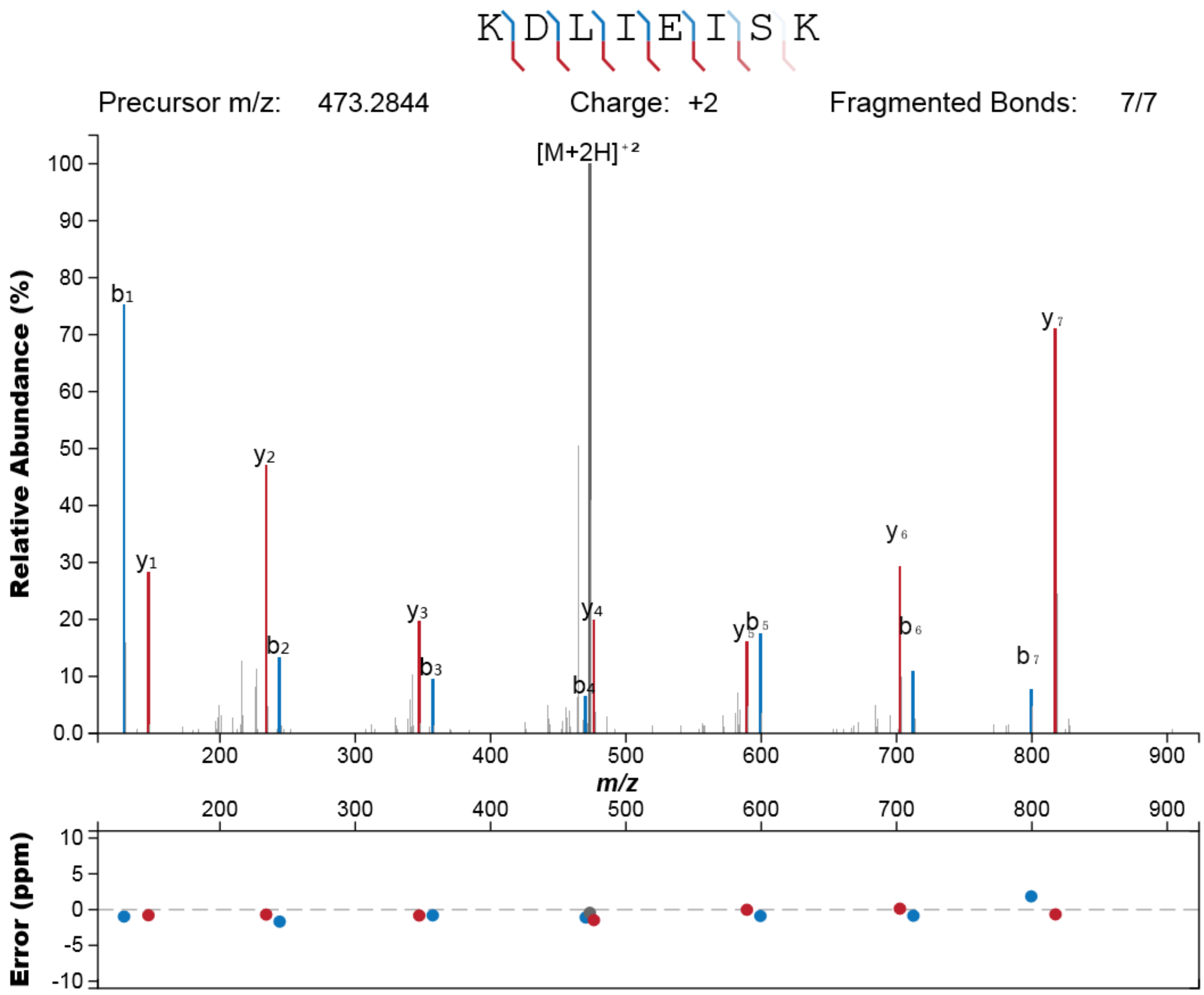

K D L I E I S K
Precursor m/z: 473.2844
Charge: +2
Fragmented Bonds: 7/7
[M+2H]+2
Relative Abundance (%)
m/z
Error (ppm)
b1 y1 y2 b2 y3 b3 b4 y4 y5 b5 y6 b6 b7 y7

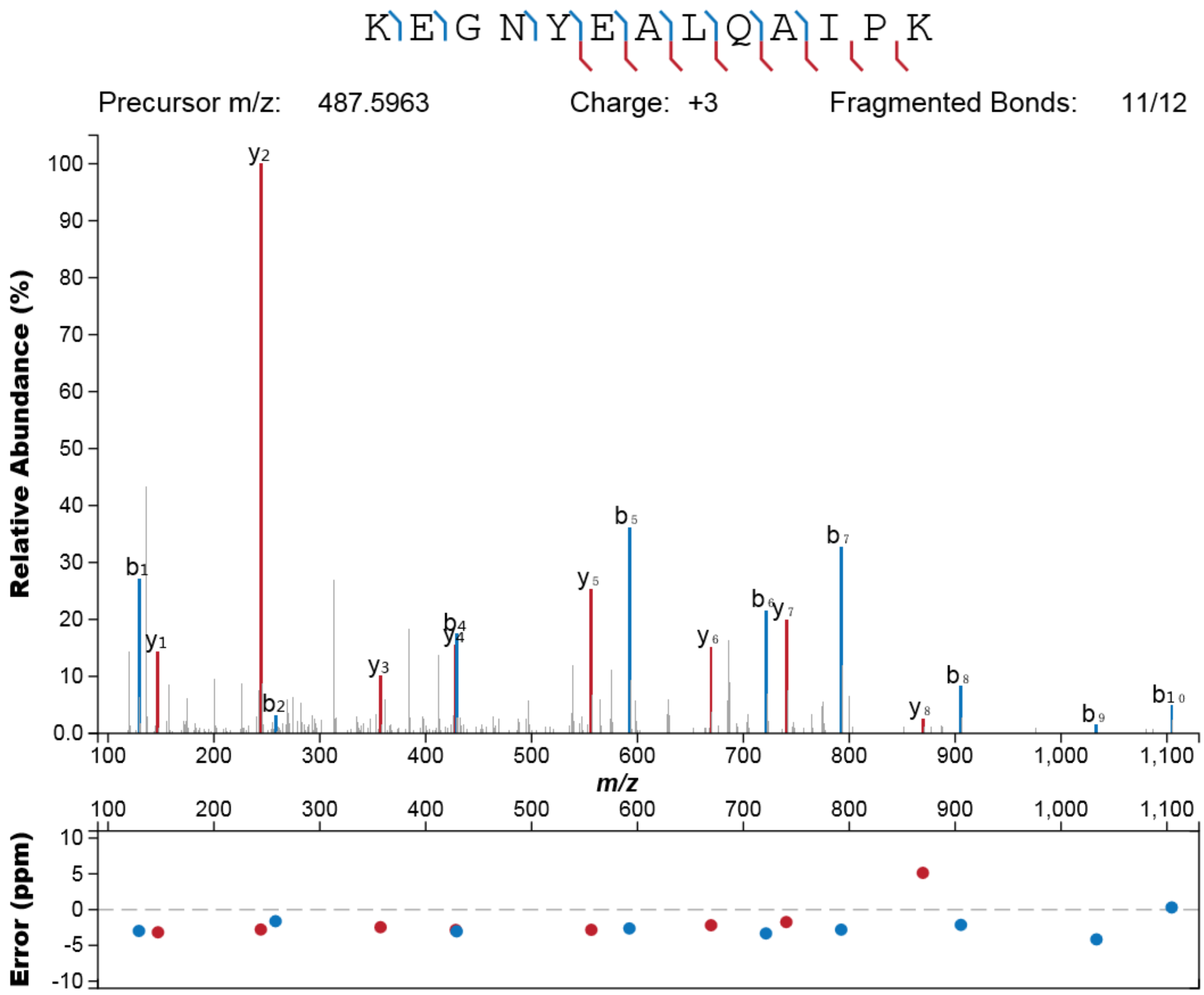

K E G N Y E A L Q A I P K
Precursor m/z: 487.5963
Charge: +3
Fragmented Bonds: 11/12
Relative Abundance (%)
m/z
Error (ppm)
y2
b1
y1
b2
y3
b4
y4
y5
b5
y6
b6
y7
b7
y8
b8
b9
b10

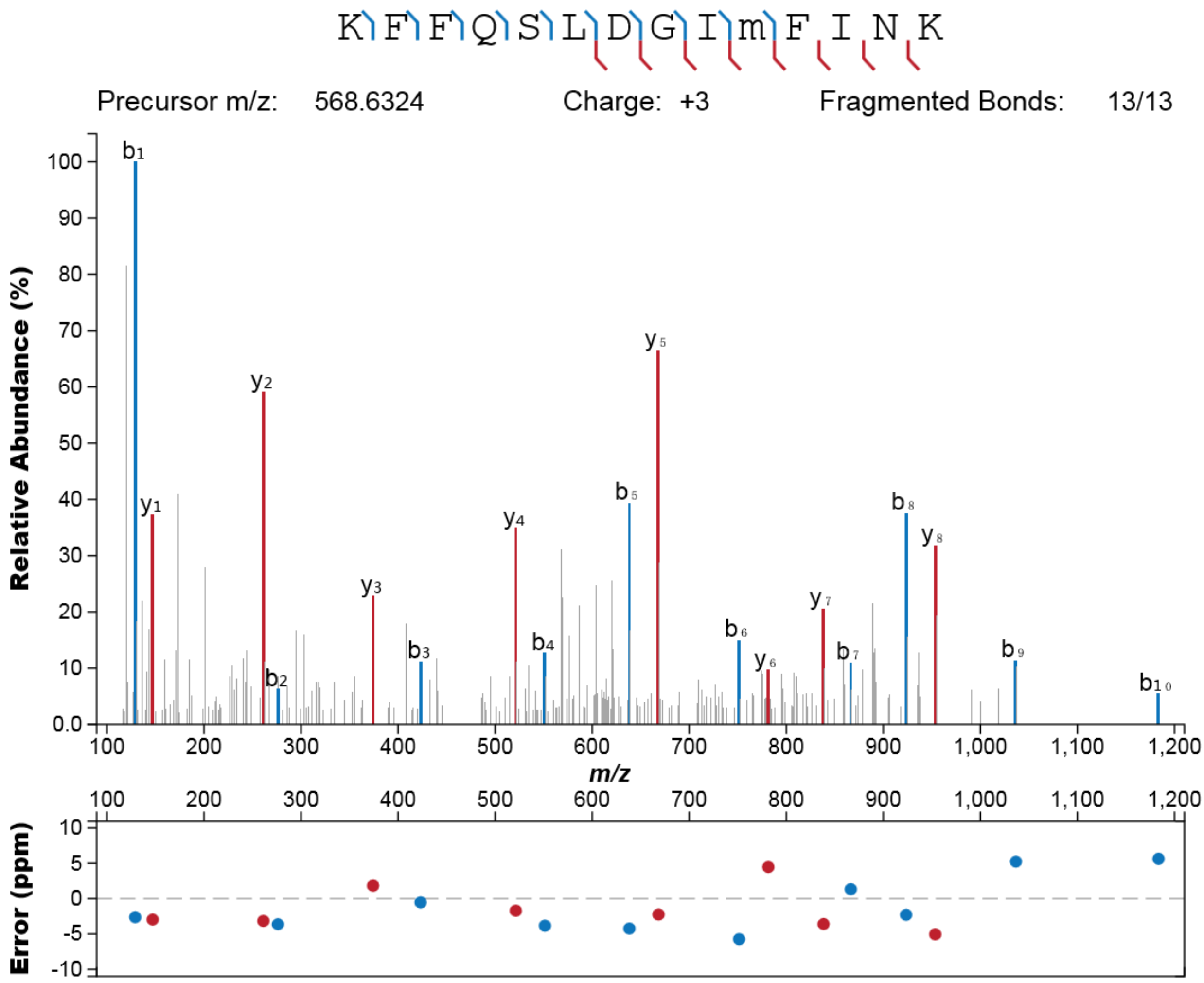
K F F Q S L D G I m F I N K
Precursor m/z: 568.6324
Charge: +3
Fragmented Bonds: 13/13
Relative Abundance (%)
m/z
Error (ppm)
b1 y1 y2 b2 y3 b3 y4 b4 b5 y5 b6 y6 y7 b7 b8 y8 b9 b10

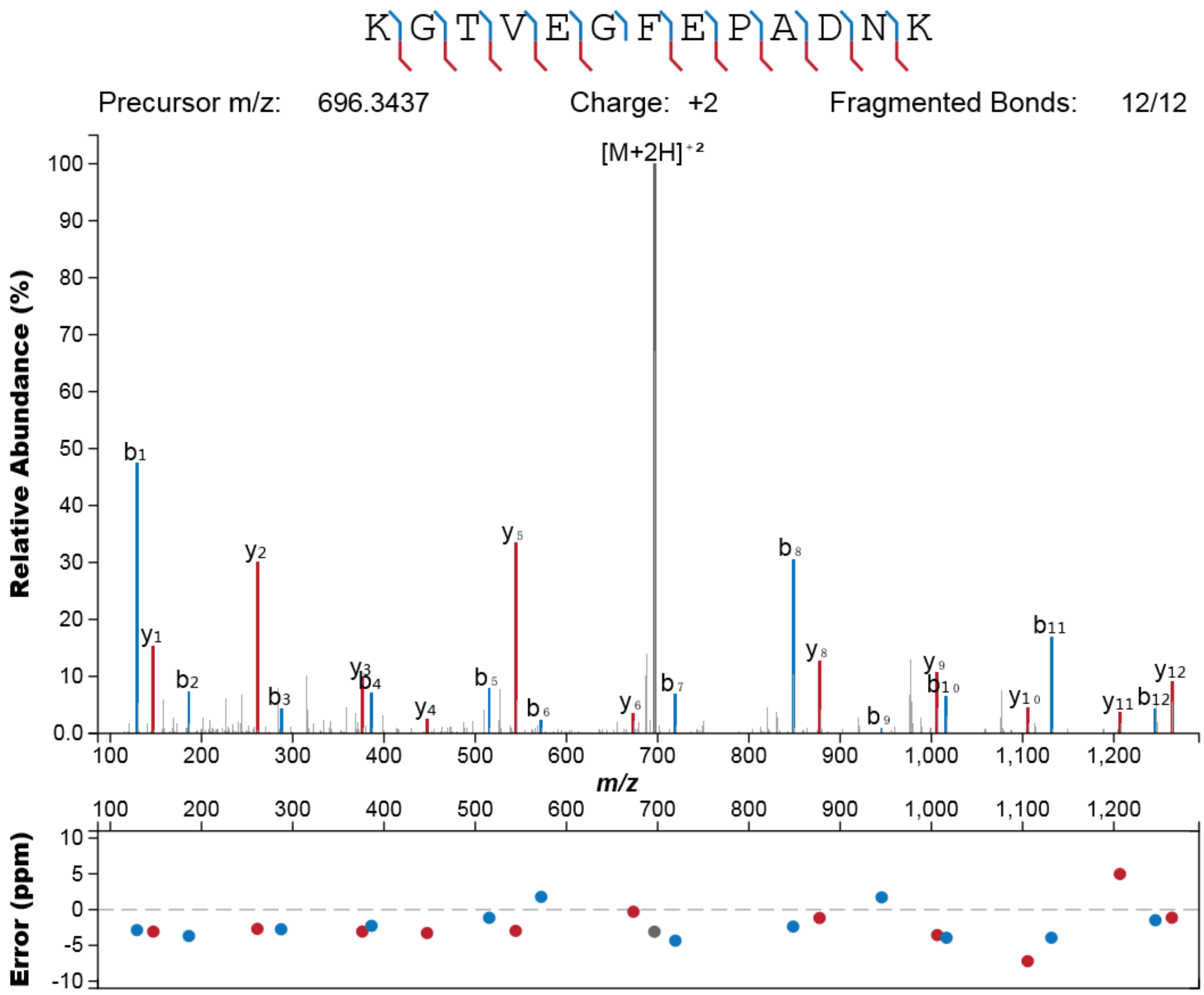

K G T V E G F E P A D N K
Precursor m/z: 696.3437
Charge: +2
Fragmented Bonds: 12/12
[M+2H]+2
Relative Abundance (%)
m/z
Error (ppm)
b1
y1
b2
y2
b3
y3
b4
y4
b5
y5
b6
y6
b7
b8
y8
b9
y9
b10
y10
b11
y11
b12
y12

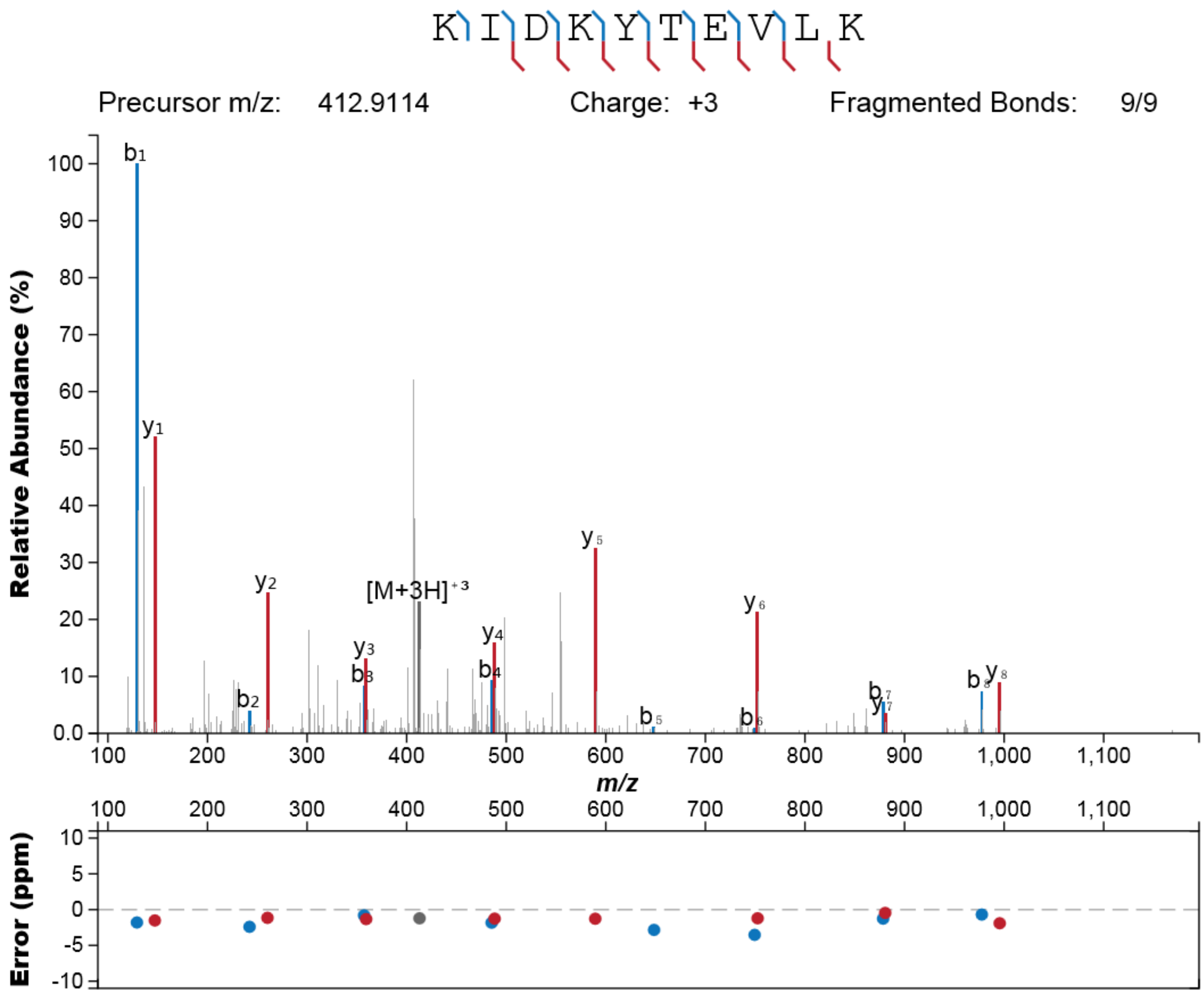

K I D K Y T E V L K
Precursor m/z: 412.9114
Charge: +3
Fragmented Bonds: 9/9
Relative Abundance (%)
m/z
Error (ppm)
[M+3H]+3

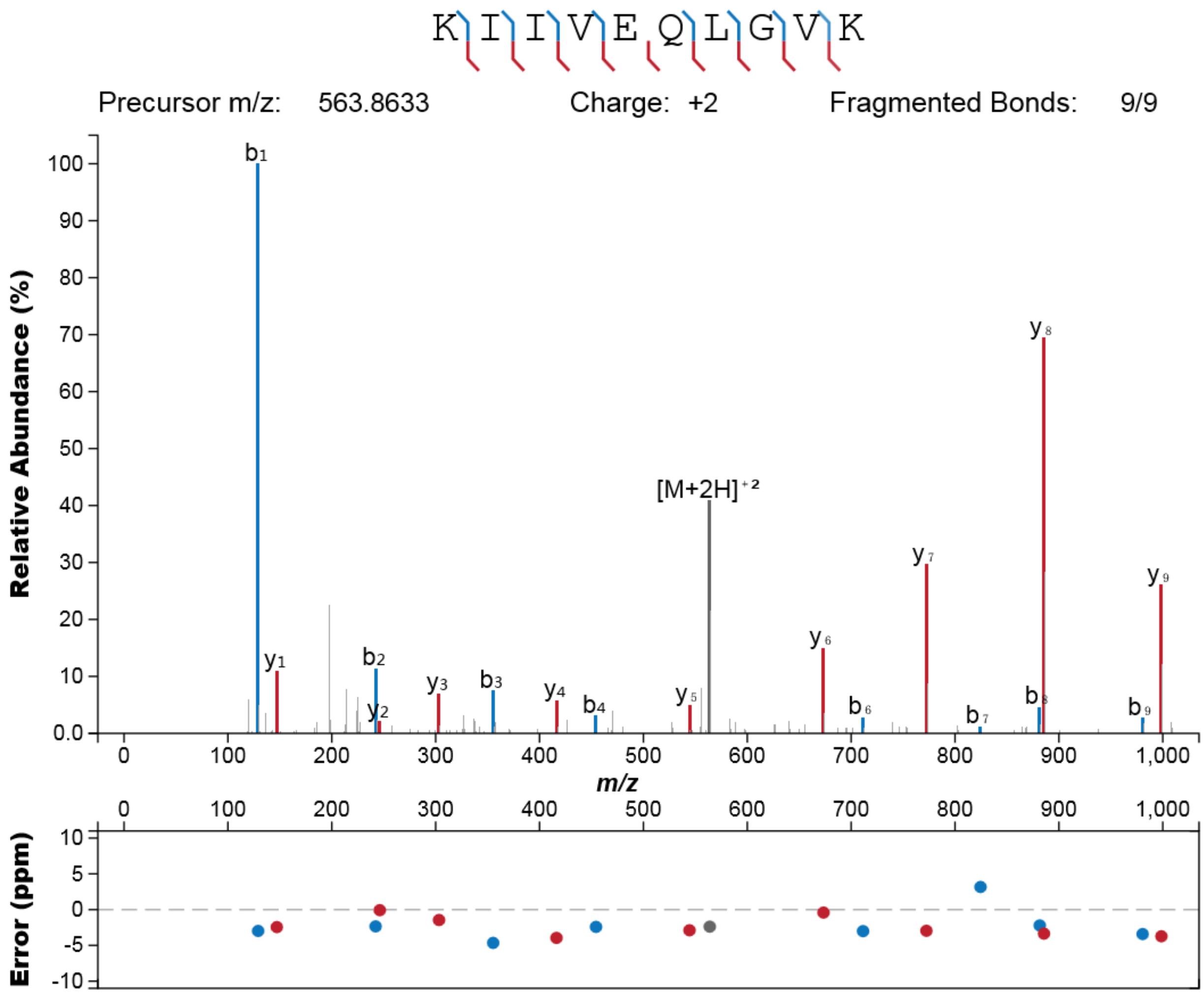

K I I V E Q L G V K
Precursor m/z: 563.8633
Charge: +2
Fragmented Bonds: 9/9
Relative Abundance (%)
m/z
Error (ppm)
[M+2H]+2
b1
y1
b2
y2
y3
b3
y4
b4
y5
y6
b6
y7
b7
y8
b8
y9
b9

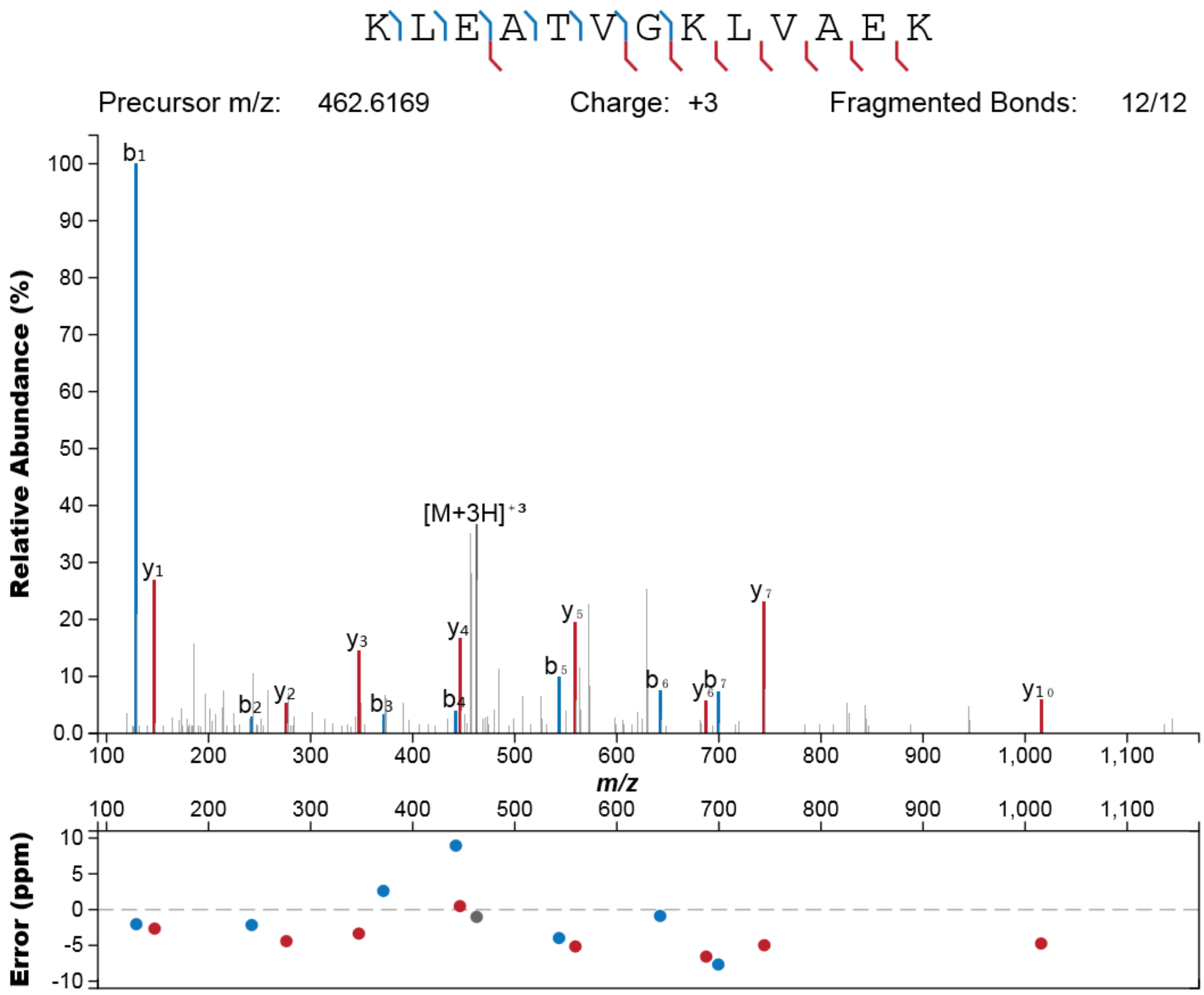
K L E A T V G K L V A E K
Precursor m/z: 462.6169
Charge: +3
Fragmented Bonds: 12/12
Relative Abundance (%)
m/z
Error (ppm)
[M+3H]+3
b1
y1
b2
y2
y3
b3
b4
y4
b5
y5
b6
y6
b7
y7
y10

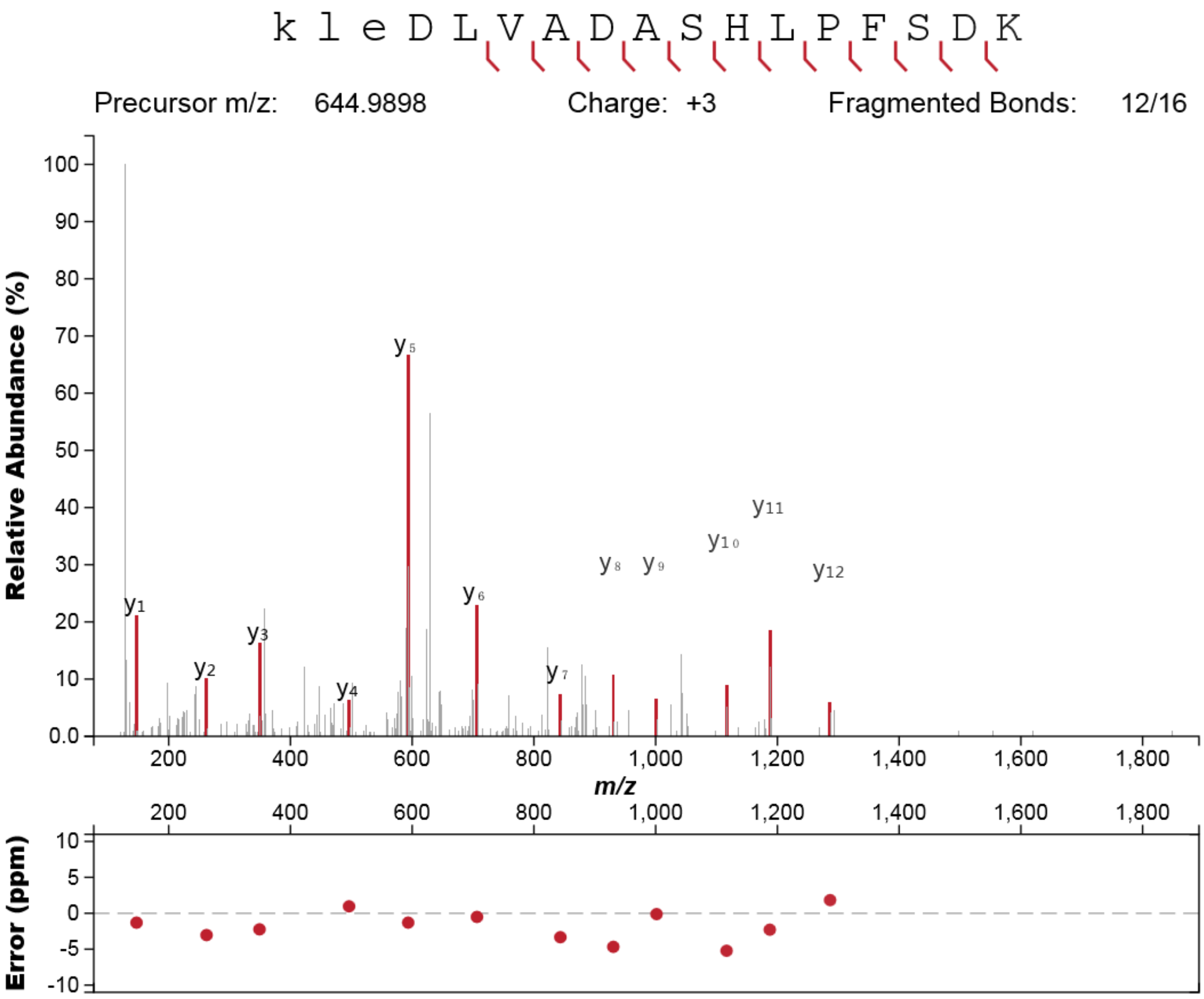
k l e D L V A D A S H L P F S D K
Precursor m/z: 644.9898
Charge: +3
Fragmented Bonds: 12/16
Relative Abundance (%)
y1
y2
y3
y4
y5
y6
y7
y8
y9
y10
y11
y12
m/z
Error (ppm)

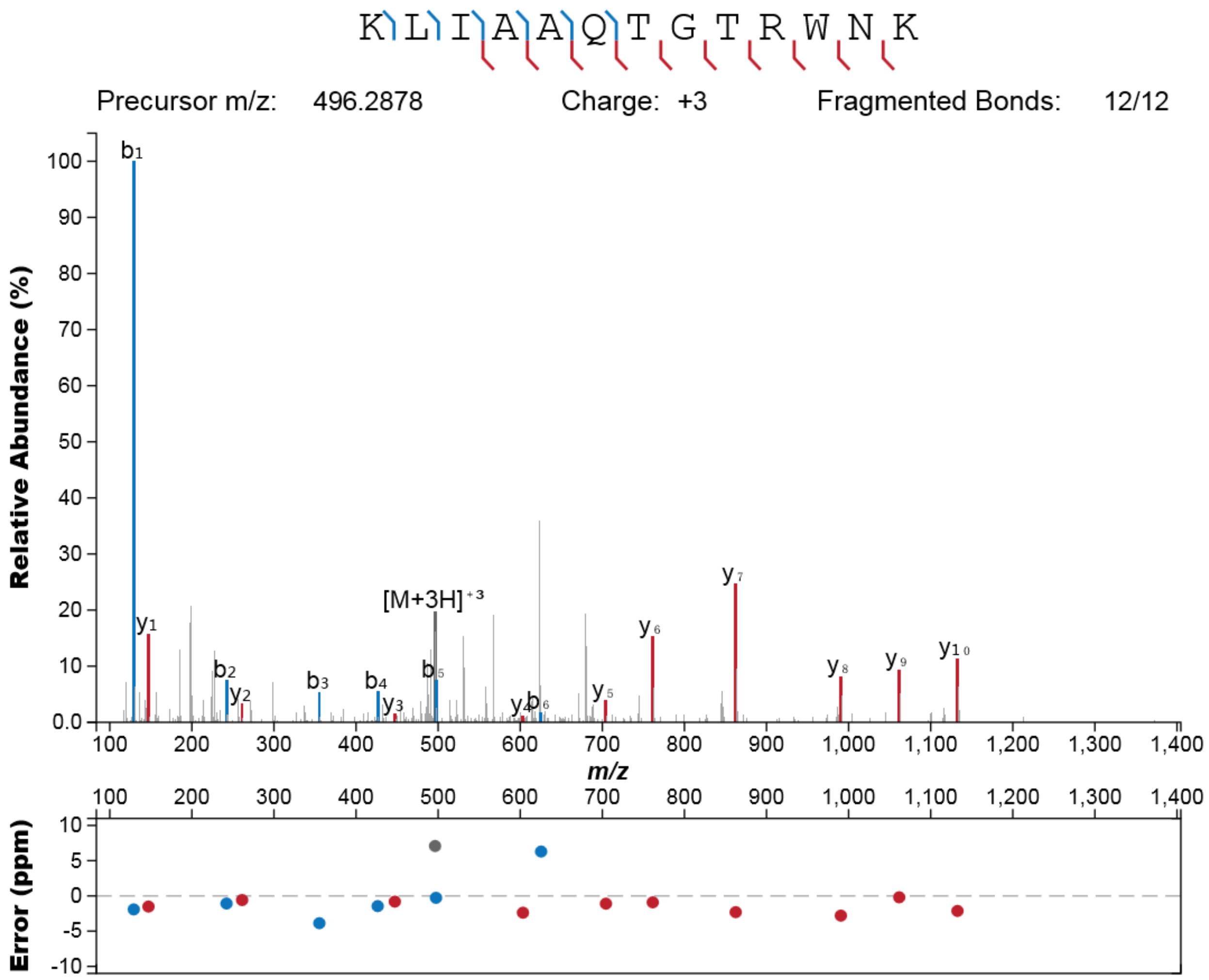

K L I A A Q T G T R W N K
Precursor m/z: 496.2878
Charge: +3
Fragmented Bonds: 12/12
Relative Abundance (%)
m/z
Error (ppm)
[M+3H]+3

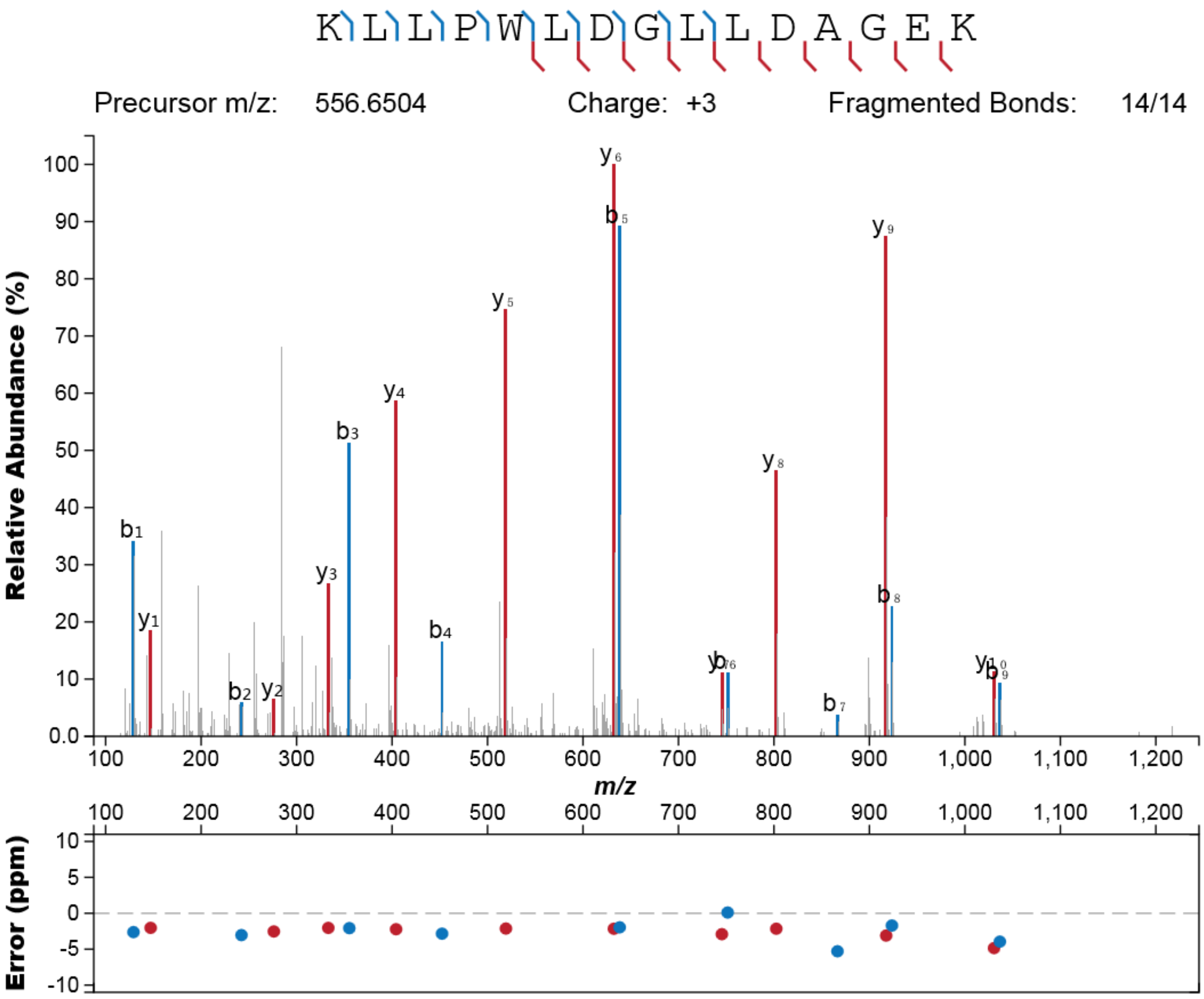

K L L P W L D G L L D A G E K
Precursor m/z: 556.6504
Charge: +3
Fragmented Bonds: 14/14
Relative Abundance (%)
m/z
Error (ppm)

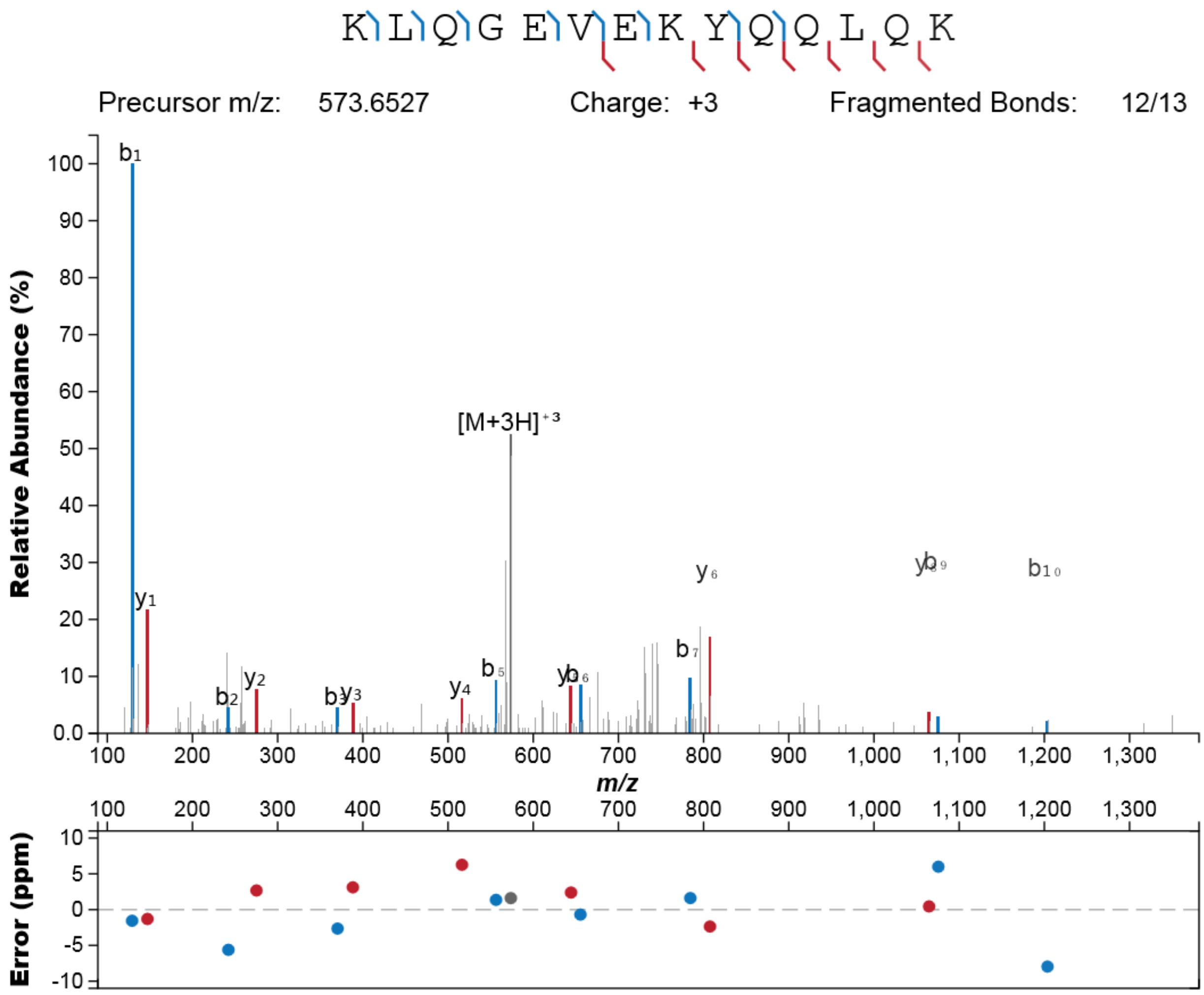

K L Q G E V E K Y Q Q L Q K
Precursor m/z: 573.6527
Charge: +3
Fragmented Bonds: 12/13
Relative Abundance (%)
m/z
Error (ppm)
[M+3H]+3

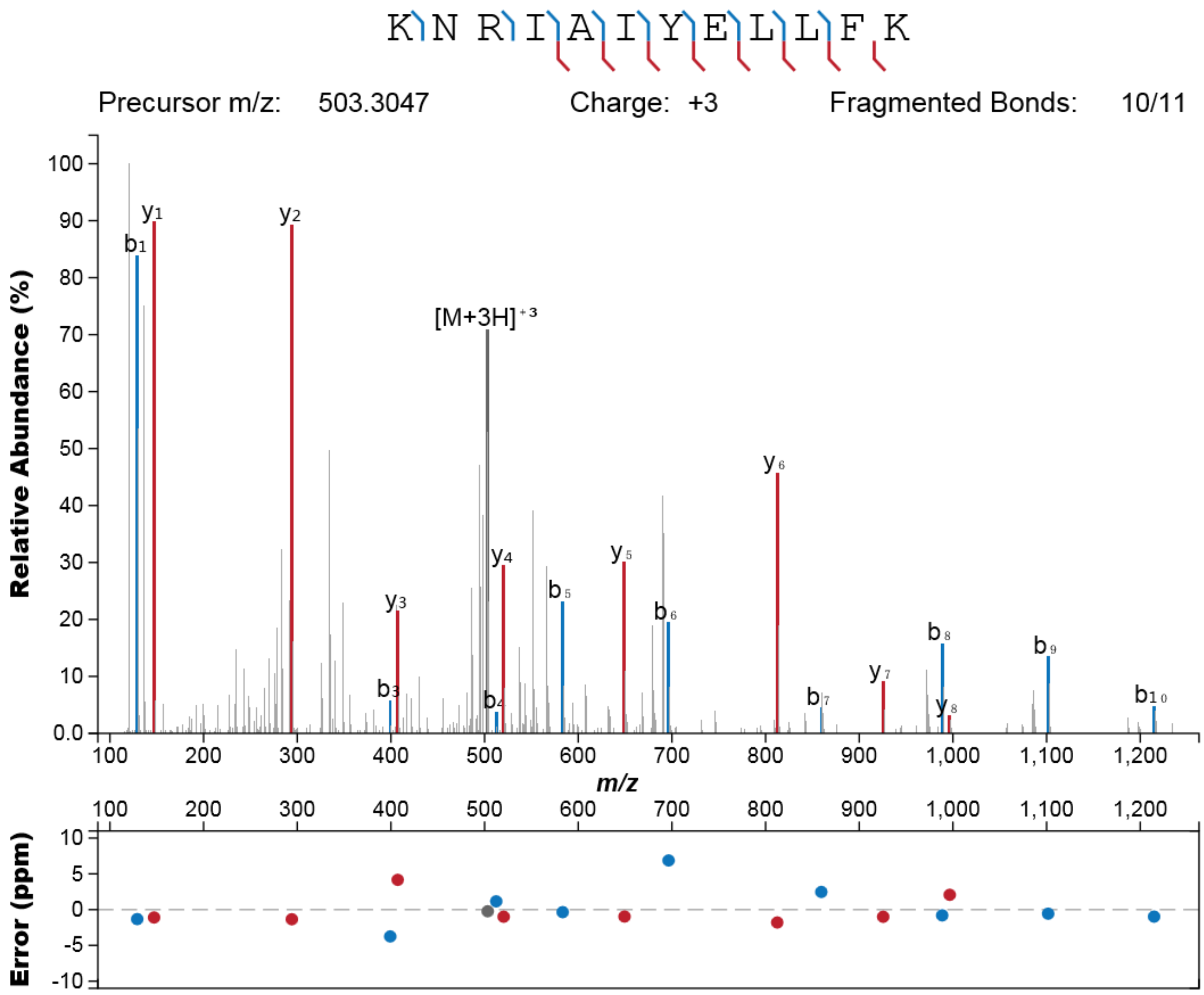

K N R I A I Y E L L F K
Precursor m/z: 503.3047
Charge: +3
Fragmented Bonds: 10/11
Relative Abundance (%)
[M+3H]+3
m/z
Error (ppm)

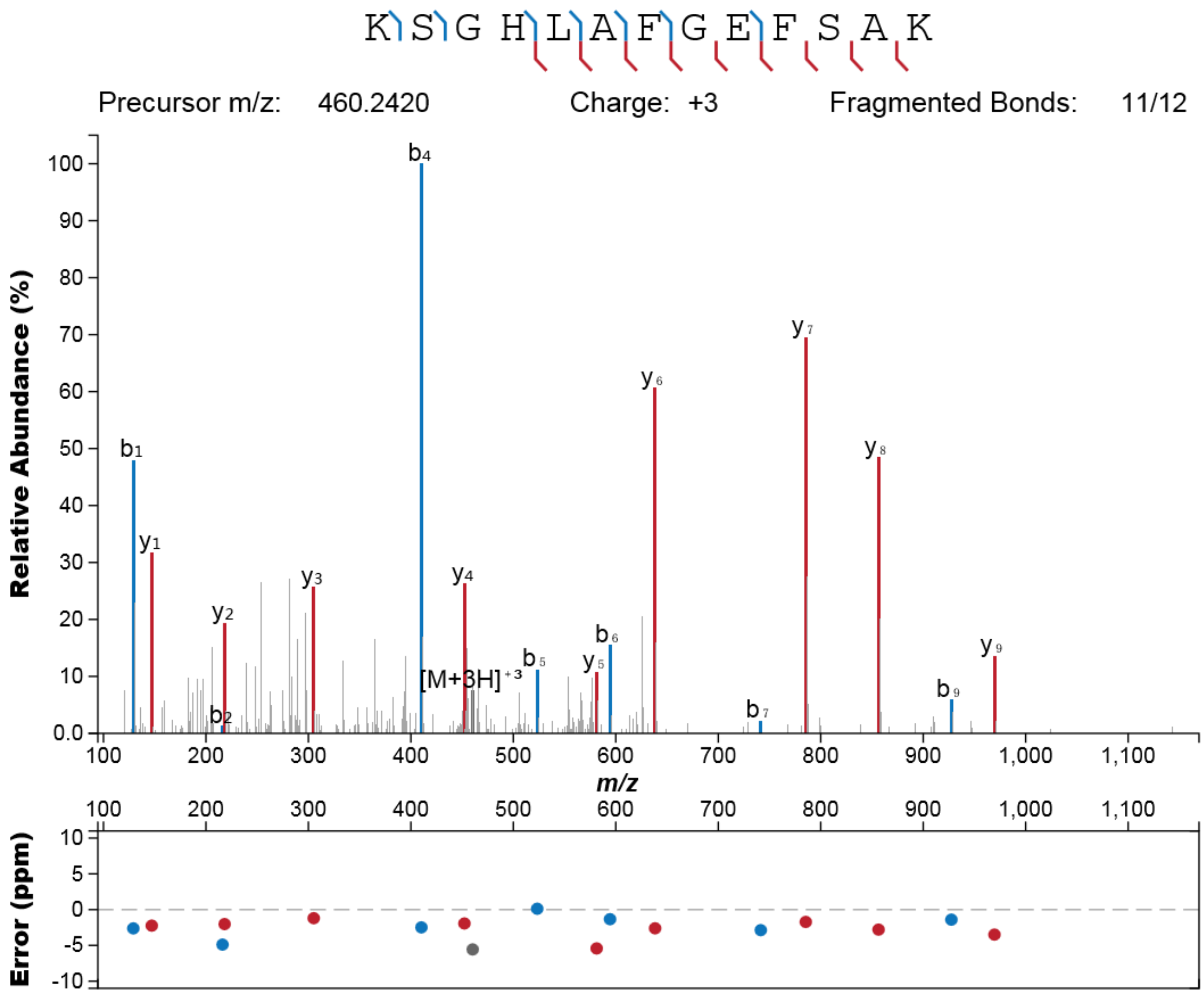

K S G H L A F G E F S A K
Precursor m/z: 460.2420
Charge: +3
Fragmented Bonds: 11/12
Relative Abundance (%)
m/z
Error (ppm)
b1
y1
y2
b2
y3
b4
y4
[M+3H]+3
b5
y5
b6
y6
b7
y7
y8
b9
y9

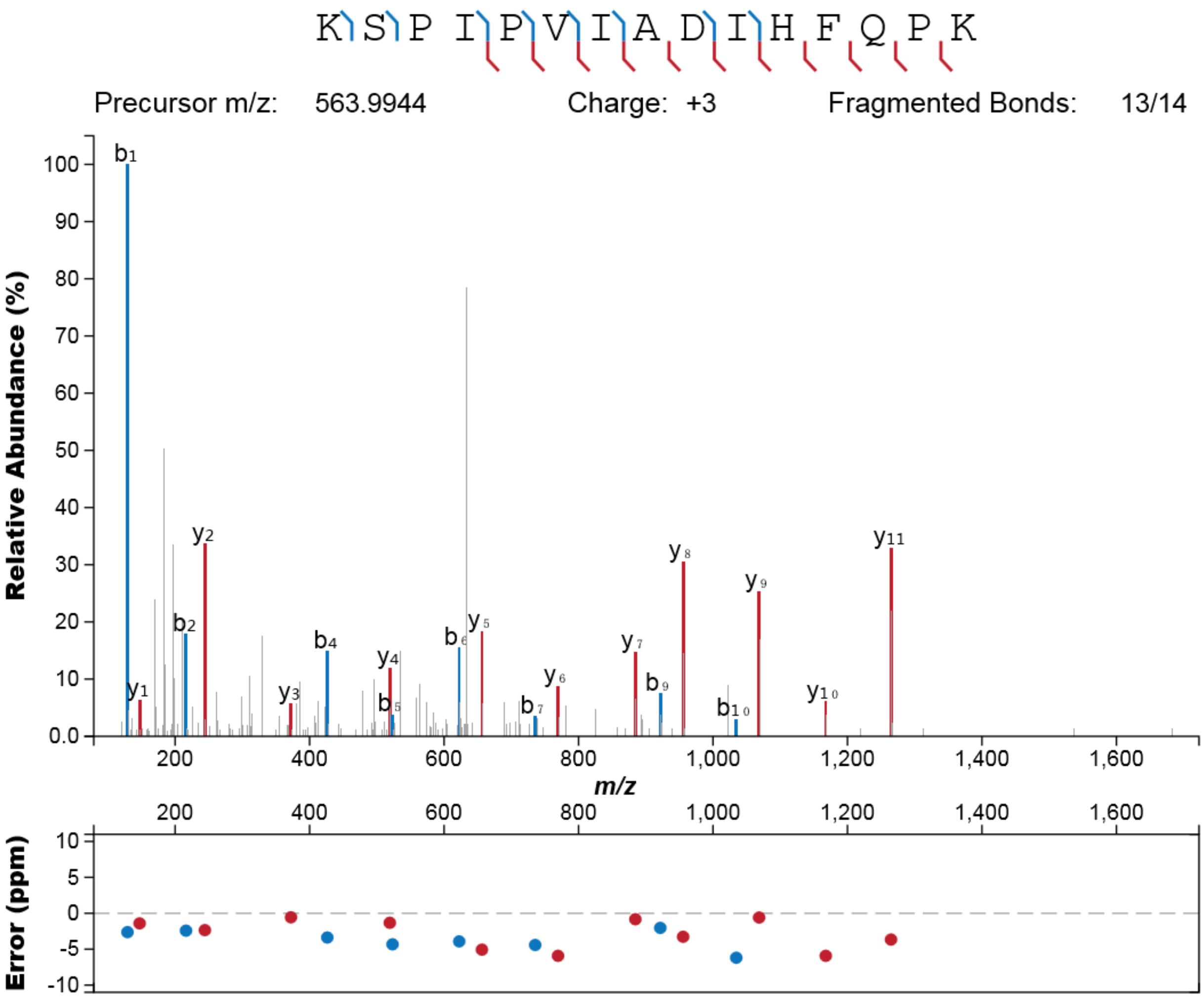
K S P I P V I A D I H F Q P K
Precursor m/z: 563.9944
Charge: +3
Fragmented Bonds: 13/14
Relative Abundance (%)
m/z
Error (ppm)
b1
b2
b4
b5
b6
b7
b9
b10
y1
y2
y3
y4
y5
y6
y7
y8
y9
y10
y11

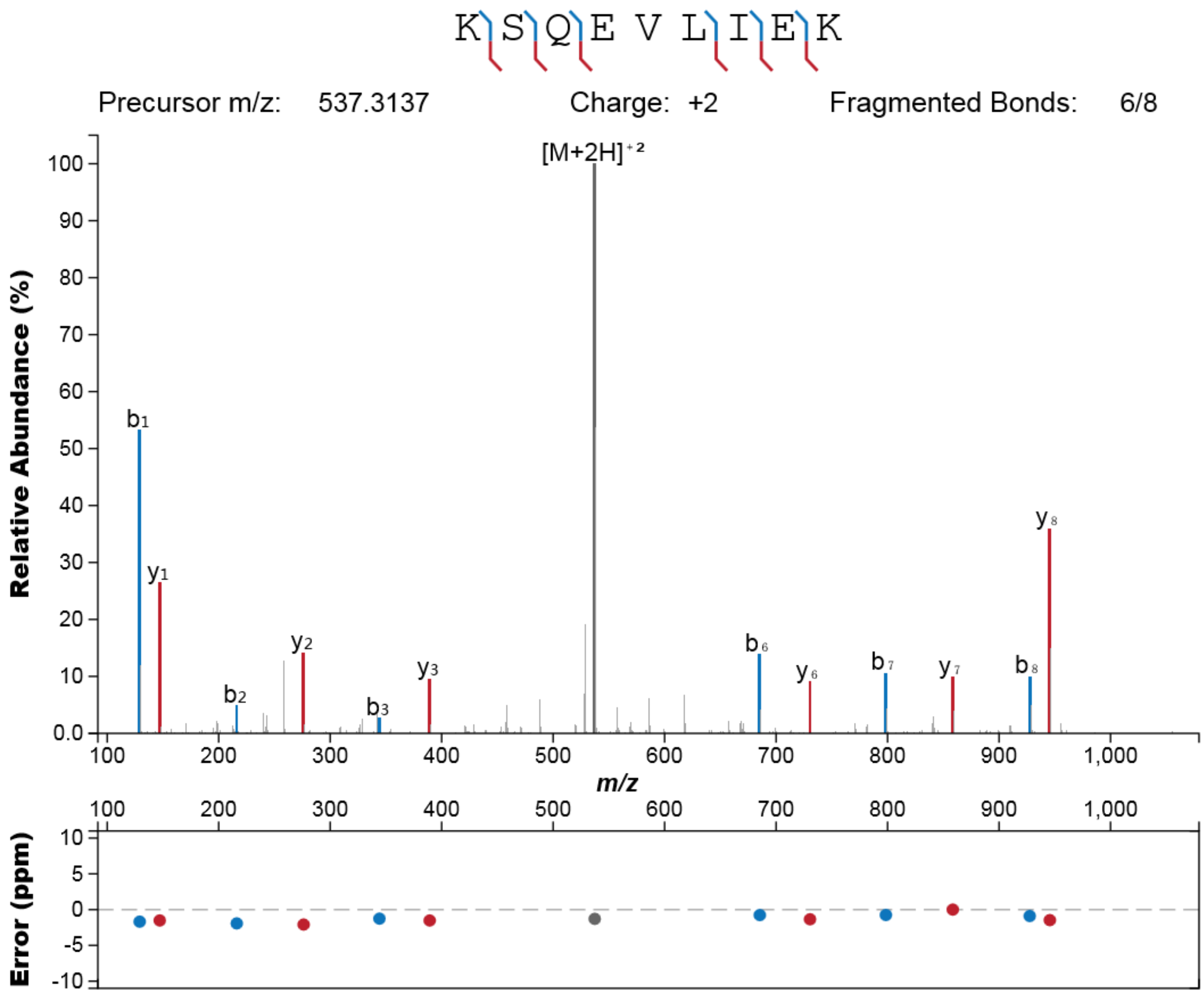

K S Q E V L I E K
Precursor m/z: 537.3137
Charge: +2
Fragmented Bonds: 6/8
[M+2H]+2
Relative Abundance (%)
m/z
Error (ppm)
b1
y1
b2
y2
b3
y3
b6
y6
b7
y7
b8
y8

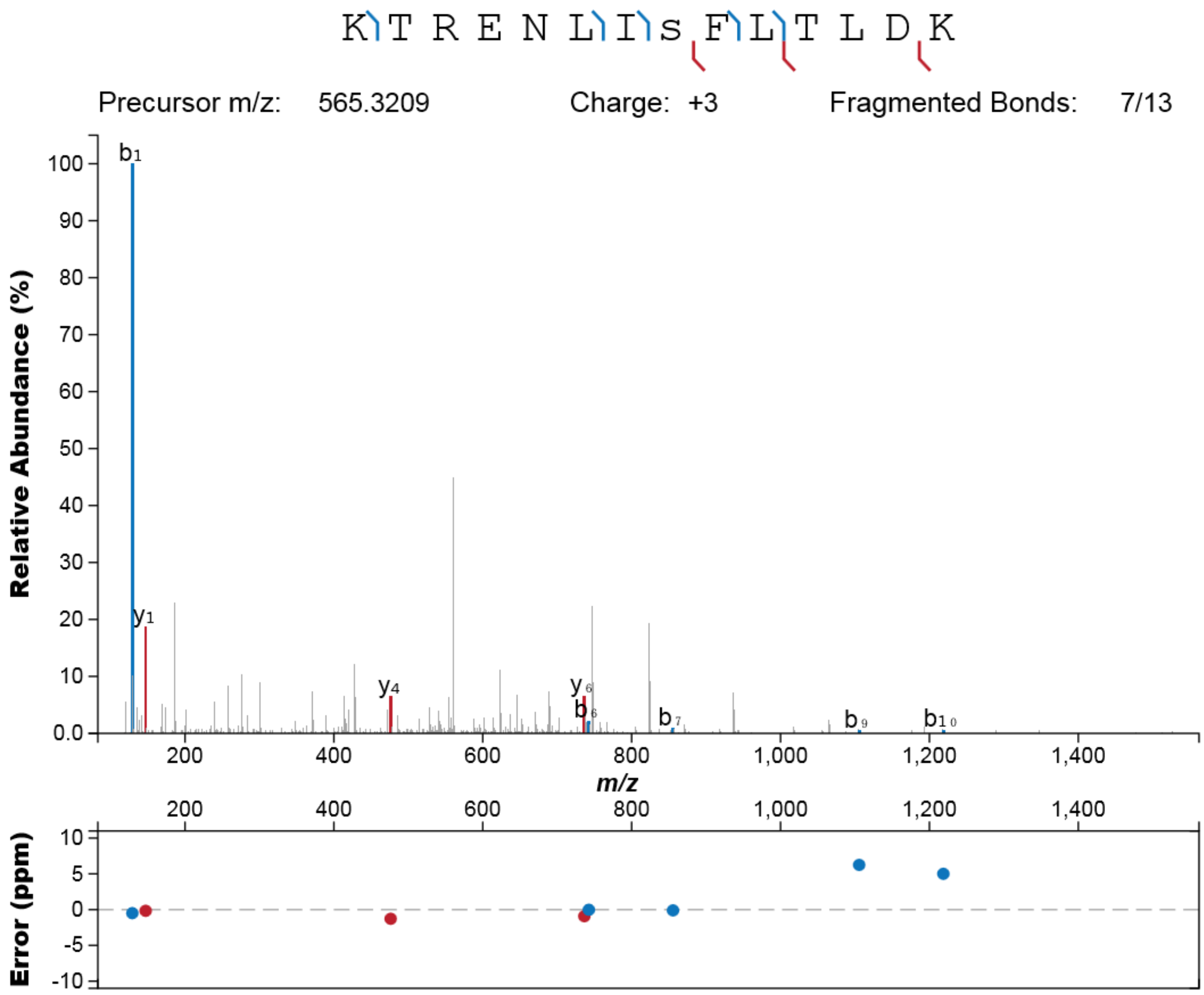

K T R E N L I s F L T L D K
Precursor m/z: 565.3209
Charge: +3
Fragmented Bonds: 7/13
Relative Abundance (%)
m/z
Error (ppm)
b1
y1
y4
y6
b6
b7
b9
b10

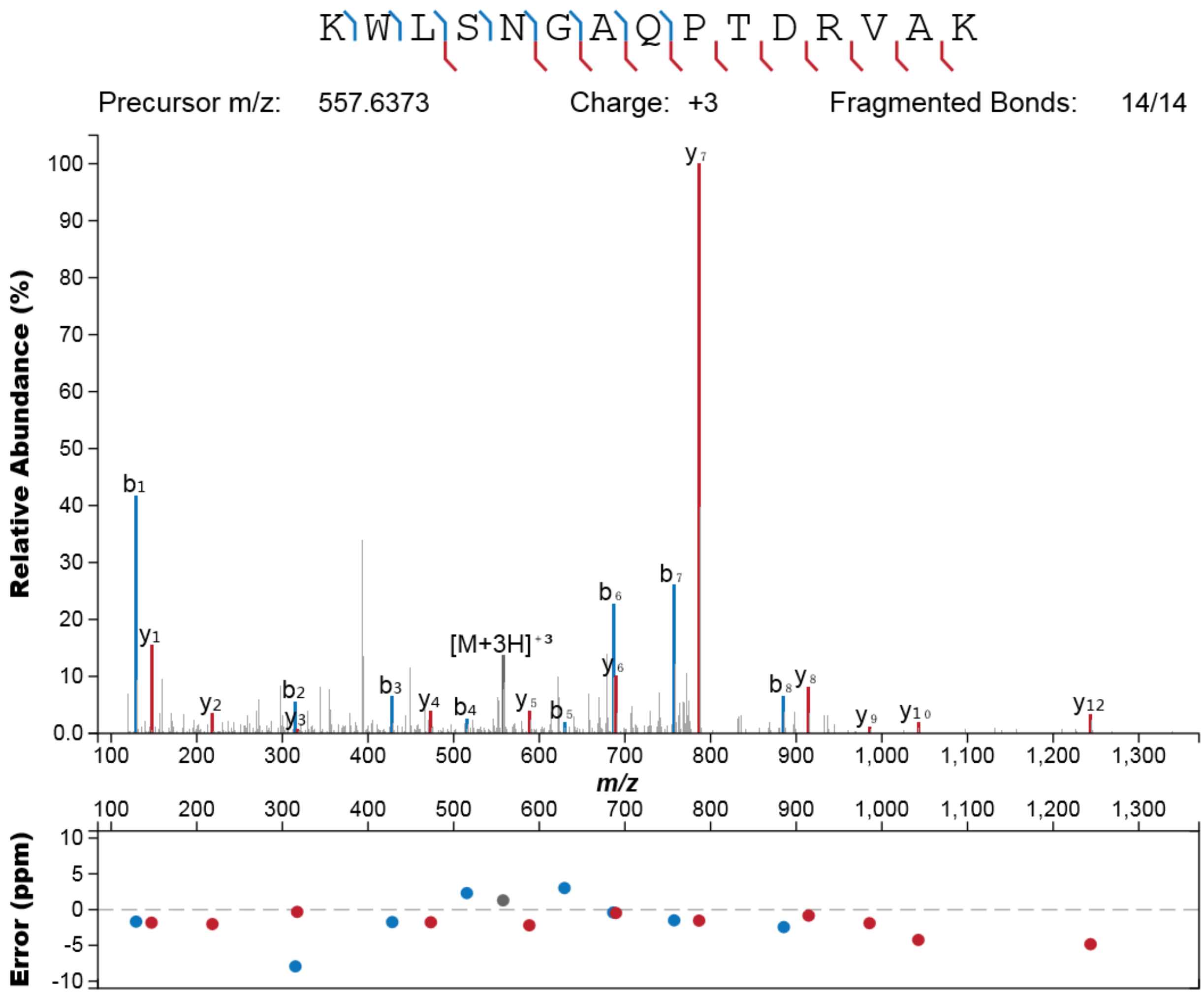

K W L S N G A Q P T D R V A K
Precursor m/z: 557.6373
Charge: +3
Fragmented Bonds: 14/14
Relative Abundance (%)
m/z
[M+3H]+3
Error (ppm)

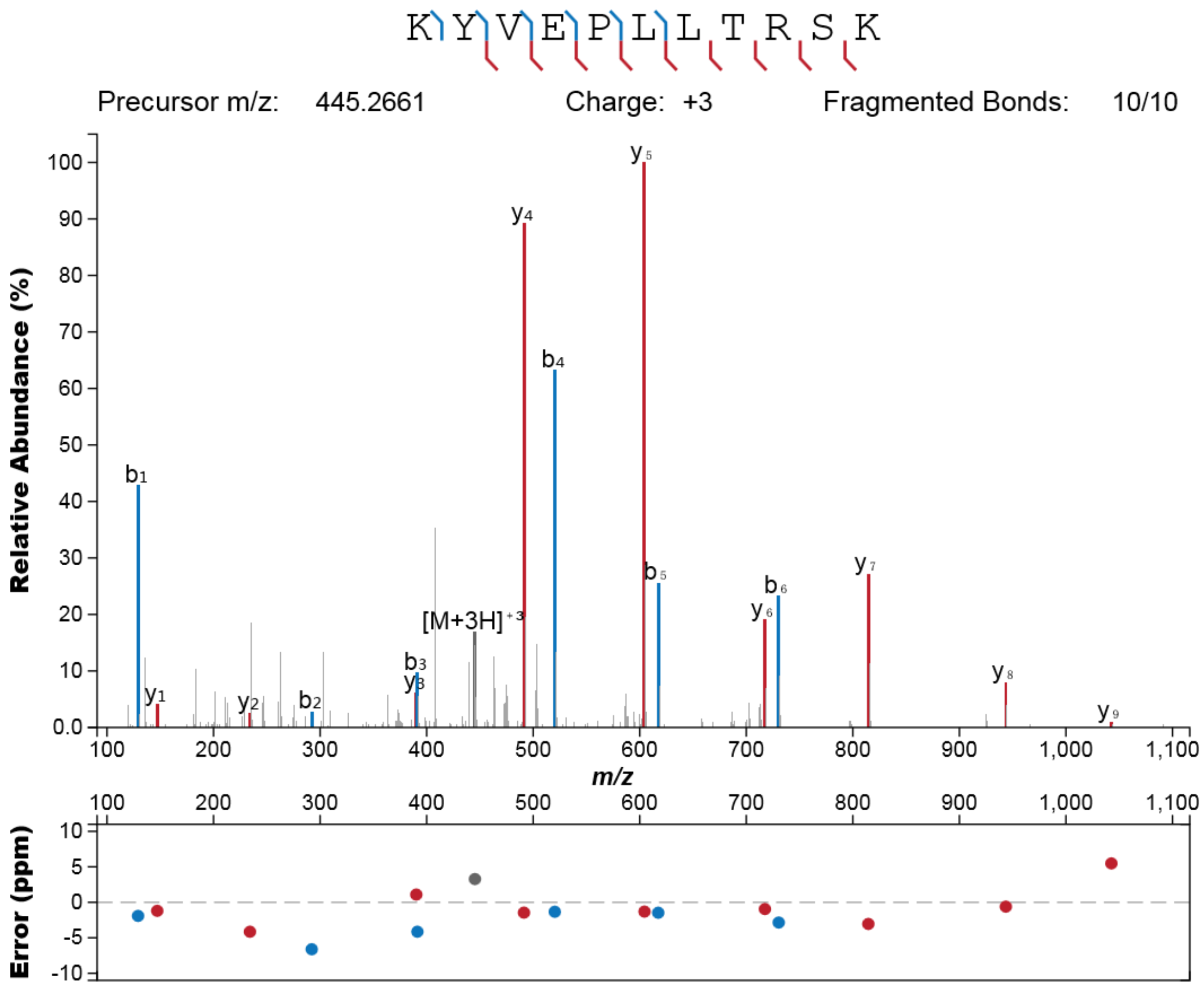

K Y V E P L L T R S K
Precursor m/z: 445.2661
Charge: +3
Fragmented Bonds: 10/10
Relative Abundance (%)
m/z
Error (ppm)
[M+3H]+3

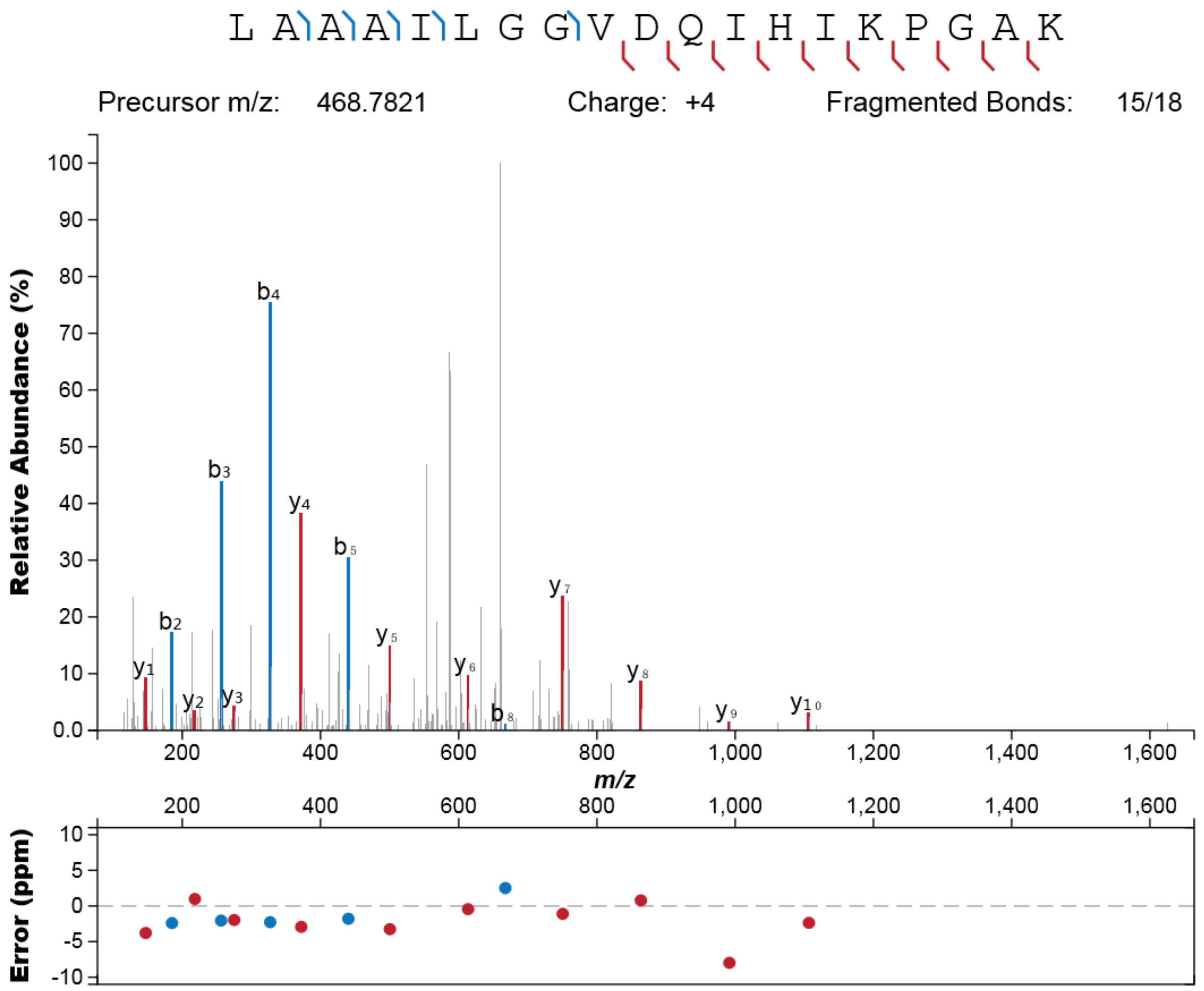

L A A A I L G G V D Q I H I K P G A K
Precursor m/z: 468.7821
Charge: +4
Fragmented Bonds: 15/18
Relative Abundance (%)
m/z
Error (ppm)
b2
b3
b4
b5
b8
y1
y2
y3
y4
y5
y6
y7
y8
y9
y10

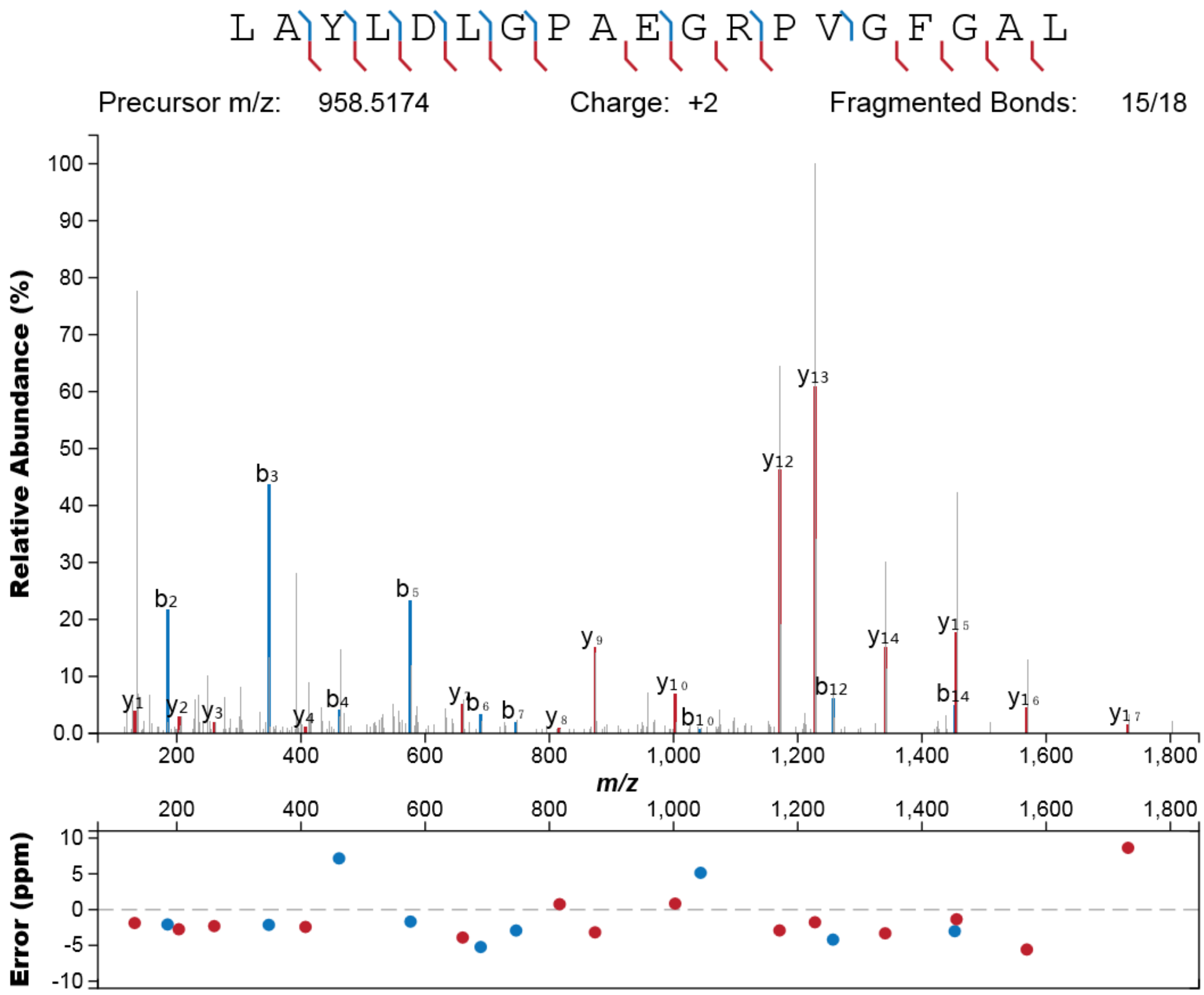
L A Y L D L G P A E G R P V G F G A L
Precursor m/z: 958.5174
Charge: +2
Fragmented Bonds: 15/18
Relative Abundance (%)
m/z
Error (ppm)
b2 b3 b4 b5 b6 b7 b10 b12 b14
y1 y2 y3 y4 y7 y8 y9 y10 y12 y13 y14 y15 y16 y17

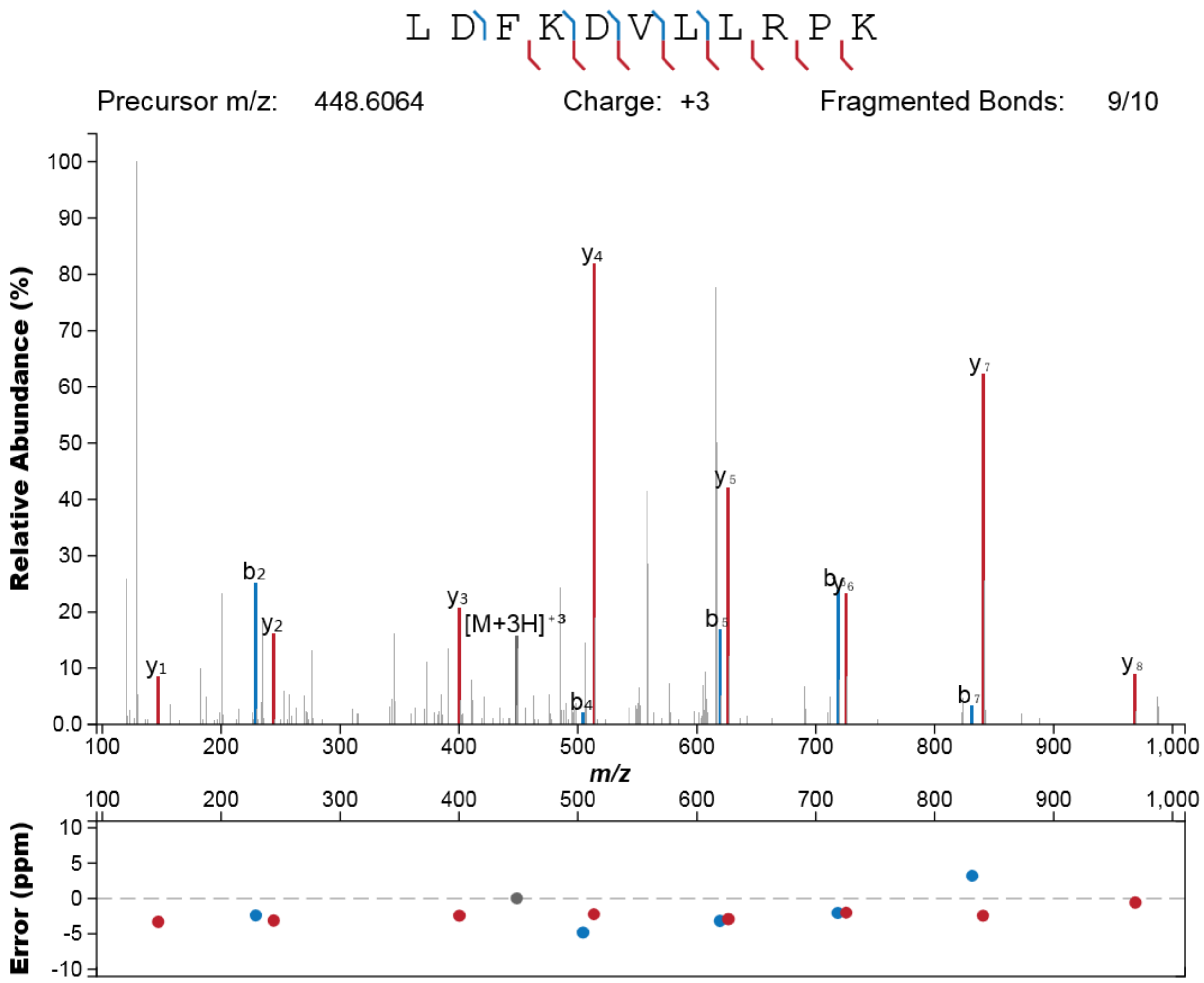

L D F K D V L L R P K
Precursor m/z: 448.6064
Charge: +3
Fragmented Bonds: 9/10
Relative Abundance (%)
m/z
Error (ppm)
y1
b2
y2
y3
[M+3H]+3
y4
b4
b5
y5
b6
y6
b7
y7
y8

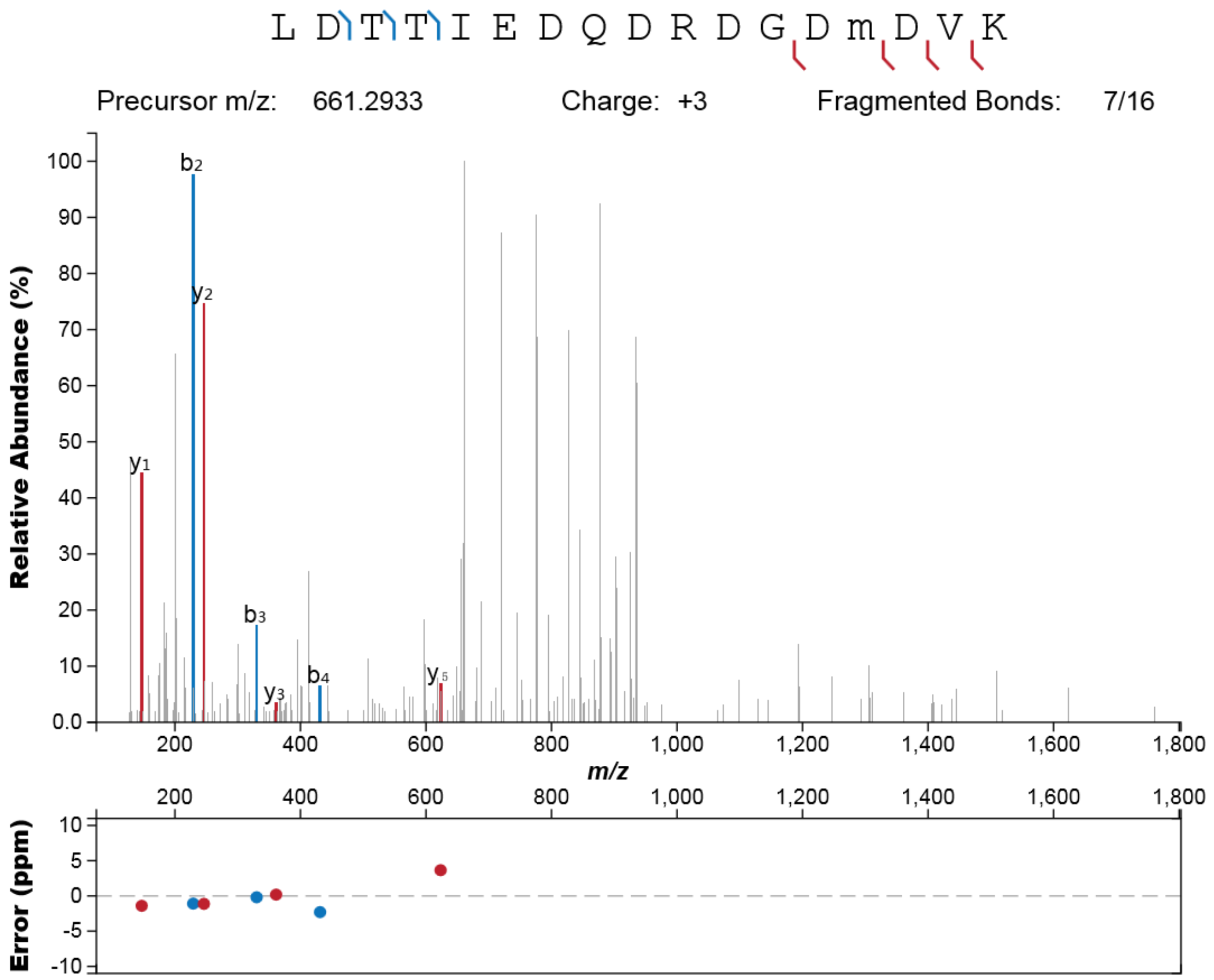
L D T T I E D Q D R D G D m D V K
Precursor m/z: 661.2933
Charge: +3
Fragmented Bonds: 7/16
Relative Abundance (%)
m/z
Error (ppm)
b2
y2
y1
b3
y3
b4
y5

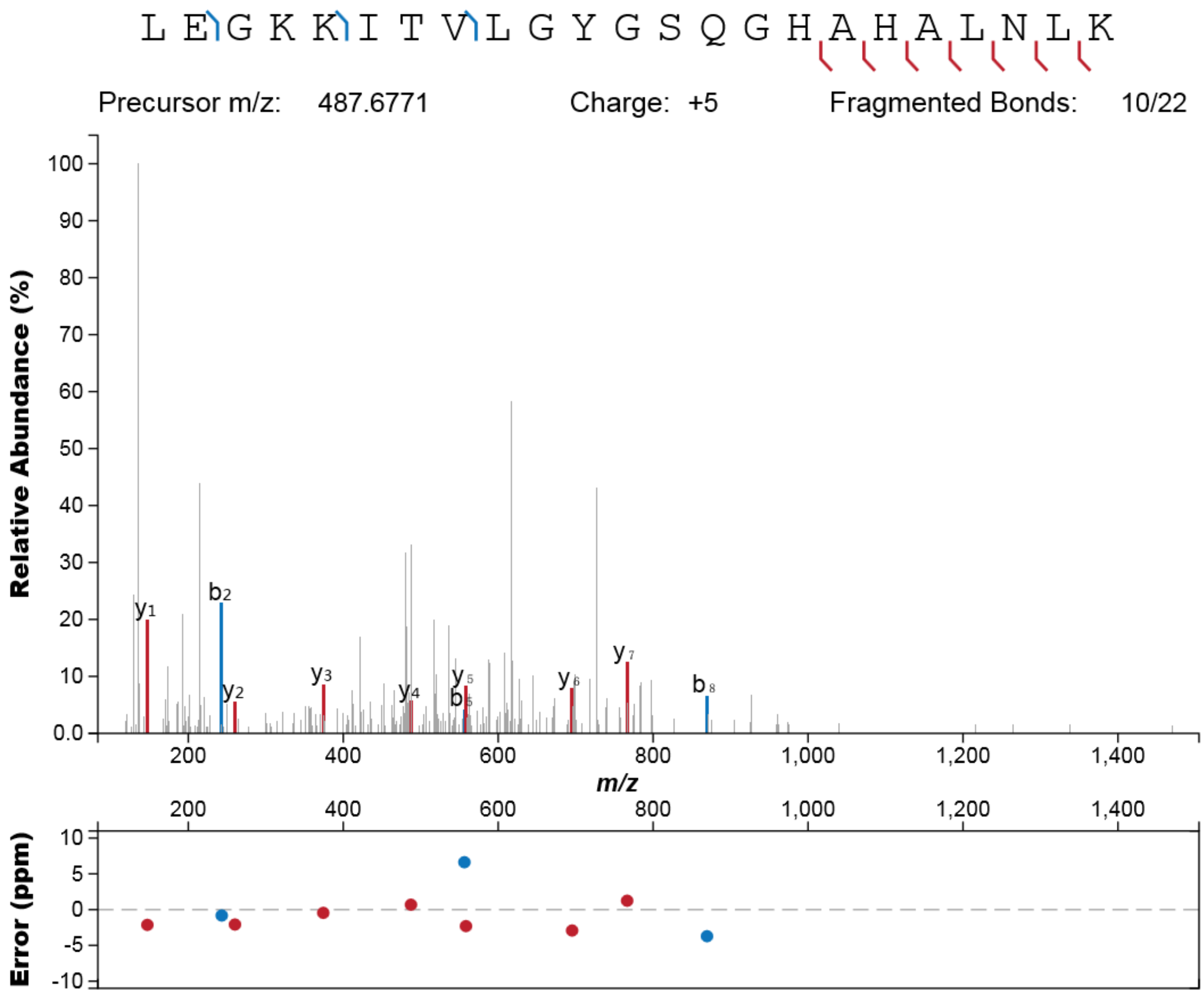

L E G K K I T V L G Y G S Q G H A H A L N L K
Precursor m/z: 487.6771
Charge: +5
Fragmented Bonds: 10/22
Relative Abundance (%)
m/z
Error (ppm)
y1
b2
y2
y3
y4
y5
b5
y6
y7
b8

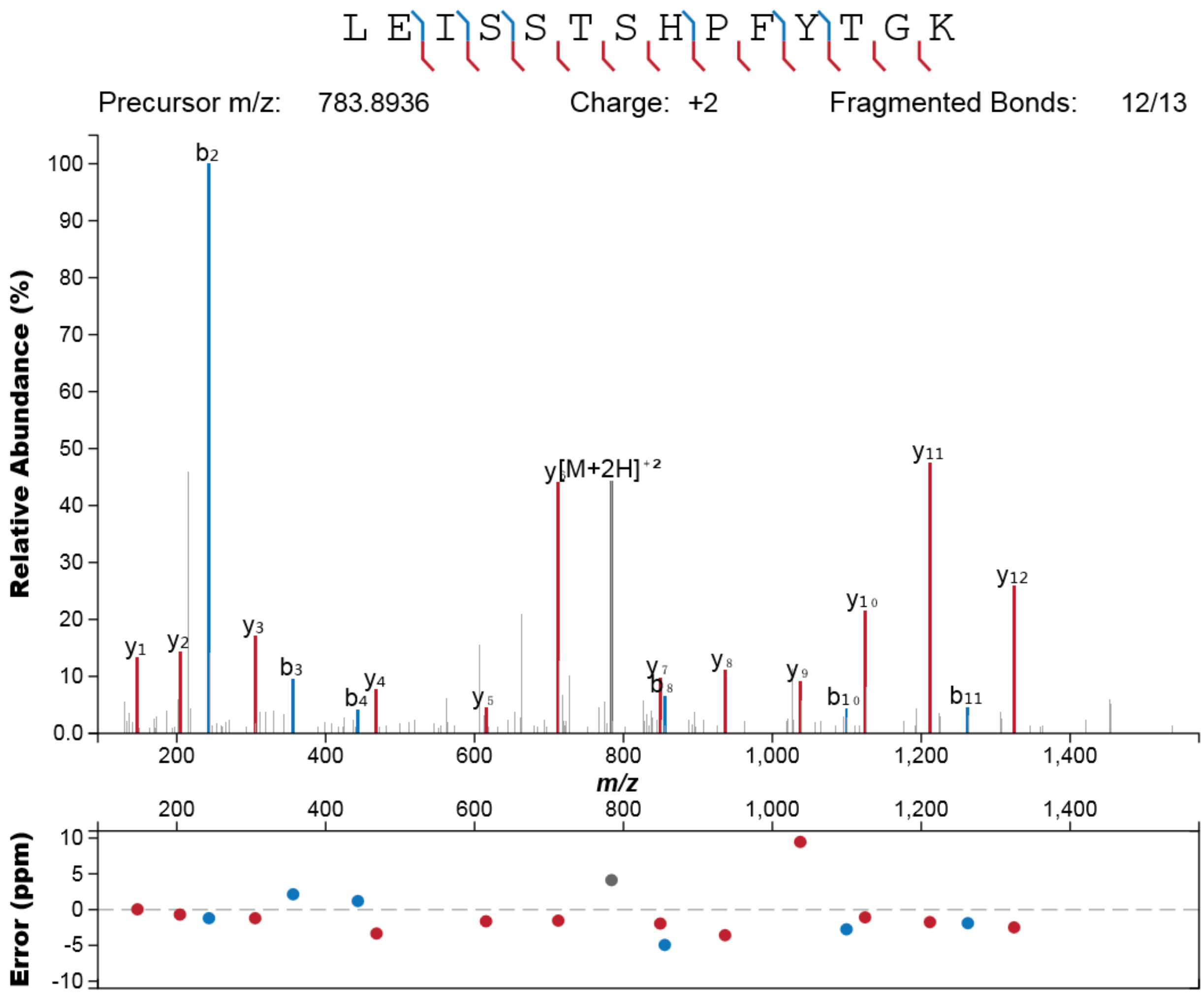

L E I S S T S H P F Y T G K
Precursor m/z: 783.8936
Charge: +2
Fragmented Bonds: 12/13
Relative Abundance (%)
m/z
Error (ppm)
b2
b3
b4
b8
b10
b11
y1
y2
y3
y4
y5
y7
y8
y9
y10
y11
y12
[M+2H]+2

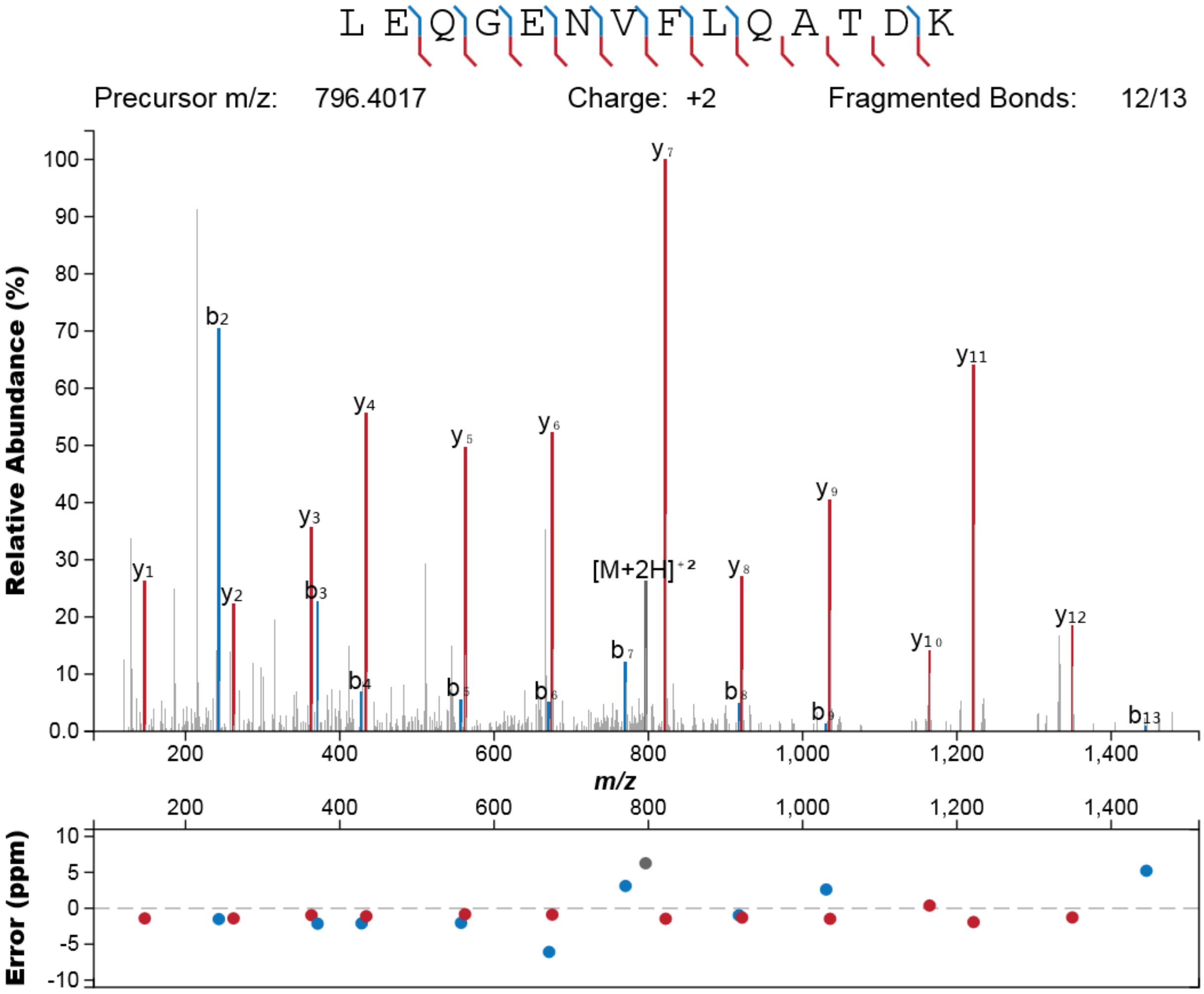

L E Q G E N V F L Q A T D K
Precursor m/z: 796.4017
Charge: +2
Fragmented Bonds: 12/13
Relative Abundance (%)
m/z
Error (ppm)
[M+2H]+2

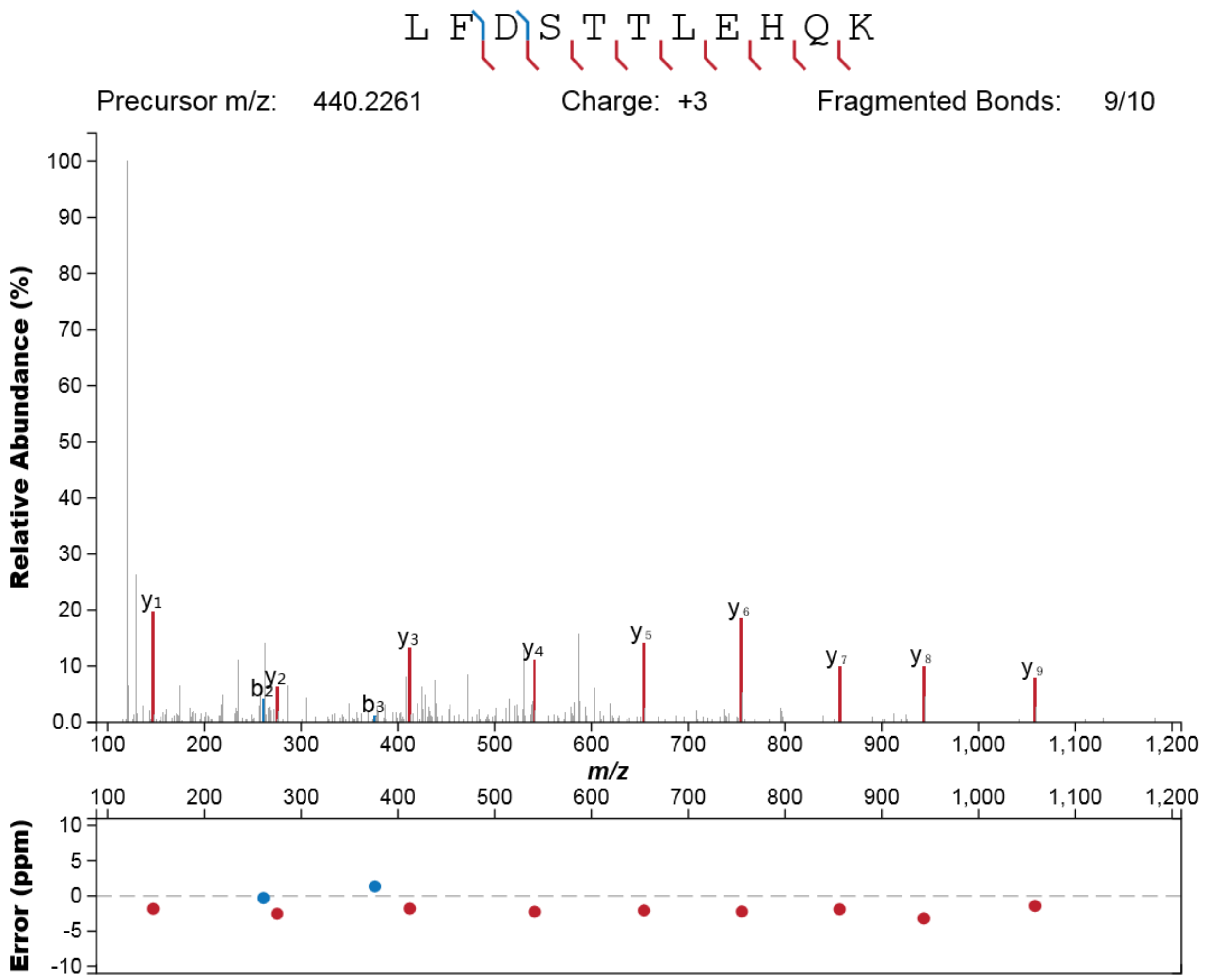

L F D S T T L E H Q K
Precursor m/z: 440.2261
Charge: +3
Fragmented Bonds: 9/10
Relative Abundance (%)
y1
b2
y2
b3
y3
y4
y5
y6
y7
y8
y9
m/z
Error (ppm)

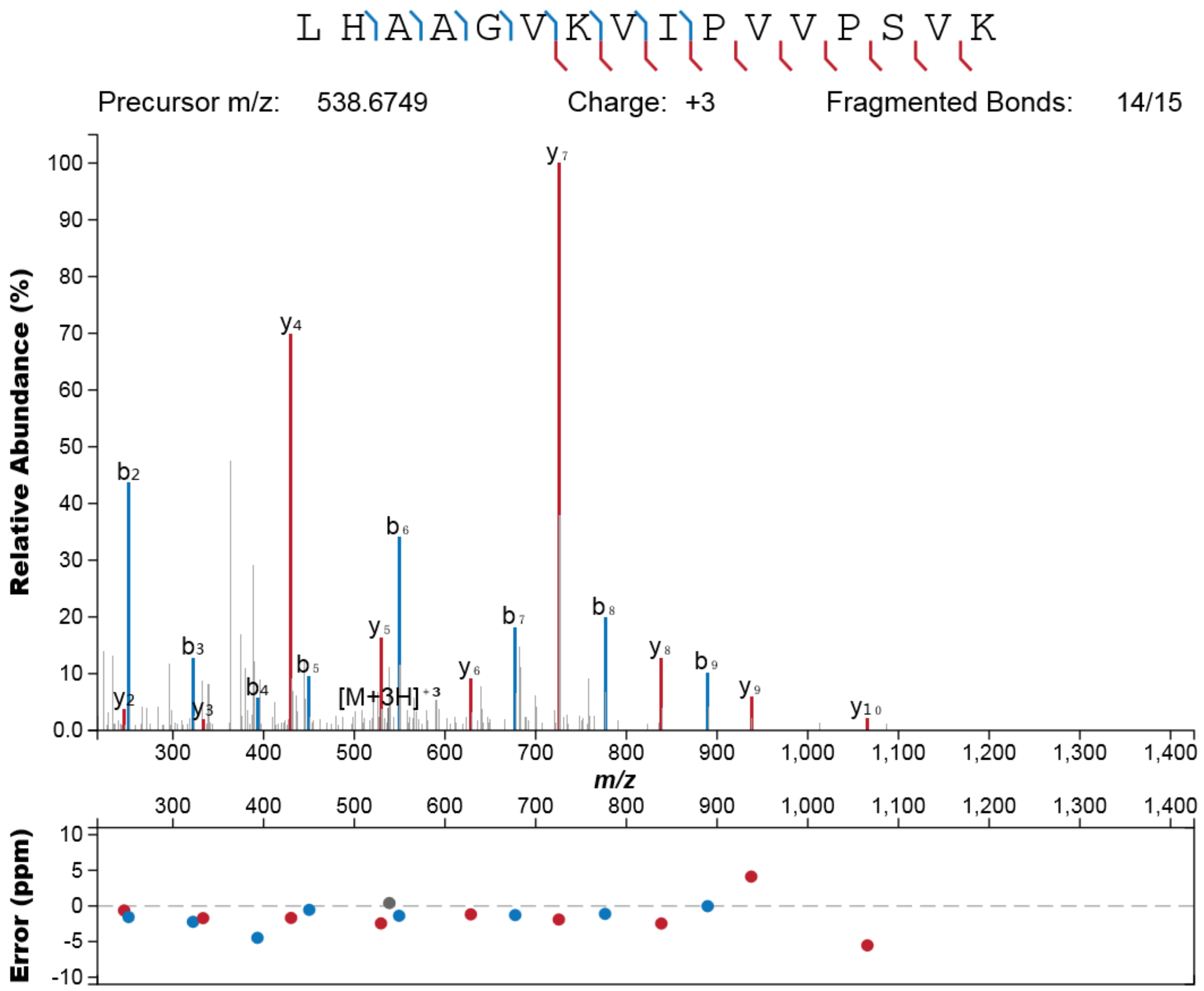
L H A A G V K V I P V V P S V K
Precursor m/z: 538.6749
Charge: +3
Fragmented Bonds: 14/15
Relative Abundance (%)
m/z
Error (ppm)
b2
b3
b4
b5
b6
b7
b8
b9
y2
y3
y4
y5
y6
y7
y8
y9
y10
[M+3H]+3

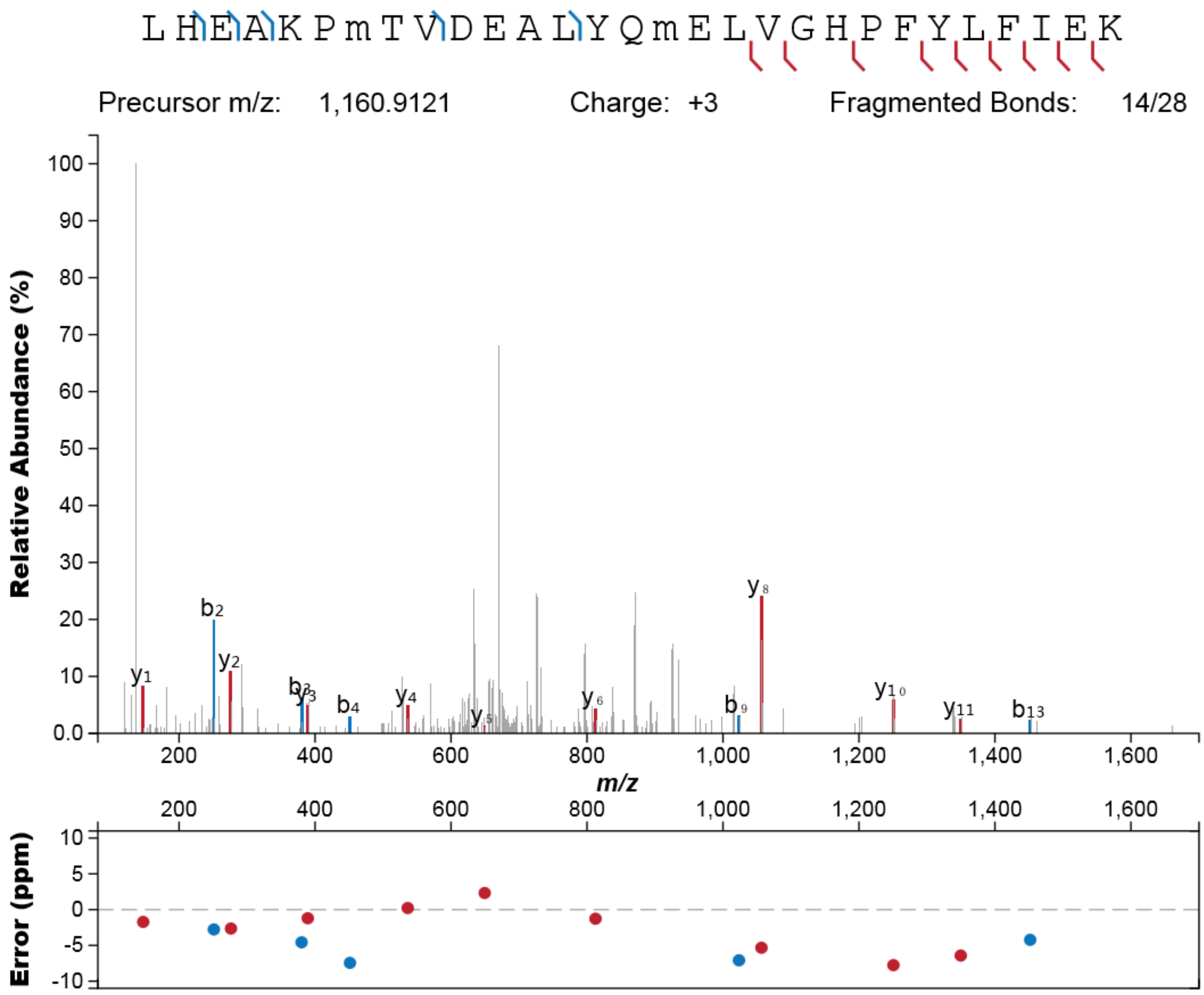

L H E A K P m T V D E A L Y Q m E L V G H P F Y L F I E K
Precursor m/z: 1,160.9121
Charge: +3
Fragmented Bonds: 14/28
Relative Abundance (%)
m/z
Error (ppm)

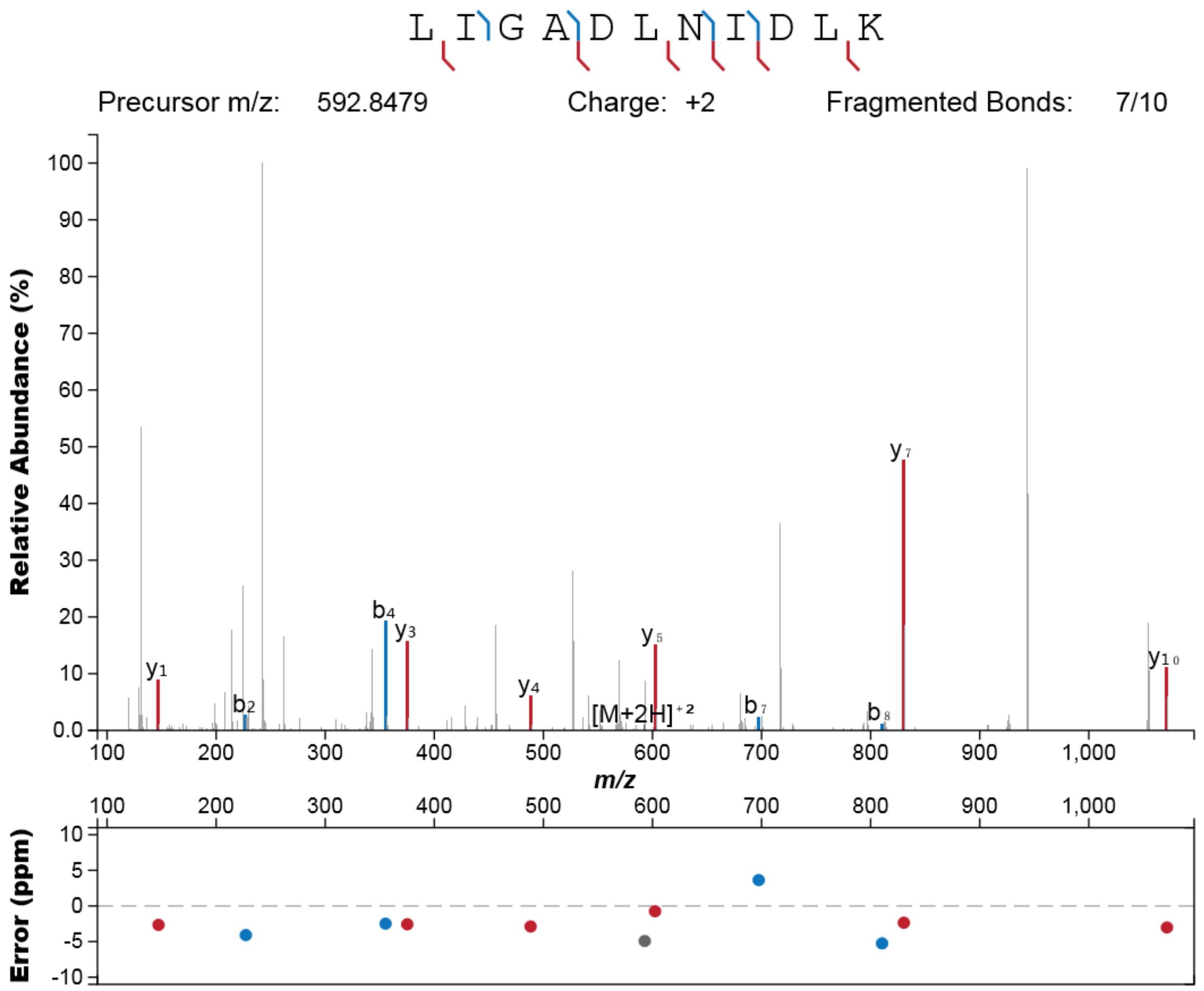
L I G A D L N I D L K
Precursor m/z: 592.8479
Charge: +2
Fragmented Bonds: 7/10
Relative Abundance (%)
m/z
Error (ppm)
y1
b2
b4
y3
y4
[M+2H]+2
y5
b7
b8
y7
y10

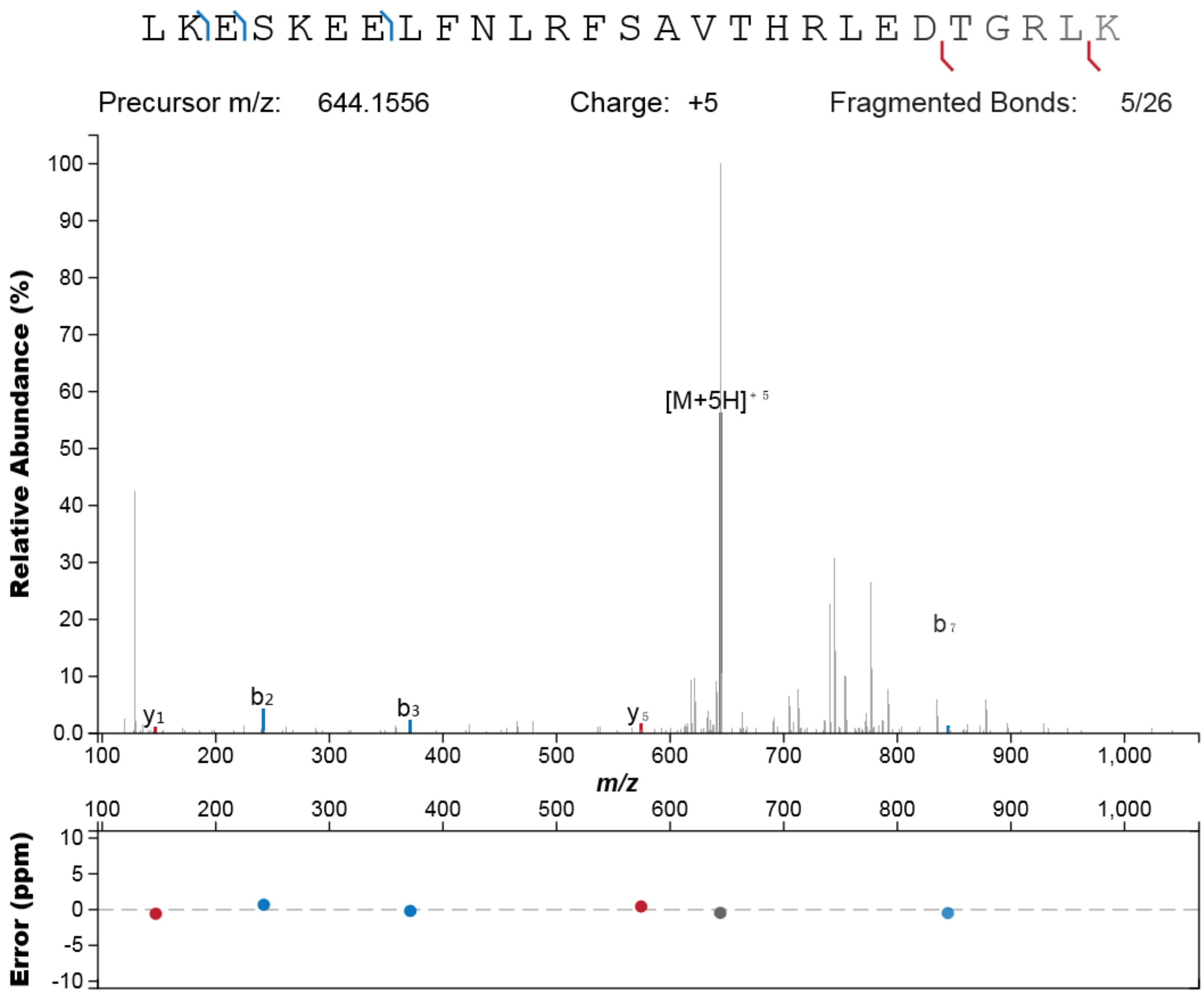

L K E S K E E L F N L R F S A V T H R L E D T G R L K
Precursor m/z: 644.1556
Charge: +5
Fragmented Bonds: 5/26
Relative Abundance (%)
[M+5H]+5
b2
b3
b7
y1
y5
m/z
Error (ppm)

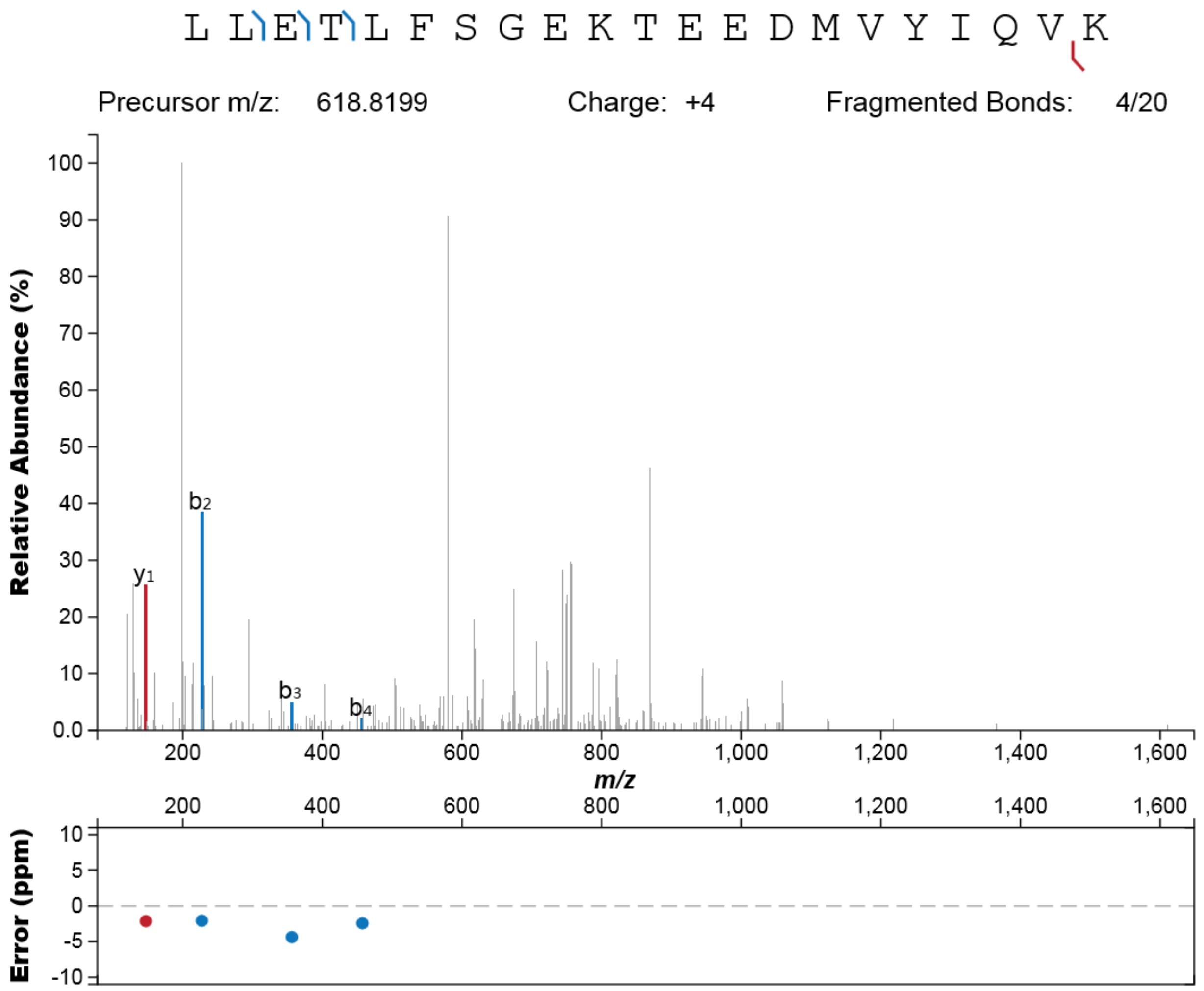

L L E T L F S G E K T E E D M V Y I Q V K
Precursor m/z: 618.8199
Charge: +4
Fragmented Bonds: 4/20
Relative Abundance (%)
m/z
Error (ppm)
y1
b2
b3
b4

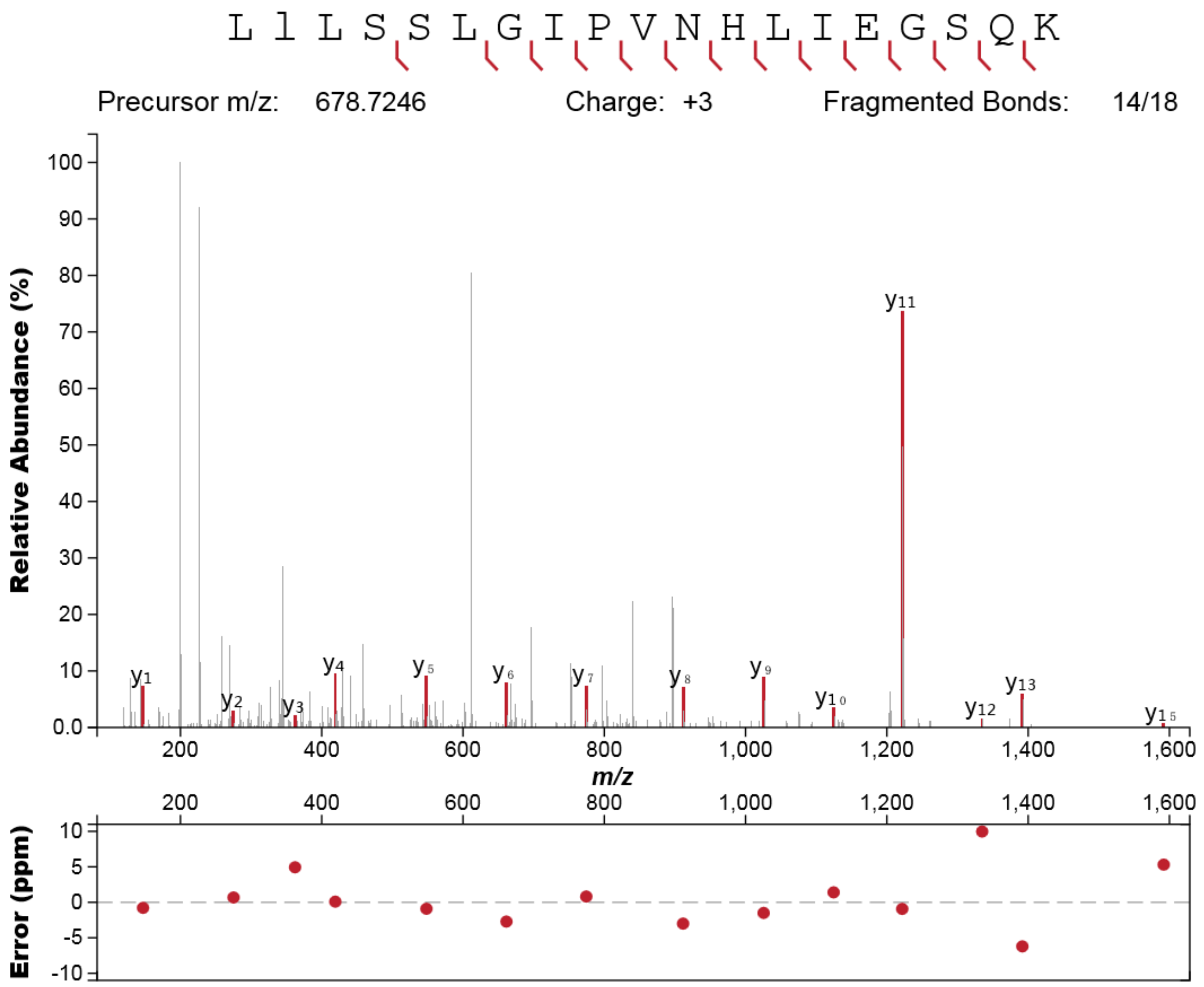

L l L S S L G I P V N H L I E G S Q K
Precursor m/z: 678.7246
Charge: +3
Fragmented Bonds: 14/18
Relative Abundance (%)
y11
y1
y2
y3
y4
y5
y6
y7
y8
y9
y10
y12
y13
y15
m/z
Error (ppm)

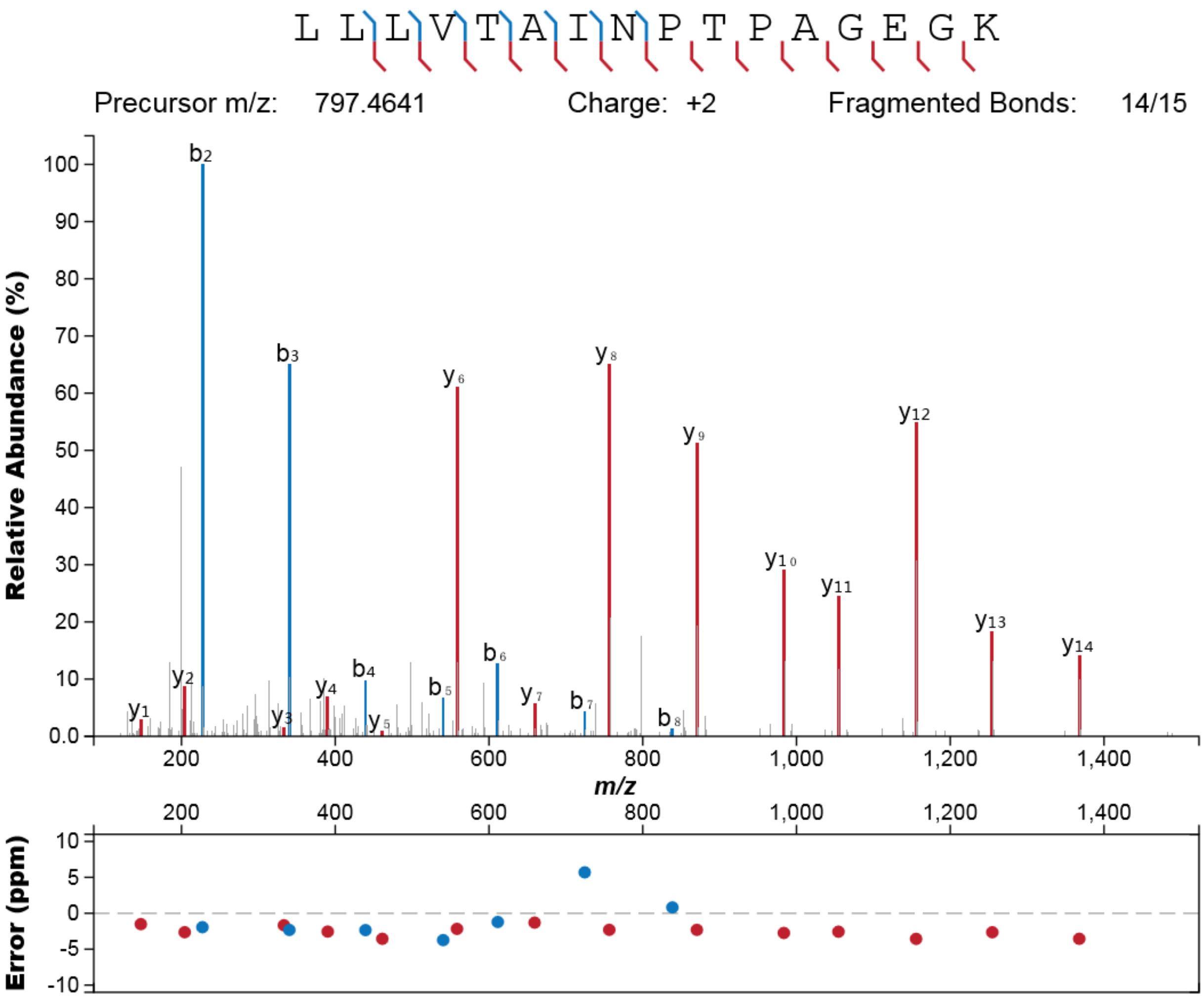

L L L V T A I N P T P A G E G K
Precursor m/z: 797.4641
Charge: +2
Fragmented Bonds: 14/15
Relative Abundance (%)
m/z
Error (ppm)
b2
b3
b4
b5
b6
b7
b8
y1
y2
y3
y4
y5
y6
y7
y8
y9
y10
y11
y12
y13
y14

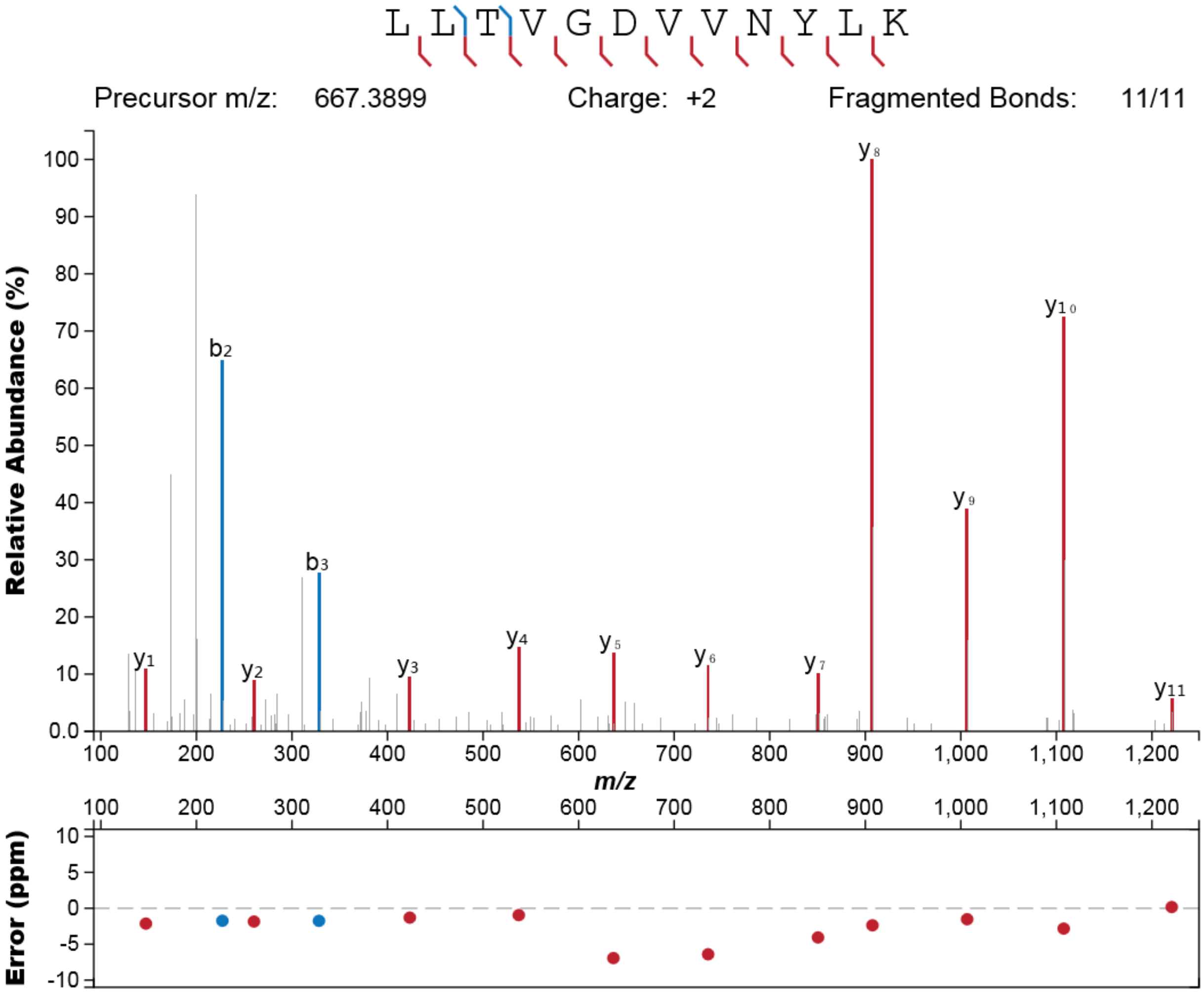

L L T V G D V V N Y L K
Precursor m/z: 667.3899
Charge: +2
Fragmented Bonds: 11/11
Relative Abundance (%)
m/z
Error (ppm)
b2
b3
y1
y2
y3
y4
y5
y6
y7
y8
y9
y10
y11

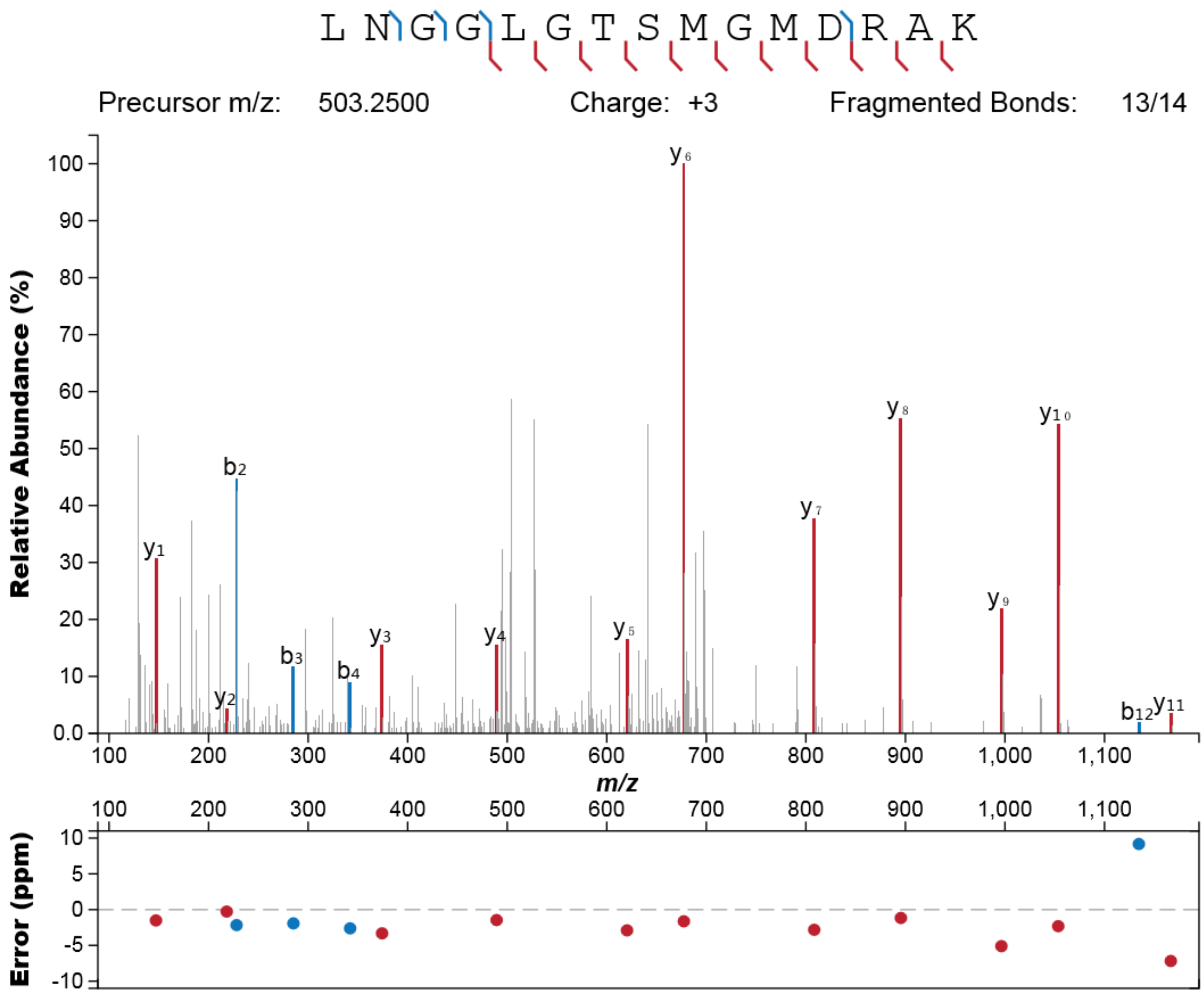

L N G G L G T S M G M D R A K
Precursor m/z: 503.2500
Charge: +3
Fragmented Bonds: 13/14
Relative Abundance (%)
m/z
Error (ppm)
y1
y2
b2
b3
b4
y3
y4
y5
y6
y7
y8
y9
y10
b12
y11

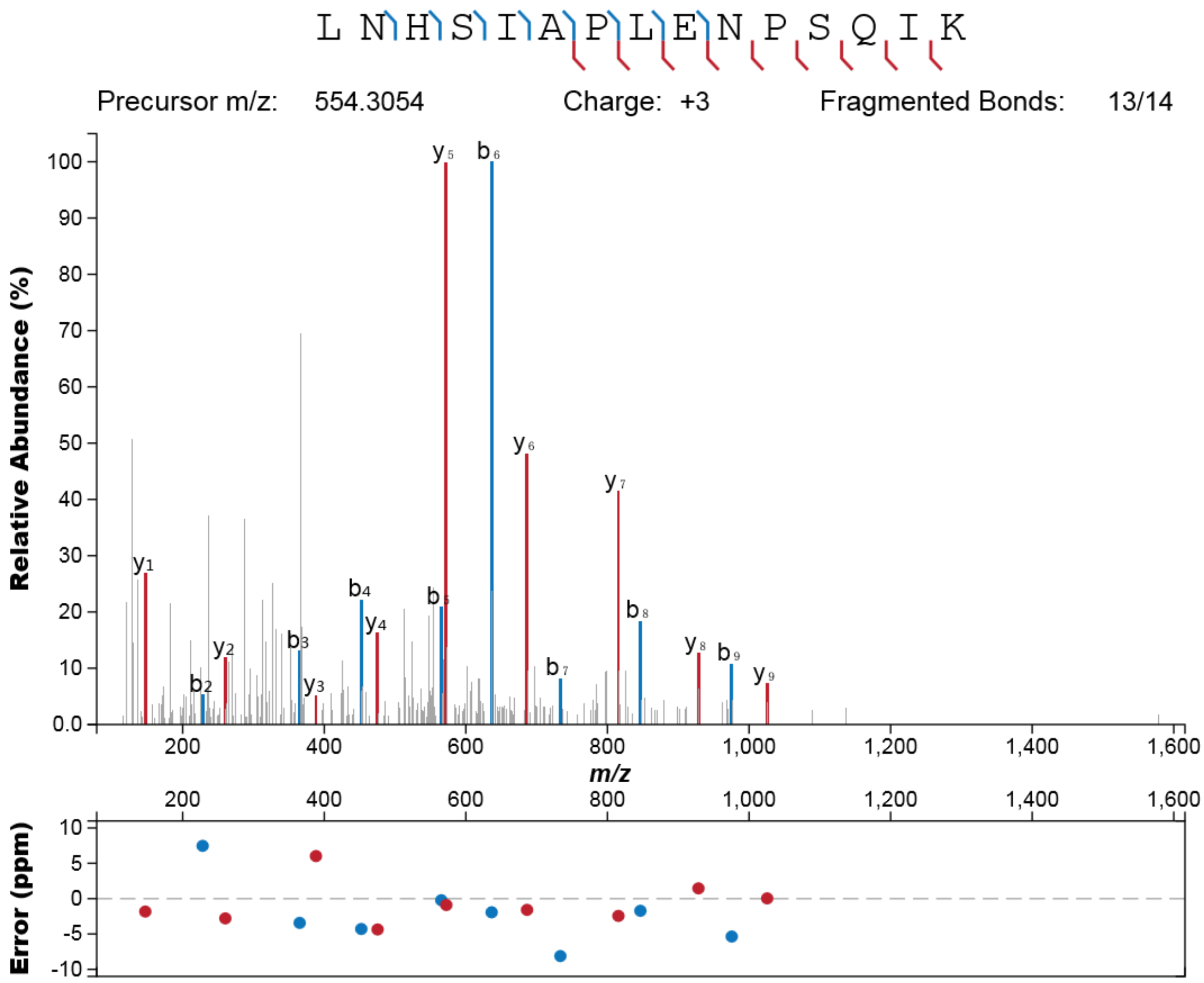

L N H S I A P L E N P S Q I K
Precursor m/z: 554.3054
Charge: +3
Fragmented Bonds: 13/14
Relative Abundance (%)
m/z
Error (ppm)
y1 b2 y2 b3 y3 b4 y4 b5 y5 b6 y6 b7 y7 b8 y8 b9 y9

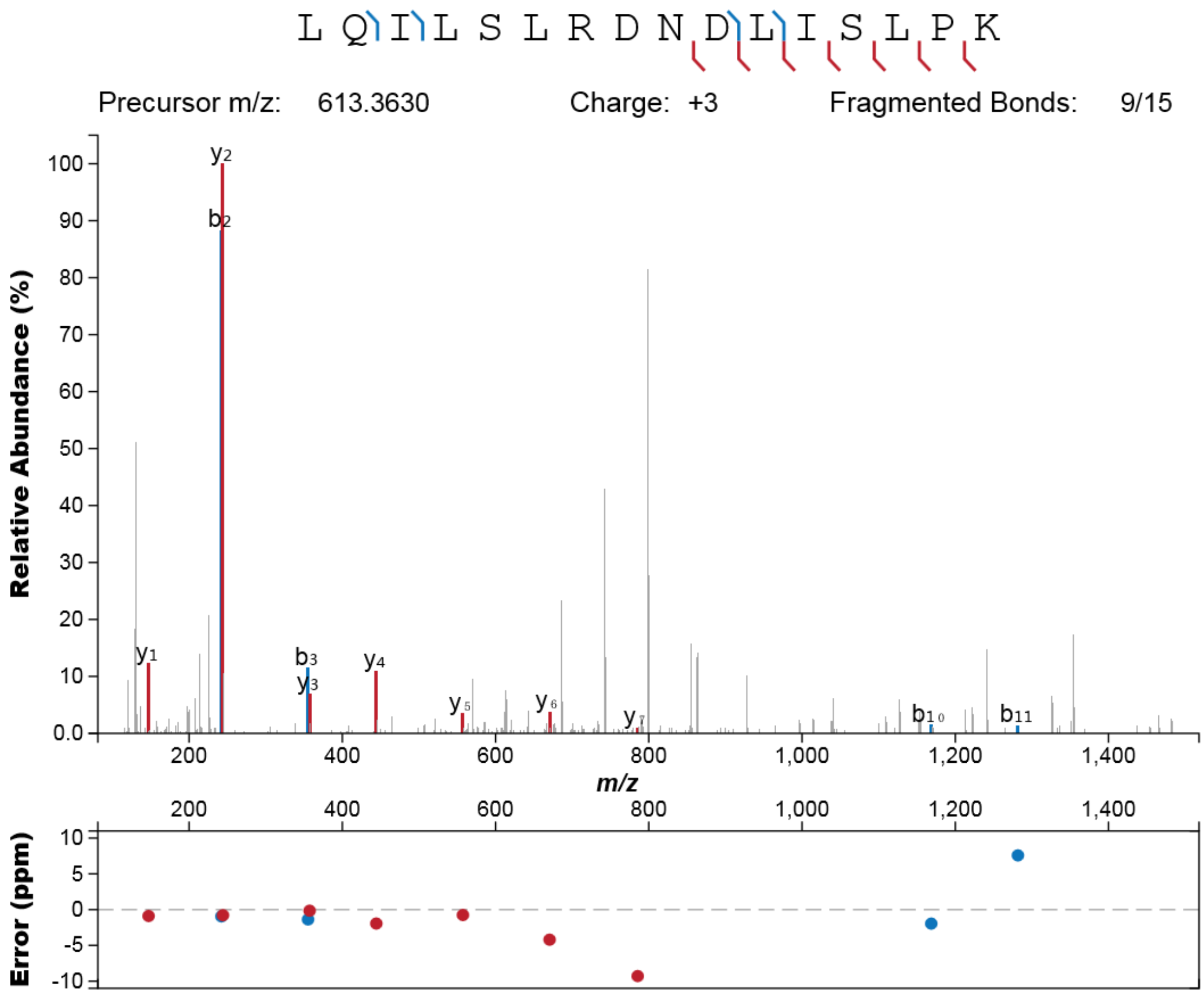

L Q I L S L R D N D L I S L P K
Precursor m/z: 613.3630
Charge: +3
Fragmented Bonds: 9/15
Relative Abundance (%)
y2
b2
y1
b3
y3
y4
y5
y6
y7
b10
b11
m/z
Error (ppm)

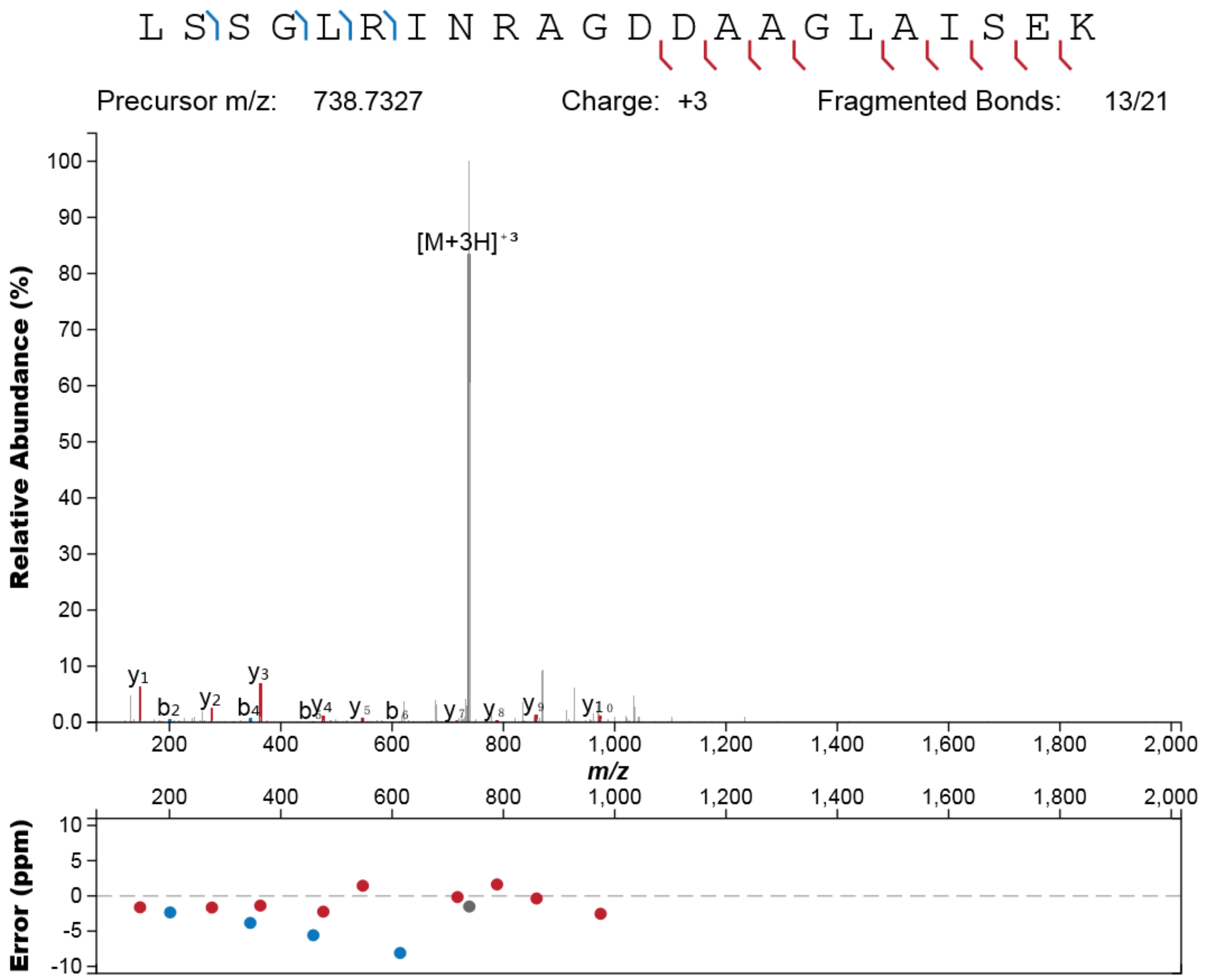

L S S G L R I N R A G D D A A G L A I S E K
Precursor m/z: 738.7327
Charge: +3
Fragmented Bonds: 13/21
Relative Abundance (%)
[M+3H]+3
m/z
Error (ppm)

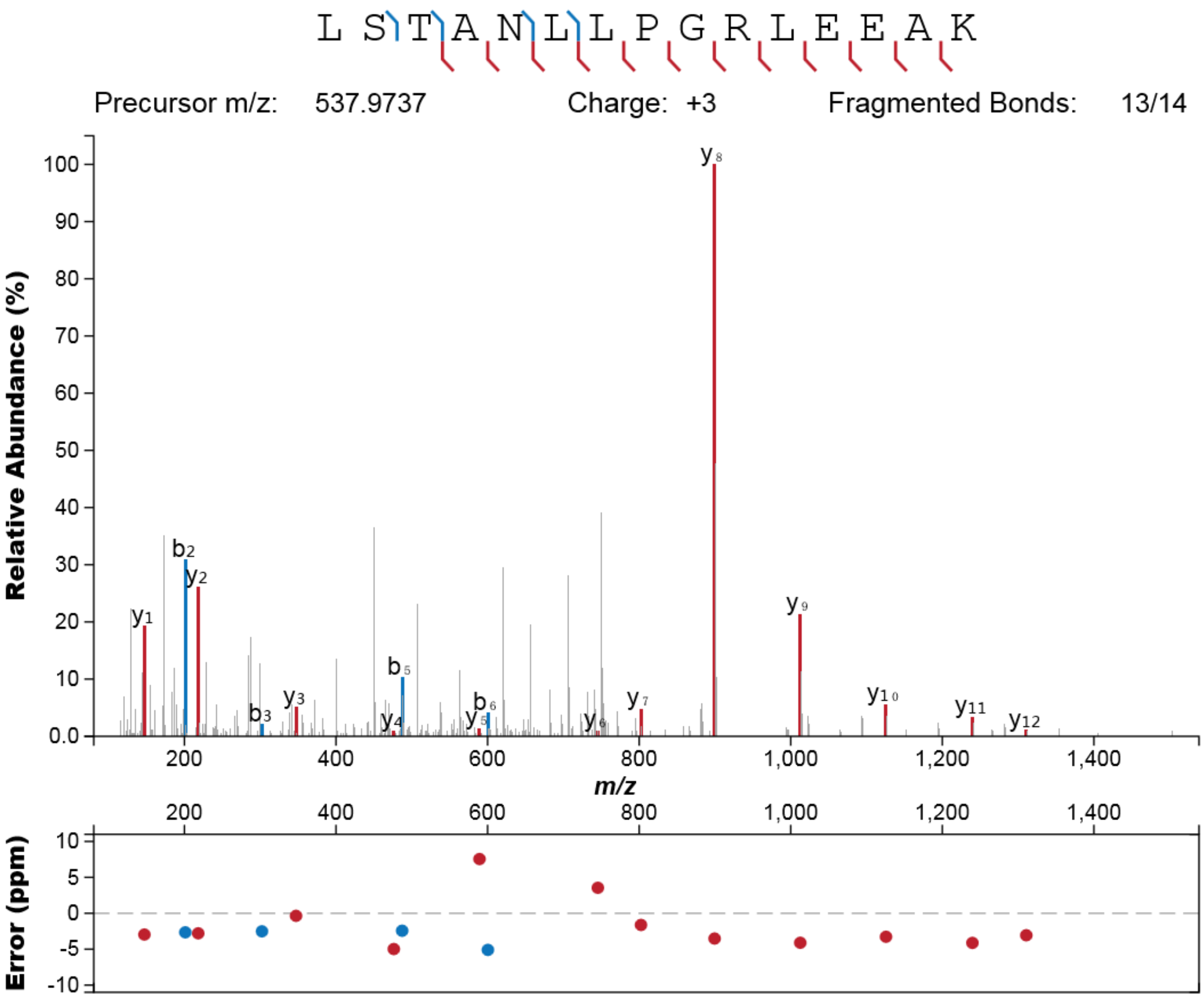

L S T A N L L P G R L E E A K
Precursor m/z: 537.9737
Charge: +3
Fragmented Bonds: 13/14
Relative Abundance (%)
m/z
Error (ppm)
y1 b2 y2 b3 y3 y4 b5 y5 b6 y6 y7 y8 y9 y10 y11 y12

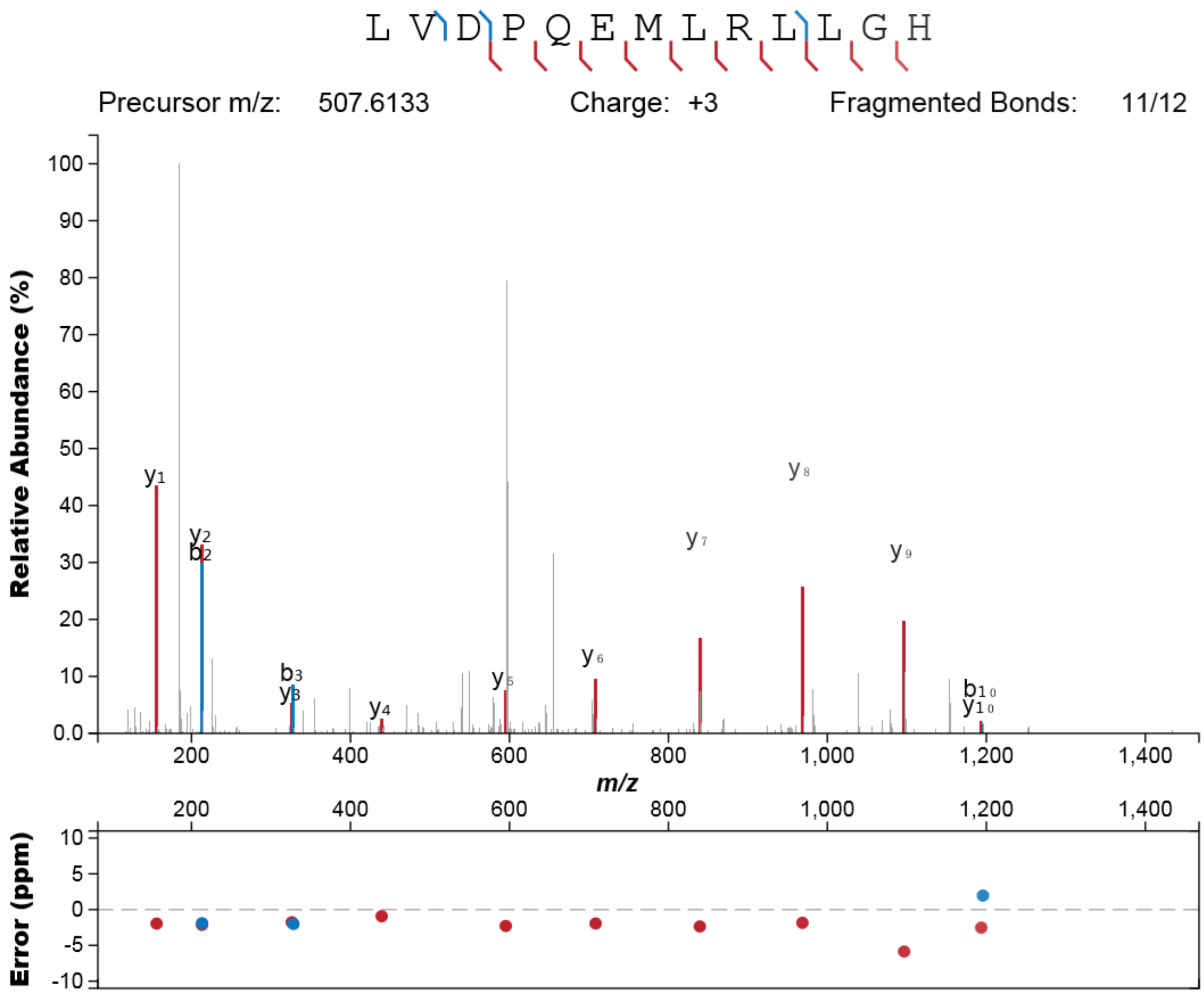

L V D P Q E M L R L L G H
Precursor m/z: 507.6133
Charge: +3
Fragmented Bonds: 11/12
Relative Abundance (%)
m/z
Error (ppm)
y1
y2
b2
b3
y3
y4
y5
y6
y7
y8
y9
b10
y10

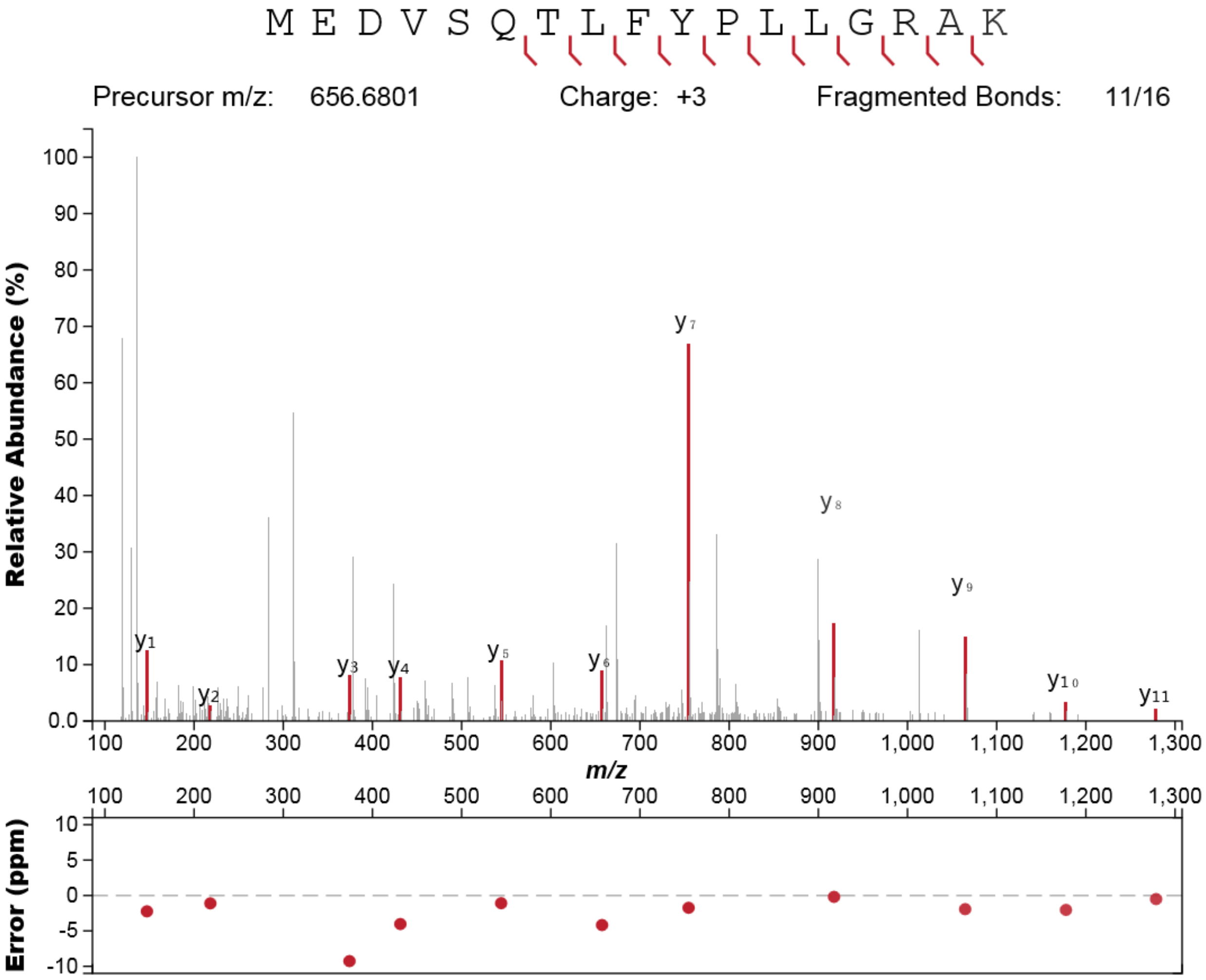

M E D V S Q T L F Y P L L G R A K
Precursor m/z: 656.6801
Charge: +3
Fragmented Bonds: 11/16
Relative Abundance (%)
m/z
Error (ppm)
y1
y2
y3
y4
y5
y6
y7
y8
y9
y10
y11

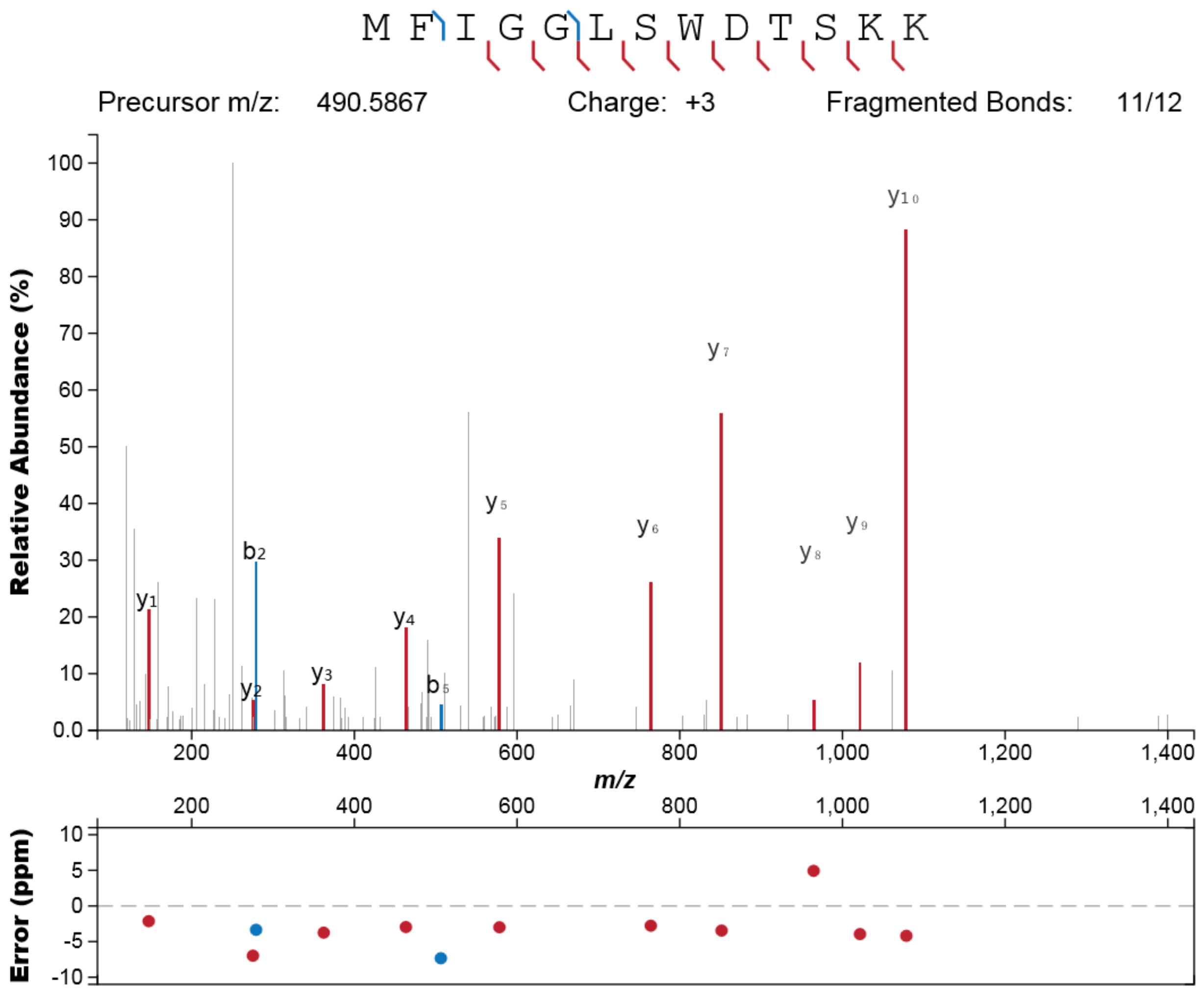
M F I G G L S W D T S K K
Precursor m/z: 490.5867
Charge: +3
Fragmented Bonds: 11/12
Relative Abundance (%)
m/z
Error (ppm)
y1
y2
b2
y3
y4
b5
y5
y6
y7
y8
y9
y10

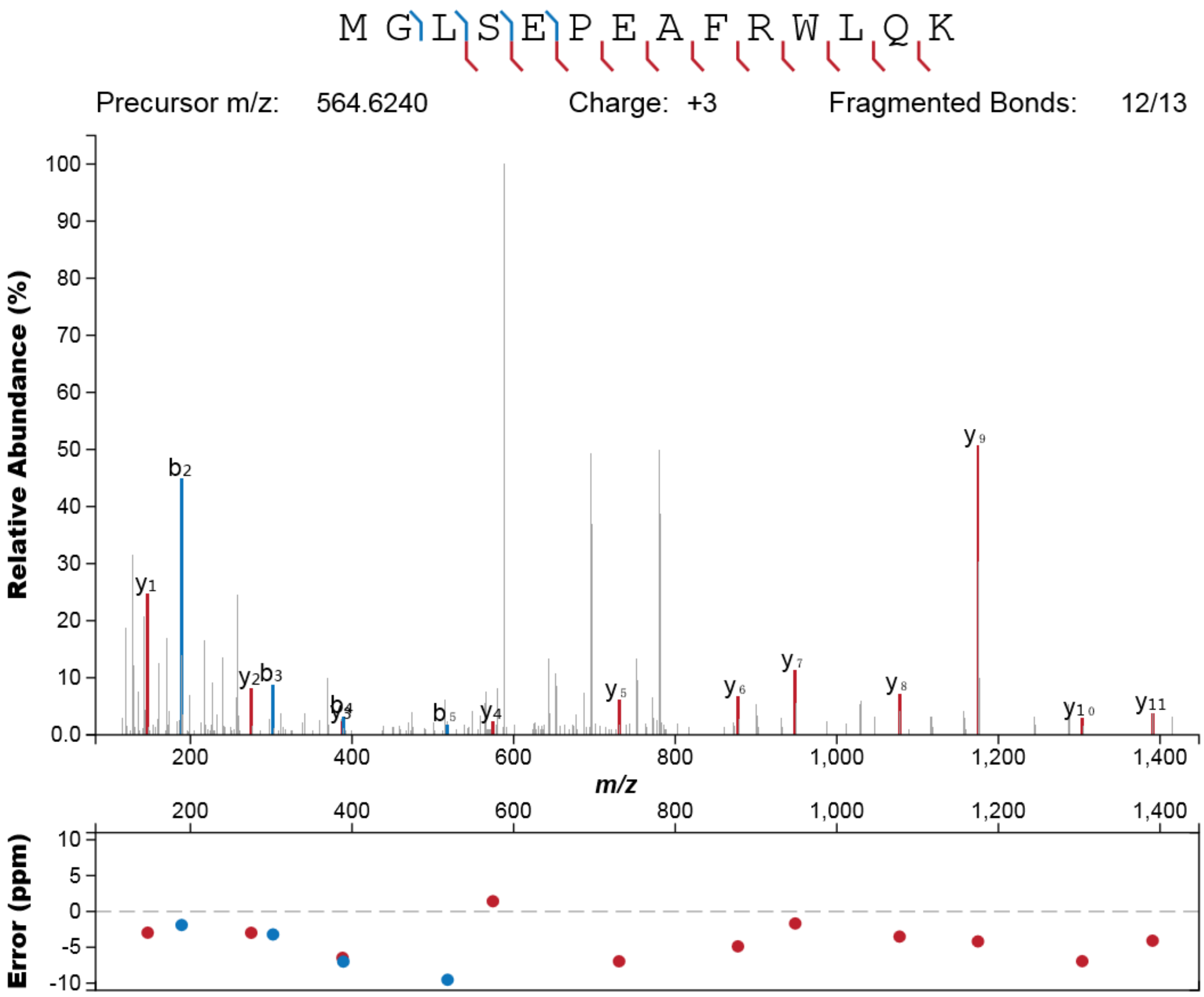

M G L S E P E A F R W L Q K
Precursor m/z: 564.6240
Charge: +3
Fragmented Bonds: 12/13
Relative Abundance (%)
m/z
Error (ppm)

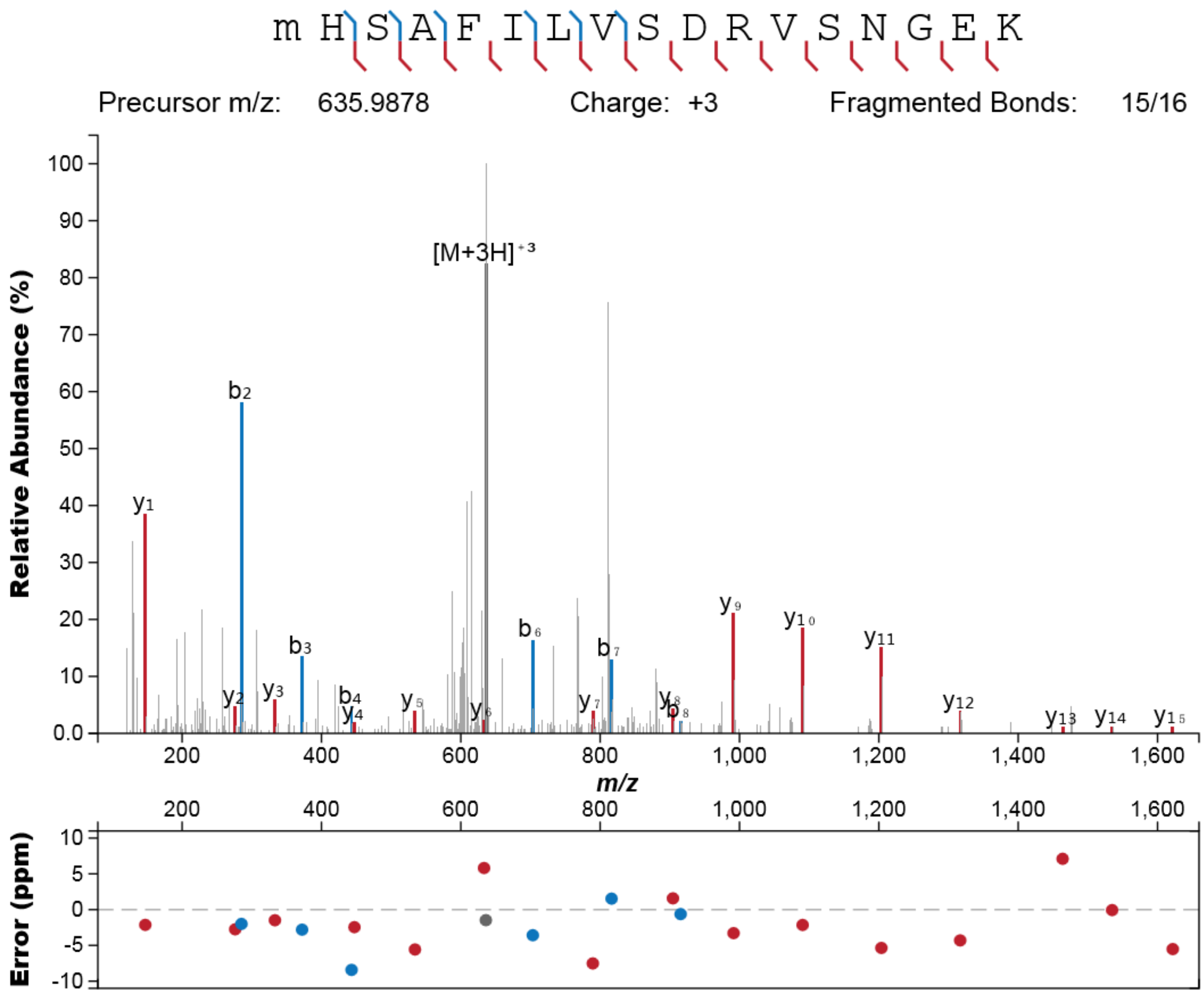

m H S A F I L V S D R V S N G E K
Precursor m/z: 635.9878
Charge: +3
Fragmented Bonds: 15/16
Relative Abundance (%)
[M+3H]+3
m/z
Error (ppm)

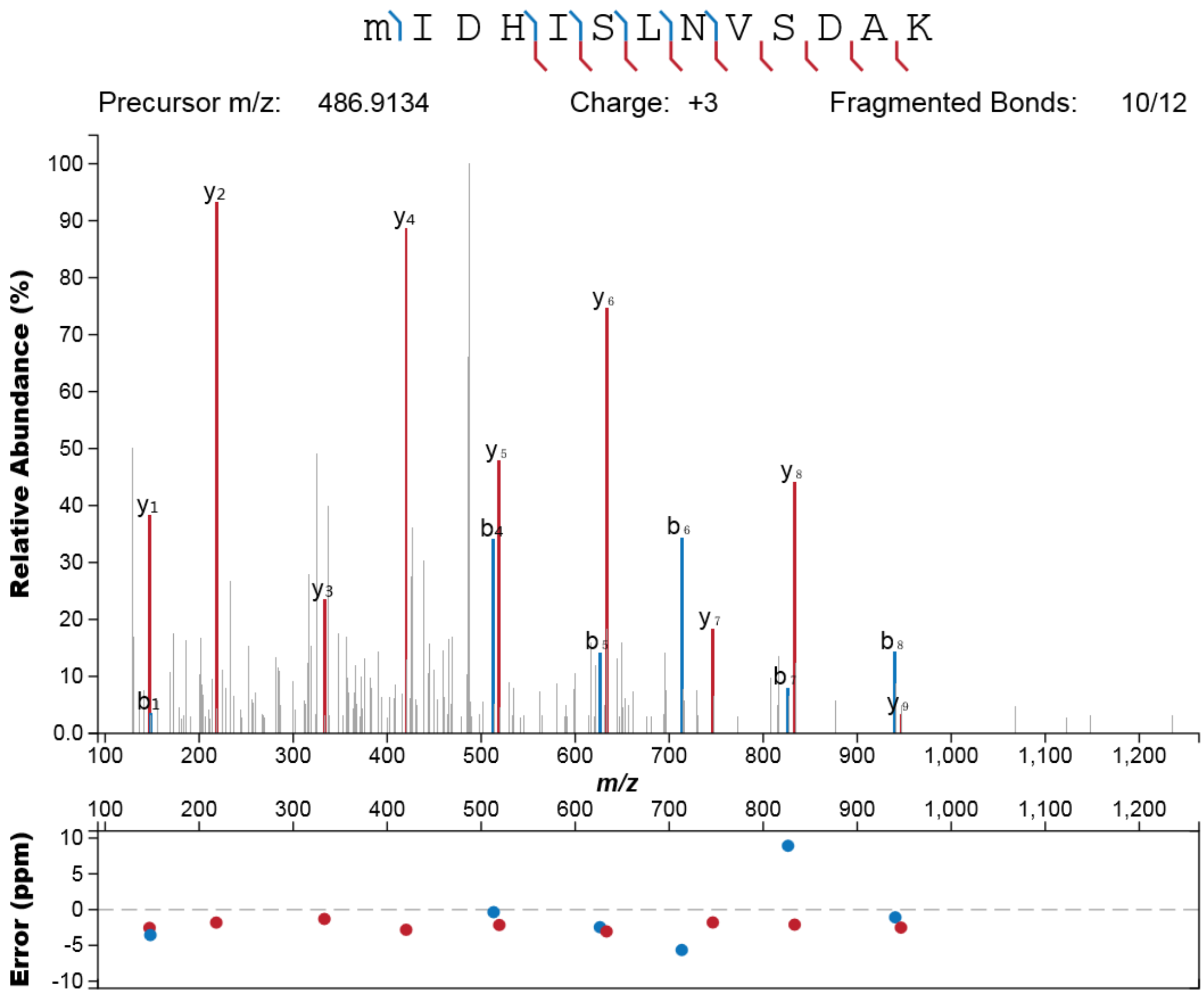
m I D H I S L N V S D A K
Precursor m/z: 486.9134
Charge: +3
Fragmented Bonds: 10/12
Relative Abundance (%)
m/z
Error (ppm)
y1
y2
y3
y4
y5
y6
y7
y8
y9
b1
b4
b5
b6
b7
b8

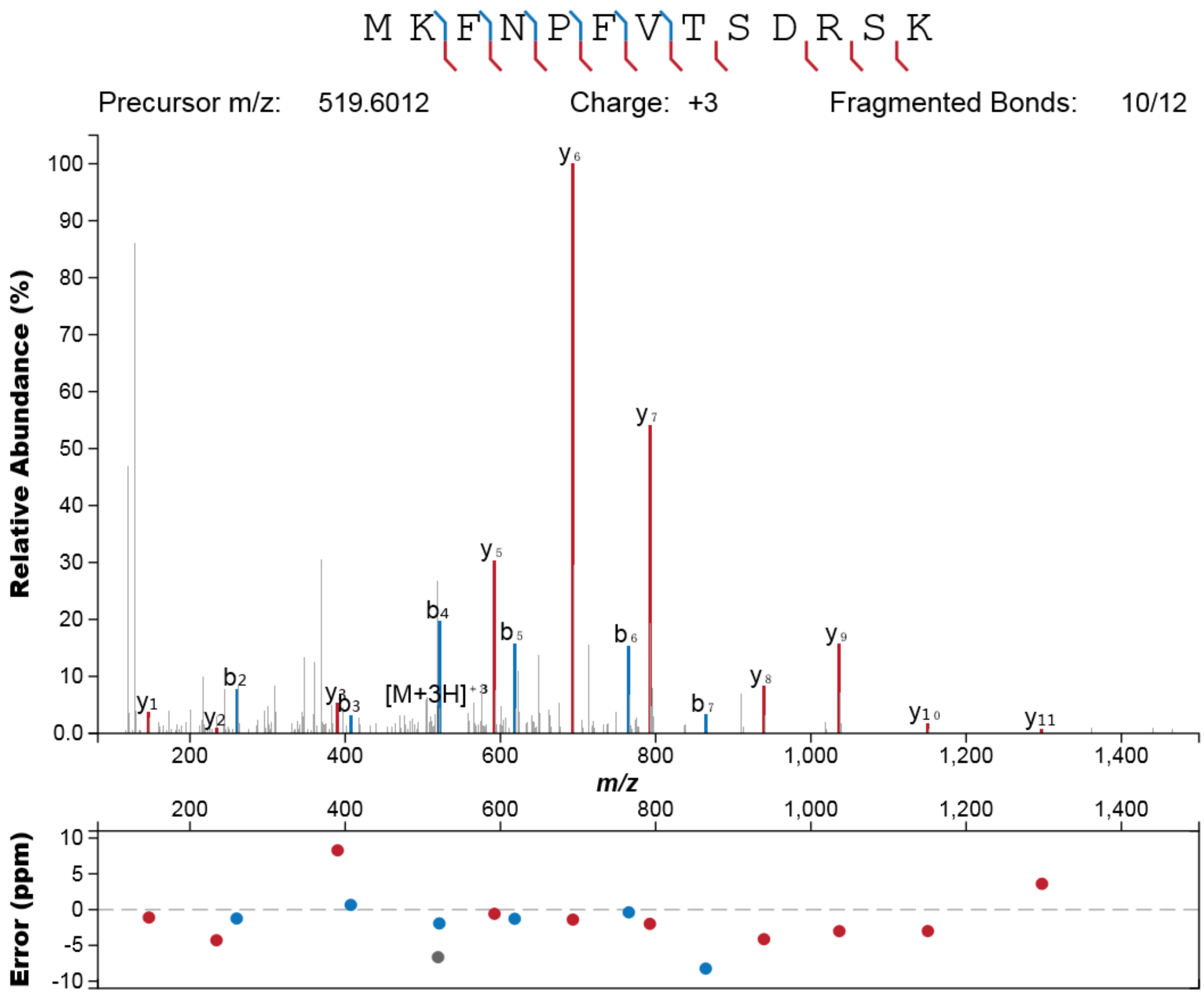
M K F N P F V T S D R S K
Precursor m/z: 519.6012
Charge: +3
Fragmented Bonds: 10/12
Relative Abundance (%)
m/z
Error (ppm)
y1 y2 b2 y3 b3 [M+3H]+3 b4 y5 b5 y6 b6 y7 b7 y8 y9 y10 y11

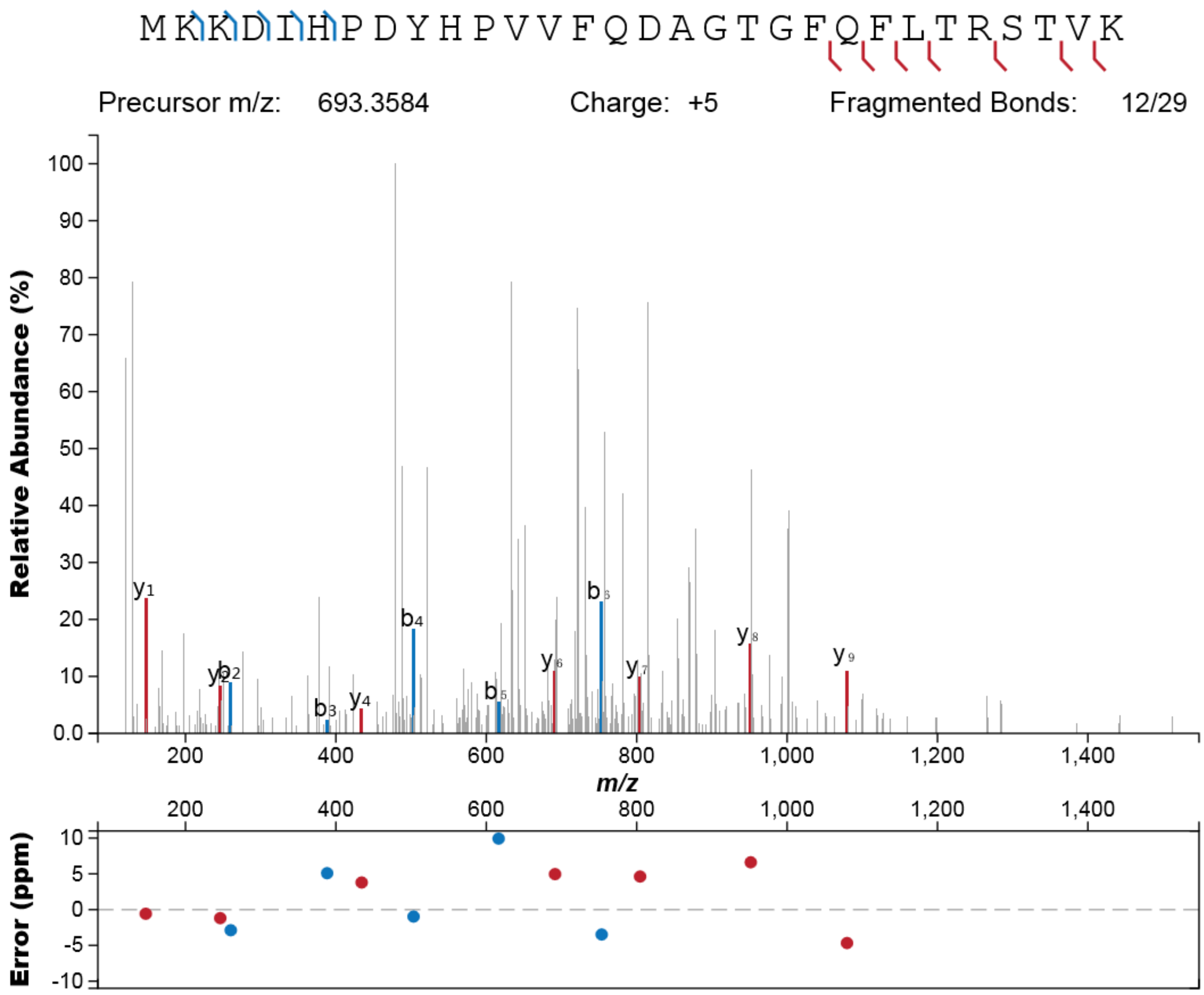

M K K D I H P D Y H P V V F Q D A G T G F Q F L T R S T V K
Precursor m/z: 693.3584
Charge: +5
Fragmented Bonds: 12/29
Relative Abundance (%)
m/z
Error (ppm)

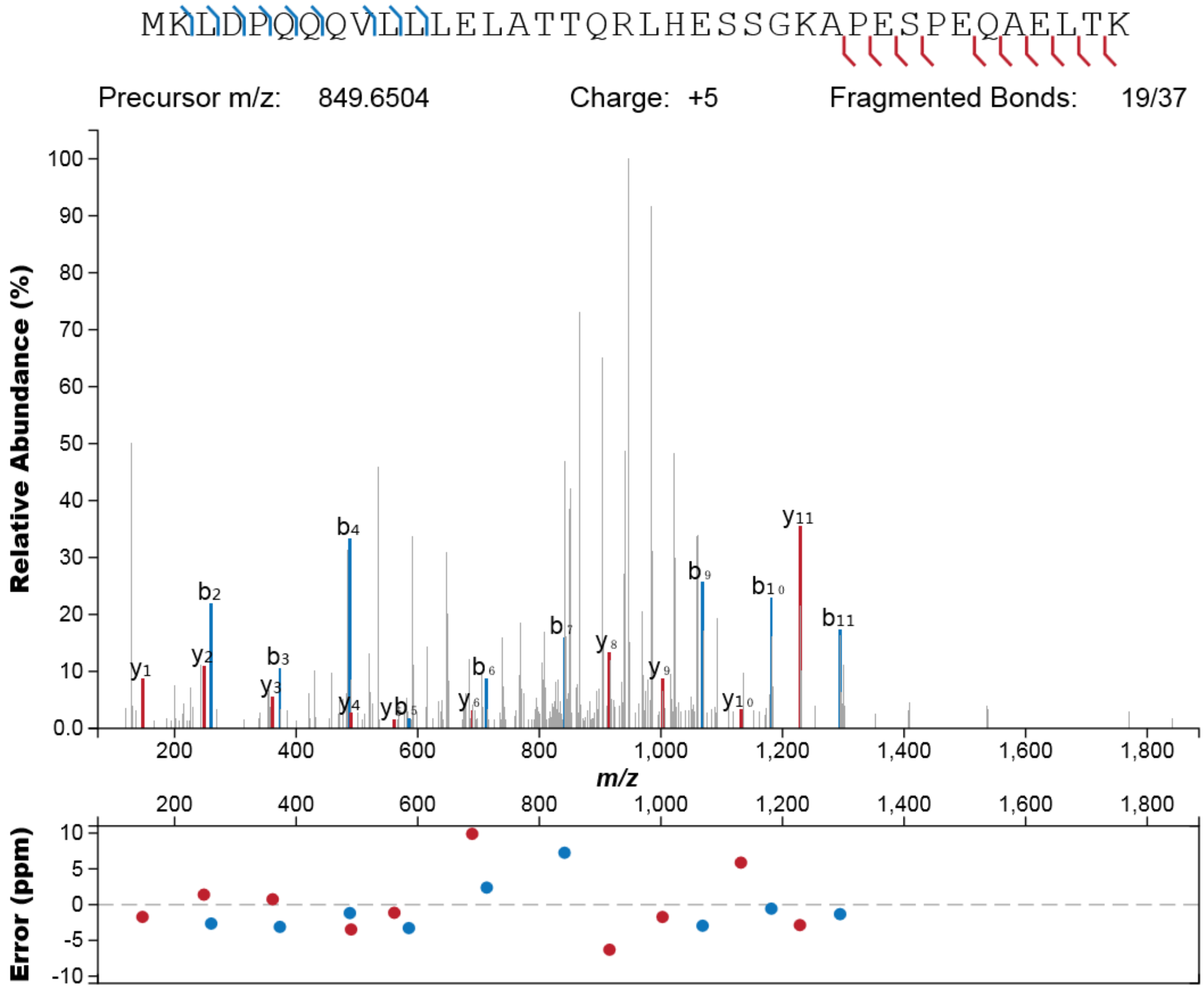
MKLDPQQQVLLLELATTQRLHESSGKAPESPEQAELTK
Precursor m/z: 849.6504
Charge: +5
Fragmented Bonds: 19/37
Relative Abundance (%)
m/z
Error (ppm)
y1
y2
y3
y4
yb5
y6
y8
y9
y10
y11
b2
b3
b4
b6
b7
b9
b10
b11

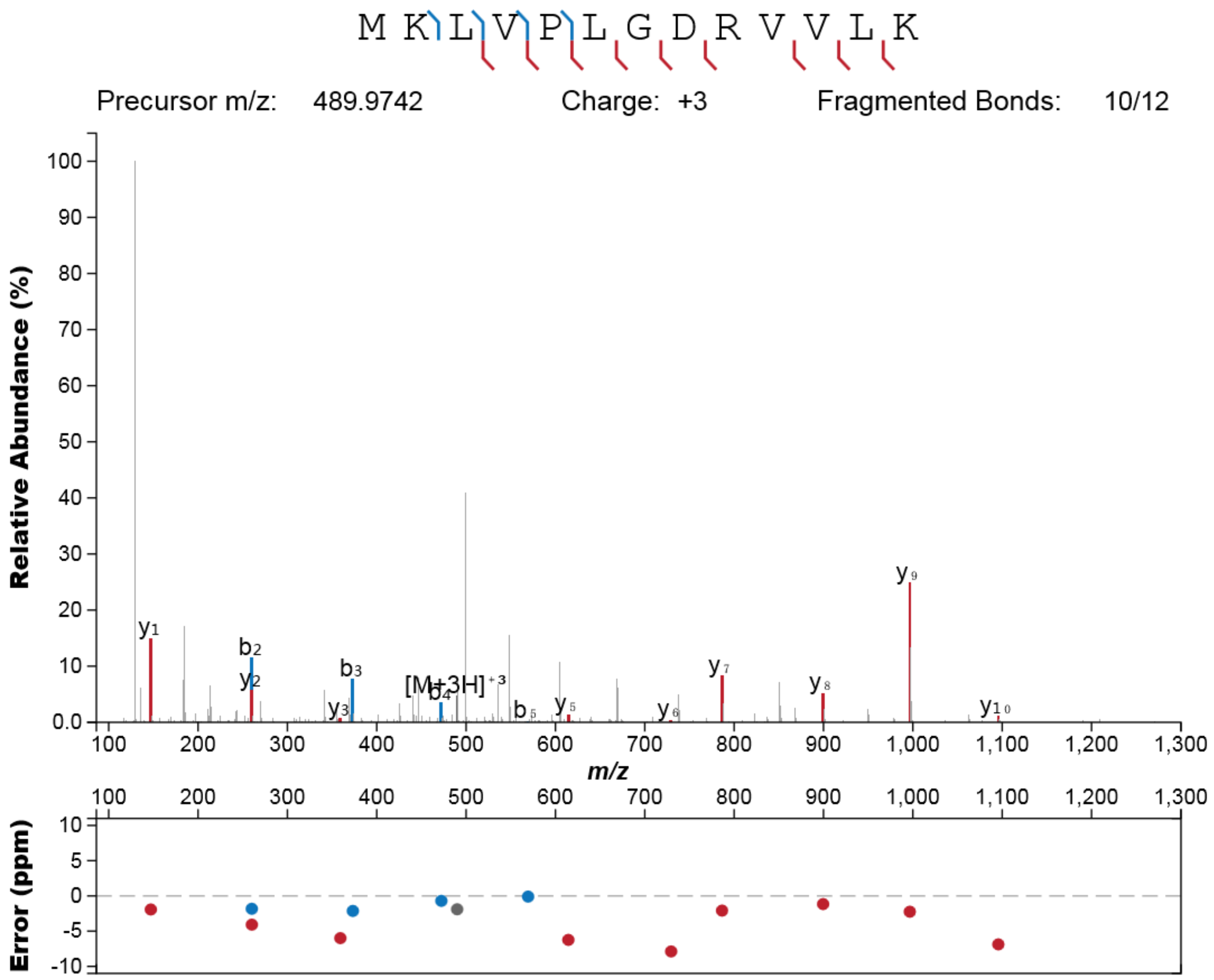

M K L V P L G D R V V L K
Precursor m/z: 489.9742
Charge: +3
Fragmented Bonds: 10/12
Relative Abundance (%)
m/z
Error (ppm)
y1
b2
y2
b3
y3
[M+3H]+3
b4
b5
y5
y6
y7
y8
y9
y10

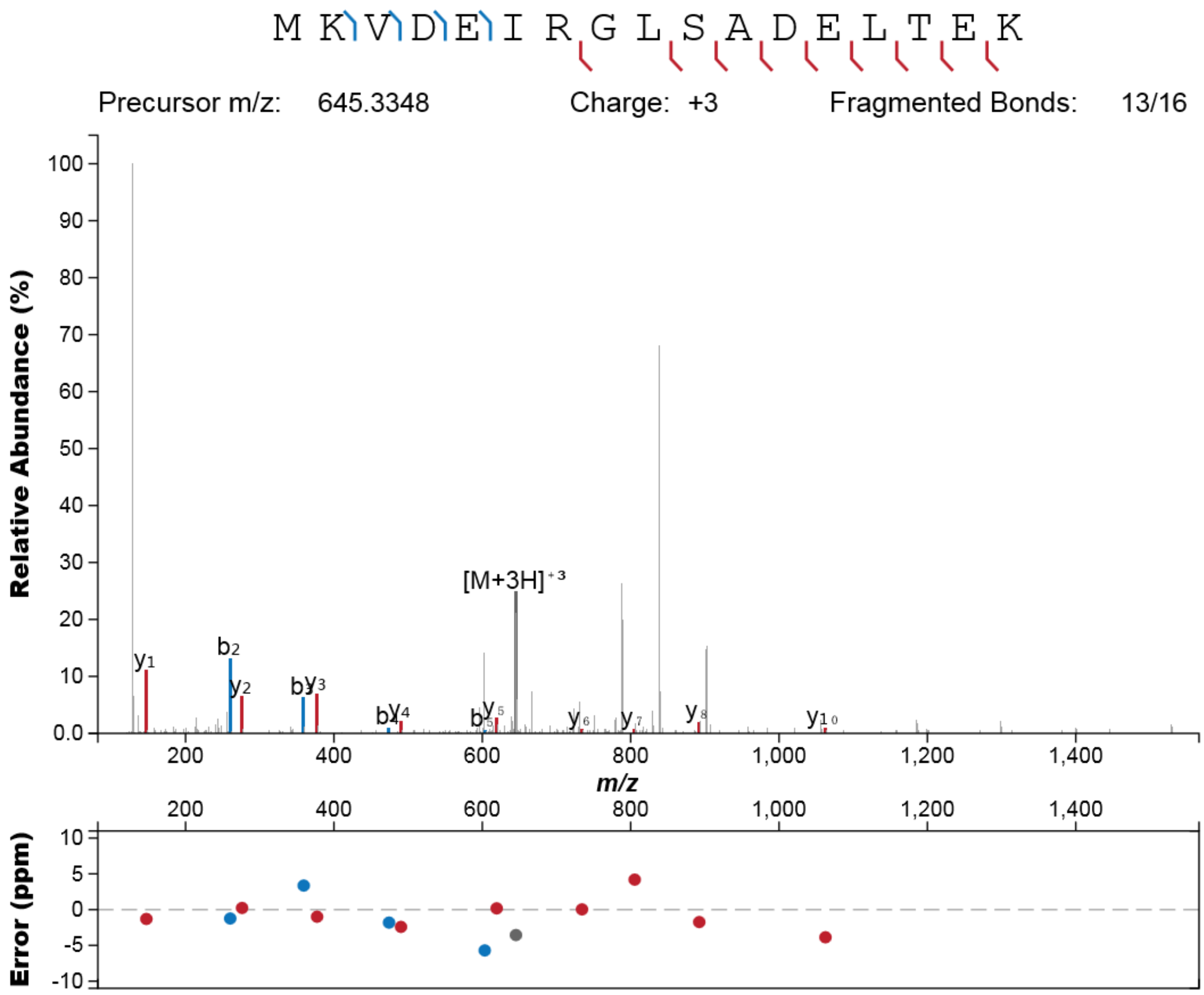

M K V D E I R G L S A D E L T E K
Precursor m/z: 645.3348
Charge: +3
Fragmented Bonds: 13/16
Relative Abundance (%)
[M+3H]+3
m/z
Error (ppm)

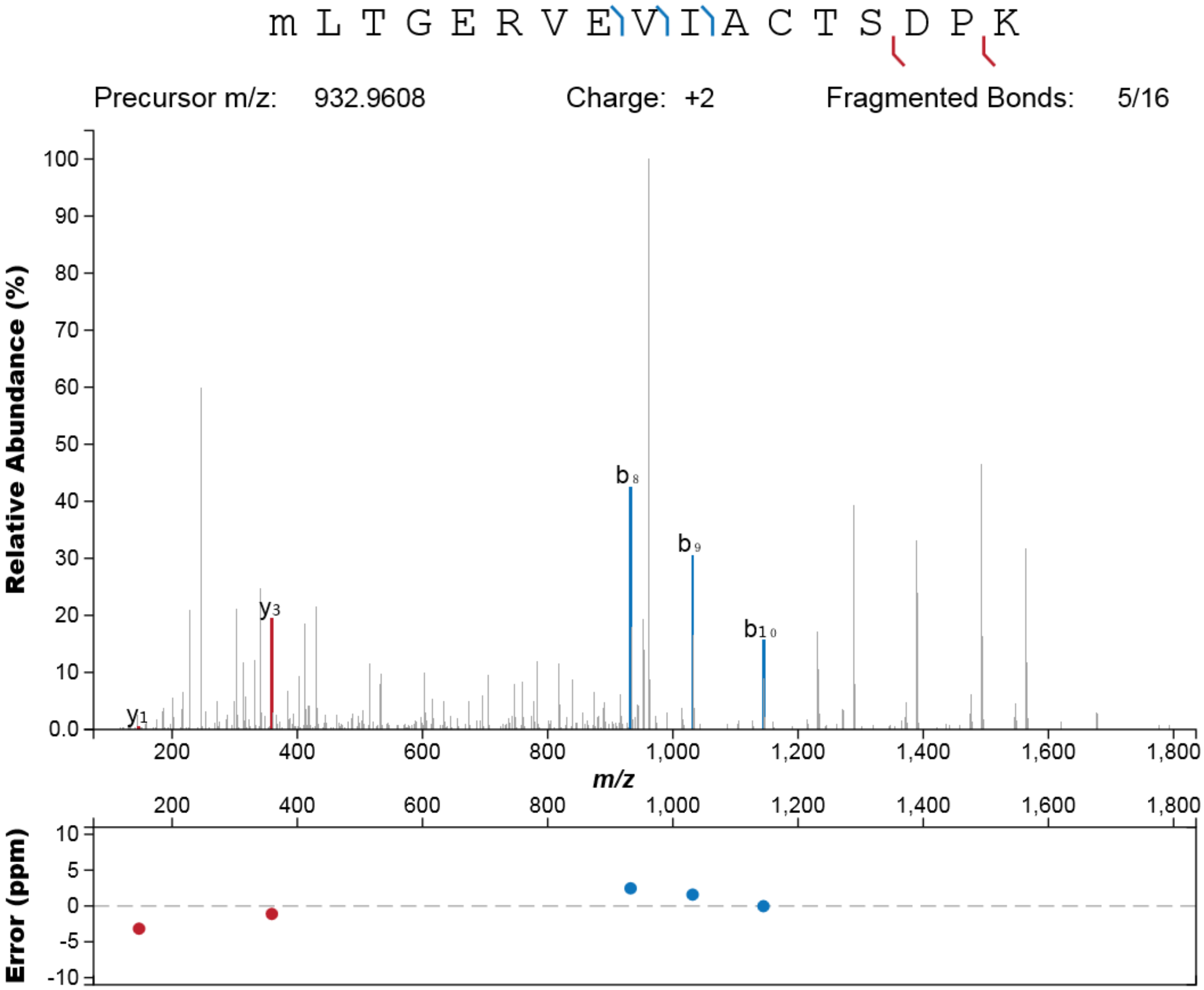

m L T G E R V E V I A C T S D P K
Precursor m/z: 932.9608
Charge: +2
Fragmented Bonds: 5/16
Relative Abundance (%)
m/z
Error (ppm)
b8
b9
b10
y1
y3

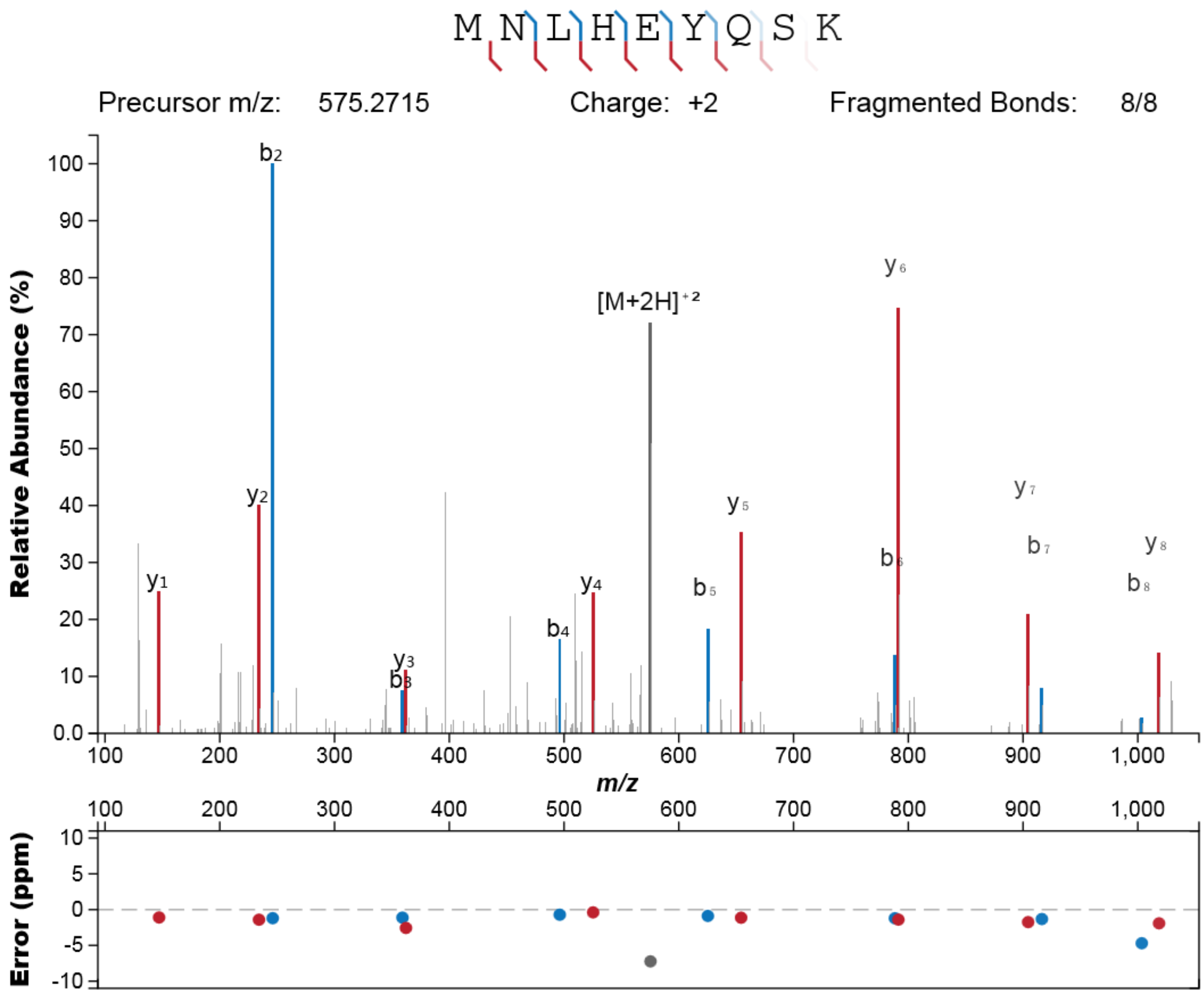

M N L H E Y Q S K
Precursor m/z: 575.2715
Charge: +2
Fragmented Bonds: 8/8
Relative Abundance (%)
m/z
Error (ppm)
[M+2H]+2
b2
y1
y2
y3
b3
b4
y4
b5
y5
y6
b6
y7
b7
y8
b8

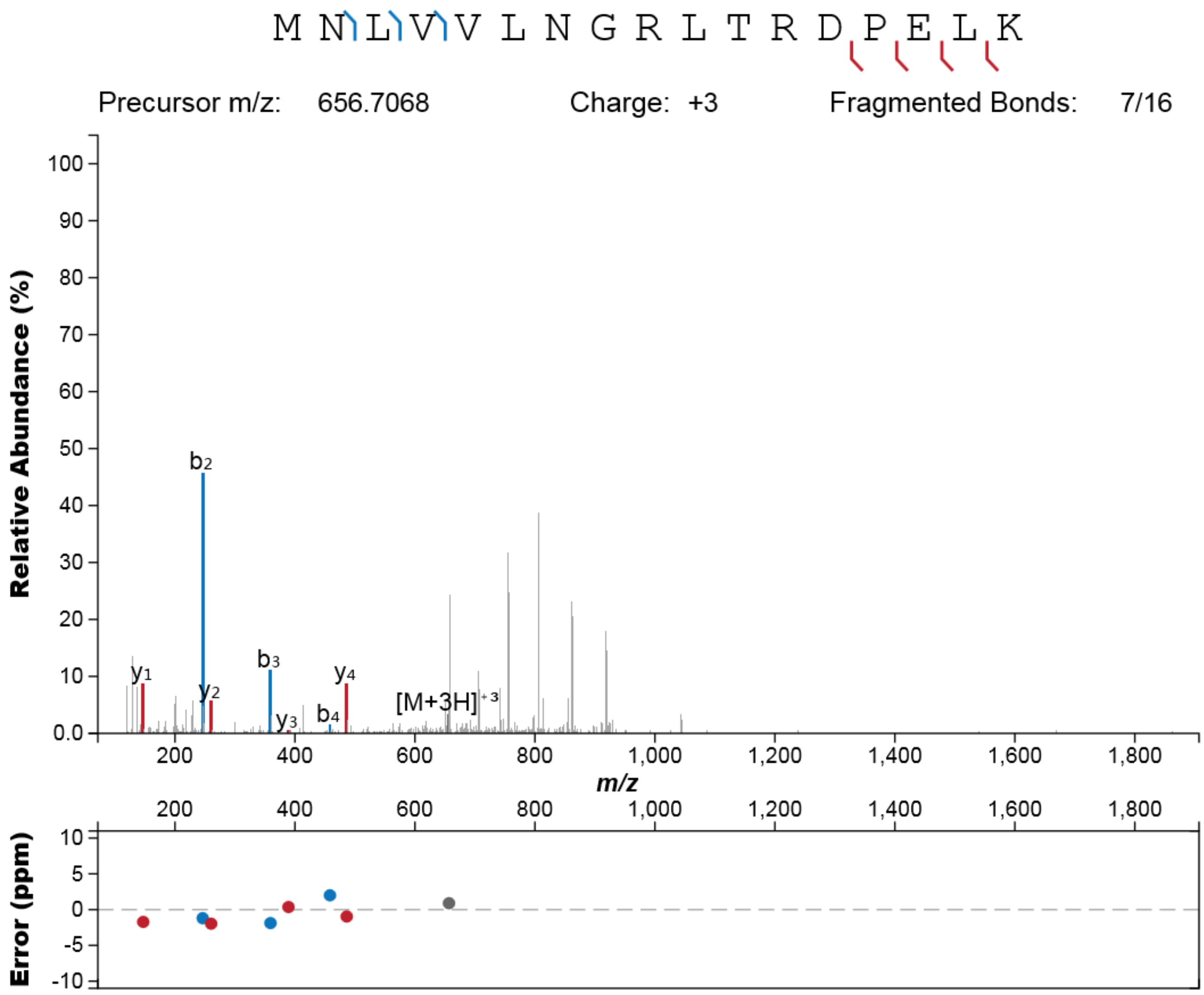
M N L V V L N G R L T R D P E L K
Precursor m/z: 656.7068
Charge: +3
Fragmented Bonds: 7/16
Relative Abundance (%)
b2
b3
b4
y1
y2
y3
y4
[M+3H]+3
m/z
Error (ppm)

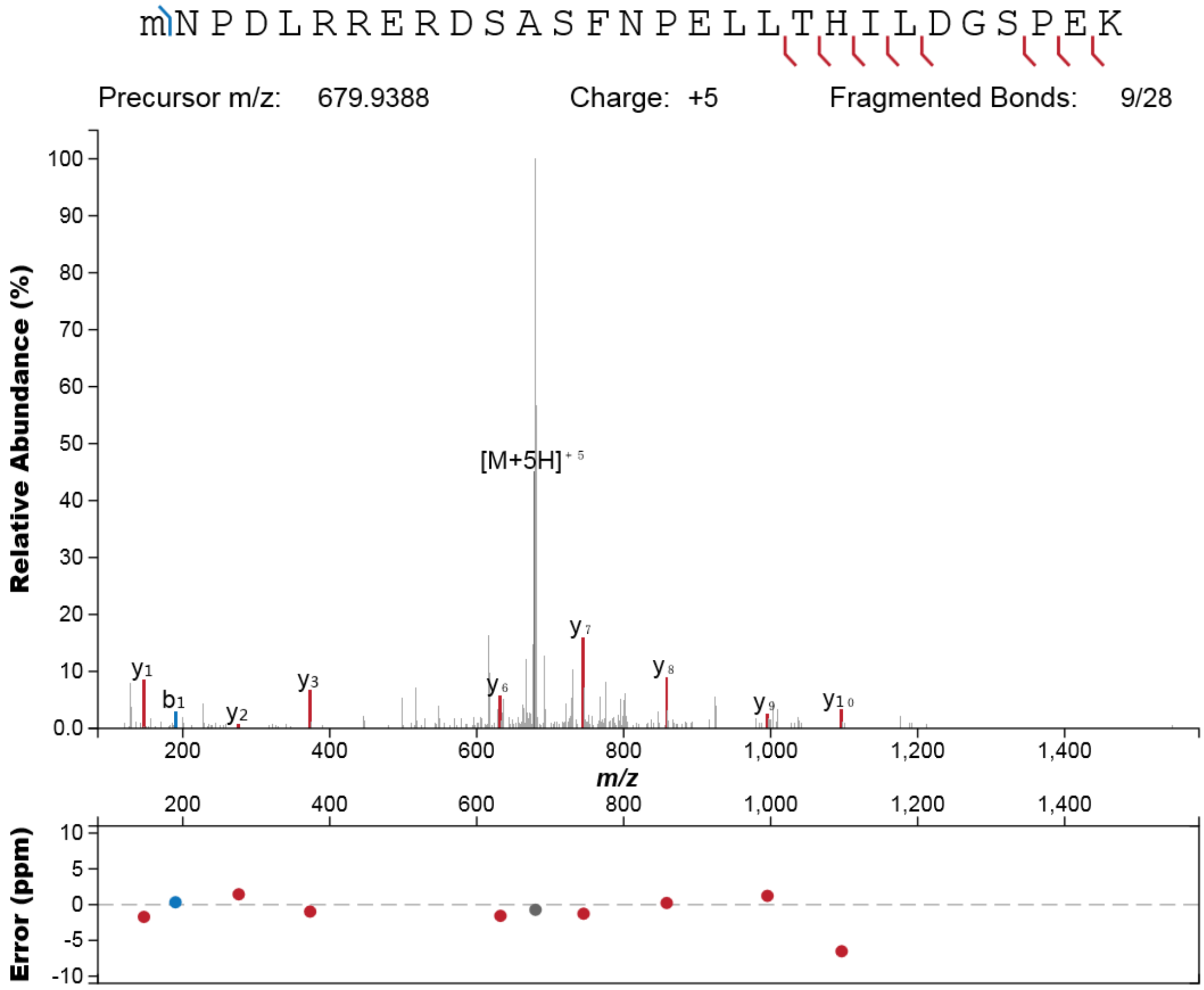

mNPDLRRERDSASFNPELLTHILDGSPEK
Precursor m/z: 679.9388
Charge: +5
Fragmented Bonds: 9/28
Relative Abundance (%)
[M+5H]+5
y1
b1
y2
y3
y6
y7
y8
y9
y10
m/z
Error (ppm)

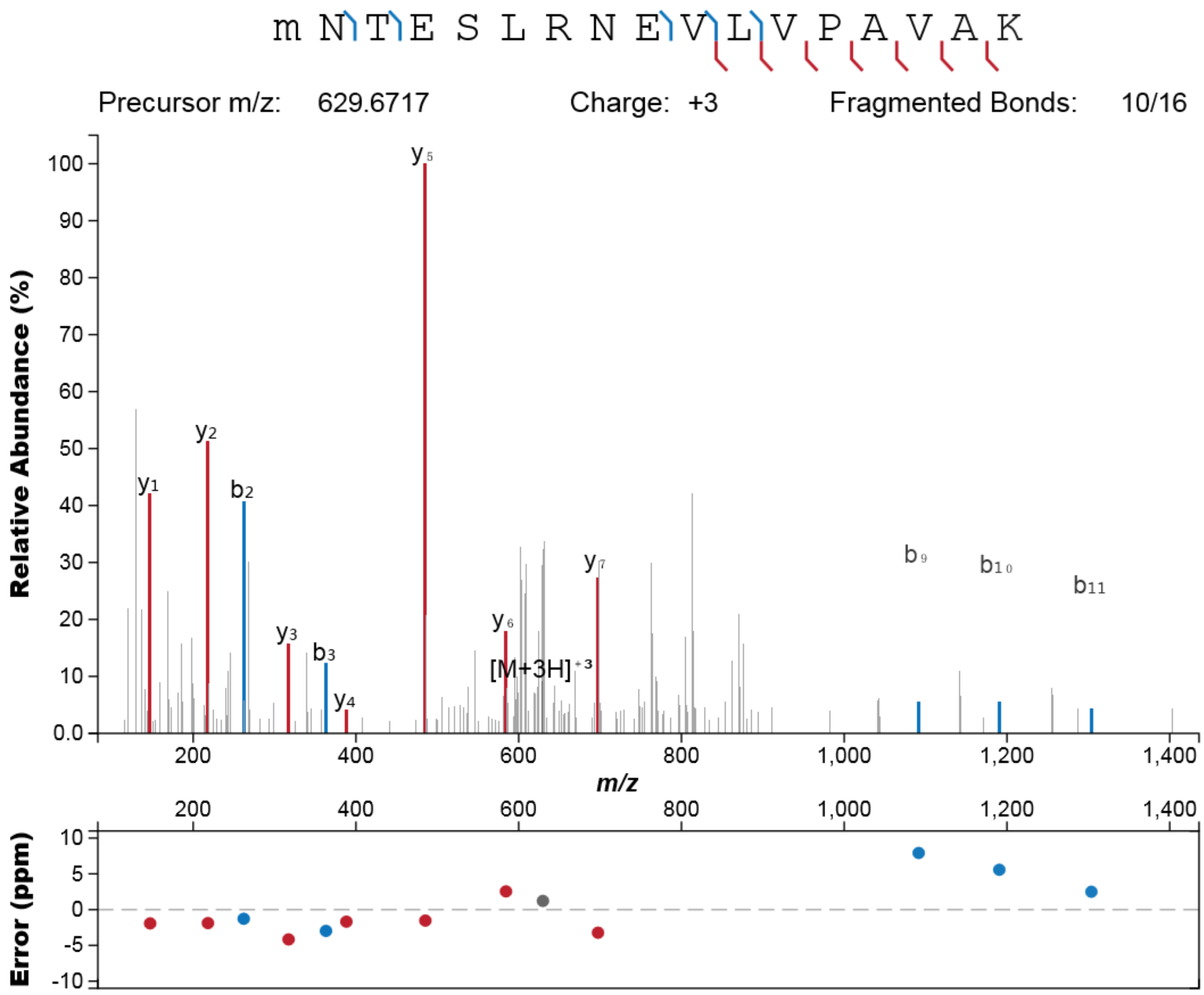
m N T E S L R N E V L V P A V A K
Precursor m/z: 629.6717
Charge: +3
Fragmented Bonds: 10/16
Relative Abundance (%)
m/z
Error (ppm)
y1
y2
b2
y3
b3
y4
y5
y6
[M+3H]+3
y7
b9
b10
b11

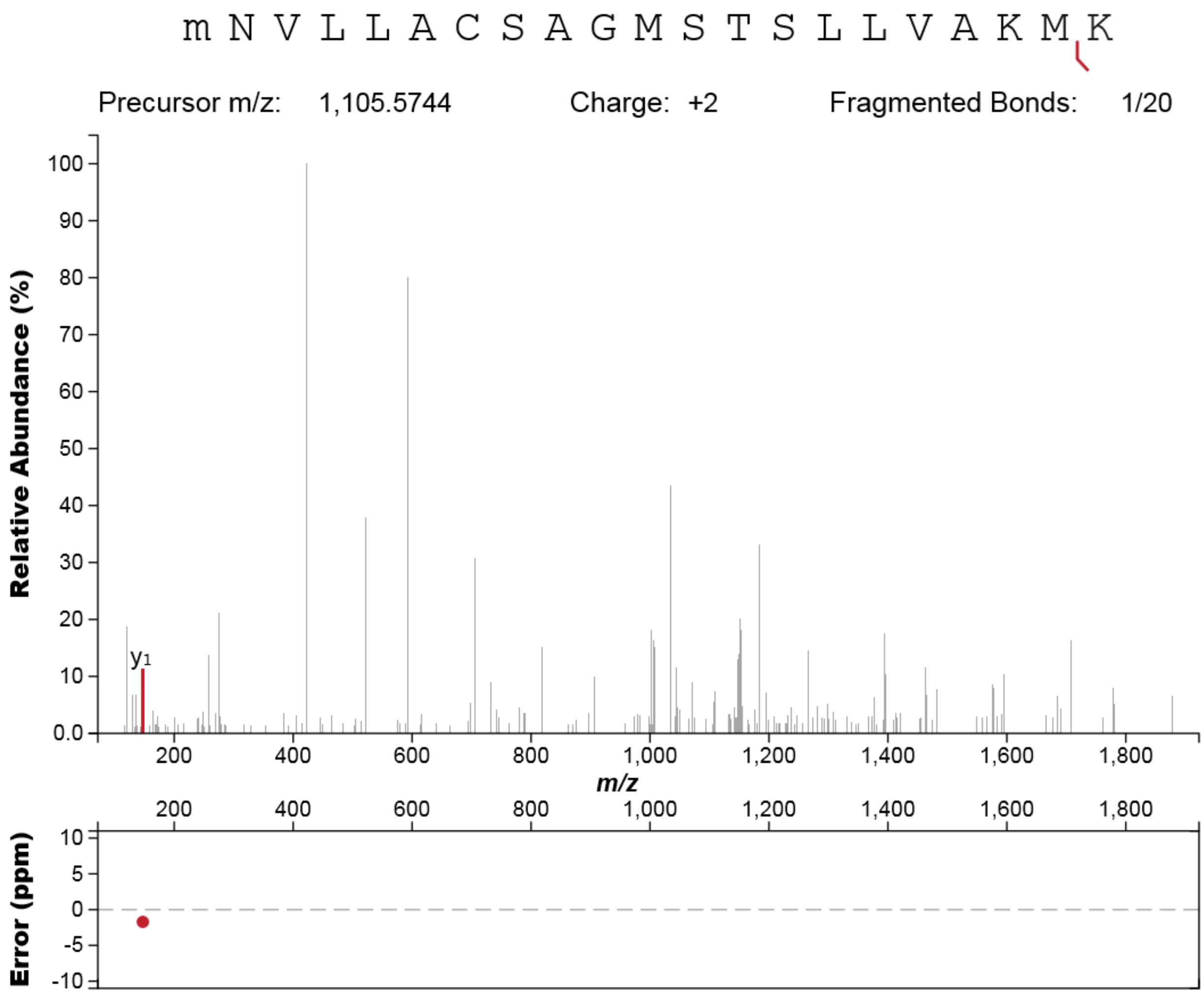
m N V L L A C S A G M S T S L L V A K M K
Precursor m/z: 1,105.5744
Charge: +2
Fragmented Bonds: 1/20
Relative Abundance (%)
y1
m/z
Error (ppm)

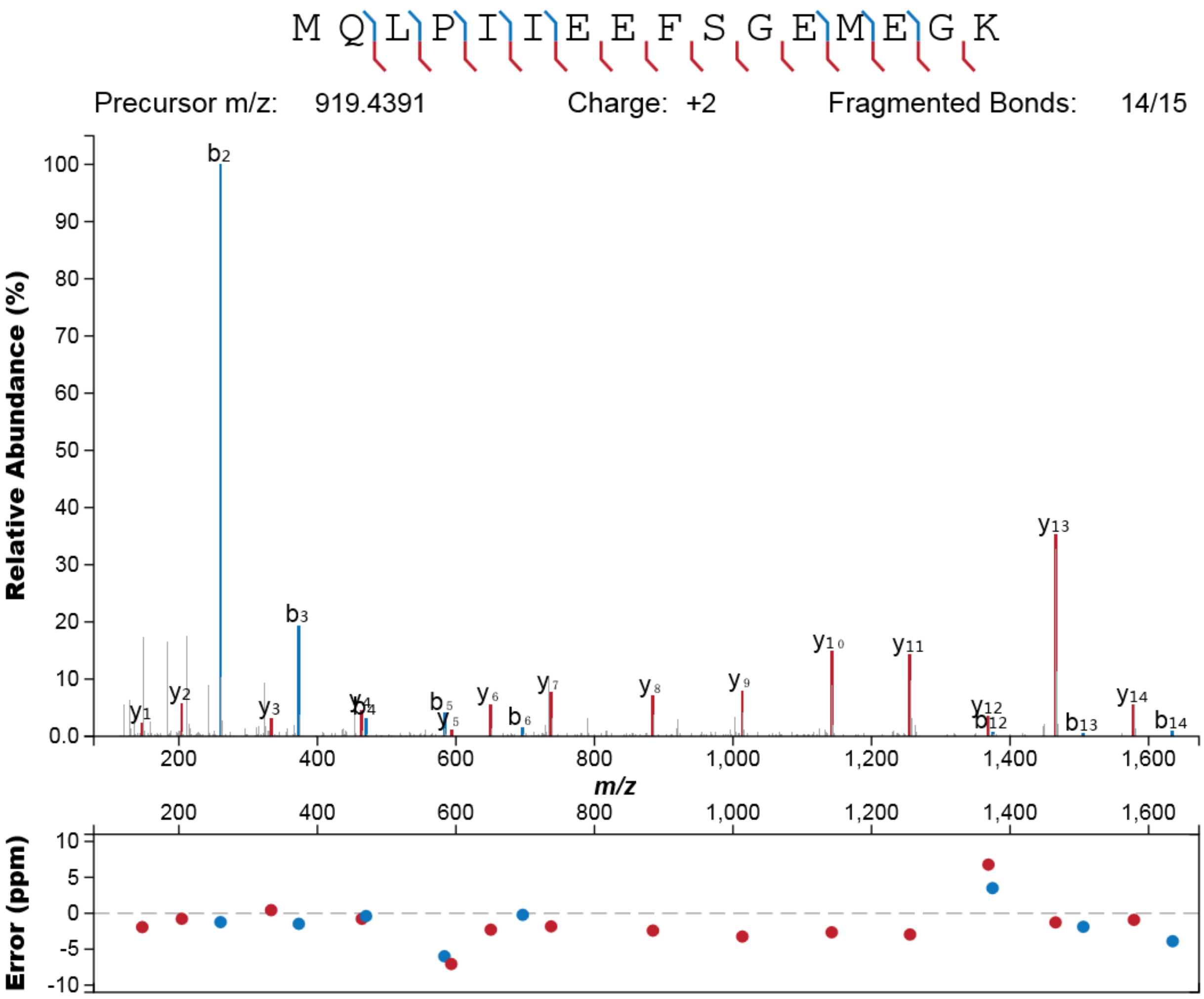
M Q L P I I E E F S G E M E G K
Precursor m/z: 919.4391
Charge: +2
Fragmented Bonds: 14/15
Relative Abundance (%)
m/z
Error (ppm)
b2
b3
y1
y2
y3
y4
b4
b5
y5
y6
b6
y7
y8
y9
y10
y11
y12
b12
y13
b13
y14
b14

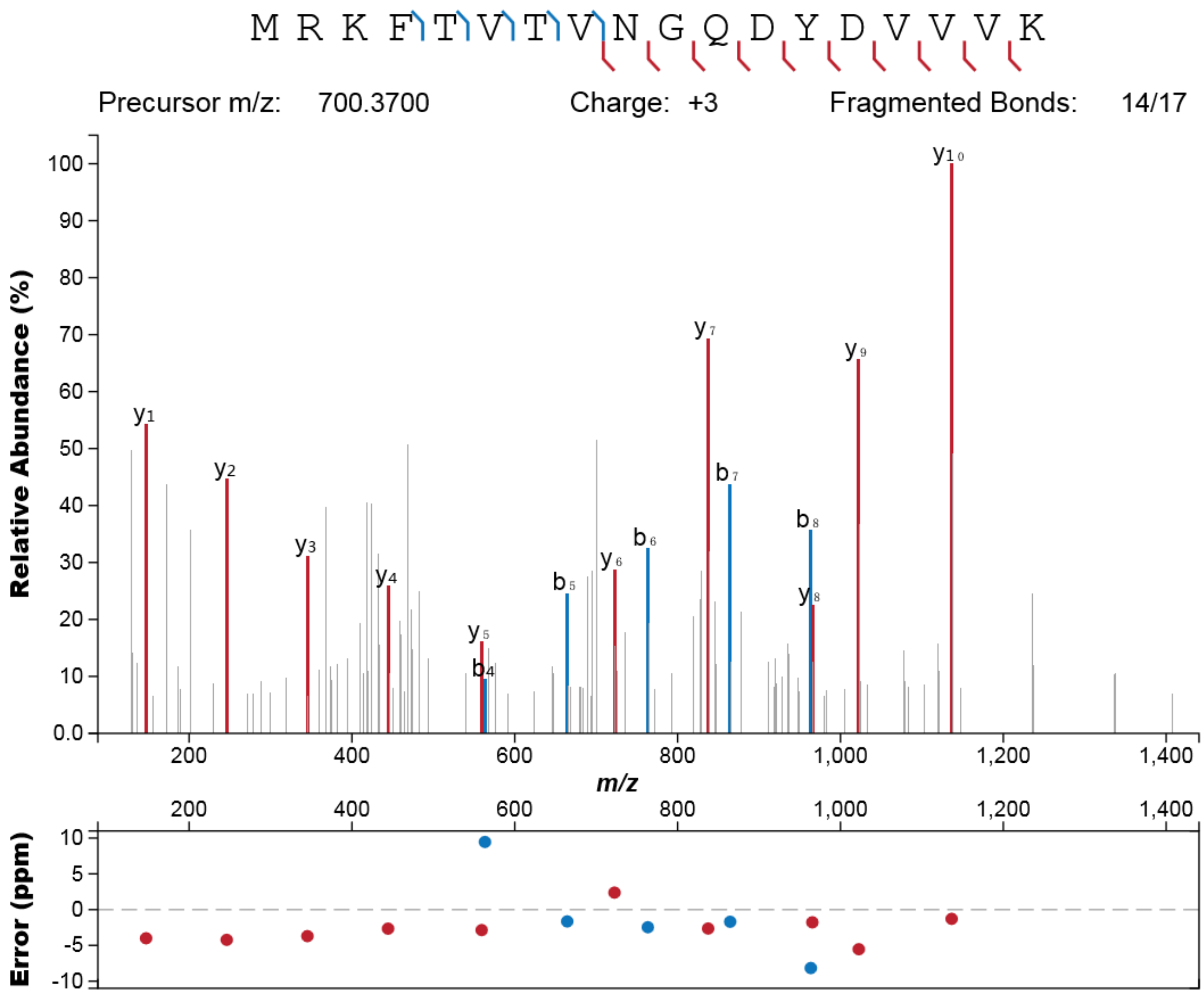
M R K F T V T V N G Q D Y D V V V K
Precursor m/z: 700.3700
Charge: +3
Fragmented Bonds: 14/17
Relative Abundance (%)
m/z
Error (ppm)
y1 y2 y3 y4 y5 b4 b5 y6 b6 y7 b7 b8 y8 y9 y10

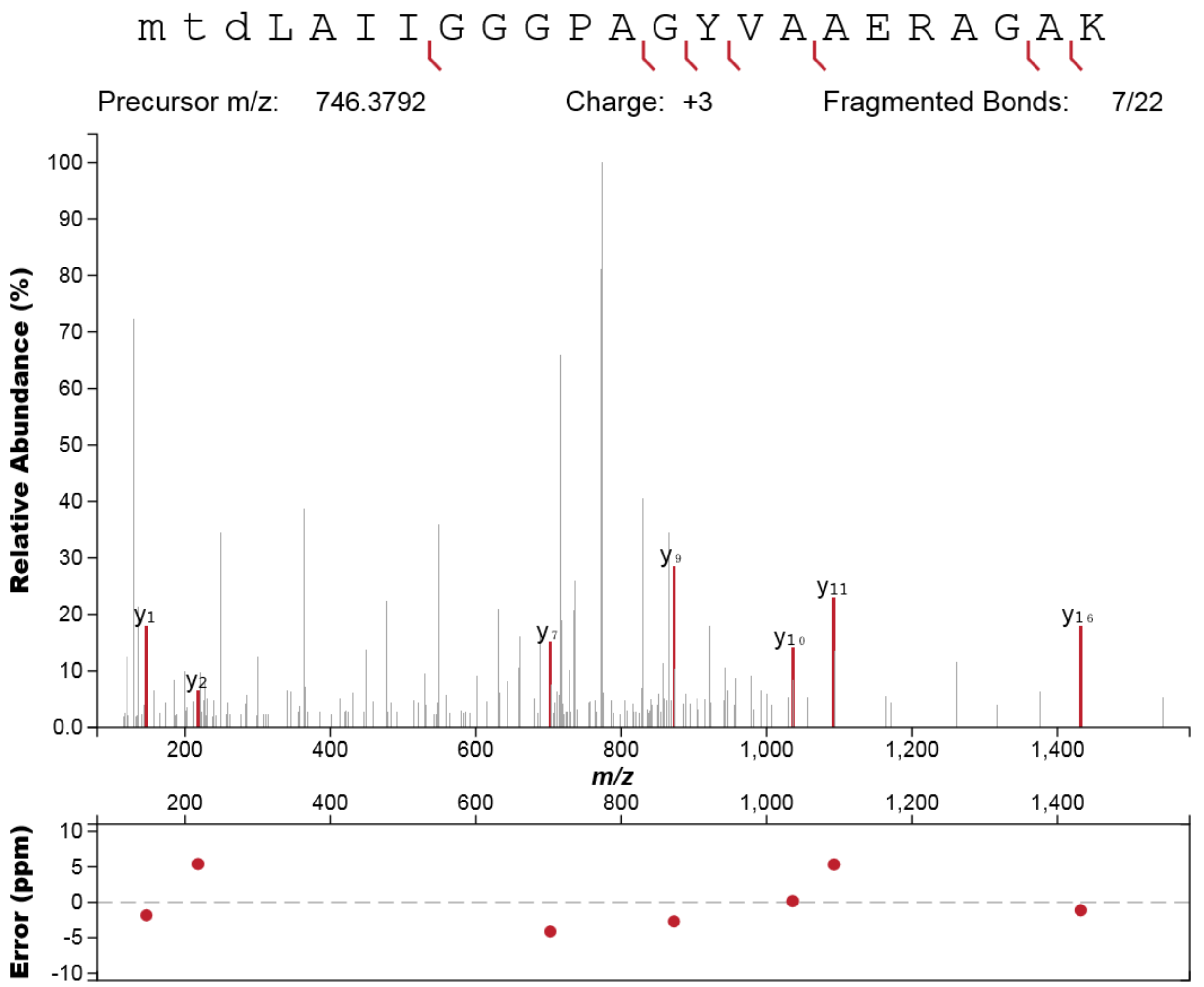

m t d L A I I G G G P A G Y V A A E R A G A K
Precursor m/z: 746.3792
Charge: +3
Fragmented Bonds: 7/22
Relative Abundance (%)
m/z
Error (ppm)
y1
y2
y7
y9
y10
y11
y16

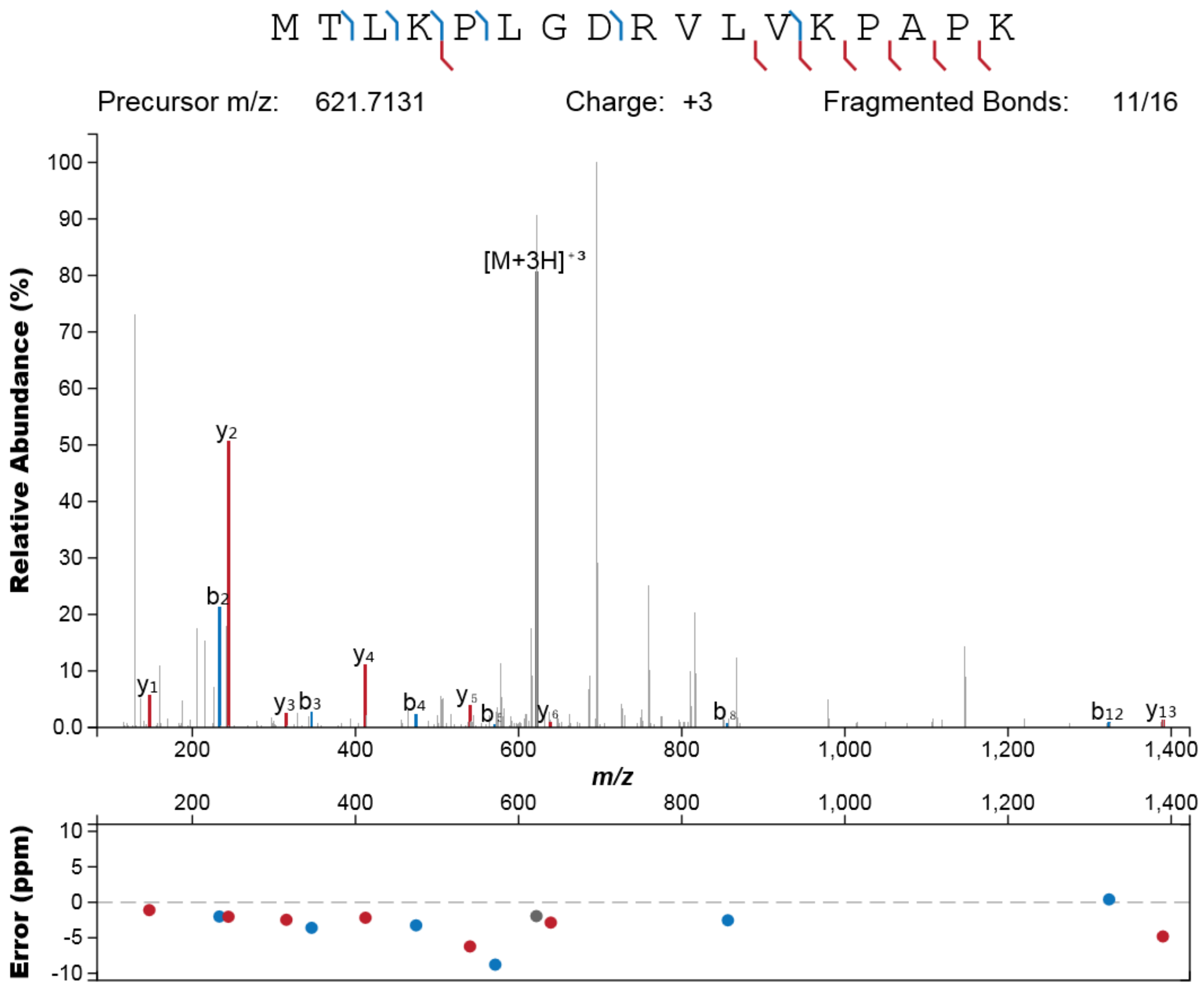

M T L K P L G D R V L V K P A P K
Precursor m/z: 621.7131
Charge: +3
Fragmented Bonds: 11/16
Relative Abundance (%)
[M+3H]+3
m/z
Error (ppm)

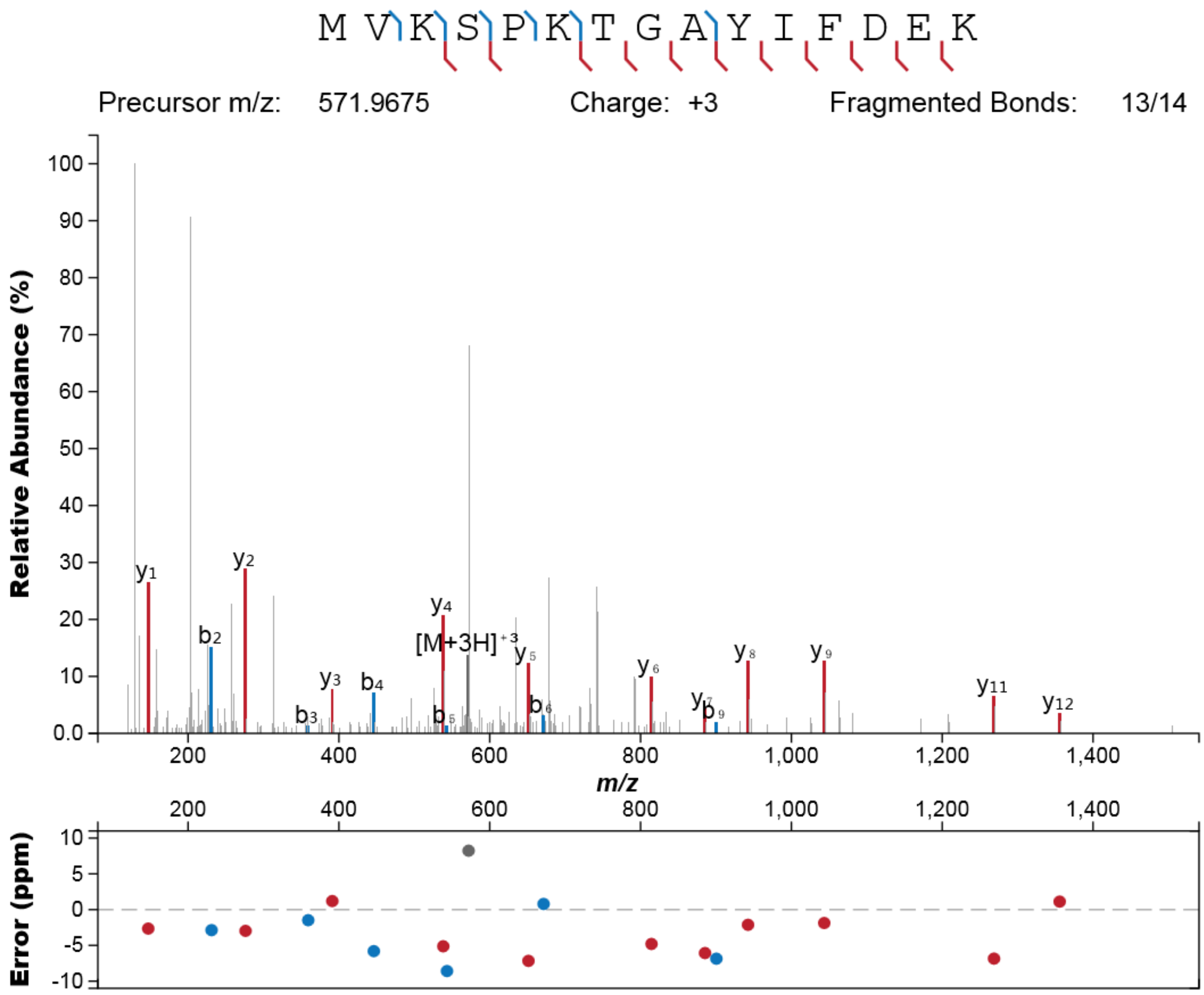

M V K S P K T G A Y I F D E K
Precursor m/z: 571.9675
Charge: +3
Fragmented Bonds: 13/14
Relative Abundance (%)
m/z
Error (ppm)
[M+3H]+3

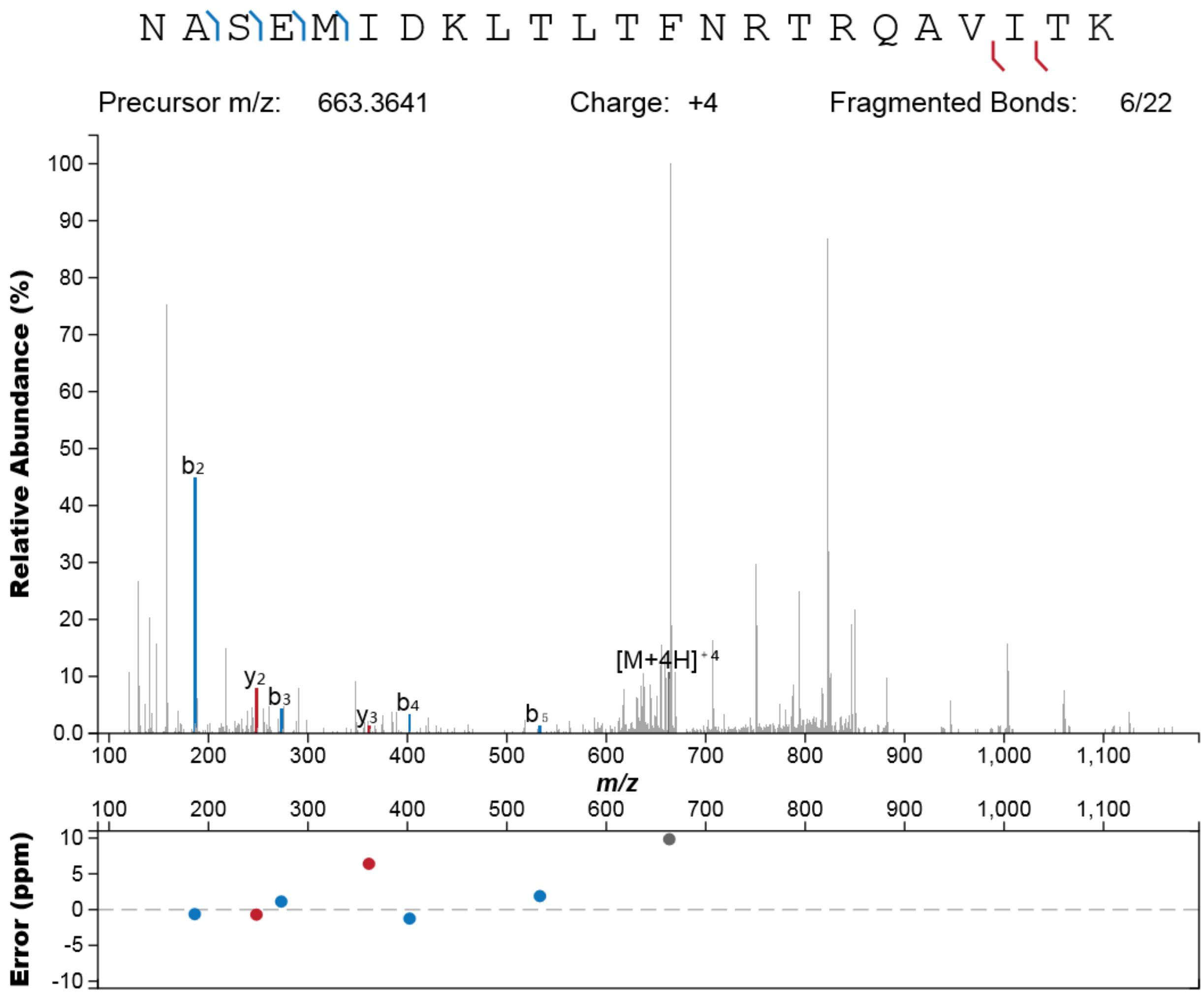

N A S E M I D K L T L T F N R T R Q A V I T K
Precursor m/z: 663.3641
Charge: +4
Fragmented Bonds: 6/22
Relative Abundance (%)
b2
y2
b3
y3
b4
b5
[M+4H]+4
m/z
Error (ppm)

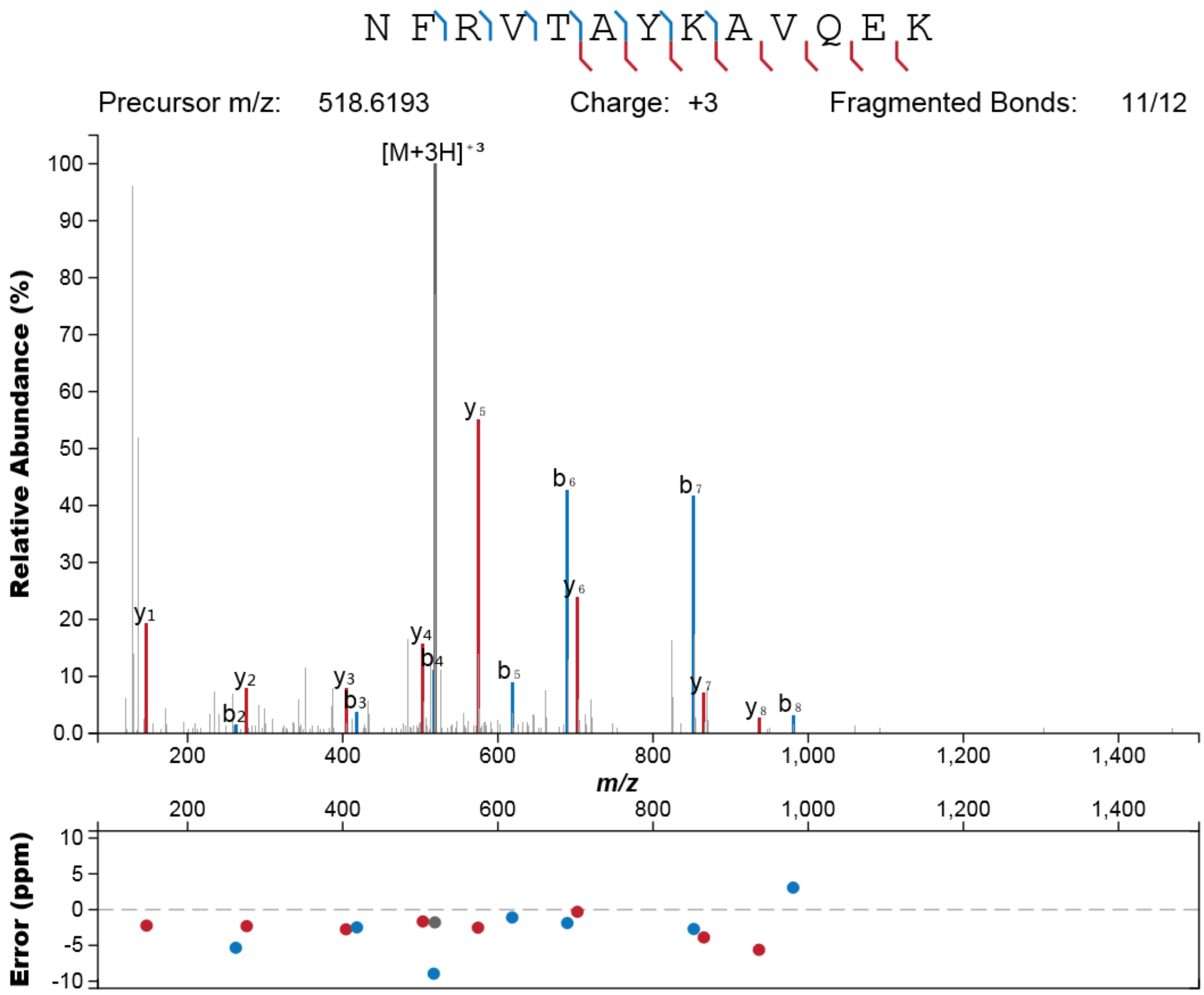

N F R V T A Y K A V Q E K
Precursor m/z: 518.6193
Charge: +3
Fragmented Bonds: 11/12
[M+3H]+3
y1
y2
b2
y3
b3
y4
b4
y5
b5
b6
y6
b7
y7
y8
b8
Relative Abundance (%)
m/z
Error (ppm)

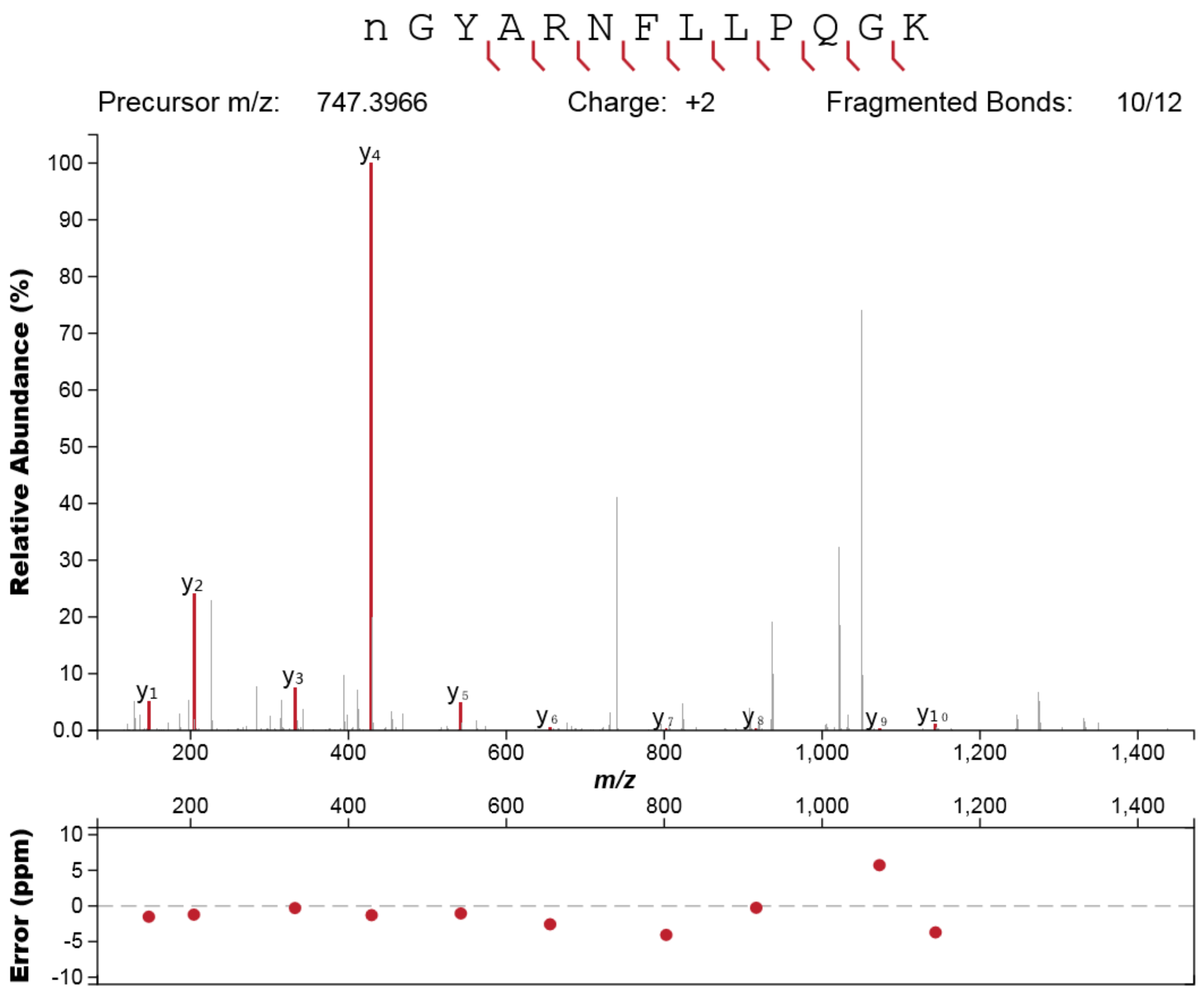

n G Y A R N F L L P Q G K
Precursor m/z: 747.3966
Charge: +2
Fragmented Bonds: 10/12
Relative Abundance (%)
m/z
Error (ppm)
y1
y2
y3
y4
y5
y6
y7
y8
y9
y10

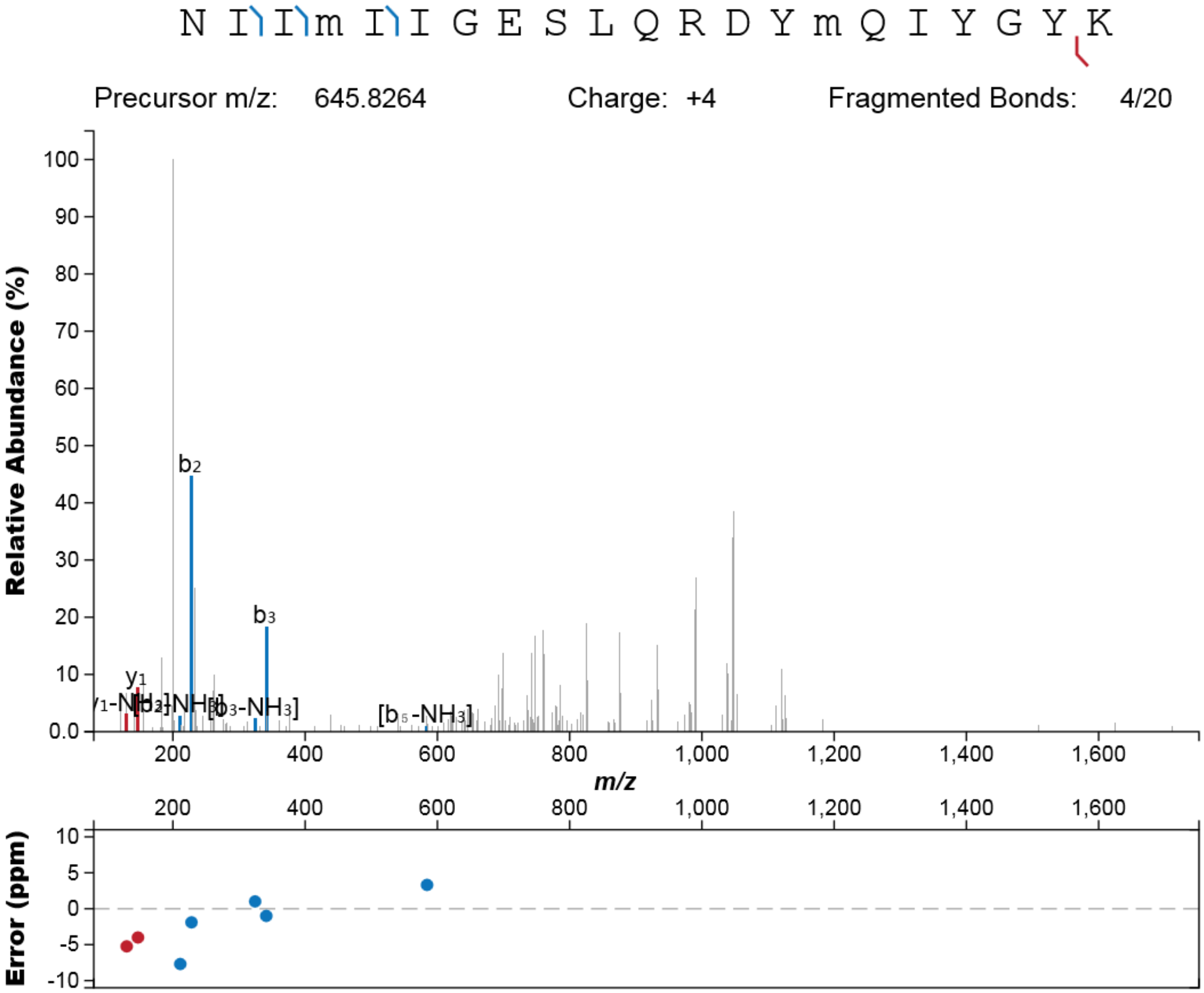
N I I m I I G E S L Q R D Y m Q I Y G Y K
Precursor m/z: 645.8264
Charge: +4
Fragmented Bonds: 4/20
Relative Abundance (%)
b2
b3
y1
[b5-NH3]
m/z
Error (ppm)

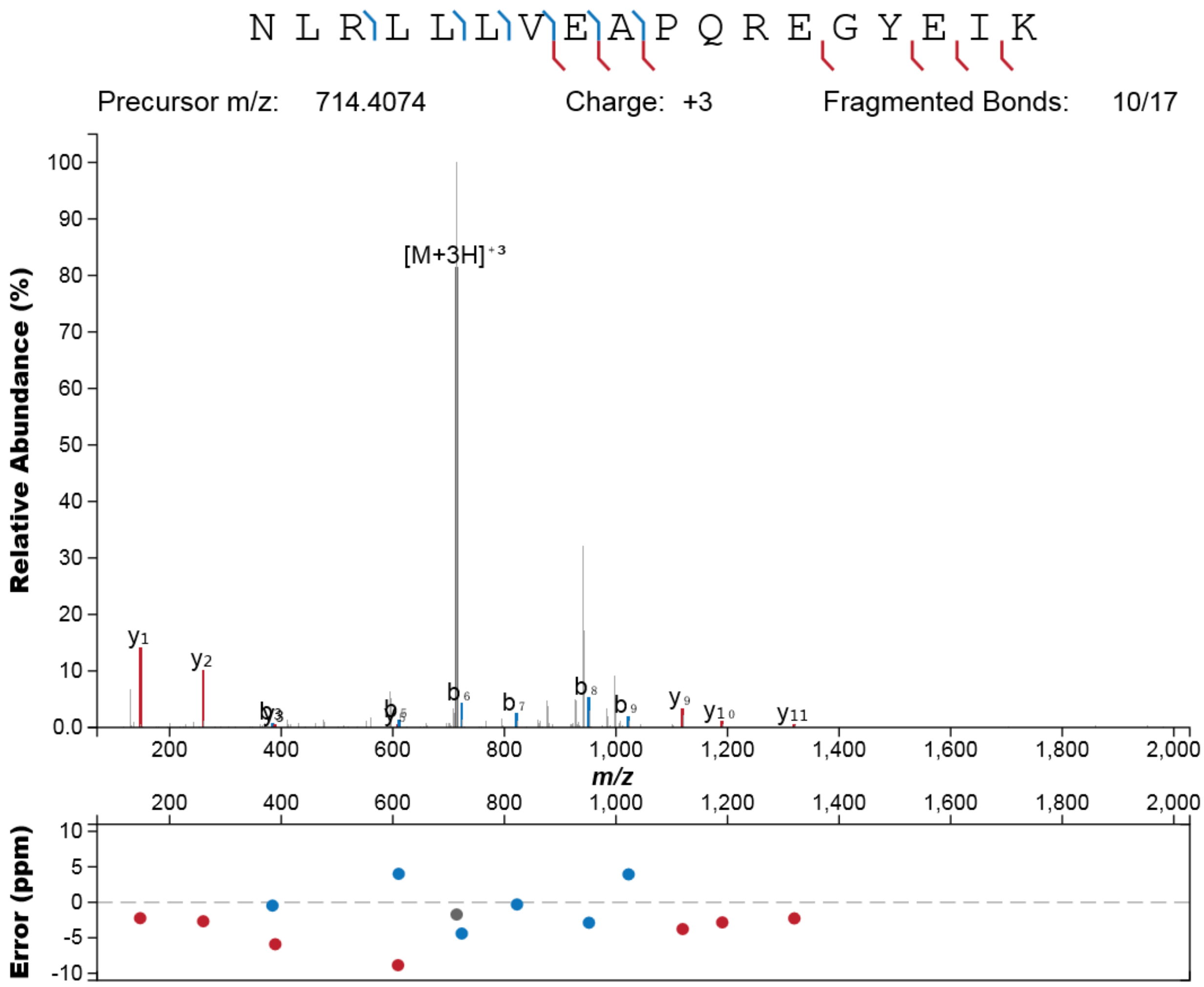
N L R L L L V E A P Q R E G Y E I K
Precursor m/z: 714.4074
Charge: +3
Fragmented Bonds: 10/17
Relative Abundance (%)
[M+3H]+3
m/z
Error (ppm)

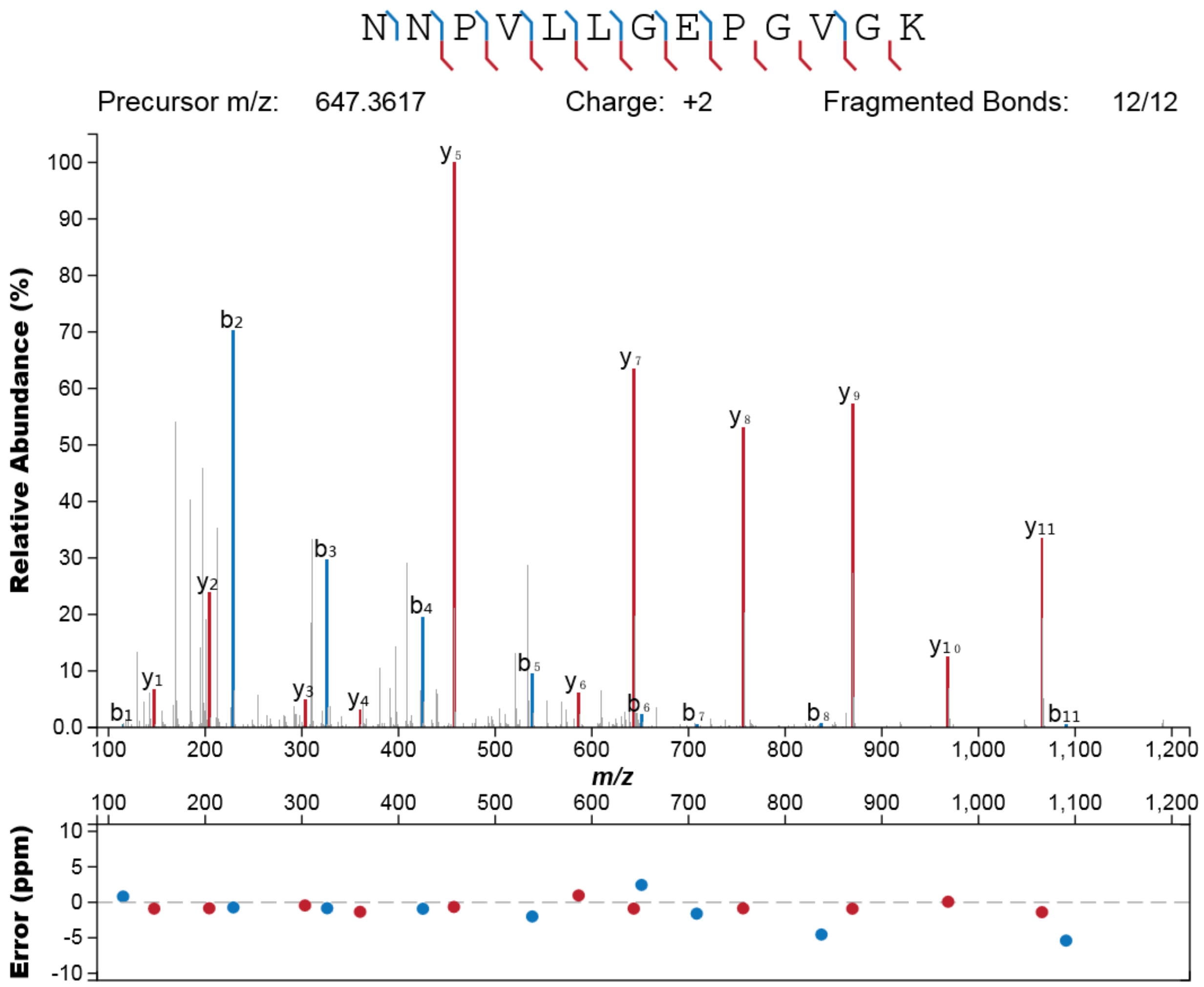

N N P V L L G E P G V G K
Precursor m/z: 647.3617
Charge: +2
Fragmented Bonds: 12/12
Relative Abundance (%)
m/z
Error (ppm)
b1
b2
b3
b4
b5
b6
b7
b8
b11
y1
y2
y3
y4
y5
y6
y7
y8
y9
y10
y11

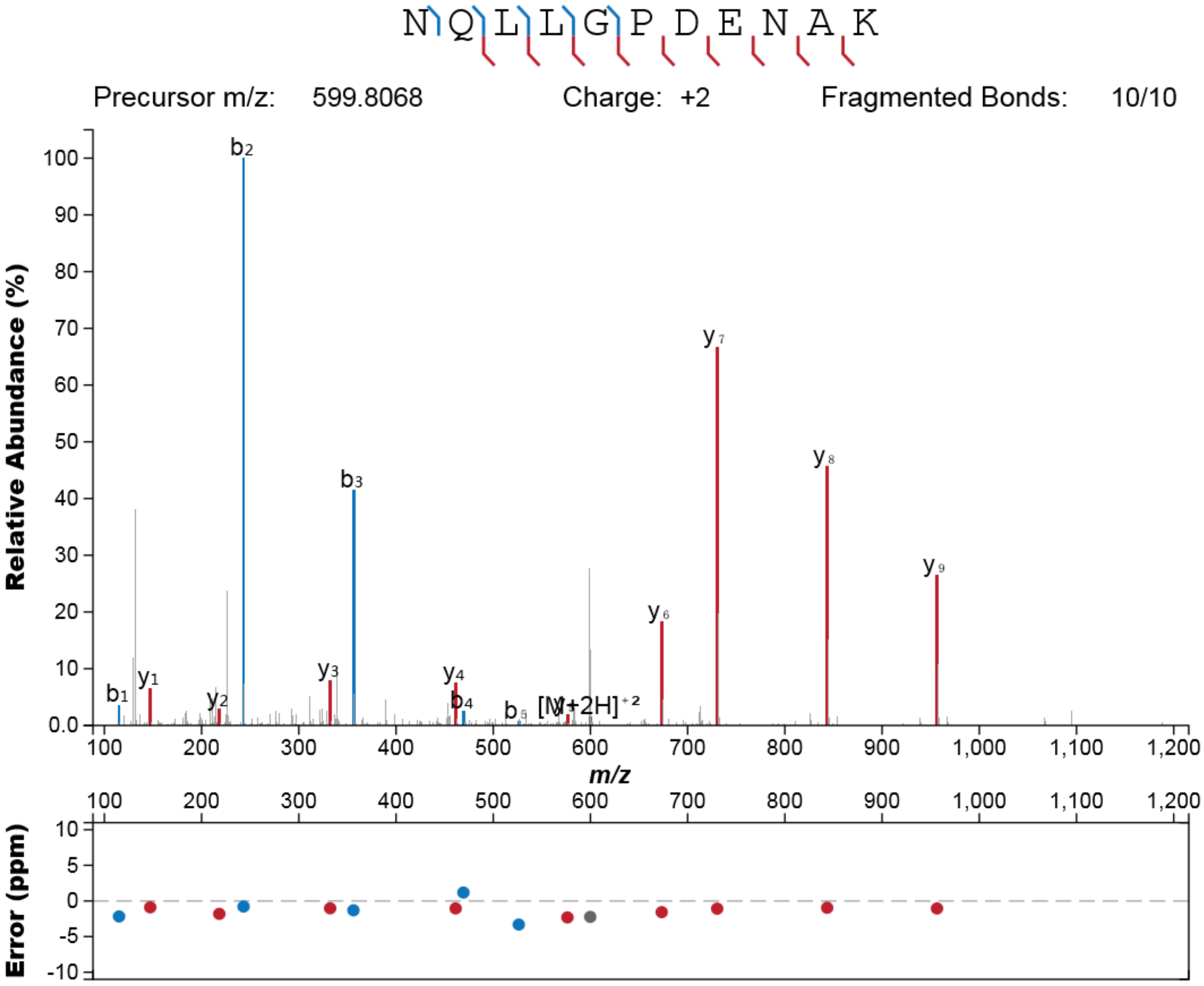
N Q L L G P D E N A K
Precursor m/z: 599.8068
Charge: +2
Fragmented Bonds: 10/10
Relative Abundance (%)
m/z
Error (ppm)
b1 y1 y2 b2 y3 b3 y4 b4 b5 [M+2H]+2 y6 y7 y8 y9

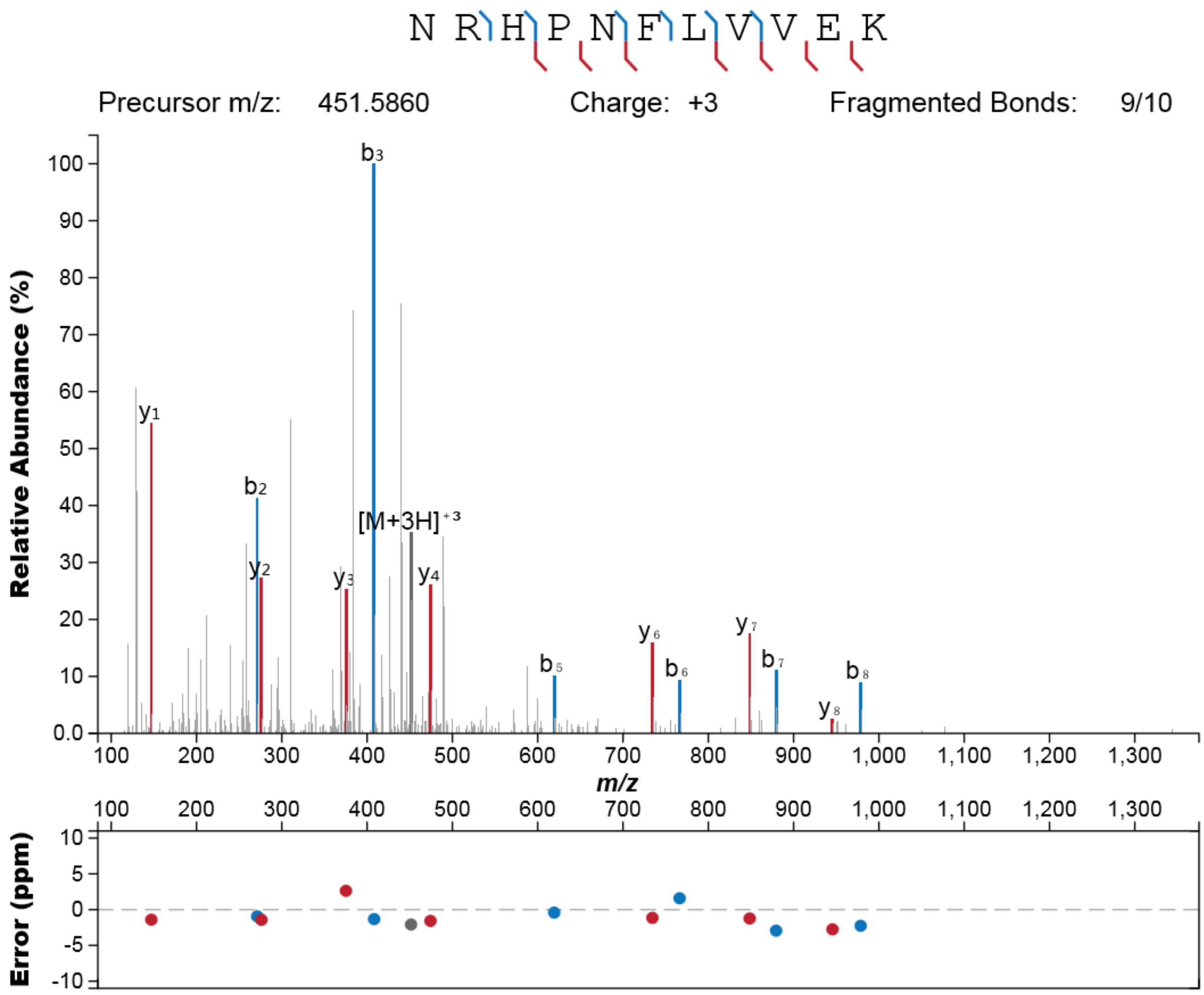
N R H P N F L V V E K
Precursor m/z: 451.5860
Charge: +3
Fragmented Bonds: 9/10
Relative Abundance (%)
y1
b2
y2
y3
b3
[M+3H]+3
y4
b5
y6
b6
y7
b7
y8
b8
m/z
Error (ppm)

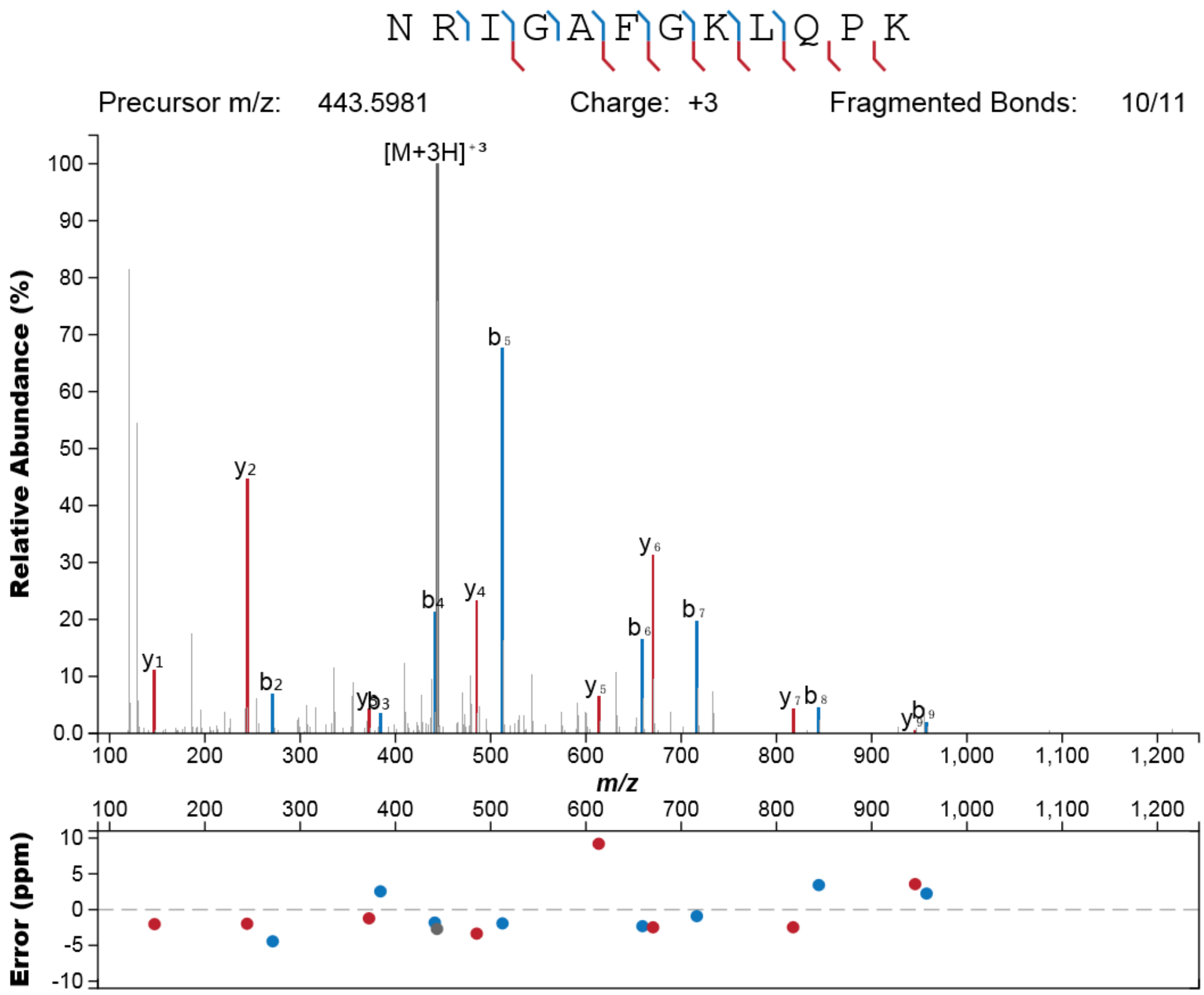

N R I G A F G K L Q P K
Precursor m/z: 443.5981
Charge: +3
Fragmented Bonds: 10/11
[M+3H]+3
Relative Abundance (%)
m/z
Error (ppm)
y1
y2
b2
b3
b4
y4
b5
y5
b6
y6
b7
y7
b8
b9

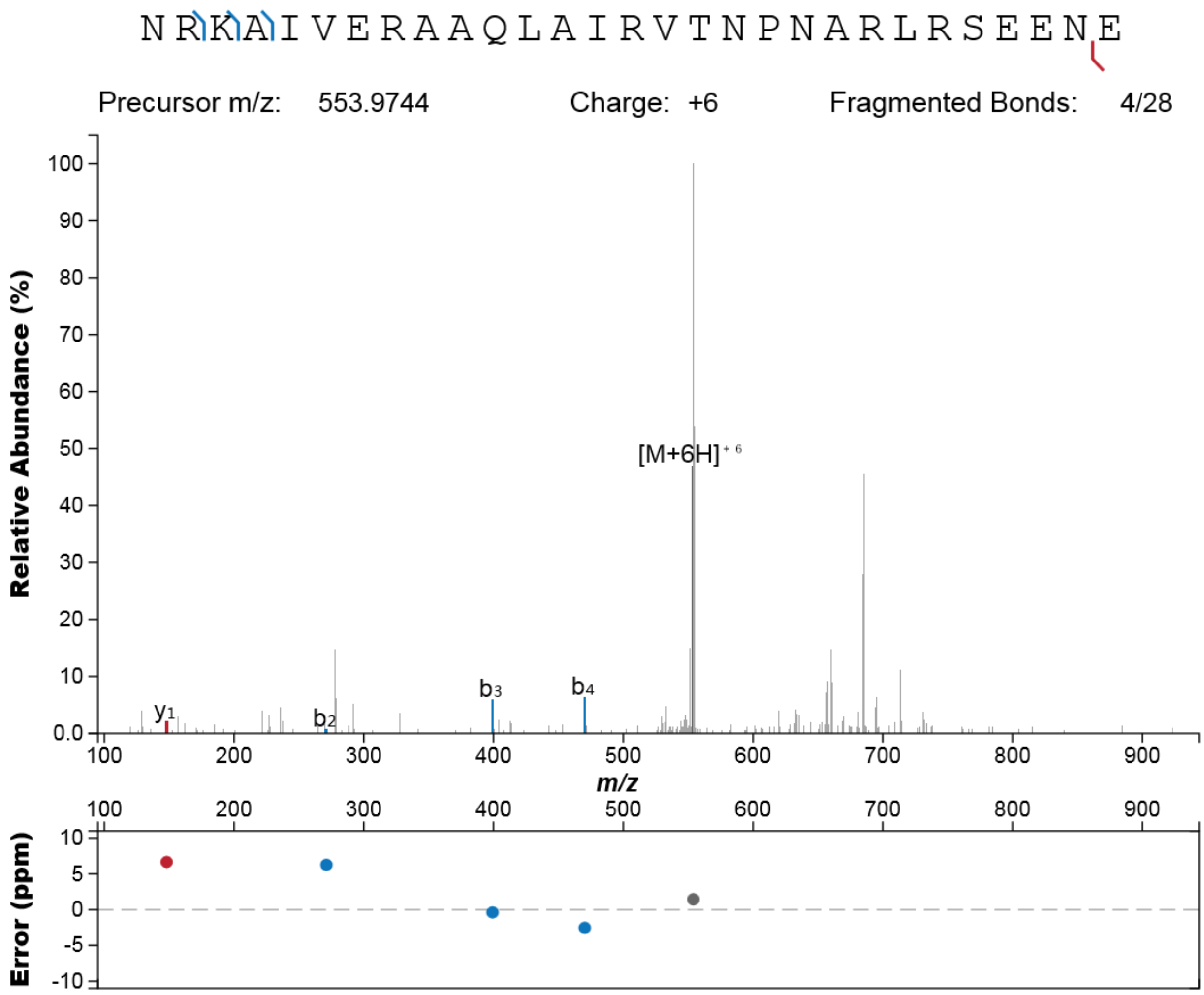

N R K A I V E R A A Q L A I R V T N P N A R L R S E E N E
Precursor m/z: 553.9744
Charge: +6
Fragmented Bonds: 4/28
Relative Abundance (%)
[M+6H]+6
y1
b2
b3
b4
m/z
Error (ppm)

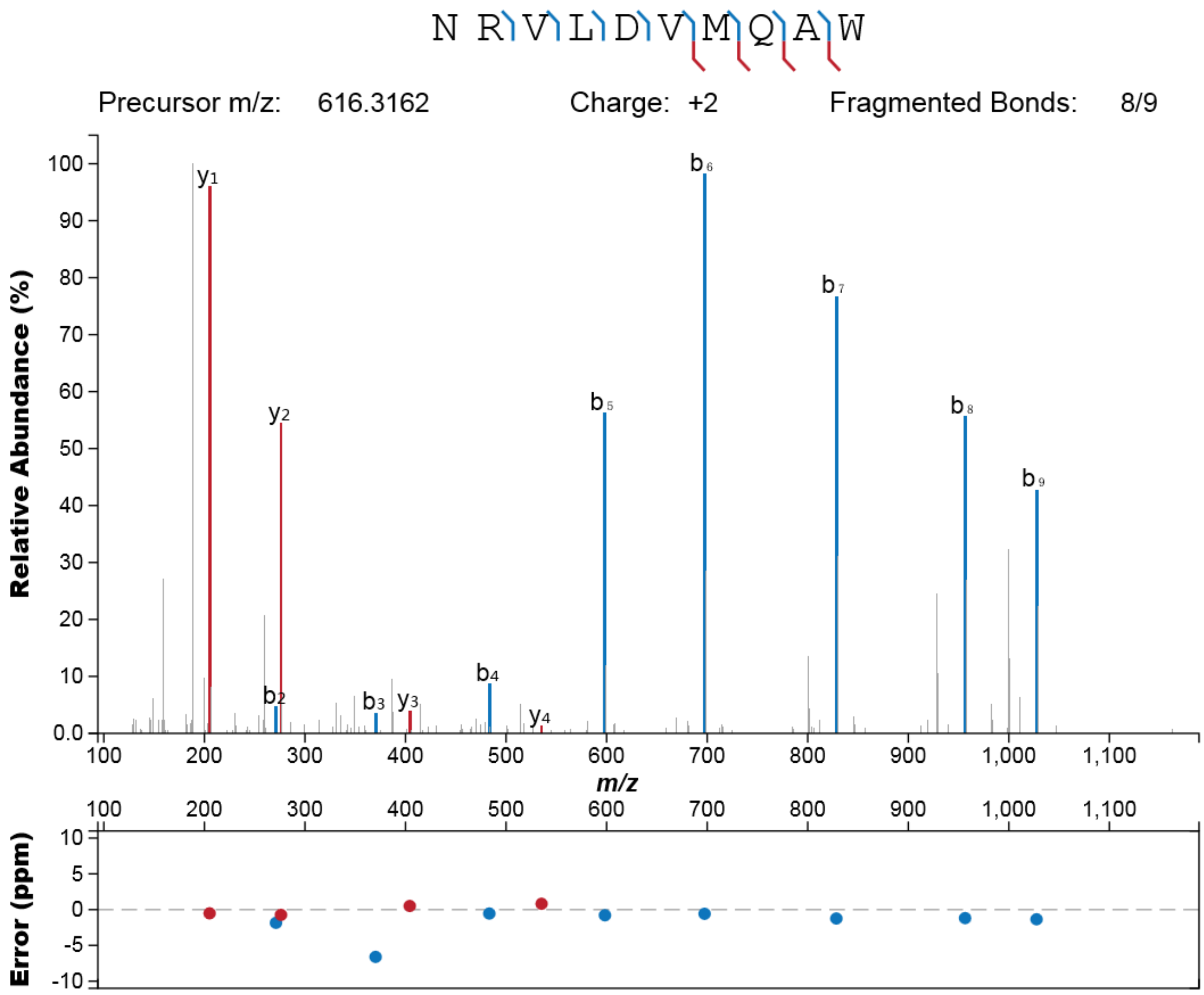

N R V L D V M Q A W
Precursor m/z: 616.3162
Charge: +2
Fragmented Bonds: 8/9
Relative Abundance (%)
y1
y2
b2
b3
y3
b4
y4
b5
b6
b7
b8
b9
m/z
Error (ppm)

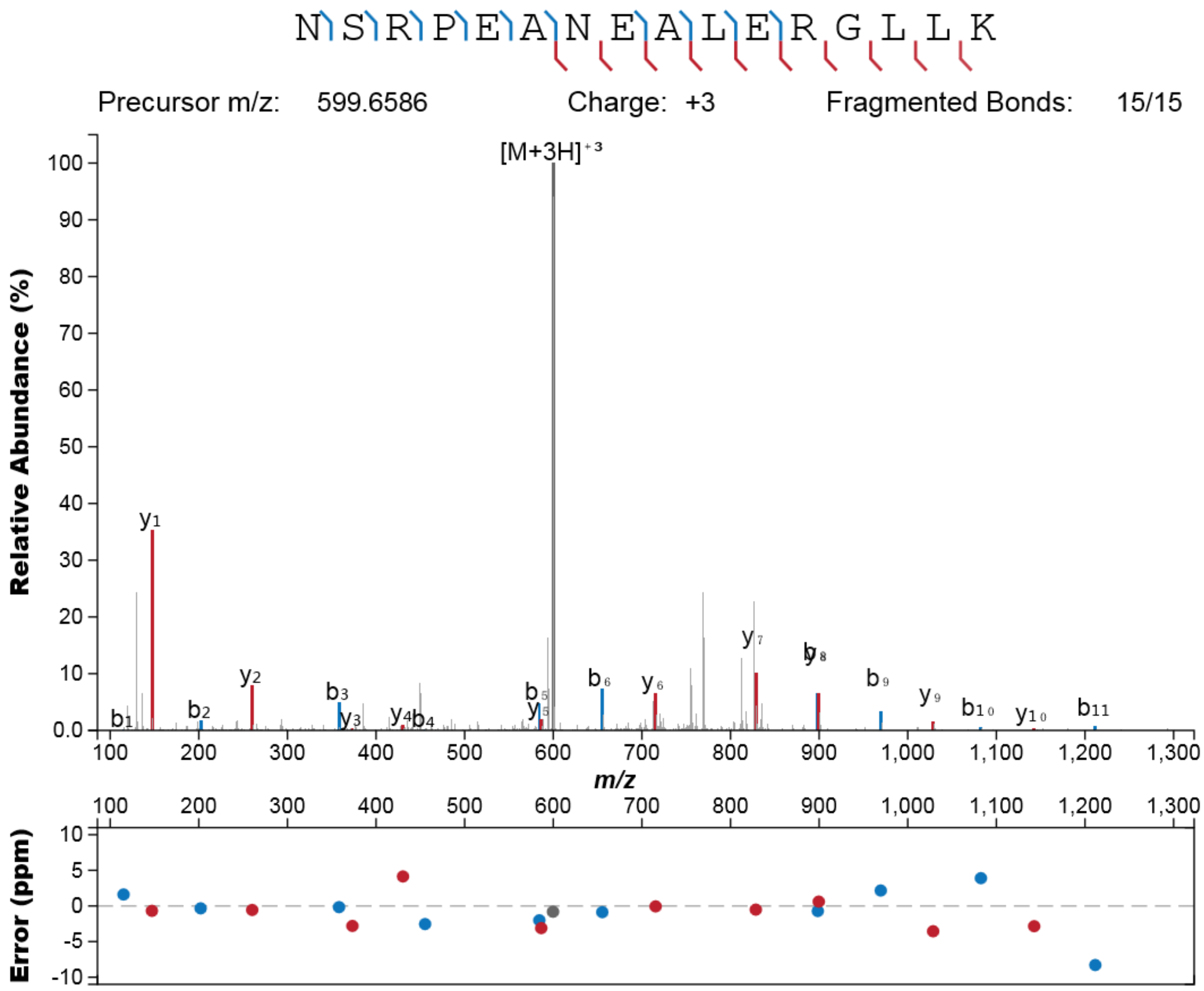

N S R P E A N E A L E R G L L K
Precursor m/z: 599.6586
Charge: +3
Fragmented Bonds: 15/15
[M+3H]+3
Relative Abundance (%)
m/z
Error (ppm)
y1
y2
y7
y6
b1
b2
b3
y3
y4
b4
b5
y5
b6
b8
y8
b9
y9
b10
y10
b11

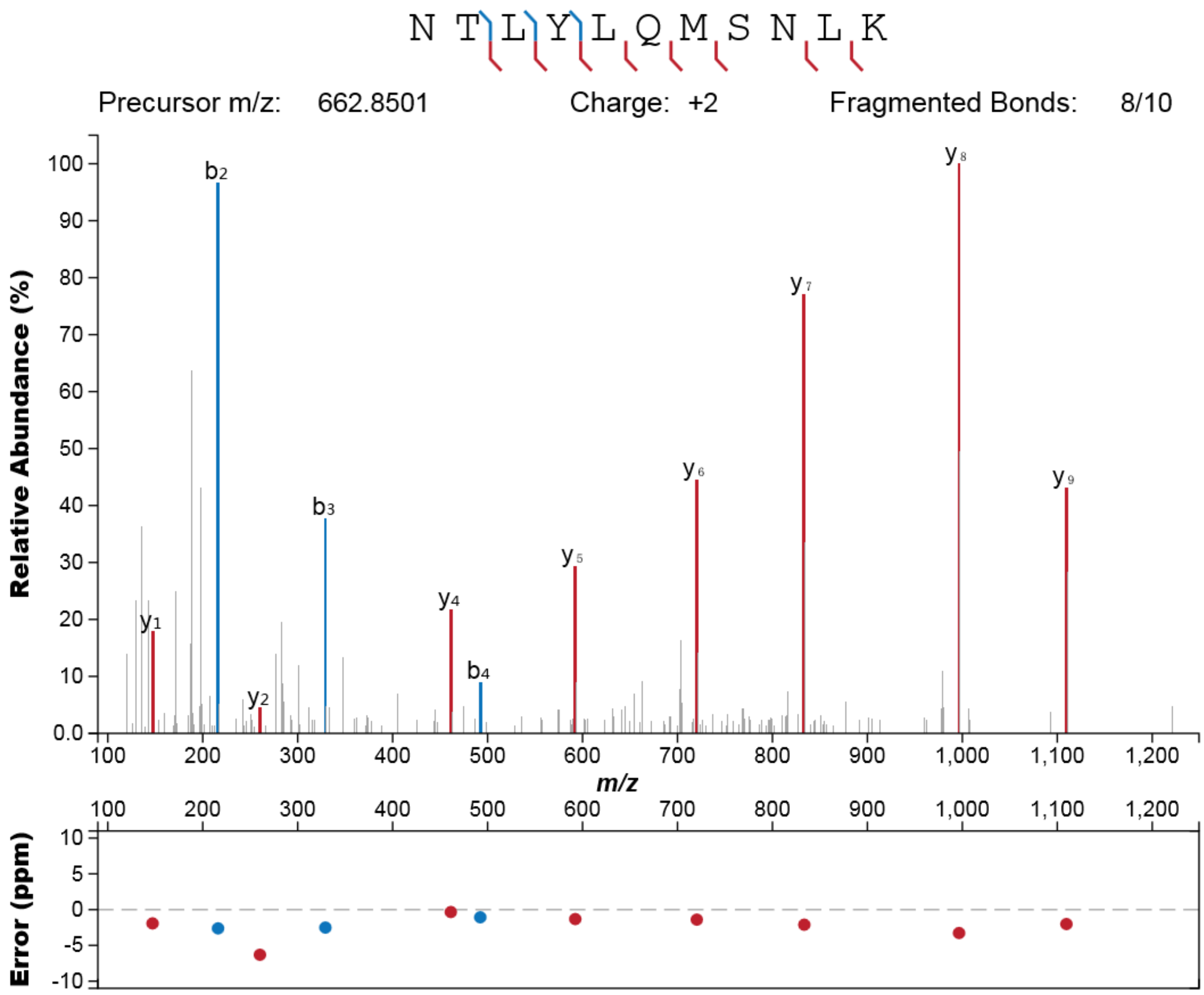

N T L Y L Q M S N L K
Precursor m/z: 662.8501
Charge: +2
Fragmented Bonds: 8/10
Relative Abundance (%)
m/z
Error (ppm)
b2
b3
b4
y1
y2
y4
y5
y6
y7
y8
y9

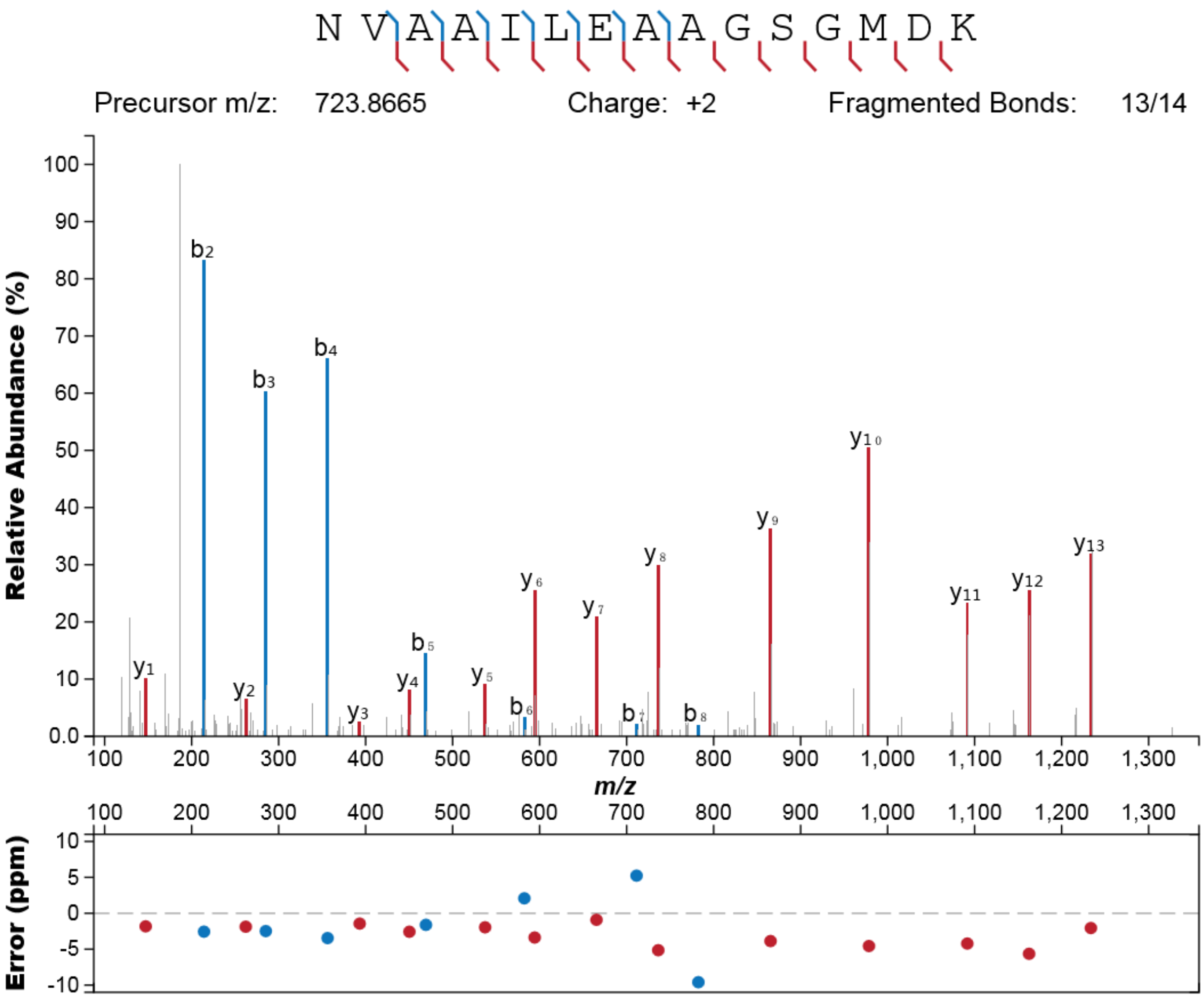
N V A A I L E A A G S G M D K
Precursor m/z: 723.8665
Charge: +2
Fragmented Bonds: 13/14
Relative Abundance (%)
m/z
Error (ppm)
b2 b3 b4 b5 b6 b7 b8
y1 y2 y3 y4 y5 y6 y7 y8 y9 y10 y11 y12 y13

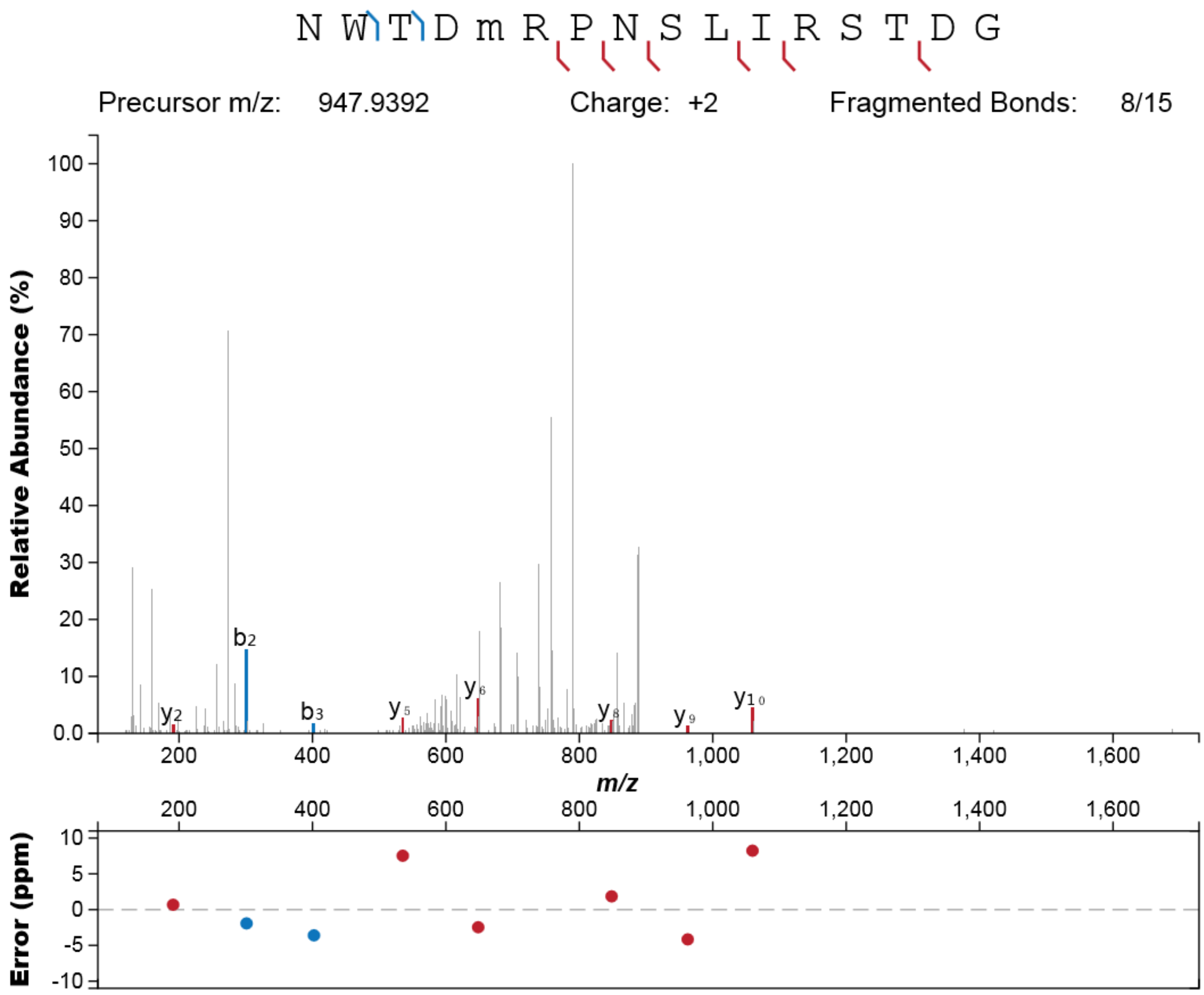
N W T D m R P N S L I R S T D G
Precursor m/z: 947.9392
Charge: +2
Fragmented Bonds: 8/15
Relative Abundance (%)
m/z
Error (ppm)
b2
b3
y2
y5
y6
y8
y9
y10

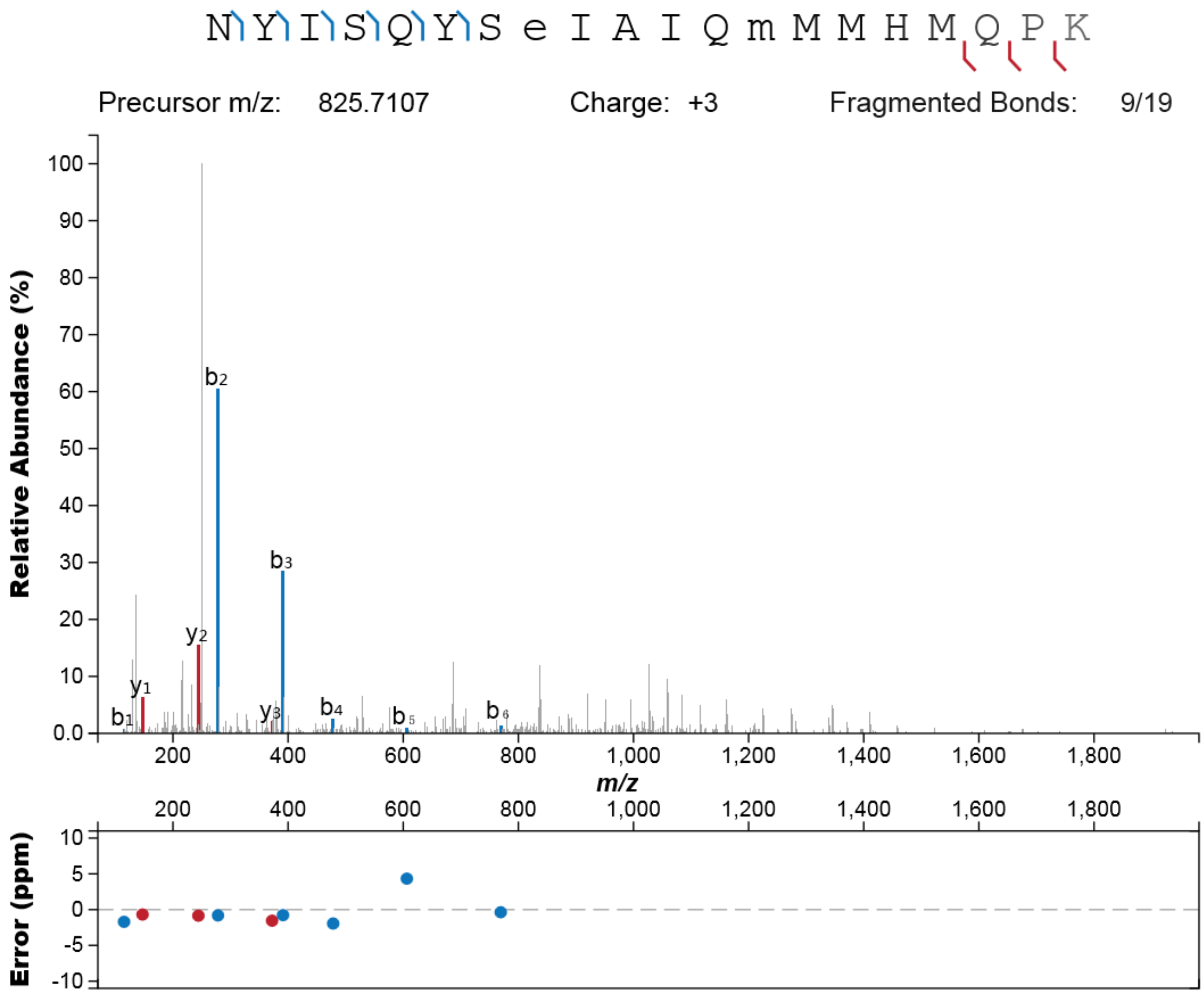
N Y I S Q Y S e I A I Q m M M H M Q P K
Precursor m/z: 825.7107
Charge: +3
Fragmented Bonds: 9/19
Relative Abundance (%)
m/z
Error (ppm)
b1
y1
y2
b2
b3
y3
b4
b5
b6

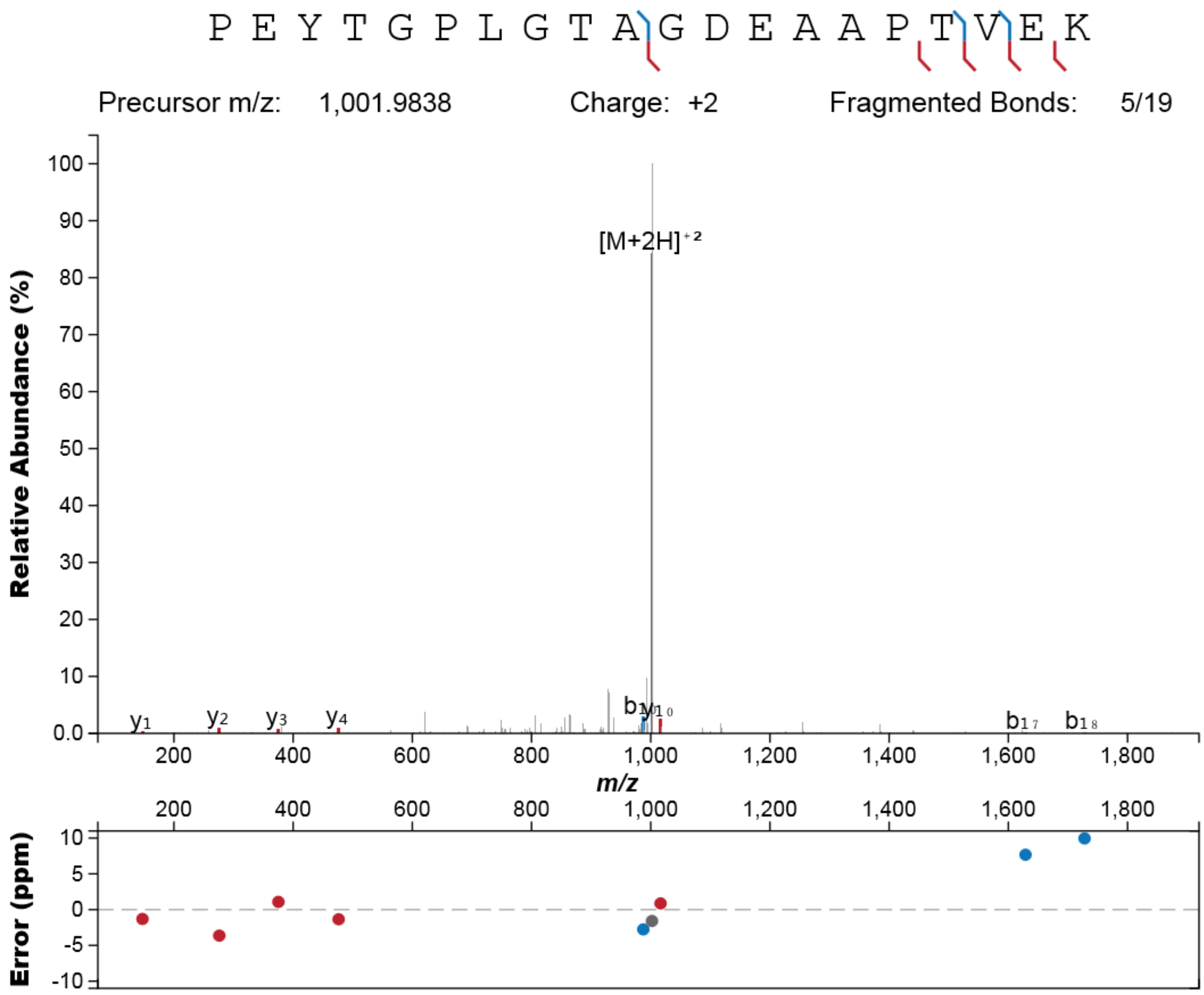

P E Y T G P L G T A G D E A A P T V E K
Precursor m/z: 1,001.9838
Charge: +2
Fragmented Bonds: 5/19
[M+2H]+2
y1
y2
y3
y4
b1y10
b17
b18
Relative Abundance (%)
m/z
Error (ppm)

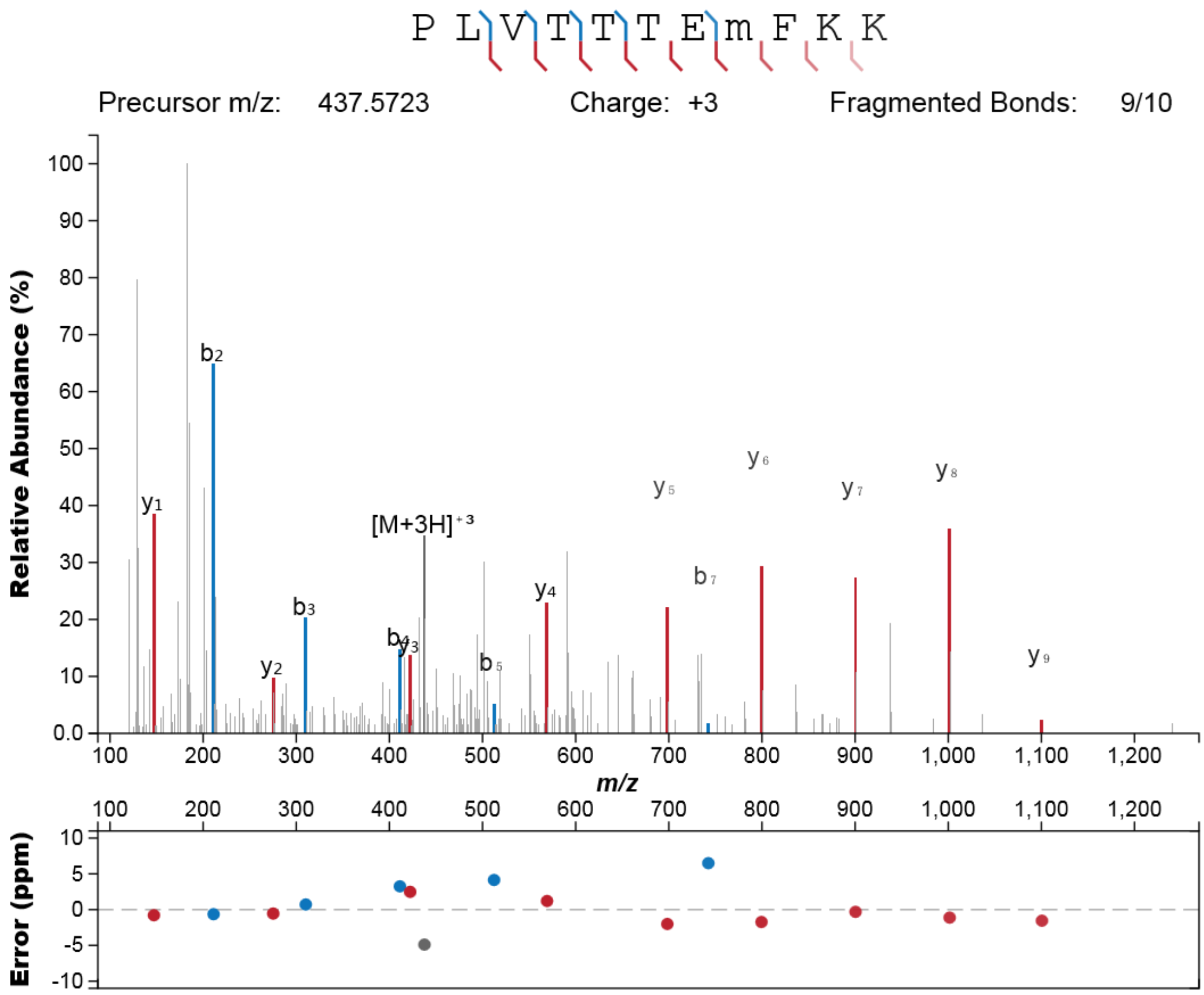

P L V T T T E m F K K
Precursor m/z: 437.5723
Charge: +3
Fragmented Bonds: 9/10
Relative Abundance (%)
m/z
Error (ppm)
y1
b2
y2
b3
[M+3H]+3
b4
y3
b5
y4
y5
y6
b7
y7
y8
y9

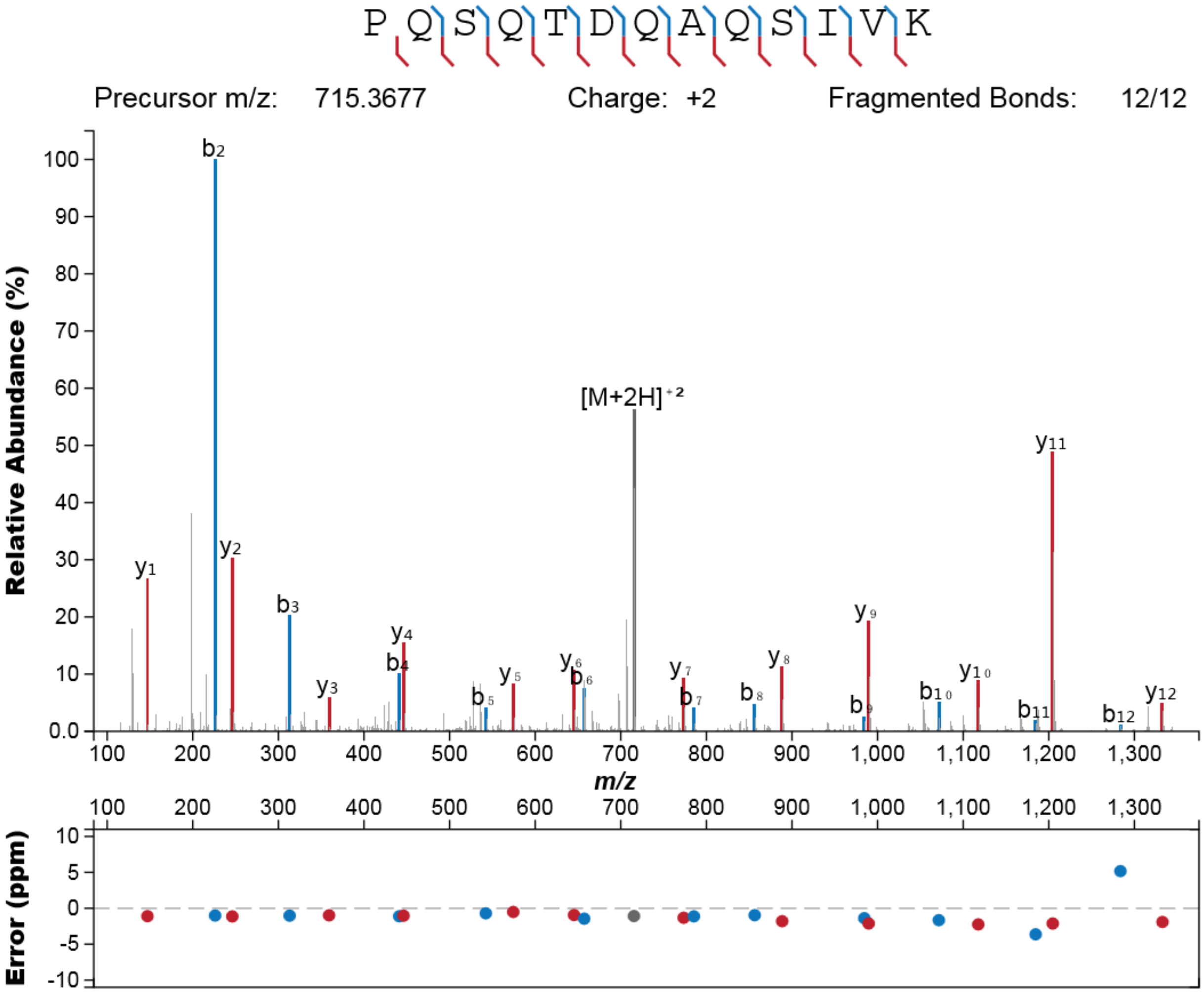

P Q S Q T D Q A Q S I V K
Precursor m/z: 715.3677
Charge: +2
Fragmented Bonds: 12/12
Relative Abundance (%)
m/z
Error (ppm)
[M+2H]+2

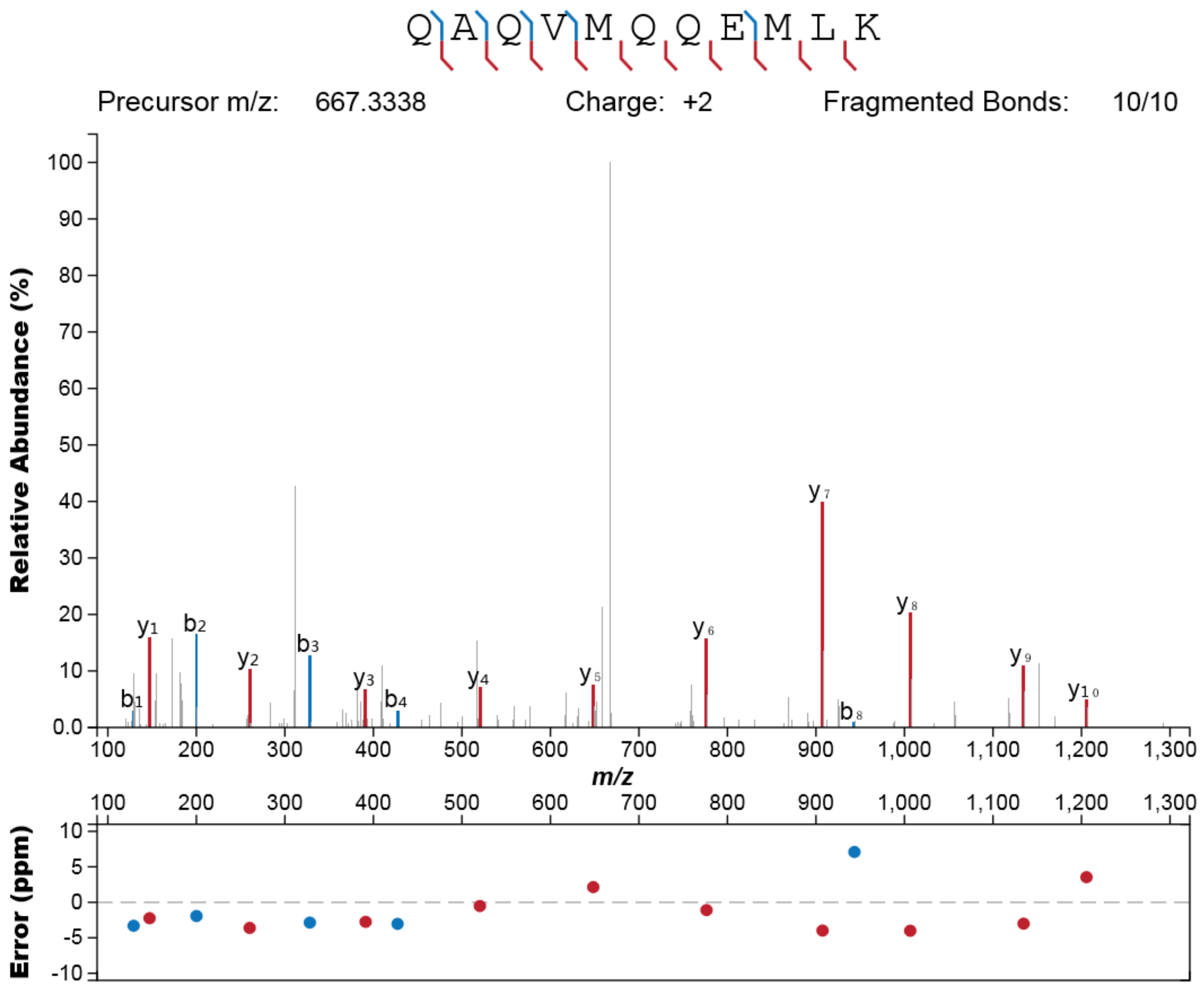

Q A Q V M Q Q E M L K
Precursor m/z: 667.3338
Charge: +2
Fragmented Bonds: 10/10
Relative Abundance (%)
m/z
Error (ppm)
y1
b2
y2
b3
y3
b4
y4
y5
y6
y7
b8
y8
y9
y10
b1

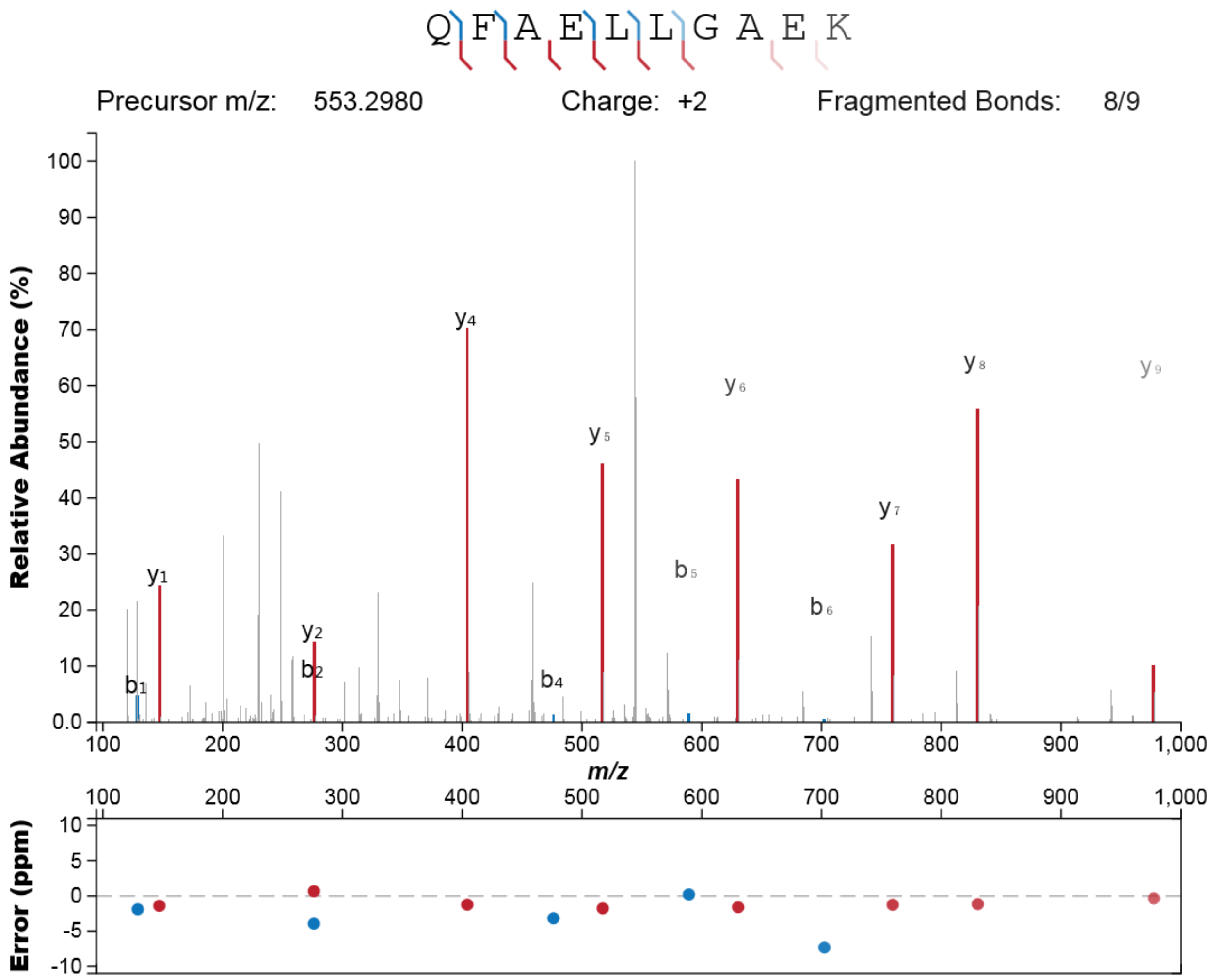

Q F A E L L G A E K
Precursor m/z: 553.2980
Charge: +2
Fragmented Bonds: 8/9
Relative Abundance (%)
m/z
Error (ppm)
y1
y2
y4
y5
y6
y7
y8
y9
b1
b2
b4
b5
b6

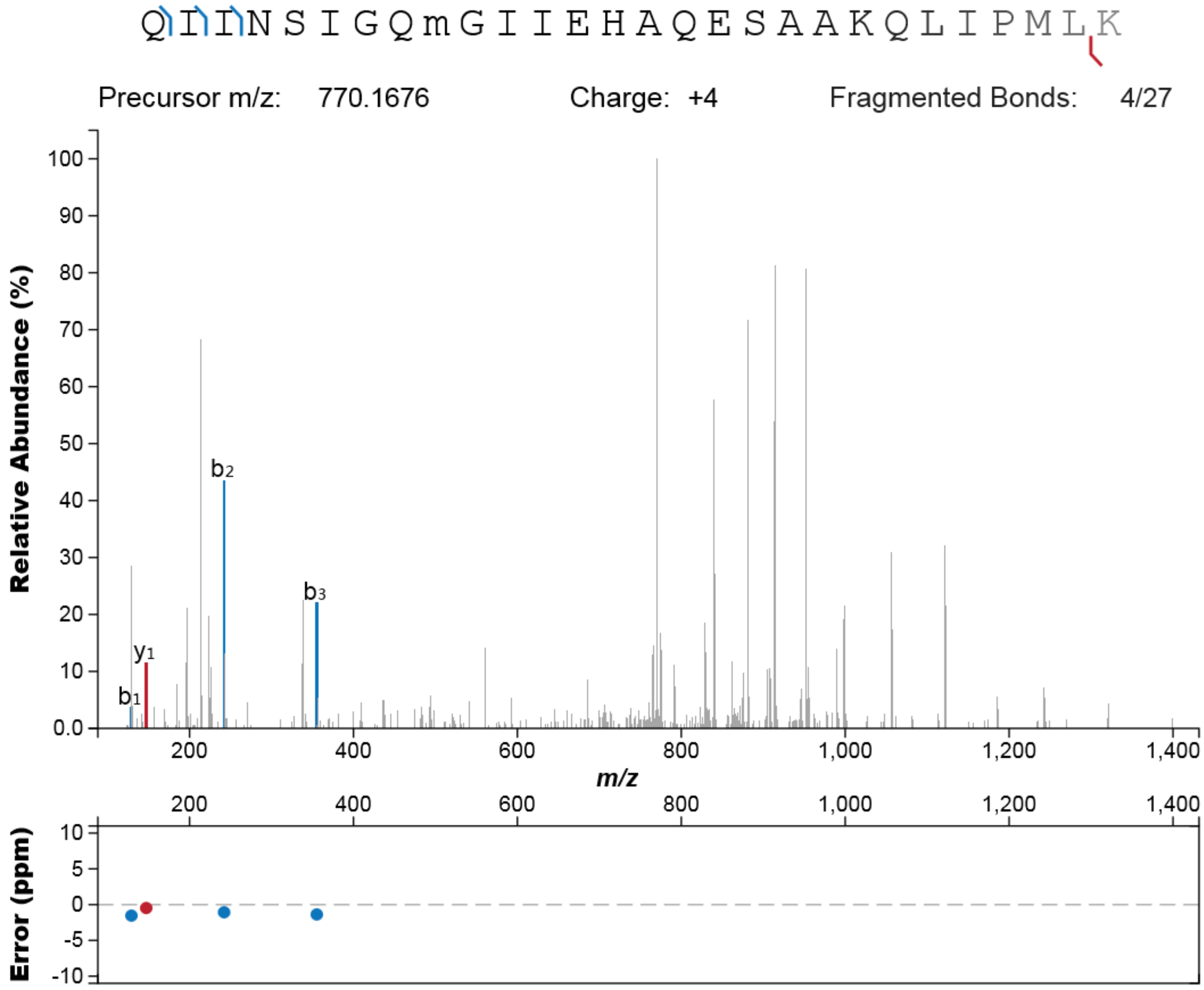

Q I I I N S I G Q m G I I E H A Q E S A A K Q L I P M L K
Precursor m/z: 770.1676
Charge: +4
Fragmented Bonds: 4/27
Relative Abundance (%)
b2
b3
y1
b1
m/z
Error (ppm)

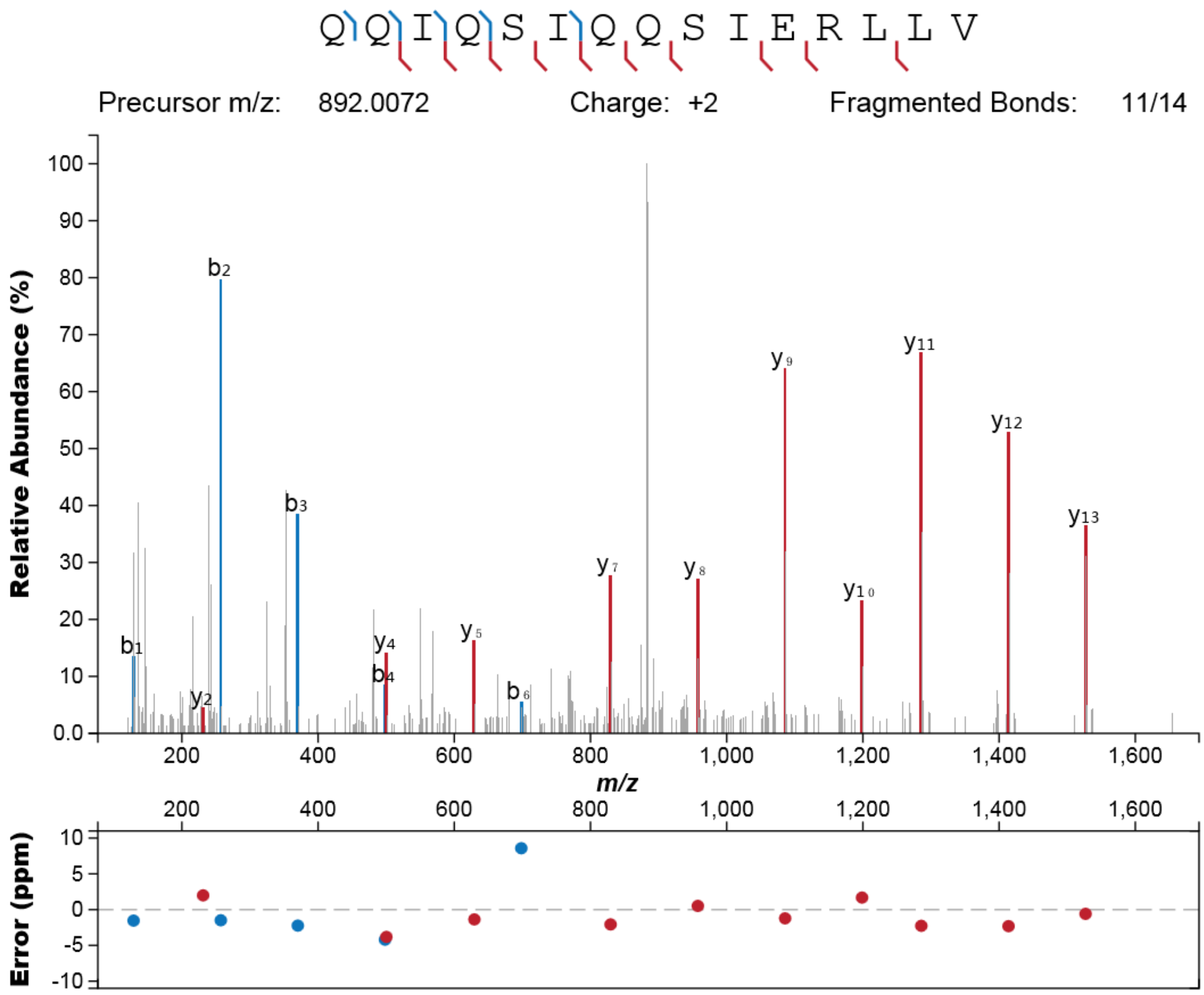

Q Q I Q S I Q Q S I E R L L V
Precursor m/z: 892.0072
Charge: +2
Fragmented Bonds: 11/14
Relative Abundance (%)
m/z
Error (ppm)
b1
b2
b3
b4
b6
y2
y4
y5
y7
y8
y9
y10
y11
y12
y13

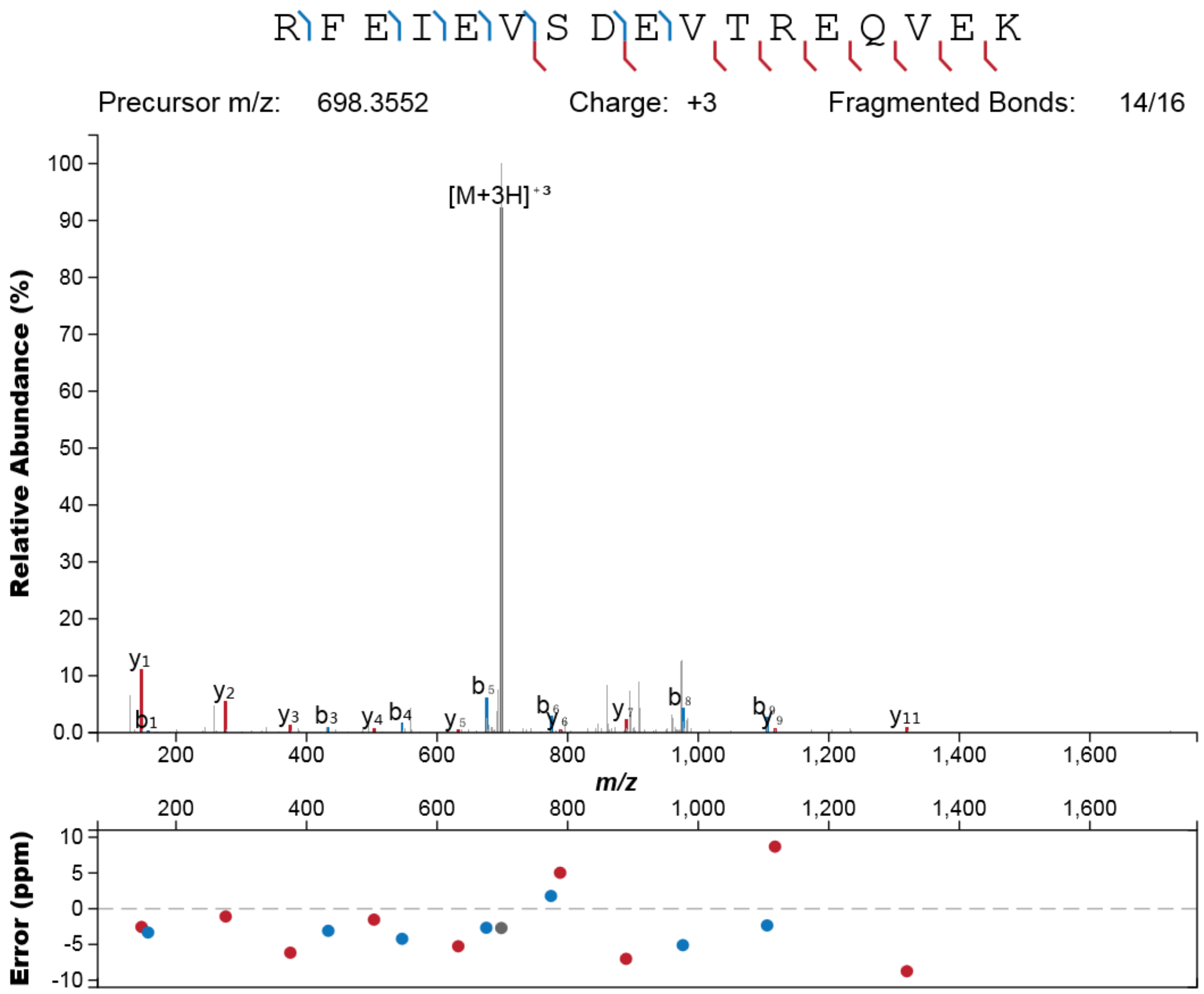
R F E I E V S D E V T R E Q V E K
Precursor m/z: 698.3552
Charge: +3
Fragmented Bonds: 14/16
[M+3H]+3
Relative Abundance (%)
m/z
Error (ppm)
y1
b1
y2
y3
b3
y4
b4
y5
b5
b6
y6
y7
b8
b9
y9
y11

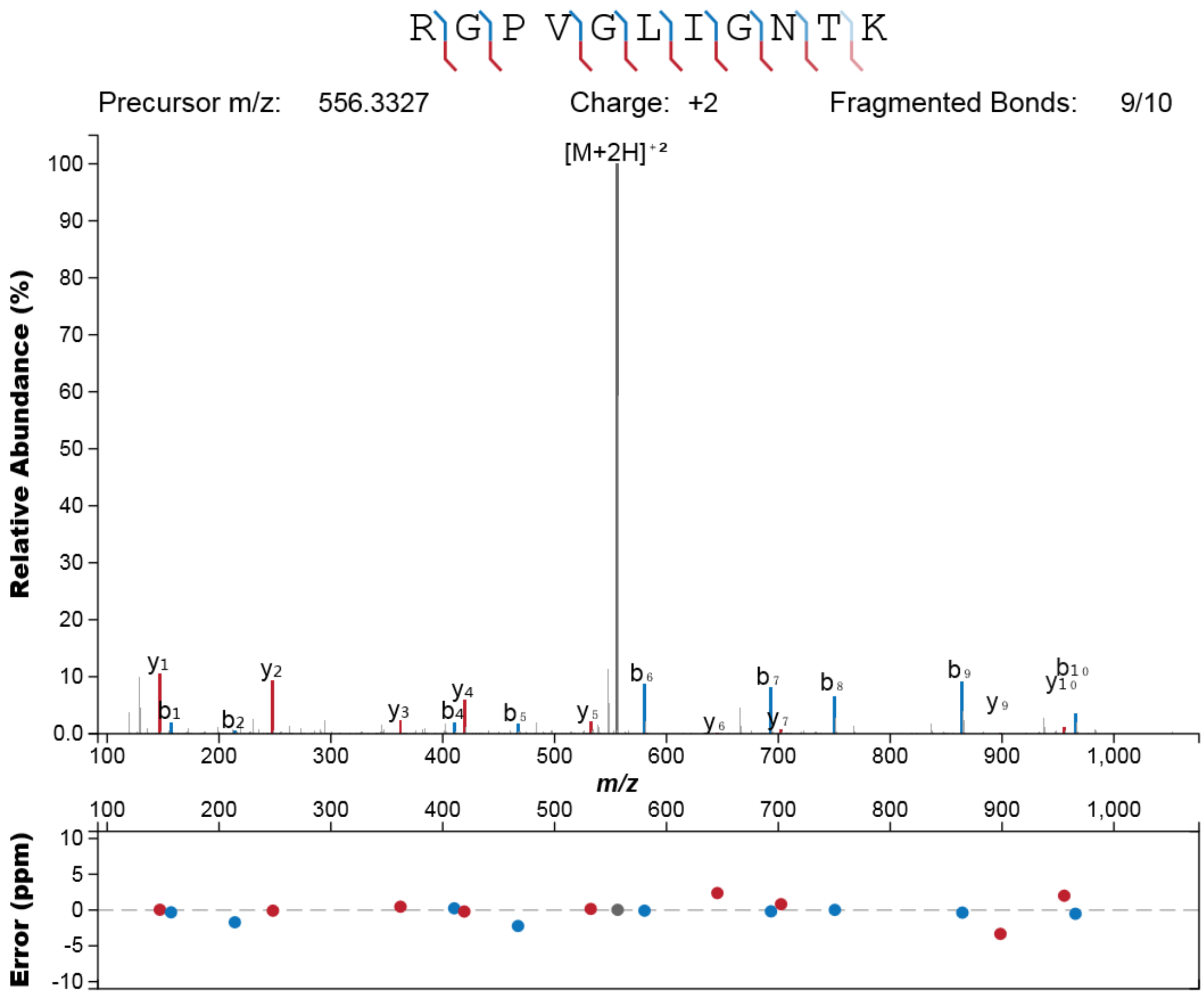

R G P V G L I G N T K
Precursor m/z: 556.3327
Charge: +2
Fragmented Bonds: 9/10
[M+2H]+2
Relative Abundance (%)
m/z
Error (ppm)
y1 b1 b2 y2 y3 b4 y4 b5 y5 b6 y6 b7 y7 b8 b9 y9 y10 b10

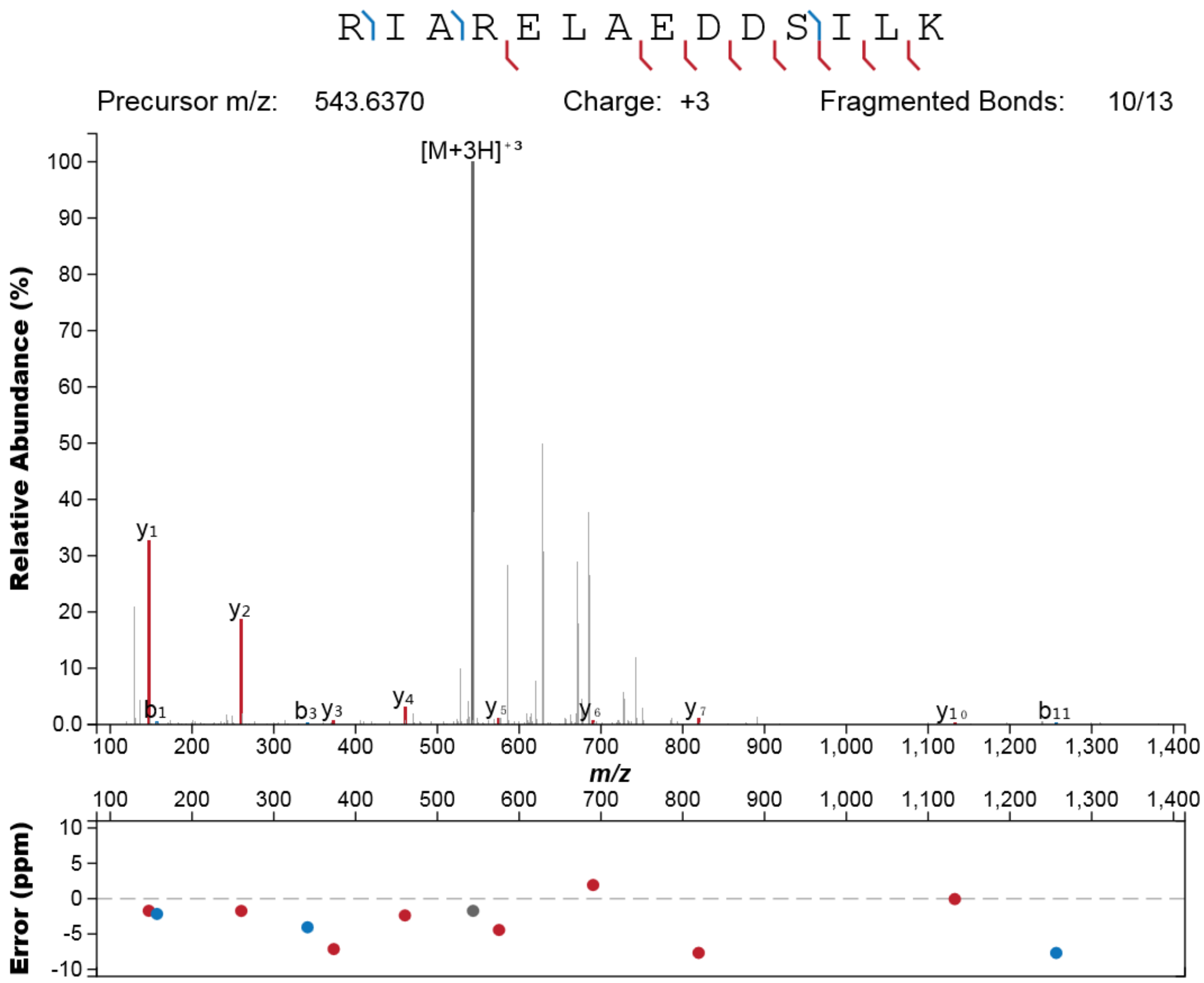
R I A R E L A E D D S I L K
Precursor m/z: 543.6370
Charge: +3
Fragmented Bonds: 10/13
[M+3H]+3
Relative Abundance (%)
m/z
Error (ppm)
y1
y2
b1
b3
y3
y4
y5
y6
y7
y10
b11

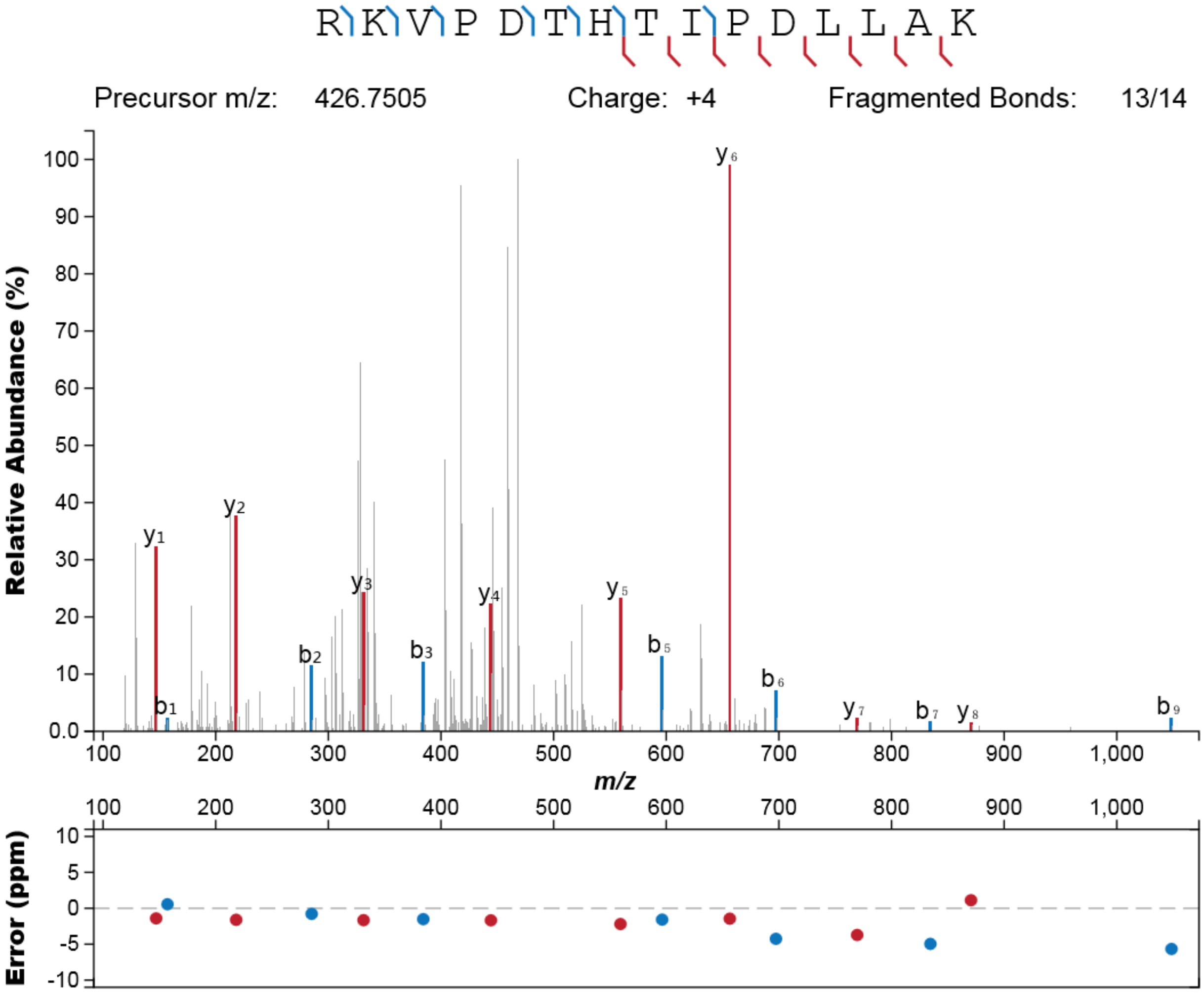
R K V P D T H T I P D L L A K
Precursor m/z: 426.7505
Charge: +4
Fragmented Bonds: 13/14
Relative Abundance (%)
m/z
Error (ppm)
y1
y2
y3
y4
y5
y6
y7
y8
b1
b2
b3
b5
b6
b7
b9

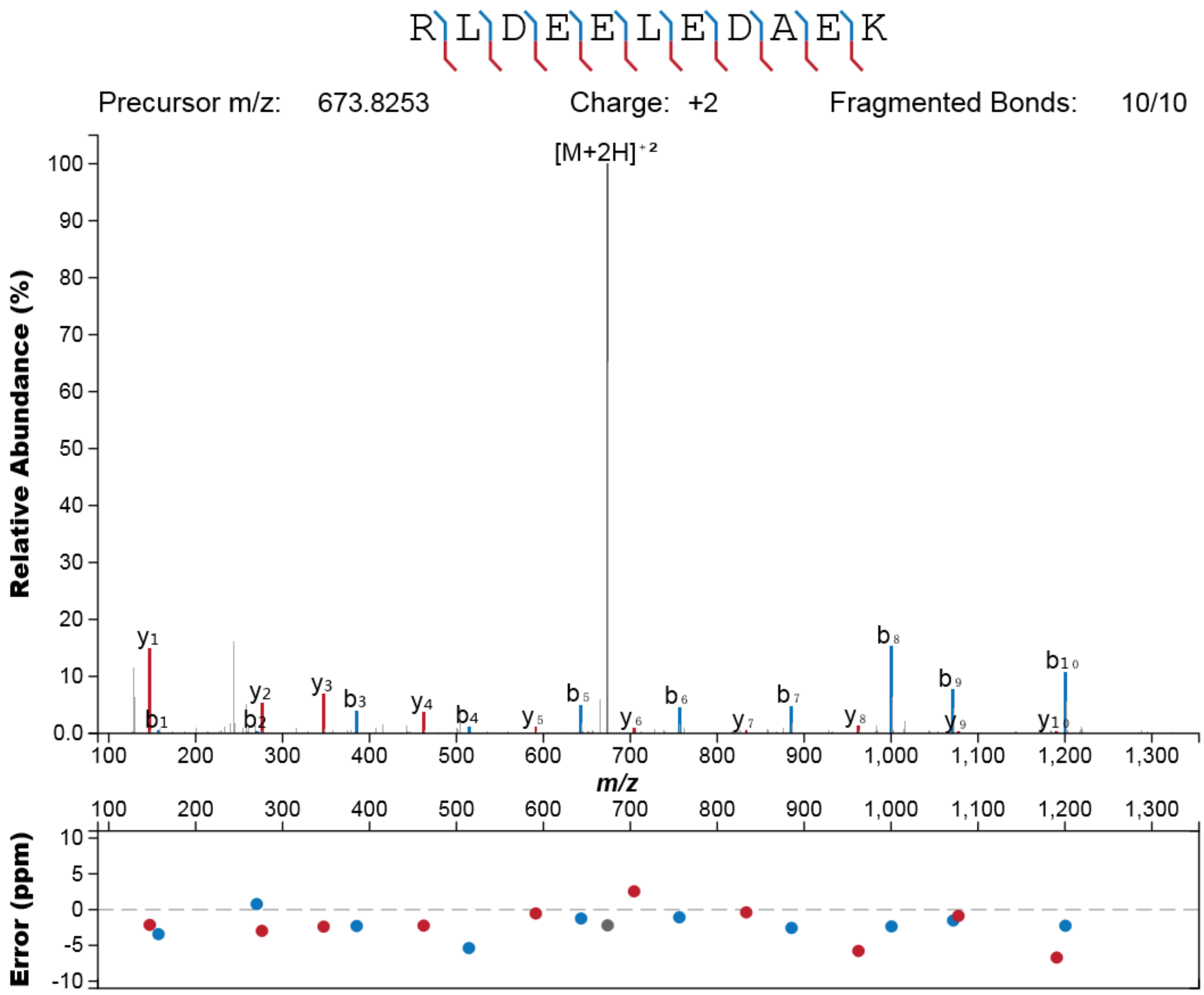
R L D E E L E D A E K
Precursor m/z: 673.8253
Charge: +2
Fragmented Bonds: 10/10
[M+2H]+2
Relative Abundance (%)
m/z
Error (ppm)
y1 b1 y2 b2 y3 b3 y4 b4 y5 b5 y6 b6 y7 b7 y8 b8 y9 b9 y10 b10

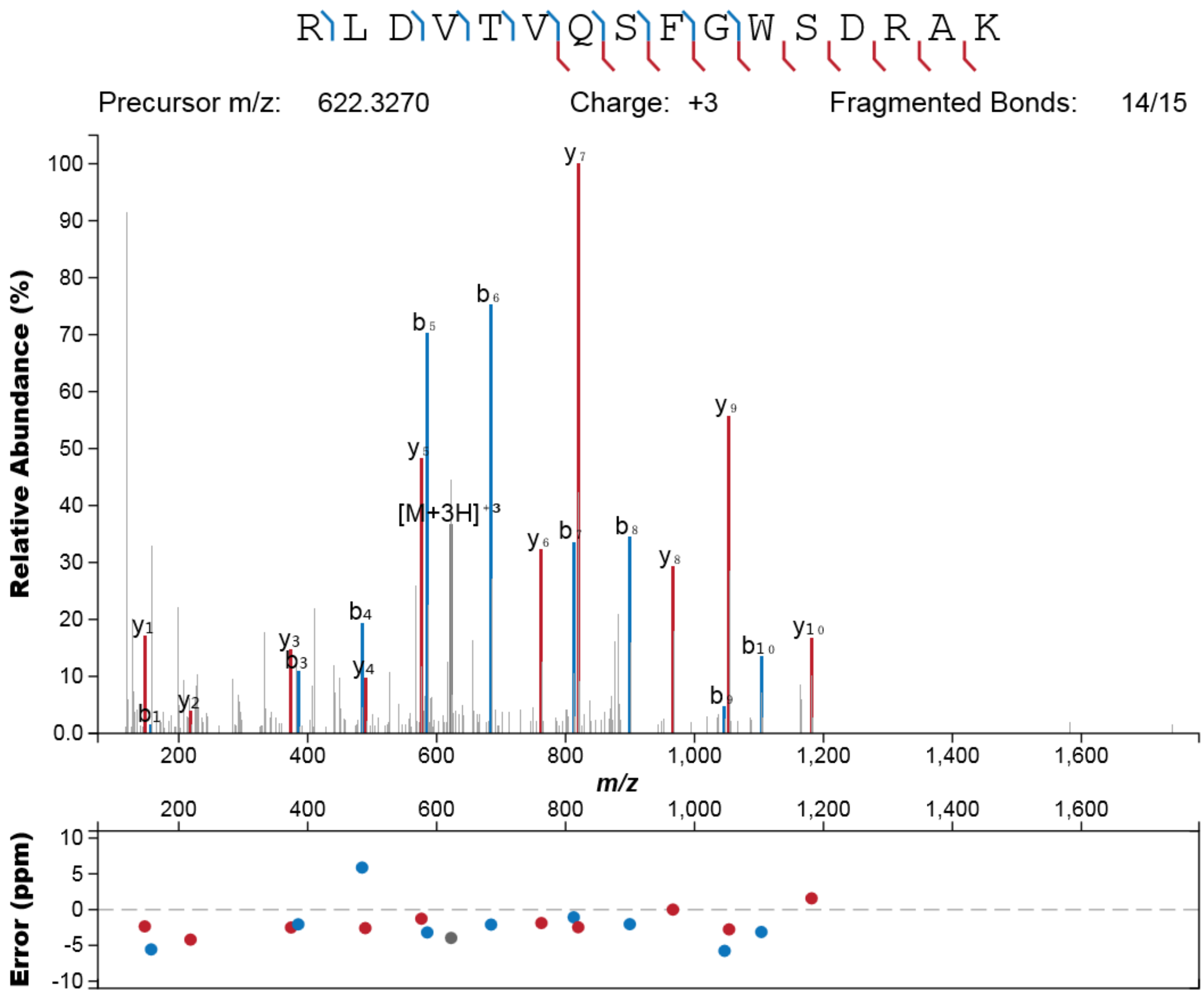

R L D V T V Q S F G W S D R A K
Precursor m/z: 622.3270
Charge: +3
Fragmented Bonds: 14/15
Relative Abundance (%)
m/z
Error (ppm)
[M+3H]+3

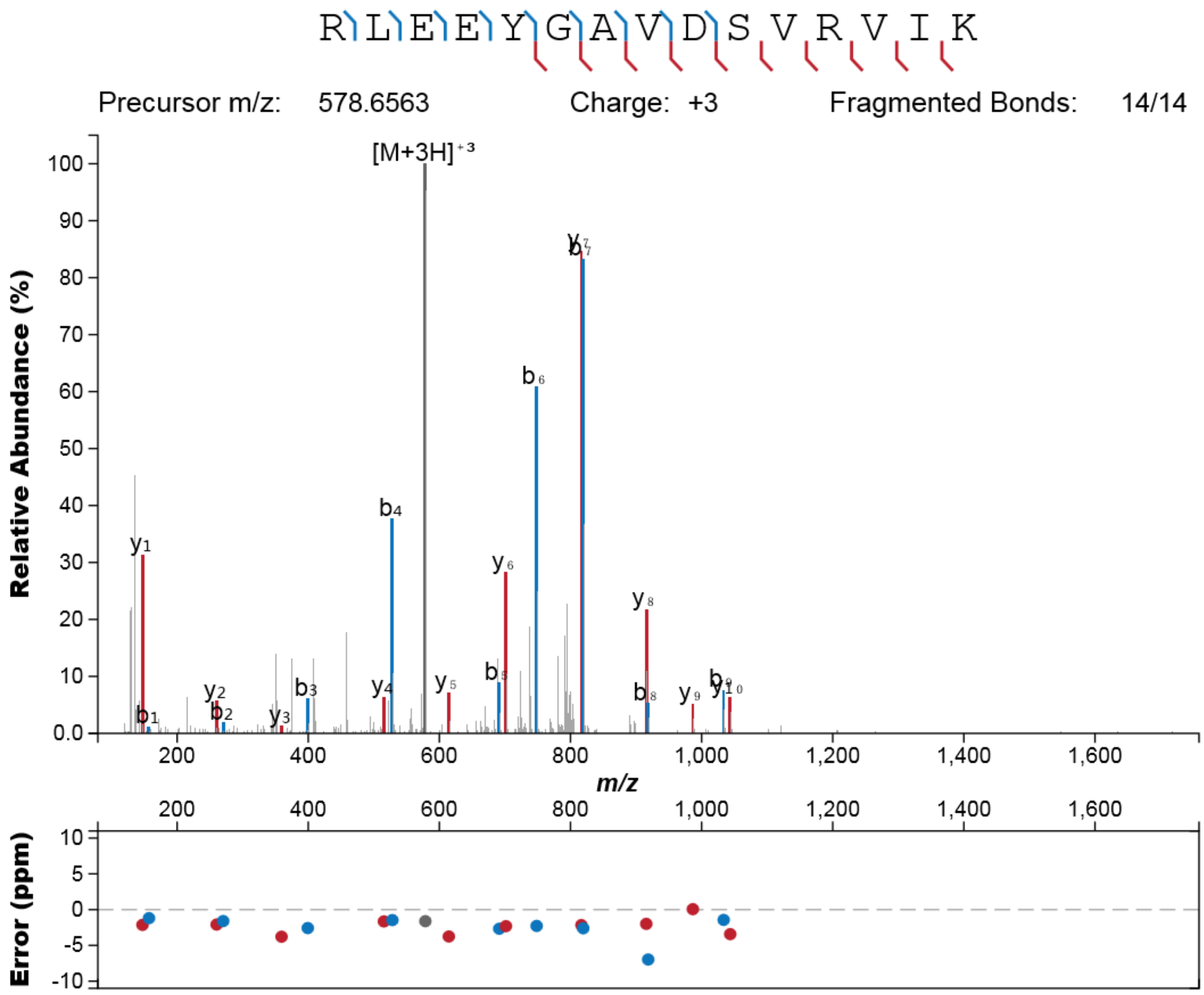
R L E E Y G A V D S V R V I K
Precursor m/z: 578.6563
Charge: +3
Fragmented Bonds: 14/14
[M+3H]+3
Relative Abundance (%)
m/z
Error (ppm)
y1 b1 y2 b2 y3 b3 y4 b4 y5 b5 y6 b6 y7 b7 y8 b8 y9 b9 y10

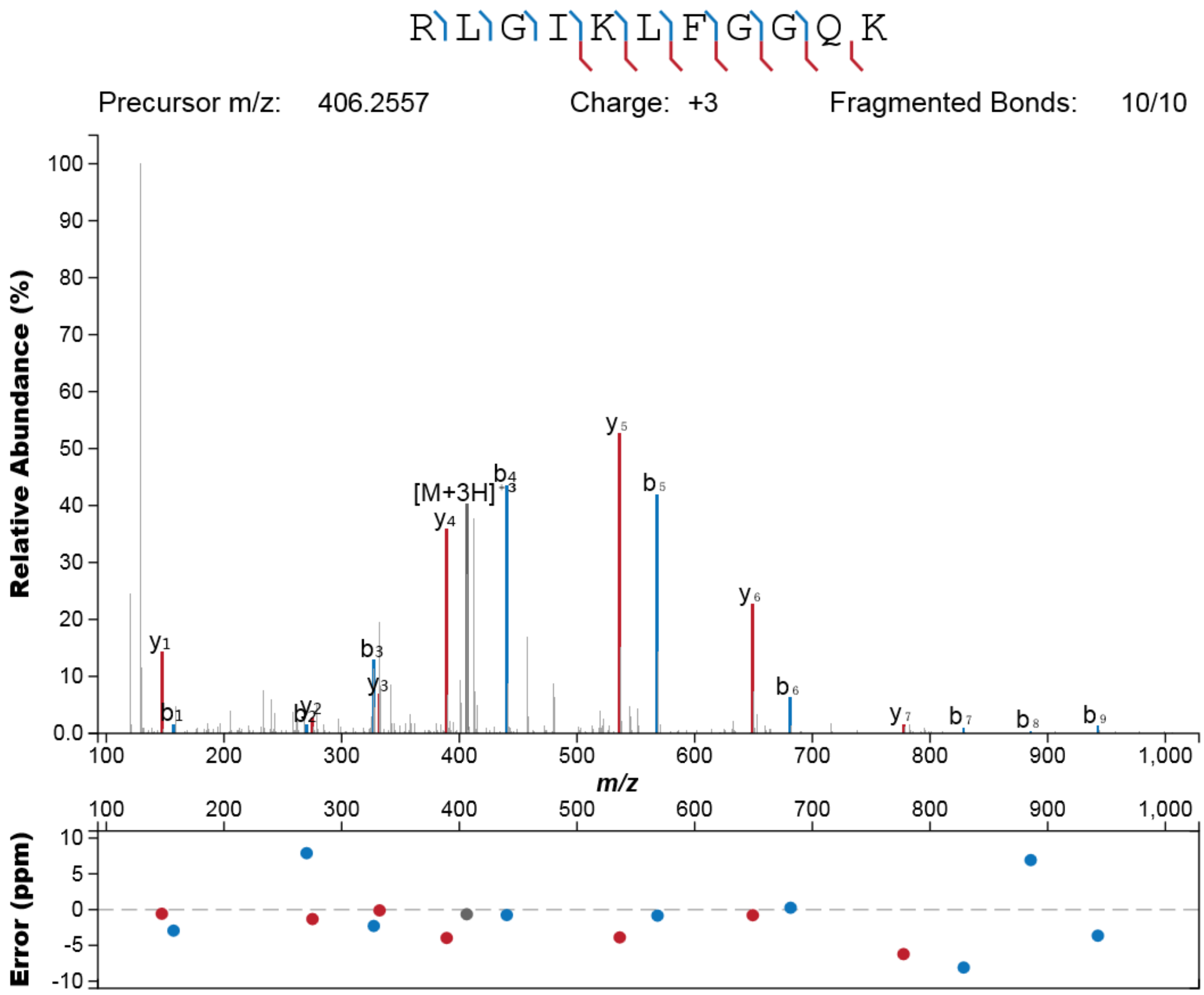
R L G I K L F G G Q K
Precursor m/z: 406.2557
Charge: +3
Fragmented Bonds: 10/10
Relative Abundance (%)
m/z
Error (ppm)
[M+3H]+3
y1
b1
y2
b2
b3
y3
y4
b4
y5
b5
y6
b6
y7
b7
b8
b9

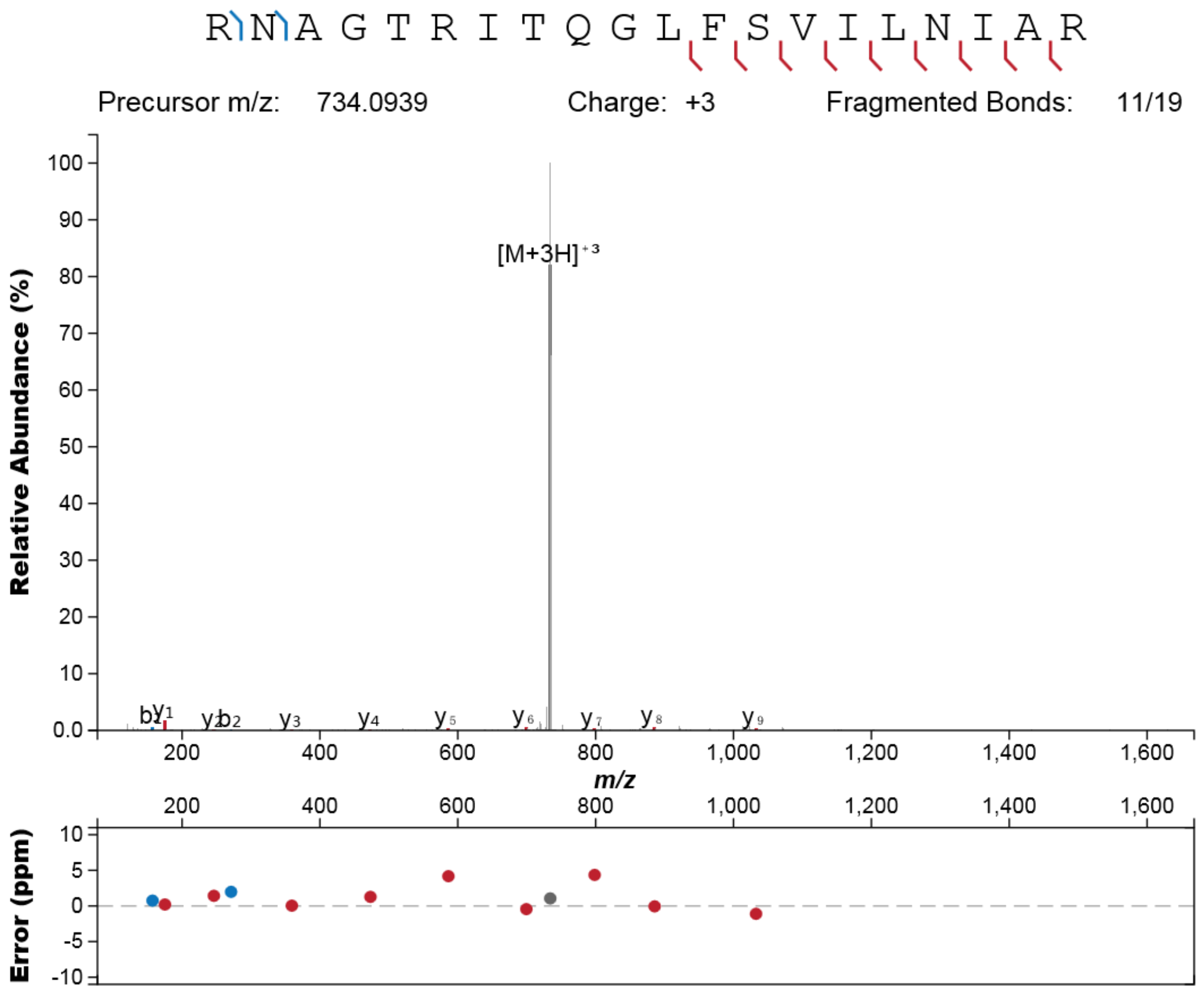

R N A G T R I T Q G L F S V I L N I A R
Precursor m/z: 734.0939
Charge: +3
Fragmented Bonds: 11/19
Relative Abundance (%)
[M+3H]+3
b1 y1 y2 b2 y3 y4 y5 y6 y7 y8 y9
m/z
Error (ppm)

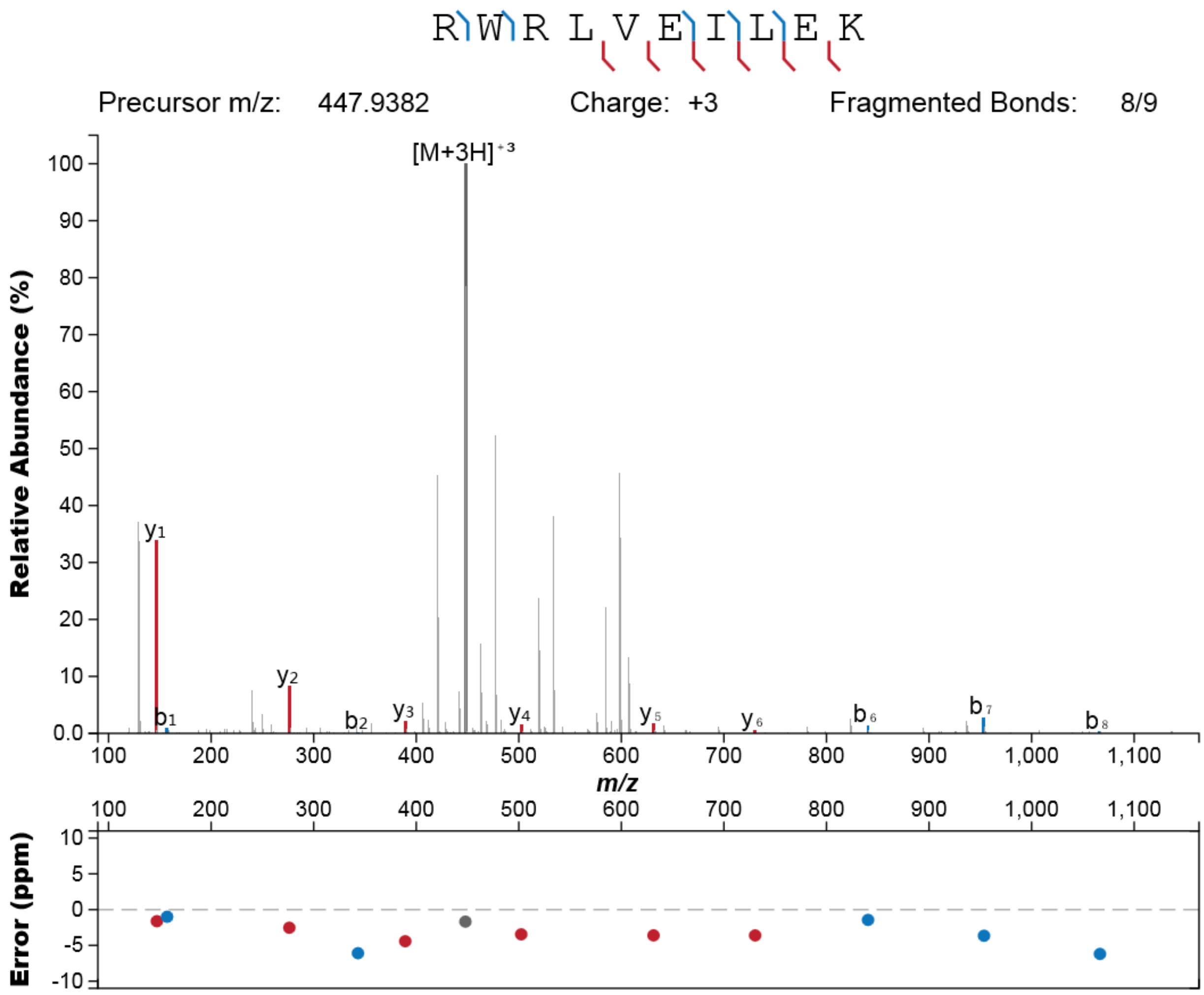

R W R L V E I L E K
Precursor m/z: 447.9382
Charge: +3
Fragmented Bonds: 8/9
[M+3H]+3
Relative Abundance (%)
m/z
Error (ppm)

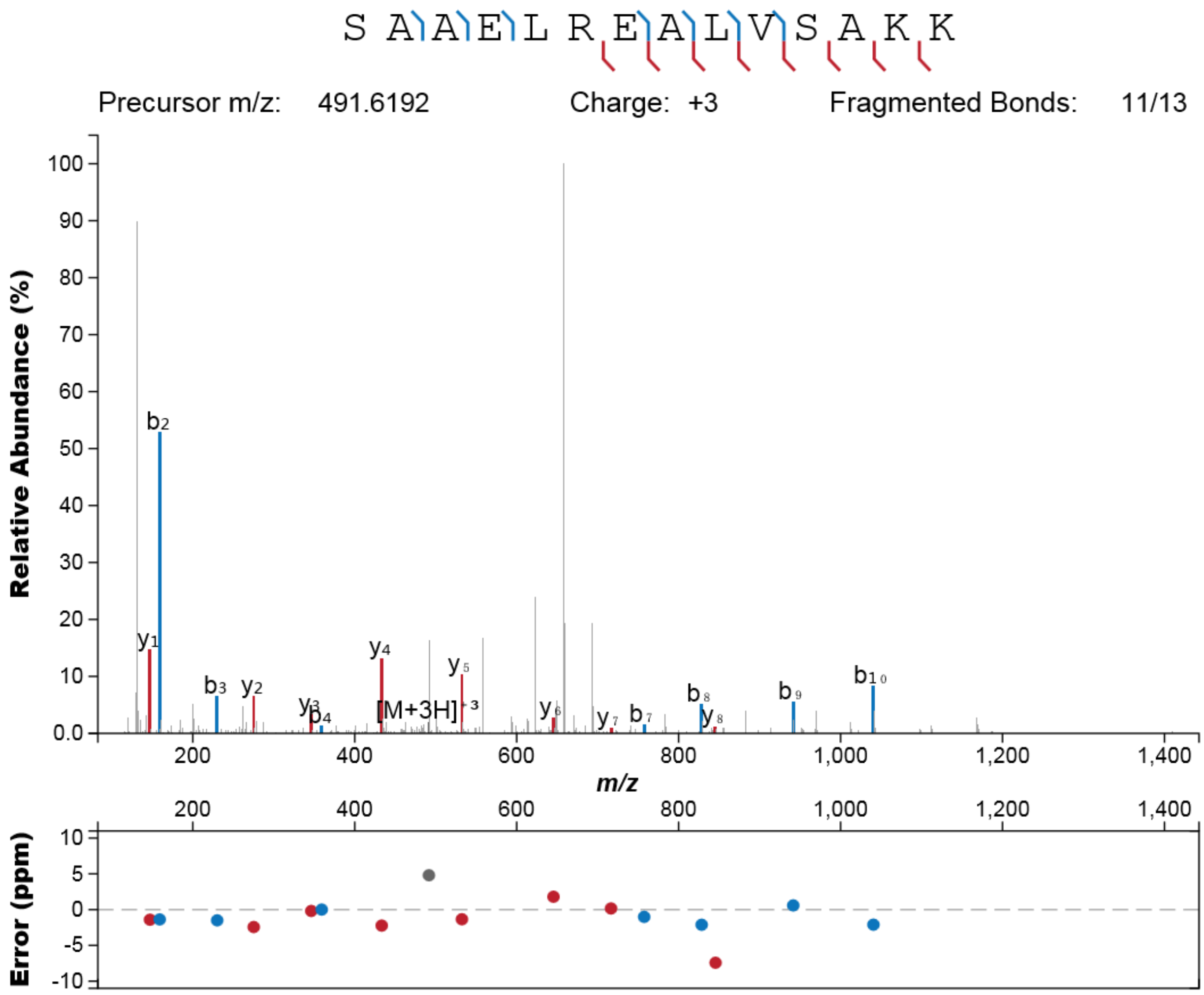
S A A E L R E A L V S A K K
Precursor m/z: 491.6192
Charge: +3
Fragmented Bonds: 11/13
Relative Abundance (%)
m/z
Error (ppm)
[M+3H]+3

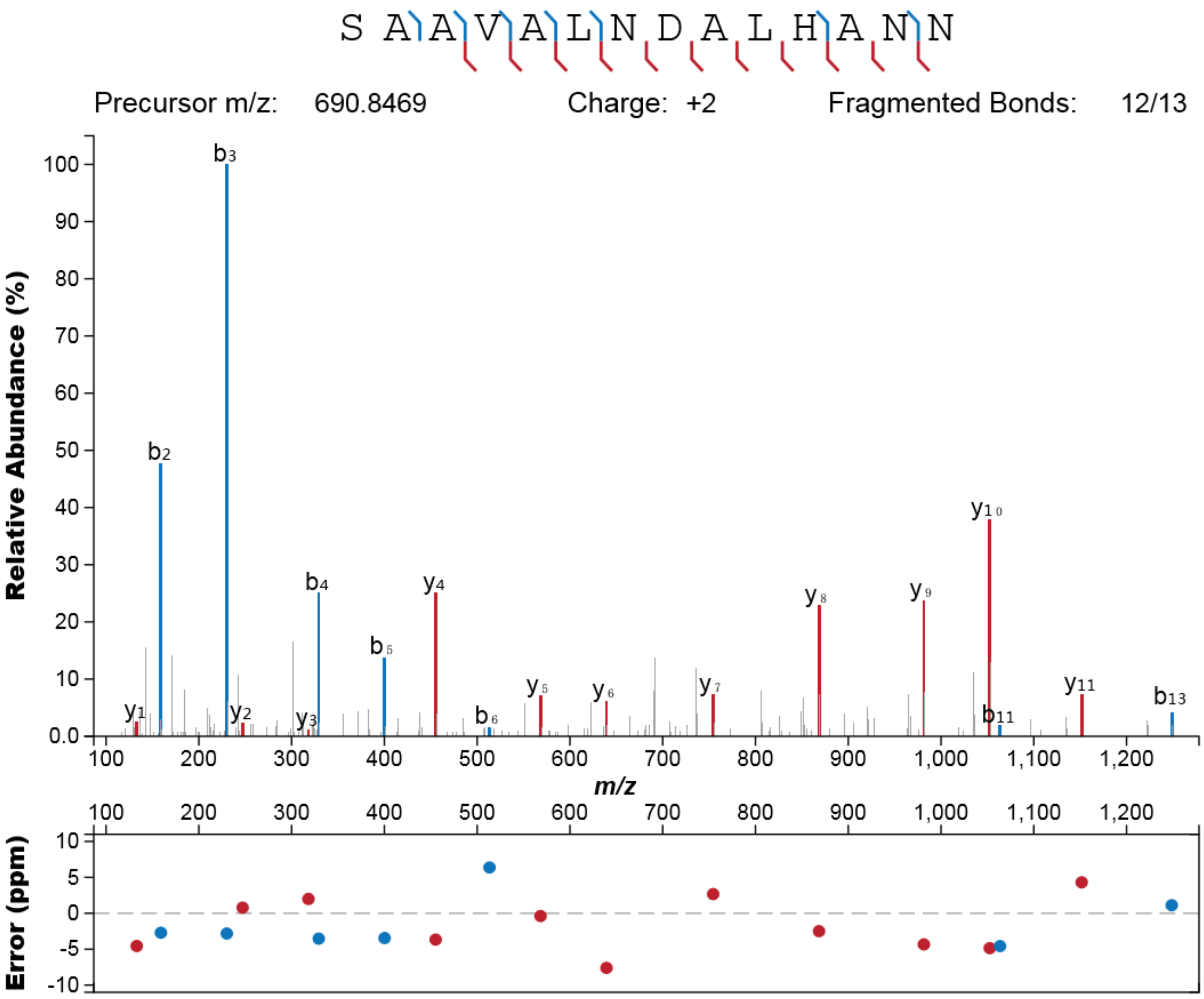
S A A V A L N D A L H A N N
Precursor m/z: 690.8469
Charge: +2
Fragmented Bonds: 12/13
Relative Abundance (%)
m/z
Error (ppm)
b2
b3
b4
b5
b6
b11
b13
y1
y2
y3
y4
y5
y6
y7
y8
y9
y10
y11

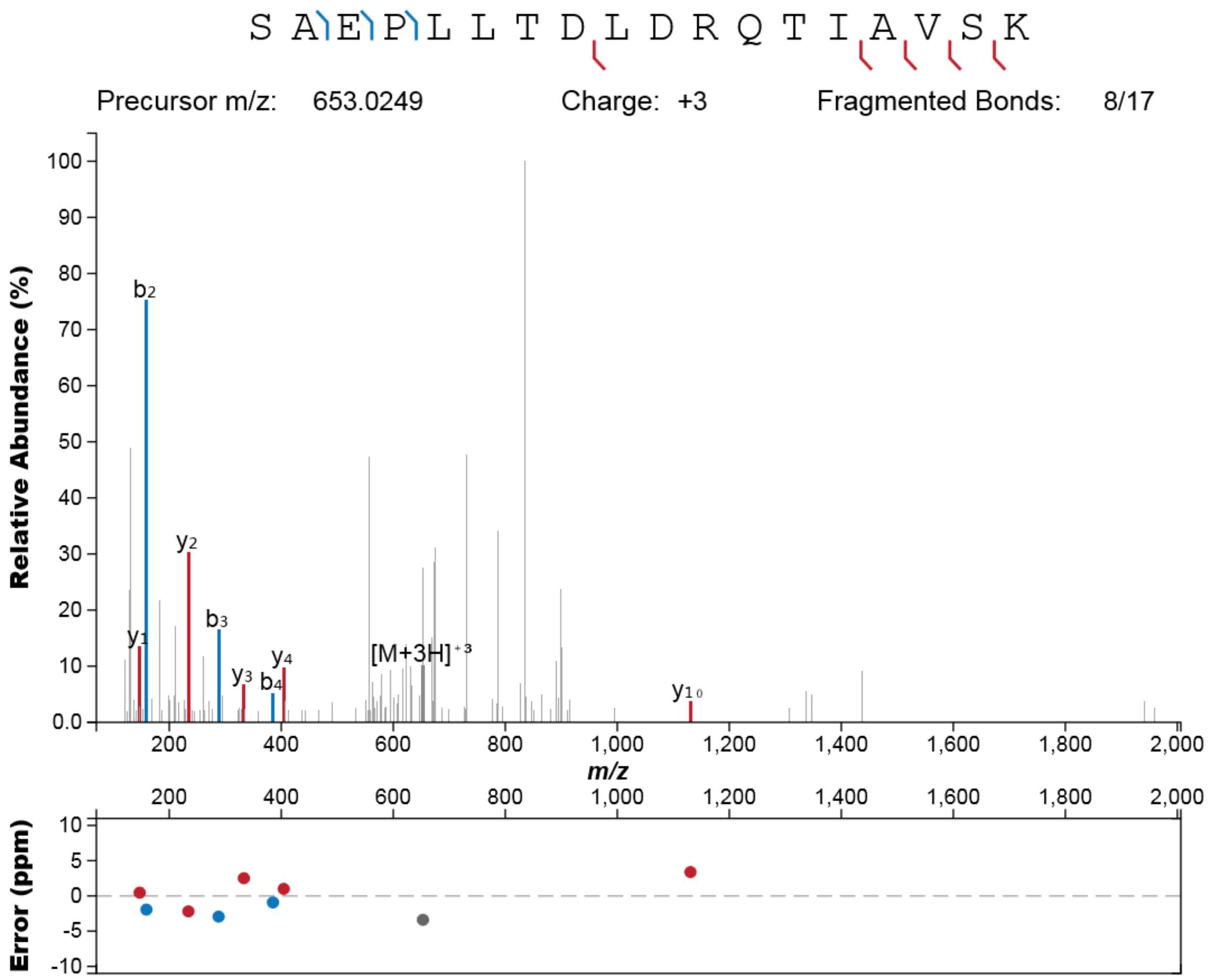
S A E P L L L T D L D R Q T I A V S K
Precursor m/z: 653.0249
Charge: +3
Fragmented Bonds: 8/17
Relative Abundance (%)
b2
y2
b3
y1
y3
b4
y4
[M+3H]+3
y10
m/z
Error (ppm)

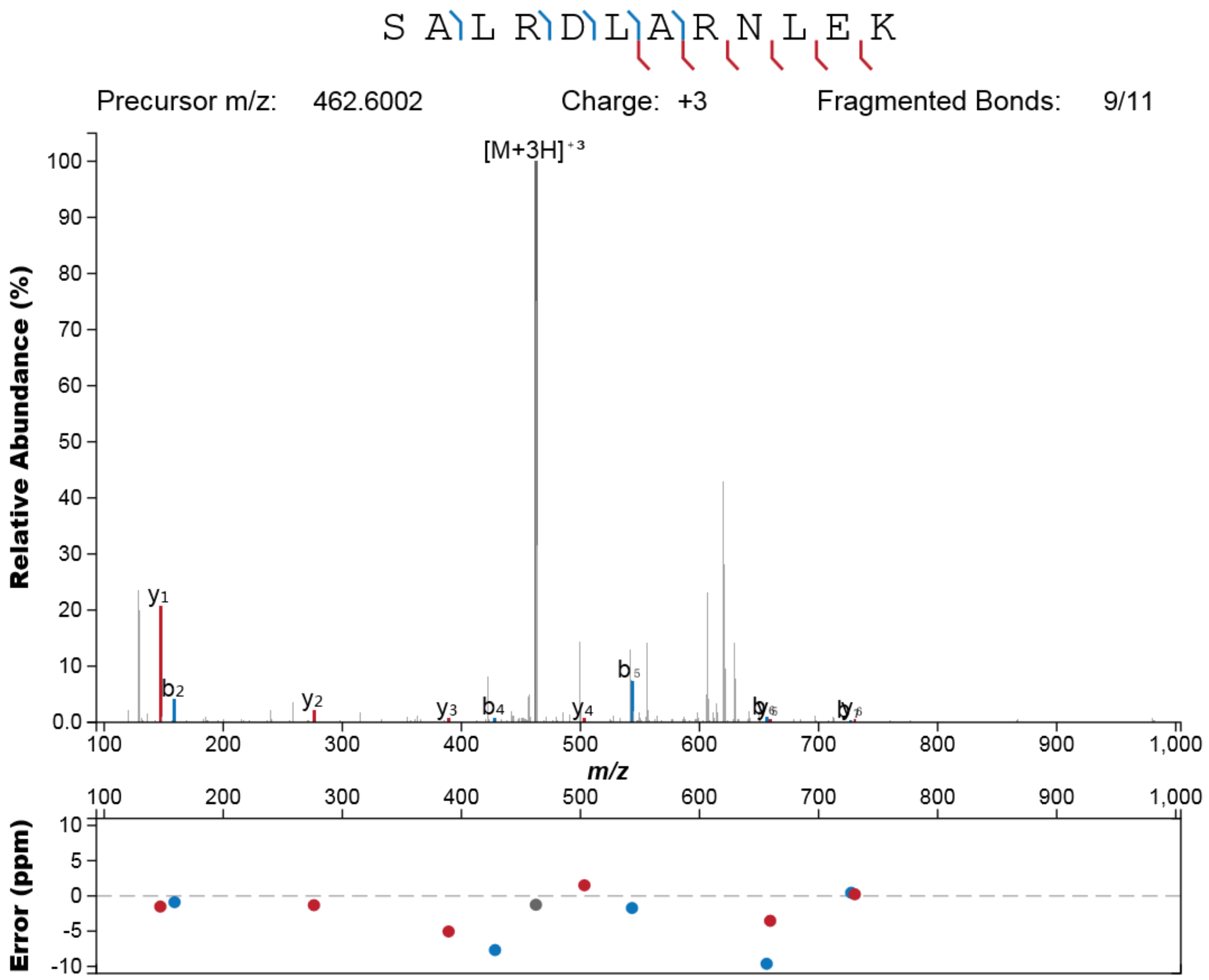
S A L R D L A R N L E K
Precursor m/z: 462.6002
Charge: +3
Fragmented Bonds: 9/11
[M+3H]+3
y1
b2
y2
y3
b4
y4
b5
Relative Abundance (%)
m/z
Error (ppm)

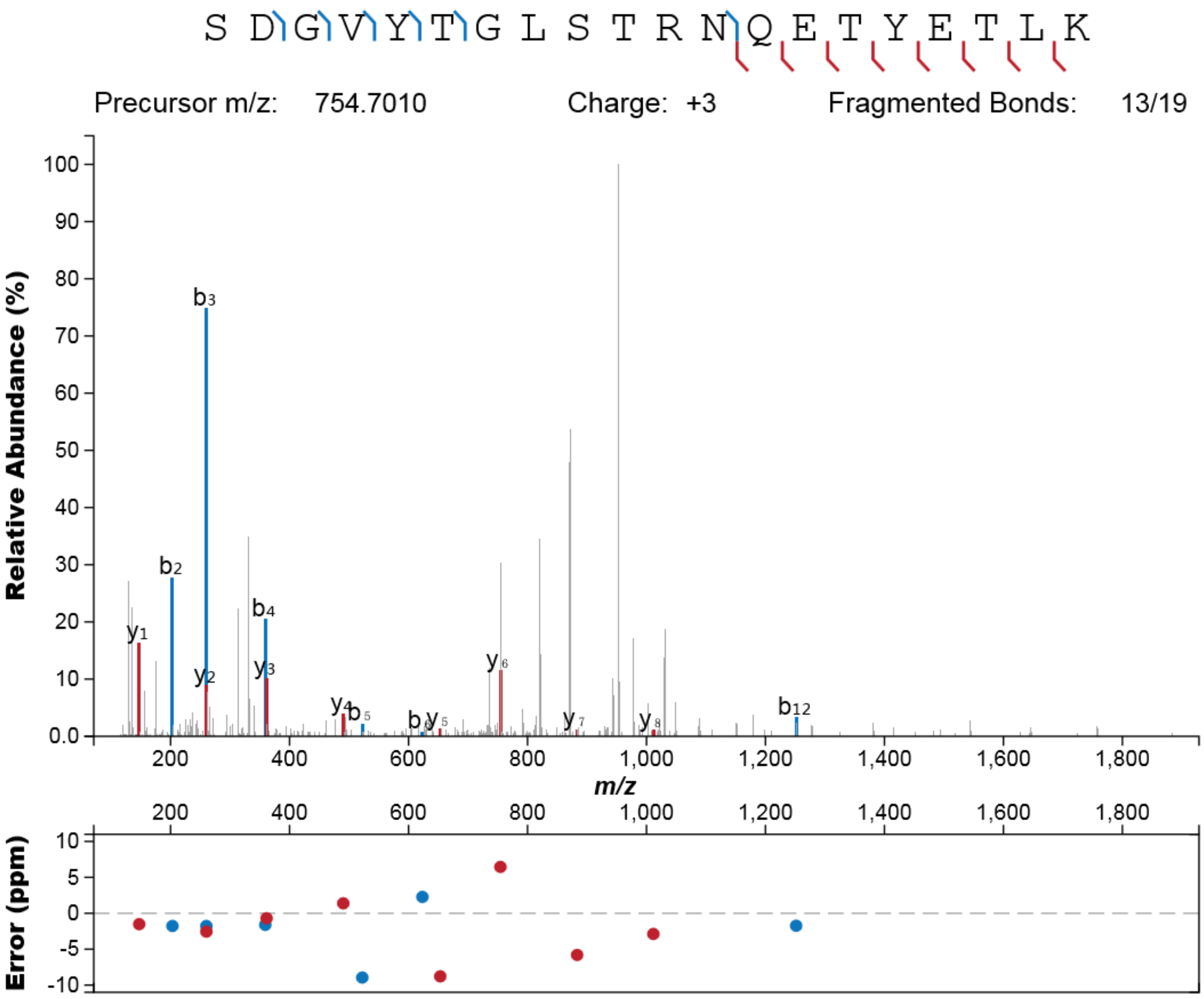

S D G V Y T G L S T R N Q E T Y E T L K
Precursor m/z: 754.7010
Charge: +3
Fragmented Bonds: 13/19
Relative Abundance (%)
m/z
Error (ppm)
b2
b3
b4
b5
b12
y1
y2
y3
y4
y5
y6
y7
y8

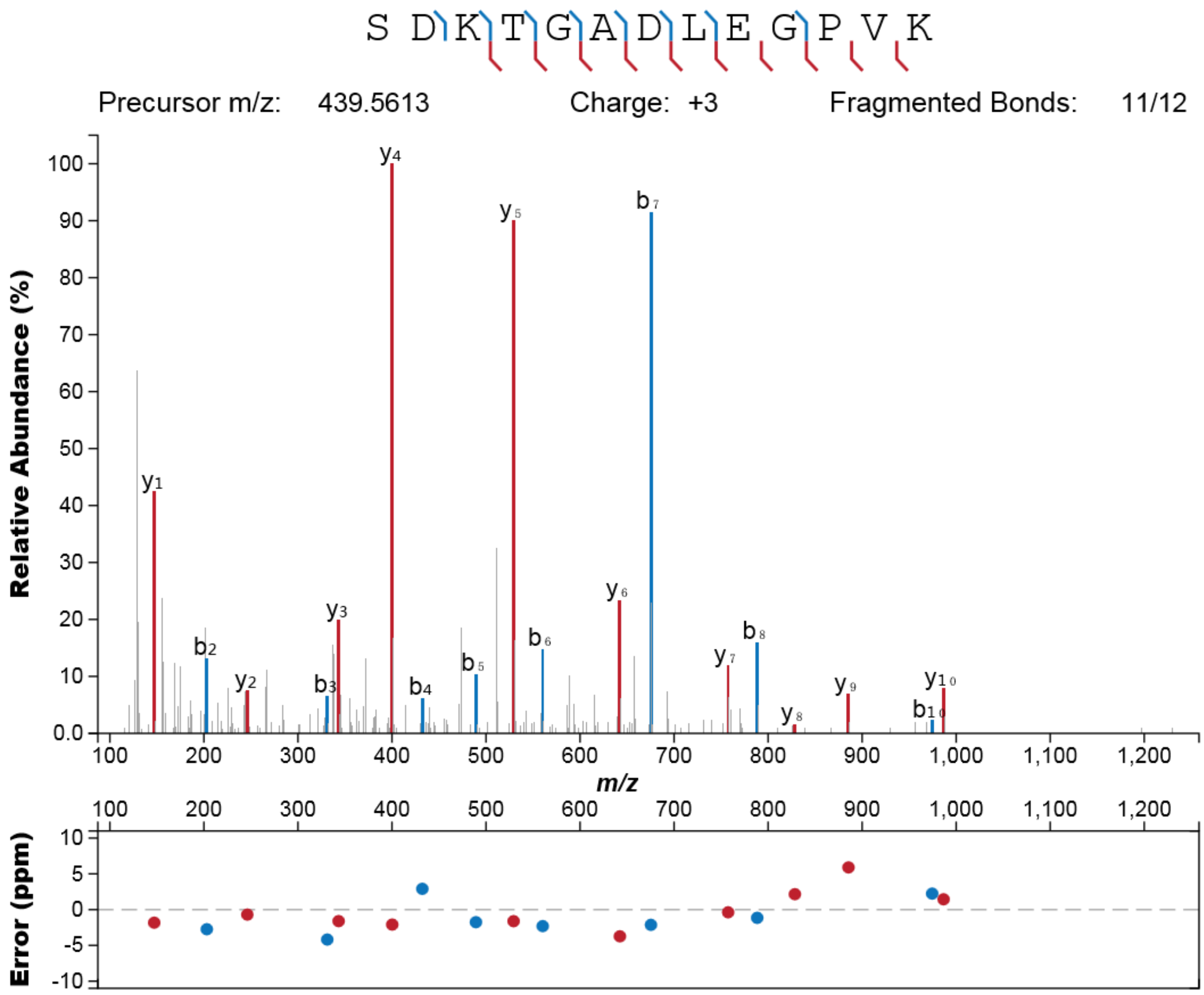

S D K T G A D L E G P V K
Precursor m/z: 439.5613
Charge: +3
Fragmented Bonds: 11/12
Relative Abundance (%)
m/z
Error (ppm)
y1
b2
y2
b3
y3
y4
b4
b5
y5
b6
y6
b7
y7
b8
y8
y9
b10
y10

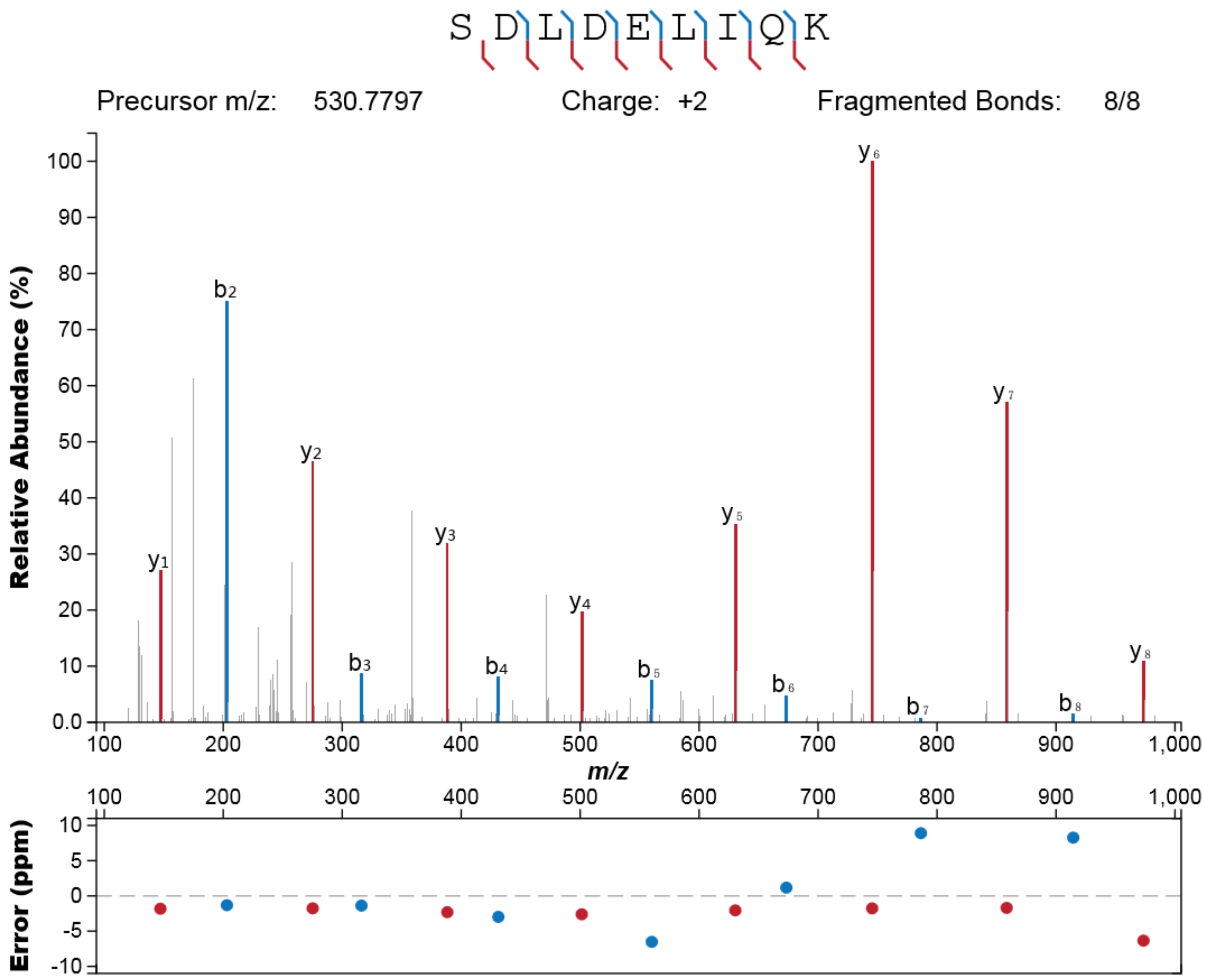

S D L D E L I Q K
Precursor m/z: 530.7797
Charge: +2
Fragmented Bonds: 8/8
Relative Abundance (%)
m/z
Error (ppm)
y1
b2
y2
b3
y3
b4
y4
b5
y5
b6
y6
b7
y7
b8
y8

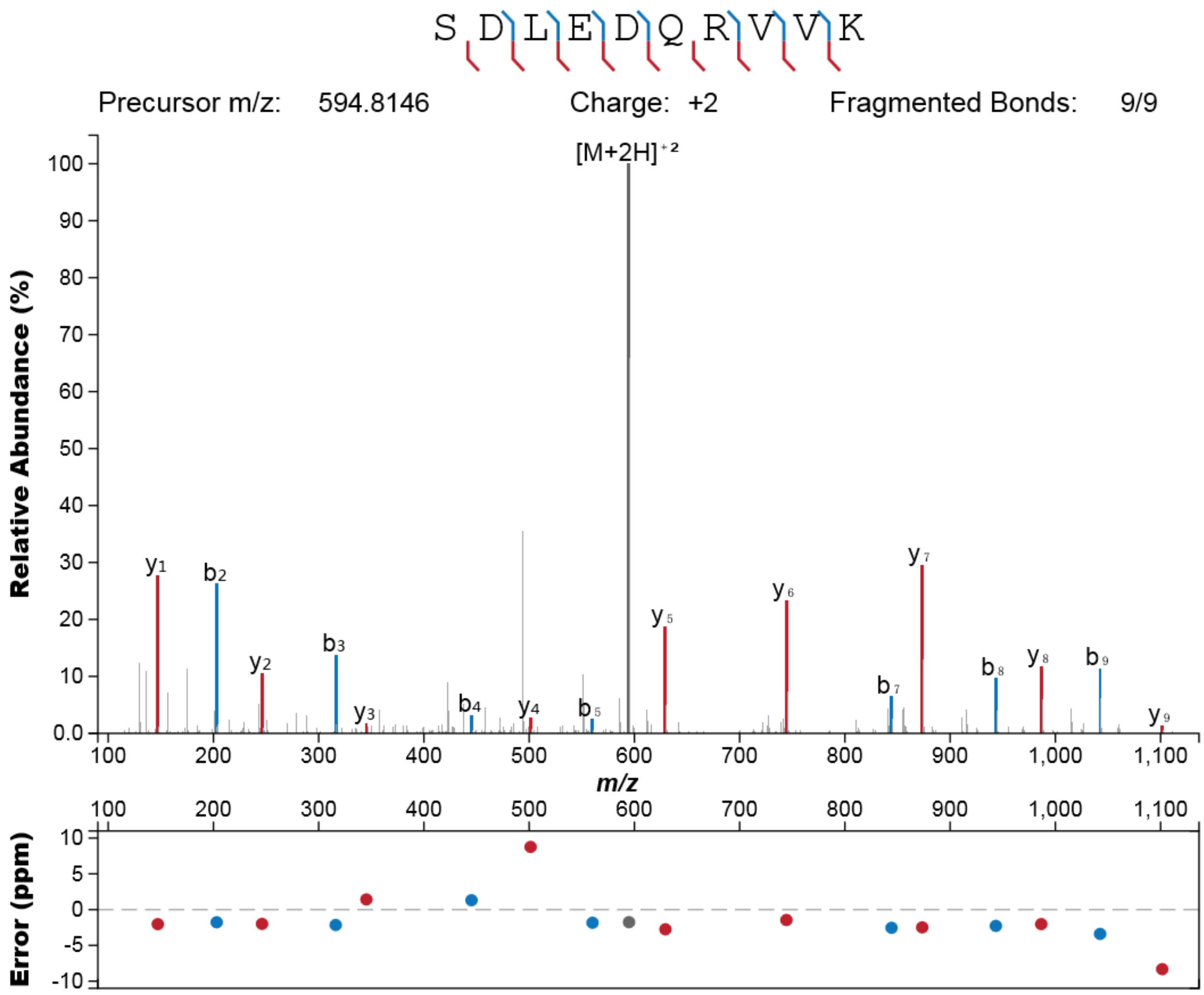
S D L E D Q R V V K
Precursor m/z: 594.8146
Charge: +2
Fragmented Bonds: 9/9
[M+2H]+2
Relative Abundance (%)
m/z
Error (ppm)
y1
b2
y2
b3
y3
b4
y4
b5
y5
y6
b7
y7
b8
y8
b9
y9

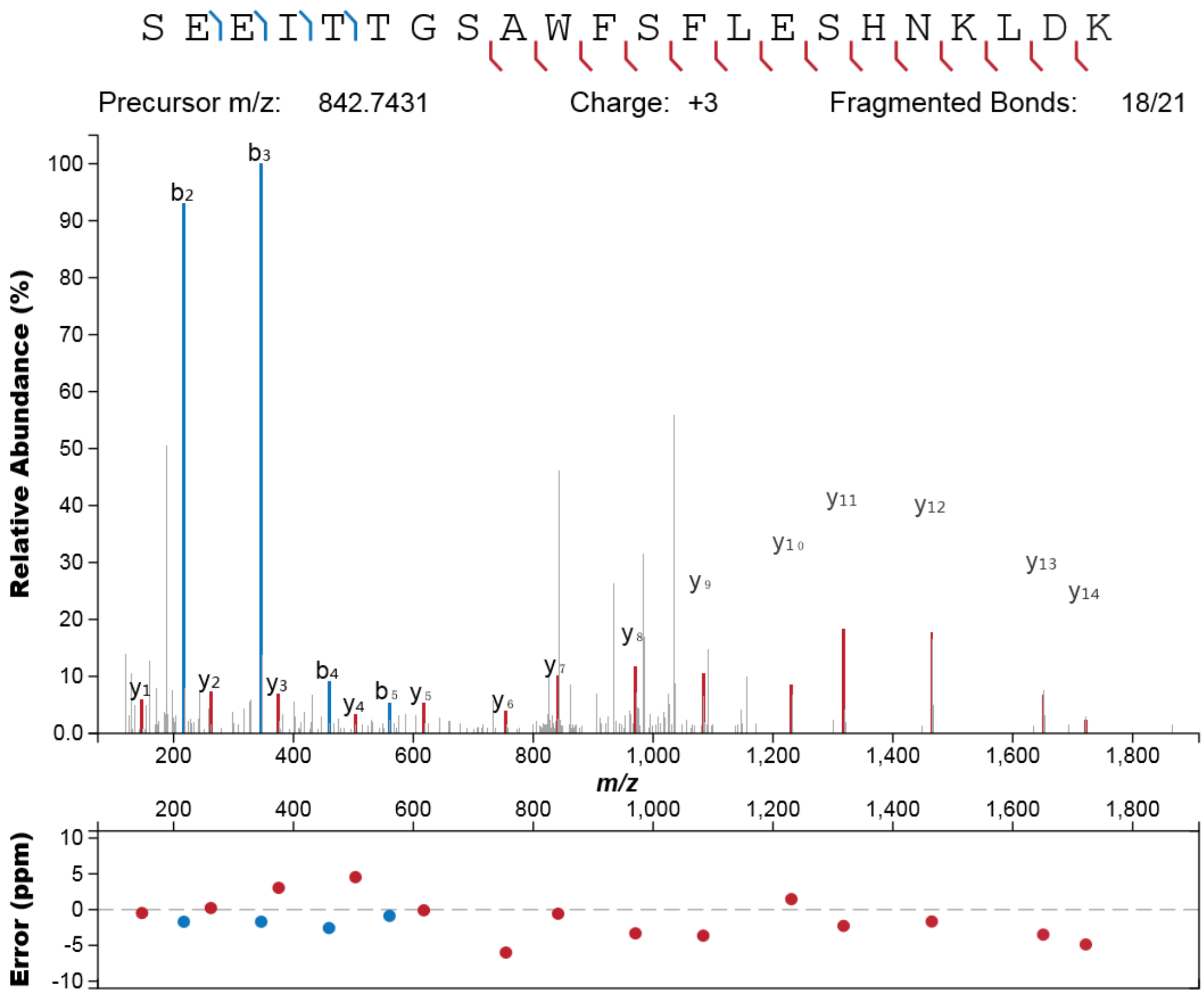
S E E I T T G S A W F S F L E S H N K L D K
Precursor m/z: 842.7431
Charge: +3
Fragmented Bonds: 18/21
Relative Abundance (%)
m/z
Error (ppm)
b2
b3
b4
b5
y1
y2
y3
y4
y5
y6
y7
y8
y9
y10
y11
y12
y13
y14

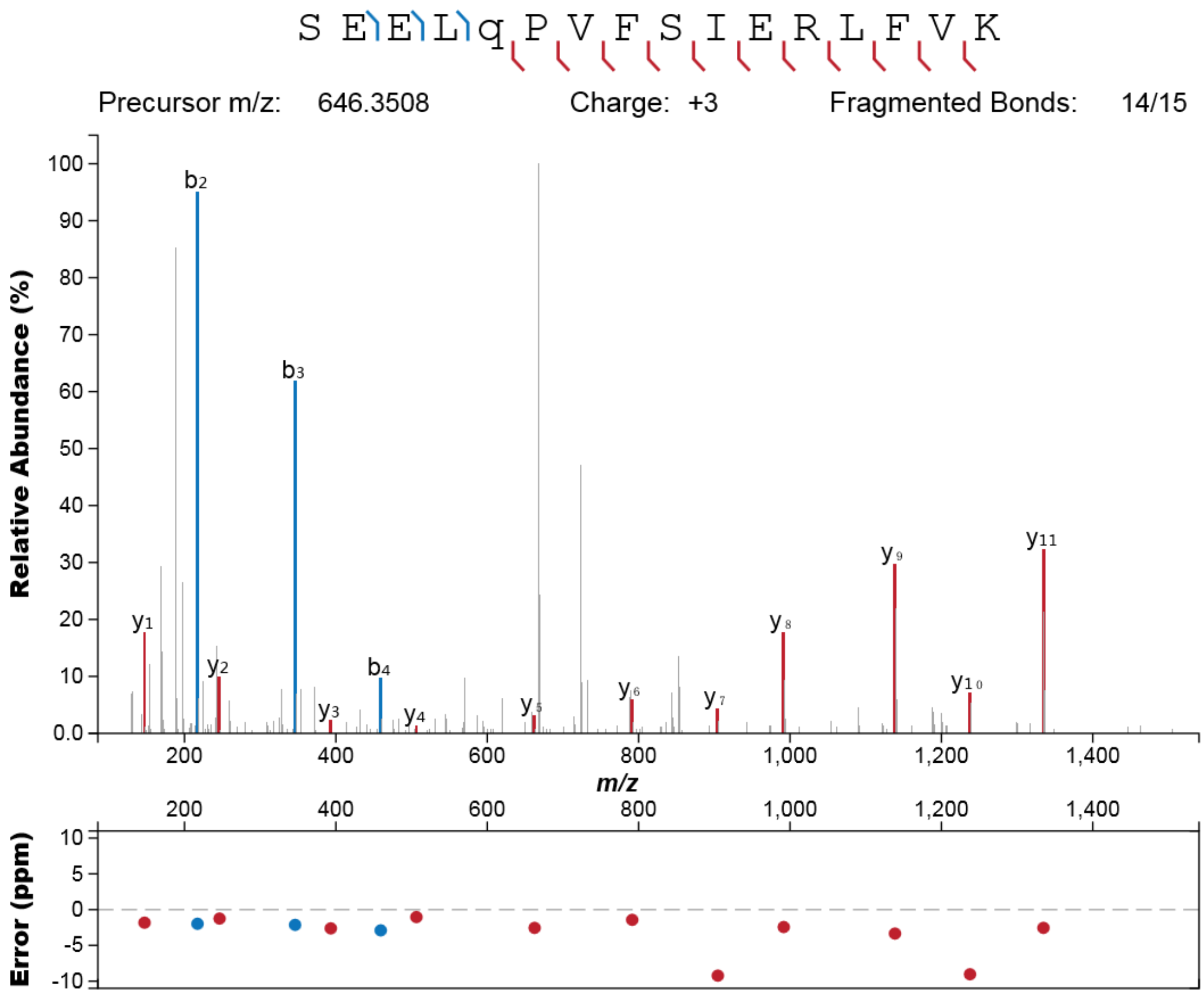
S E E L q P V F S I E R L F V K
Precursor m/z: 646.3508
Charge: +3
Fragmented Bonds: 14/15
Relative Abundance (%)
m/z
Error (ppm)
b2
b3
b4
y1
y2
y3
y4
y5
y6
y7
y8
y9
y10
y11

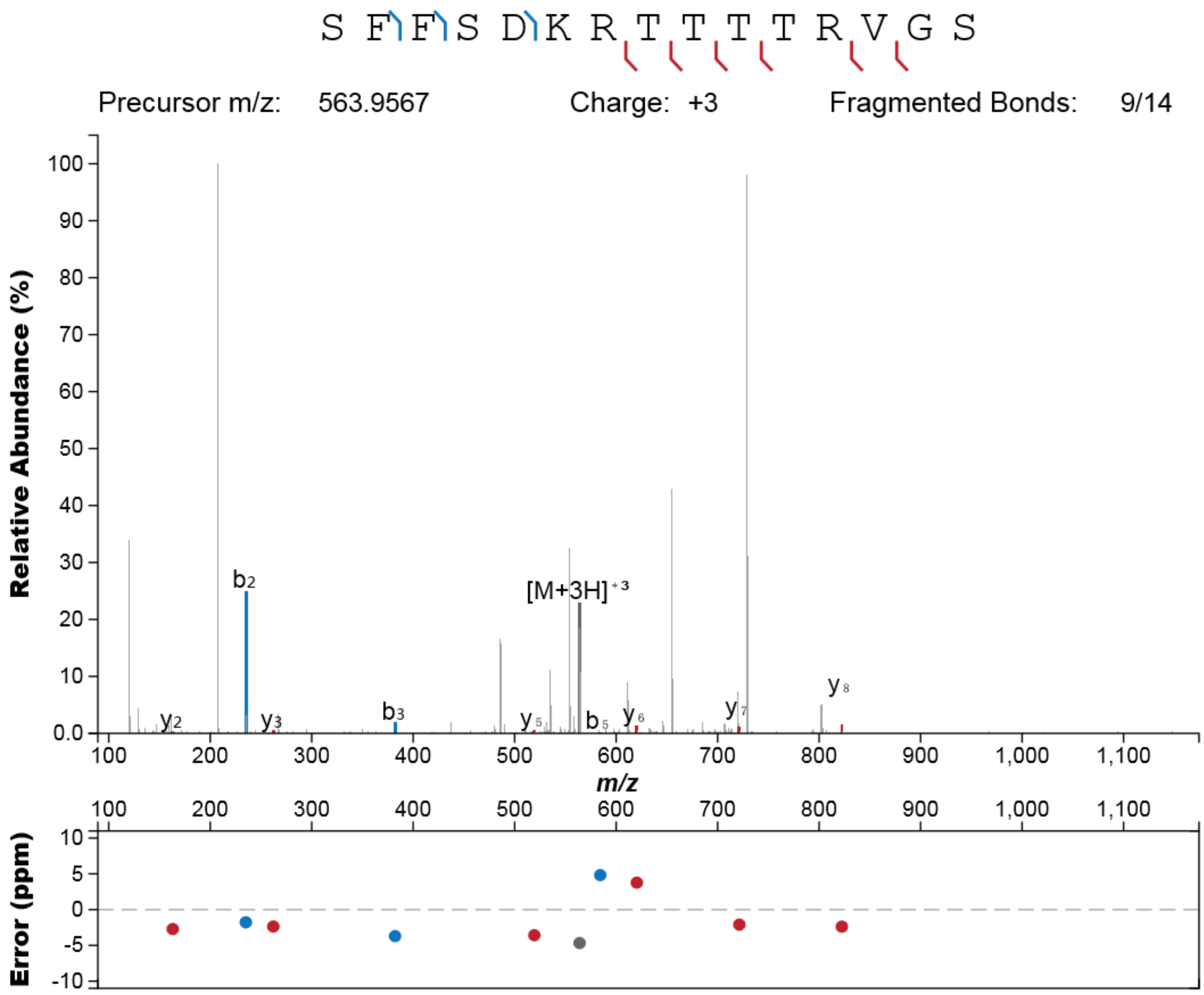
S F F S D K R T T T T R V G S
Precursor m/z: 563.9567
Charge: +3
Fragmented Bonds: 9/14
Relative Abundance (%)
b2
[M+3H]+3
y2
y3
b3
y5
b5
y6
y7
y8
m/z
Error (ppm)

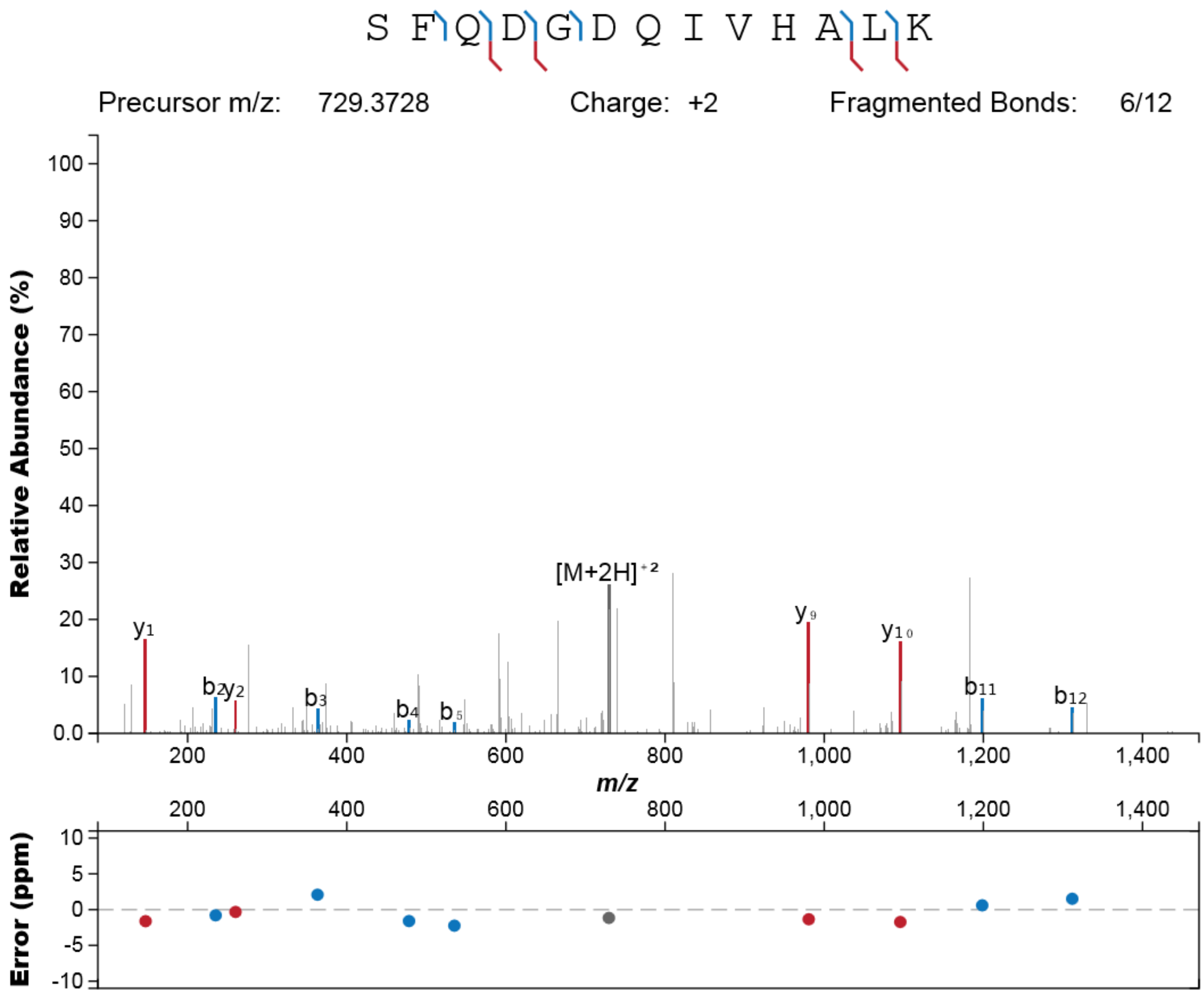

S F Q D G D Q I V H A L K
Precursor m/z: 729.3728
Charge: +2
Fragmented Bonds: 6/12
Relative Abundance (%)
[M+2H]+2
y1
b2
y2
b3
b4
b5
y9
y10
b11
b12
m/z
Error (ppm)

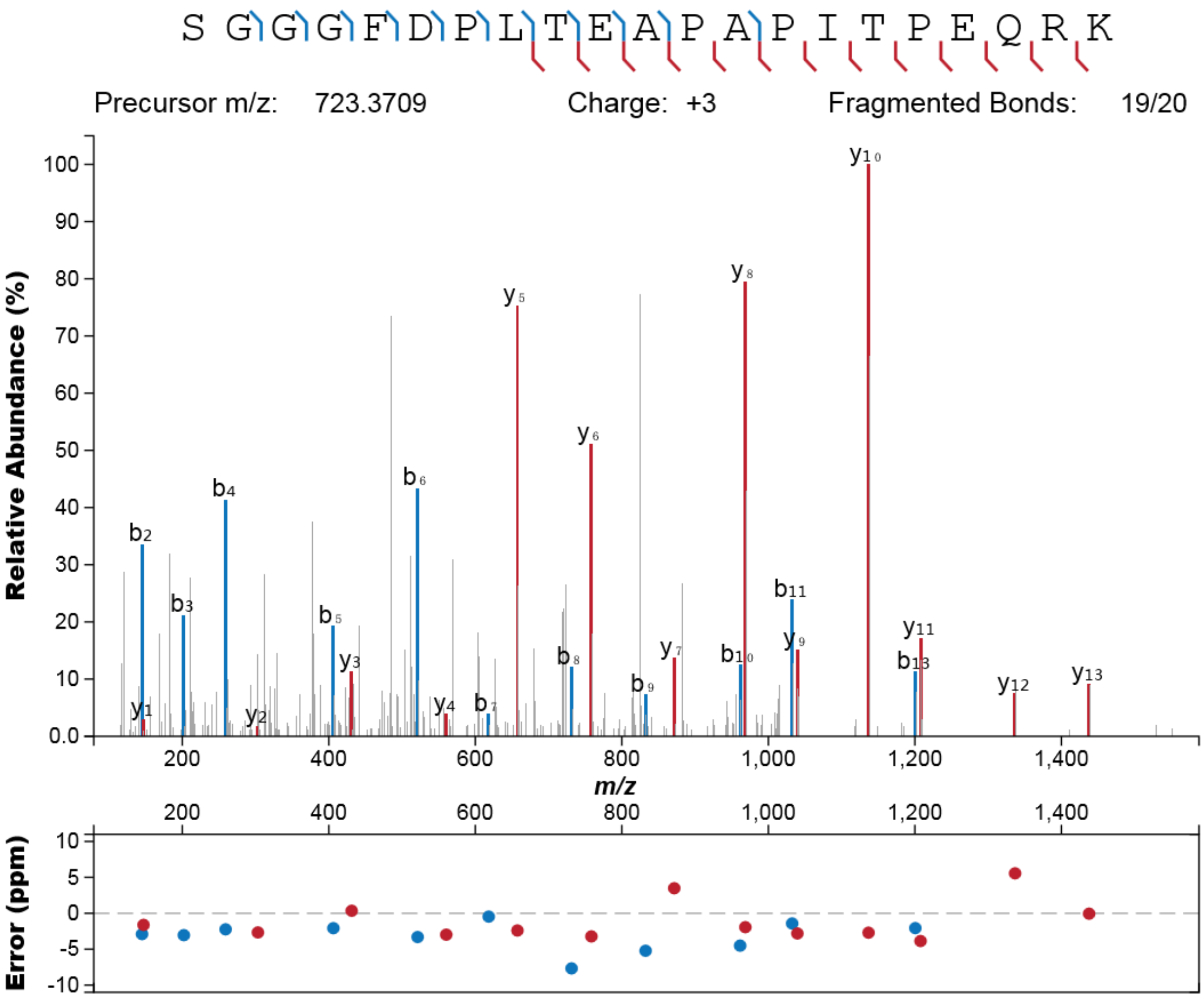

S G G G F D P L T E A P A P I T P E Q R K
Precursor m/z: 723.3709
Charge: +3
Fragmented Bonds: 19/20
Relative Abundance (%)
m/z
Error (ppm)

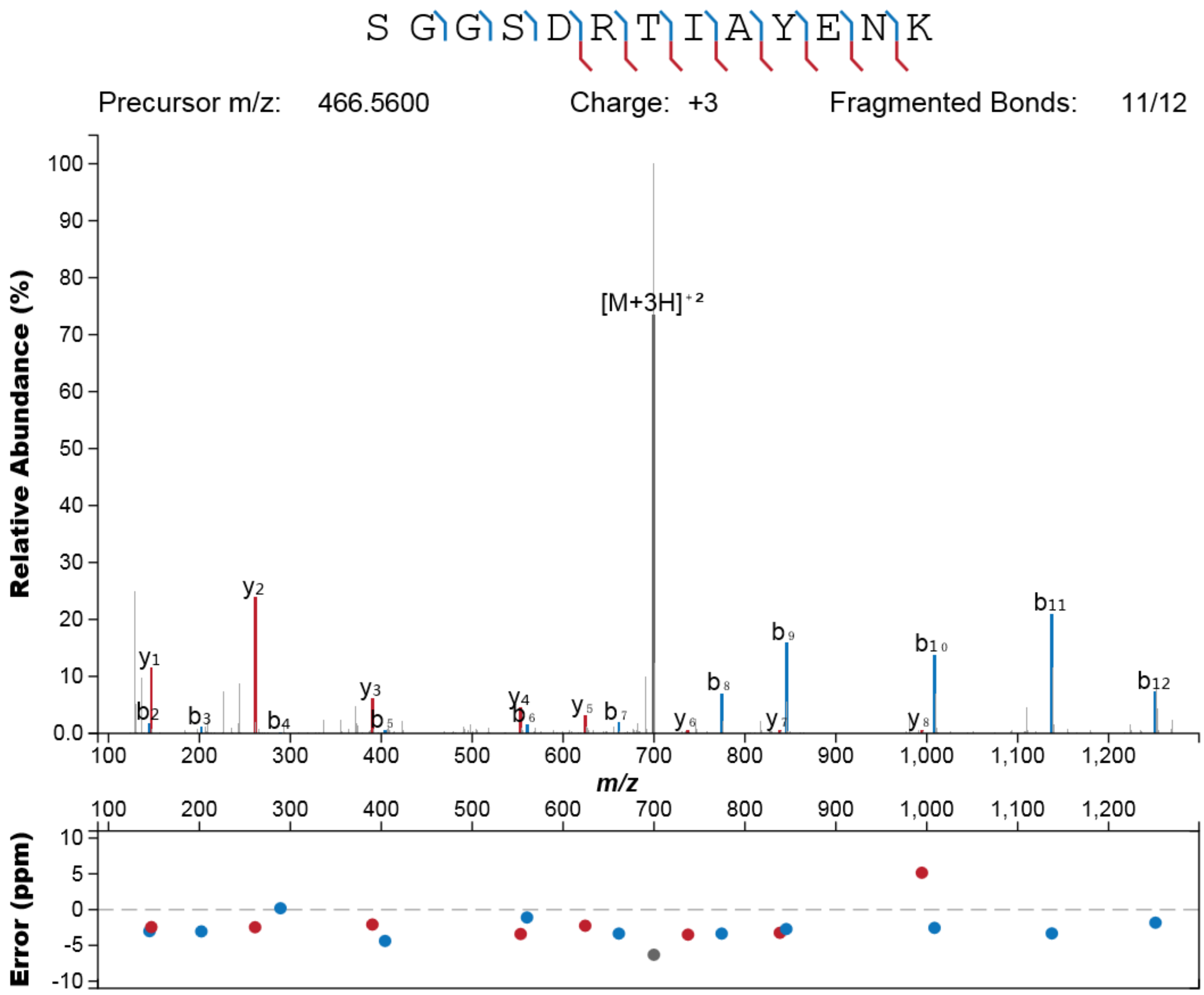

S G G S D R T I A Y E N K
Precursor m/z: 466.5600
Charge: +3
Fragmented Bonds: 11/12
Relative Abundance (%)
[M+3H]+2
y1
y2
y3
y4
y5
y6
y7
y8
b2
b3
b4
b5
b6
b7
b8
b9
b10
b11
b12
m/z
Error (ppm)

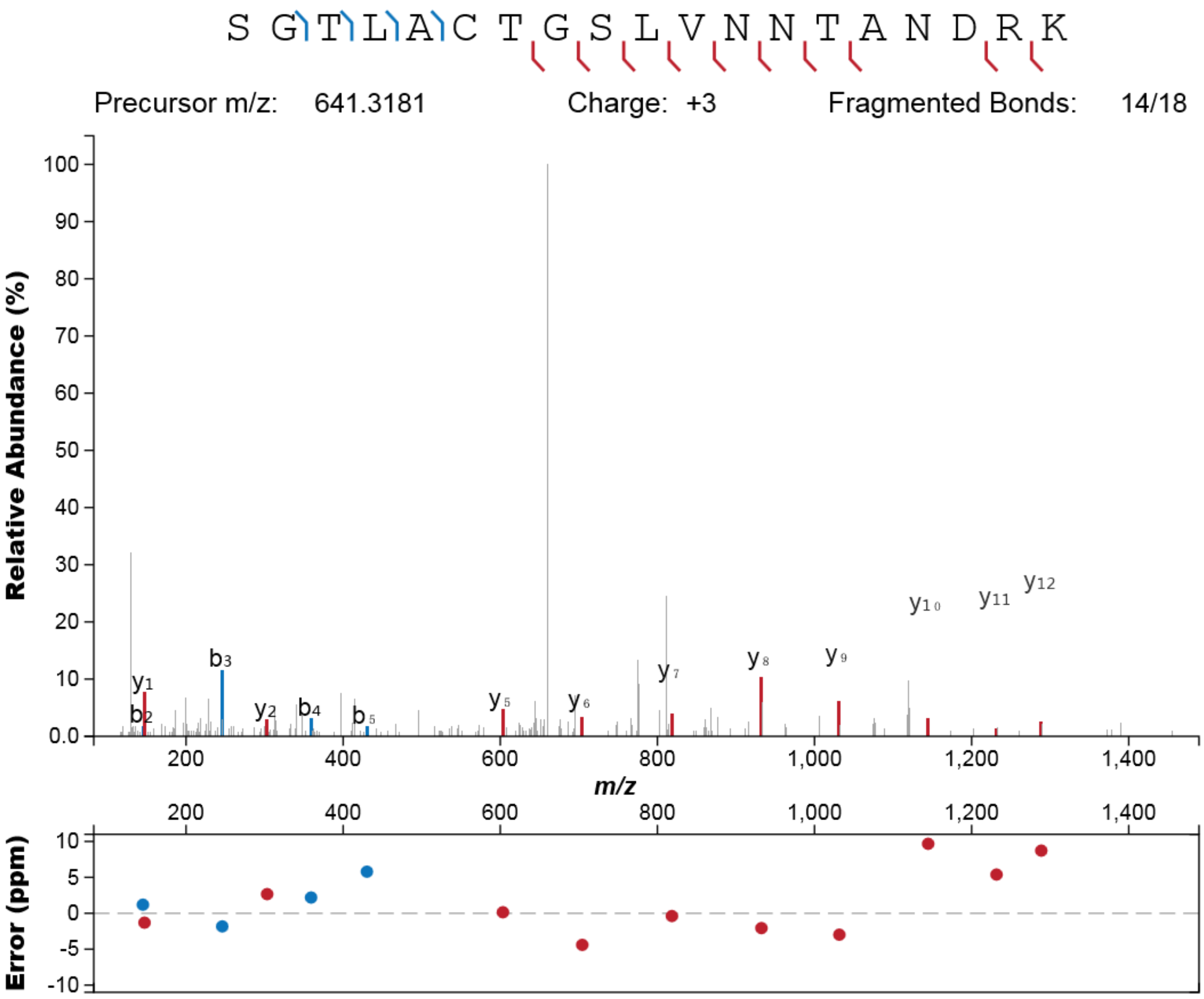

S G T L A C T G S L V N N T A N D R K
Precursor m/z: 641.3181
Charge: +3
Fragmented Bonds: 14/18
Relative Abundance (%)
m/z
Error (ppm)

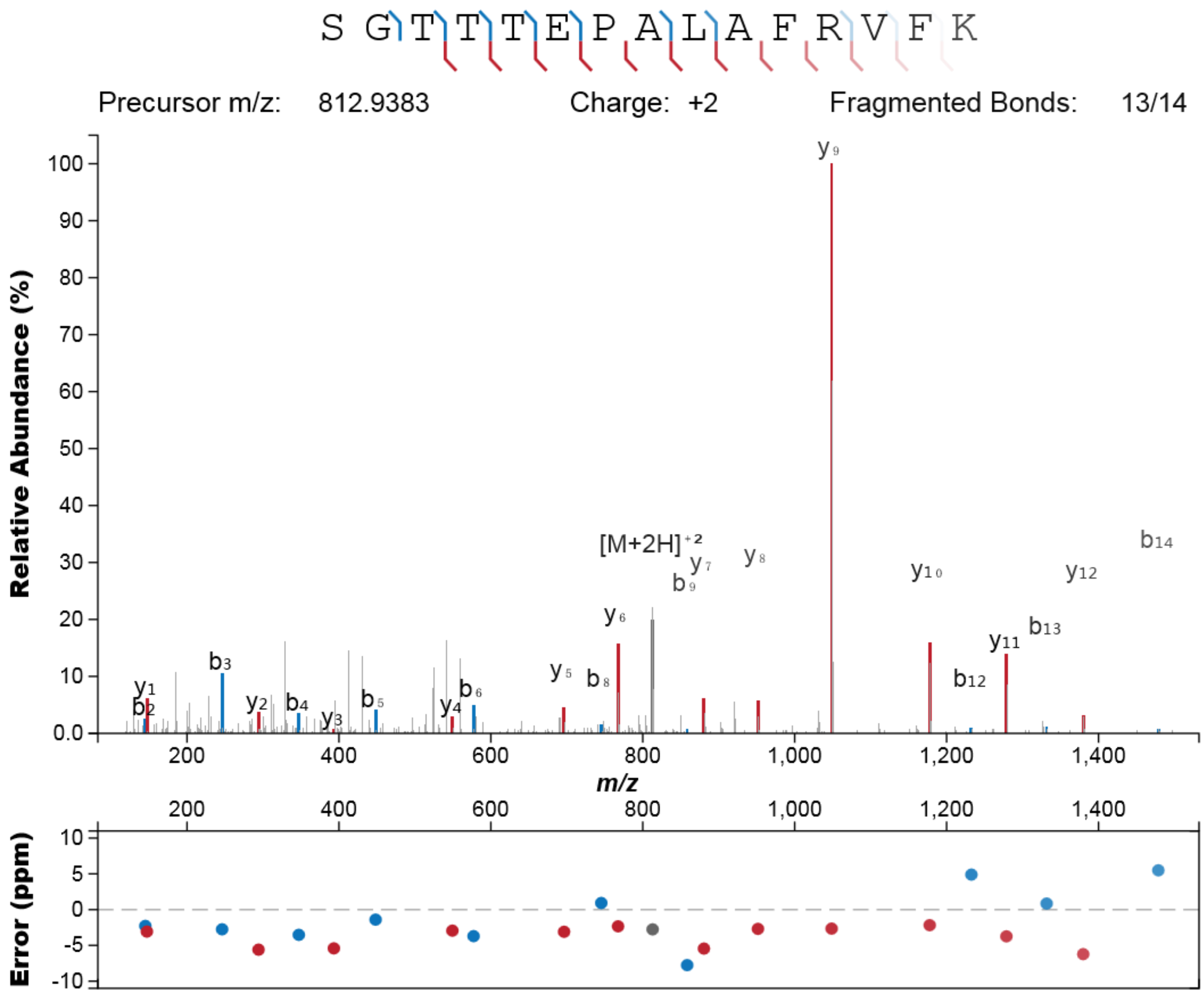

S G T T T E P A L A F R V F K
Precursor m/z: 812.9383
Charge: +2
Fragmented Bonds: 13/14
Relative Abundance (%)
m/z
Error (ppm)
[M+2H]+2
y1
b2
b3
y2
b4
y3
b5
y4
b6
y5
b8
y6
b9
y7
y8
y9
y10
b12
y11
b13
y12
b14

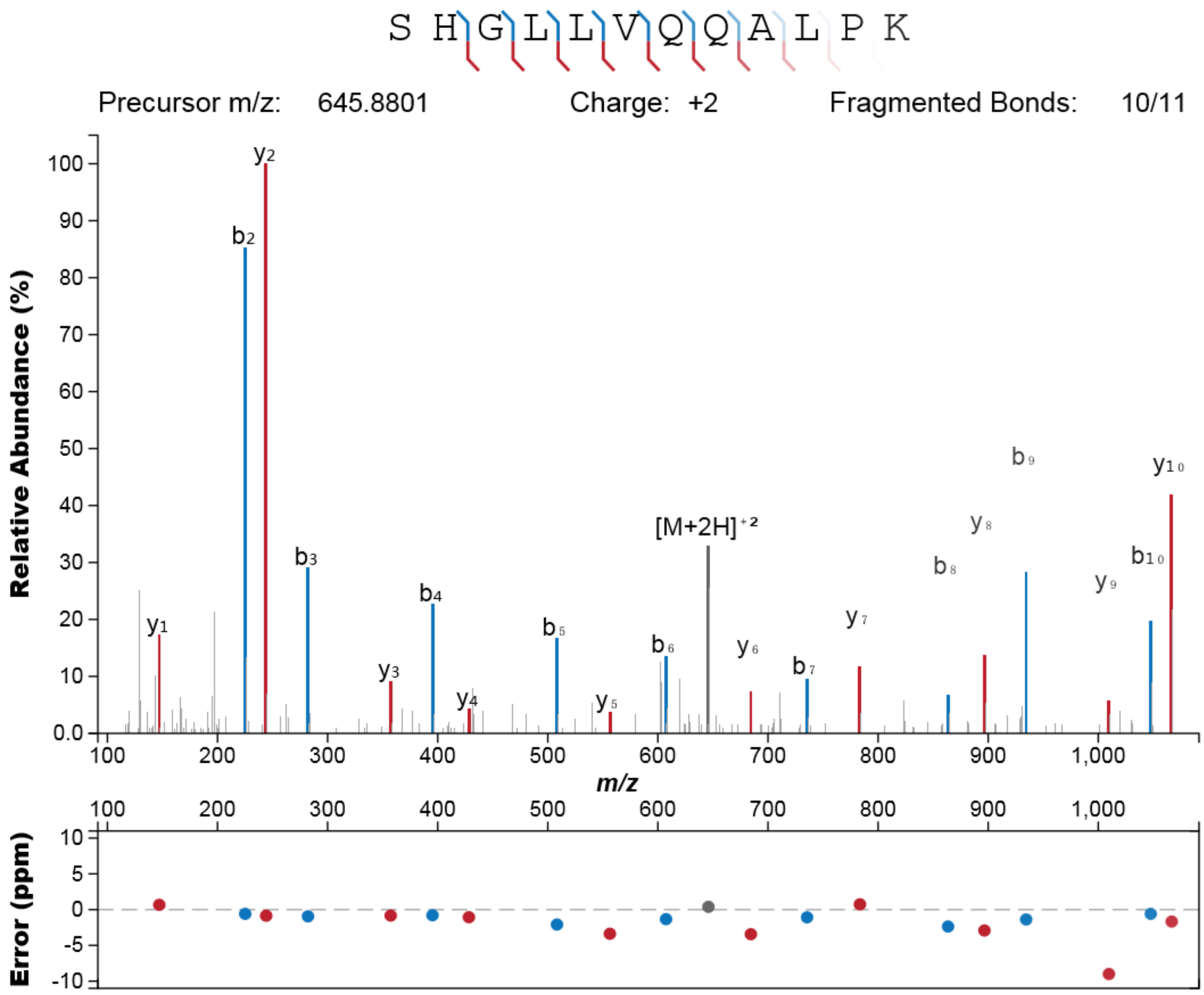
S H G L L V Q Q A L P K
Precursor m/z: 645.8801
Charge: +2
Fragmented Bonds: 10/11
Relative Abundance (%)
m/z
Error (ppm)
[M+2H]+2
y1 y2 y3 y4 y5 y6 y7 y8 y9 y10
b2 b3 b4 b5 b6 b7 b8 b9 b10

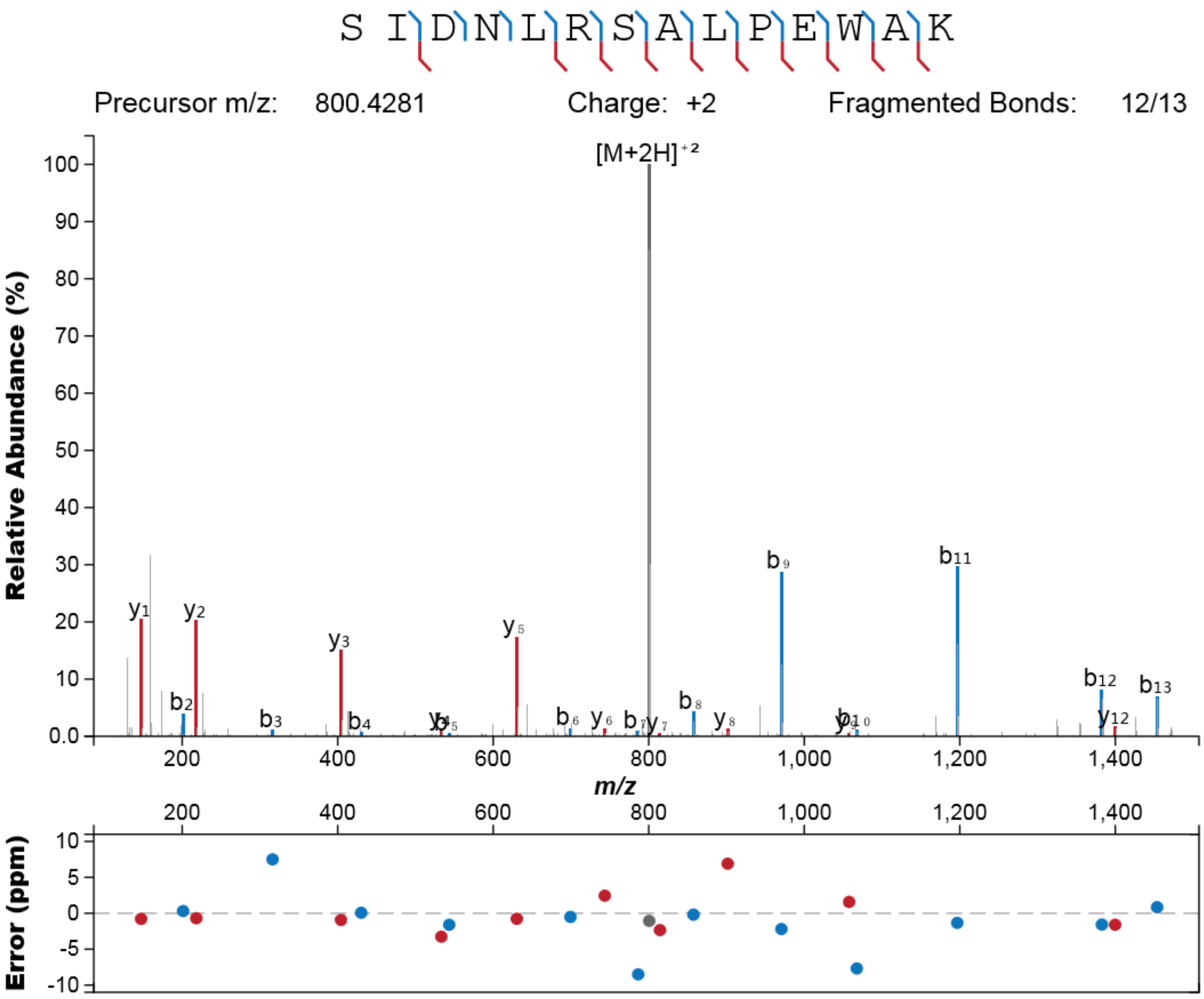
S I D N L R S A L P E W A K
Precursor m/z: 800.4281
Charge: +2
Fragmented Bonds: 12/13
[M+2H]+2
Relative Abundance (%)
m/z
Error (ppm)
y1
y2
y3
y5
b2
b3
b4
b6
b8
b9
b11
b12
b13
y12
0.0
10
20
30
40
50
60
70
80
90
100
200
400
600
800
1,000
1,200
1,400
-10
-5
0
5
10

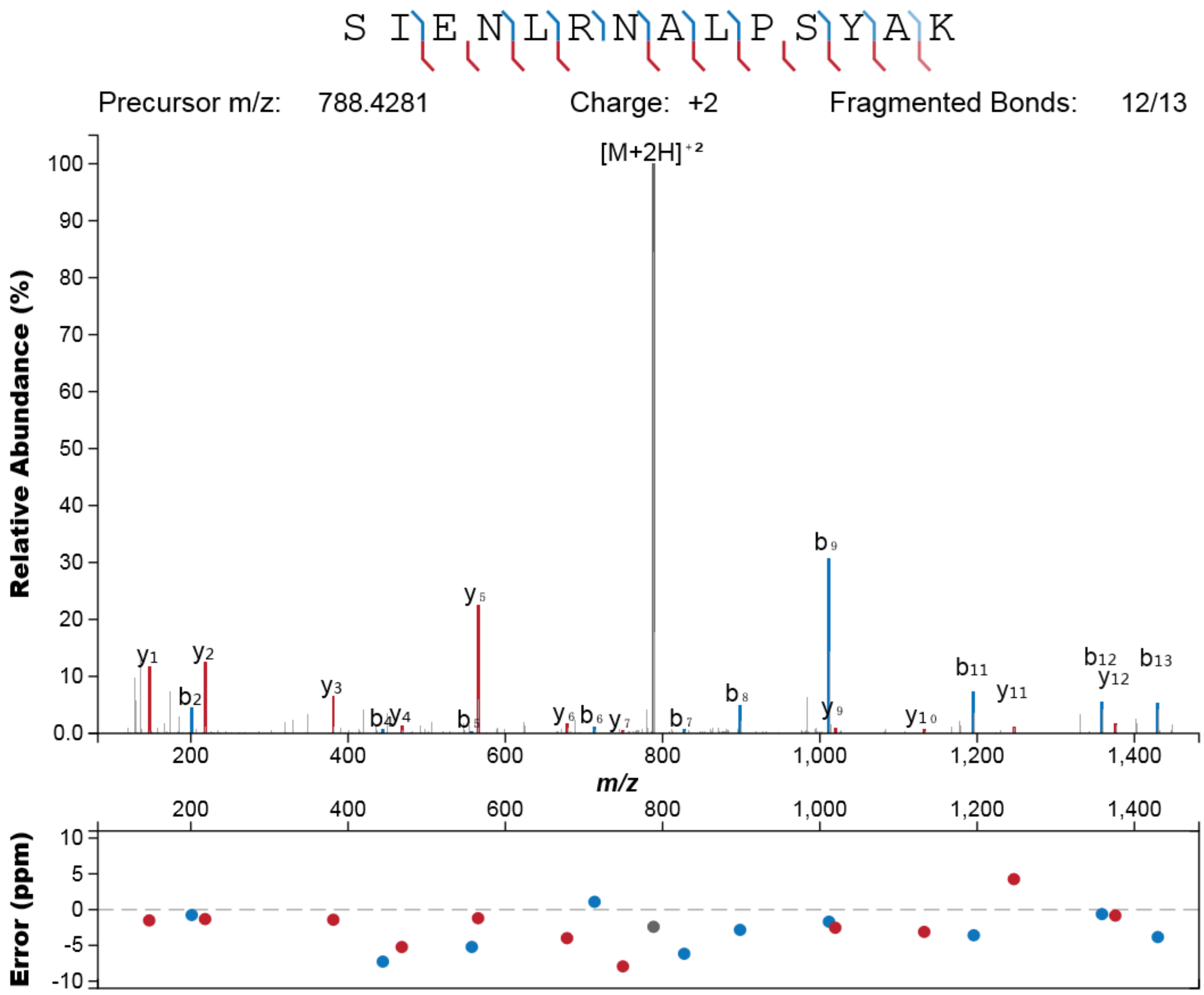
S I E N L R N A L P S Y A K
Precursor m/z: 788.4281
Charge: +2
Fragmented Bonds: 12/13
[M+2H]+2
Relative Abundance (%)
m/z
Error (ppm)
y1
y2
b2
y3
b4
y4
b5
y5
y6
b6
y7
b7
b8
b9
y9
y10
b11
y11
b12
y12
b13

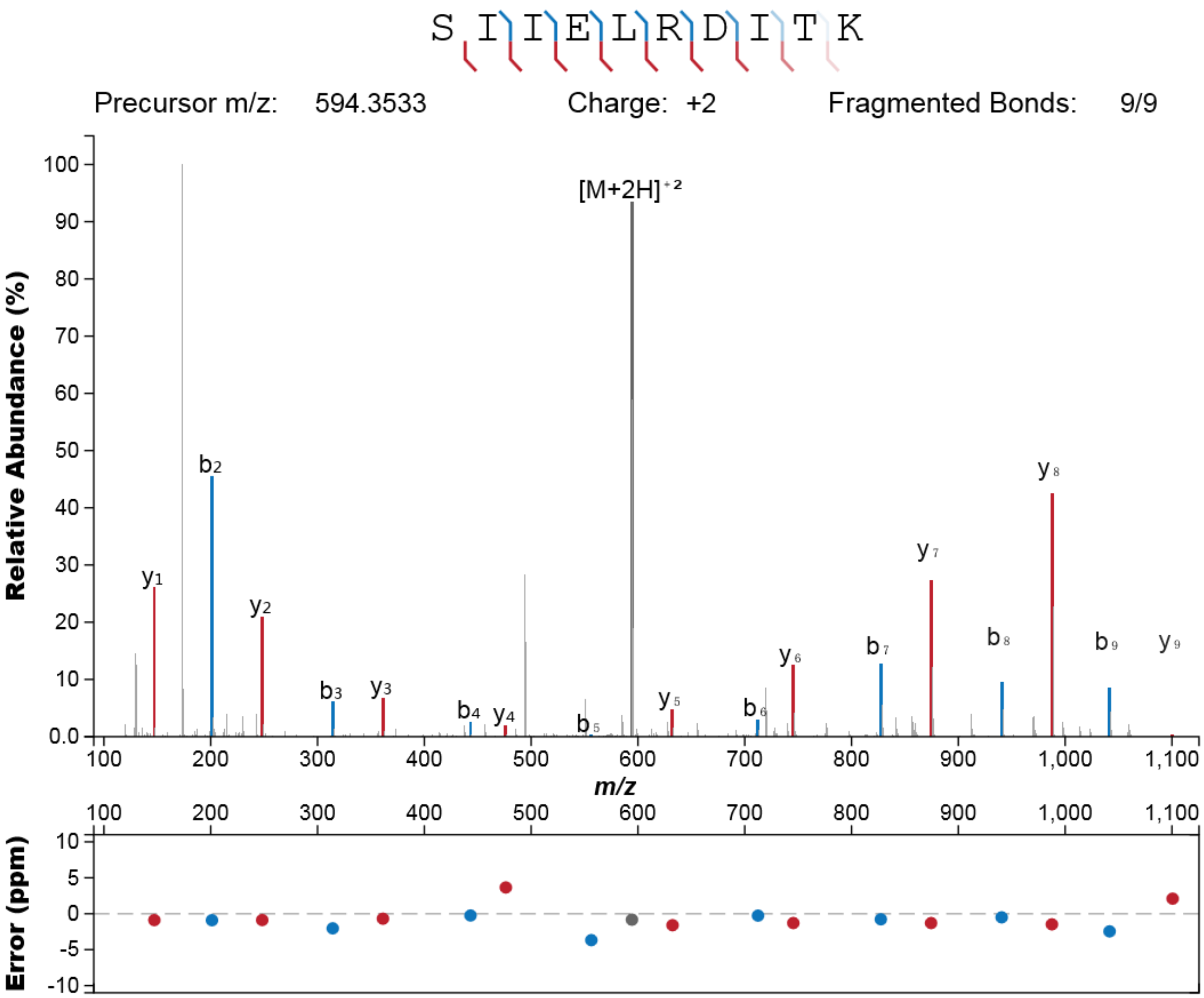

S I I E L R D I T K
Precursor m/z: 594.3533
Charge: +2
Fragmented Bonds: 9/9
Relative Abundance (%)
[M+2H]+2
y1
b2
y2
b3
y3
b4
y4
b5
y5
b6
y6
b7
y7
b8
y8
b9
y9
m/z
Error (ppm)

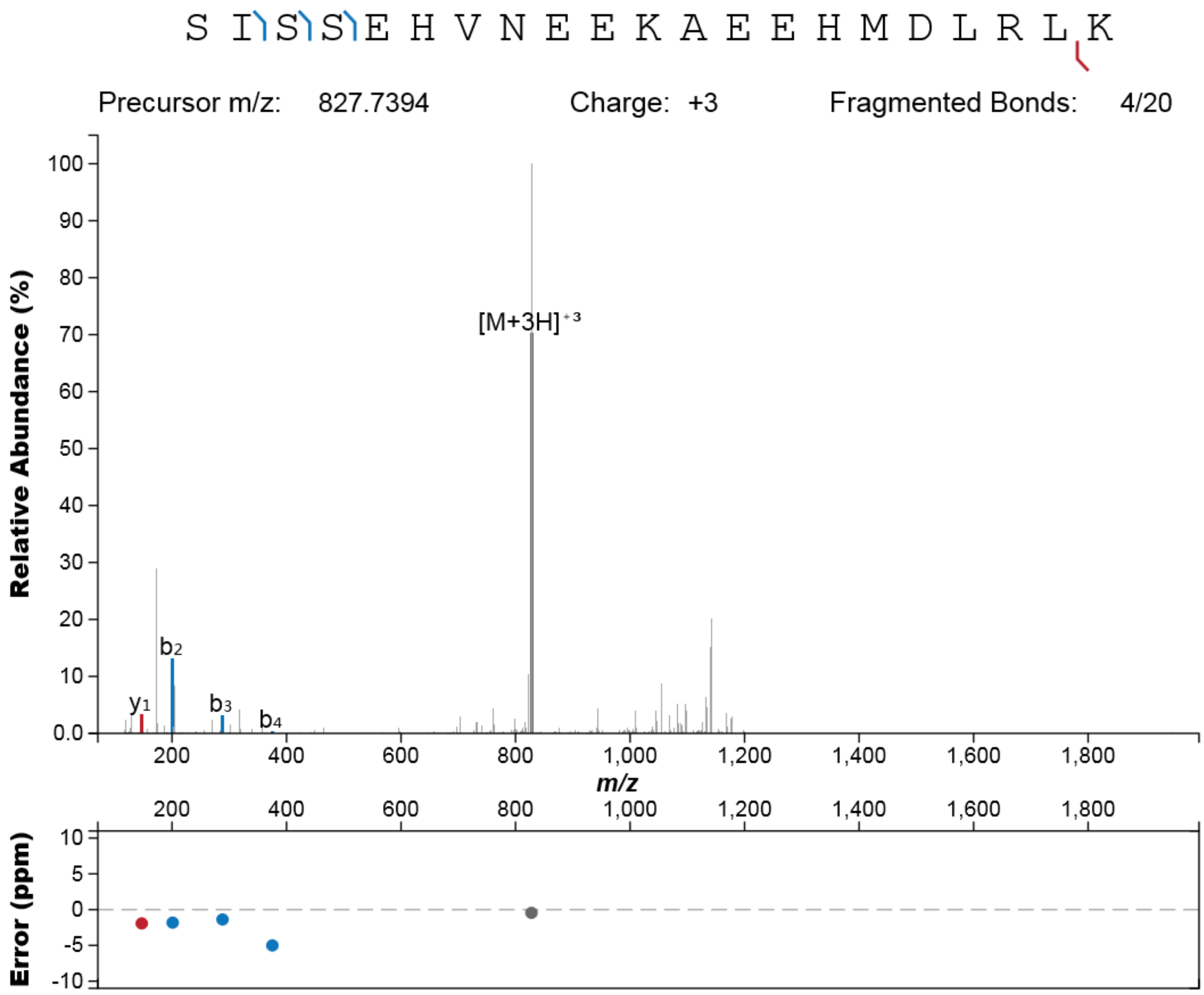
S I S S E H V N E E K A E E H M D L R L K
Precursor m/z: 827.7394
Charge: +3
Fragmented Bonds: 4/20
Relative Abundance (%)
[M+3H]+3
y1
b2
b3
b4
m/z
Error (ppm)

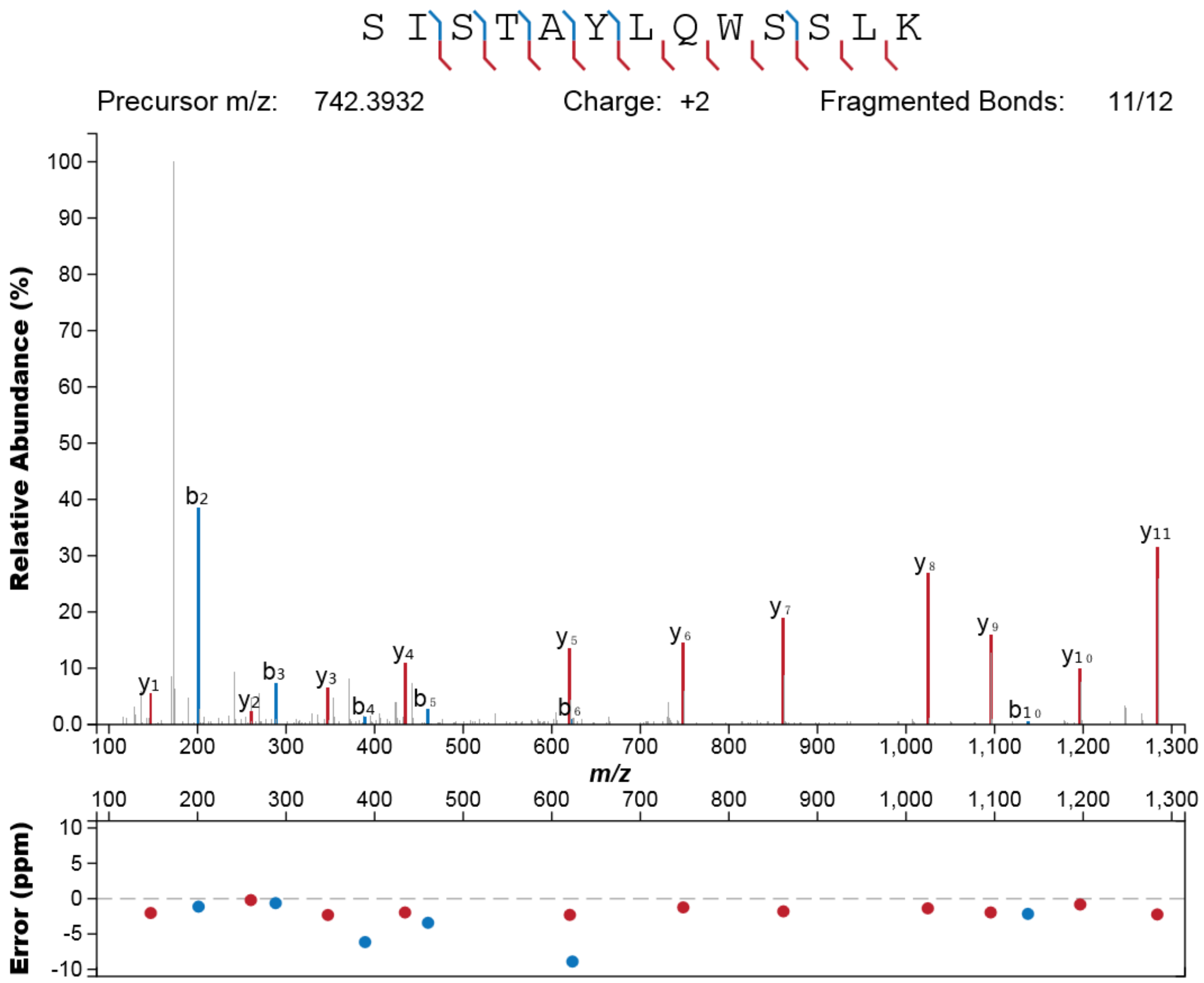
S I S T A Y L Q W S S L K
Precursor m/z: 742.3932
Charge: +2
Fragmented Bonds: 11/12
Relative Abundance (%)
m/z
Error (ppm)
b2
b3
b4
b5
b6
b10
y1
y2
y3
y4
y5
y6
y7
y8
y9
y10
y11

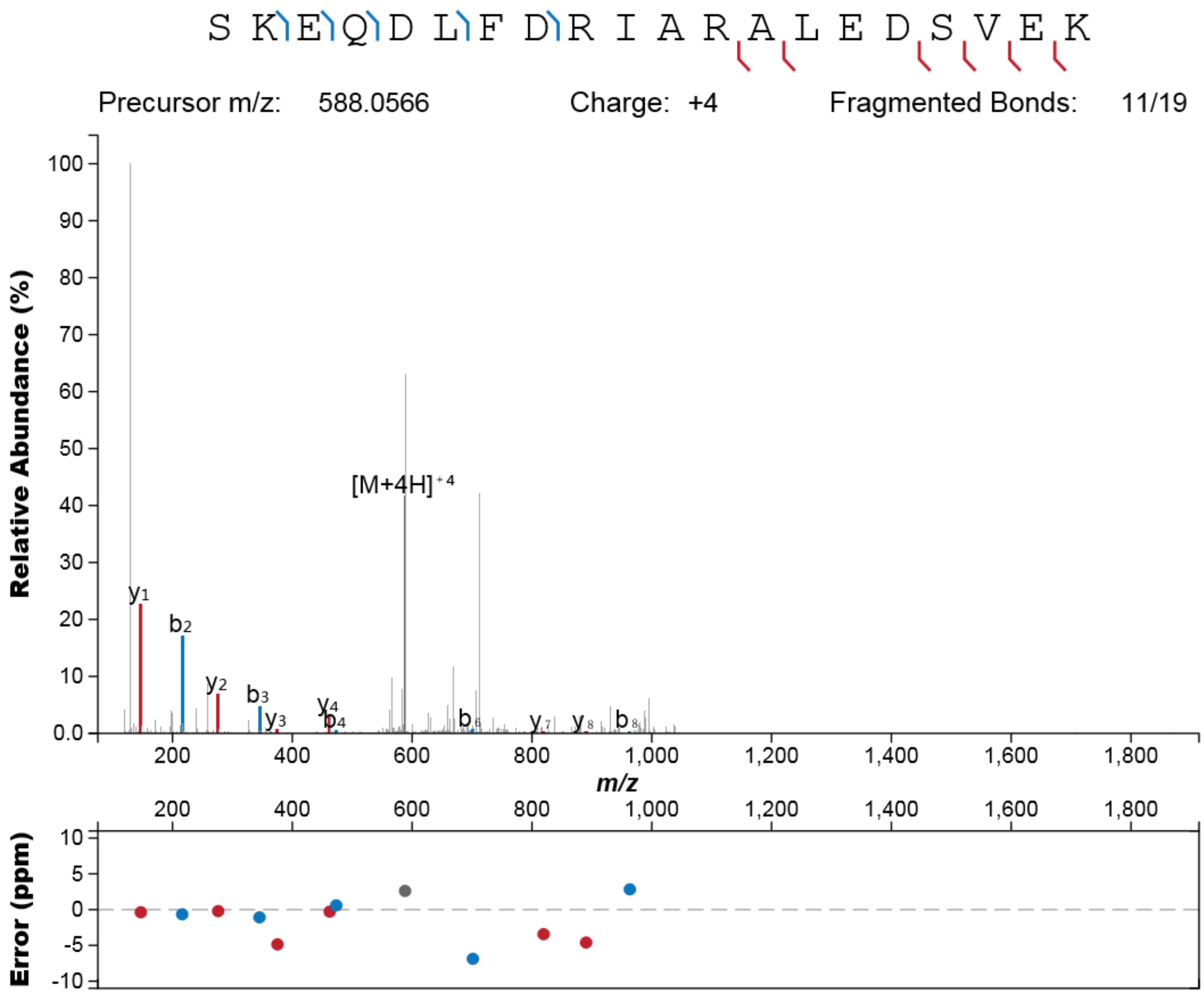

S K E Q D L F D R I A R A L E D S V E K
Precursor m/z: 588.0566
Charge: +4
Fragmented Bonds: 11/19
Relative Abundance (%)
[M+4H]+4
y1
b2
y2
b3
y3
y4
b4
b6
y7
y8
b8
m/z
Error (ppm)

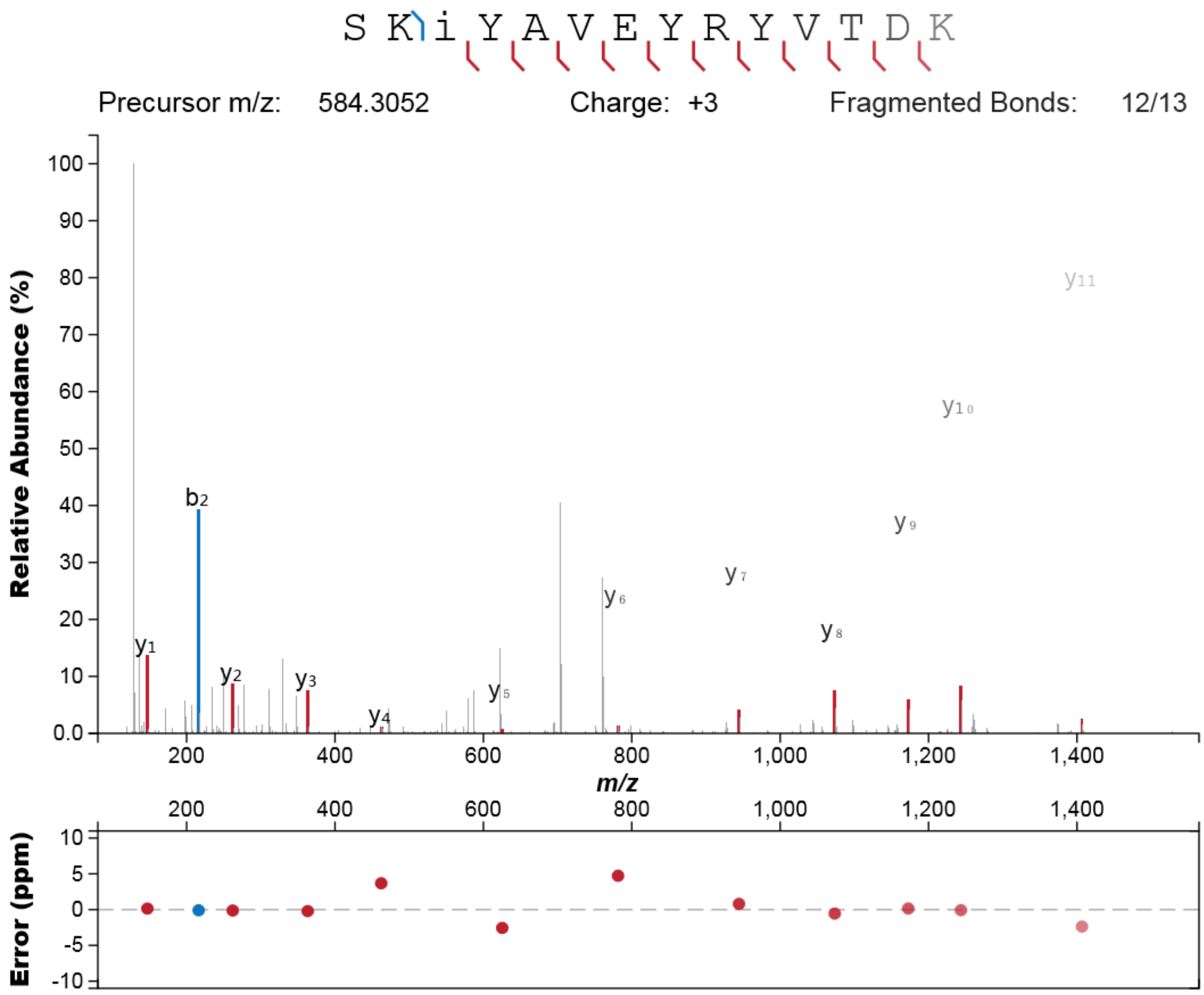

S K i Y A V E Y R Y V T D K
Precursor m/z: 584.3052
Charge: +3
Fragmented Bonds: 12/13
Relative Abundance (%)
m/z
Error (ppm)
b2
y1
y2
y3
y4
y5
y6
y7
y8
y9
y10
y11

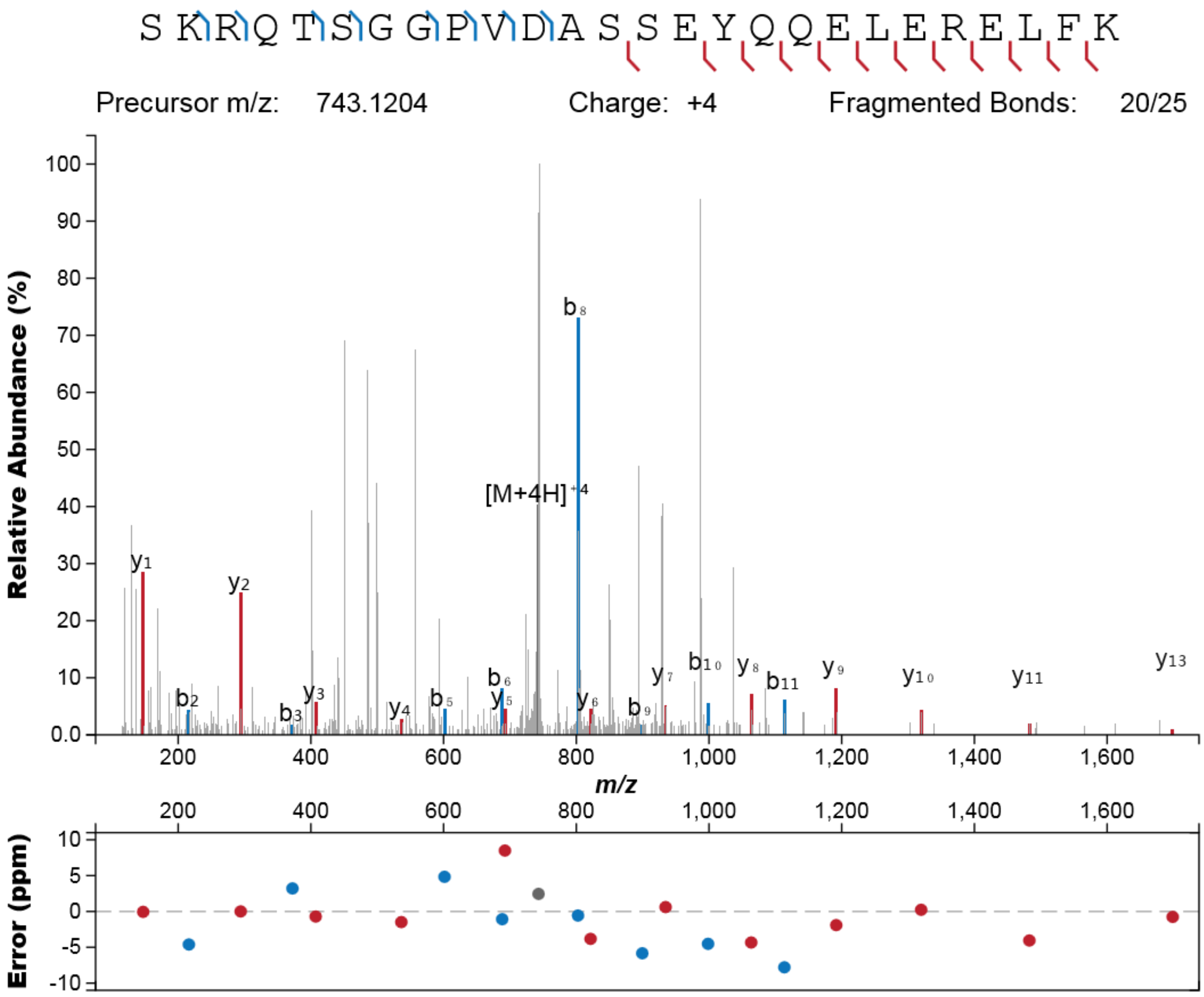

S K R Q T S G G P V D A S S E Y Q Q E L E R E L F K
Precursor m/z: 743.1204
Charge: +4
Fragmented Bonds: 20/25
Relative Abundance (%)
m/z
[M+4H]+4
Error (ppm)

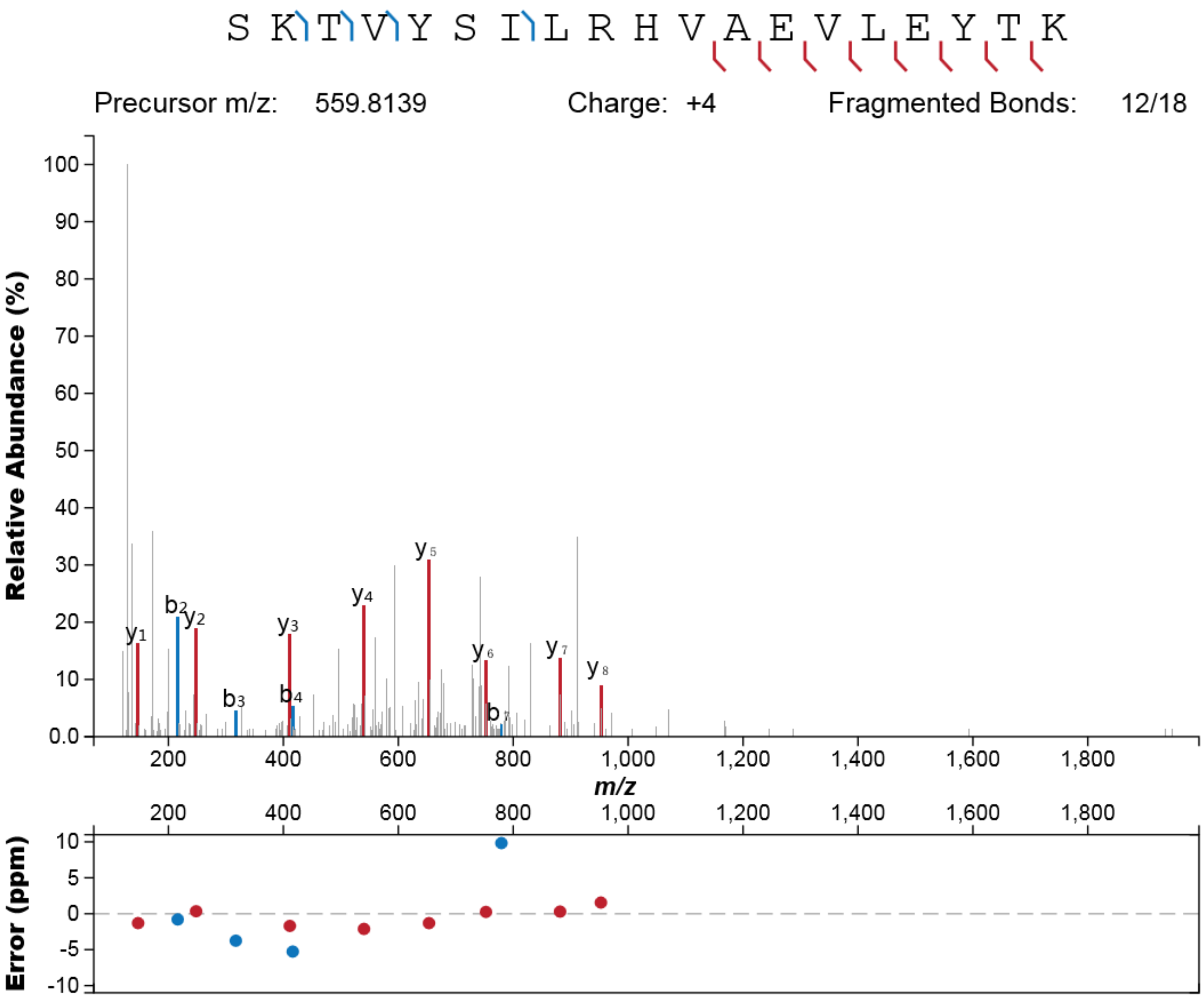

S K T V Y S I L R H V A E V L E Y T K
Precursor m/z: 559.8139
Charge: +4
Fragmented Bonds: 12/18
Relative Abundance (%)
m/z
Error (ppm)

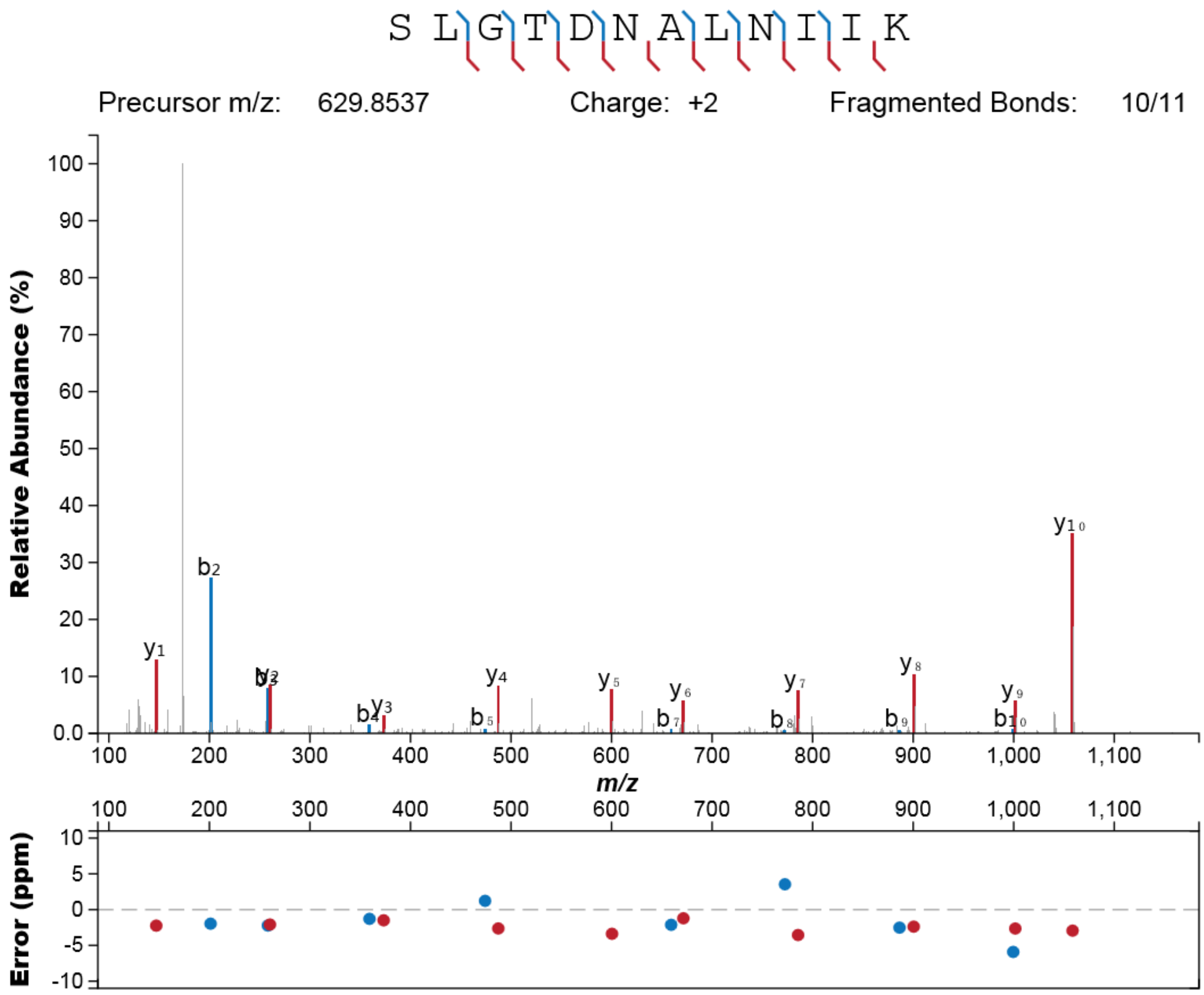

S L G T D N A L N I I K
Precursor m/z: 629.8537
Charge: +2
Fragmented Bonds: 10/11
Relative Abundance (%)
m/z
Error (ppm)

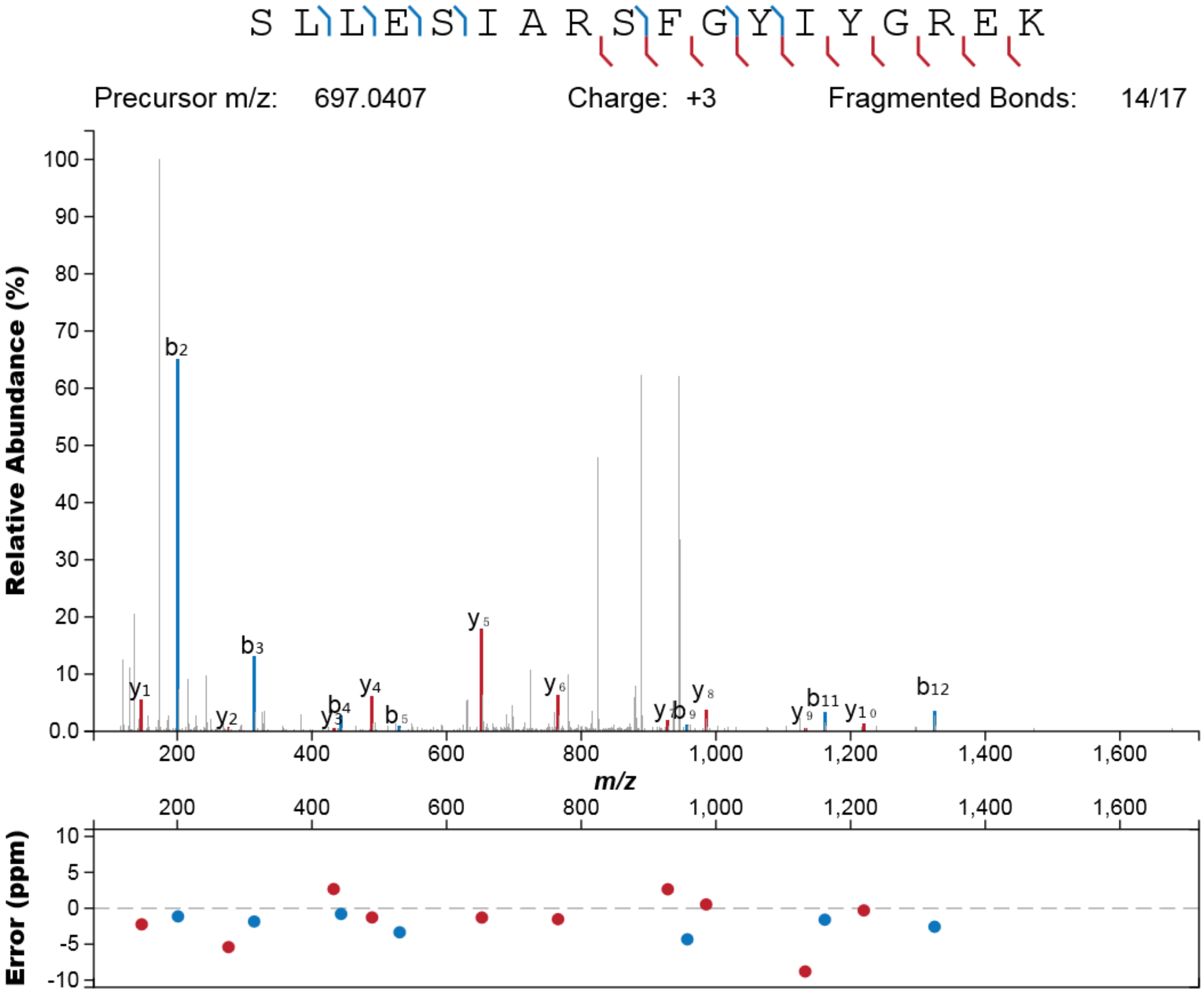
S L L E S I A R S F G Y I Y G R E K
Precursor m/z: 697.0407
Charge: +3
Fragmented Bonds: 14/17
Relative Abundance (%)
m/z
Error (ppm)
b2
b3
b4
b5
b9
b11
b12
y1
y2
y3
y4
y5
y6
y8
y9
y10

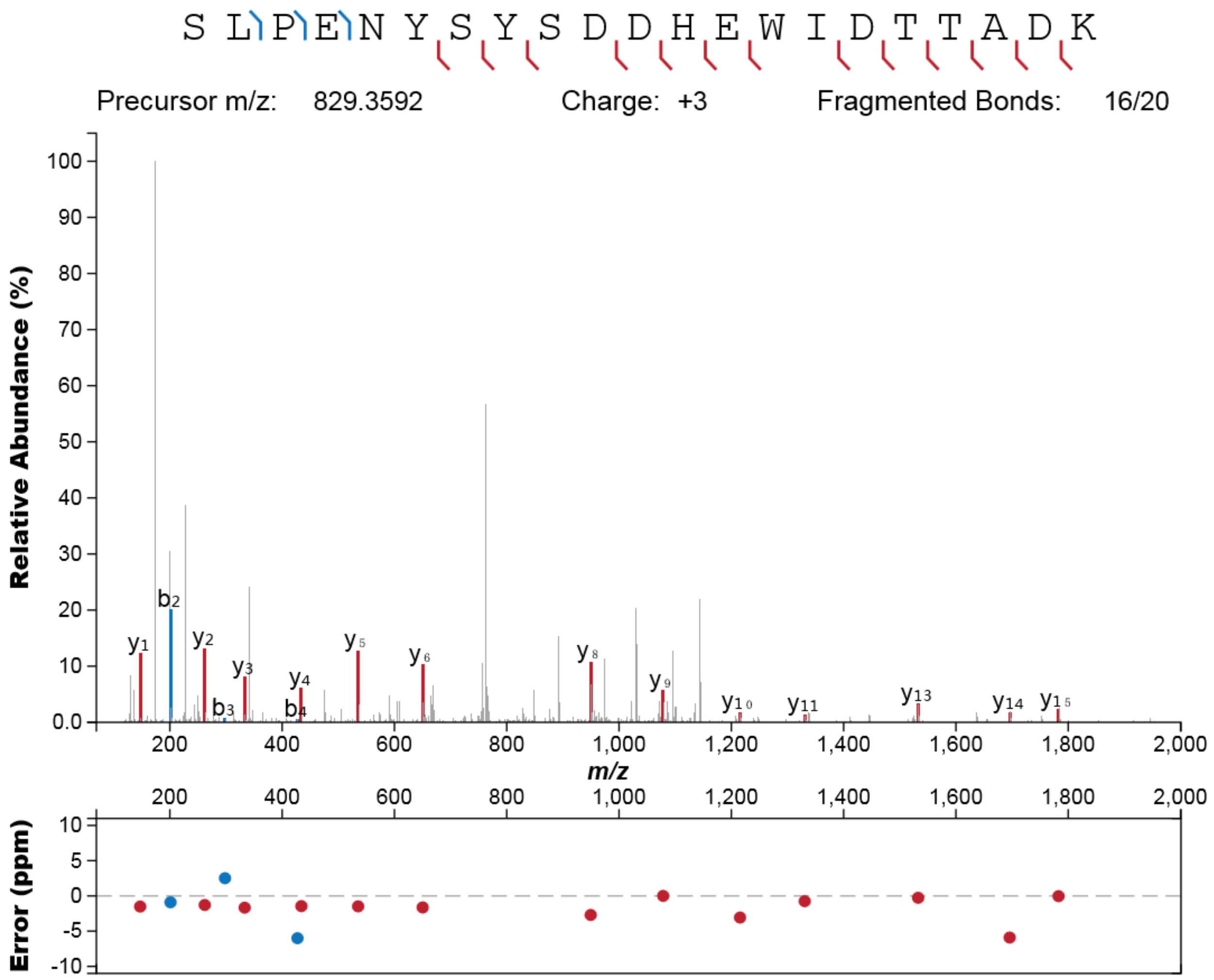

S L P E N Y S Y S D D H E W I D T T A D K
Precursor m/z: 829.3592
Charge: +3
Fragmented Bonds: 16/20
Relative Abundance (%)
m/z
Error (ppm)
y1
b2
y2
b3
y3
y4
b4
y5
y6
y8
y9
y10
y11
y13
y14
y15

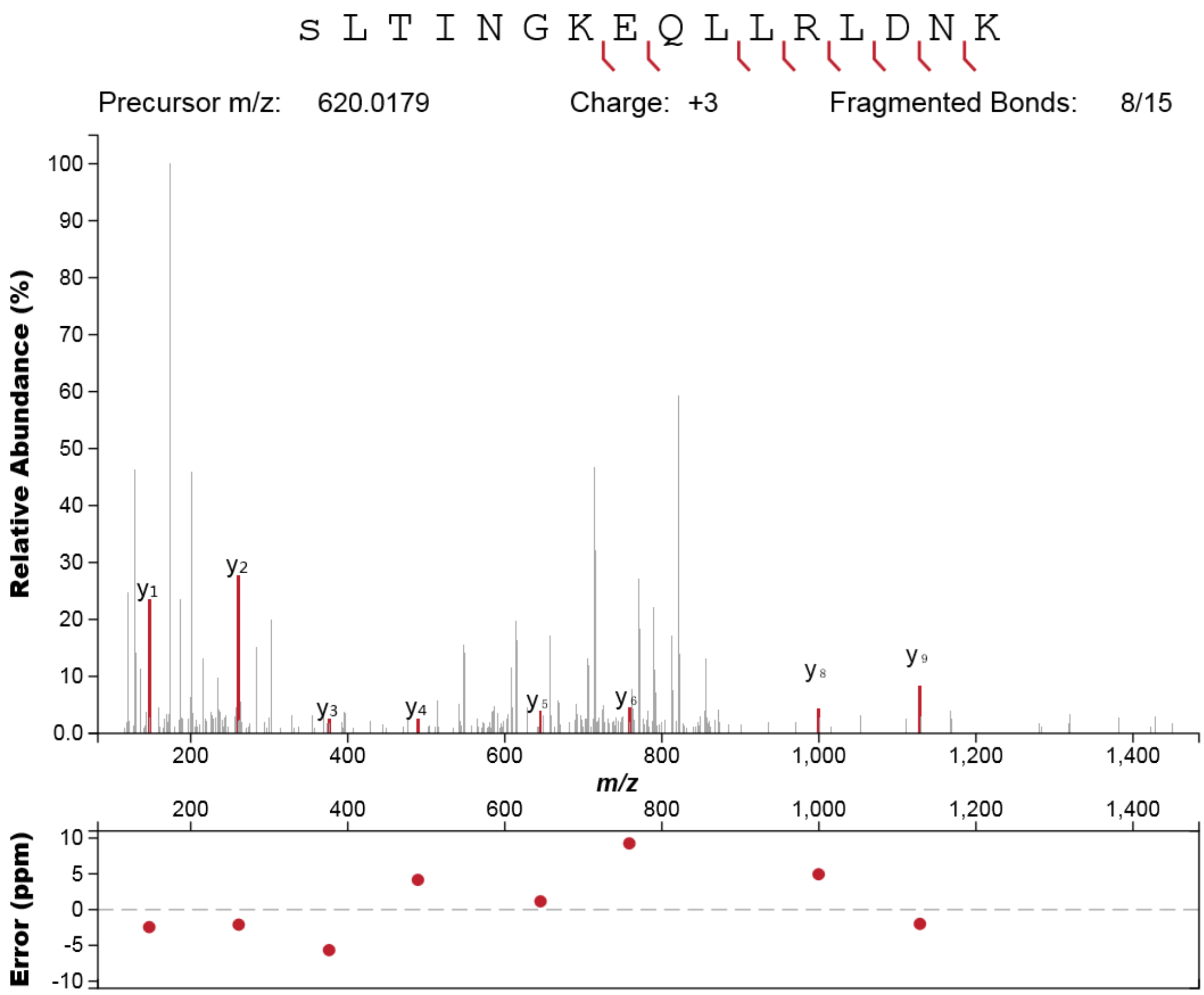

s L T I N G K E Q L L R L D N K
Precursor m/z: 620.0179
Charge: +3
Fragmented Bonds: 8/15
Relative Abundance (%)
m/z
Error (ppm)
y1
y2
y3
y4
y5
y6
y8
y9

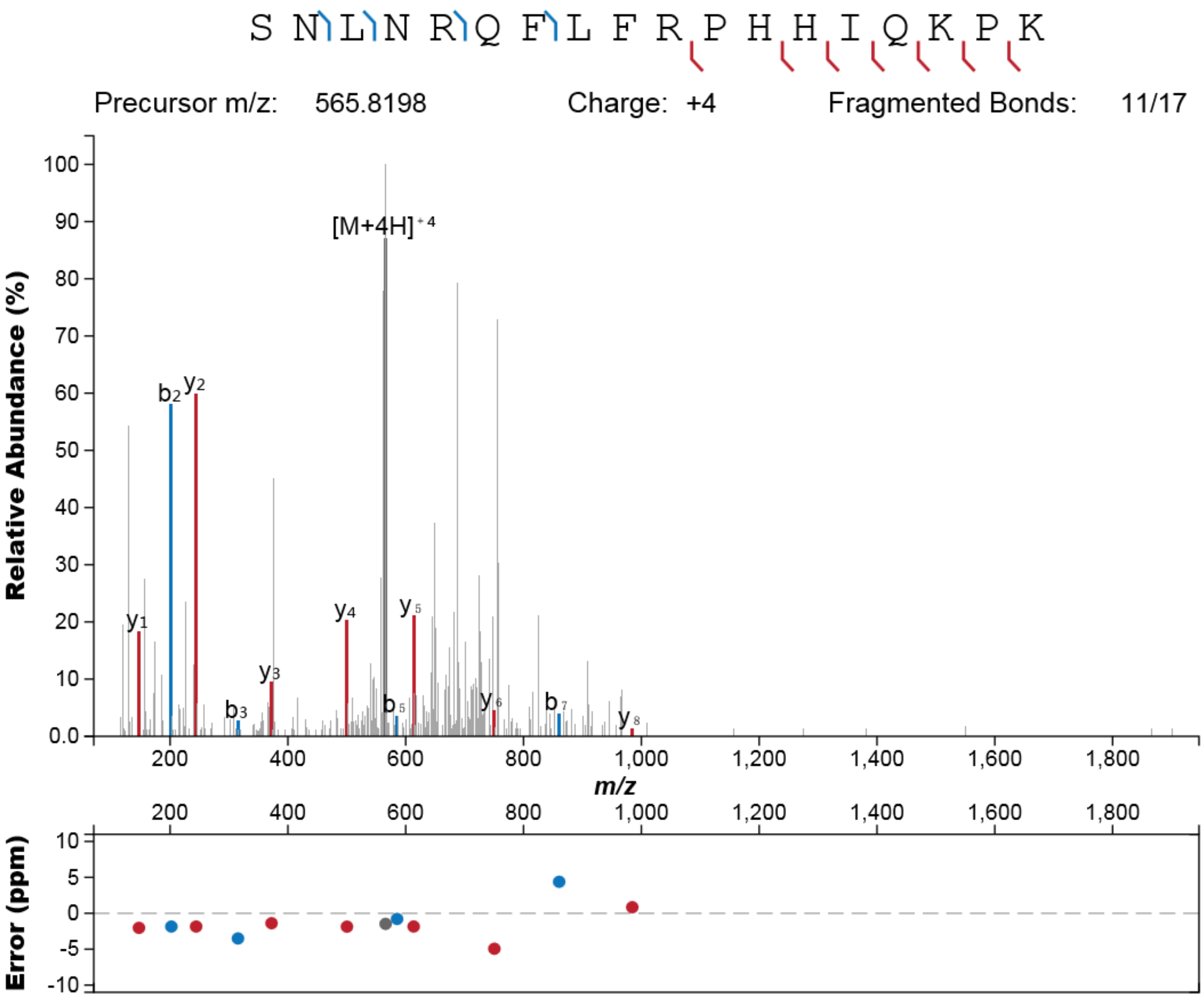

S N L N R Q F L F R P H H I Q K P K
Precursor m/z: 565.8198
Charge: +4
Fragmented Bonds: 11/17
Relative Abundance (%)
[M+4H]+4
b2
y2
y1
b3
y3
y4
b5
y5
y6
b7
y8
m/z
Error (ppm)

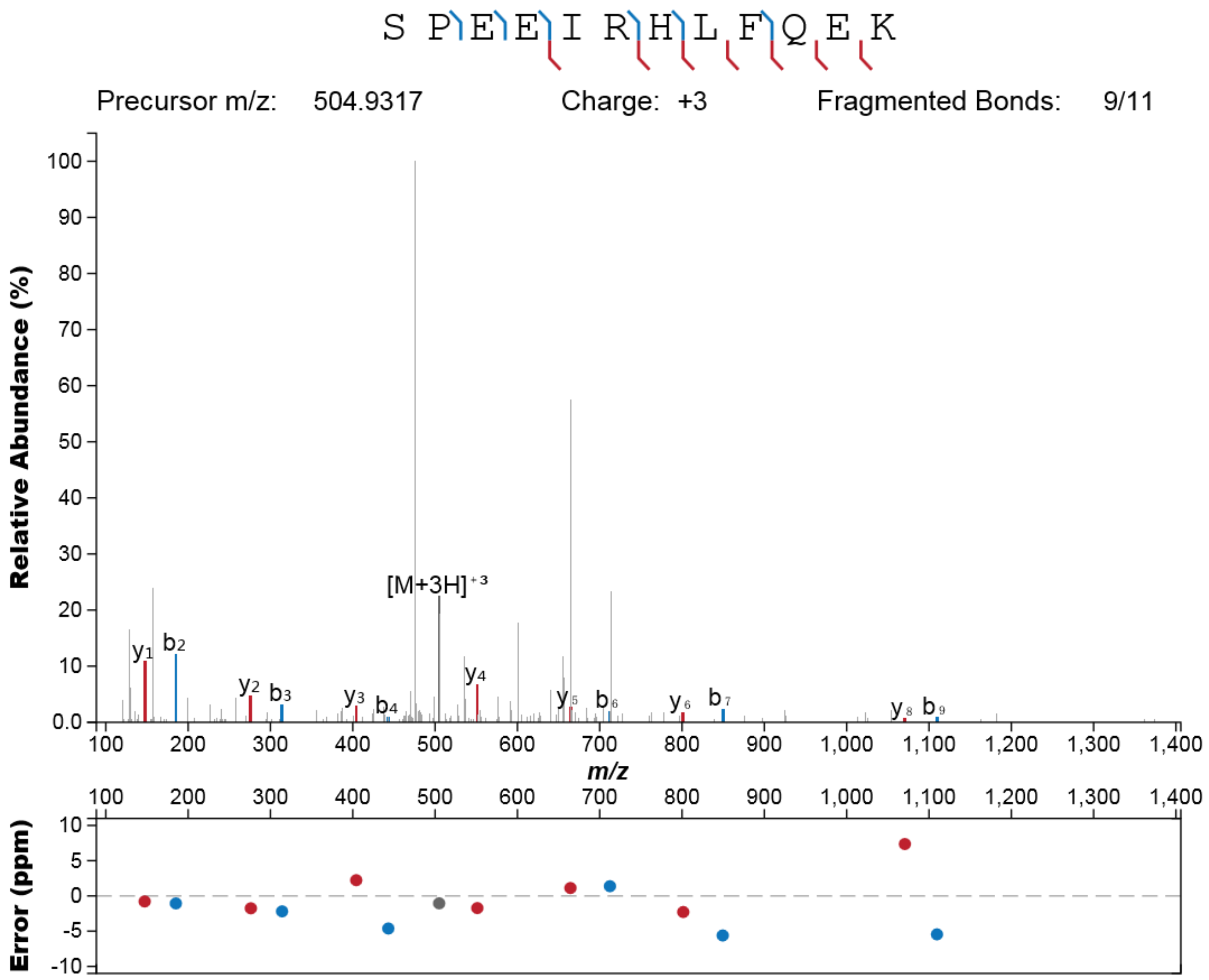
S P E E I R H L F Q E K
Precursor m/z: 504.9317
Charge: +3
Fragmented Bonds: 9/11
Relative Abundance (%)
[M+3H]+3
y1
b2
y2
b3
y3
b4
y4
y5
b6
y6
b7
y8
b9
m/z
Error (ppm)

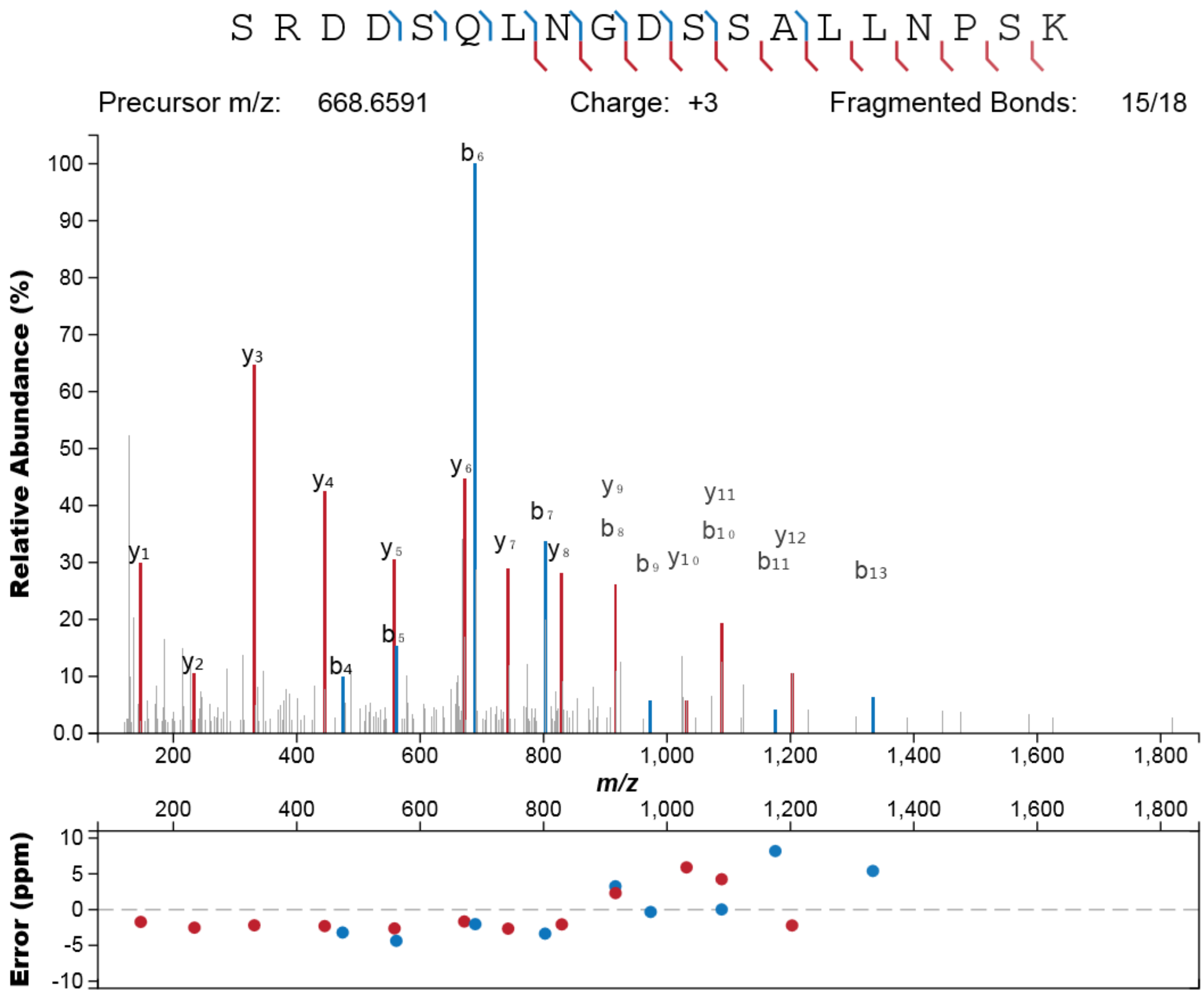

S R D D S Q L N G D S S A L L N P S K
Precursor m/z: 668.6591
Charge: +3
Fragmented Bonds: 15/18
Relative Abundance (%)
m/z
Error (ppm)
b6
y3
y4
y6
y1
y5
b5
y2
b4
y7
b7
y8
y9
b8
b9
y10
y11
b10
b11
y12
b13

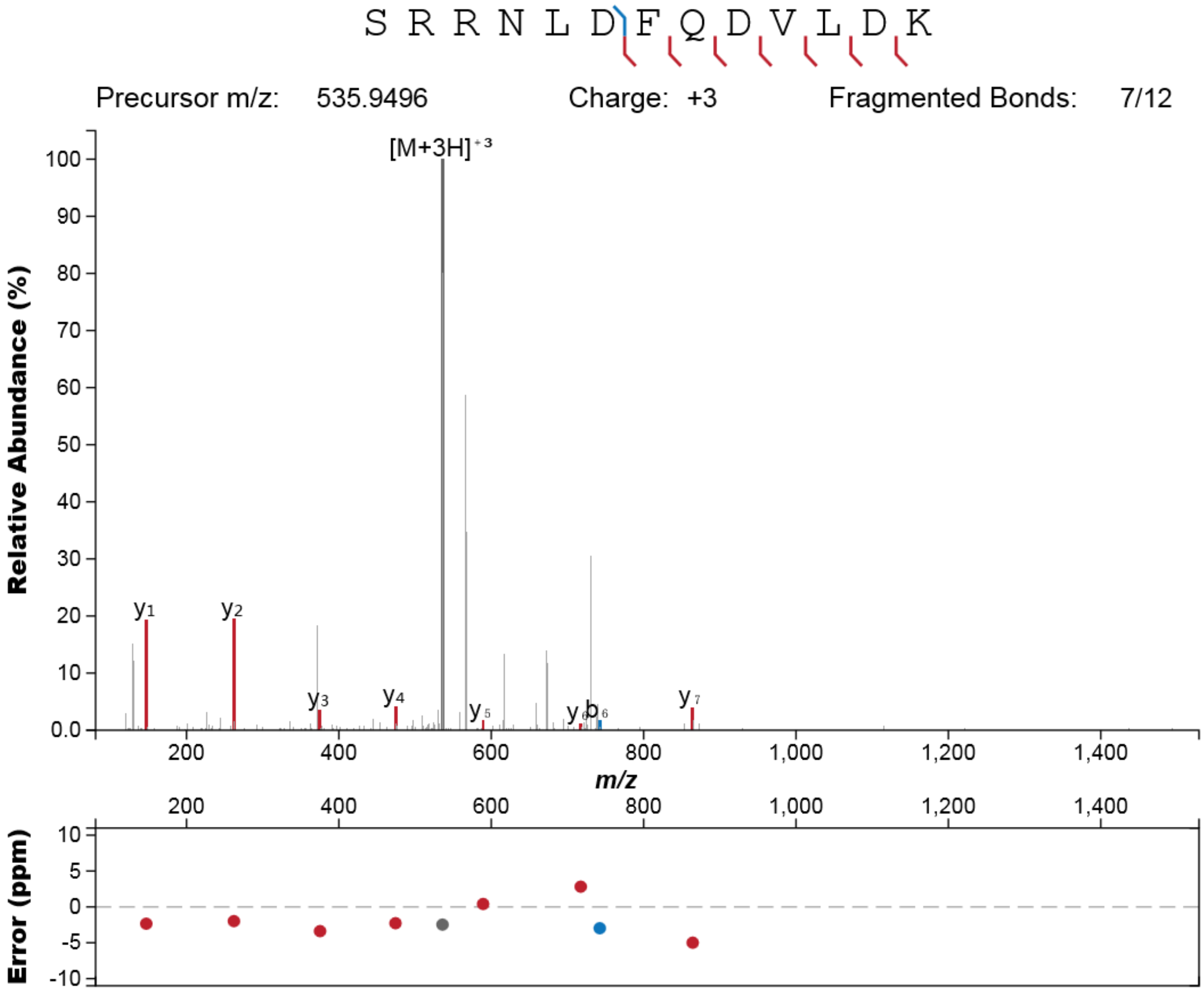

S R R N L D F Q D V L D K
Precursor m/z: 535.9496
Charge: +3
Fragmented Bonds: 7/12
[M+3H]+3
Relative Abundance (%)
m/z
Error (ppm)

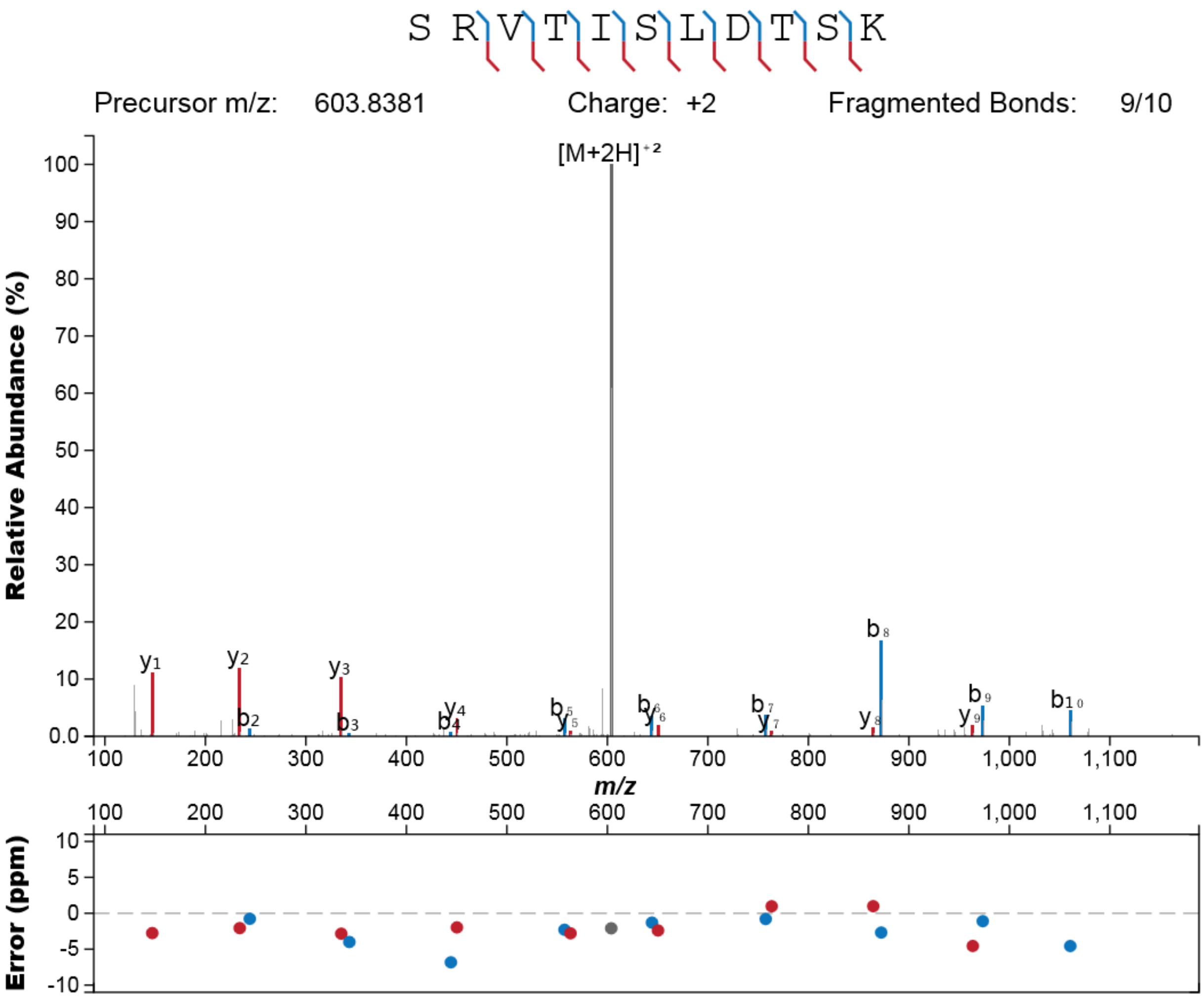

S R V T I S L D T S K
Precursor m/z: 603.8381
Charge: +2
Fragmented Bonds: 9/10
[M+2H]+2
Relative Abundance (%)
m/z
Error (ppm)
y1
y2
y3
y4
y5
y6
y7
y8
y9
b2
b3
b4
b5
b6
b7
b8
b9
b10

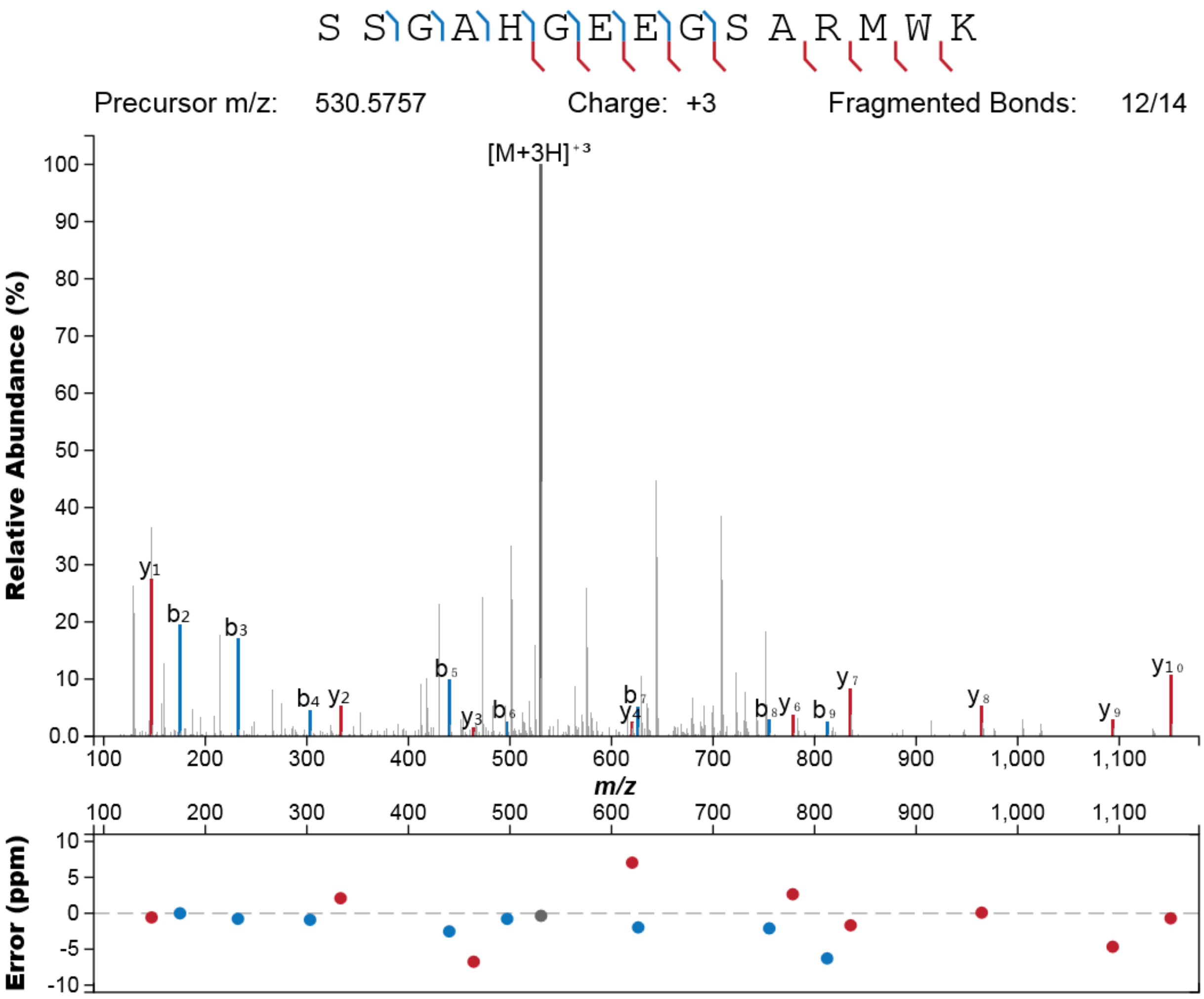

S S G A H G E E G S A R M W K
Precursor m/z: 530.5757
Charge: +3
Fragmented Bonds: 12/14
[M+3H]+3
Relative Abundance (%)
m/z
Error (ppm)
y1
b2
b3
b4
y2
b5
y3
b6
b7
y4
b8
y6
b9
y7
y8
y9
y10

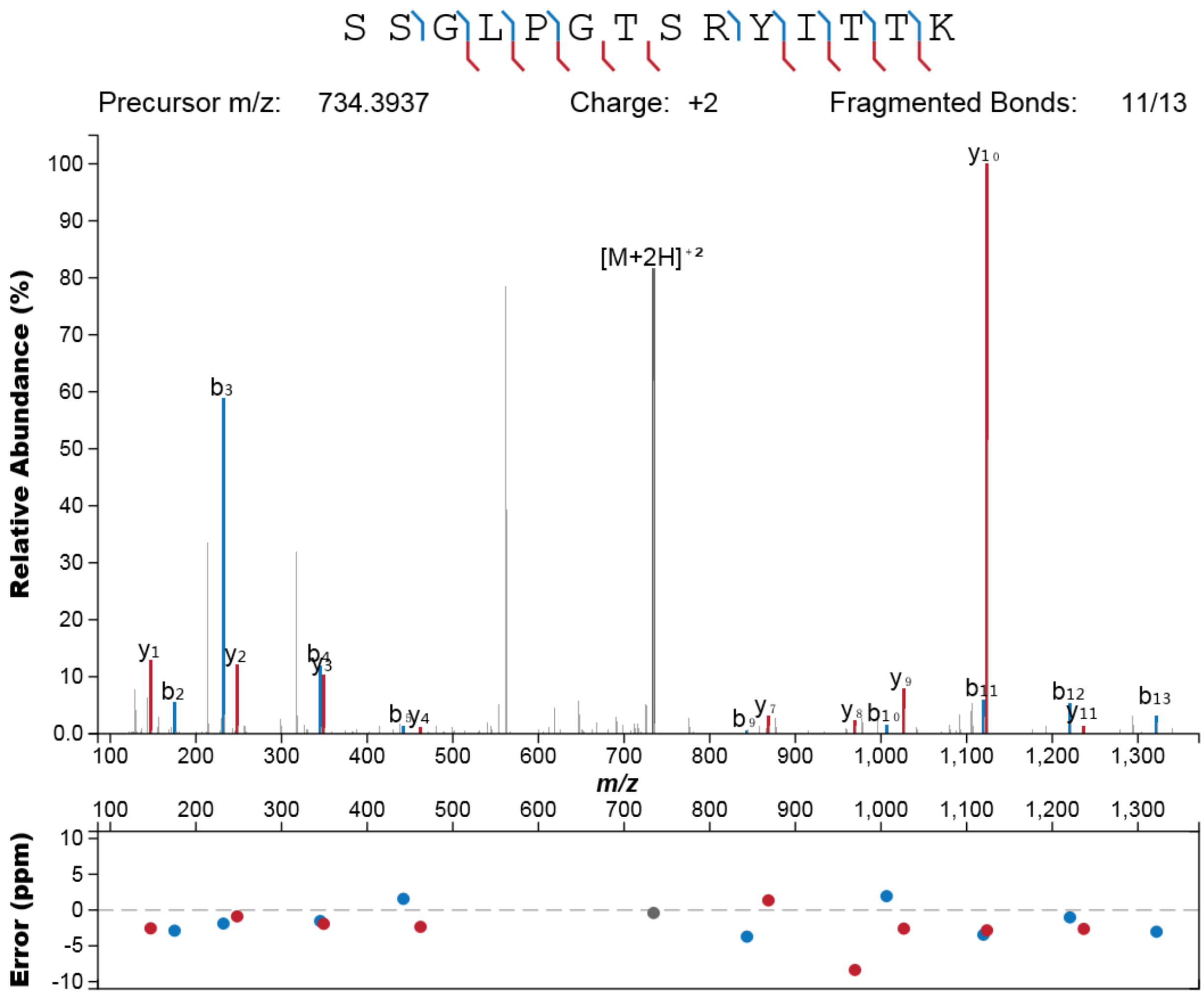

S S G L P G T S R Y I T T K
Precursor m/z: 734.3937
Charge: +2
Fragmented Bonds: 11/13
Relative Abundance (%)
[M+2H]+2
y10
b3
y1
b2
y2
b4
y3
b5
y4
b9
y7
y8
b10
y9
b11
b12
y11
b13
m/z
Error (ppm)

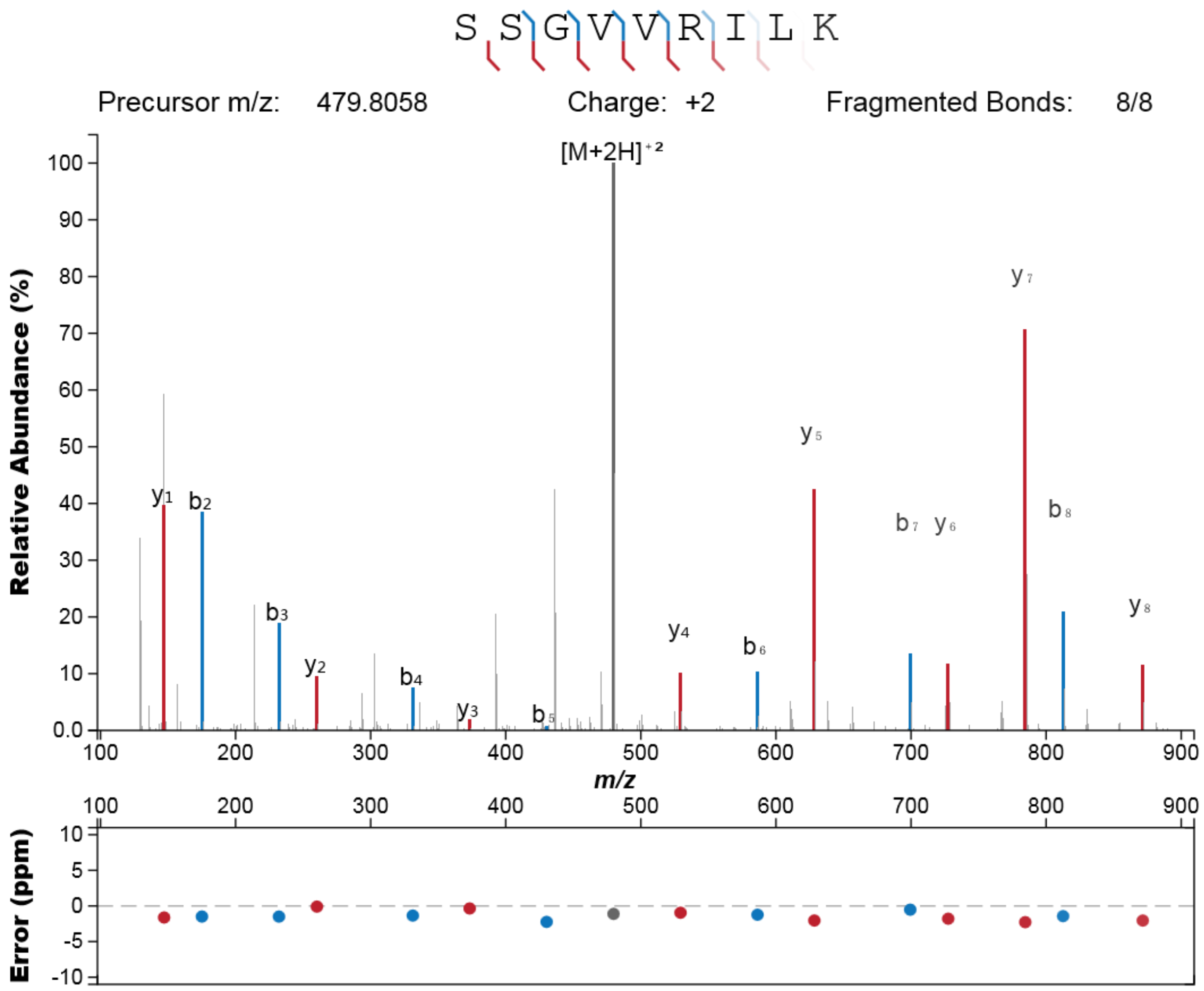

S S G V V R I L K
Precursor m/z: 479.8058
Charge: +2
Fragmented Bonds: 8/8
[M+2H]+2
Relative Abundance (%)
m/z
Error (ppm)
y1
b2
b3
y2
b4
y3
b5
y4
b6
y5
b7
y6
y7
b8
y8

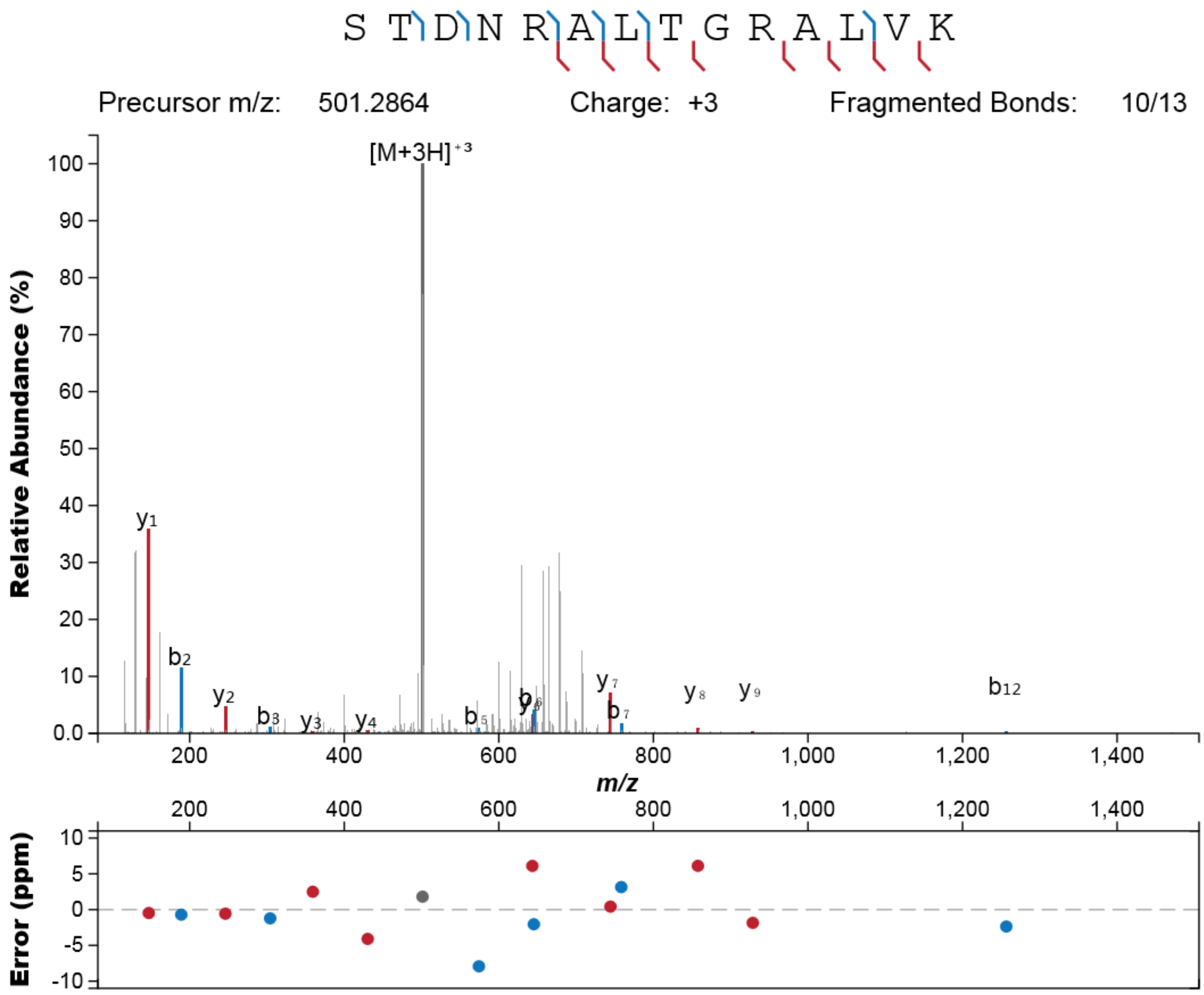
S T D N R A L T G R A L V K
Precursor m/z: 501.2864
Charge: +3
Fragmented Bonds: 10/13
[M+3H]+3
Relative Abundance (%)
m/z
Error (ppm)

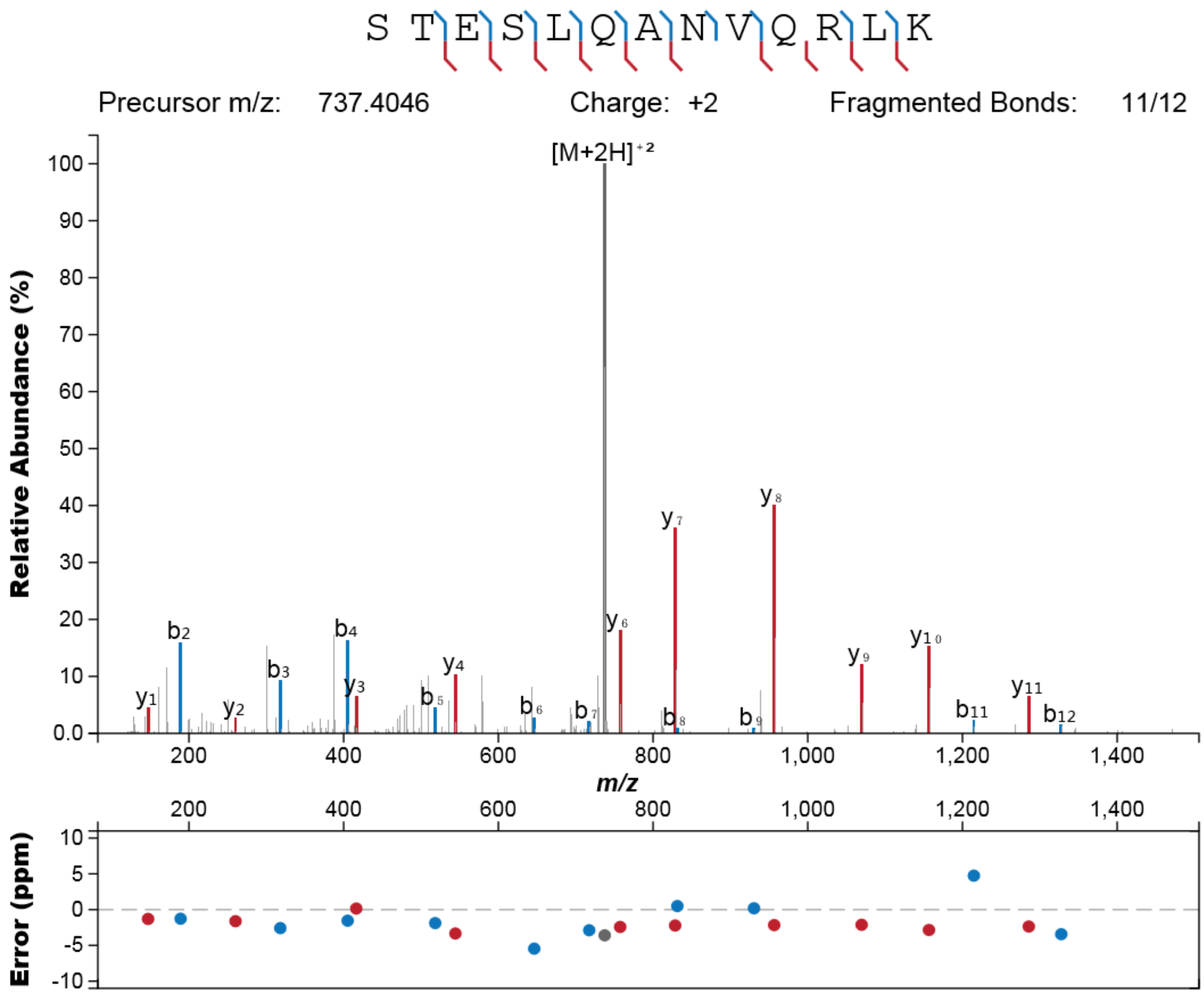

S T E S L Q A N V Q R L K
Precursor m/z: 737.4046
Charge: +2
Fragmented Bonds: 11/12
[M+2H]+2
Relative Abundance (%)
m/z
Error (ppm)
y1 y2 y3 y4 y6 y7 y8 y9 y10 y11
b2 b3 b4 b5 b6 b7 b8 b9 b11 b12

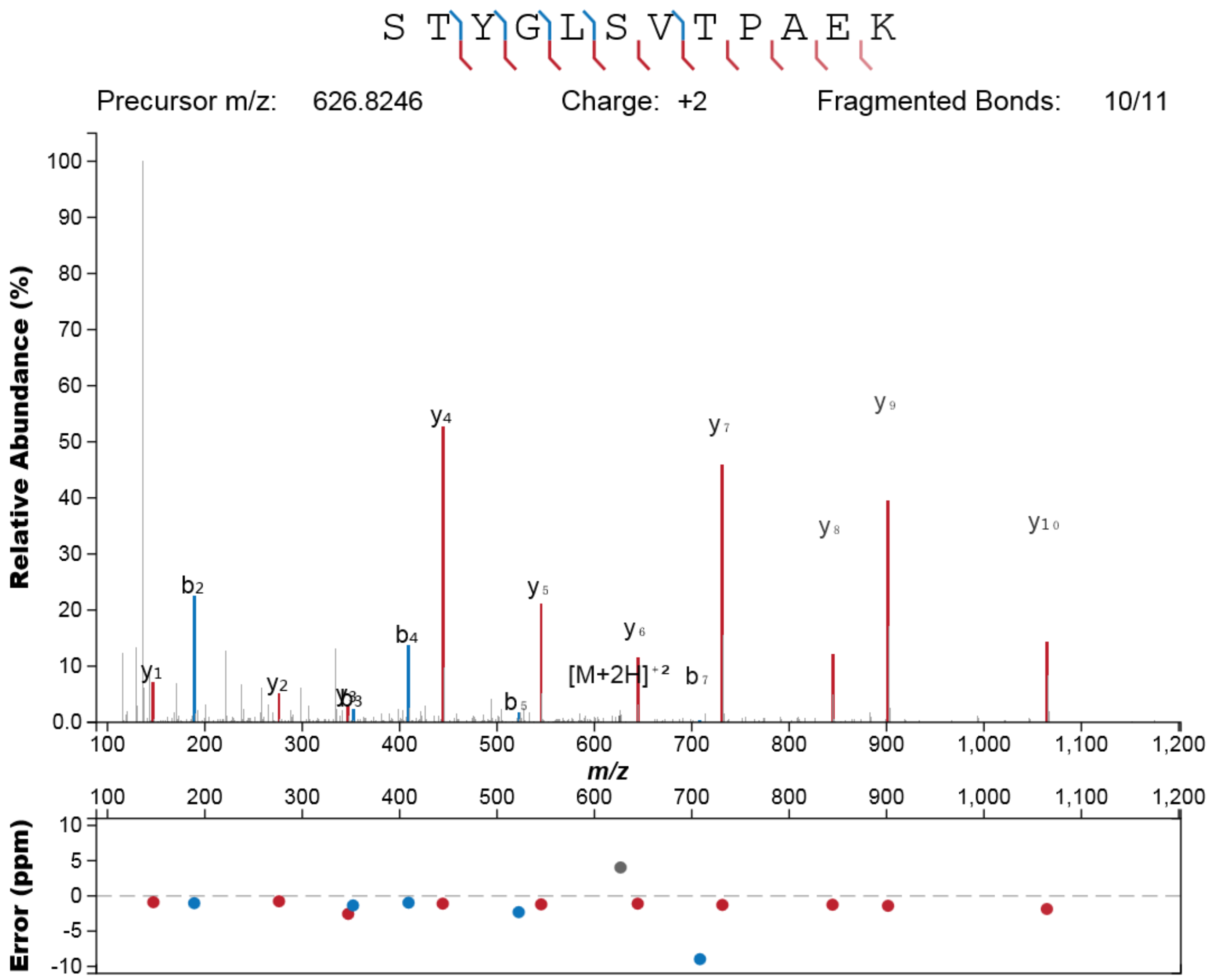

S T Y G L S V T P A E K
Precursor m/z: 626.8246
Charge: +2
Fragmented Bonds: 10/11
Relative Abundance (%)
m/z
Error (ppm)
y1
b2
y2
y3
b3
b4
y4
b5
y5
y6
[M+2H]+2
b7
y7
y8
y9
y10

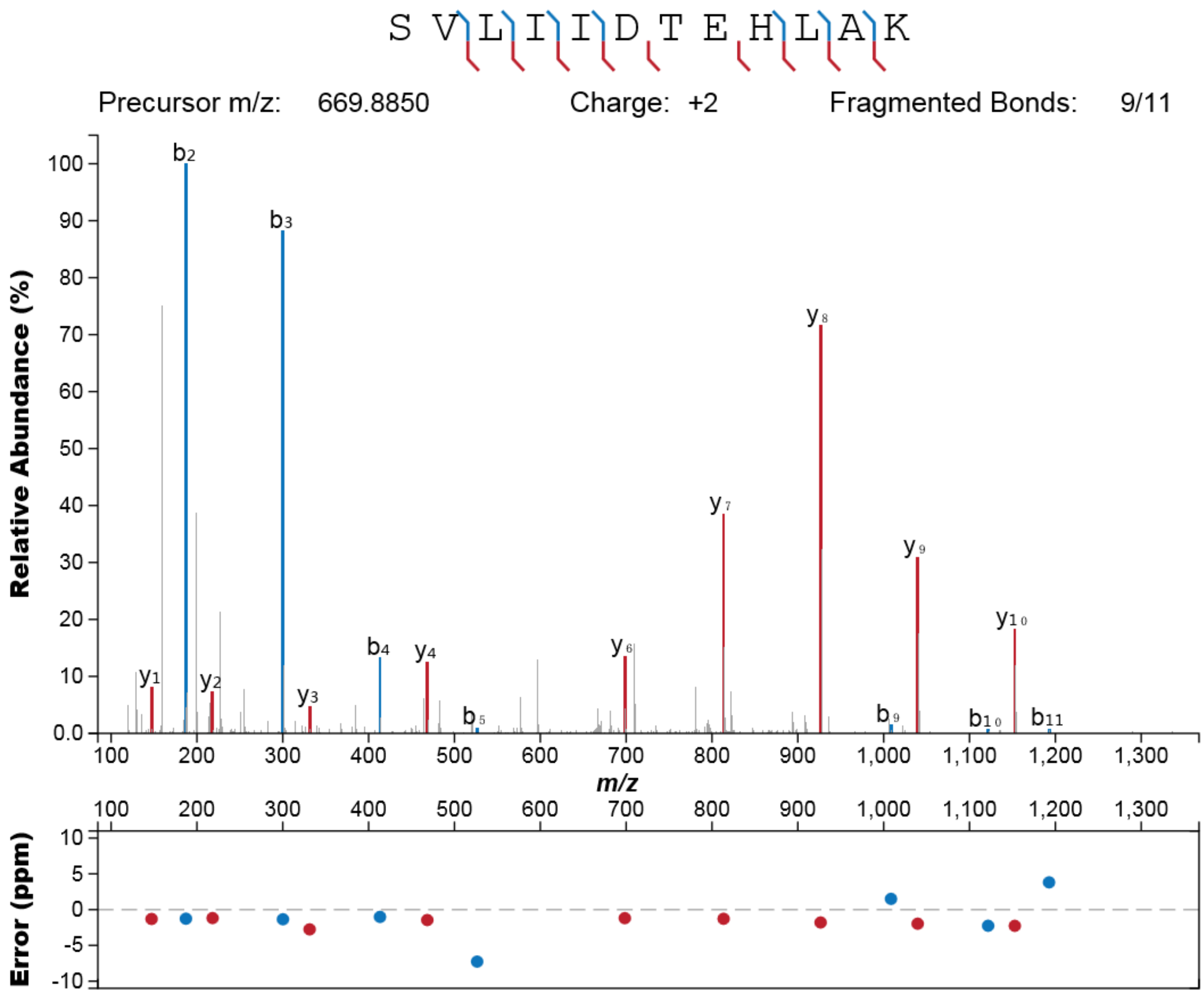
S V L I I D T E H L A K
Precursor m/z: 669.8850
Charge: +2
Fragmented Bonds: 9/11
Relative Abundance (%)
m/z
Error (ppm)
b2
b3
b4
b5
b9
b10
b11
y1
y2
y3
y4
y6
y7
y8
y9
y10

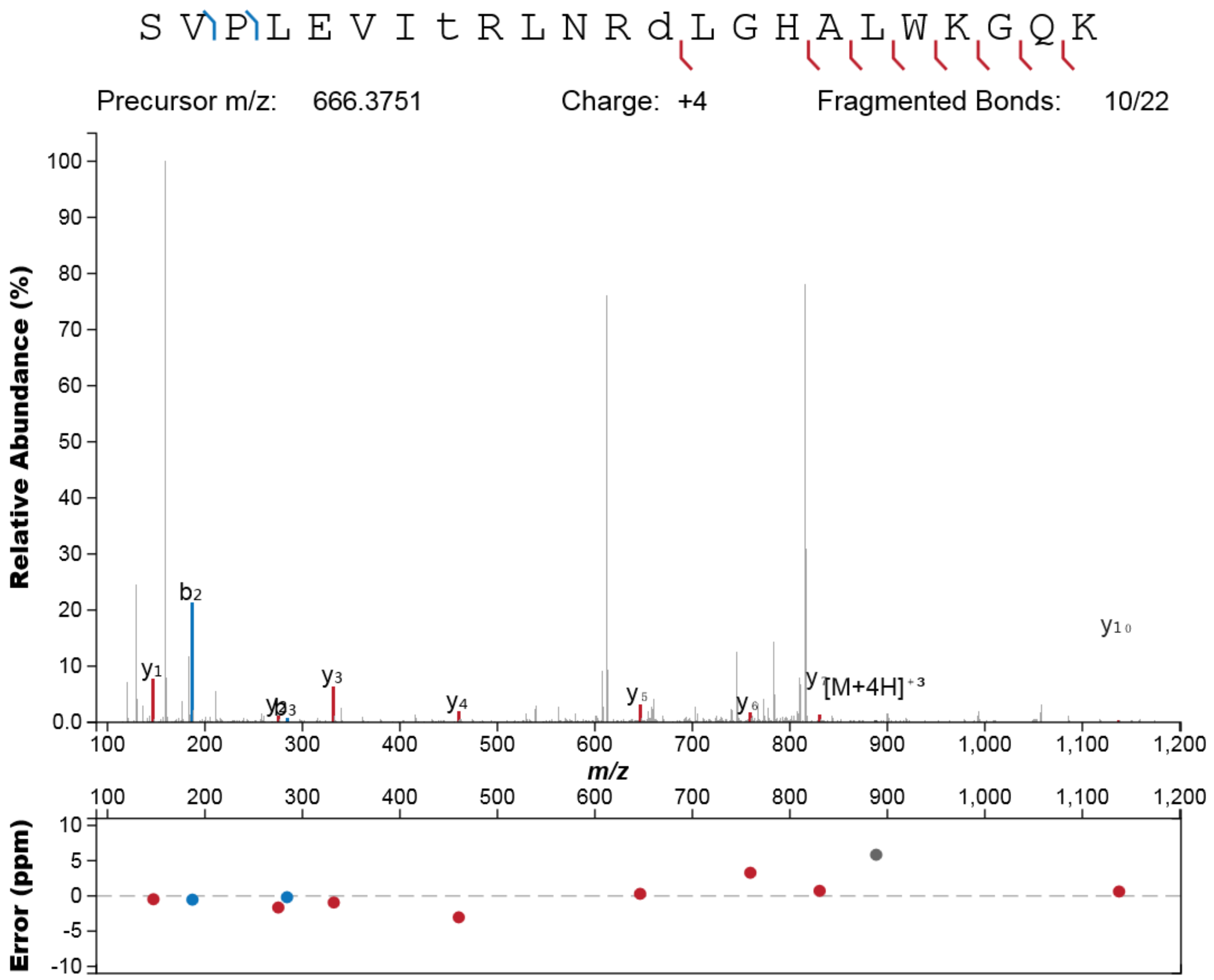

S V P L E V I t R L N R d L G H A L W K G Q K
Precursor m/z: 666.3751
Charge: +4
Fragmented Bonds: 10/22
Relative Abundance (%)
m/z
Error (ppm)
b2
y1
y2
b3
y3
y4
y5
y6
y7
[M+4H]+3
y10

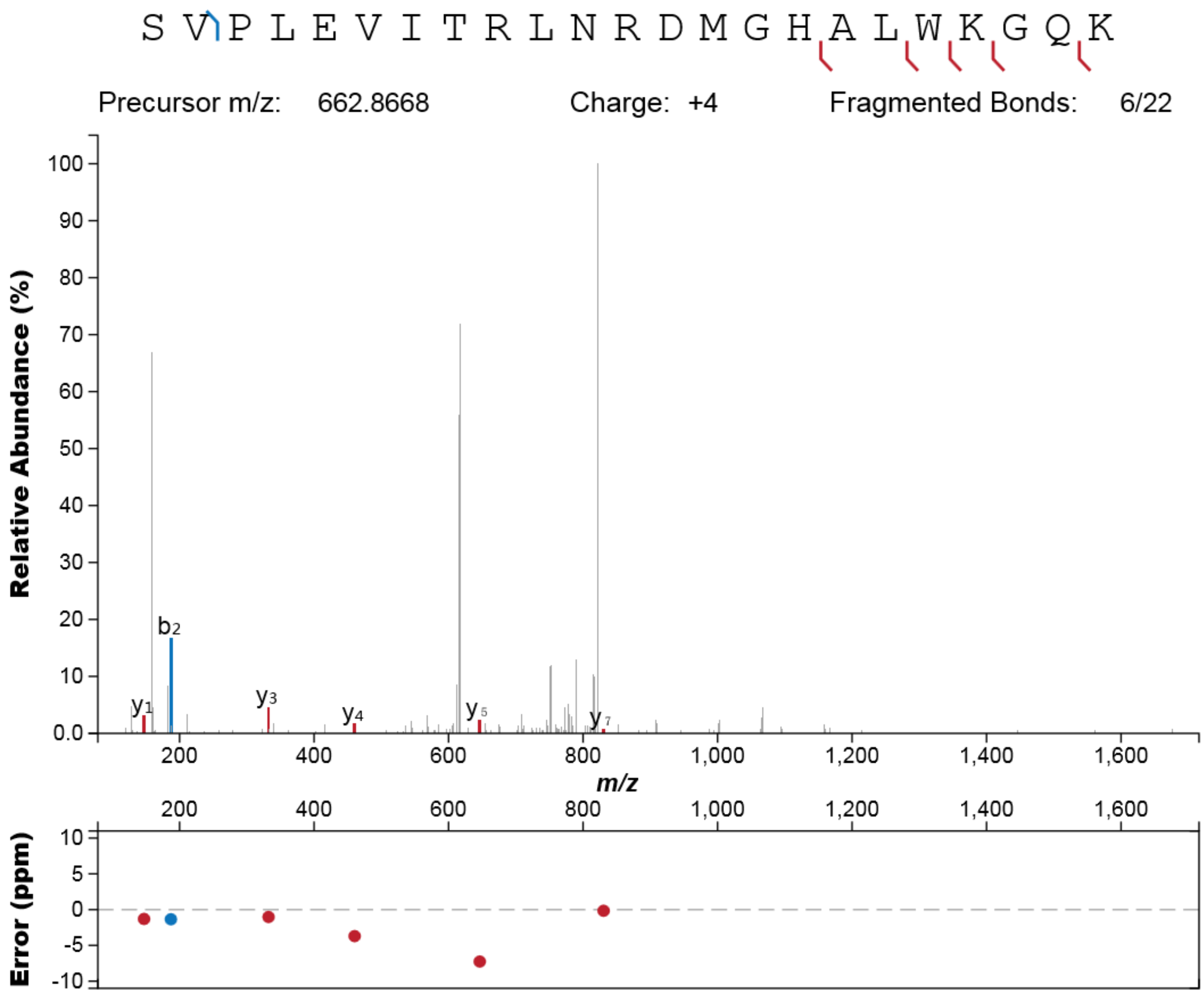
S V P L E V I T R L N R D M G H A L W K G Q K
Precursor m/z: 662.8668
Charge: +4
Fragmented Bonds: 6/22
Relative Abundance (%)
m/z
Error (ppm)
b2
y1
y3
y4
y5
y7

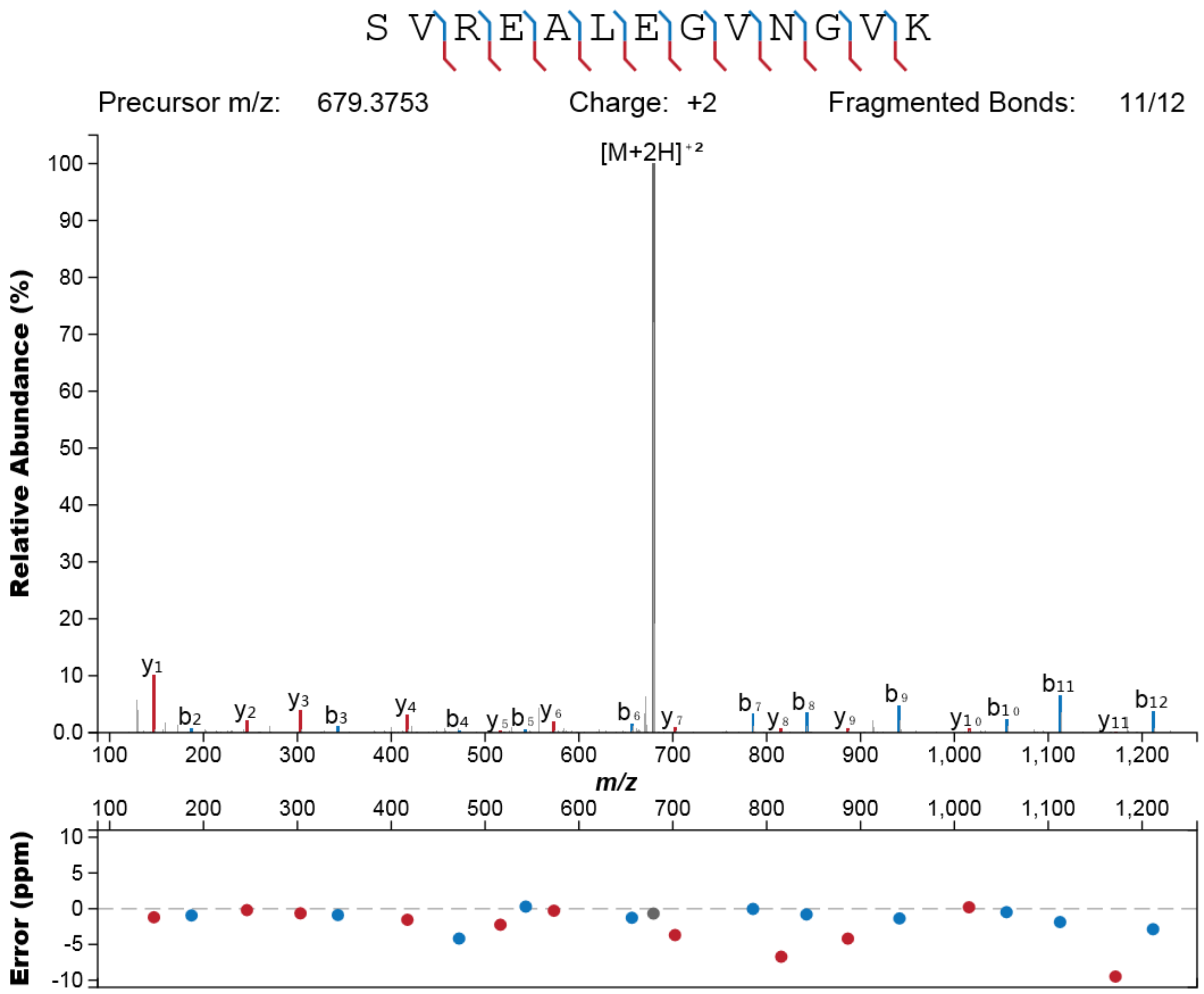

S V R E A L E G V N G V K
Precursor m/z: 679.3753
Charge: +2
Fragmented Bonds: 11/12
[M+2H]+2
Relative Abundance (%)
m/z
Error (ppm)
y1 b2 y2 y3 b3 y4 b4 y5 b5 y6 b6 y7 b7 y8 b8 y9 b9 y10 b10 b11 y11 b12

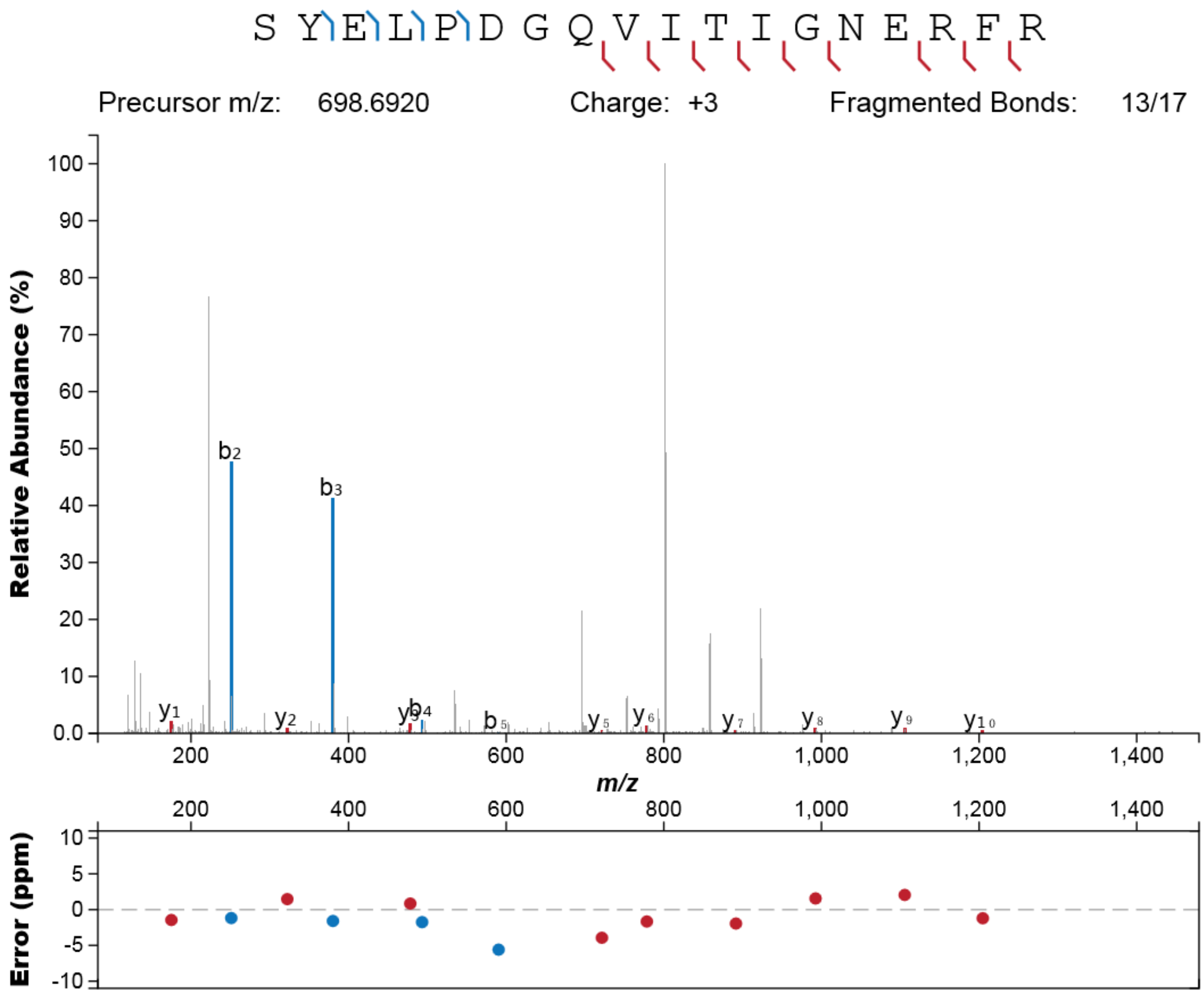

S Y E L P D G Q V I T I G N E R F R
Precursor m/z: 698.6920
Charge: +3
Fragmented Bonds: 13/17
Relative Abundance (%)
b2
b3
b4
b5
y1
y2
y3
y5
y6
y7
y8
y9
y10
m/z
Error (ppm)

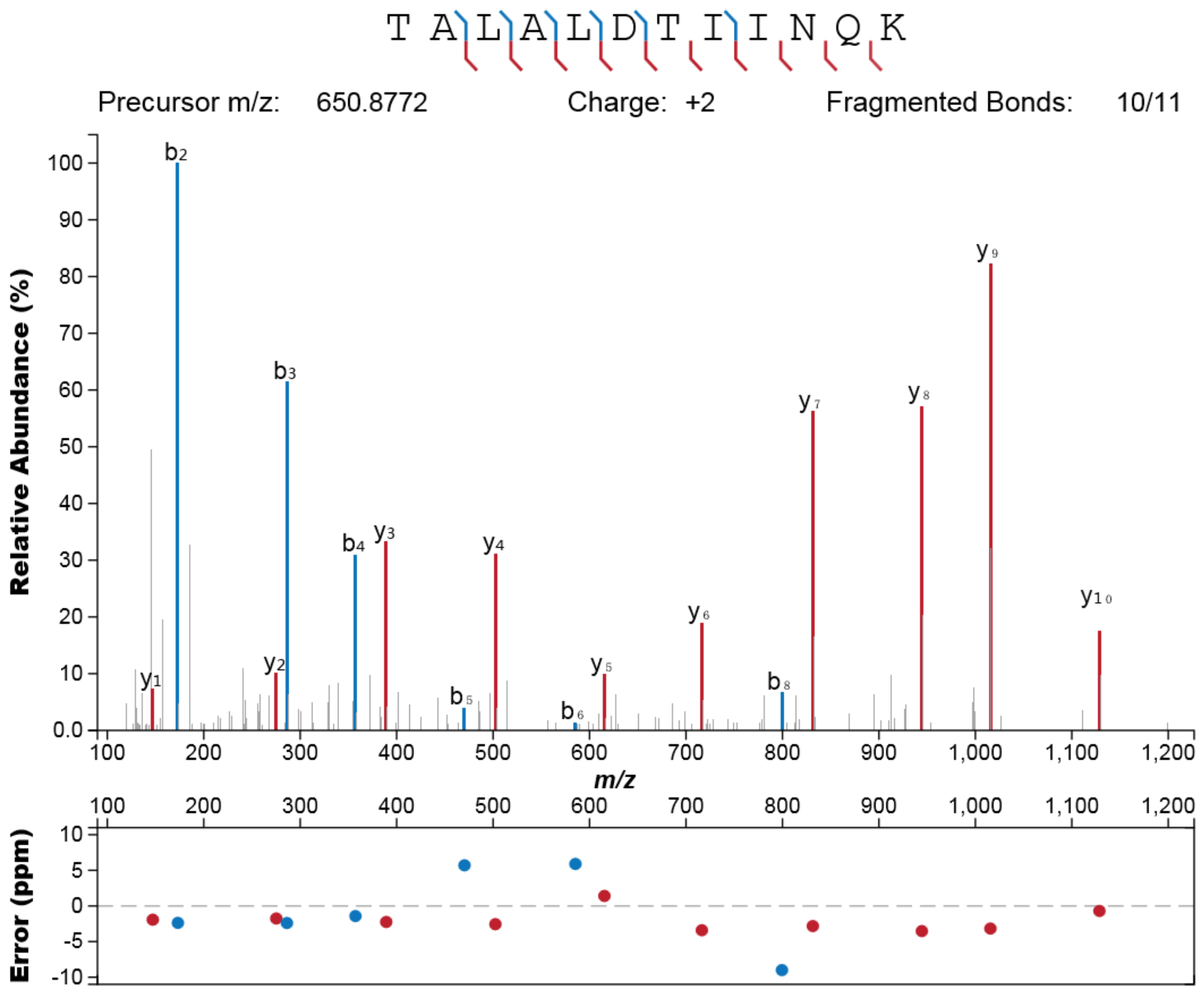

T A L A L D T I I N Q K
Precursor m/z: 650.8772
Charge: +2
Fragmented Bonds: 10/11
Relative Abundance (%)
m/z
Error (ppm)
b2
b3
b4
b5
b6
b8
y1
y2
y3
y4
y5
y6
y7
y8
y9
y10

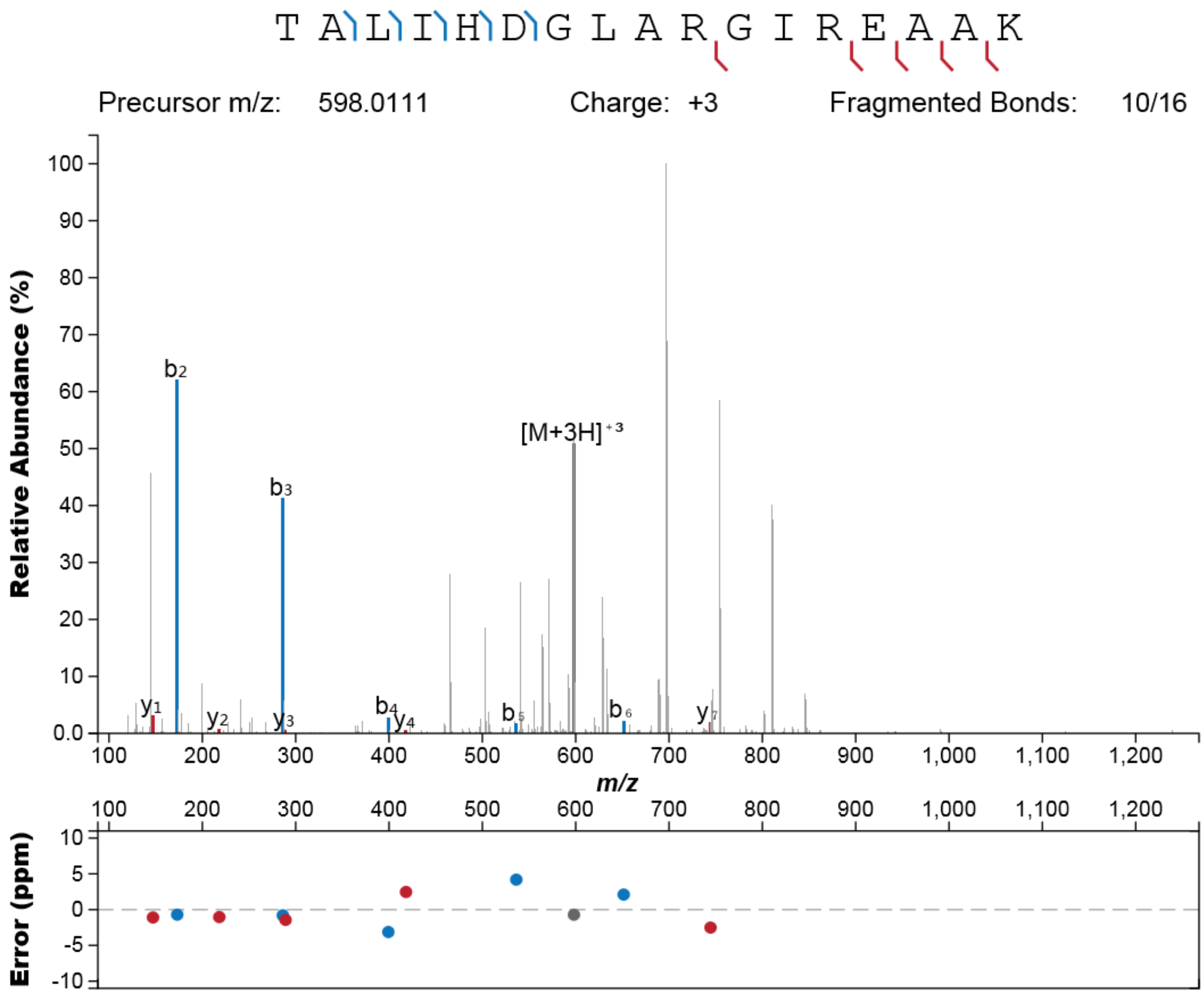
T A L I H D G L A R G I R E A A K
Precursor m/z: 598.0111
Charge: +3
Fragmented Bonds: 10/16
Relative Abundance (%)
[M+3H]+3
b2
b3
b4
b5
b6
y1
y2
y3
y4
y7
m/z
Error (ppm)

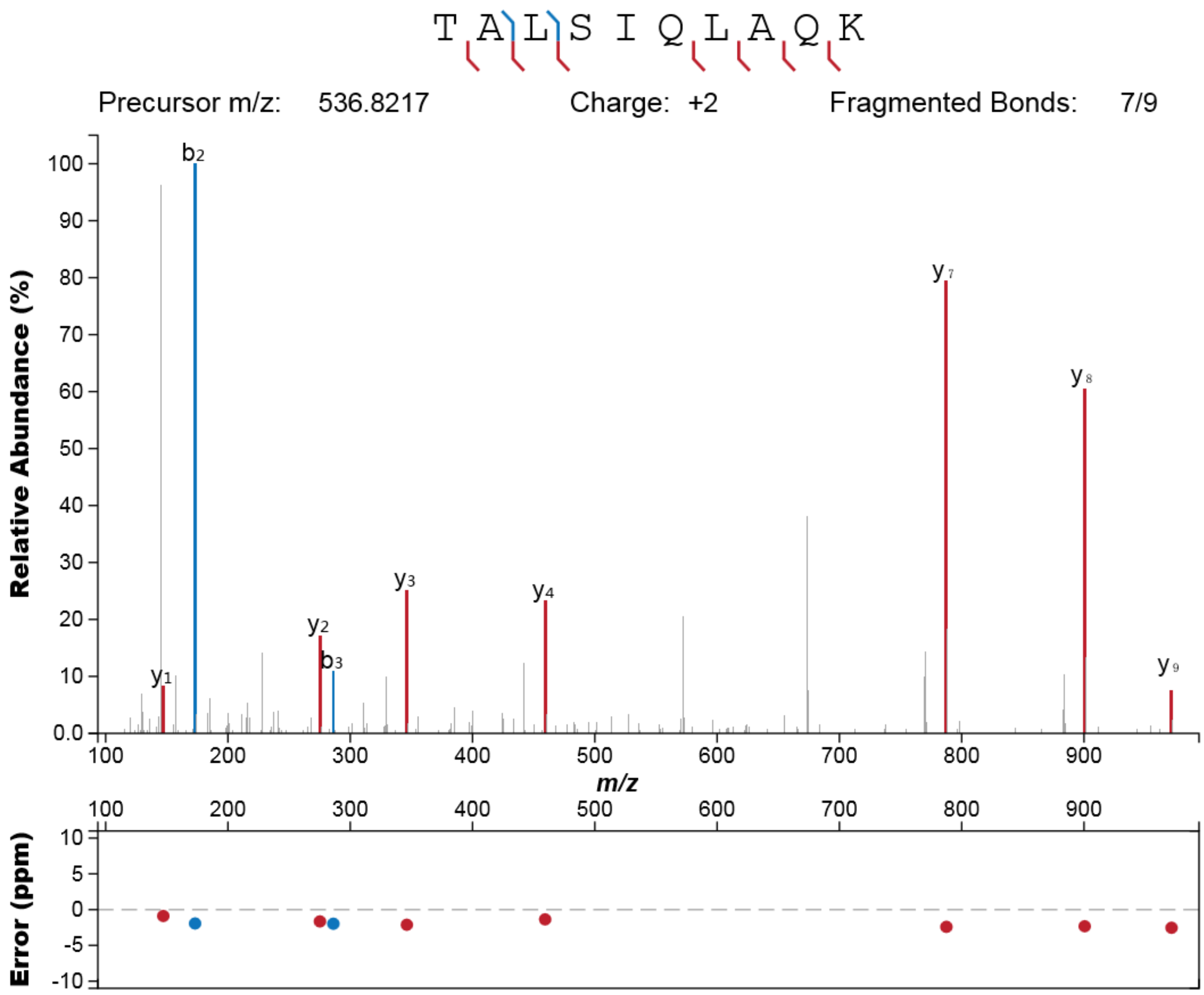
T A L S I Q L A Q K
Precursor m/z: 536.8217
Charge: +2
Fragmented Bonds: 7/9
Relative Abundance (%)
m/z
Error (ppm)
b2
b3
y1
y2
y3
y4
y7
y8
y9

t I R V K F Q G G R E A S G I L K

Precursor m/z: 469.7745 Charge: +4 Fragmented Bonds: 6/16

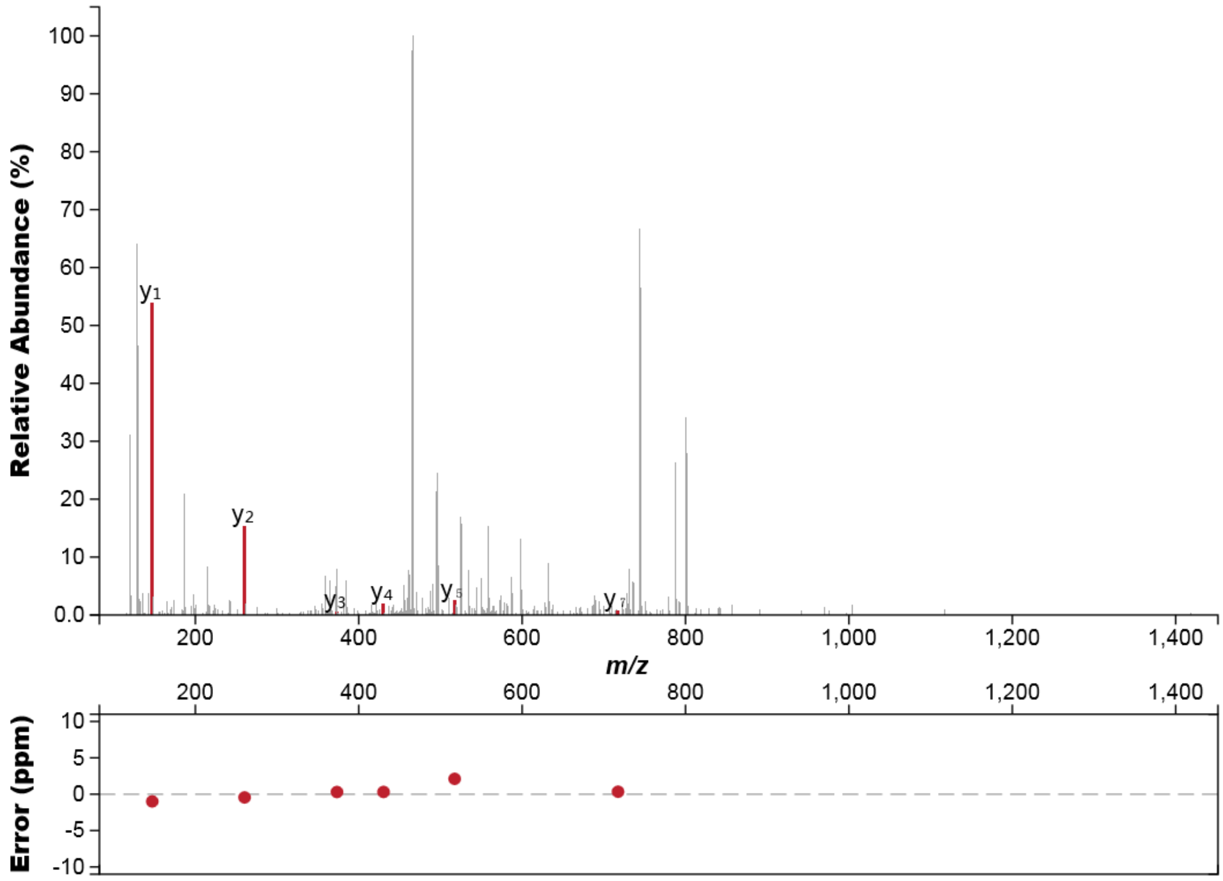

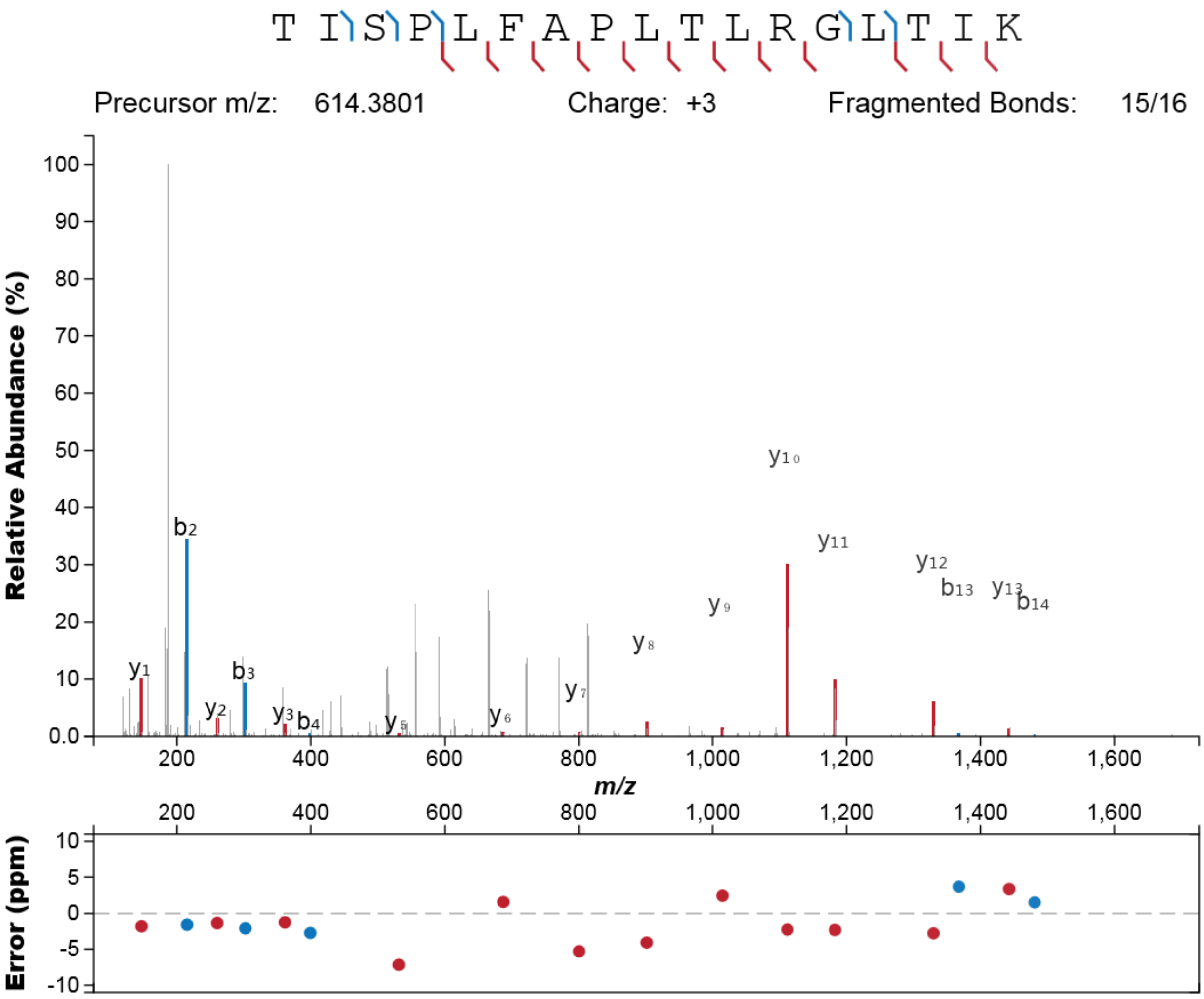

T I S P L F A P L T L R G L T I K
Precursor m/z: 614.3801
Charge: +3
Fragmented Bonds: 15/16
Relative Abundance (%)
m/z
Error (ppm)
y1
b2
y2
b3
y3
b4
y5
y6
y7
y8
y9
y10
y11
y12
b13
y13
b14

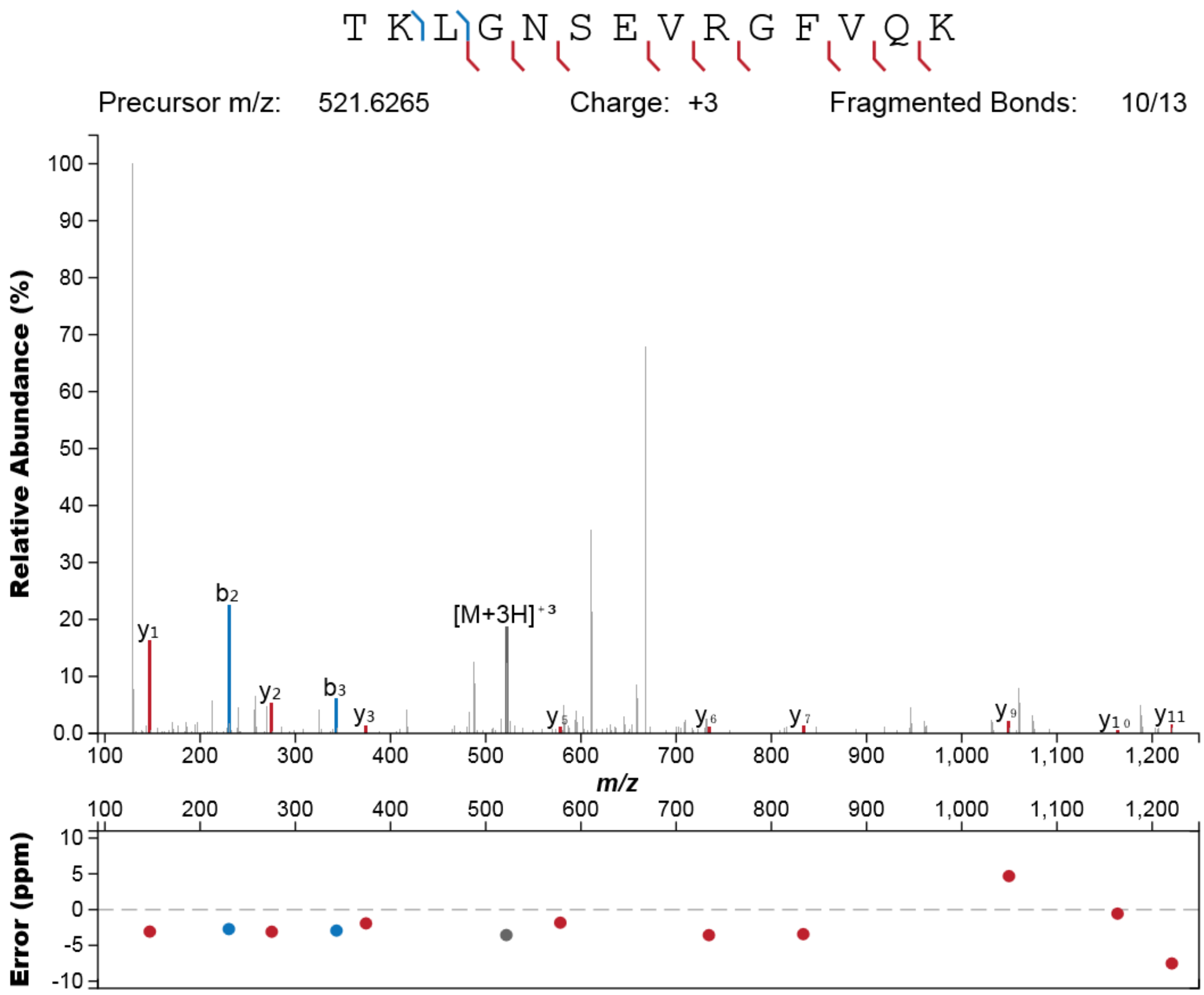

T K L G N S E V R G F V Q K
Precursor m/z: 521.6265
Charge: +3
Fragmented Bonds: 10/13
Relative Abundance (%)
m/z
Error (ppm)
[M+3H]+3
b2
b3
y1
y2
y3
y5
y6
y7
y9
y10
y11

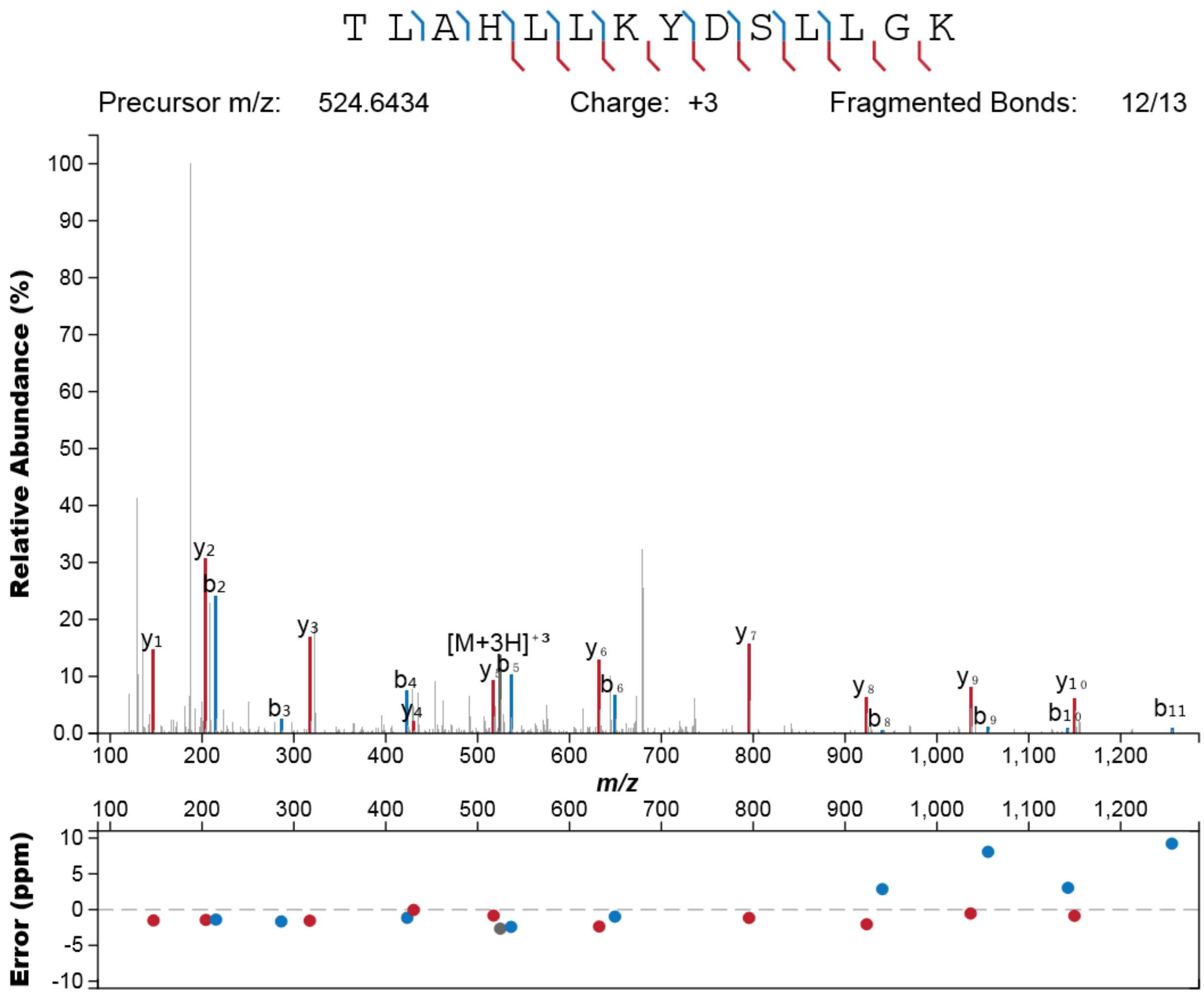

T L A H L L K Y D S L L G K
Precursor m/z: 524.6434
Charge: +3
Fragmented Bonds: 12/13
Relative Abundance (%)
m/z
Error (ppm)
y1 y2 b2 y3 b3 b4 y4 [M+3H]+3 y5 b5 y6 b6 y7 y8 b8 y9 b9 y10 b10 b11

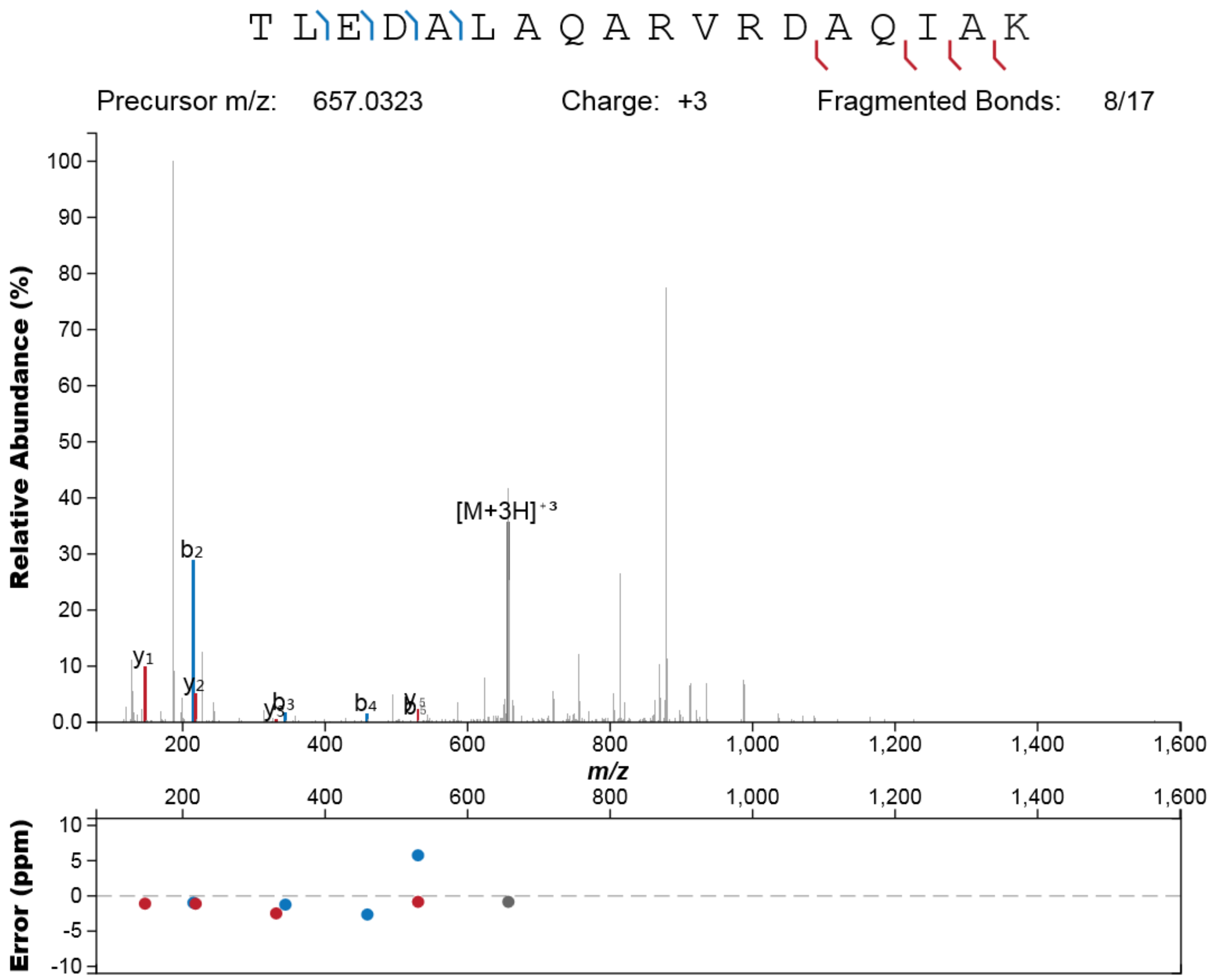
T L E D A L A Q A R V R D A Q I A K
Precursor m/z: 657.0323
Charge: +3
Fragmented Bonds: 8/17
Relative Abundance (%)
[M+3H]+3
b2
y1
y2
b3
b4
m/z
Error (ppm)

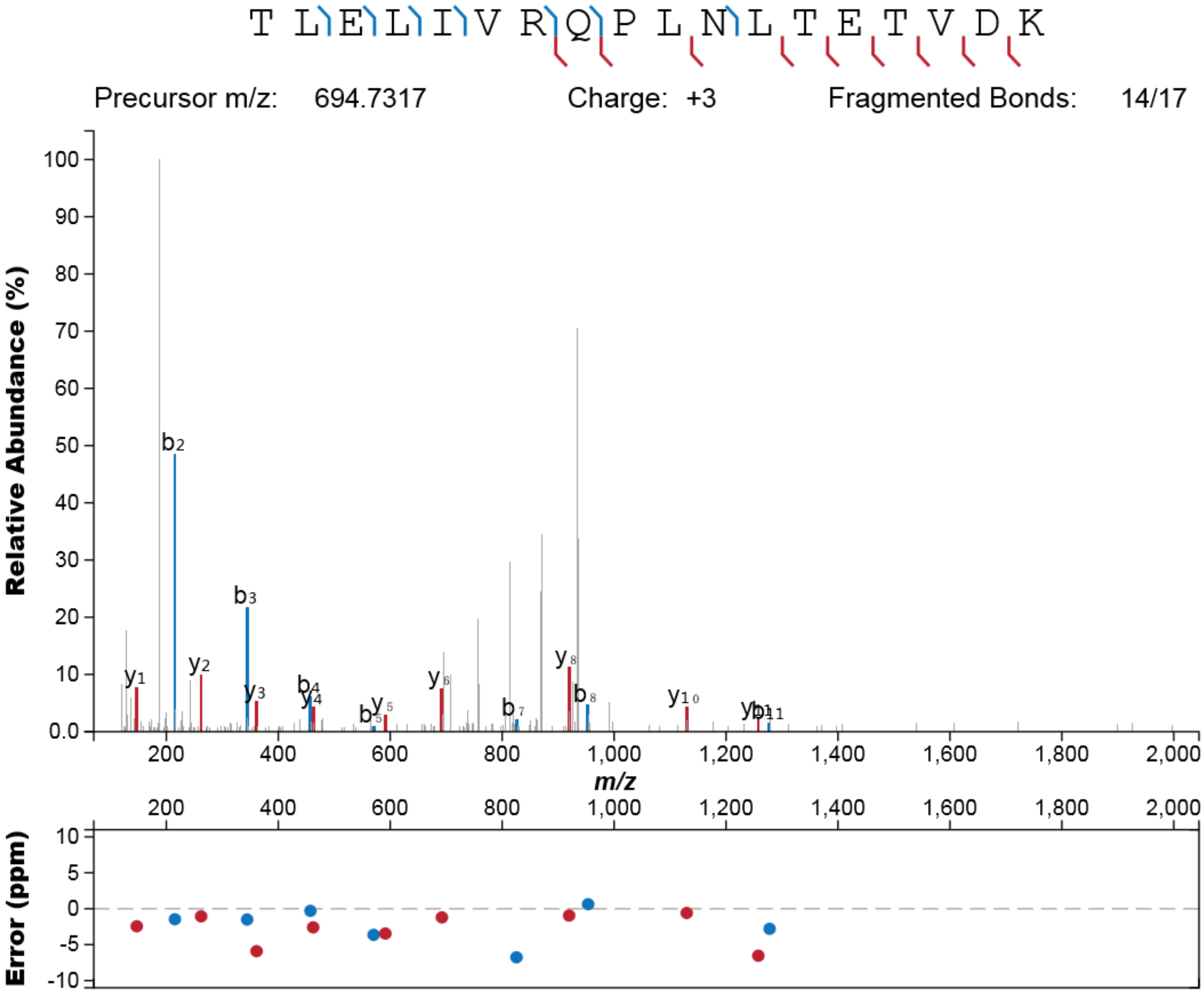

T L E L I V R Q P L N L T E T V D K
Precursor m/z: 694.7317
Charge: +3
Fragmented Bonds: 14/17
Relative Abundance (%)
m/z
Error (ppm)

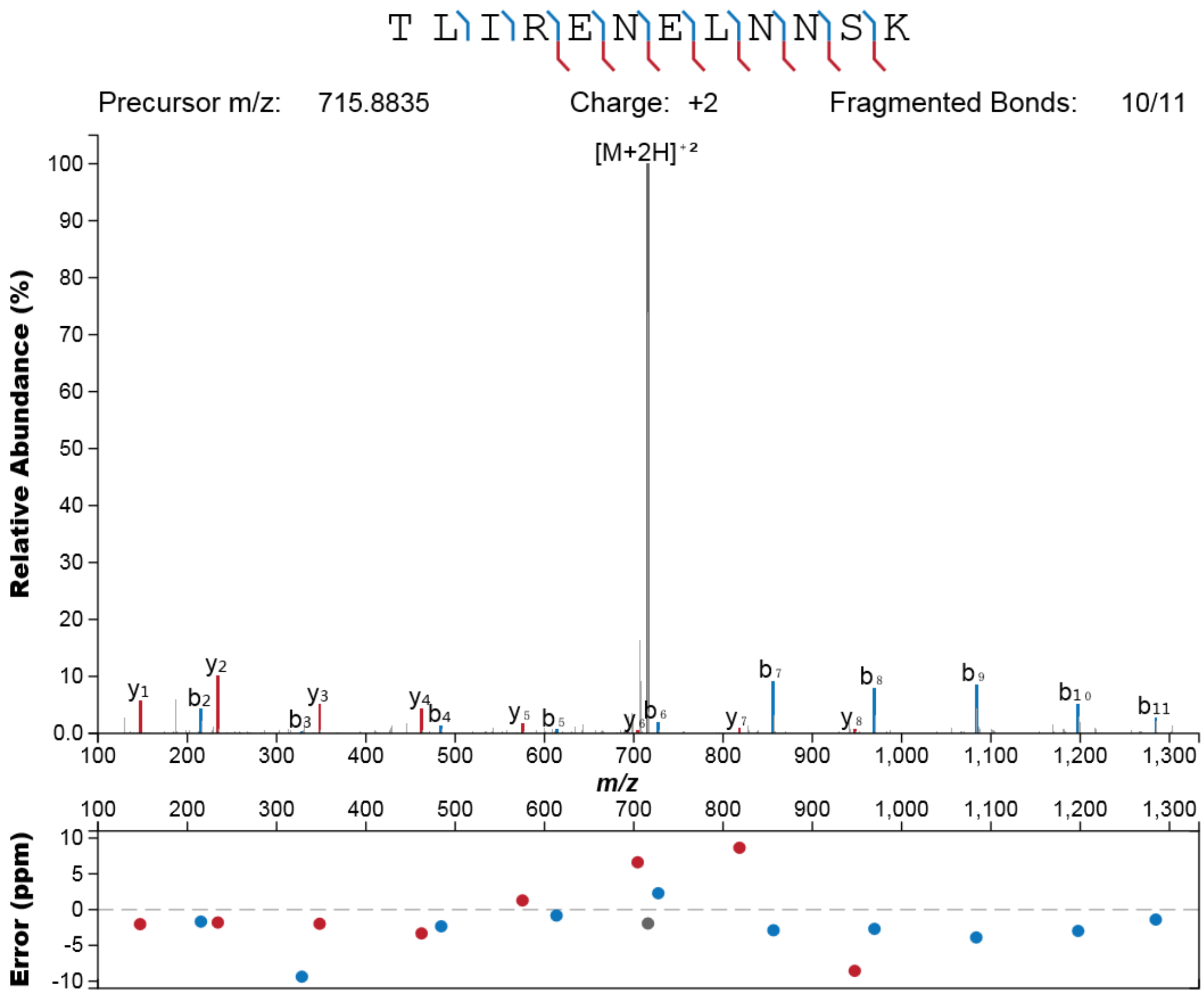

T L I R E N E L N N S K
Precursor m/z: 715.8835
Charge: +2
Fragmented Bonds: 10/11
[M+2H]+2
Relative Abundance (%)
m/z
Error (ppm)
y1
y2
y3
y4
y5
y6
y7
y8
b2
b3
b4
b5
b6
b7
b8
b9
b10
b11

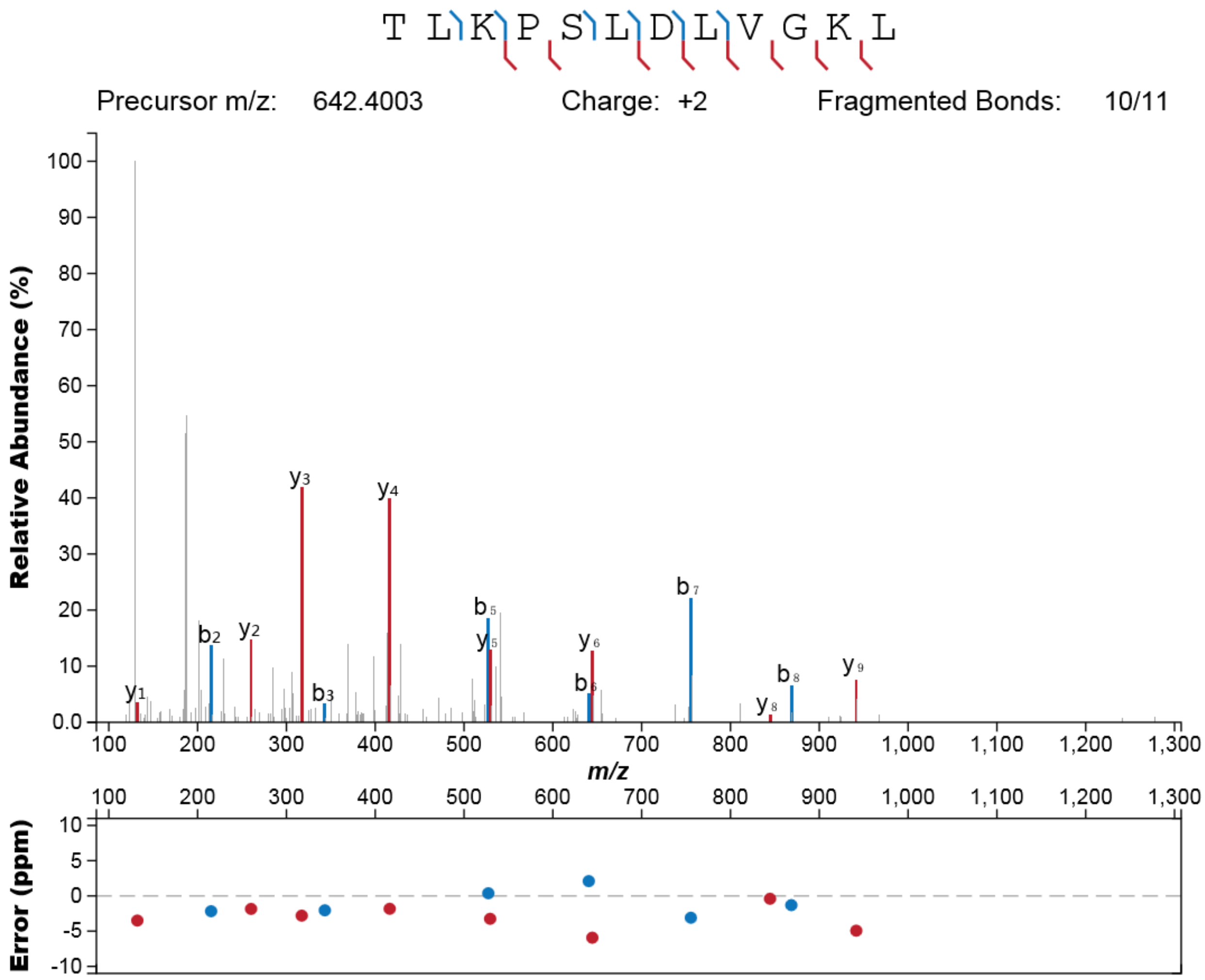

T L K P S L D L V G K L
Precursor m/z: 642.4003
Charge: +2
Fragmented Bonds: 10/11
Relative Abundance (%)
m/z
Error (ppm)
y1
b2
y2
y3
b3
y4
b5
y5
b6
y6
b7
y8
b8
y9

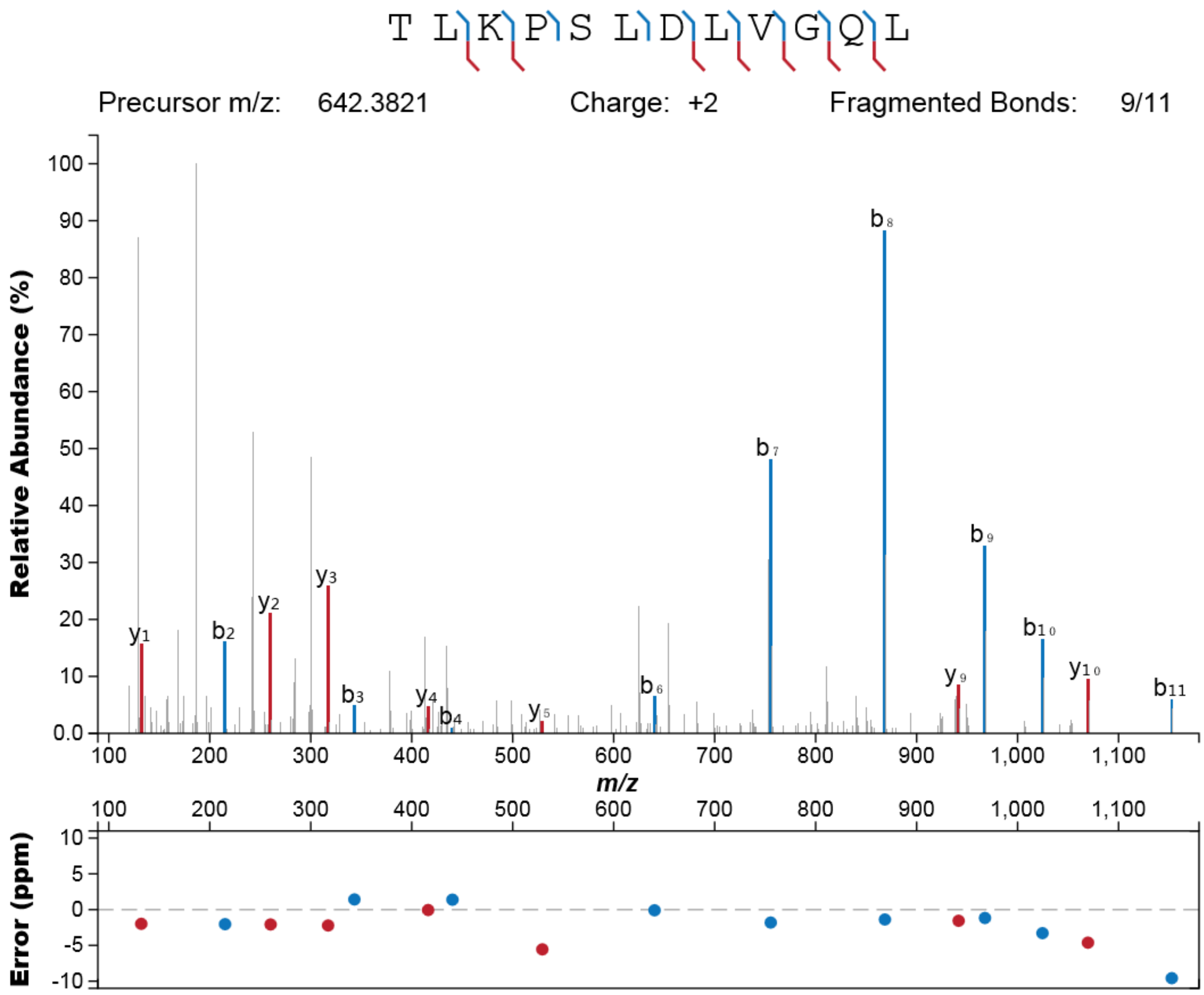

T L K P S L D L V G Q L
Precursor m/z: 642.3821
Charge: +2
Fragmented Bonds: 9/11
Relative Abundance (%)
m/z
Error (ppm)
y1 b2 y2 y3 b3 y4 b4 y5 b6 b7 b8 y9 b9 b10 y10 b11

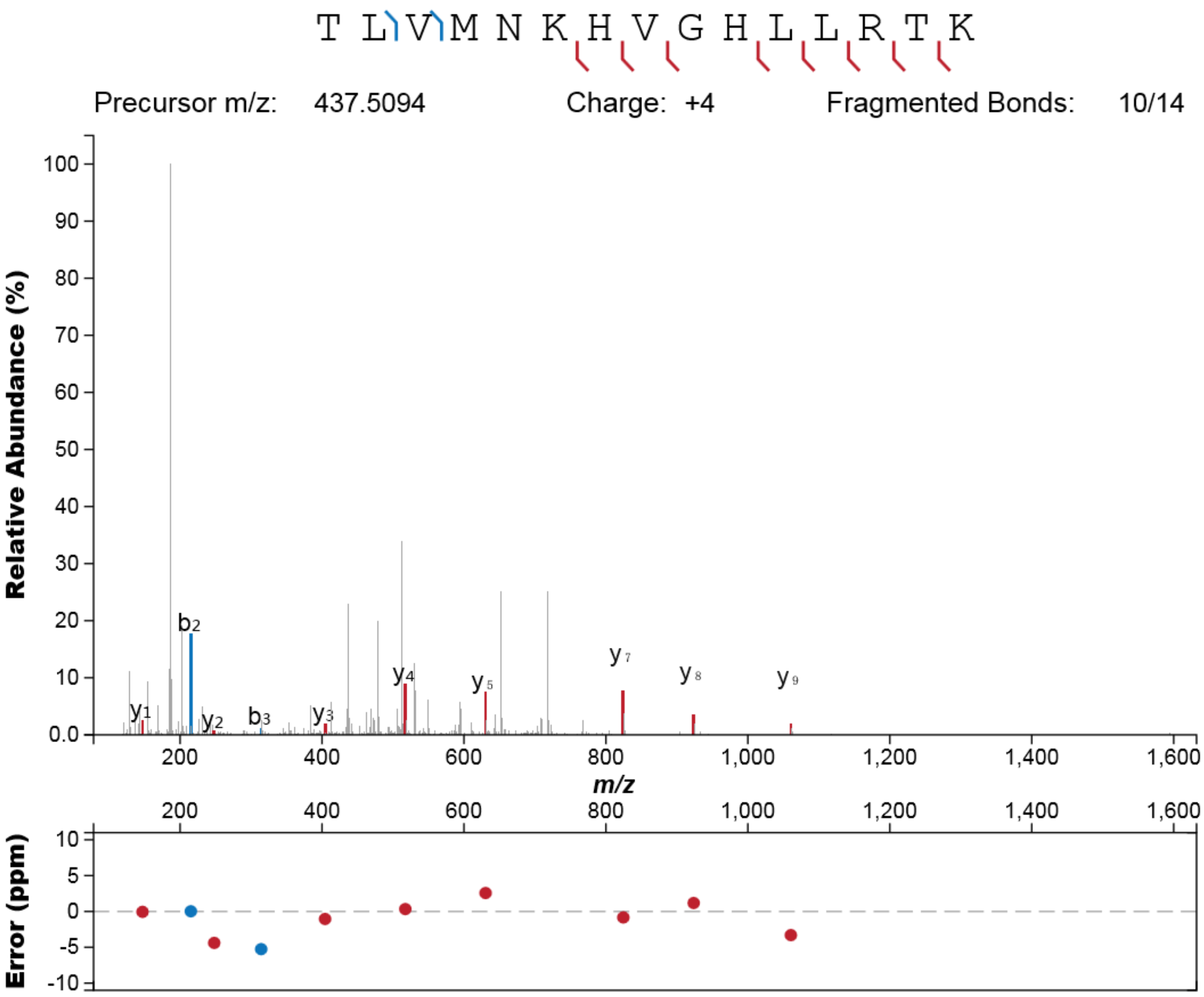
T L V M N K H V G H L L R T K
Precursor m/z: 437.5094
Charge: +4
Fragmented Bonds: 10/14
Relative Abundance (%)
m/z
Error (ppm)
b2
b3
y1
y2
y3
y4
y5
y7
y8
y9

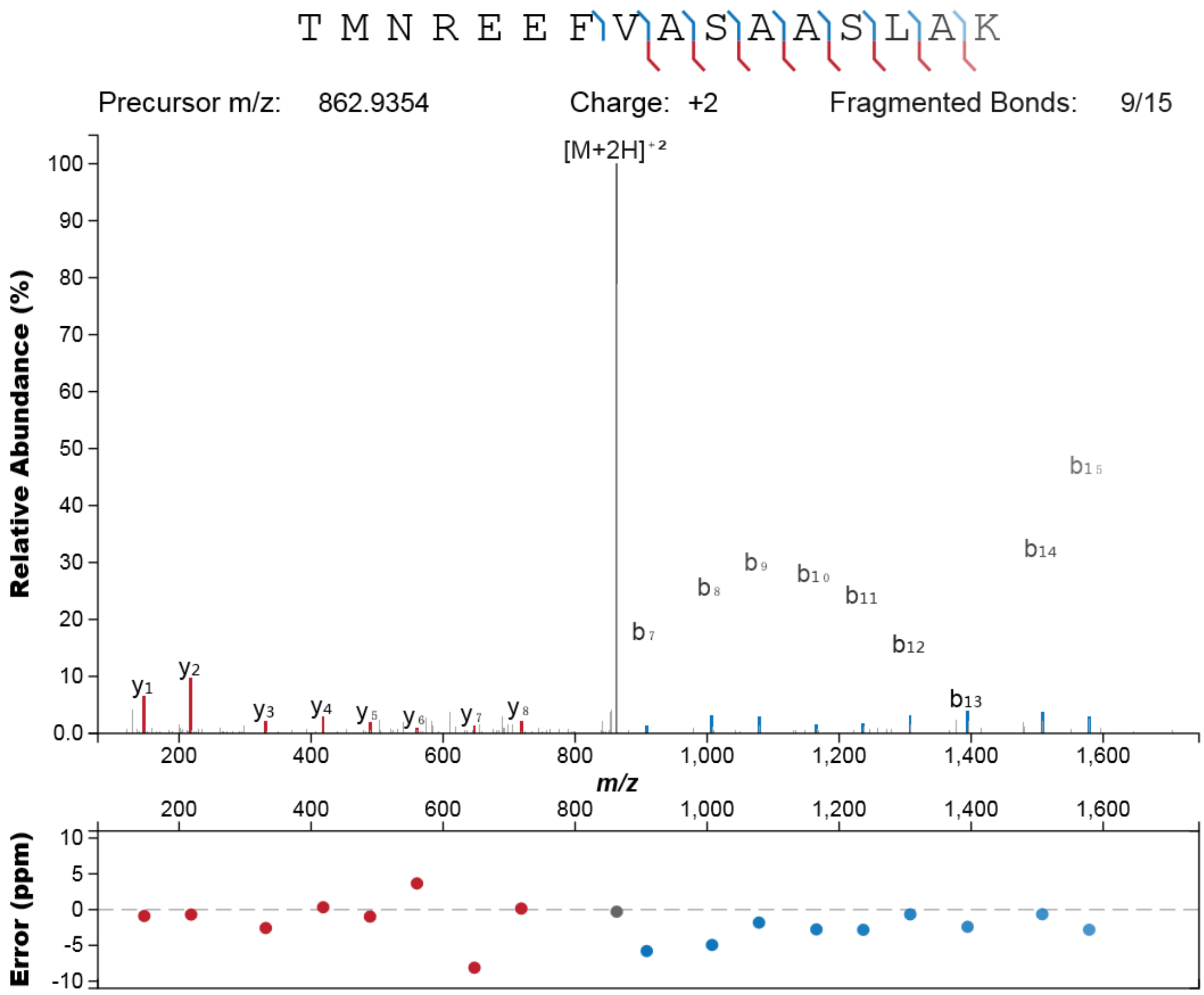
T M N R E E F V A S A A S L A K
Precursor m/z: 862.9354
Charge: +2
Fragmented Bonds: 9/15
[M+2H]+2
Relative Abundance (%)
m/z
Error (ppm)
y1
y2
y3
y4
y5
y6
y7
y8
b7
b8
b9
b10
b11
b12
b13
b14
b15

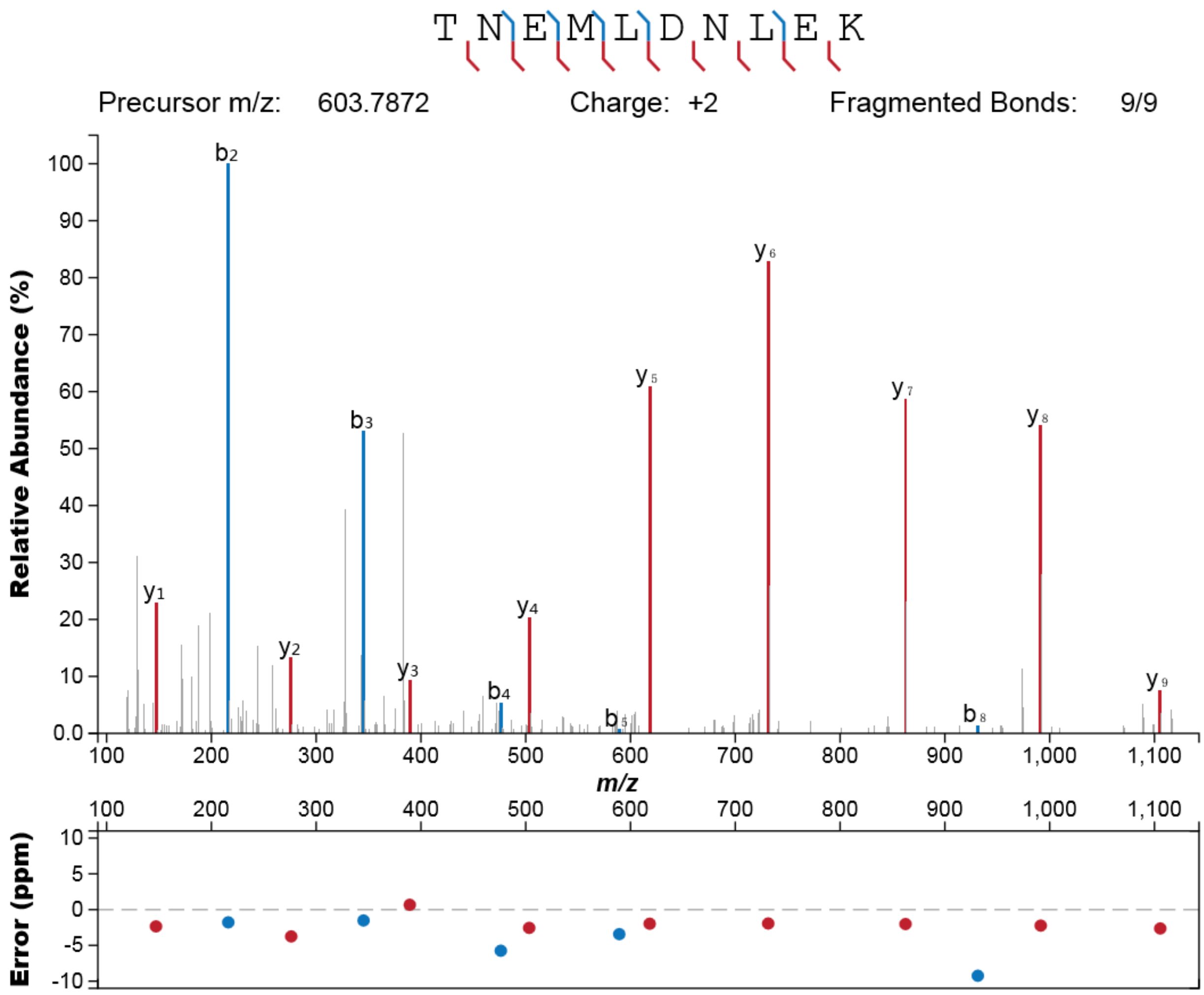

T N E M L D N L E K
Precursor m/z: 603.7872
Charge: +2
Fragmented Bonds: 9/9
Relative Abundance (%)
m/z
Error (ppm)
b2
b3
b4
b5
b8
y1
y2
y3
y4
y5
y6
y7
y8
y9

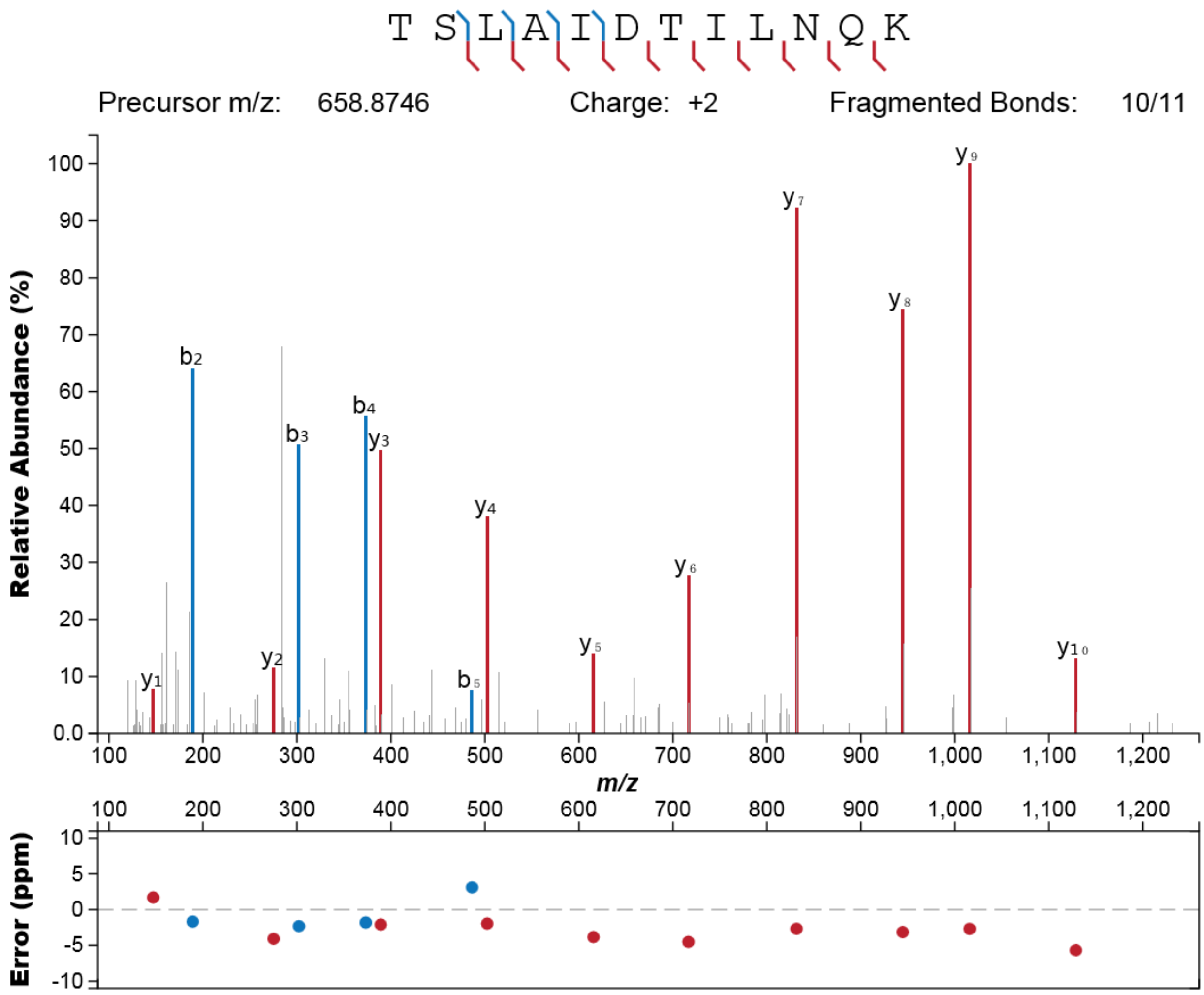
T S L A I D T I L N Q K
Precursor m/z: 658.8746
Charge: +2
Fragmented Bonds: 10/11
Relative Abundance (%)
m/z
Error (ppm)
b2
b3
b4
b5
y1
y2
y3
y4
y5
y6
y7
y8
y9
y10

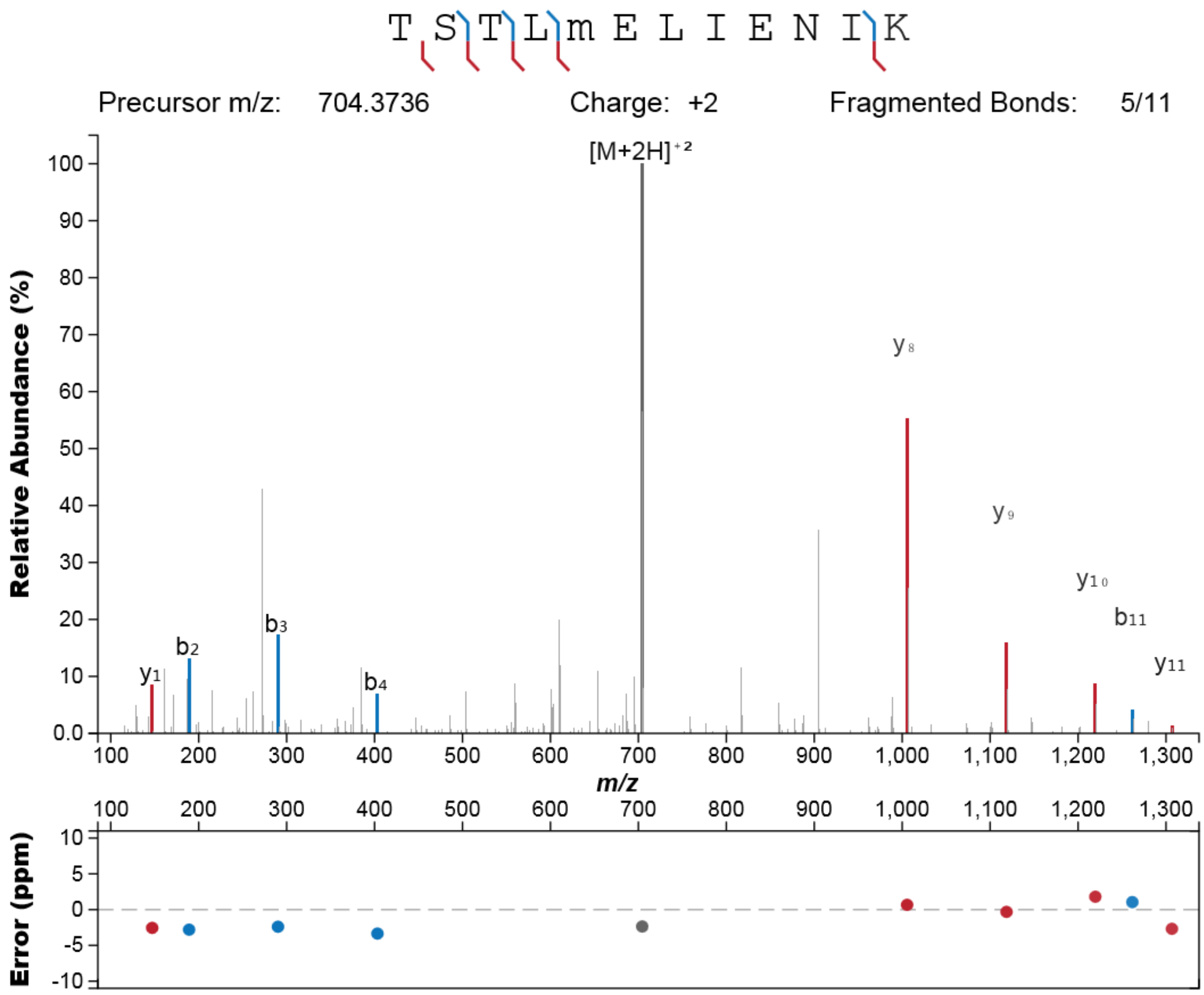

T S T L m E L I E N I K
Precursor m/z: 704.3736
Charge: +2
Fragmented Bonds: 5/11
[M+2H]+2
y8
y9
y10
b11
y11
y1
b2
b3
b4
Relative Abundance (%)
m/z
Error (ppm)

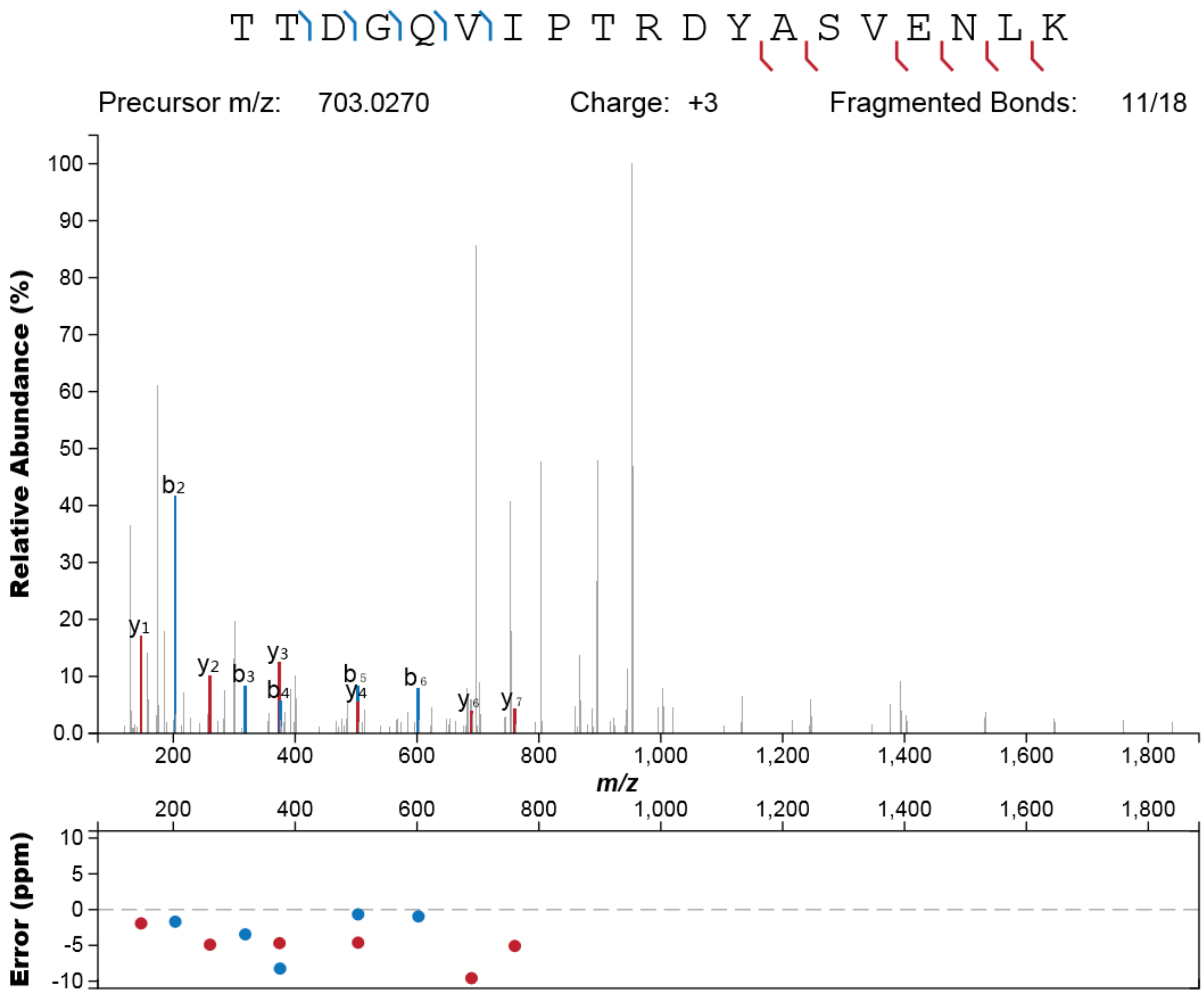

T T D G Q V I P T R D Y A S V E N L K
Precursor m/z: 703.0270
Charge: +3
Fragmented Bonds: 11/18
Relative Abundance (%)
m/z
Error (ppm)
y1
b2
y2
b3
y3
b4
b5
y4
b6
y6
y7

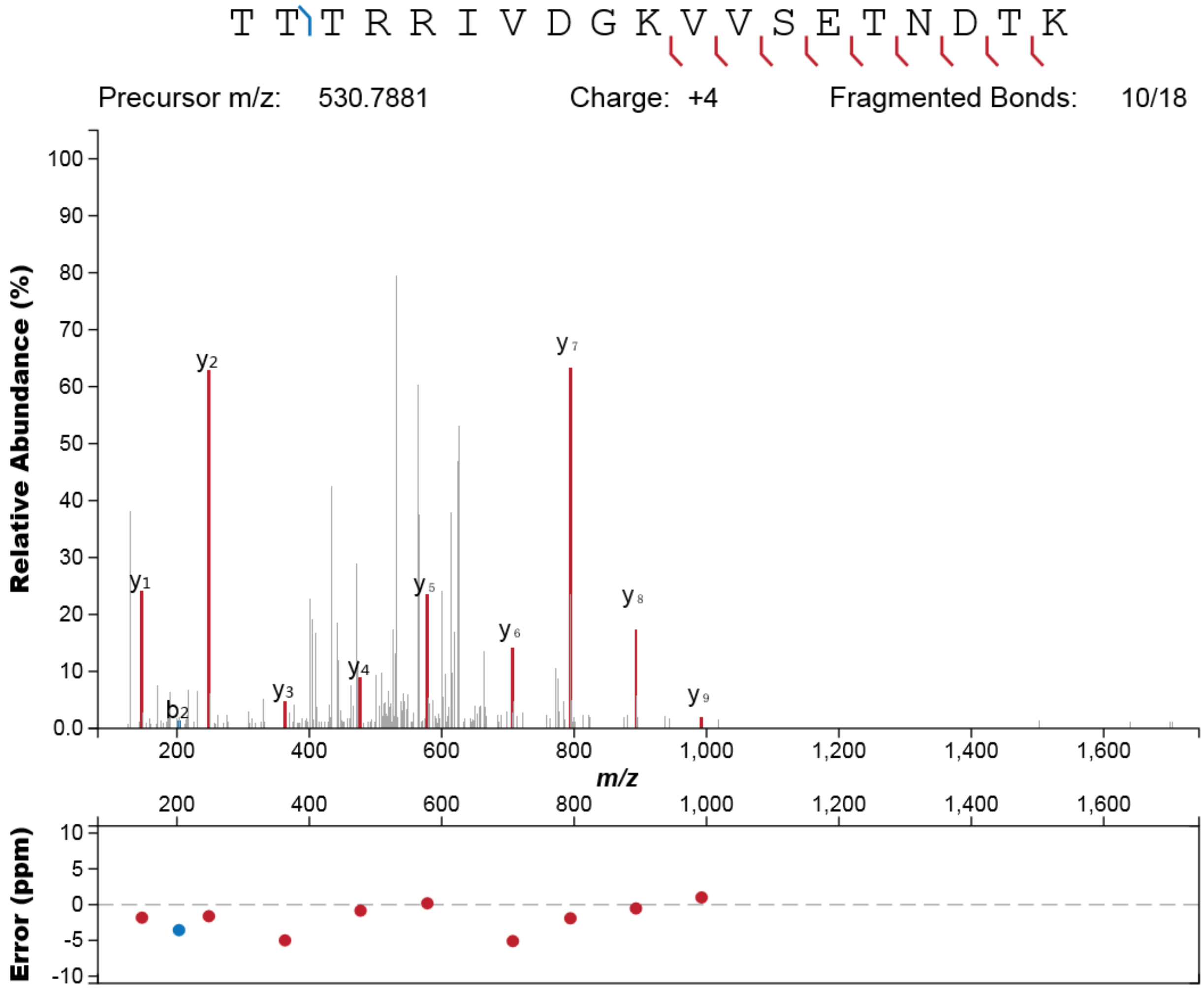

T T T R R I V D G K V V S E T N D T K
Precursor m/z: 530.7881
Charge: +4
Fragmented Bonds: 10/18
Relative Abundance (%)
m/z
Error (ppm)
y1
y2
y3
y4
y5
y6
y7
y8
y9
b2

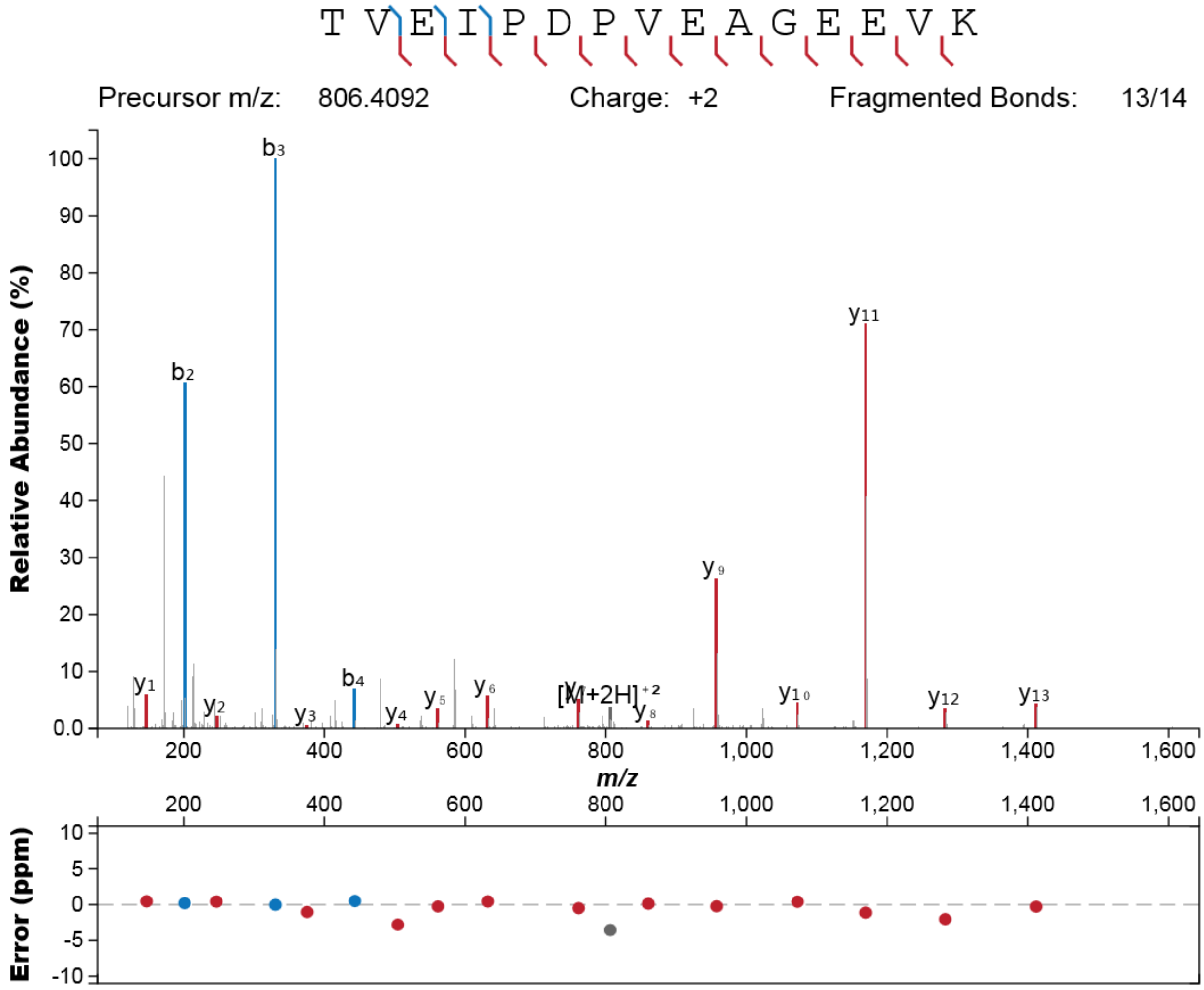
T V E I P D P V E A G E E V K
Precursor m/z: 806.4092
Charge: +2
Fragmented Bonds: 13/14
Relative Abundance (%)
b2
b3
b4
y1
y2
y3
y4
y5
y6
y8
y9
y10
y11
y12
y13
[M+2H]+2
m/z
Error (ppm)

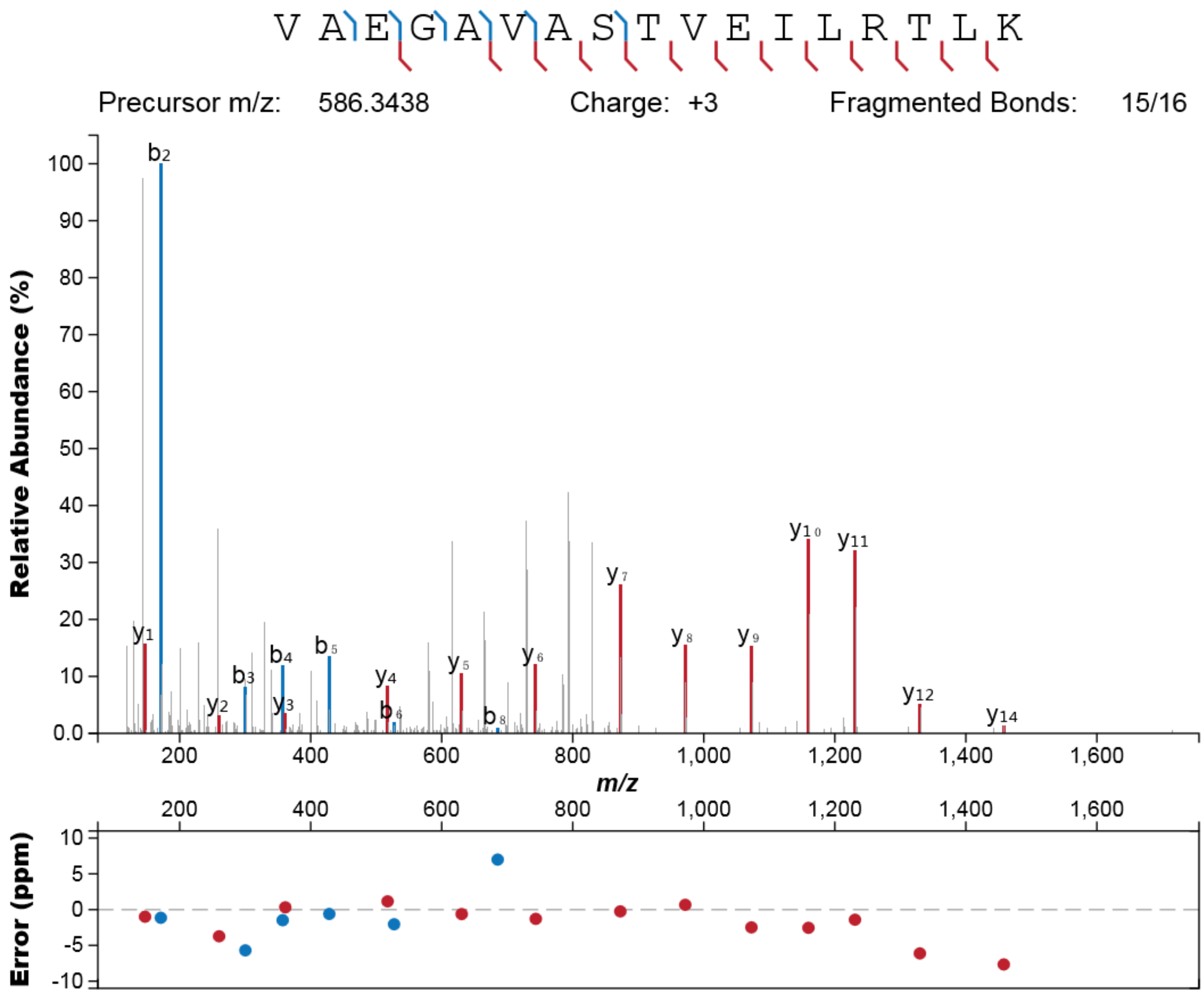
V A E G A V A S T V E I L R T L K
Precursor m/z: 586.3438
Charge: +3
Fragmented Bonds: 15/16
Relative Abundance (%)
m/z
Error (ppm)
b2
b3
b4
b5
b6
b8
y1
y2
y3
y4
y5
y6
y7
y8
y9
y10
y11
y12
y14

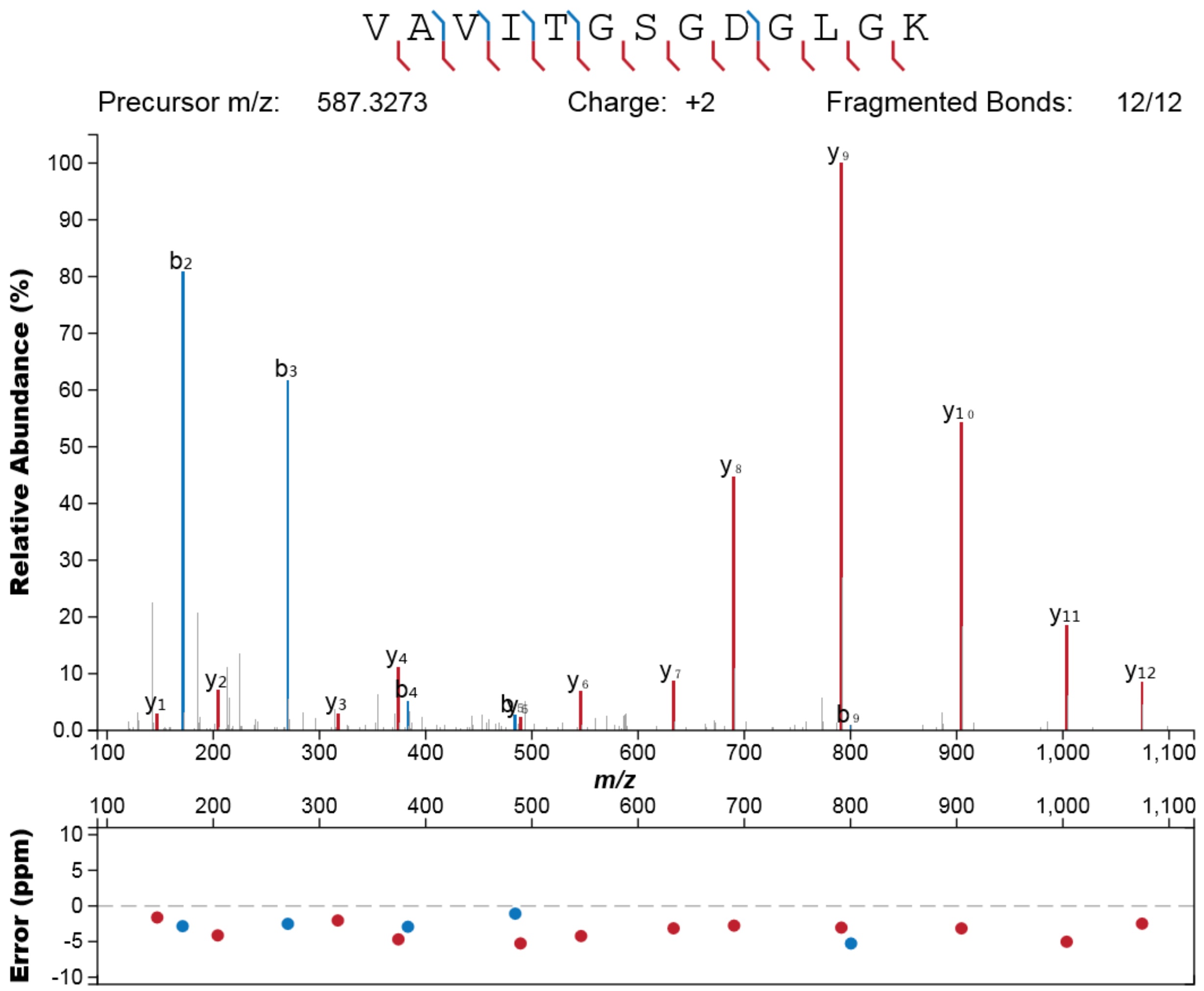

V A V I T G S G D G L G K
Precursor m/z: 587.3273
Charge: +2
Fragmented Bonds: 12/12
Relative Abundance (%)
m/z
Error (ppm)
b2
b3
b4
b9
y1
y2
y3
y4
y6
y7
y8
y9
y10
y11
y12

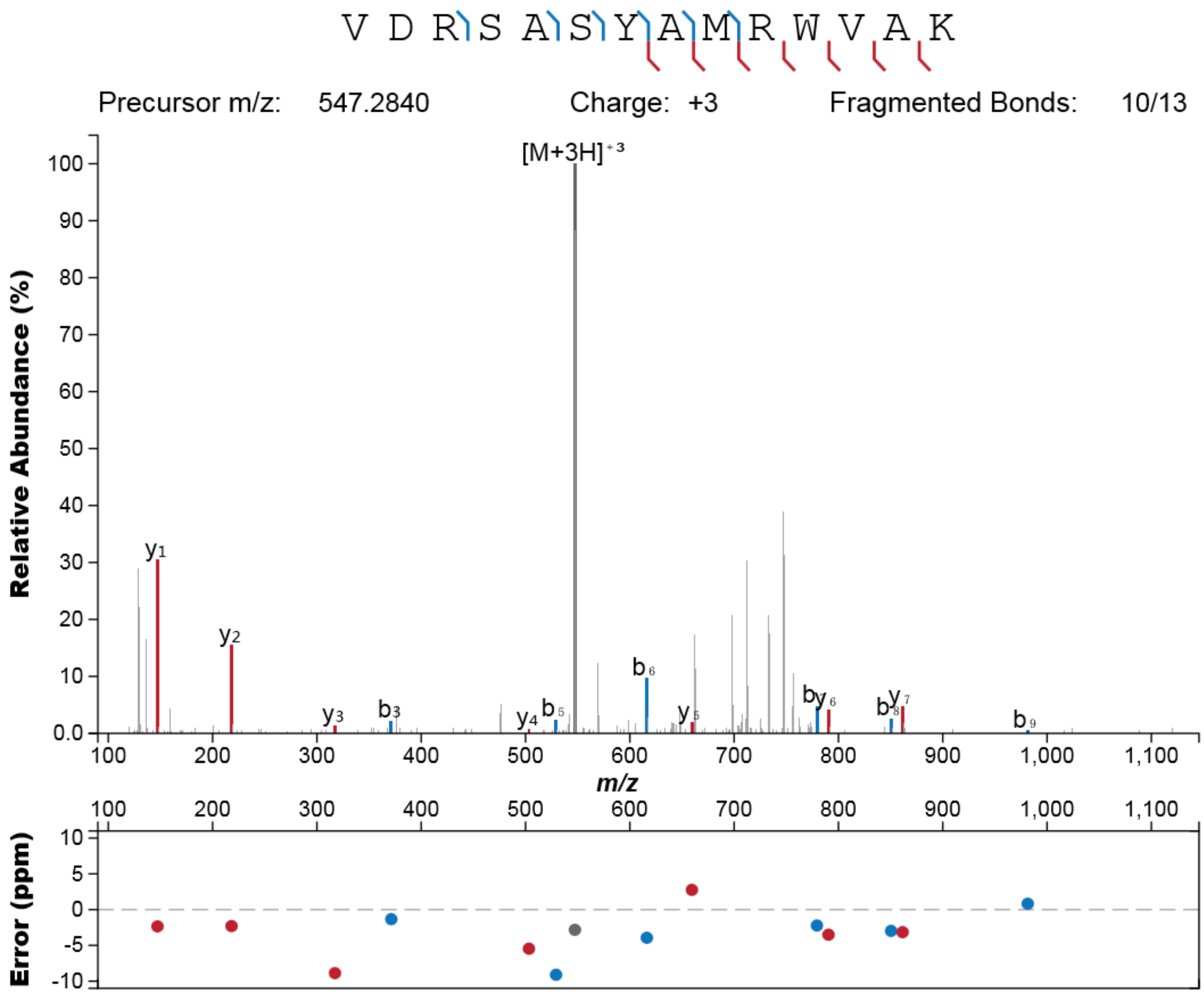
V D R S A S Y A M R W V A K
Precursor m/z: 547.2840
Charge: +3
Fragmented Bonds: 10/13
[M+3H]+3
Relative Abundance (%)
m/z
Error (ppm)
y1
y2
y3
b3
y4
b5
b6
y5
b y6
b8
y7
b9

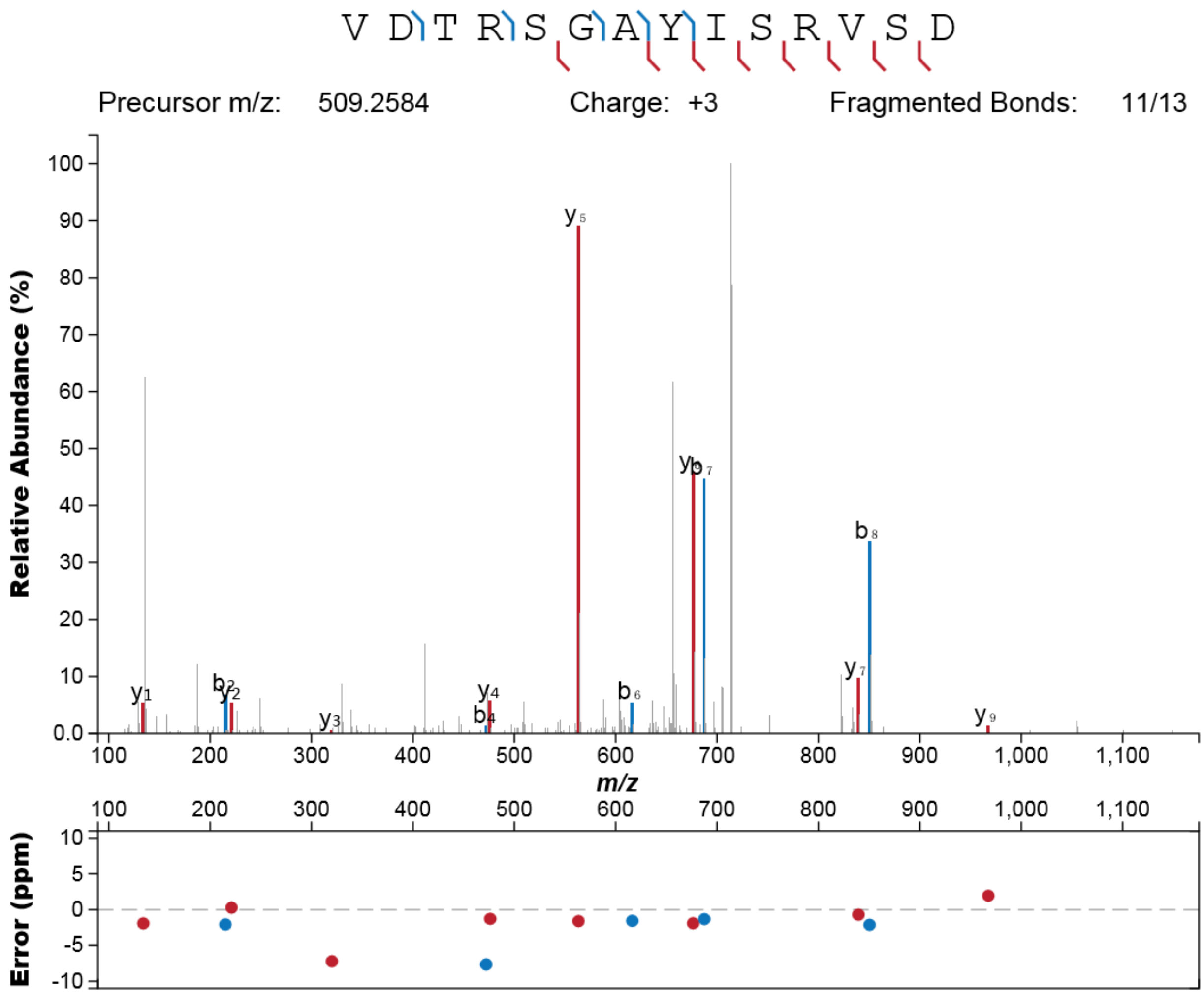

V D T R S G A Y I S R V S D
Precursor m/z: 509.2584
Charge: +3
Fragmented Bonds: 11/13
Relative Abundance (%)
m/z
Error (ppm)
y1
b2
y2
y3
y4
b4
y5
b6
y6
b7
y7
b8
y9

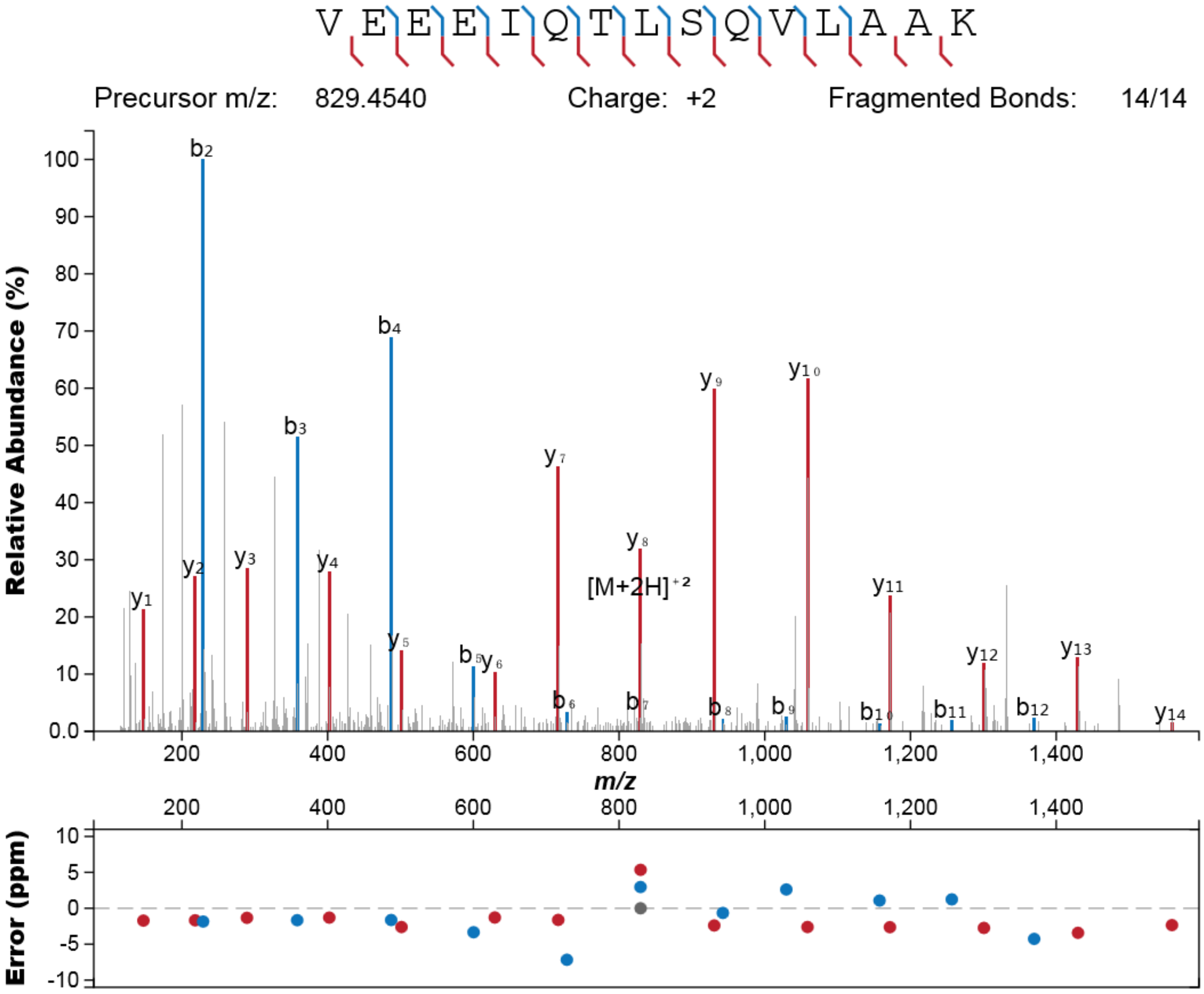
V E E E I Q T L S Q V L A A K
Precursor m/z: 829.4540
Charge: +2
Fragmented Bonds: 14/14
Relative Abundance (%)
m/z
Error (ppm)
[M+2H]+2

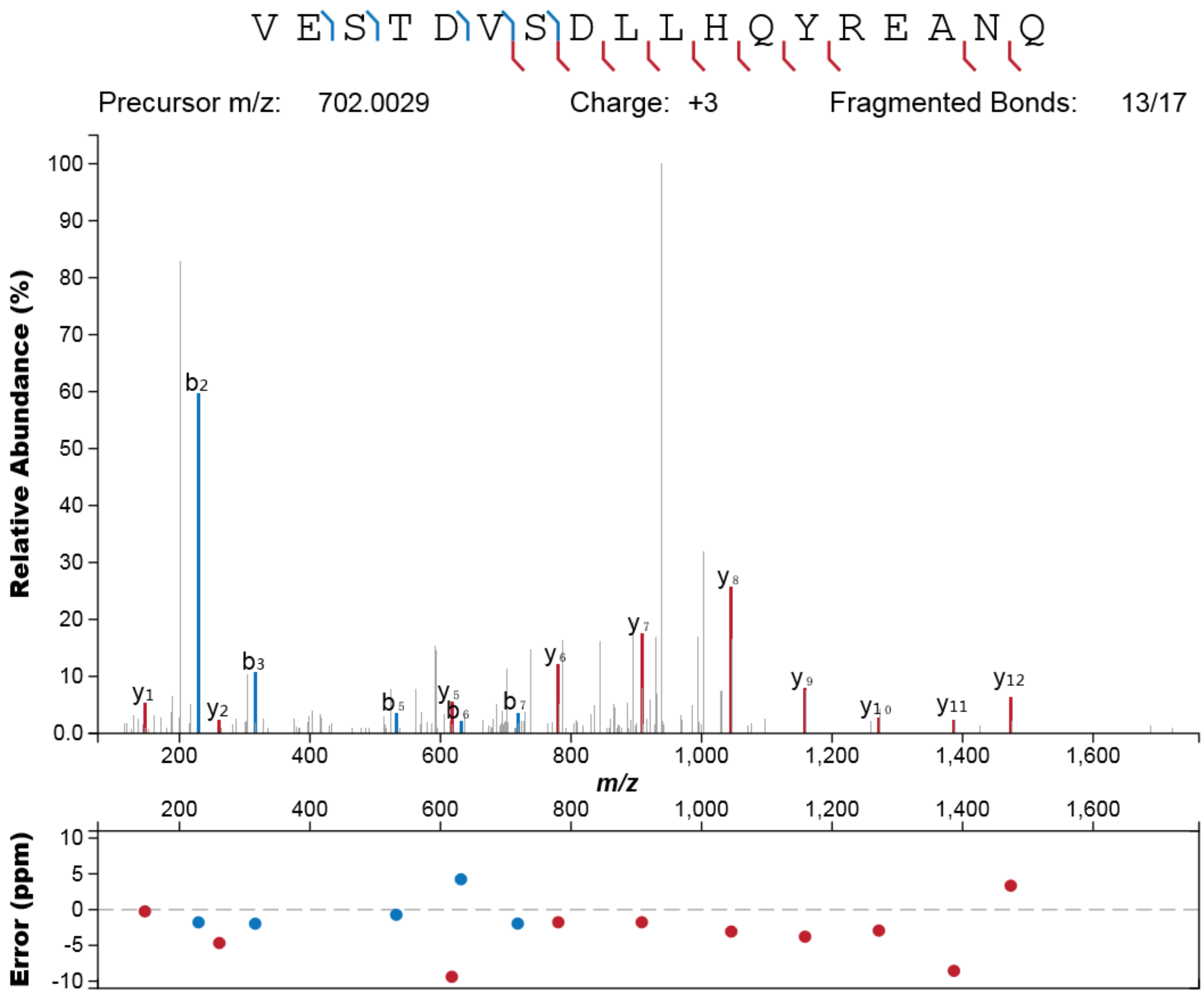
V E S T D V S D L L H Q Y R E A N Q
Precursor m/z: 702.0029
Charge: +3
Fragmented Bonds: 13/17
Relative Abundance (%)
m/z
Error (ppm)
b2
b3
b5
b6
b7
y1
y2
y5
y6
y7
y8
y9
y10
y11
y12

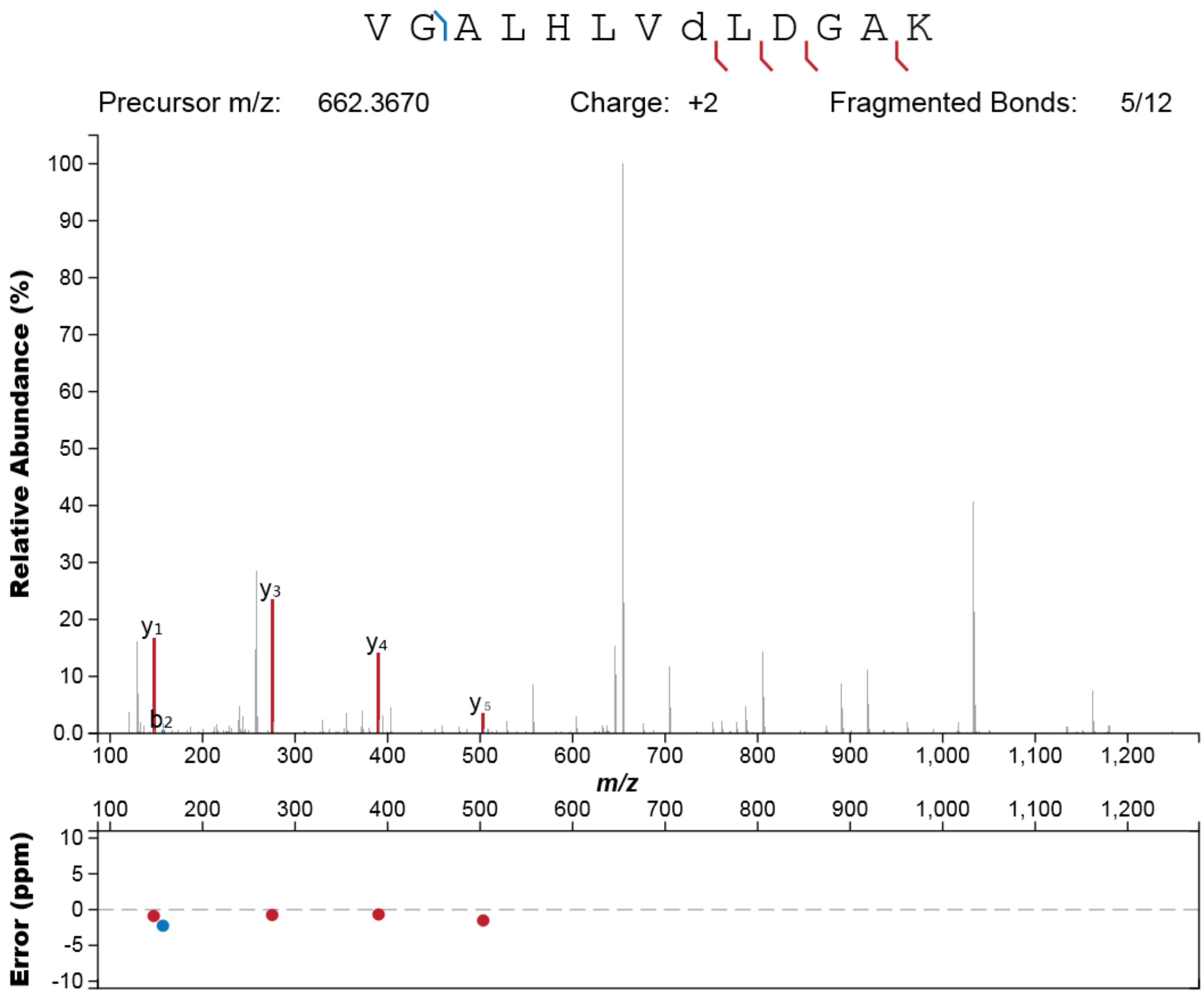
V G A L H L V d L D G A K
Precursor m/z: 662.3670
Charge: +2
Fragmented Bonds: 5/12
Relative Abundance (%)
y1
b2
y3
y4
y5
m/z
Error (ppm)

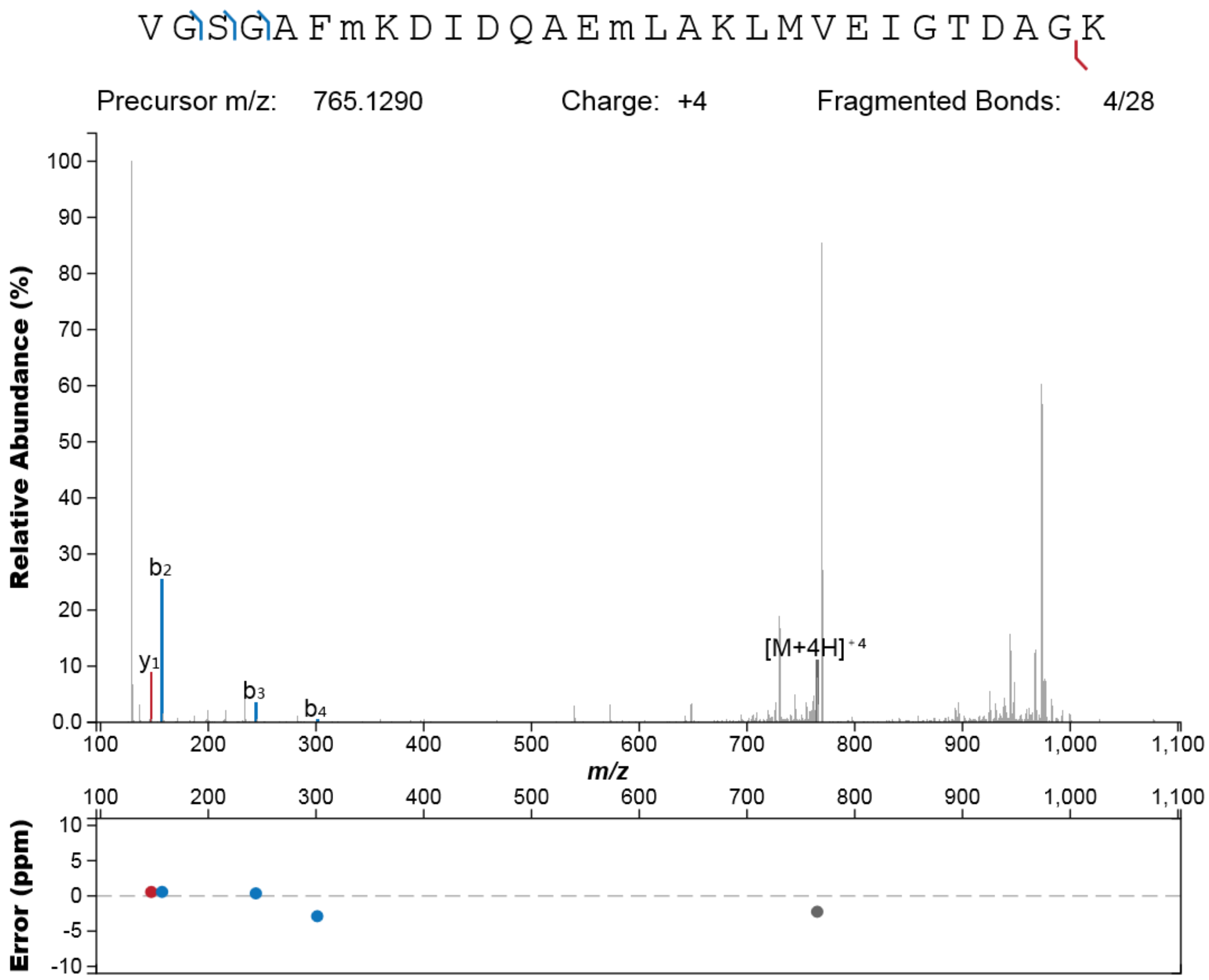
V G S G A F m K D I D Q A E m L A K L M V E I G T D A G K
Precursor m/z: 765.1290
Charge: +4
Fragmented Bonds: 4/28
Relative Abundance (%)
b2
y1
b3
b4
[M+4H]+4
m/z
Error (ppm)

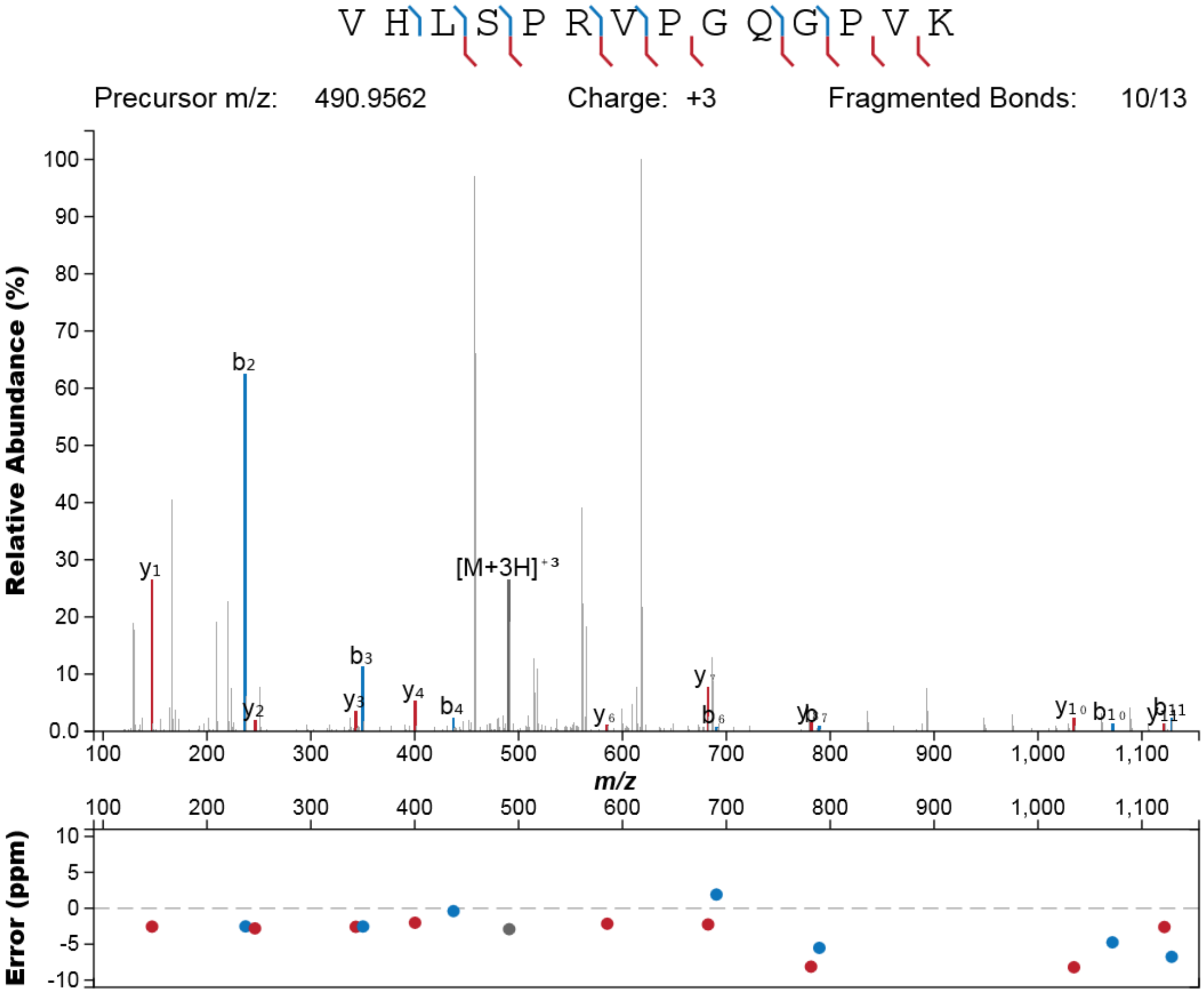
V H L S P R V P G Q G P V K
Precursor m/z: 490.9562
Charge: +3
Fragmented Bonds: 10/13
Relative Abundance (%)
m/z
Error (ppm)
[M+3H]+3
y1
b2
y2
b3
y3
y4
b4
y6
b6
y10
b10
b11

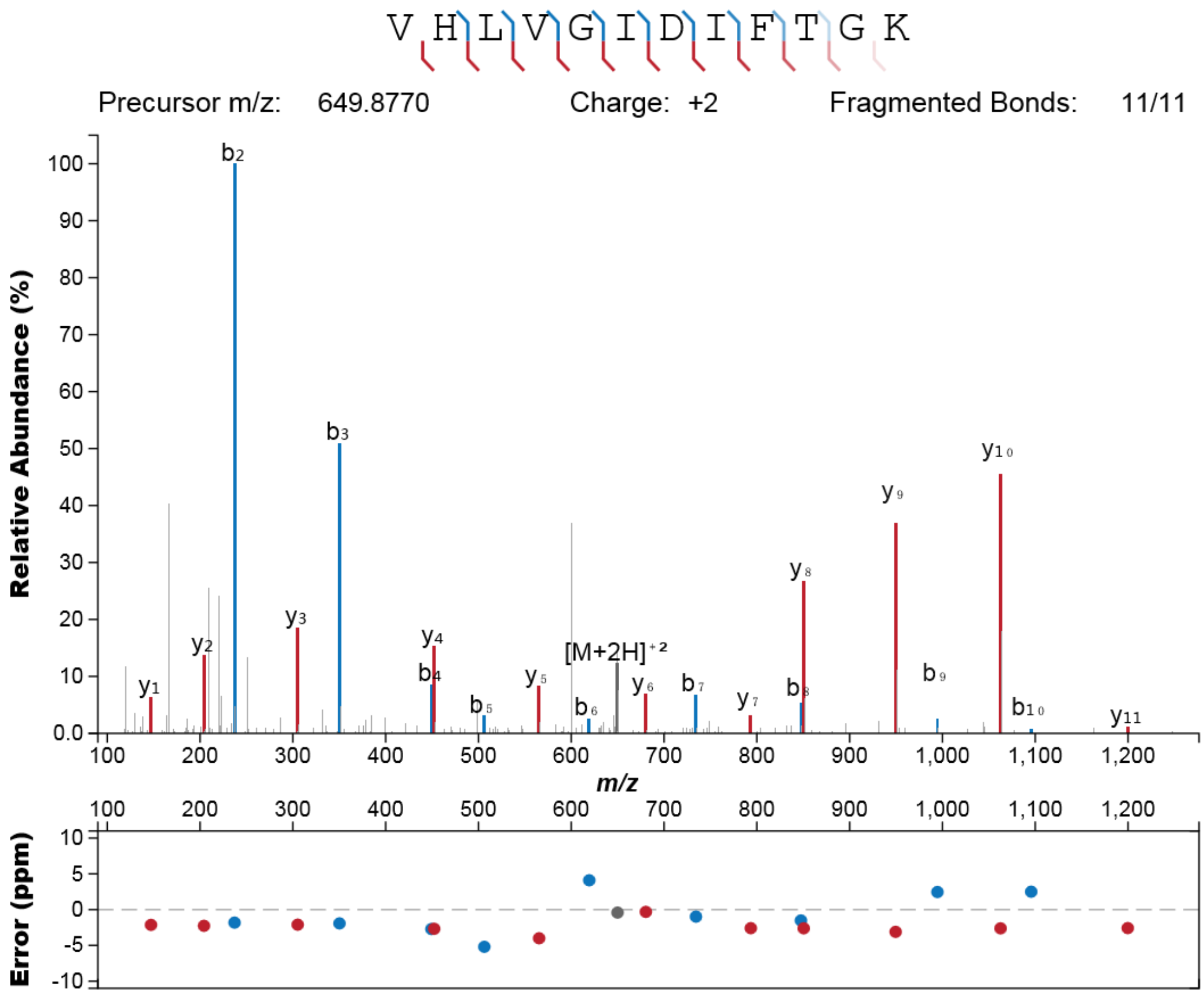
V H L V G I D I F T G K
Precursor m/z: 649.8770
Charge: +2
Fragmented Bonds: 11/11
Relative Abundance (%)
m/z
Error (ppm)
[M+2H]⁺²
b2 b3 b4 b5 b6 b7 b8 b9 b10
y1 y2 y3 y4 y5 y6 y7 y8 y9 y10 y11

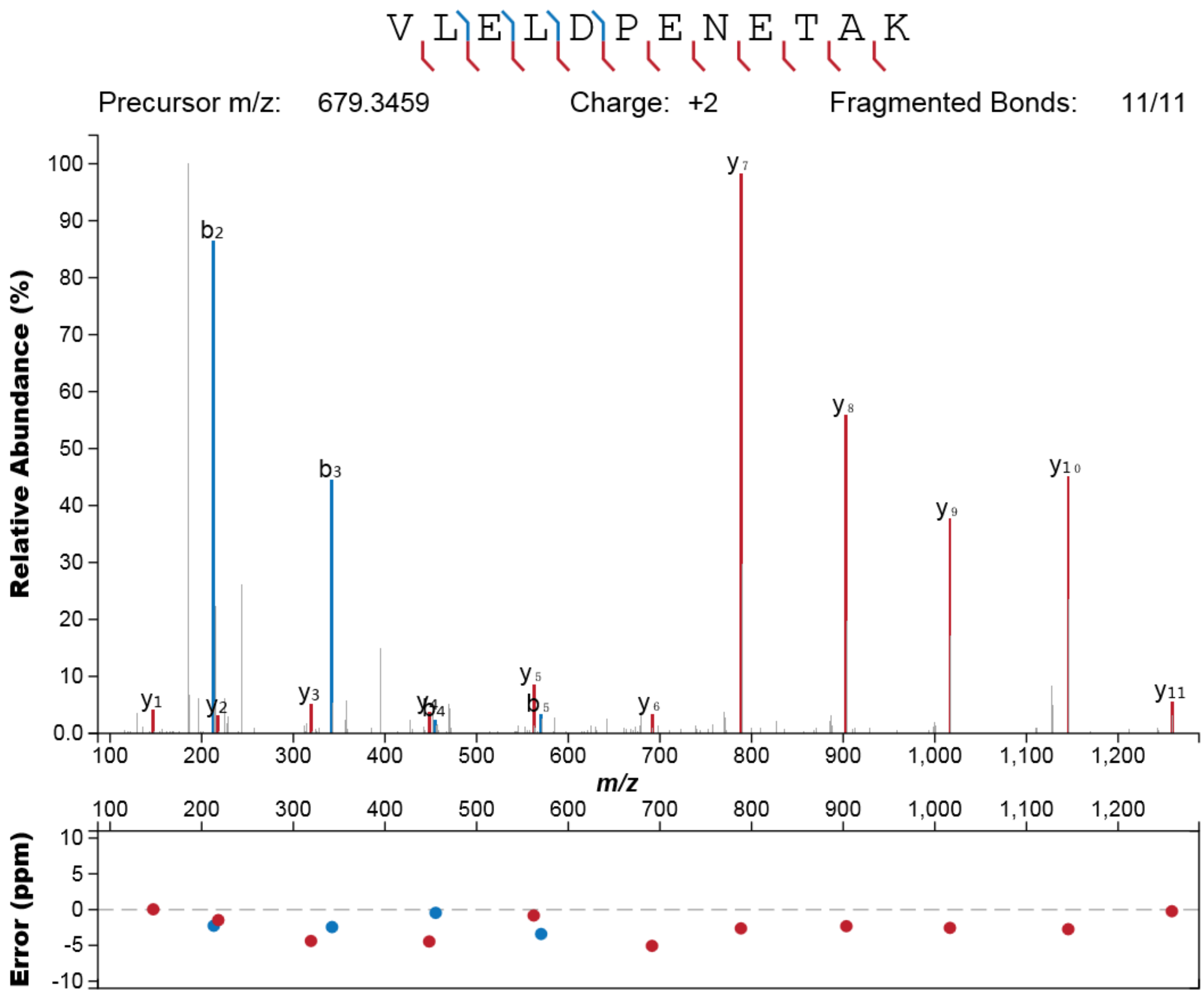

V L E L D P E N E T A K
Precursor m/z: 679.3459
Charge: +2
Fragmented Bonds: 11/11
Relative Abundance (%)
m/z
Error (ppm)
b2
b3
b4
b5
y1
y2
y3
y4
y5
y6
y7
y8
y9
y10
y11

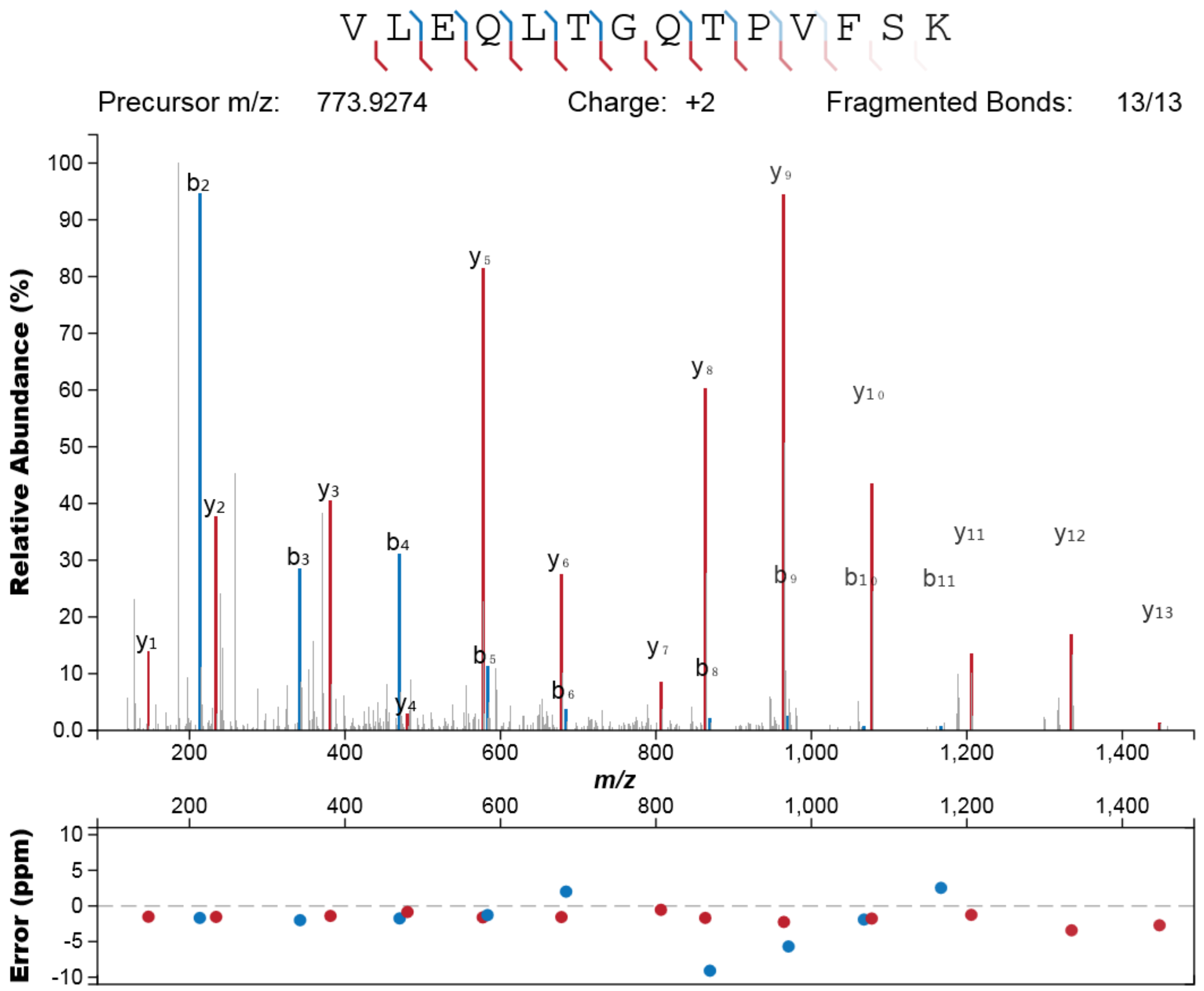

V L E Q L T G Q T P V F S K
Precursor m/z: 773.9274
Charge: +2
Fragmented Bonds: 13/13
Relative Abundance (%)
m/z
Error (ppm)
b2 b3 b4 b5 b6 b8 b9 b10 b11
y1 y2 y3 y4 y5 y6 y7 y8 y9 y10 y11 y12 y13

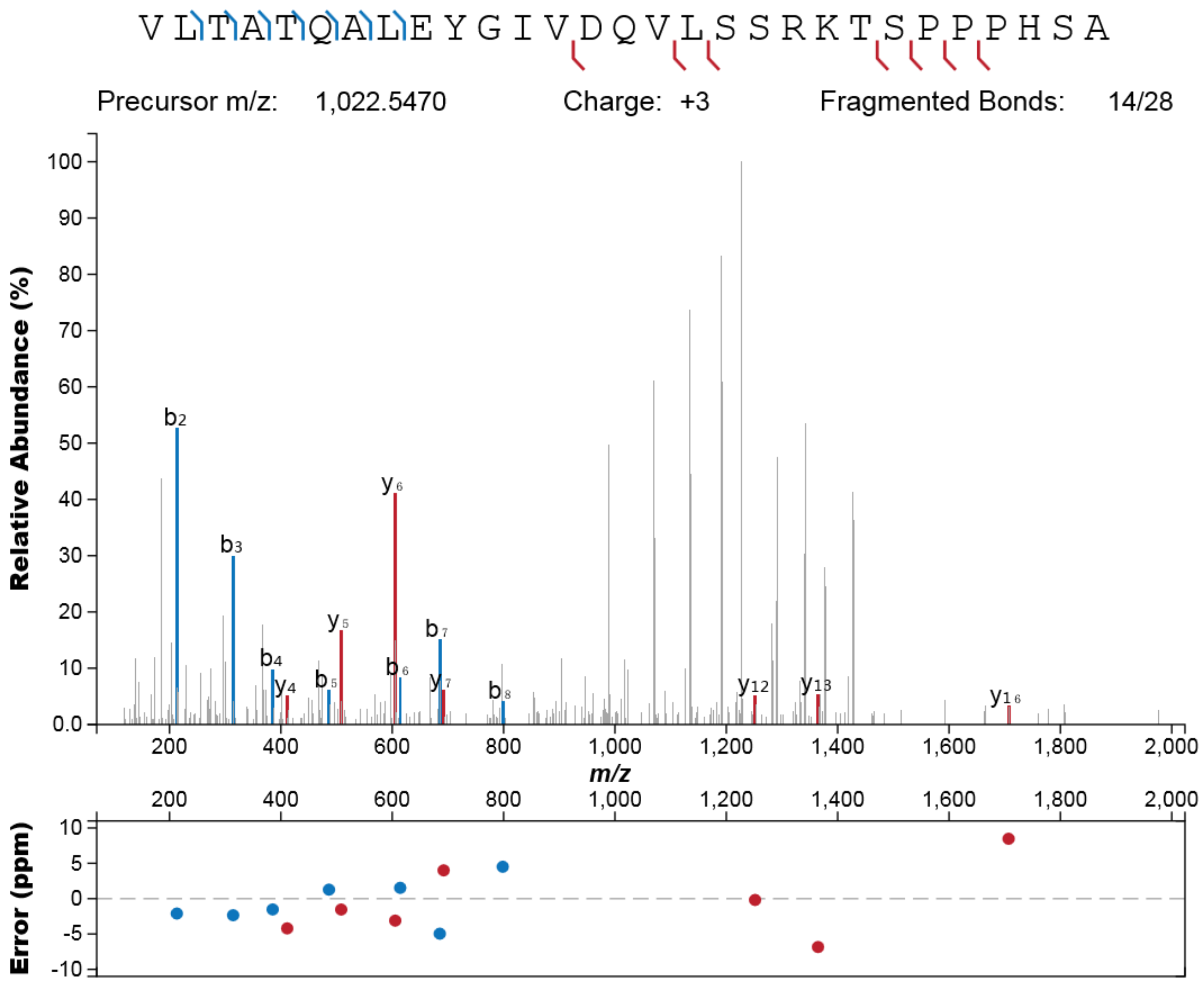

V L T A T Q A L E Y G I V D Q V L S S R K T S P P P H S A
Precursor m/z: 1,022.5470
Charge: +3
Fragmented Bonds: 14/28
Relative Abundance (%)
m/z
Error (ppm)
b2
b3
b4
y4
b5
y5
y6
b6
b7
y7
b8
y12
y13
y16

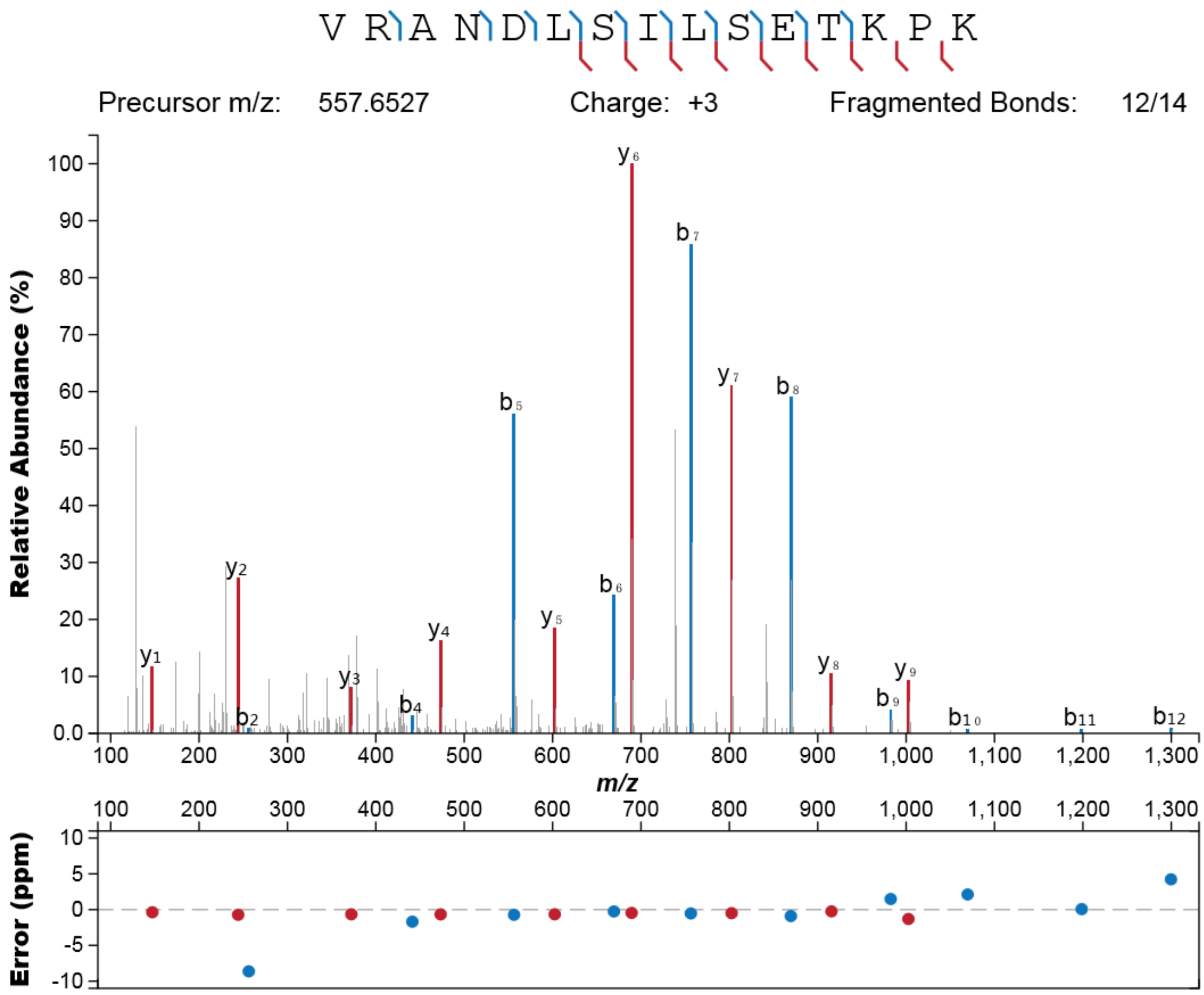

V R A N D L S I L S E T K P K
Precursor m/z: 557.6527
Charge: +3
Fragmented Bonds: 12/14
Relative Abundance (%)
m/z
Error (ppm)
y1 y2 b2 y3 b4 y4 b5 y5 b6 y6 b7 y7 b8 y8 b9 y9 b10 b11 b12

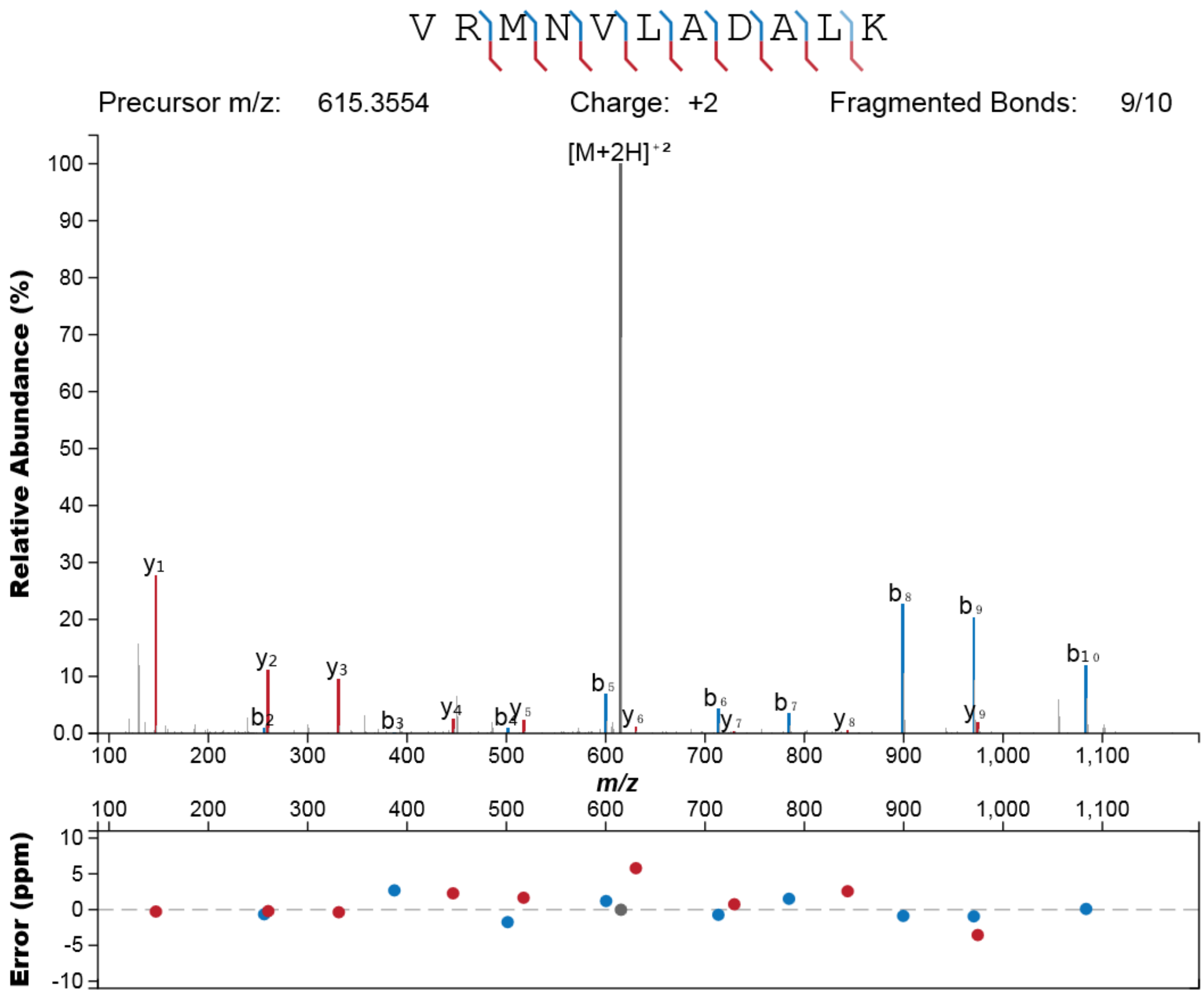

V R M N V L A D A L K
Precursor m/z: 615.3554
Charge: +2
Fragmented Bonds: 9/10
[M+2H]+2
Relative Abundance (%)
m/z
Error (ppm)
y1
y2
y3
y4
y5
y6
y7
y8
y9
b2
b3
b4
b5
b6
b7
b8
b9
b10

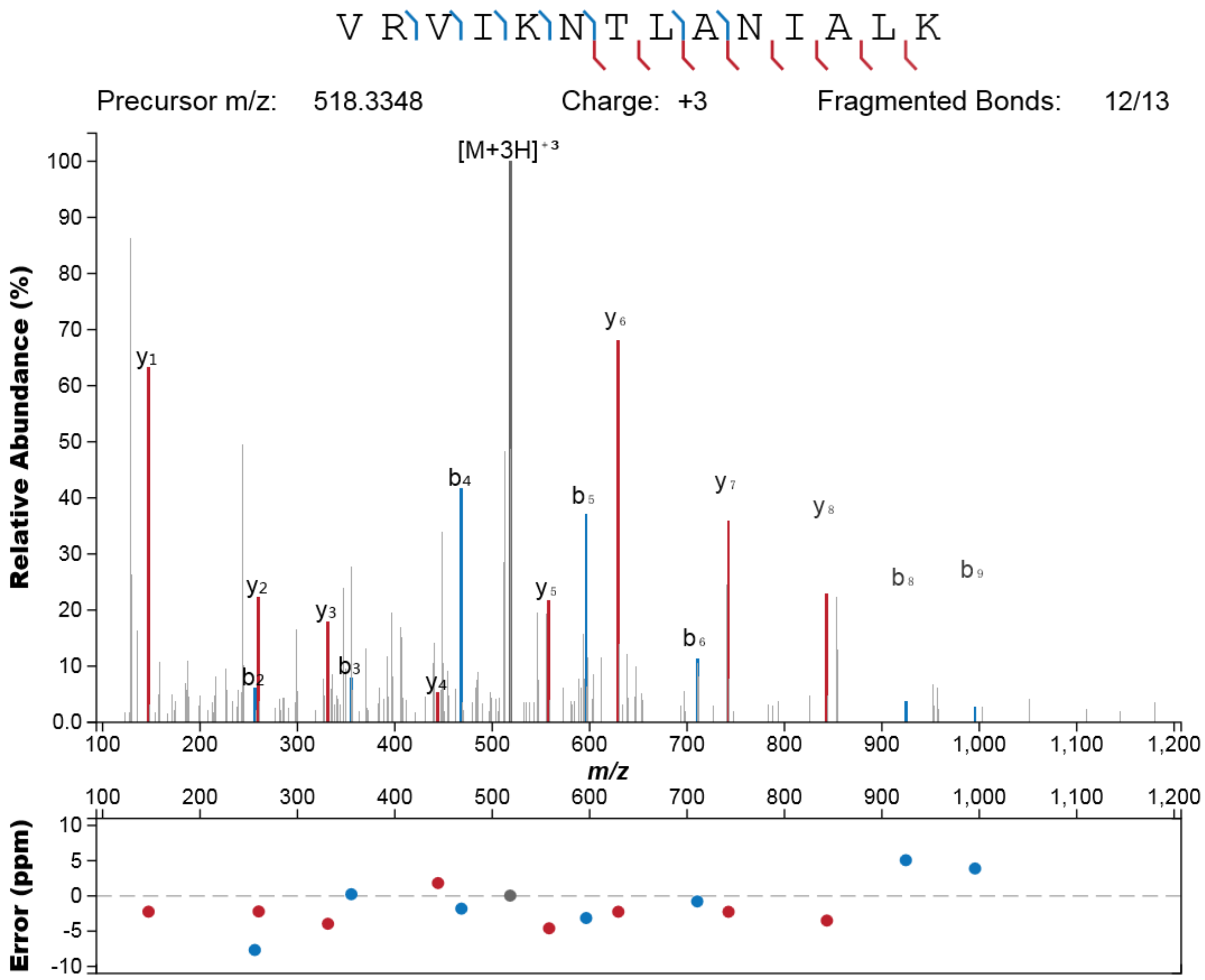

V R V I K N T L A N I A L K
Precursor m/z: 518.3348
Charge: +3
Fragmented Bonds: 12/13
[M+3H]+3
y1
y2
y3
y4
y5
y6
y7
y8
b2
b3
b4
b5
b6
b8
b9
Relative Abundance (%)
m/z
Error (ppm)

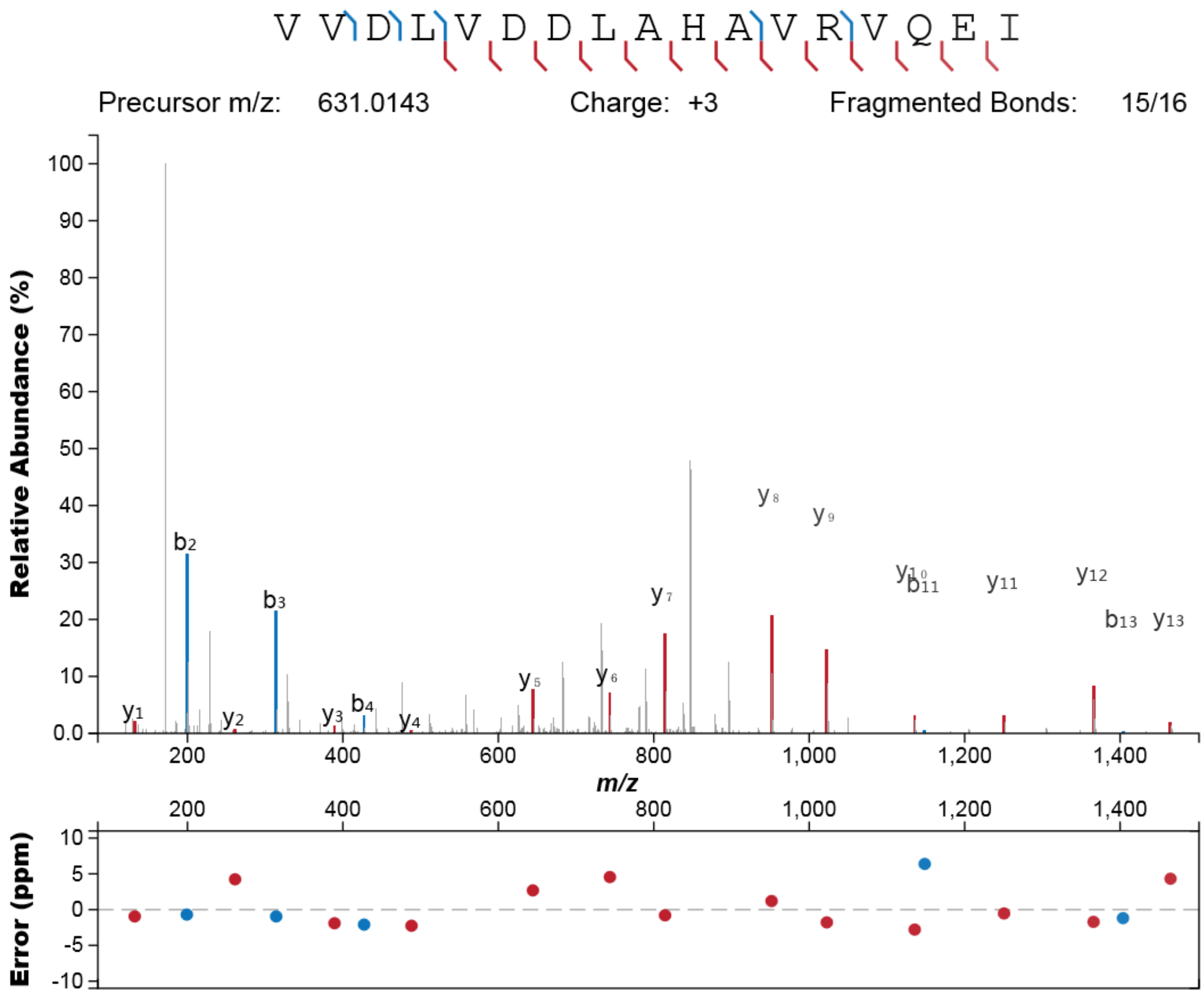
V V D L V D D L A H A V R V Q E I
Precursor m/z: 631.0143
Charge: +3
Fragmented Bonds: 15/16
Relative Abundance (%)
m/z
Error (ppm)
b2
b3
b4
b11
b13
y1
y2
y3
y4
y5
y6
y7
y8
y9
y10
y11
y12
y13

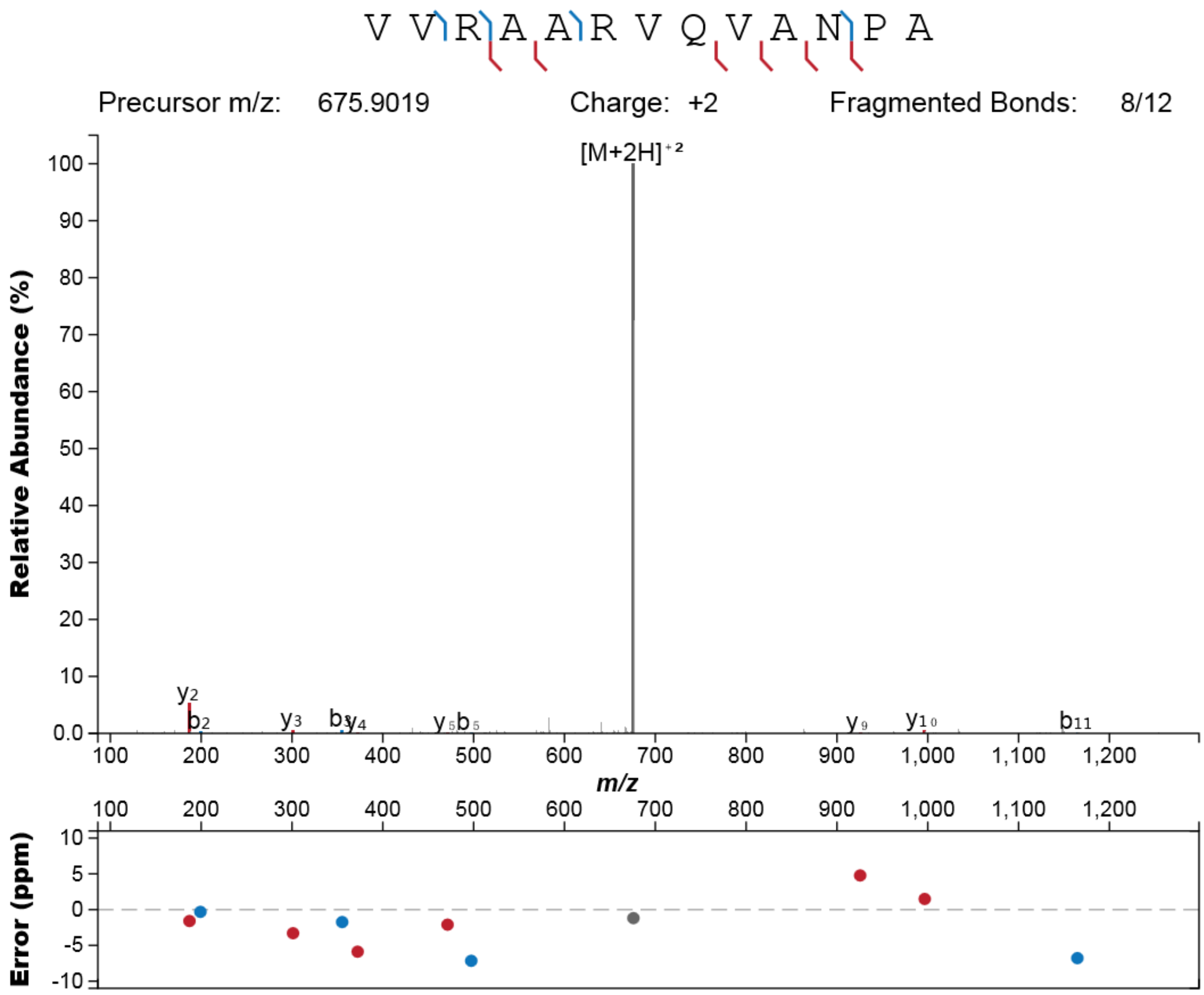
V V R A A R V Q V A N P A
Precursor m/z: 675.9019
Charge: +2
Fragmented Bonds: 8/12
[M+2H]+2
Relative Abundance (%)
y2
b2
y3
b3
y4
y5
b5
y9
y10
b11
m/z
Error (ppm)

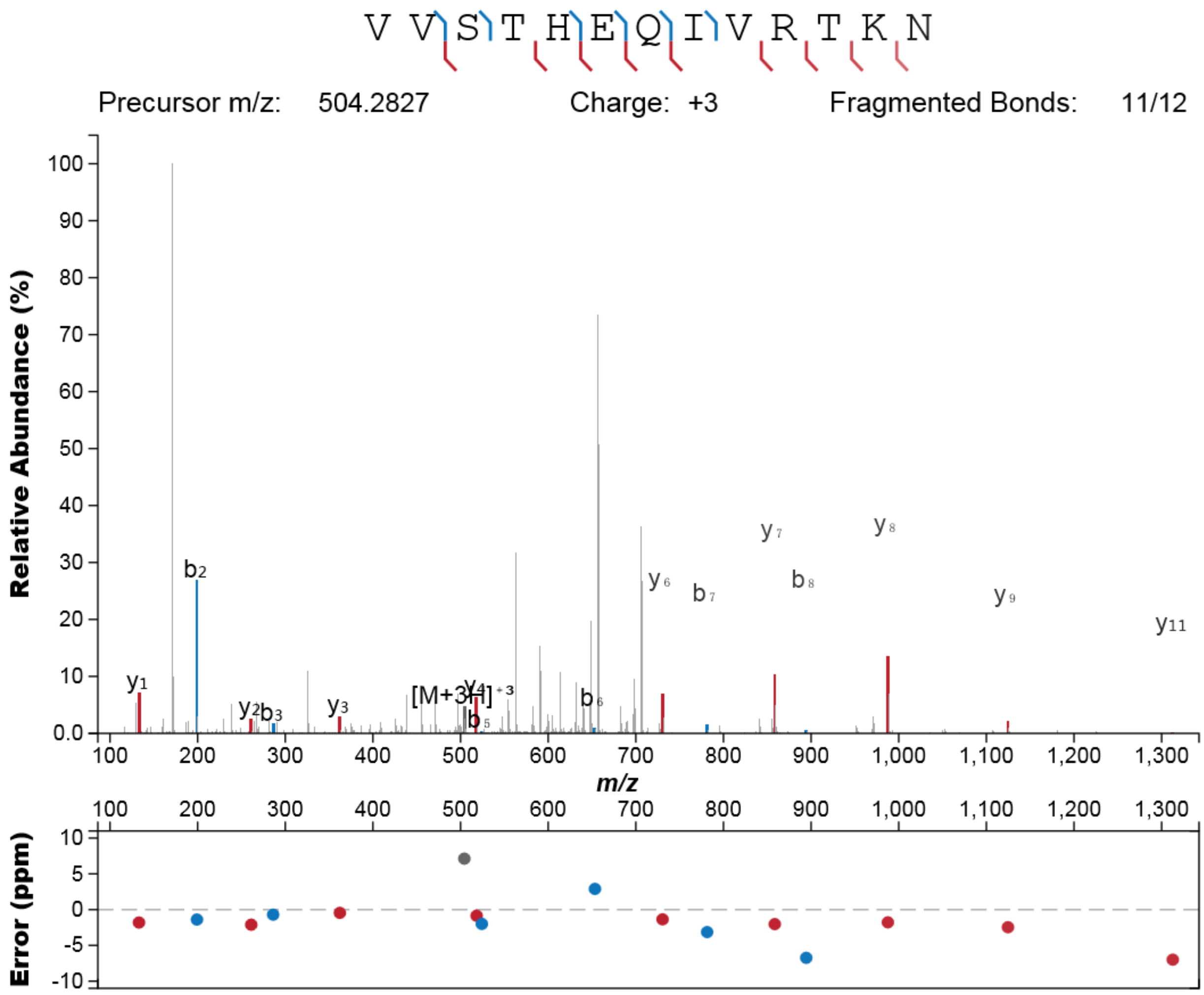

V V S T H E Q I V R T K N
Precursor m/z: 504.2827
Charge: +3
Fragmented Bonds: 11/12
Relative Abundance (%)
m/z
Error (ppm)
y1
b2
y2
b3
y3
[M+3H]+3
y4
b5
b6
y6
b7
y7
b8
y8
y9
y11

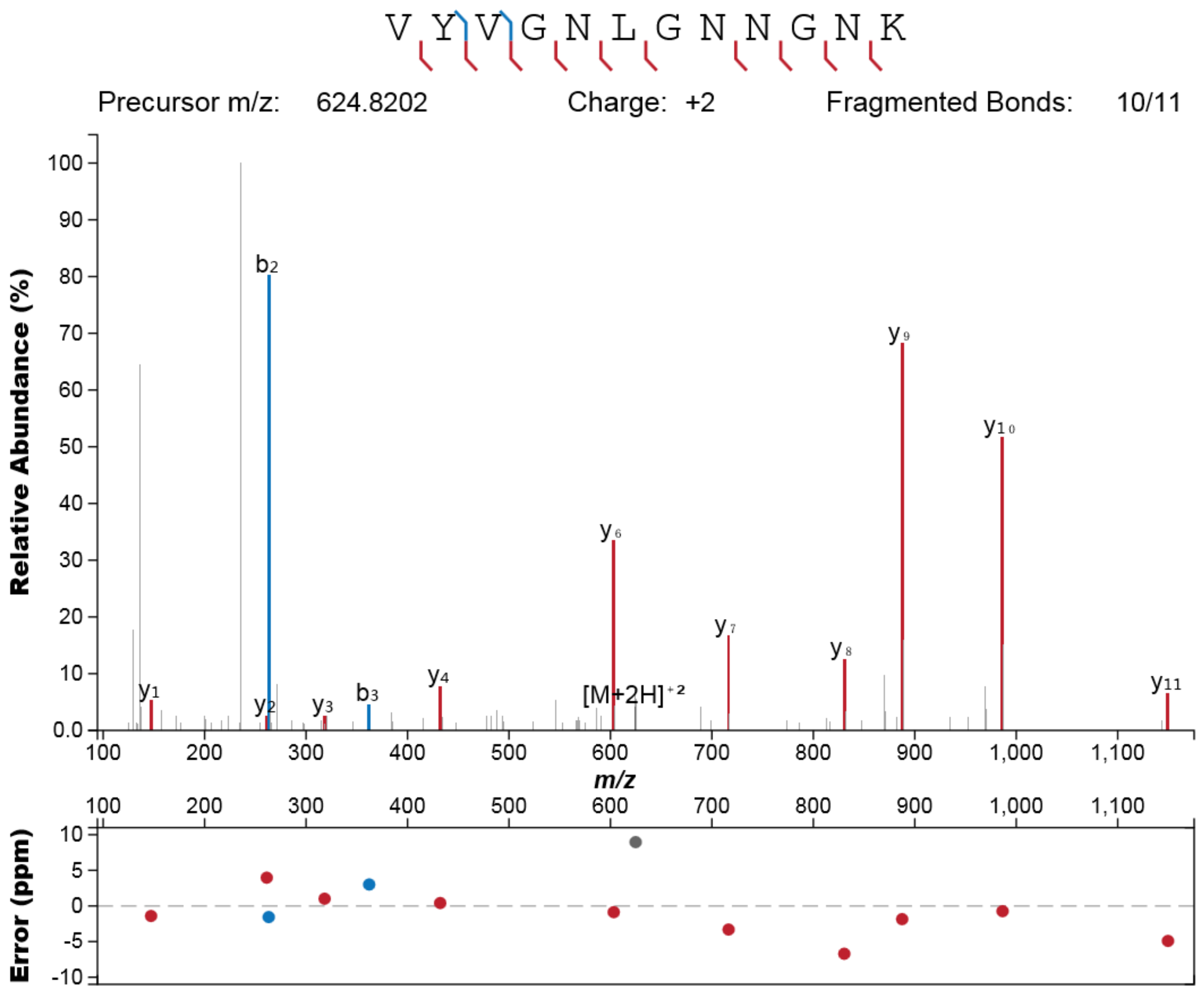
V Y V G N L G N N G N K
Precursor m/z: 624.8202
Charge: +2
Fragmented Bonds: 10/11
Relative Abundance (%)
b2
y9
y10
y6
y7
y8
y4
y1
y2
y3
b3
[M+2H]+2
y11
m/z
Error (ppm)

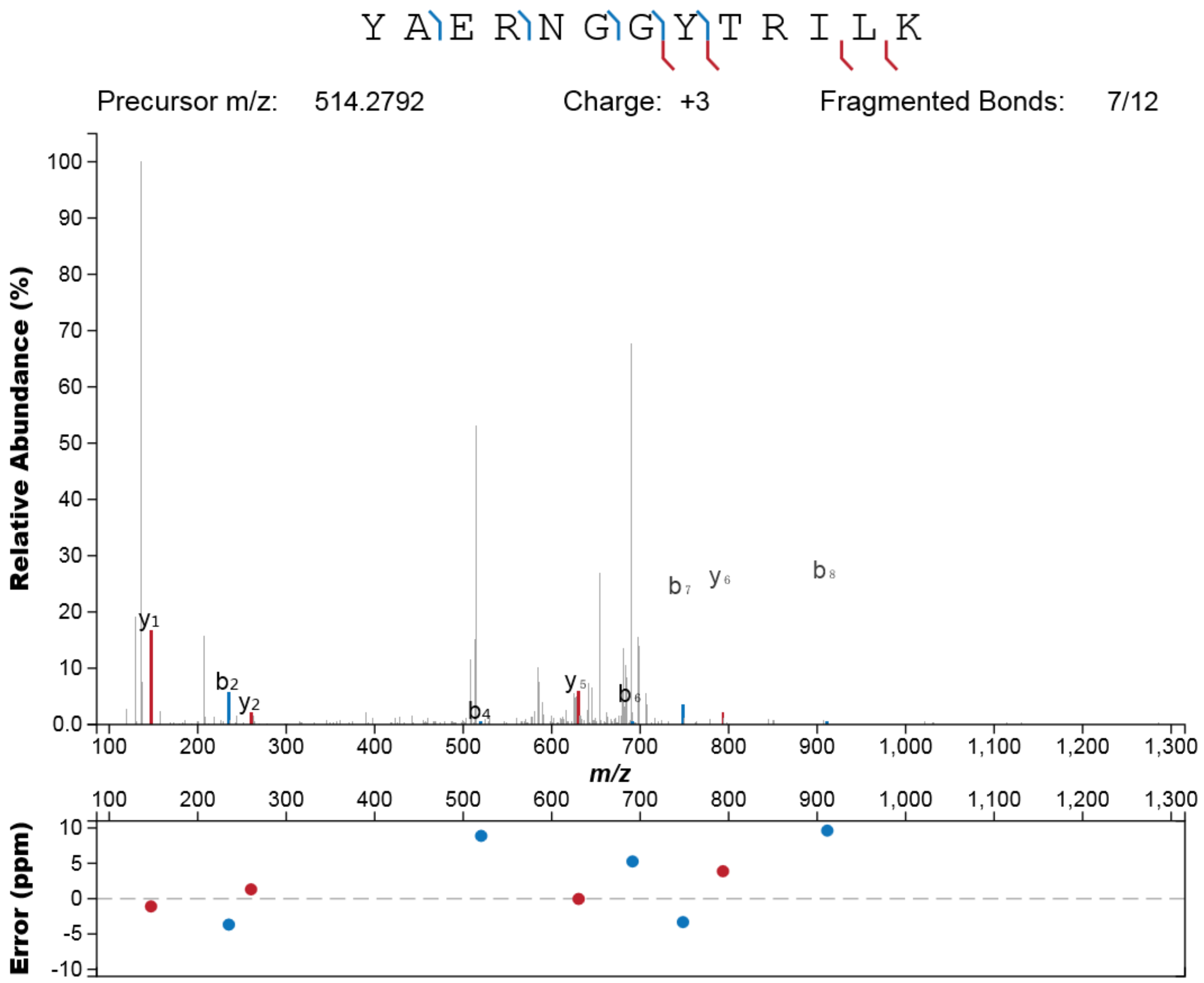

Y A E R N G G Y T R I L K
Precursor m/z: 514.2792
Charge: +3
Fragmented Bonds: 7/12
Relative Abundance (%)
y1
b2
y2
b4
y5
b6
b7
y6
b8
m/z
Error (ppm)

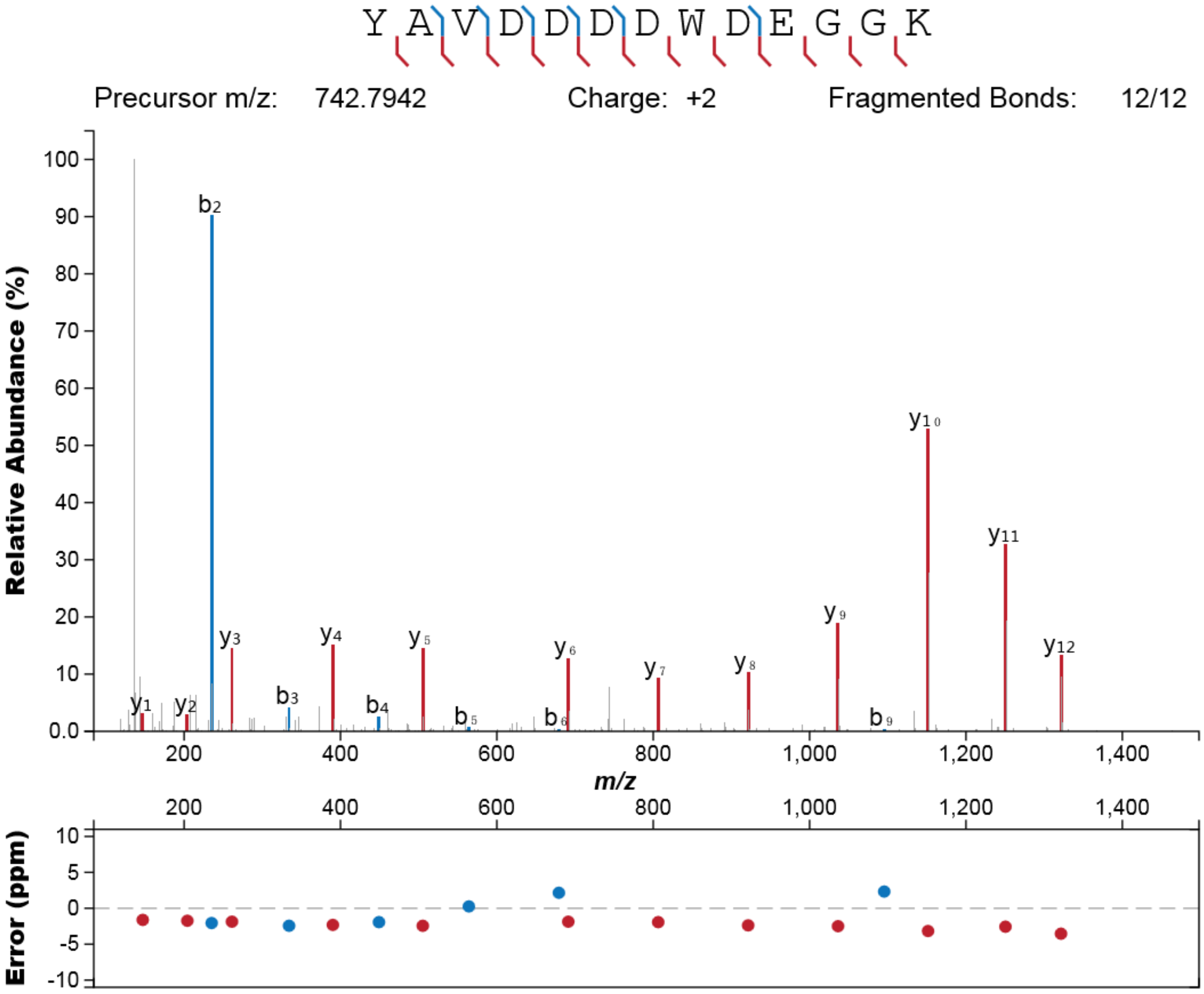

Y A V D D D D W D E G G K
Precursor m/z: 742.7942
Charge: +2
Fragmented Bonds: 12/12
Relative Abundance (%)
m/z
Error (ppm)
b2
y1
y2
y3
b3
y4
b4
y5
b5
y6
b6
y7
y8
y9
b9
y10
y11
y12

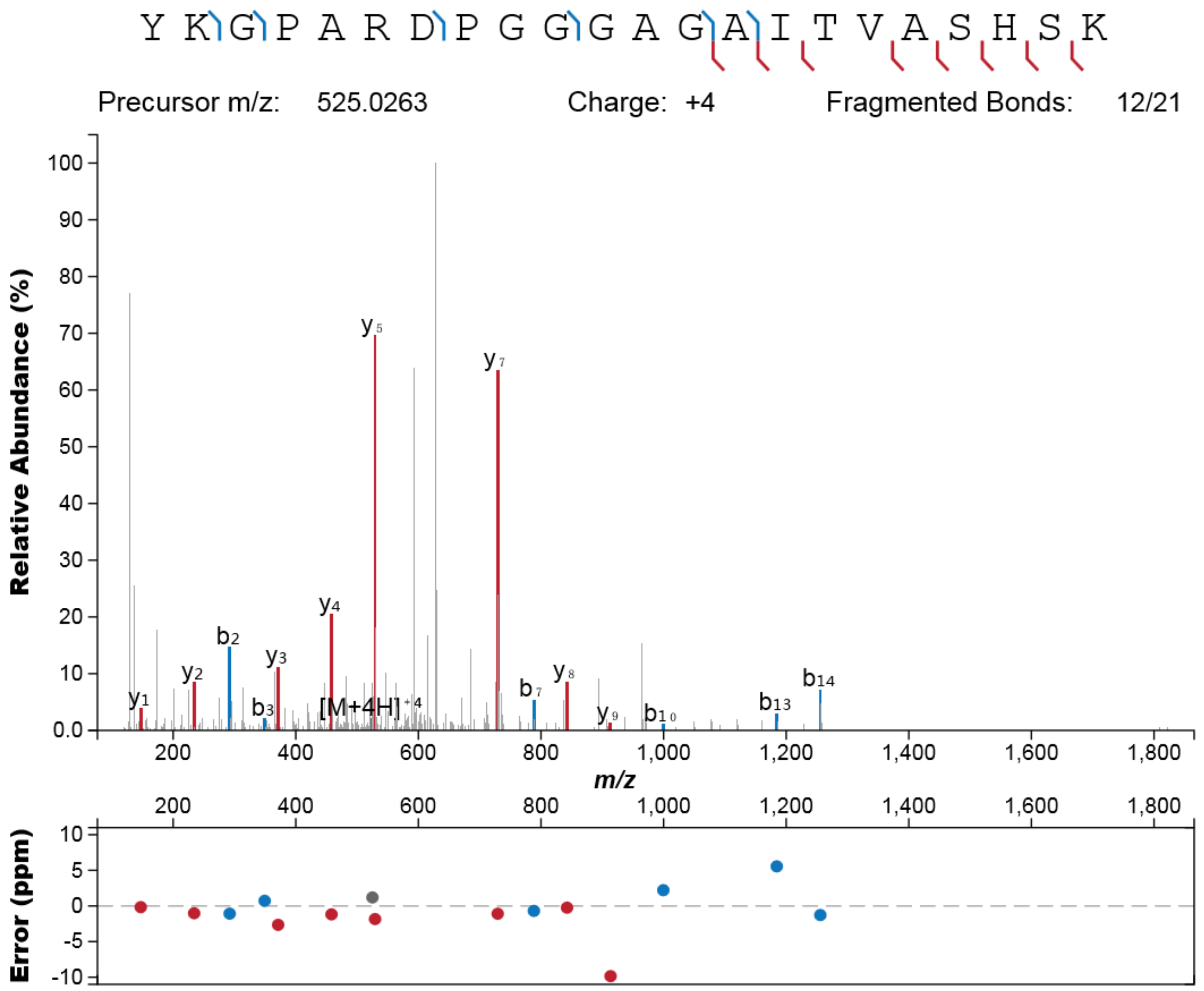

Y K G P A R D P G G G A G A I T V A S H S K
Precursor m/z: 525.0263
Charge: +4
Fragmented Bonds: 12/21
Relative Abundance (%)
m/z
Error (ppm)
y1
y2
b2
b3
y3
y4
[M+4H]+4
y5
y7
b7
y8
y9
b10
b13
b14

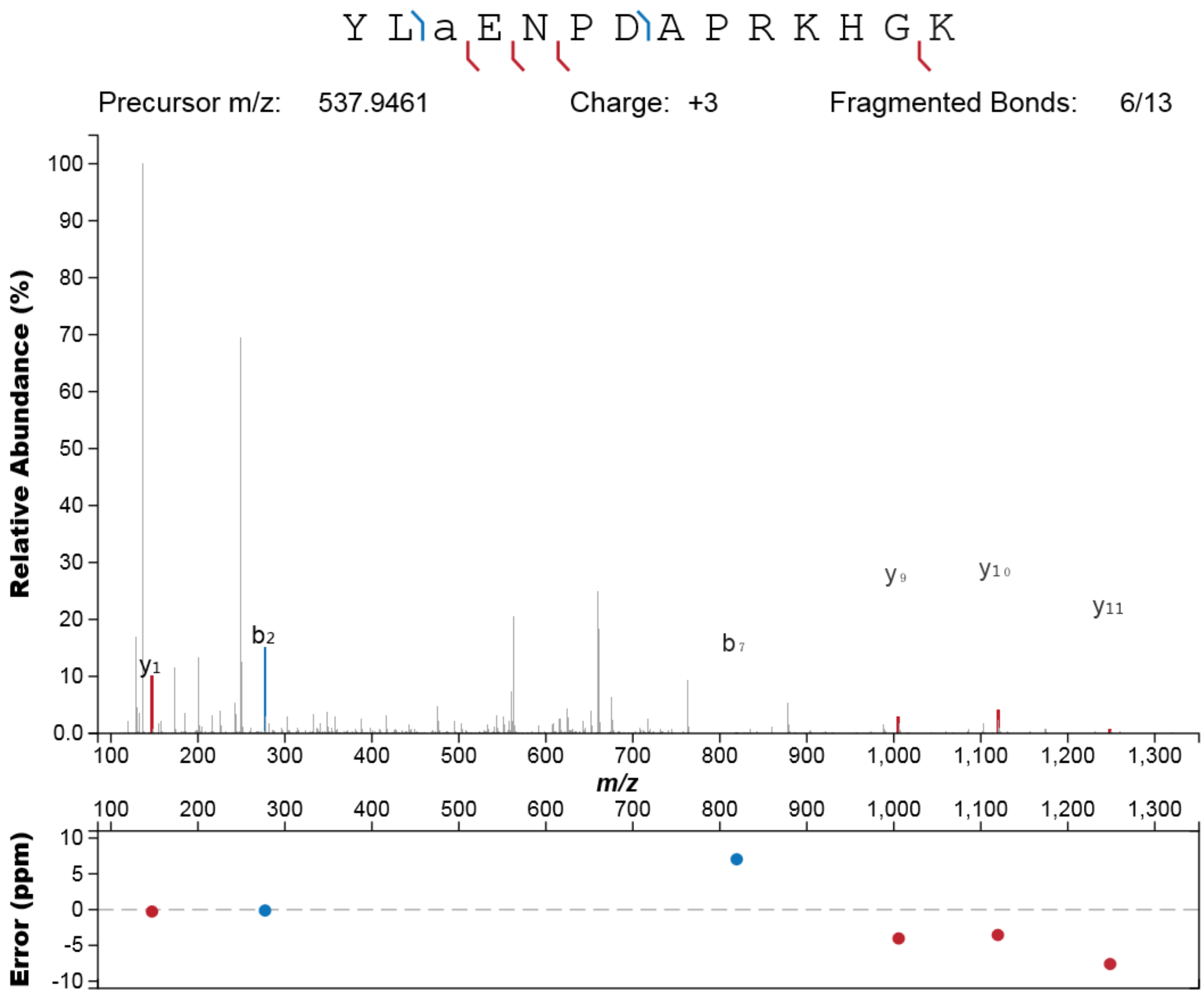
Y L a E N P D A P R K H G K
Precursor m/z: 537.9461
Charge: +3
Fragmented Bonds: 6/13
Relative Abundance (%)
m/z
Error (ppm)
y1
b2
b7
y9
y10
y11

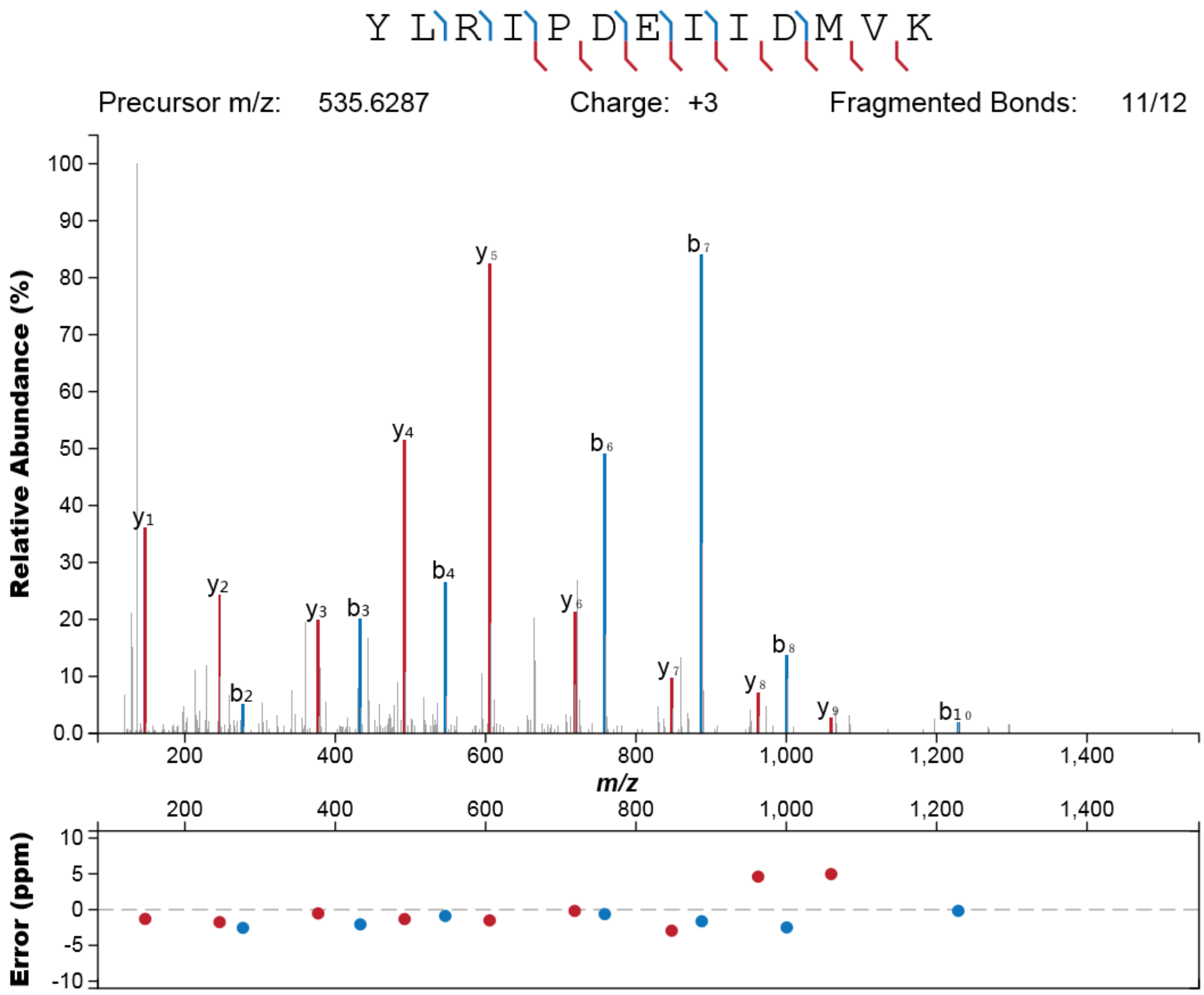

Y L R I P D E I I D M V K
Precursor m/z: 535.6287
Charge: +3
Fragmented Bonds: 11/12
Relative Abundance (%)
m/z
Error (ppm)
y1
y2
b2
y3
b3
y4
b4
y5
y6
b6
y7
b7
y8
b8
y9
b10

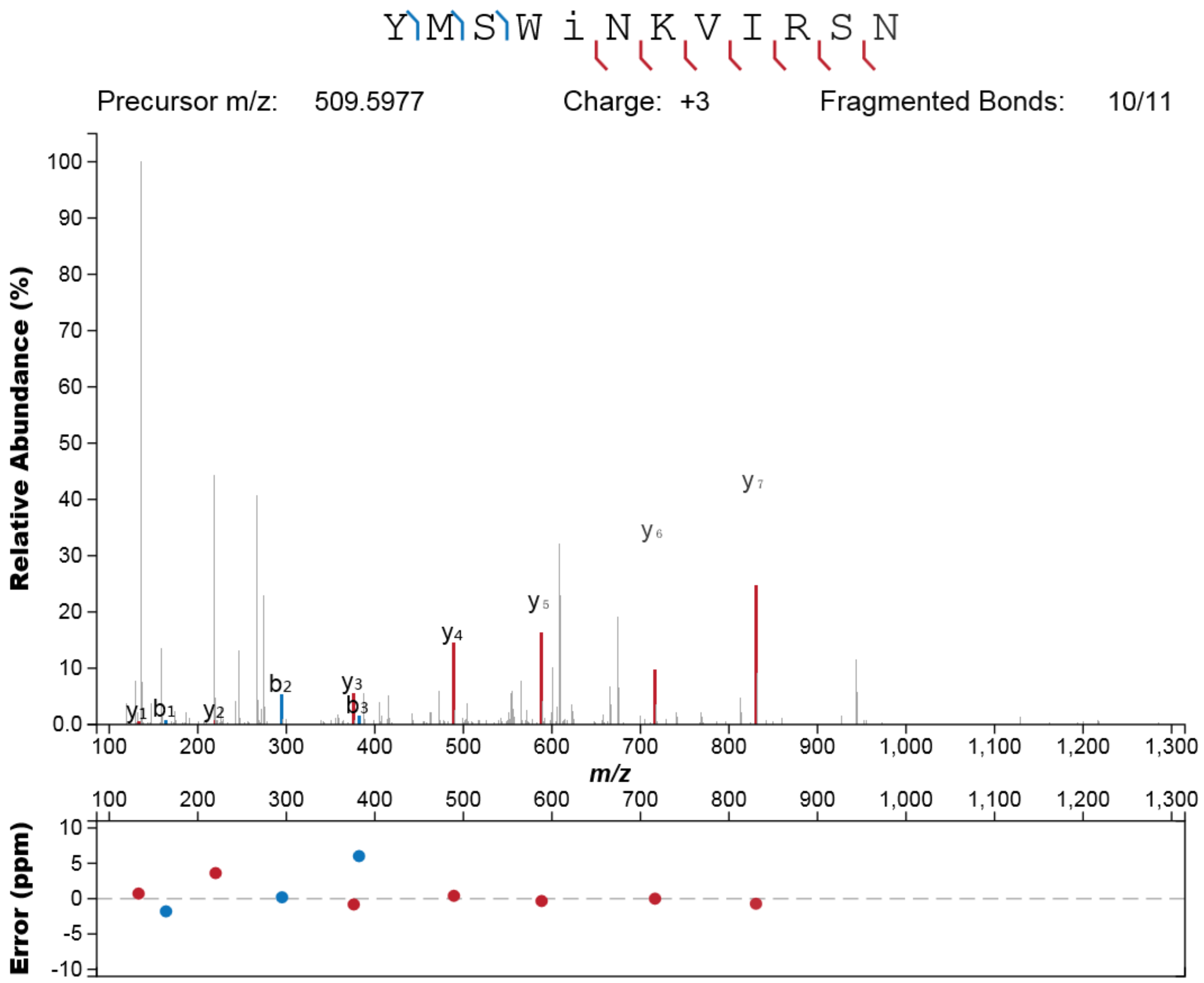

Y M S W i N K V I R S N
Precursor m/z: 509.5977
Charge: +3
Fragmented Bonds: 10/11
Relative Abundance (%)
m/z
Error (ppm)
y1
b1
y2
b2
y3
b3
y4
y5
y6
y7

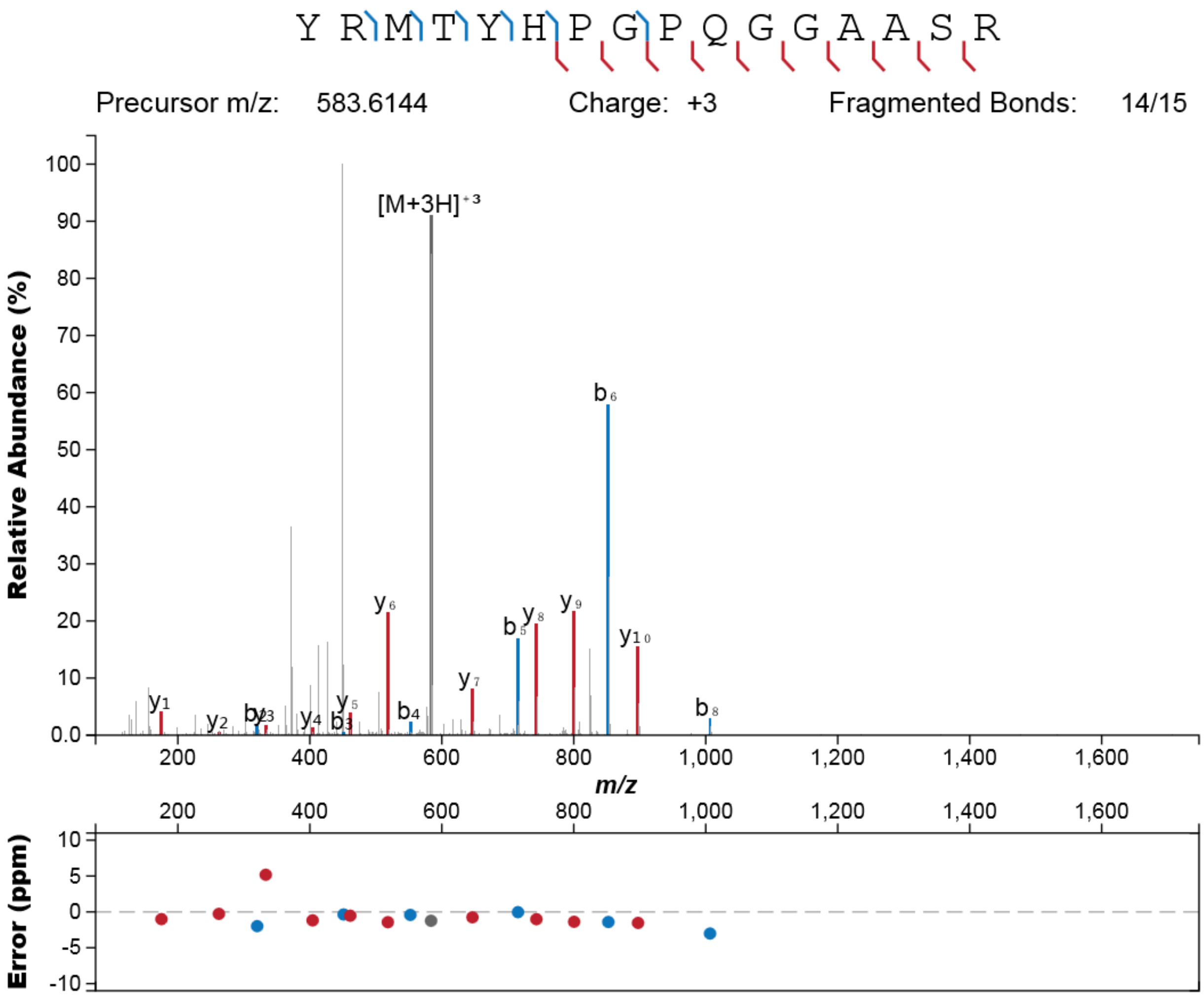

Y R M T Y H P G P Q G G A A S R
Precursor m/z: 583.6144
Charge: +3
Fragmented Bonds: 14/15
[M+3H]+3
Relative Abundance (%)
m/z
Error (ppm)

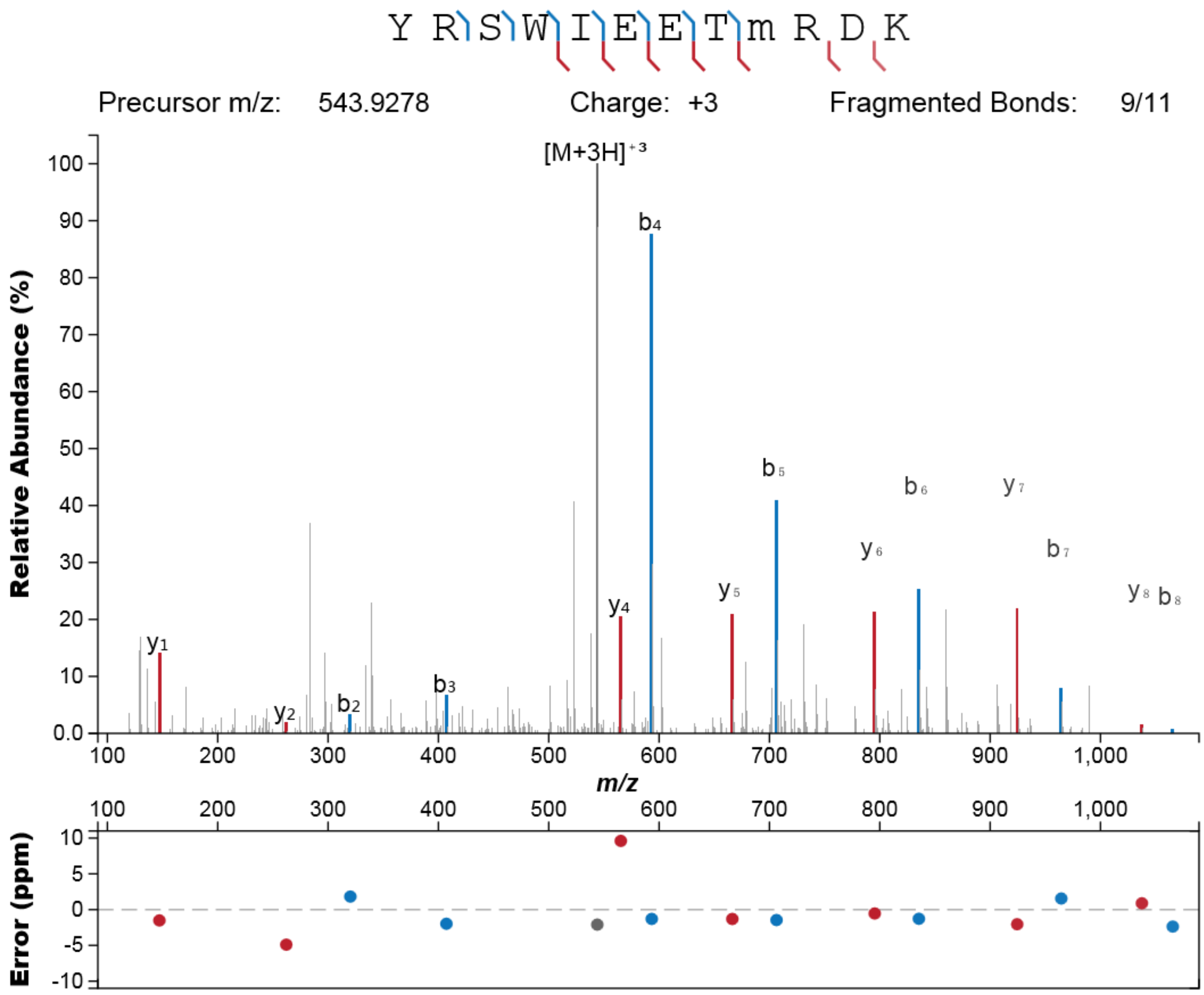

Y R S W I E E T m R D K
Precursor m/z: 543.9278
Charge: +3
Fragmented Bonds: 9/11
[M+3H]+3
Relative Abundance (%)
m/z
Error (ppm)
y1
y2
b2
b3
y4
b4
y5
b5
y6
b6
y7
b7
y8
b8

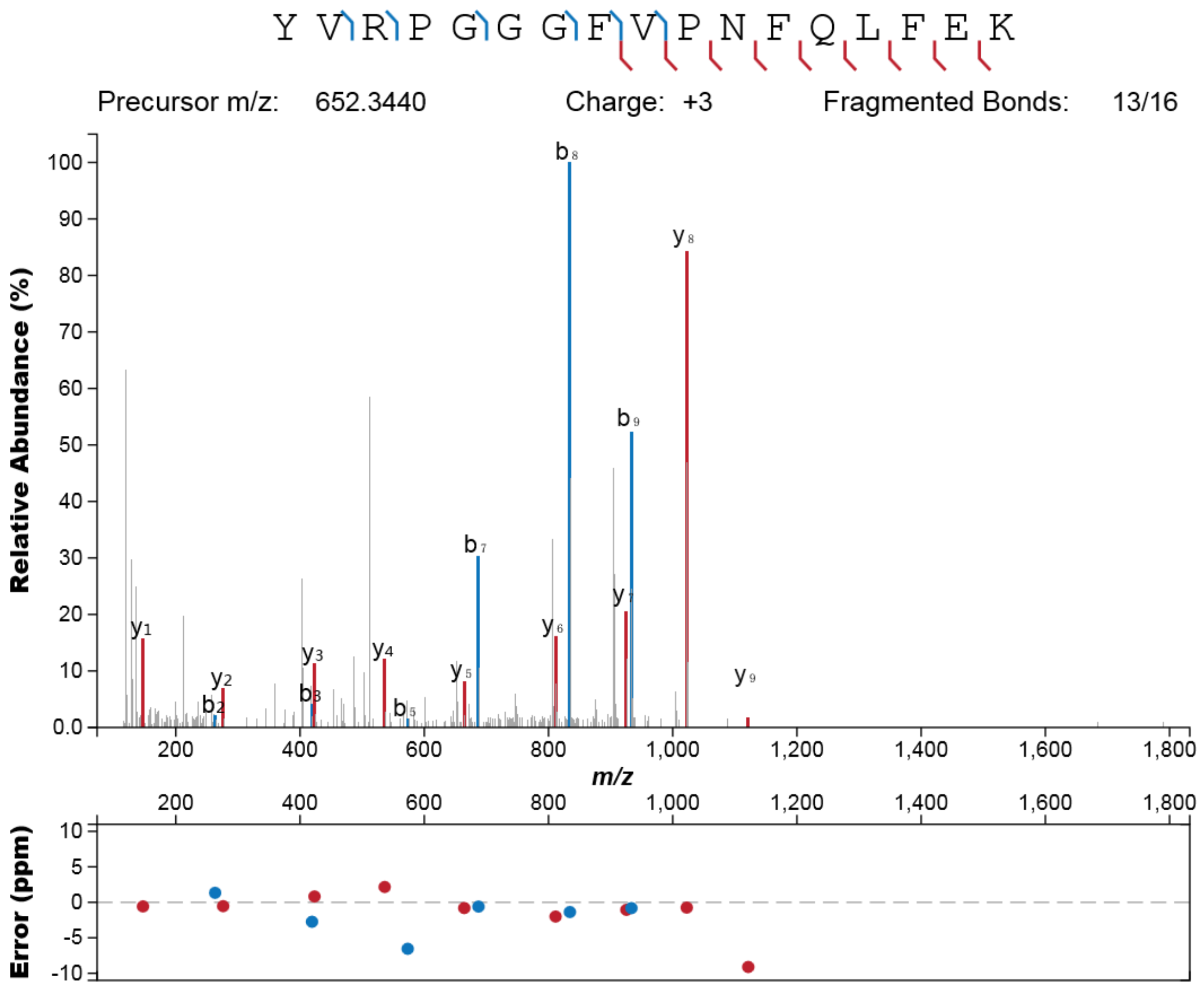

Y V R P G G G F V P N F Q L F E K
Precursor m/z: 652.3440
Charge: +3
Fragmented Bonds: 13/16
Relative Abundance (%)
m/z
Error (ppm)

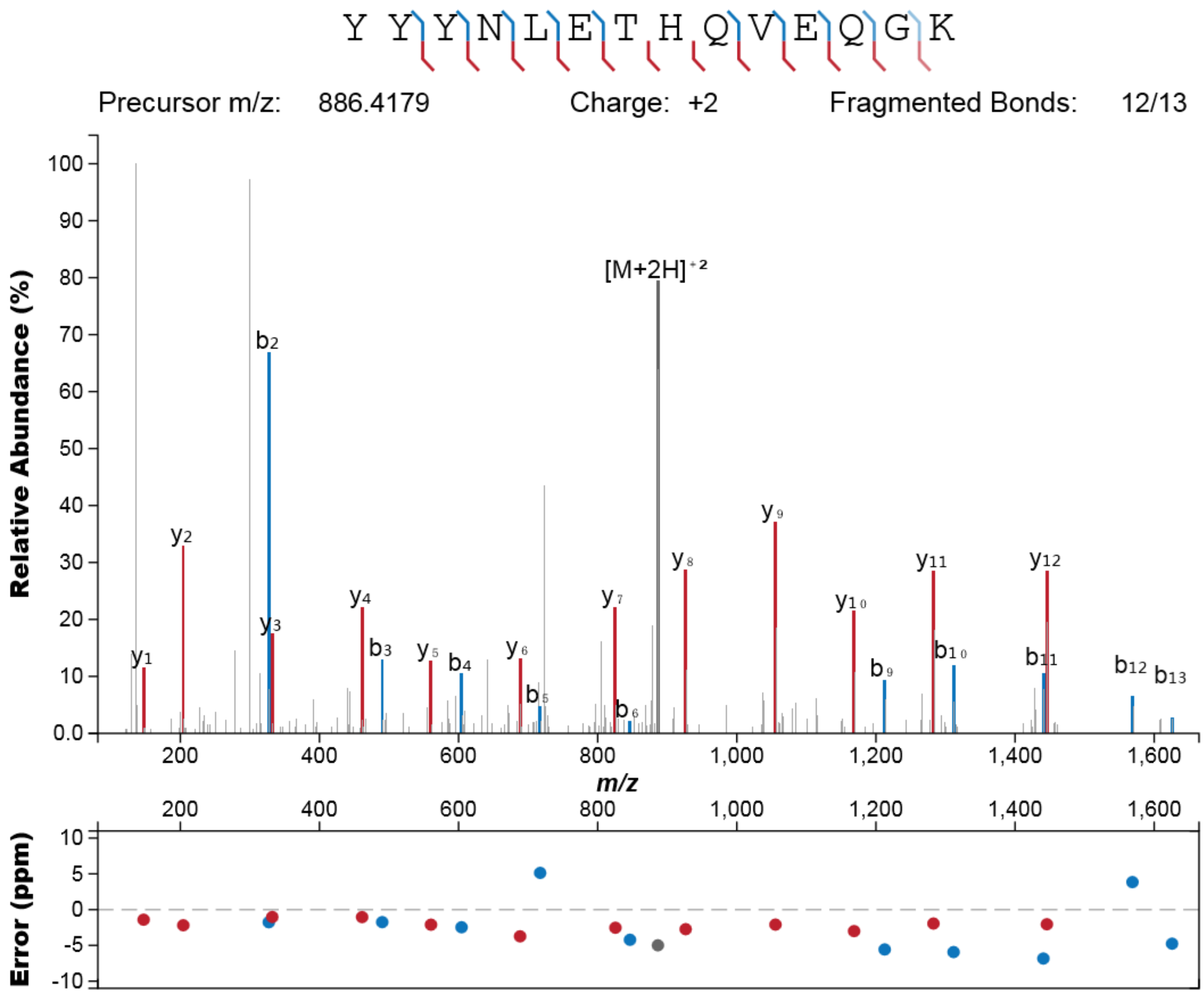

Y Y Y N L E T H Q V E Q G K
Precursor m/z: 886.4179
Charge: +2
Fragmented Bonds: 12/13
Relative Abundance (%)
[M+2H]+2
m/z
Error (ppm)
