# Supplementary material for: Proteomics, Lipidomics, Metabolomics, and 16S DNA Sequencing of Dental Plaque From Patients With Diabetes and Periodontal Disease
Source: Mol Cell Proteomics. 2021 Jul 29;20:100126. doi: 10.1016/j.mcpro.2021.100126 (PMC8426274; doi:10.1016/j.mcpro.2021.100126)
Supplement: Supplemental Figures S1–S4 [file mmc3.pdf]

Supplemental Materials
Multi-omics of dental plaque from patients with diabetes and periodontal disease

Supplemental Figures

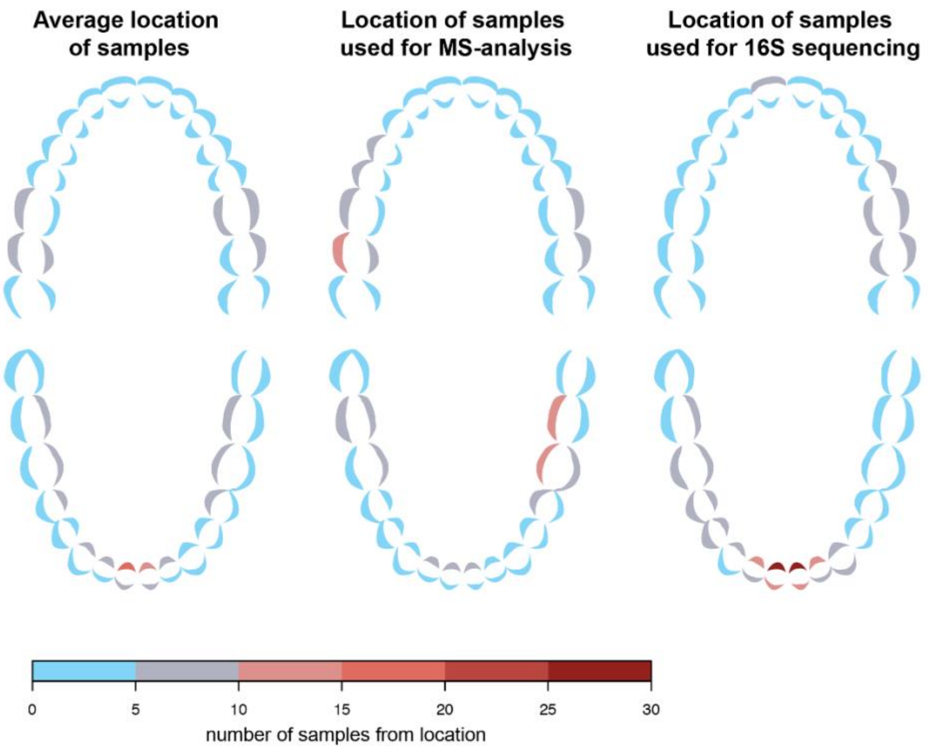

Figure S1. Plaque sample locations used in the study. We obtained three supragingival plaque samples from each patient from various locations. Sample locations are plotted by heat color on a representative teeth diagram, distinguishing upper and lower teeth and buccal and palatal locations.

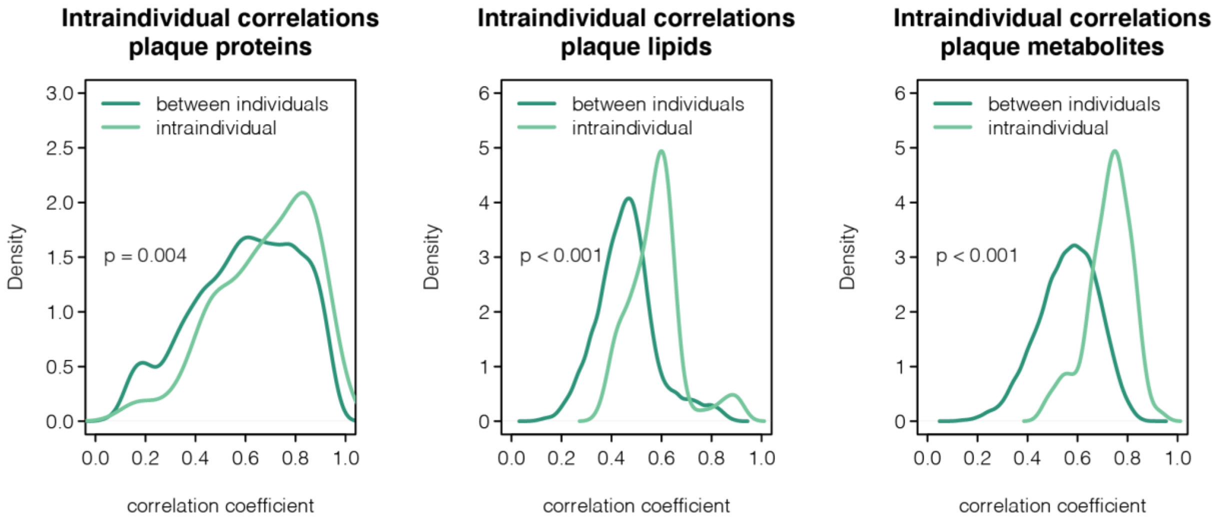

Figure S2. Intra- and inter-individual variations in proteins, lipids, and metabolite abundances. Correlation coefficients were calculated with Kendall rank-based approach, and p-values were calculated using the Kolmogorov–Smirnov test.

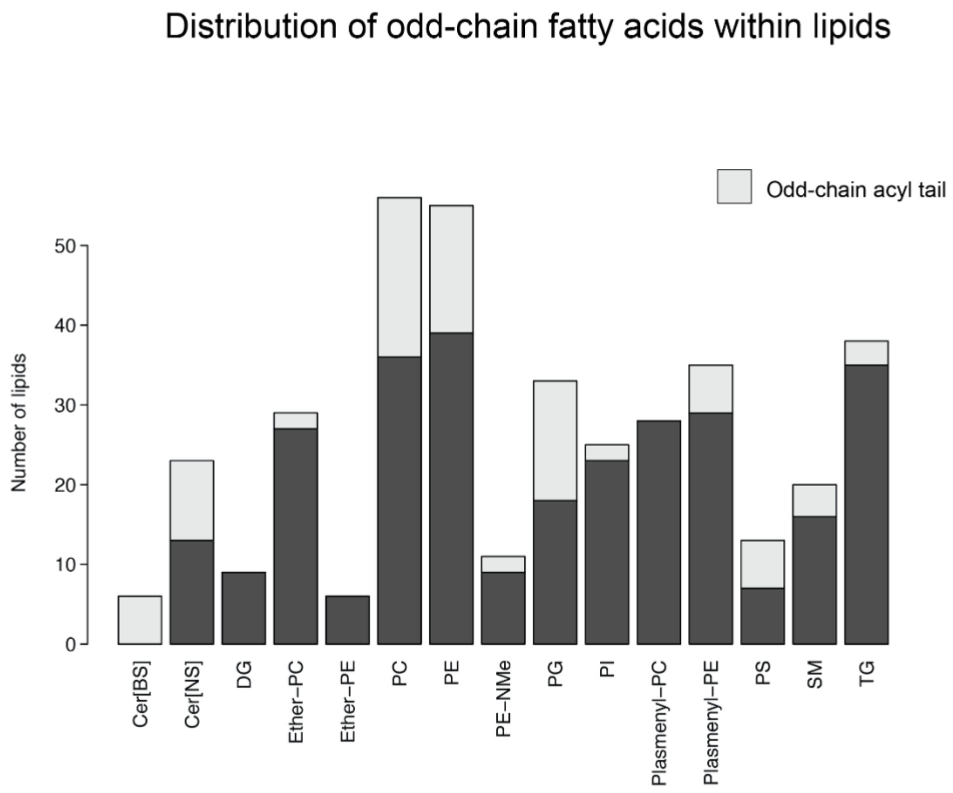

Figure S3. Number of lipids containing odd-chain fatty acids. Odd-chains were counted only if the full specific acyl-chain was odd, or if the total acyl-chain length was odd.

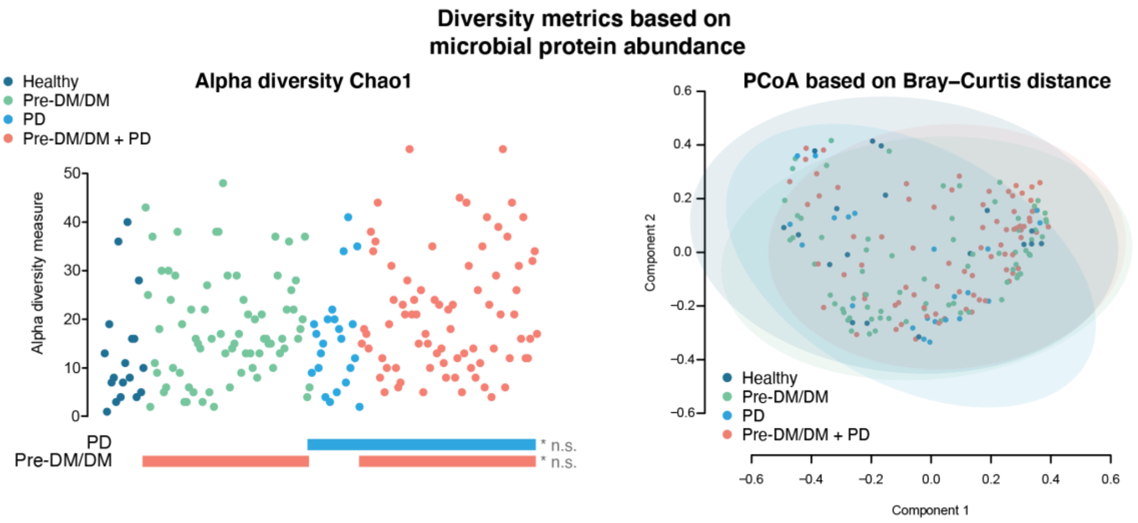

Figure S4. Diversity metrics based on microbial protein abundance. Left panel shows Chao1 calculated based on microbial protein abundance observed in plaque samples (2 per individual); right panel shows the Bray-Curtis distance. PD, periodontal disease; DM, diabetes.
